# Metabolomics Study of the Effect of Transcription Factor NOR-like1 on Flavonoids in Tomato at Different Stages of Maturity Using UPLC-MS/MS

**DOI:** 10.3390/foods12244445

**Published:** 2023-12-11

**Authors:** Di Guan, Ying Zhao, Xiaodan Zhao, Daqi Fu

**Affiliations:** 1 School of Food and Health, Beijing Technology and Business University, Beijing 100048, China; 2 College of Food Science and Nutritional Engineering, China Agricultural University, Beijing 100083, China

**Keywords:** tomato, metabolomics, NOR-like1, flavonoids

## Abstract

Tomato fruits are rich in flavonoids. This study explores the effect of transcription factor SlNOR-like1 on the accumulation of flavonoids in tomato fruits at different ripening stages. We used ultra-pressure liquid chromatography–tandem mass spectrometry (UPLC-MS/MS) to analyze wild-type (WT) and NOR-like1 CRISPR/Cas9-edited (NOR-like1) tomato fruits. A total of 50 flavonoid metabolites were accurately identified and determined in tomatoes. The flavonoid metabolic differences were observed among the different tomato sample groups using PCA and OPLS-DA analysis. There were 16 differential flavonoids (13 upregulated and 3 downregulated) identified between WT-GR (WT tomato at the green-ripening stage) and NOR-like1-GR (NOR-like1 tomato at the green-ripening stage), 9 differential flavonoids (six upregulated and three downregulated) identified between WT-BR3 (WT tomato at the color-breaking stage) and NOR-like1-BR3 (NOR-like1 tomato at the color-breaking stage), and 12 differential flavonoids (11 upregulated and 1 downregulated) identified between WT-BR9 (WT tomato at the red-ripening stage) and NOR-like1-BR9 (NOR-like1 tomato at the red-ripening stage). Rutin, nicotiflorin, naringenin chalcone, eriodictyol, and naringenin-7-glucoside were the five flavonoids with the highest content in the ripening stages (BR3 and BR9) in both WT and NOR-like1 tomato fruits. The overall flavonoid contents in WT tomato fruits changed little from GR to BR3 and decreased from BR3 to BR9; meanwhile, in the NOR-like1 tomato fruits, the total amounts of the flavonoids exhibited an increasing trend during all three ripening stages. The accumulation pattern of flavonoid metabolites in NOR-like1 tomato fruits differed from that in WT tomato fruits, especially in the later ripening process of BR9. The transcription factor SlNOR-like1 has an impact on the accumulation of flavonoids in tomato fruits. The results provide a preliminary basis for subsequent research into its regulatory mechanism and will be helpful for attaining future improvements in the nutritional quality and postharvest treatment of tomato fruits.

## 1. Introduction

Tomatoes contain many flavonoids. As typical and representative polyphenolic compounds, flavonoids have very important functional effects. Flavonoids can effectively improve the stress resistance of plants and resist a variety of biological and abiotic stresses [1,2] occurring in the process of plant growth and development; they also have antioxidant and other effects in animal experiments and human disease studies [3]. Tomato is an important vegetable crop throughout the world, and the study of its methods of genome repair is of great significance for the stable quality of tomato [4]. The ripening of tomatoes leads to significant physiological and biochemical changes, and primary metabolites play crucial roles during fruit development and ripening, improving tomato taste and flavor [5].

**Figure 1:**
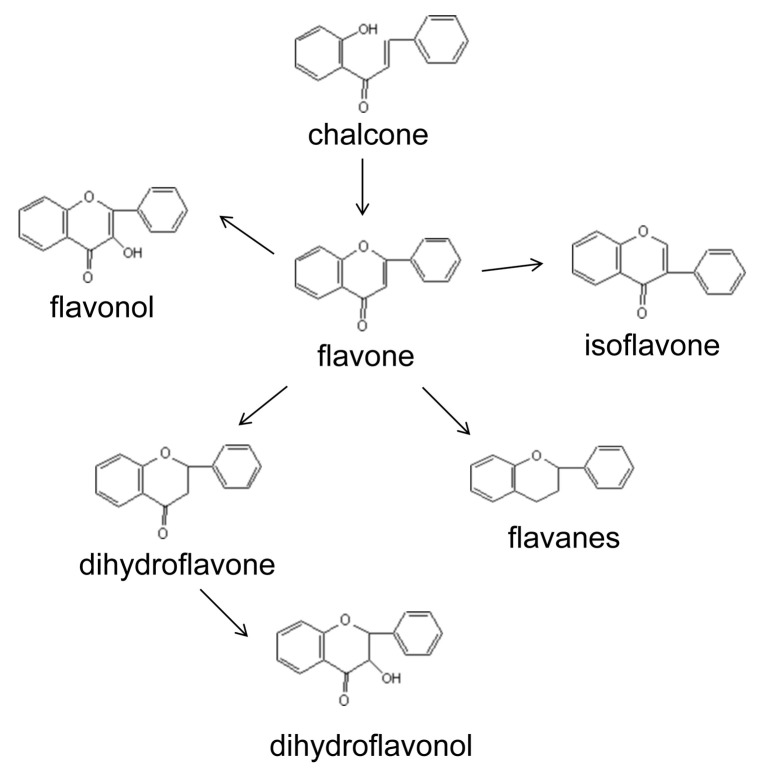
Representative flavonoid derivatives and relationships among their structures.

Flavonoids are secondary metabolites that are modified by a wide range of chemical reactions and are ubiquitous in the biosynthetic pathways in plants [6]. The synthesis of flavonoid compounds, including chalcones, flavonoids, flavonols, anthocyanins, and proanthocyanidins, is a complex process, and their metabolic pathways have been extensively studied using biochemical and molecular biology techniques (Figure 1) [7,8,9].

In recent years, many scholars have conducted extensive research on flavonoid substances in fruits, including their composition, content, detection methods, and related genes [10,11,12,13]. At present, the influence of transcription factors on flavonoids has mainly been focused on the MYB, bHLH, and WD40 transcription factors; research into NAC transcription factors is only in the initial stage, and the function and regulation of *NAC* genes is relatively poorly studied in tomatoes [14]. Previous studies have confirmed that NAC has different expression patterns in different species and have isolated and identified NAC transcription factors in plants to study their effects on plant growth and development, biotic and abiotic stress, and plant metabolites.

The study of transcription factor families is an important area of post-genomic research. The biosynthetic pathways of plant flavonoids comprise actions of structural genes and regulatory genes, and the enzymes in the synthetic pathway are catalyzed by the proteins encoding synthetic structural genes, including *FLS*, *F3H*, *CHS*, *CHI*, *C4H*, etc. Moreover, they are regulated by transcription factors [15].

Huang et al. (2022) identified 183 NAC transcription factors from the radish *OZ-16* genome [16]. The expression patterns of 10 RsNAC genes were verified by means of qPCR, which provided comprehensive information on the radish *NAC* gene family and identified candidate *RsNACs* associated with taproot discoloration. Sun et al. (2019) discovered an NAC transcription factor, *MdNAC52*, which accelerates the expression of this gene when the color of apples deepens, and the overexpression of the *MdNAC52* gene can stimulate the accumulation of anthocyanins [17]. Duan et al. (2020) identified a total of 111 putative NAC transcription factors based on celery transcriptomic and genomic databases. Under field conditions, the overexpression of *SNAC1* significantly improved the drought tolerance of transgenic rice without affecting yield [18,19]. Rice overexpressing *OsNAC10* resulted in higher yields under both normal and field drought conditions [20]. Guo et al. (2022) identified *IbNAC* genes associated with abiotic stress through RNA-seq detection and qRT-PCR analysis, and bioinformatics data supported the prospect of genetically engineering candidate regulators, such as *IbNAC006*, *IbNAC029*, *IbNAC138*, and *IbNAC143*, to improve the stress tolerance of sweet potatoes [21]. Soybean GmNAC06 transcription factor and capsicum CaNAC46 transcription factor were found to be positive regulators of salt stress tolerance [22,23]. Similarly, *SINAC4* and *SINAC1I* genes are involved in the regulation of salt tolerance and early tolerance in tomatoes [24,25].

NOR-like1 is a novel positive regulator of tomato fruit ripening and plays an important role in transcriptional regulatory networks. CRISPR/Cas9 gene editing technology was used to obtain NOR-like1 gene knockout mutants, and it was found that the fruit ripening of NOR-like1 mutant fruits was delayed by 14 days compared with wild-type tomatoes; moreover, the ripening process after color breaking was also significantly inhibited [26]. Compared with the wild-type, fruit softening was significantly inhibited in CR-NOR (CRISPR/Cas9-edited NAC-NOR) but was slightly affected in the NOR mutant and accelerated in overexpressed NAC-NOR (oe-NOR) fruit [27].

In our previous study, we preliminarily explored the effects of the NOR-like1 transcription factor on the profile of metabolites at different stages of tomato maturity using transcriptomics and untargeted metabolomics and found that NOR-like1 significantly affected the changes in flavonoids at different developmental stages [28]. Therefore, the specific influence of the transcription factor SlNORlike1 on tomato flavonoid compounds needs to be further studied. In this study, fruits of wild-type and CRISPR/Cas9-edited lines were collected at different ripening stages: green ripening (GR), color breaking (BR3), and red ripening (BR9). We quantitatively analyzed the flavonoids in the samples. Regression curves of flavonoid standards were established. The flavonoids identified in the sample were fitted to the standard curve, and a total of 50 flavonoids were detected. Then, we analyzed these 50 flavonoids using multivariate analysis. From a paired comparison, we screened out the differential flavonoids between WT and NOR-like1 tomato fruits at each ripening stage and explored the trends in the accumulated flavonoids in WT and NOR-like1 during ripening to understand the effect of NOR-like1 on flavonoids during tomato ripening.

## 2. Materials and Methods

### 2.1. Plant Materials and Sample Preparation

Wild-type tomato Ailsa Craig (AC) and a NOR-like1 tomato transgenic line employing CRISPR/Cas9 gene-editing techniques were both grown in a greenhouse at China Agricultural University. Fruits of wild-type and NOR-like1 fruits were collected at different ripening stages (GR, BR3, and BR9). The sampling method was as described in the previous study [28]. Thus, samples were divided into six groups: WT-GR, WT-BR3, WT-BR9, NOR-like1-GR, NOR-like1-BR3, and NOR-like1-BR9. The samples were ground into powder after freeze-drying. Then, 20 mg of powder was weighed and extracted with 0.5 mL of 70% methanol. [2H6]-Daidzein, [2H3]-rutin, and [13C3]-(+/−)-gallocatechin were used as internal standards, and 10 μL of internal standards (4000 nmol/L) was added into the extract for quantitation. After ultrasonic extraction for 30 min, the extract was centrifuged at 12,000× *g* at 4 °C for 5 min and the supernatant was filtered through a 0.22 μm membrane for further liquid chromatography–tandem mass spectrometry (LC-MS/MS) analysis.

### 2.2. Ultra-Performance Liquid Chromatography (UPLC) Conditions

The sample extracts were analyzed using a UPLC-ESI-MS/MS system, ExionLC™ AD (SCIEX, Framingham, MA, USA) coupled with a QTRAP^®^ 6500+ (SCIEX, Framingham, MA, USA) mass spectrometer, which was based on the description by Zhang et al. [29]. Using [2H6] Daidzein, [2H3] rutin, [13C3]-(+/−)-gallocatechin as internal standard substances, the mixed concentration of these internal standard substances is 4000 nmol/L, with an added scalar of 10 μL and an injection volume of 2 μL. All samples were analyzed using Waters ACQUITY UPLC HSS T3 C18 (100 mm × 2.1 mm i.d., 1.8 µm) at 40 °C. The solvent system was water with 0.05% formic acid as the A phase and acetonitrile with 0.05% formic acid as the B phase. At the beginning of the reaction, gradient elution was set as A/B at 90:10 (*V*/*V*); then, A/B was adjusted to 80:20 (*V*/*V*) after 1 min, 30:70 (*V*/*V*) after 9 min, 5:95 (*V*/*V*) after 12.5 min, and 90:10 (*V*/*V*) after 13.6 min until the end of the reaction. The gradient elution program protocol was performed as described by Wang et al. [30]. The eluent was then connected to an ESI-triple quadrupole-linear ion trap (QTRAP)-MS.

### 2.3. Electrospray Ionization (ESI)–Tandem Mass Spectrometry (MS/MS) (ESI-MS/MS) Conditions

Linear ion trap (LIT) and triple quadrupole (QQQ) scans were conducted on a triple quadrupole-linear ion trap mass spectrometer (QTRAP) equipped with an ESI operating in positive and negative ion mode. The ESI source operation parameters were as follows: an ion source, ESI+/−; the temperature of the electric spray ion source (ESI) was 550 °C; the mass spectrum voltage was 5500 V in the positive ion mode and −4500 V in the negative ion mode; and the curtain gas pressure (CUR) was 35 psi. The Metware Database (MWDB), self-built based on standards, was applied to qualitatively analyze the data from mass spectrometry detection. Quantitative analysis was completed using the Multiple Reaction Monitoring (MRM) mode of triple-quadrupole mass spectrometry. The mass spectrometry data were processed using Analyst 1.6.3 software (SCIEX, Toronto, ON, Canada). MultiQuant 3.0.3 software (SCIEX, Toronto, ON, Canada) was used to quantify all metabolites.

### 2.4. Multivariate Statistical Analysis of Flavonoid Metabolites

Unsupervised principal component analysis (PCA) was performed using the built-in statistical prcomp function of the R software of V3.5.1 version (www.r-project.org/; accessed on 10 February 2022); the data were unit variance scaled before unsupervised PCA was conducted.

The metabolite content data in the cluster analysis were standardized using unit variance scaling (UV), and all samples were subjected to cluster heat map analysis. The R program script was used to draw the cluster heat map.

OPLS-DA was used to screen differential variables by removing uncorrelated differences. Firstly, log_2_-centralization was performed on the data; then, the MetaboAnalystR package (version V1.0.1) in the R language (version V3.5.1) was used for OPLSR (the R package script used for volcanic maps is ggplot2 v3.36), the Anal function was used for OPLS-DA analysis, and, finally, ggplot2 was used to draw the map to obtain OPLS-DA analysis results. Based on the results of OPLS-DA, the variable importance in projection (VIP) and fold change in univariate analysis were combined to perform further screening for differential metabolites with threshold values of fold changes of ≥2 or ≤0.5 and VIP ≥ 1. The annotation results of the significantly different metabolites are classified by pathway enrichment in the KEGG database (https://www.kegg.jp/), illustrating all possible metabolic pathways.

## 3. Results and Discussion

**Figure 2:**
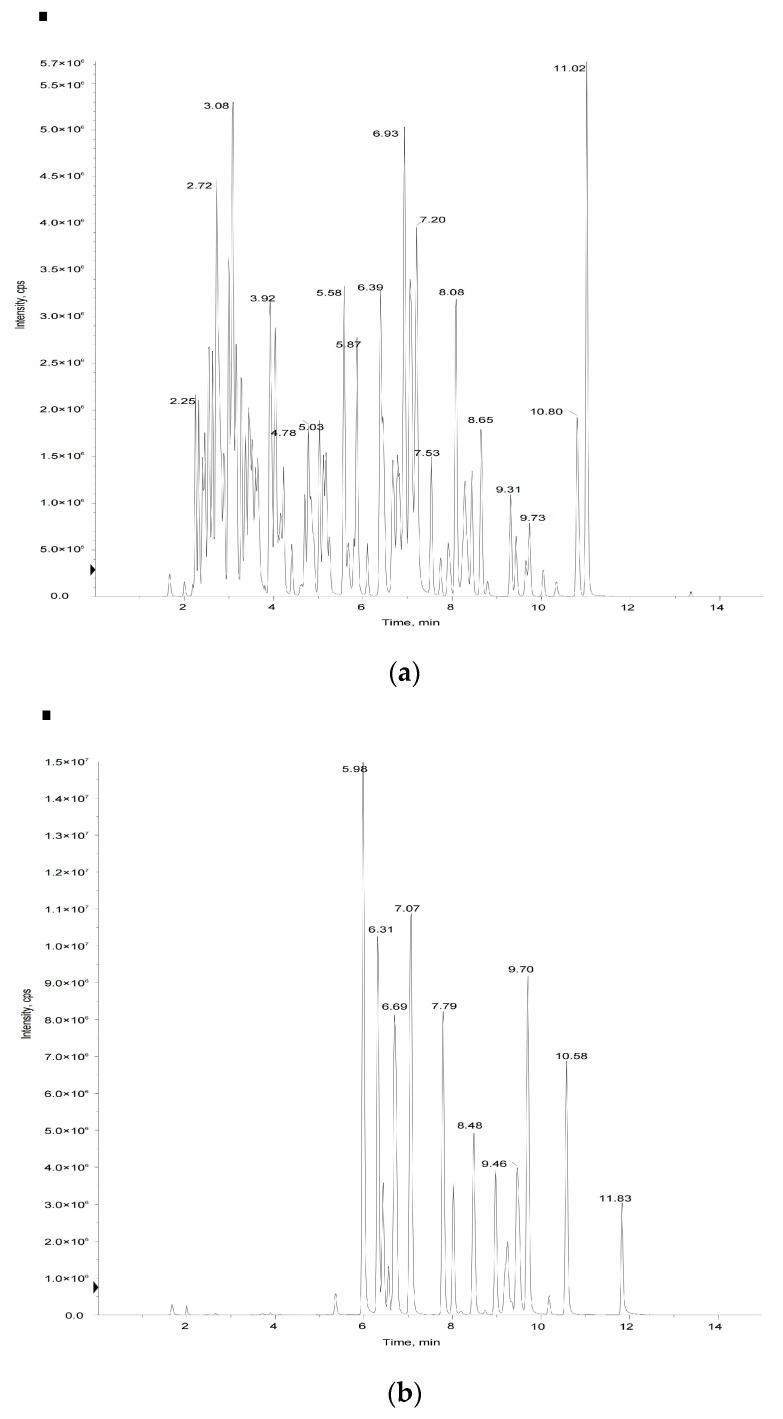
Mass spectrometry quality control (QC) total ion current (TIC) superposition diagram: (**a**) negative-ion TIC diagram, (**b**) positive-ion mode TIC diagram.

In this study, we used metabolomics based on UPLC-MS/MS to determine the flavonoid profiles of tomato fruits. Methods for the accurate determination of flavonoid standards and their regression curves were established. A total of 50 flavonoid metabolites were identified in the tomato samples. Total ion current diagrams detected by mass spectrometry of QC samples are shown in Figure 2. Detailed information on the fifty flavonoid metabolites identified in the tomato fruit samples are in Table S1. MS/MS spectra of the fifty flavonoids identified in the tomato samples are in Supplementary File S1. UHPLC-MS/MS parameters are in Supplementary File S2. Calibration curves and quantitative details of the fifty flavonoids are shown in Supplementary File S3.

### 3.1. Comprehensive Analysis of Flavonoid Compounds

**Figure 3:**
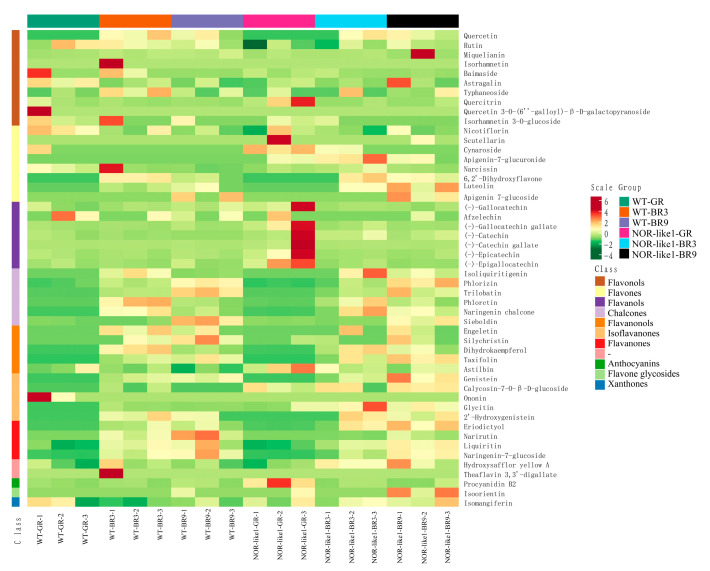
Heatmap visualization of all 50 detected flavonoid metabolites. Each sample has its own column, while each flavonoid is represented by one row. The scale represents the content level obtained after standardization (the redder the color, the higher the content).

The hierarchical cluster analysis (HCA) results of samples and metabolites are presented as heatmaps with dendrograms. In HCA, the normalized signal intensities of metabolites (unit variance scaling) are visualized as a color spectrum. We performed a qualitative and quantitative determination of the metabolites of the flavonoids in tomato fruits; 50 flavonoid metabolites in the fruit samples were identified and quantitatively determined, including 6 chalcones, 8 flavonols, 4 flavanones, 5 flavanonols, 1 flavone glycosides, 8 flavones, 10 flavonols, 6 isoflavones, 1 xanthone, and 1 other flavonoid (Figure 3).

### 3.2. Differential Flavonoid Metabolites Based on Principal Component Analysis (PCA)

**Figure 4:**
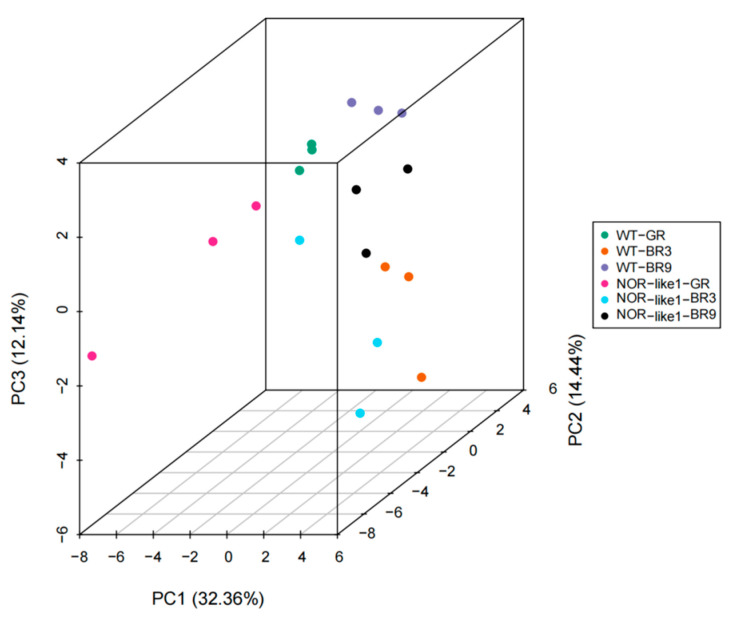
PCA analysis of the flavonoid metabolites in the tomato fruits of the six sample groups (3D image). Points with different colors represent different sample groups.

In the present study, the three principal components—PC1, PC2, and PC3—accounted for 82.69%, 6.2%, and 3.59% of the variability in the samples, respectively (Figure 4). The six different sample groups were clearly separated. The PCA results demonstrate a significant trend for the separation of the flavonoid metabolomes between the groups and indicate the difference in metabolites between each group of samples.

### 3.3. Screening and Analysis of Differential Flavonoid Metabolites

To analyze the trends in the changes in flavonoid metabolites during the fruit ripening in WT and NOR-like1 tomato fruit, differential flavonoid metabolites were screened and analyzed through a paired comparison. All flavonoids detected in tomato fruits were screened to identify differential metabolites with fold a change ≥2 or ≤0.5 and VIP ≥ 1, which are represented in volcano maps. The results of the paired comparison are shown in Table 1. The contents of 50 flavonoids at different stages are detailed in Table 2.

#### 3.3.1. Orthogonal Projections to Latent Structures Discriminant Analysis (OPLS-DA) of Different Tomato Sample Groups

**Figure 5:**
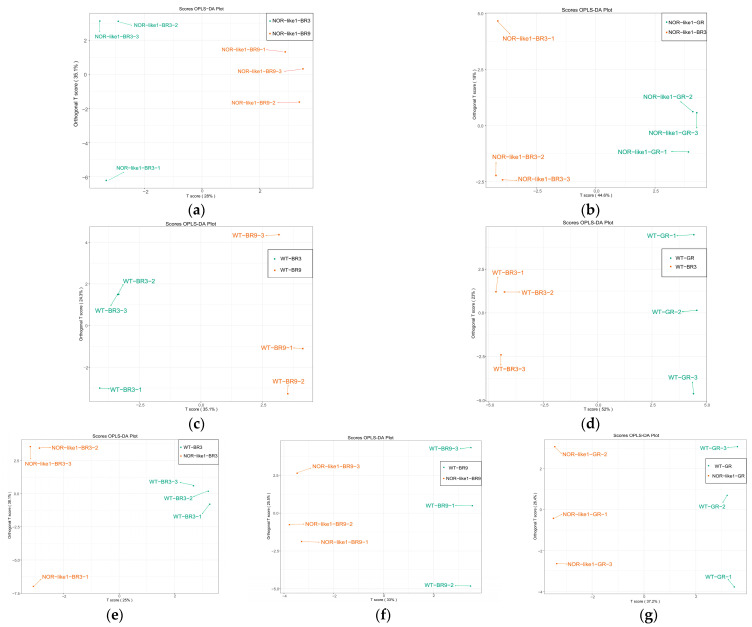
OPLS-DA model plots and loading plots of pairwise comparisons between the different tomato sample groups: (**a**) NOR-like1-BR3 vs. NOR-like1-BR9 (R^2^X = 0.786, R^2^Y = 0.993, Q^2^ = 0.63); (**b**) NOR-like1-GR vs. NOR-like1-BR3 (R^2^X = 0.626, R^2^Y = 0.999, Q^2^ = 0.822); (**c**) WT-BR3 vs. WT-BR9 (R^2^X = 0.594, R^2^Y = 0.989, Q^2^ = 0.771); (**d**) WT-GR vs. WT-BR3 (R^2^X = 0.75, R^2^Y = 0.999, Q^2^ = 0.943); (**e**) WT-BR3 vs. NOR-like1-BR3 (R^2^X = 0.755, R^2^Y = 0.955, Q^2^ = 0.632); (**f**) WT-BR9 vs. NOR-like1-BR9 (R^2^X = 0.585, R^2^Y = 0.998, Q^2^ = 0.752); (**g**) WT-GR vs. NOR-like1-GR (R^2^X = 0.726, R^2^Y = 0.999, Q^2^ = 0.891).

Pairwise comparisons were carried out using Orthogonal Partial Least Squares–Discriminant Analysis (OPLS-DA) models to clarify the metabolic differences observed among the different tomato sample groups. The parameters of the OPLS-DA evaluation model include R^2^X, R^2^Y, and Q^2^, where R^2^X and R^2^Y represent the explanatory power of the constructed model to the X and Y matrices. Q^2^ represents the predictive ability of the model. Q^2^ > 0.5 is generally considered to indicate an acceptable model and, theoretically, the closer the values of R^2^ and Q^2^ to 1, the better the model [31]. The OPLS-DA results of the pairwise comparison between groups are shown in Figure 5. In the results, the R^2^ values of all the comparison groups were above 0.5, which indicated a good fit for the model. Different tomato groups were distributed in different zones, implying that there were significant differences in their metabolites.

#### 3.3.2. Changes in Flavonoid Metabolites in WT Tomato Fruits at Different Ripening Stages

**Figure 6:**
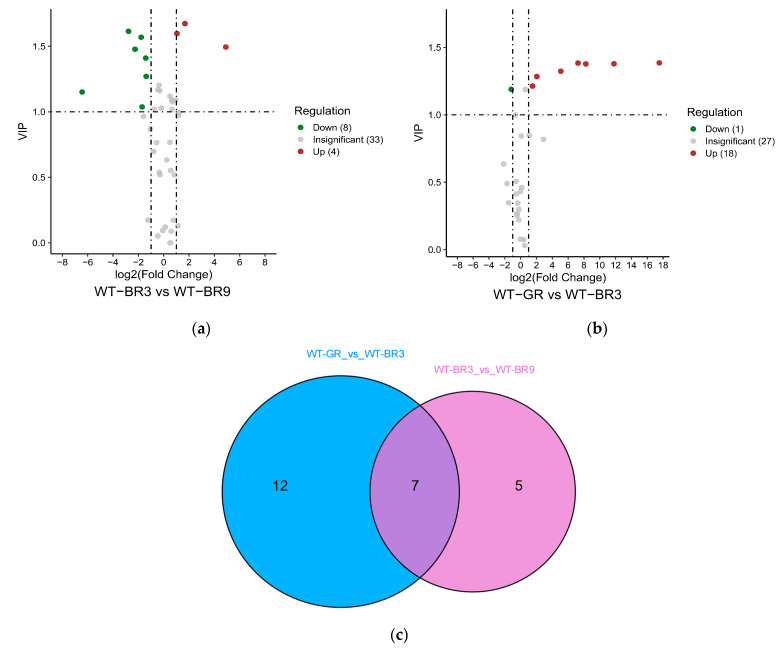
The differential metabolite analysis results in different ripening stages in WT tomato fruit: (**a**,**b**) The volcano plot shows the differential metabolite expression levels between WT-GR vs. WT-BR3 and WT-BR3 vs. WT-BR9, respectively. (**c**)The Venn diagram shows the differential metabolites from different ripening stages in WT tomato fruits.

The expression of metabolites varied at different growth stages of wild tomato fruits. Twelve differential metabolites (four upregulated and eight downregulated) were identified between WT-BR3 and WT-BR9 (Figure 6a). Nineteen differential metabolites (eighteen upregulated and one downregulated) were identified between WT-GR and WT-BR3 (Figure 6b).

The Venn diagram shows that there were seven common differential metabolites in the two combinations: 6,2′-dihydroxyflavone, dihydrokaempferol, phloretin, naringenin chalcone, trilobatin, isoliquiritigenin, and phlorizin (Figure 6c).

The individual metabolite contents are shown in Table 2. The total contents of all the flavonoids in WT-GR, WT-BR3, and WT-BR9 were 281.02 mg/kg DW, 276.25 mg/kg DW, and 223.37 mg/kg DW, respectively. Rutin, nicotiflorin, naringenin chalcone, eriodictyol, and naringenin-7-glucoside were the five flavonoids with the highest content in the ripening stages (BR3 and BR9) of the WT tomato fruits.

Rutin and nicotiflorin were the two richest components in WT-GR. Rutin changed insignificantly, while nicotiflorin decreased from GR to BR3. The content of naringenin chalcone increased to 28.46 mg/kg DW in BR3, indicating its biosynthesis was very active in this period.

The contents of some other flavonoids also changed during the ripening stages. Phlorizin and trilobatin increased significantly throughout the entire ripening process. The contents of nine flavonoids (luteolin, liquiritin, eriodictyol, narirutin, engeletin, genistein, glycitin, quercetin, and taxifolin) increased significantly from the GR stage to the BR3 stage but changed insignificantly from the BR3 stage to the BR9 stage. The contents of 6,2′-dihydroxyflavone, dihydrokaempferol, phloretin, naringenin chalcone, and isoliquiritigenin increased significantly from the GR stage to the BR3 stage but declined significantly from the BR3 stage to the BR9 stage. These results showed that the contents of a variety of flavonoids increased significantly in the GR stage to the BR3 stage, which indicated that flavonoid metabolite biosynthesis is very active in this period.

In contrast, in the later ripening process BR9, the differential flavonoid metabolites in WT-BR9 tomato fruits were mostly downregulated compared with those in WT-BR3. Astragalin declined significantly during the entire ripening process of WT tomato fruits. Narcissin, baimaside, and sieboldin changed little from GR to BR3 but decreased from BR3 to BR9. In WT-BR3, rutin, nicotiflorin, and naringenin chalcone were the three predominant components, while their contents all decreased from BR3 to BR9. Taken together, the decreased contents of the three main flavonoids and other downregulated flavonoid metabolites lead to a lower total flavonoid content in WT-BR9 than in WT-BR3.

#### 3.3.3. Change in Flavonoid Metabolites in NOR-like1 Tomato Fruits at Different Ripening Stages

**Figure 7:**
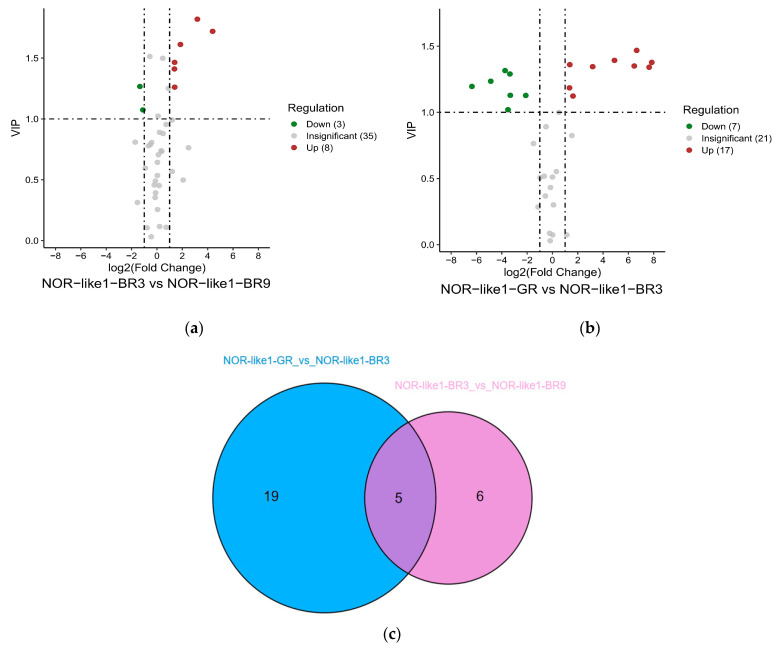
The differential metabolite analysis results at different ripening stages in NOR-like1 tomato fruits: (**a**,**b**) Volcano plot showing the differential metabolite expression levels among NOR-like1-GR vs. NOR-like1-BR3 and NOR-like1-BR3 vs. NOR-like1-BR9, respectively. (**c**) Venn diagram showing the differential metabolites from different ripening stages in NOR-like1 tomato fruits.

To investigate the changes in flavonoid metabolites among different ripening stages of NOR-like1 tomato fruits, differentially accumulated flavonoid metabolites were analyzed among NOR-like1-GR vs. NOR-like1-BR3 and NOR-like1-BR3 vs. NOR-like1-BR9. Eleven differential metabolites (eight upregulated and three downregulated) were identified between NOR-like1-BR3 and NOR-like1-BR9 (Figure 7a). Twenty-four differential metabolites (seventeen upregulated and seven downregulated) were identified between NOR-like1-GR and NOR-like1-BR3 (Figure 7b).

The Venn diagram shows that two combinations have five common differential metabolites (genistein, naringenin-7-glucoside, phloretin, trilobatin, and phlorizin) (Figure 7c).

The total contents of all the flavonoids in NOR-like1-GR, NOR-like1-BR3, and NOR-like1-BR9 were 176.87 mg/kg DW, 214.39 mg/kg DW, and 245.93 mg/kg DW, respectively. Rutin, nicotiflorin, naringenin chalcone, eriodictyol, and naringenin-7-glucoside were also the five flavonoids with the highest content in the ripening stages (BR3 and BR9) in NOR-like1 tomato fruits.

Rutin and nicotiflorin were the two dominant flavonoids among the NOR-like1-GR fruits. From GR to BR3, the contents of rutin and naringenin chalcone increased to 125.43 mg/kg DW and 29.6 mg/kg DW in BR3, respectively, while nicotiflorin decreased from 68.36 mg/kg DW to 46.47 mg/kg DW. Moreover, there were seventeen upregulated flavonoid metabolites between NOR-like1-GR and NOR-like1-BR3 (Figure 7b). The results showed that flavonoid metabolites accumulated in large amounts from the GR stage to the BR3 stage in NOR-like1 tomato fruits. During this period, flavonoid metabolite biosynthesis is very active. The increased contents of the two main flavonoids (rutin and naringenin chalcone) and other upregulated flavonoid metabolites lead to a higher total of flavonoid content in NOR-like1-BR3 than that in NOR-like1-GR.

From BR3 to BR9, the contents of rutin, nicotiflorin, eriodictyol, and naringenin-7-glucoside increased to 145.01 mg/kg DW, 60.35 mg/kg DW, 11.84 mg/kg DW, and 3.72 mg/kg DW, respectively. The content of these four components comprised more than 90% of the total content of all the flavonoids in NOR-like1-BR9. Naringenin chalcone decreased slightly from 29.6 to 21.6 mg/kg DW. The contents of the other flavonoids also changed from BR3 to BR9. Among them, eight were upregulated and three were downregulated. The increased contents of the four dominant flavonoids and the eight upregulated flavonoids led to an increase in the total flavonoid content in NOR-like1-BR9 than that in NOR-like1-BR3.

#### 3.3.4. Differential Flavonoid Metabolites between WT and NOR-like1 Tomato Fruits

**Figure 8:**
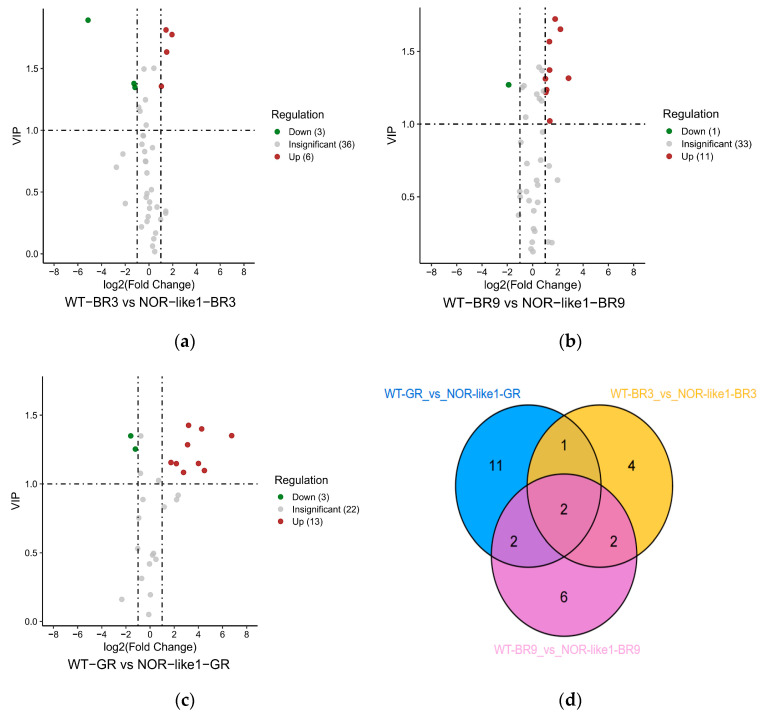
Analysis of differential metabolites between WT and NOR-like1 tomato fruits at the same ripening stage: (**a**–**c**) The volcano plot shows the expression levels of the differential metabolites among WT-BR3 vs. NOR-like1-BR3, WT-BR9 vs. NOR-like1-BR9, and WT-GR vs. NOR-like1-GR, respectively. (**d**) The Venn diagram shows the differential metabolites between WT and NOR-like1 tomato fruits at the same ripening stage.

In order to explore the differences in the flavonoid metabolites at different growth stages of the two varieties of tomato fruits, differential metabolites were screened using a pairwise comparison. Figure 8a–c presents Volcano plots showing the levels of differential metabolites between WT and NOR-like1 at different maturity stages. Detailed information about the contents of flavonoid metabolites detected in each tomato fruit sample is shown in Table 2.

During the BR3 stage, nine differential flavonoids (six upregulated and three downregulated) were identified between WT-BR3 and NOR-like1-BR3 (Figure 8a). During the BR9 stage, 12 differential metabolites (11 upregulated and 1 downregulated) were identified between WT-BR9 and NOR-like1-BR9 (Figure 8b). During the GR stage, 16 differential metabolites (13 upregulated and 3 downregulated) were identified between WT-GR and NOR-like1-GR (Figure 8c).

The Venn diagram shows that there were two common differential metabolites (glycitin and apigenin-7-glucuronide) in the three combinations (Figure 8d), three common differential metabolites (glycitin, apigenin-7-glucuronide, and cynaroside) in the combination of WT-GR_vs_NOR-like1-GR and WT-BR3_vs_NOR-like1-BR3, four common differential metabolites (glycitin, naringenin chalcone, apigenin-7-glucuronide, and calycosin-7-O-β-D-glucoside) in the combination of WT-BR9_vs_NOR-like1-BR9 and WT-BR3_vs_NOR-like1-BR3, and four common differential metabolites (glycitin, apigenin-7-glucuronide, narirutin, and (-)-catechin) in the combination of WT-GR_vs_NOR-like1-GR and WT-BR9_vs_NOR-like1-BR9. Apigenin-7-glucuronide was detected only in NOR-like1 tomato fruits in all three maturation stages and not in the WT. The contents of glycitin in NOR-like1 tomato fruits were significantly higher than those in the WT in all three maturation stages.

Compared to the WT tomato fruits, most of the flavonoids were significantly changed in NOR-like1 tomato fruits during the ripening stages, indicating that SlNOR-like1 does regulate the biosynthesis of flavonoids. In our previous study, by using untargeted metabolomics analysis to study the metabolic profile of NOR-like1 gene-edited tomato fruits with WT tomato fruits for comparison, it was found that flavonoid metabolites changed significantly during the whole ripening stages [28], which corresponded to the results here.

The total amount of all the flavonoids in NOR-like1 tomato fruits was 176.87 mg/kg DW in the GR stage and increased to 214.39 mg/kg DW in BR3 and 245.93 mg/kg DW in BR9. The total amount of all the flavonoids in WT tomato fruits was 281.02 mg/kg DW in the GR stage, which slightly decreased to 276.27 mg/kg DW in BR3 and then to 223.37 mg/kg DW in BR9. The total amount of all the flavonoids in NOR-like1 tomato fruits was lower than in WT tomato fruits during the GR stage and BR3 stage. However, during the BR9 stage, the total amount of all the flavonoids in NOR-like1 tomato fruits was higher than in WT tomato fruits. The dynamic changes of the flavonoids differed among the two different tomato strains.

To explore the reason causing such a difference, we compared the contents of the predominant flavonoid compounds and their changes in two different tomato strains and found that rutin, nicotiflorin, naringenin chalcone, eriodictyol, and naringenin-7-glucoside were the five flavonoids with the highest content in the ripening stages (BR3 and BR9) in both WT and NOR-like1 tomato fruits. However, the accumulation patterns of some flavonoids differed between the WT and NOR-like1 tomato fruits. Rutin was the most abundant and significantly variable. Rutin has been extensively detected in tomatoes in multiple literature studies [32,33,34,35,36,37,38,39]. In our study, the content of rutin in WT tomato fruits was highest during the GR stage (175.26 mg/kg DW), and its content in NOR-like1-GR tomato fruits was 101.18 mg/kg DW. However, during the subsequent growth process, the content of rutin gradually increased in NOR-like1 tomato fruits but decreased in the WT. In BR9, the content of rutin in NOR-like1 tomato fruits was 145.01 mg/kg DW and, in WT tomato fruits, was 147.79 mg/kg DW, which was very close. In our research, rutin was the most abundant flavonoid in both WT tomato fruits and NOR-like1 tomato fruits; however, the accumulation patterns in the two varieties were different.

Naringenin chalcone and rutin were detected in large quantities in different varieties of tomatoes [40,41]. Similarly, in our study, we found that the content of naringenin chalcone and rutin in WT tomato fruits was different from that in NOR-like1 tomato fruits but were both very high. The content of naringenin chalcone in NOR-like1 tomato fruit was highest during the BR3 stage (29.6 mg/kg DW); the content of naringenin chalcone in WT-BR3 tomato fruits was 28.46 mg/kg DW. When in the BR9 growth stage, the content of naringenin chalcone in NOR-like1 tomato fruits was 21.6 mg/kg DW, which was much higher than that in WT tomato fruit (10.67 mg/kg DW). The content of naringenin chalcone decreased from the BR3 stage to the BR9 stage. In our study, six types of chalcones showed significant changes, including isoliquirigenin, philorizin, trilobatin, philoretin, naringenin chalcone, and sieboldin. Among them, naringenin chalcone showed the most significant changes and had the highest content. Iijima et al. (2008) demonstrated that naringenin chalcone was the major chalcone in tomato fruit, revealing the highest accumulation levels at the breaker stage, which then decreased during ripening [42]. Similarly, Raffo et al. (2002) also mentioned that the content of naringenin gradually decreased with maturity [43]. Naringenin chalcone and rutin are considered to be the most abundant substances based on a description by Muir et al. (2001) [44].

The content of nicotiflorin in WT tomato fruits was highest during the GR stage (102.77 mg/kg DW), and the total content of nicotiflorin in NOR-like1-GR tomato fruits was 68.37 mg/kg DW. When in the BR3 growth stage, the content of nicotiflorin in NOR-like1 tomato fruit was 46.47 mg/kg DW, and the content of nicotiflorin in WT-BR3 tomato fruit was 65.96 mg/kg DW. When in the BR9 growth stage, the content of nicotiflorin NOR-like1 tomato fruits was 60.35 mg/kg DW; meanwhile, the content of nicotiflorin WT tomato fruits decreased to 53.63 mg/kg DW, lower than the NOR-like1 tomato fruits. Nicotiflorin was also extensively detected in tomatoes in a study by Le Gall et al. (2003) [45].

The content of eriodictyol increased during the GR stage to the BR3 stage, with 4.89 mg/kg DW in WT-BR3 and 7.18 mg/kg DW in NOR-like1-BR3, decreased slightly to 4.66 mg/kg DW in WT-BR9, but increased to 11.84 mg/kg DW in NOR-like1-BR9. For tomatoes in the same growth period, the content of eriodictol in NOR-like1 tomato fruits was significantly higher than that in the WT. Mintz Oron et al. (2008) found that tomatoes contain a large amount of chalconingenin, naringenin, eriodictyol, and nicotiflorin [46].

From the above data comparison, the accumulation pattern of flavonoid metabolites in NOR-like1 tomato fruits differed from that in WT tomato fruits, especially in the later ripening process BR9. The contents of the four dominant flavonoids (rutin, nicotiflorin, eriodictyol, and naringenin-7-glucoside) increased, and there were eight upregulated flavonoids in NOR-like1-BR9 than that in NOR-like1-BR3. The differential flavonoid metabolites in WT-BR9 tomato fruits were mostly downregulated compared with those in WT-BR3. As the three predominant components in WT-BR3, the contents of rutin, nicotiflorin, and naringenin chalcone decreased from BR3 to BR9. The transcriptomics results in our previous studies [28] showed that the expression levels of key genes involved in the biosynthesis of flavonoid metabolites were upregulated significantly during BR9 in NOR-like1 tomatoes compared to WT tomatoes, which may provide some support to show that NOR-like1 influences the metabolomics of flavonoid metabolites in tomato fruits at the level of gene expression.

### 3.4. Enrichment Analysis of Differential Flavonoid Metabolites

**Figure 9:**
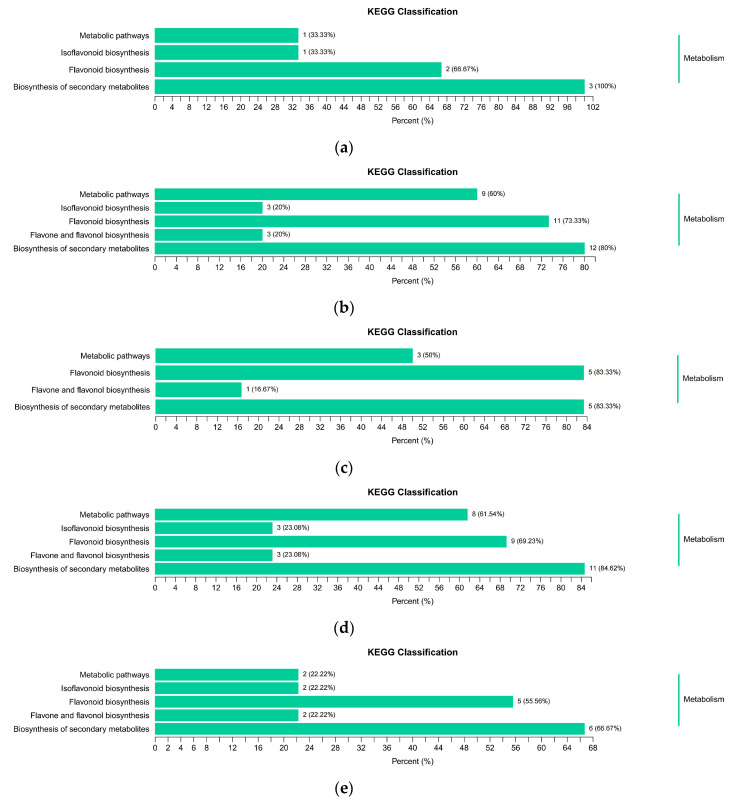

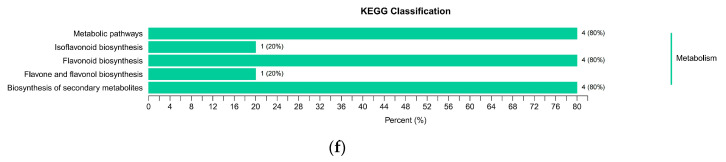
KEGG enrichment analysis of the differential metabolites in the comparisons of (**a**) NOR-like1-BR3 vs. NOR-like1-BR9, (**b**) NOR-like1-GR vs. NOR-like1-BR3, (**c**) WT-BR3 vs. WT-BR9, (**d**) WT-GR vs. WT-BR3, (**e**) WT-GR vs. NOR-like1-GR, and (**f**) WT-BR9 vs. NOR-like1-BR9.

The results of further KEGG enrichment analysis are shown in Figure 9a–f. It is revealed that the significant enrichment of the differential flavonoid metabolites was distributed in the following pathways: flavonoid biosynthesis, flavone and flavonol biosynthesis, the biosynthesis of secondary metabolites, metabolic pathways, and isoflavonoid biosynthesis. The most enriched pathway was the ‘biosynthesis of secondary metabolites’. This was followed by ‘flavonoid biosynthesis’.

In NOR-like1-BR3_vs_NOR-like1-BR9 (Figure 9a), phlorizin and phloretin were enriched in the flavonoid biosynthesis pathway; genistein was enriched in the isoflavonoid biosynthesis. In NOR-like1-GR_vs_NOR-like1-BR3 (Figure 9b), quercitrin, quercetin, and luteolin were enriched in flavone and flavonol biosynthesis; isoliquiritigenin, phlorizin, phloretin, naringenin chalcone, (-)-epicatechin, (-)-epigallocatechin, eriodictyol, dihydrokaempferol, taxifolin, luteolin, and quercetin were related to ‘flavonoid biosynthesis’; genistein, glycitin, and 2′-hydroxygenistein were enriched in isoflavonoid biosynthesis. In WT-BR3_vs_WT-BR9 (Figure 9c), baimaside was enriched in flavone and flavonol biosynthesis. Isoliquiritigenin, phlorizin, phloretin, naringenin chalcone, and dihydrokaempferol were enriched in flavonoid biosynthesis. In WT-GR_vs_WT-BR3 (Figure 9d), luteolin, quercetin, and astragalin were enriched in flavone and flavonol biosynthesis. Isoliquiritigenin, phlorizin, phloretin, naringenin chalcone, eriodictyol, dihydrokaempferol, taxifolin, luteolin, and quercetin were enriched in flavonoid biosynthesis. Genistein, glycitin, and 2′-hydroxygenistein were related to ‘isoflavonoid biosynthesis’. In WT-GR_vs_NOR-like1-GR (Figure 9e), astragalin and quercitrin were enriched in flavone and flavonol biosynthesis. Phlorizin, phloretin, naringenin chalcone, (-)-epicatechin, and (-)-epigallocatechin were enriched in flavonoid biosynthesis. The regulation of ononin and glycitin was related to isoflavonoid biosynthesis. In WT-BR9_vs_NOR-like1-BR9 (Figure 9f), luteolin was enriched in flavone and flavonol biosynthesis. Isoliquiritigenin, naringenin chalcone, eriodictyol, and luteolin were enriched in flavonoid biosynthesis. Glycitin was related to ‘isoflavonoid biosynthesis’. In all the above comparison groups, flavonoid biosynthesis was significantly enriched, which may indicate that the differential flavonoids in these enrichment pathways may lead to changes in flavonoid accumulation between the two types of tomato fruits during the whole ripening process.

## 4. Conclusions

Flavonoid quantitative metabolomics was used to accurately quantify and analyze the changes in the flavonoid metabolites of wild-type tomato fruits (WT) and gene-edited tomato fruits (NOR-like1) during the tomato ripening process. A total of 50 flavonoid metabolites were identified from the WT and NOR-like1 tomato fruits using UPLC-MS/MS. Rutin, nicotiflorin, naringenin chalcone, eriodictyol, and naringenin-7-glucoside were the five flavonoids with the highest content in the ripening stages (BR3 and BR9) in both WT and NOR-like1 tomato fruits. The overall flavonoid contents in WT tomato fruits changed little from GR to BR3 and decreased from BR3 to BR9; meanwhile, in NOR-like1 tomato fruits, the total amounts of the flavonoids exhibited an increasing trend during all three ripening stages. The accumulation pattern of flavonoid metabolites in NOR-like1 tomato fruits differed from that in WT tomato fruits, especially in the later ripening process BR9. These findings highlight significant differences in the profile of flavonoid metabolites between WT and NOR-like1 tomato samples and showed that NOR-like1 regulated the biosynthesis of flavonoids and changed the accumulation pattern of flavonoids. Overall, our research provides new insights into the dynamic changes in flavonoid profiles caused by transcription factor NOR-like1 during ripening. Our future studies may focus on the flavonoids most significantly affected, extensively investigate the interaction mechanism between transcription factor NOR-like1 and the related genes, and elucidate the regulatory mechanism of NOR-like1 on flavonoids.

## Figures and Tables

**Table 1:** Results of pairwise comparison of fifty flavonoid metabolites identified in the six tomato sample groups.

Index	Compounds	WT-GR vs. NOR-like1-GR		WT-BR3 vs. NOR-like1-BR3		WT-BR9 vs. NOR-like1-BR9		NOR-like1-GR vs. NOR-like1-BR3		NOR-like1-BR3 vs. NOR-like1-BR9		WT-GR vs. WT-BR3		WT-BR3 vs. WT-BR9	
		VIP	Log_2_FC	VIP	Log_2_FC	VIP	Log_2_FC	VIP	Log_2_FC	VIP	Log_2_FC	VIP	Log_2_FC	VIP	Log_2_FC
1	Astilbin	-	-	-	-	-	-	-	-	-	-	-	-	-	-
2	Miquelianin	-	-	-	-	-	-	-	-	-	-	-	-	-	-
9	Genistein	-	-	-	-	-	-	1.48	inf	1.41	1.38	1.38	inf	-	-
106	Typhaneoside	-	-	1.38	−1.27	-	-	-	-	-	-	-	-	-	-
11	Hydroxysafflor yellow A	-	-	-	-	1.24	1.11	1.19	1.35	-	-	-	-	-	-
115	Quercitrin	1.08	2.77	-	-	-	-	1.24	−4.87	-	-	-	-	-	-
117	Engeletin	-	-	1.35	−1.18	-	-	-	-	-	-	1.39	inf	-	-
118	Narcissin	-	-	-	-	-	-	-	-	-	-	-	-	1.04	−1.71
119	Astragalin	1.25	−1.22	-	-	-	-	-	-	-	-	1.19	−1.19	-	-
124	Silychristin	-	-	-	-	-	-	-	-	-	-	-	-	-	-
126	Eriodictyol	-	-	-	-	1.37	1.34	1.35	6.46	-	-	1.38	8.24	-	-
13	Isoorientin	-	-	-	-	1.32	2.84	-	-	1.83	inf	-	-	-	-
135	Theaflavin 3,3′-digallate	-	-	-	-	-	-	-	-	-	-	-	-	-	-
136	6,2′-Dihydroxyflavone	-	-	-	-	1.22	1.05	1.05	inf	-	-	1.39	inf	1.27	−1.38
138	Baimaside	-	-	-	-	-	-	-	-	-	-	-	-	1.15	−6.44
139	Dihydrokaempferol	-	-	-	-	-	-	1.47	6.66	-	-	1.38	7.25	1.57	−1.78
145	Procyanidin B2	1.1	4.5	-	-	-	-	1.02	−3.51	-	-	-	-	-	-
147	(-)-Epicatechin	1.29	3.11	-	-	-	-	1.32	−3.74	-	-	-	-	-	-
150	(-)-Catechin gallate	1.35	6.76	-	-	-	-	1.2	−6.36	-	-	-	-	-	-
152	Ononin	1.13	−inf	-	-	-	-	-	-	-	-	-	-	-	-
153	Glycitin	1.63	inf	1.77	1.94	1.72	1.78	1.12	1.62	-	-	1.39	inf	-	-
157	Isomangiferin	-	-	1.63	1.49	-	-	-	-	-	-	-	-	-	-
160	Apigenin 7-glucoside	-	-	-	-	-	-	-	-	1.83	inf	-	-	1.2	inf
169	Quercetin 3-O-(6″-galloyl)-β-D-galactopyranoside	-	-	-	-	-	-	-	-	-	-	-	-	-	-
176	Naringenin−7-glucoside	-	-	-	-	-	-	1.39	4.9	1.46	1.39	1.32	5.07	-	-
177	Phloretin	1.08	inf	-	-	-	-	1.34	7.64	1.07	−1.11	1.39	inf	1.48	−2.26
183	2′-Hydroxygenistein	-	-	-	-	-	-	1.05	inf	-	-	1.38	inf	-	-
184	Afzelechin	-	-	-	-	-	-	-	-	-	-	-	-	-	-
191	Naringenin chalcone	1.15	4.01	-	-	1.31	1.02	1.38	7.85	-	-	1.38	11.8	1.41	−1.42
195	(-)-Gallocatechin	1.15	2.18	-	-	-	-	1.13	−2.11	-	-	-	-	-	-
197	Isorhamnetin 3-O-glucoside	-	-	-	-	-	-	-	-	-	-	-	-	-	-
201	Trilobatin	-	-	-	-	-	-	1.48	inf	1.26	1.4	1.39	inf	1.67	1.68
202	Sieboldin	-	-	1.36	1.04	-	-	-	-	1.82	3.19	-	-	1.49	4.91
23	Quercetin	-	-	-	-	-	-	1.05	inf	-	-	1.39	inf	-	-
25	Apigenin-7-glucuronide	1.15	inf	1.98	inf	1.24	inf	-	-	1.27	−1.34	-	-	-	-
28	Scutellarin	-	-	-	-	-	-	-	-	-	-	-	-	-	-
30	Isoliquiritigenin	-	-	-	-	1.65	2.2	1.05	inf	-	-	1.38	inf	1.61	_2.77
42	Nicotiflorin	-	-	-	-	-	-	-	-	-	-	-	-	-	-
48	Narirutin	-	-	1.89	−5.14	1.27	−1.89	-	-	1.72	4.39	1.39	17.52	-	-
50	Luteolin	-	-	-	-	1.02	1.36	1.05	inf	-	-	1.39	inf	-	-
55	(-)-Catechin	-	-	1.81	1.43	1.57	1.33	-	-	-	-	-	-	-	-
56	Taxifolin	-	-	-	-	-	-	1.48	inf	-	-	1.38	inf	-	-
57	Rutin	-	-	-	-	-	-	-	-	-	-	-	-	-	-
58	Isorhamnetin	-	-	-	−	-	-	-	-	-	-	-	-	-	-
65	Phlorizin	1.35	−1.60	-	-	-	-	1.35	3.18	1.61	1.85	1.28	2.05	1.6	1.05
69	Liquiritin	-	-	-	-	-	-	1.36	1.37	-	-	1.21	1.5	-	-
80	(-)-Gallocatechin gallate	1.4	4.27	-	-	-	-	1.13	−3.33	-	-	-	-	-	-
84	(-)-Epigallocatechin	1.42	3.19	-	-	-	-	1.29	−3.36	-	-	-	-	-	-
90	Cynaroside	1.16	1.73	1.38	inf	-	-	-	-	1.27	−inf	-	-	-	-
96	Calycosin-7-O-β-D-glucoside	1.64	inf	-	-	1.74	inf	-	-	-	-	-	-	1.23	−inf

* Log_2_ FC ≥ 1 or ≤−1 and VIP ≥ 1 were considered to denote significant difference. “Inf” means infinity; “-“ in the table indicates insignificant difference.

**Table 2:** The individual contents (mg/kg DW) of 50 flavonoids at different stages in the fruit of WT and NOR-like1 tomatoes.

Index	Compounds	WT-(mg/kg)	WT-BR3 (mg/kg)	WT-BR9 (mg/kg)	NOR-like1-GR (mg/kg)	NOR-like1-BR3 (mg/kg)	NOR-like1-BR9 (mg/kg)
1	Astilbin	0.021 ± 0.01	0.018 ± 0	0.013 ± 0	0.03 ± 0.01	0.024 ± 0	0.022 ± 0
2	Miquelianin	0.011 ± 0	0.018 ± 0	0.021 ± 0.01	0.013 ± 0	0.014 ± 0	0.06 ± 0.09
9	Genistein	-	0.013 ± 0	0.018 ± 0	-	0.011 ± 0.01	0.03 ± 0.01
106	Typhaneoside	0.012 ± 0.01	0.09 ± 0.04	0.03 ± 0.03	0.018 ± 0.03	0.04 ± 0.07	0.04 ± 0.04
11	Hydroxysafflor yellow A	0.08 ± 0.06	0.17 ± 0.09	0.10 ± 0.03	0.08 ± 0.04	0.21 ± 0.02	0.22 ± 0.11
115	Quercitrin	0.015 ± 0.02	0.004 ± 0	0.002 ± 0	0.1 ± 0.09	0.004 ± 0.01	0.006 ± 0.01
117	Engeletin	-	0.016 ± 0.01	0.008 ± 0.01	-	0.007 ± 0.01	0.008 ± 0.01
118	Narcissin	1.36 ± 0.31	1.76 ± 1.87	0.54 ± 0.12	0.8 ± 0.16	1.14 ± 0.28	0.77 ± 0.08
119	Astragalin	0.27 ± 0.06	0.12 ± 0.04	0.09 ± 0.07	0.12 ± 0.06	0.1 ± 0.02	0.23 ± 0.24
124	Silychristin	-	0.013 ± 0.01	0.03 ± 0.01	0.003 ± 0.01	0.003 ± 0.01	0.017 ± 0.02
126	Eriodictyol	0.016 ± 0	4.89 ± 2.31	4.67 ± 2.54	0.08 ± 0.06	7.19 ± 5.16	11.84 ± 3.07
13	Isoorientin	-	-	0.005 ± 0.01	0.008 ± 0.01	-	0.04 ± 0.02
135	Theaflavin 3,3′-digallate	-	0.05 ± 0.09	-	-	-	-
136	6,2′-Dihydroxyflavone	-	0.019 ± 0	0.007 ± 0	-	0.014 ± 0.01	0.015 ± 0
138	Baimaside	0.59 ± 1	0.5 ± 0.53	0.006 ± 0	0.12 ± 0.08	0.07 ± 0.1	0.023 ± 0.03
139	Dihydrokaempferol	0.004 ± 0	0.63 ± 0.1	0.19 ± 0.06	0.005 ± 0	0.51 ± 0.26	0.32 ± 0.15
145	Procyanidin B2	0.016 ± 0.02	0.023 ± 0.02	0.04 ± 0.03	0.37 ± 0.17	0.03 ± 0.03	0.028 ± 0.05
147	(-)-Epicatechin	0.05 ± 0.03	0.04 ± 0.02	0.05 ± 0.02	0.45 ± 0.44	0.03 ± 0.01	0.04 ± 0.03
150	(-)-Catechin gallate	0.015 ± 0.01	0.01 ± 0.01	0.021 ± 0.03	1.57 ± 2.43	0.019 ± 0.02	0.017 ± 0.01
152	Ononin	0.04 ± 0.05	-	-	-	-	-
153	Glycitin	-	0.05 ± 0.01	0.04 ± 0	0.06 ± 0.05	0.19 ± 0.1	0.13 ± 0.03
157	Isomangiferin	0.005 ± 0	0.002 ± 0	0.003 ± 0	0.005 ± 0	0.005 ± 0	0.006 ± 0
160	Apigenin 7-glucoside	-	-	0.011 ± 0.01	-	-	0.013 ± 0.01
169	Quercetin 3-O-(6″-galloyl)-β-D-galactopyranoside	0.003 ± 0	-	-	-	-	-
176	Naringenin-7-glucoside	0.08 ± 0.08	2.58 ± 0.75	4.05 ± 1.64	0.05 ± 0.03	1.42 ± 0.91	3.72 ± 0.31
177	Phloretin	-	1.80 ± 0.44	0.37 ± 0.17	0.005 ± 0.01	1.07 ± 0.68	0.50 ± 0.27
183	2′-Hydroxygenistein	-	0.006 ± 0	0.003 ± 0	-	0.005 ± 0	0.005 ± 0
184	Afzelechin	0.04 ± 0.04	0.013 ± 0.02	0.019 ± 0.02	0.02 ± 0.04	-	0.009 ± 0.02
191	Naringenin chalcone	0.008 ± 0	28.46 ± 5.57	10.67 ± 4.94	0.13 ± 0.15	29.60 ± 22.19	21.62 ± 5.88
195	(-)-Gallocatechin	0.04 ± 0.04	0.05 ± 0.01	0.08 ± 0.03	0.20 ± 0.19	0.05 ± 0.01	0.04 ± 0.01
197	Isorhamnetin 3-O-glucoside	0.004 ± 0	0.004 ± 0.01	0.002 ± 0	0.002 ± 0	0.001 ± 0	-
201	Trilobatin	-	0.008 ± 0	0.03 ± 0	-	0.007 ± 0	0.018 ± 0.01
202	Sieboldin	0.008 ± 0.01	0.007 ± 0.01	0.22 ± 0.05	0.017 ± 0	0.015 ± 0.01	0.14 ± 0.04
23	Quercetin	-	0.13 ± 0.04	0.09 ± 0.05	-	0.09 ± 0.08	0.09 ± 0.03
25	Apigenin-7-glucuronide	-	-	-	0.002 ± 0	0.007 ± 0	0.003 ± 0
28	Scutellarin	-	-	-	0.019 ± 0.03	-	0.005 ± 0.01
30	Isoliquiritigenin	-	0.005 ± 0	0.001 ± 0	-	0.006 ± 0.01	0.003 ± 0
42	Nicotiflorin	102.77 ± 14.56	65.96 ± 25.76	53.63 ± 13.28	68.37 ± 45.17	46.47 ± 13.75	60.35 ± 23.33
48	Narirutin	-	0.04 ± 0.01	0.09 ± 0.05	-	0.001 ± 0	0.025 ± 0.02
50	Luteolin	-	0.009 ± 0	0.013 ± 0.01	-	0.015 ± 0.01	0.03 ± 0.01
55	(-)-Catechin	0.07 ± 0.03	0.06 ± 0.01	0.05 ± 0	0.34 ± 0.43	0.16 ± 0.06	0.12 ± 0.04
56	Taxifolin	-	0.04 ± 0.01	0.06 ± 0.02	-	0.05 ± 0.04	0.09 ± 0.01
57	Rutin	175.26 ± 44.54	168.46 ± 14.85	147.79 ± 18.69	101.18 ± 47.22	125.43 ± 29.2	145.01 ± 15.91
58	Isorhamnetin	-	0.004 ± 0.01	-	-	-	-
65	Phlorizin	0.012 ± 0.01	0.05 ± 0.01	0.11 ± 0.01	0.004 ± 0	0.04 ± 0.02	0.13 ± 0.02
69	Liquiritin	0.004 ± 0	0.011 ± 0	0.013 ± 0.01	0.004 ± 0	0.01 ± 0	0.013 ± 0
80	(-)-Gallocatechin gallate	0.09 ± 0.07	0.06 ± 0.04	0.11 ± 0.12	1.66 ± 1.57	0.17 ± 0.19	0.06 ± 0.02
84	(-)-Epigallocatechin	0.09 ± 0.06	0.06 ± 0.03	0.10 ± 0.11	0.85 ± 0.52	0.08 ± 0.06	0.04 ± 0.02
90	Cynaroside	0.05 ± 0.09	-	-	0.17 ± 0.03	0.06 ± 0.05	-
96	Calycosin-7-O-β-D-glucoside	-	0.009 ± 0.01	-	0.025 ± 0.01	0.025 ± 0.02	0.03 ± 0
total amount		281.02 ± 28.02	276.25 ± 22.95	223.37 ± 33.78	176.87 ± 66.48	214.39 ± 39.51	245.93 ± 35.24

* The result is indicated as the mean ± SD (n = 3); “-” in the table indicates not detected.

## Data Availability

The data supporting the results of this study are included in the present article.

## Supplementary Materials

The following supporting information can be downloaded at: https://www.mdpi.com/article/10.3390/foods12244445/s1, Table S1. Detailed information on the fifty flavonoid metabolites identified in the tomato fruit samples. Supplementary File S1. MS/MS spectra of the fifty flavonoids identified in the tomato samples. Supplementary File S2. UHPLC-MS/MS parameters of the fifty flavonoids. Supplementary File S3. Calibration curves and quantitative details of the fifty flavonoids.

## References

[1] Gao J., Song Y., Mao J. (2007). The determination of flavonoids in some fruit and vegetables. China Food Addit..

[2] Treutter D. (2006). Significance of flavonoids in plant resistance: A review. Environ. Chem. Lett..

[3] Bui T.T., Nguyen T.H. (2017). Natural product for the treatment of Alzheimer’s disease. J. Basic Clin. Physiol. Pharmacol..

[4] Tan T., Li Y., Tang B., Chen Y., Chen X., Xie Q., Hu Z., Chen G. (2022). Knockout of SlALKBH2 weakens the DNA damage repair ability of tomato. Plant Sci..

[5] Tao X., Wu Q., Huang S., Zhu B., Chen F., Liu B., Cai L., Mao L., Luo Z., Li L. (2022). Exogenous abscisic acid regulates primary metabolism in postharvest cherry tomato fruit during ripening. Sci. Hortic..

[6] Monribot-Villanueva J.L., Altúzar-Molina A., Aluja M., Zamora-Briseño J.A., Elizalde-Contreras J.M., Bautista-Valle M.V., Arellano de Los Santos J., Sánchez-Martínez D.E., Rivera-Reséndiz F.J., Vázquez-Rosas-Landa M. (2022). Integrating proteomics and metabolomics approaches to elucidate the ripening process in white Psidium guajava. Food Chem..

[7] Yonekura-Sakakibara K., Higashi Y., Nakabayashi R. (2019). The Origin and Evolution of Plant Flavonoid Metabolism. Front. Plant Sci..

[8] Xiao J., Muzashvili T.S., Georgiev M.I. (2014). Advances in the biotechnological glycosylation of valuable flavonoids. Biotechnol. Adv..

[9] Liu Z.-Q. (2022). What about the progress in the synthesis of flavonoid from 2020?. Eur. J. Med. Chem..

[10] Zhang X., Qi X., Chen Y., Yao H., Zheng Y. (2020). Research Advances of Flavonoids in Strawberry. North. Hortic..

[11] Tejada S., Pinya S., Martorell M., Capó X., Tur J.A., Pons A., Sureda A. (2018). Potential Anti-inflammatory Effects of Hesperidin from the Genus Citrus. Curr. Med. Chem..

[12] Shen N., Wang T., Gan Q., Liu S., Wang L., Jin B. (2022). Plant flavonoids: Classification, distribution, biosynthesis, and antioxidant activity. Food Chem..

[13] Staszowska-Karkut M., Materska M. (2020). Phenolic Composition, Mineral Content, and Beneficial Bioactivities of Leaf Extracts from Black Currant (*Ribes nigrum* L.), Raspberry (*Rubus idaeus*), and Aronia (*Aronia melanocarpa*). Nutrients.

[14] Tohge T., de Souza L.P., Fernie A.R. (2017). Current understanding of the pathways of flavonoid biosynthesis in model and crop plants. J. Exp. Bot..

[15] Song X., Wei J., Di S., Pang Y. (2019). Recent Advances in the Regulation Mechanism of Transcription Factors and Metabolic Engineering of Anthocyanins. Chin. Bull. Bot..

[16] Huang Y., Cui L., Chen W., Liu Z., Yuan W., Zhu F., Jiao Z., Zhang Z., Deng X., Wang L. (2022). Comprehensive analysis of NAC transcription factors and their expressions during taproot coloration in radish (*Raphanus sativus* L.). Sci. Hortic..

[17] Sun Q., Jiang S., Zhang T., Xu H., Fang H., Zhang J., Su M., Wang Y., Zhang Z., Wang N. (2019). Apple NAC transcription factor MdNAC52 regulates biosynthesis of anthocyanin and proanthocyanidin through MdMYB9 and MdMYB11. Plant Sci..

[18] Duan A.-Q., Yang X.-L., Feng K., Liu J.-X., Xu Z.-S., Xiong A.-S. (2020). Genome-wide analysis of NAC transcription factors and their response to abiotic stress in celery (*Apium graveolens* L.). Comput. Biol. Chem..

[19] Hu H., Dai M., Yao J., Xiao B., Li X., Zhang Q., Xiong L. (2006). Overexpressing a NAM, ATAF, and CUC (NAC) transcription factor enhances drought resistance and salt tolerance in rice. Proc. Natl. Acad. Sci. USA.

[20] Jeong J.S., Kim Y.S., Baek K.H., Jung H., Ha S.-H., Do Choi Y., Kim M., Reuzeau C., Kim J.-K. (2010). Root-Specific Expression of OsNAC10 Improves Drought Tolerance and Grain Yield in Rice under Field Drought Conditions. Plant Physiol..

[21] Guo F., Liu S., Zhang C., Dong T., Meng X., Zhu M. (2022). Genome-wide systematic survey and analysis of NAC transcription factor family and their response to abiotic stress in sweetpotato. Sci. Hortic..

[22] Li M., Chen R., Jiang Q., Sun X., Zhang H., Hu Z. (2021). GmNAC06, a NAC domain transcription factor enhances salt stress tolerance in soybean. Plant Mol. Biol..

[23] Ma J., Wang L.-Y., Dai J.-X., Wang Y., Lin D. (2021). The NAC-type transcription factor CaNAC46 regulates the salt and drought tolerance of transgenic Arabidopsis thaliana. BMC Plant Biol..

[24] Wang L., Hu Z., Zhu M., Zhu Z., Hu J., Qanmber G., Chen G. (2017). The abiotic stress-responsive NAC transcription factor SlNAC11 is involved in drought and salt response in tomato (*Solanum lycopersicum* L.). Plant Cell Tissue Organ Cult. (PCTOC).

[25] Zhu M., Chen G., Zhang J., Zhang Y., Xie Q., Zhao Z., Pan Y., Hu Z. (2014). The abiotic stress-responsive NAC-type transcription factor SlNAC4 regulates salt and drought tolerance and stress-related genes in tomato (*Solanum lycopersicum*). Plant Cell Rep..

[26] Gao Y., Wei W., Zhao X., Tan X., Fan Z., Zhang Y., Jing Y., Meng L., Zhu B., Zhu H. (2018). A NAC transcription factor, NOR-like1, is a new positive regulator of tomato fruit ripening. Hortic. Res..

[27] Gao Y., Wei W., Fan Z., Zhao X., Zhang Y., Jing Y., Zhu B., Zhu H., Shan W., Chen J. (2020). Re-evaluation of the nor mutation and the role of the NAC-NOR transcription factor in tomato fruit ripening. J. Exp. Bot..

[28] Yang X., Zhao X., Fu D., Zhao Y. (2022). Integrated Analysis of Widely Targeted Metabolomics and Transcriptomics Reveals the Effects of Transcription Factor NOR-like1 on Alkaloids, Phenolic Acids, and Flavonoids in Tomato at Different Ripening Stages. Metabolites.

[29] Zhang H., Du X., Yu J., Jin H., Liu N. (2022). Comparative Metabolomics study of flavonoids in the pericarp of different coloured bitter gourds (*Momordica charantia* L.). Physiol. Mol. Biol. Plants.

[30] Wang Y., Cheng J., Jiang W., Chen S. (2022). Metabolomics study of flavonoids in Coreopsis tinctoria of different origins by UPLC-MS/MS. PeerJ.

[31] Li J., Hossain M.S., Ma H., Yang Q., Gong X., Yang P., Feng B. (2020). Comparative metabolomics reveals differences in flavonoid metabolites among different coloured buckwheat flowers. J. Food Compos. Anal..

[32] Hunt G.M., Baker E.A. (1980). Phenolic constituents of tomato fruit cuticles. Phytochemistry.

[33] Iijima Y., Nakamura Y., Ogata Y., Tanaka K., Sakurai N., Suda K., Suzuki T., Suzuki H., Okazaki K., Kitayama M. (2008). Metabolite annotations based on the integration of mass spectral information. Plant J..

[34] Krishnan M., Babu S., Thomas S.A., Surulivel J.S., Ayyanar K. (2022). Molecular docking analysis of VEGF with compounds from tomato. Bioinformation.

[35] Spencer J.P., Kuhnle G.G., Hajirezaei M., Mock H.P., Sonnewald U., Rice-Evans C. (2005). The genotypic variation of the antioxidant potential of different tomato varieties. Free Radic. Res..

[36] Slimestad R., Fossen T., Verheul M.J. (2008). The flavonoids of tomatoes. J. Agric. Food Chem..

[37] Vallverdú-Queralt A., Jáuregui O., Di Lecce G., Andrés-Lacueva C., Lamuela-Raventós R.M. (2011). Screening of the polyphenol content of tomato-based products through accurate-mass spectrometry (HPLC-ESI-QTOF). Food Chem..

[38] Le Gall G., DuPont M.S., Mellon F.A., Davis A.L., Collins G.J., Verhoeyen M.E., Colquhoun I.J. (2003). Characterization and content of flavonoid glycosides in genetically modified tomato (Lycopersicon esculentum) fruits. J. Agric. Food Chem..

[39] Larbat R., Le Bot J., Bourgaud F., Robin C., Adamowicz S. (2012). Organ-specific responses of tomato growth and phenolic metabolism to nitrate limitation. Plant Biol..

[40] Morelli C.F., Cutignano A., Speranza G., Abbamondi G.R., Rabuffetti M., Iodice C., De Prisco R., Tommonaro G. (2023). Taste Compounds and Polyphenolic Profile of Tomato Varieties Cultivated with Beneficial Microorganisms: A Chemical Investigation on Nutritional Properties and Sensory Qualities. Biomolecules.

[41] Wang H., Zhang Z., Song J., Tian M., Li R., Cui X. (2023). Phenolic compound identification in tomato fruit by UPLC-QTOF-MS. LWT.

[42] Iijima Y., Suda K., Suzuki T., Aoki K., Shibata D. (2008). Metabolite Profiling of Chalcones and Flavanones in Tomato Fruit. J. Jpn. Soc. Hortic. Sci..

[43] Raffo A., Leonardi C., Fogliano V., Ambrosino P., Salucci M., Gennaro L., Bugianesi R., Giuffrida F., Quaglia G. (2002). Nutritional value of cherry tomatoes (Lycopersicon esculentum Cv. Naomi F1) harvested at different ripening stages. J. Agric. Food Chem..

[44] Muir S.R., Collins G.J., Robinson S., Hughes S., Bovy A., Ric De Vos C.H., van Tunen A.J., Verhoeyen M.E. (2001). Overexpression of petunia chalcone isomerase in tomato results in fruit containing increased levels of flavonols. Nat. Biotechnol..

[45] Le Gall G., Colquhoun I.J., Davis A.L., Collins G.J., Verhoeyen M.E. (2003). Metabolite profiling of tomato (*Lycopersicon esculentum*) using 1H NMR spectroscopy as a tool to detect potential unintended effects following a genetic modification. J. Agric. Food Chem..

[46] Mintz-Oron S., Mandel T., Rogachev I., Feldberg L., Lotan O., Yativ M., Wang Z., Jetter R., Venger I., Adato A. (2008). Gene expression and metabolism in tomato fruit surface tissues. Plant Physiol..

