# Supplementary material for: Metabolomics Study of the Effect of Transcription Factor NOR-like1 on Flavonoids in Tomato at Different Stages of Maturity Using UPLC-MS/MS
Source: Foods. 2023 Dec 11;12(24):4445. doi: 10.3390/foods12244445 (PMC10742431; doi:10.3390/foods12244445)

Compound name: Astilbin (449.1 / 285.0)

Sample Name	Sample Type	Area (cps)	Is Area (cps)	RT (min)	S/N	Target Conc	Calculated Conc.()
STD_0.5nM	Standard	1.04e3	N/A	2.90	9.1	0.5000	3.075874e-1
STD_1nM	Standard	3.81e3	N/A	2.88	14.2	1.0000	1.014366e0
STD_5nM	Standard	2.66e4	N/A	2.88	37.4	5.0000	6.816256e0
STD_10nM	Standard	3.89e4	N/A	2.89	39.6	10.0000	9.940285e0
STD_20nM	Standard	8.07e4	N/A	2.88	38.1	20.0000	2.060403e1
STD_50nM	Standard	1.98e5	N/A	2.89	62.0	50.0000	5.048030e1
STD_100nM	Standard	3.82e5	N/A	2.89	48.4	100.0000	9.733717e1
STD_200nM	Standard	N/A	N/A	N/A	N/A	200.0000	N/A
STD_500nM	Standard	N/A	N/A	N/A	N/A	500.0000	N/A
STD_1000nM	Standard	N/A	N/A	N/A	N/A	1000.0000	N/A
STD_2000nM	Standard	N/A	N/A	N/A	N/A	2000.0000	N/A
V1.0_MW_RQC1_20211018	Quality Control	N/A	N/A	N/A	N/A	0.0000	N/A
Blank	Unknown	N/A	N/A	N/A	N/A	N/A	N/A
V1.0_MWMS_20211021_1	Unknown	6.27e5	N/A	2.89	50.4	N/A	1.596504e2
MWXS212101D3_R1	Quality Control	6.81e5	N/A	2.89	50.6	0.0000	1.733673e2
MWXS212101D3_R2	Quality Control	6.33e5	N/A	2.89	48.2	0.0000	1.612760e2
MWXS212101D3_R3	Quality Control	6.47e5	N/A	2.89	48.9	0.0000	1.648422e2
A21233250b_b	Unknown	5.19e3	N/A	2.94	15.4	N/A	1.364767e0
A21233251b_b	Unknown	5.54e3	N/A	2.93	16.2	N/A	1.455099e0
A21233252b_b	Unknown	1.03e4	N/A	2.94	20.1	N/A	2.653501e0
A21233253b_b	Unknown	6.07e3	N/A	2.93	13.0	N/A	1.588932e0
A21233254b_b	Unknown	5.03e3	N/A	2.93	9.3	N/A	1.324983e0
A21233255b_b	Unknown	7.10e3	N/A	2.93	15.3	N/A	1.850203e0
A21233256b_b	Unknown	3.23e3	N/A	2.92	5.9	N/A	8.651257e-1
A21233257b_b	Unknown	5.67e3	N/A	2.94	7.6	N/A	1.486291e0
A21233258b_b	Unknown	4.39e3	N/A	2.93	5.5	N/A	1.159875e0
A21233259b_b	Unknown	7.52e3	N/A	2.93	17.7	N/A	1.957969e0
A21233260b_b	Unknown	1.19e4	N/A	2.93	20.3	N/A	3.074219e0
A21233261b_b	Unknown	1.46e4	N/A	2.93	16.5	N/A	3.753957e0
A21233262b_b	Unknown	9.53e3	N/A	2.94	18.2	N/A	2.471183e0
A21233263b_b	Unknown	7.27e3	N/A	2.93	15.2	N/A	1.893518e0
A21233264b_b	Unknown	7.38e3	N/A	2.93	10.3	N/A	1.923572e0
A21233265b_b	Unknown	5.36e3	N/A	2.93	6.7	N/A	1.408908e0
A21233266b_b	Unknown	8.54e3	N/A	2.93	12.8	N/A	2.216755e0
A21233267b_b	Unknown	8.26e3	N/A	2.94	12.5	N/A	2.145825e0

Compound name: Astilbin
Regression Equation: y = 3926.68587 x + -169.27244 (r = 0.99760) (weighting: 1 / x)

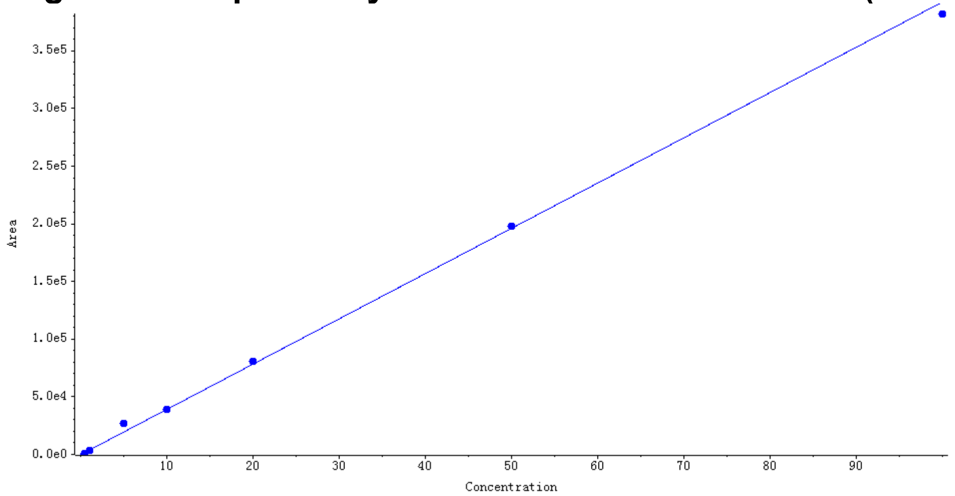

Peak Review

V1.0_MWMS_20211021_1
Astilbin AREA:6.27e5 S/N:50.4

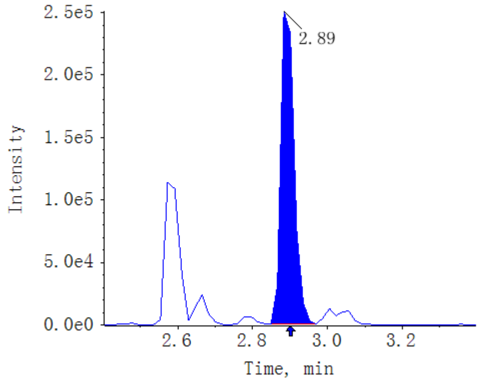

A21233250b_b
Astilbin AREA:5.19e3 S/N:15.4

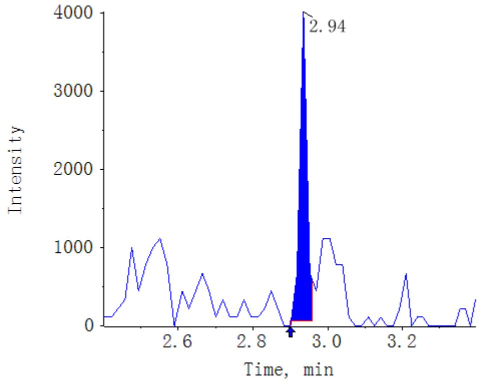

A21233251b_b
Astilbin AREA:5.54e3 S/N:16.2

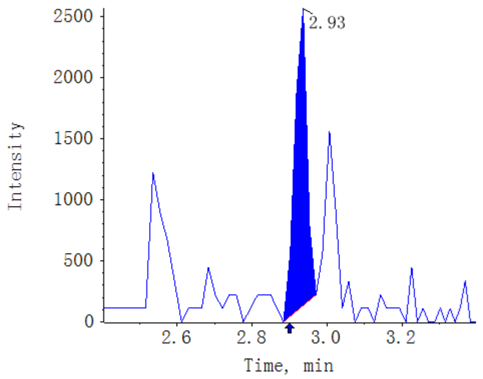

A21233252b_b
Astilbin AREA:1.03e4 S/N:20.1

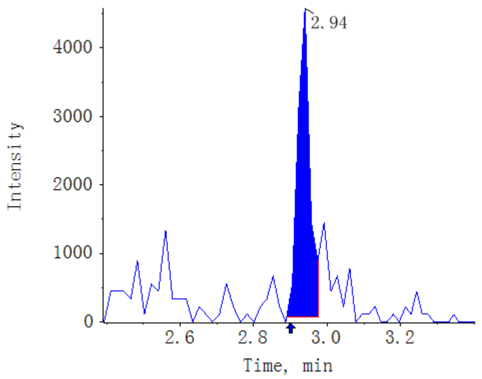

A21233253b_b
Astilbin AREA:6.07e3 S/N:13.0

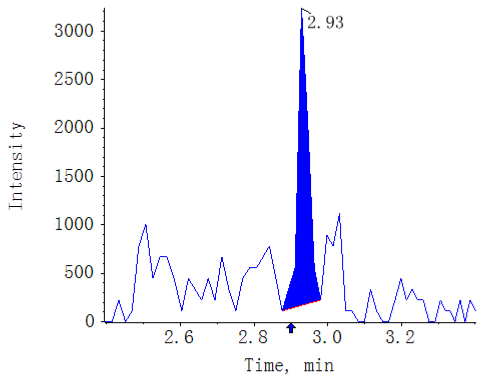

A21233254b_b
Astilbin AREA:5.03e3 S/N:9.3

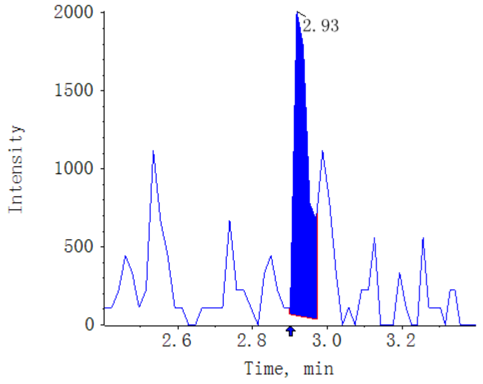

A21233255b_b
Astilbin AREA:7.10e3 S/N:15.3

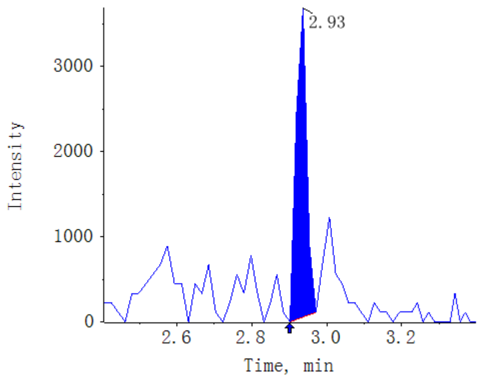

A21233256b_b
Astilbin AREA:3.23e3 S/N:5.9

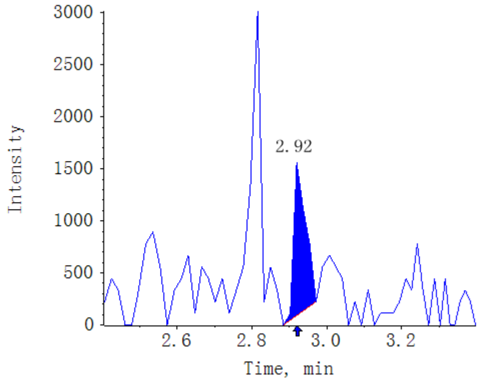

A21233257b_b
Astilbin AREA:5.67e3 S/N:7.6

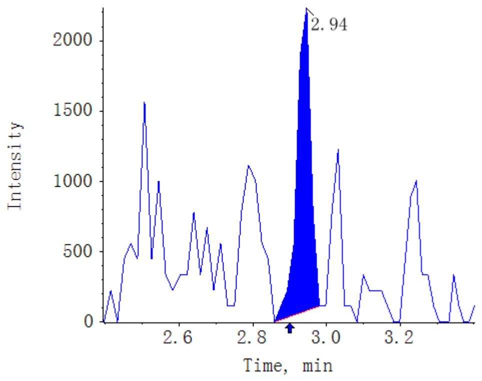

A21233258b_b
Astilbin AREA:4.39e3 S/N:5.5

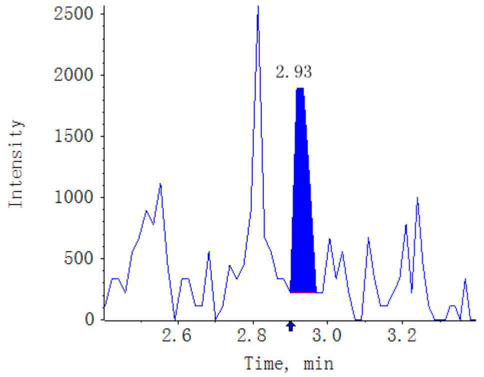

A21233259b_b
Astilbin AREA:7.52e3 S/N:17.7

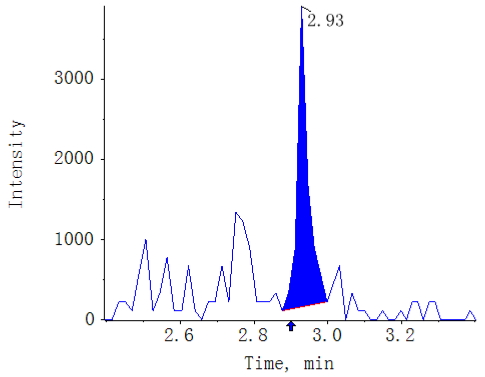

A21233260b_b
Astilbin AREA:1.19e4 S/N:20.3

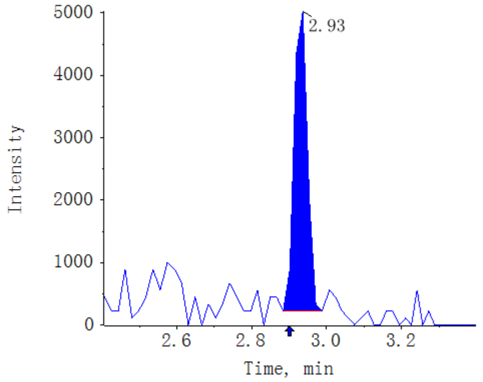

A21233261b_b
Astilbin AREA:1.46e4 S/N:16.5

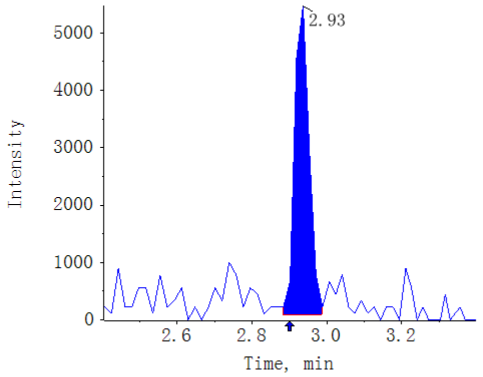

A21233262b_b
Astilbin AREA:9.53e3 S/N:18.2

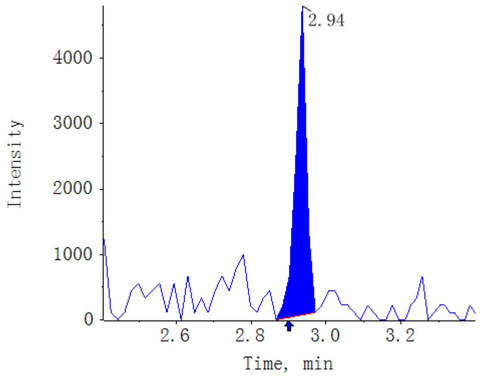

A21233263b_b
Astilbin AREA:7.27e3 S/N:15.2

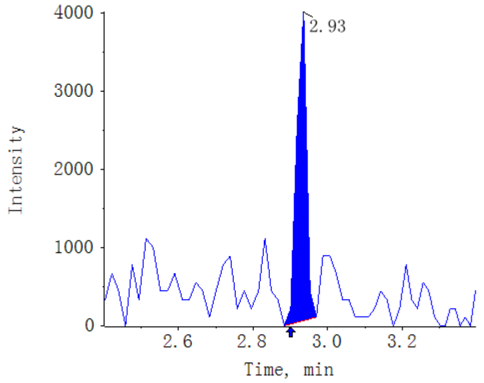

A21233264b_b
Astilbin AREA:7.38e3 S/N:10.3

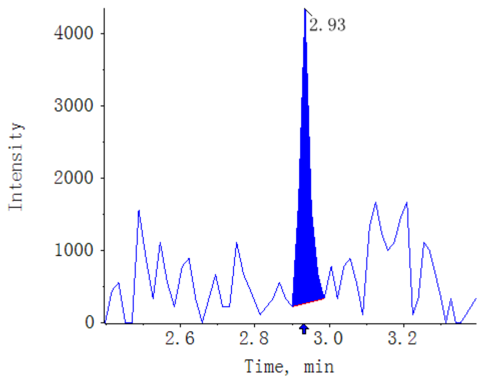

A21233265b_b
Astilbin AREA:5.36e3 S/N:6.7

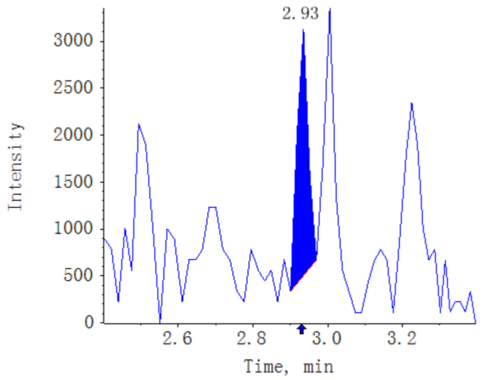

A21233266b_b
Astilbin AREA:8.54e3 S/N:12.8

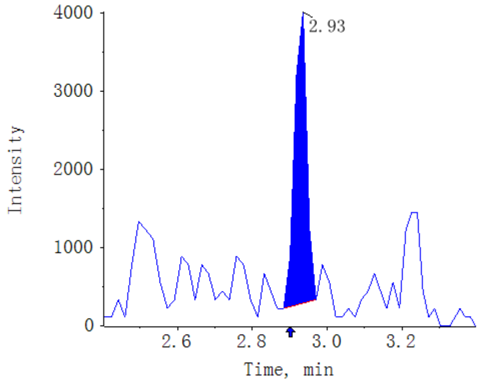

A21233267b_b
Astilbin AREA:8.26e3 S/N:12.5

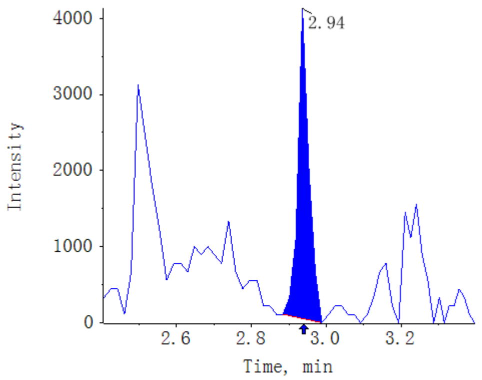

Result Table	MWXS-21-2101D-3_18_WH6500-5_A20-3_V1.0_TY_20211028	Algorithm Used	MQ4
Acquisition Method	Flavonoids_V1.0_WH6500-5_LT_20211025.dam	Instrument Name	QTRAP 6500+ Low Mass
Project	N/A	Analytes QTY	204:31

Compound name: Nicotiflorin (593.2 / 285.1)

Sample Name	Sample Type	Area (cps)	Is Area (cps)	RT (min)	S/N	Target Conc	Calculated Conc.()
STD_0.5nM	Standard	3.80e3	N/A	2.83	27.4	0.5000	3.339511e-1
STD_1nM	Standard	8.30e3	N/A	2.83	23.8	1.0000	1.041180e0
STD_5nM	Standard	4.77e4	N/A	2.83	124.4	5.0000	7.228634e0
STD_10nM	Standard	6.38e4	N/A	2.82	94.4	10.0000	9.761422e0
STD_20nM	Standard	1.16e5	N/A	2.82	83.7	20.0000	1.801543e1
STD_50nM	Standard	3.07e5	N/A	2.83	107.6	50.0000	4.800850e1
STD_100nM	Standard	6.35e5	N/A	2.83	119.8	100.0000	9.951060e1
STD_200nM	Standard	1.29e6	N/A	2.82	114.9	200.0000	2.026003e2
STD_500nM	Standard	N/A	N/A	N/A	N/A	500.0000	N/A
STD_1000nM	Standard	N/A	N/A	N/A	N/A	1000.0000	N/A
STD_2000nM	Standard	N/A	N/A	N/A	N/A	2000.0000	N/A
V1.0_MW_RQC1_20211018	Quality Control	1.49e5	N/A	2.82	1.9	0.0000	2.318610e1
Blank	Unknown	N/A	N/A	N/A	N/A	N/A	N/A
V1.0_MWMS_20211021_1	Unknown	1.17e6	N/A	2.83	132.5	N/A	1.836913e2
MWXS212101D3_R1	Quality Control	1.17e6	N/A	2.83	132.2	0.0000	1.830834e2
MWXS212101D3_R2	Quality Control	1.17e6	N/A	2.83	138.5	0.0000	1.836526e2
MWXS212101D3_R3	Quality Control	1.16e6	N/A	2.83	145.3	0.0000	1.813327e2
A21233250b_b	Unknown	5.11e7	N/A	2.83	420.6	N/A	8.026956e3
A21233251b_b	Unknown	4.24e7	N/A	2.83	347.4	N/A	6.656387e3
A21233252b_b	Unknown	3.75e7	N/A	2.83	449.4	N/A	5.894246e3
A21233253b_b	Unknown	2.46e7	N/A	2.83	421.1	N/A	3.862287e3
A21233254b_b	Unknown	1.93e7	N/A	2.83	448.1	N/A	3.029318e3
A21233255b_b	Unknown	4.08e7	N/A	2.83	441.7	N/A	6.404210e3
A21233256b_b	Unknown	2.22e7	N/A	2.83	341.5	N/A	3.485179e3
A21233257b_b	Unknown	2.89e7	N/A	2.84	465.3	N/A	4.541700e3
A21233258b_b	Unknown	1.86e7	N/A	2.83	299.6	N/A	2.913671e3
A21233259b_b	Unknown	1.06e7	N/A	2.83	375.8	N/A	1.661897e3
A21233260b_b	Unknown	4.84e7	N/A	2.82	393.3	N/A	7.594609e3
A21233261b_b	Unknown	2.69e7	N/A	2.83	343.4	N/A	4.217960e3
A21233262b_b	Unknown	2.40e7	N/A	2.83	330.1	N/A	3.771207e3
A21233263b_b	Unknown	2.25e7	N/A	2.82	465.5	N/A	3.535343e3
A21233264b_b	Unknown	1.30e7	N/A	2.83	431.0	N/A	2.038142e3
A21233265b_b	Unknown	3.64e7	N/A	2.83	451.8	N/A	5.717229e3
A21233266b_b	Unknown	1.86e7	N/A	2.82	410.5	N/A	2.919524e3
A21233267b_b	Unknown	2.11e7	N/A	2.84	390.4	N/A	3.308238e3

Compound name: Nicotiflorin
Regression Equation: y = 6368.48298 x + 1672.21495 (r = 0.99814) (weighting: 1 / x)

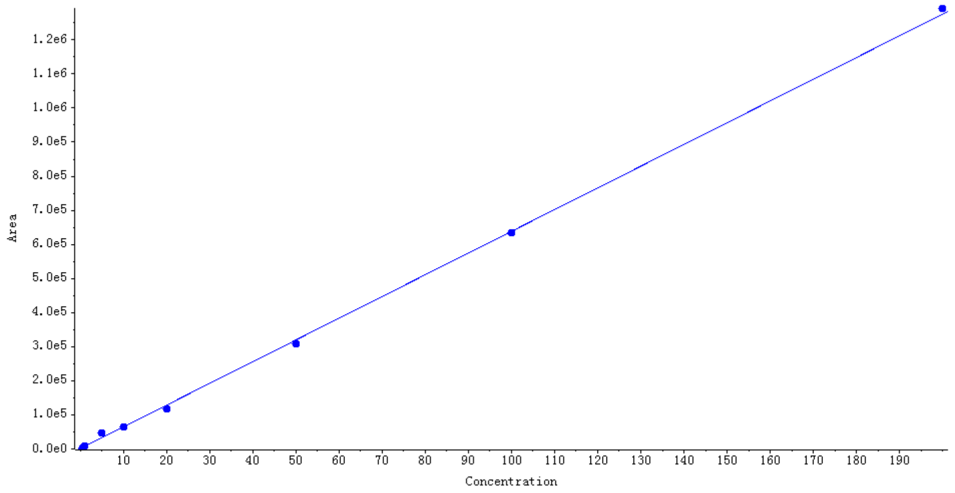

Peak Review

Blank
Nicotiflorin AREA:N/A S/N:N/A

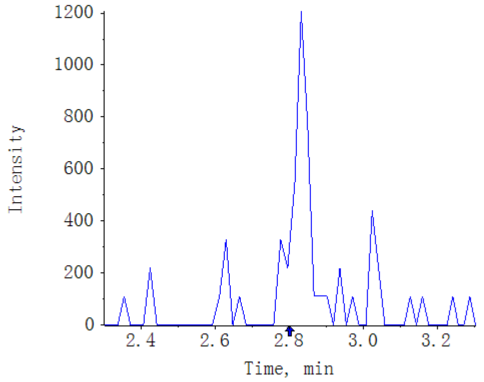

V1.0_MWMS_20211021_1
Nicotiflorin AREA:1.17e6
S/N:132.5

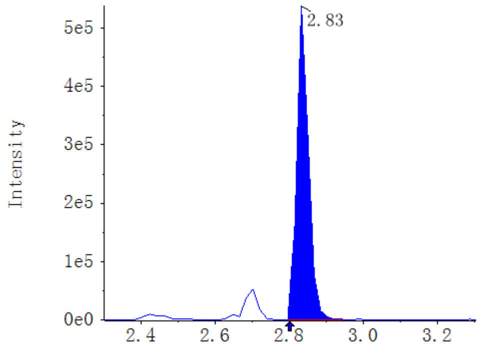

A21233250b_b
Nicotiflorin AREA:5.11e7
S/N:420.6

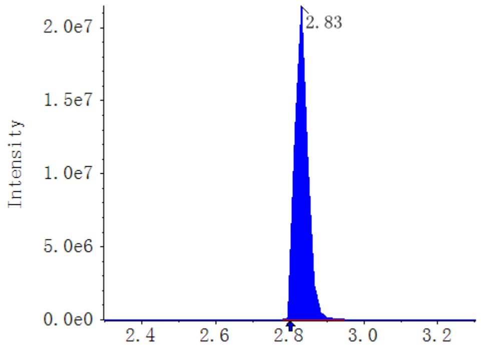

A21233251b_b
Nicotiflorin AREA:4.24e7
S/N:347.4

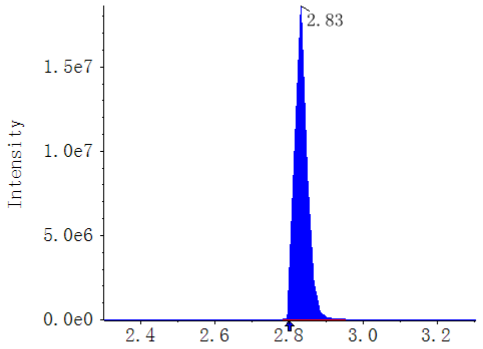

A21233252b_b
Nicotiflorin AREA:3.75e7
S/N:449.4

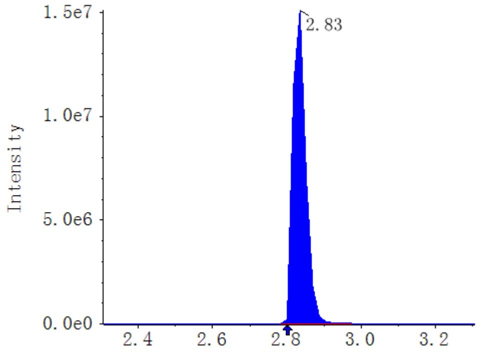

A21233253b_b
Nicotiflorin AREA:2.46e7
S/N:421.1

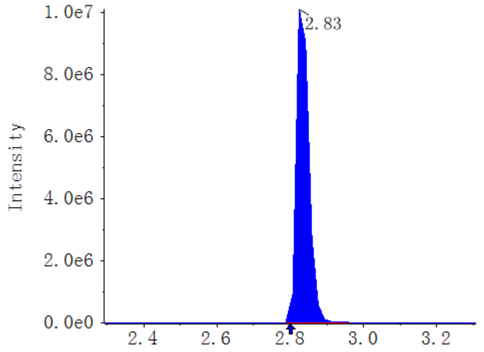

A21233254b_b
Nicotiflorin AREA:1.93e7
S/N:448.1

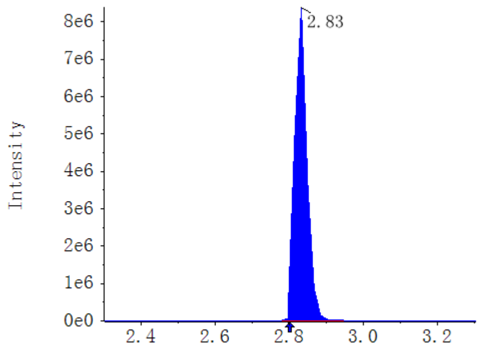

A21233255b_b
Nicotiflorin AREA:4.08e7
S/N:441.7

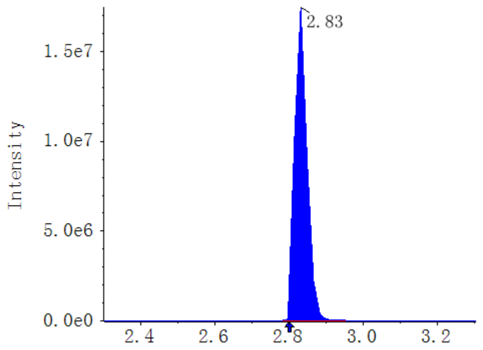

A21233256b_b
Nicotiflorin AREA:2.22e7
S/N:341.5

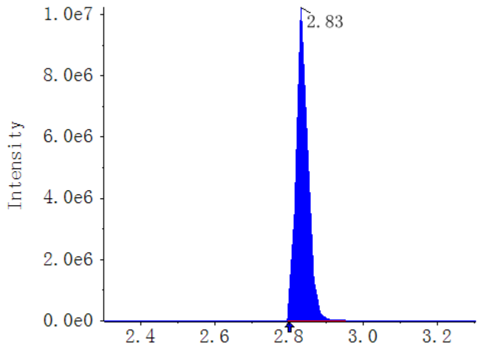

A21233257b_b
Nicotiflorin AREA:2.89e7
S/N:465.3

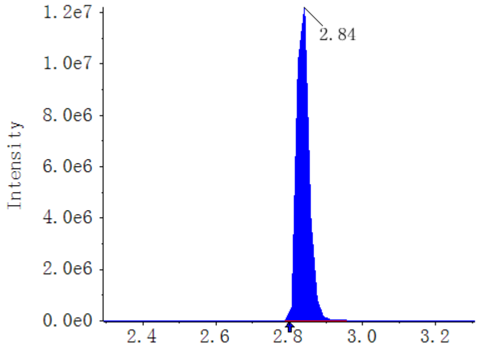

A21233258b_b
Nicotiflorin AREA:1.86e7
S/N:299.6

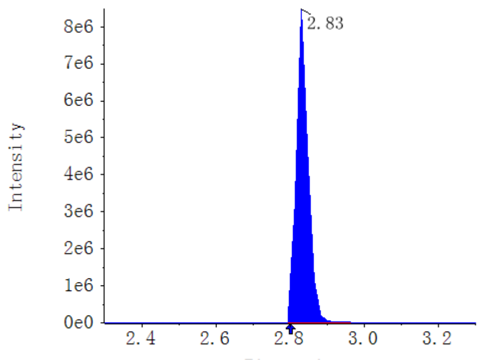

A21233259b_b
Nicotiflorin AREA:1.06e7
S/N:375.8

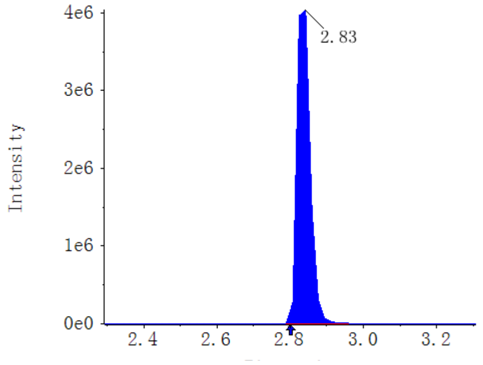

A21233260b_b
Nicotiflorin AREA:4.84e7
S/N:393.3

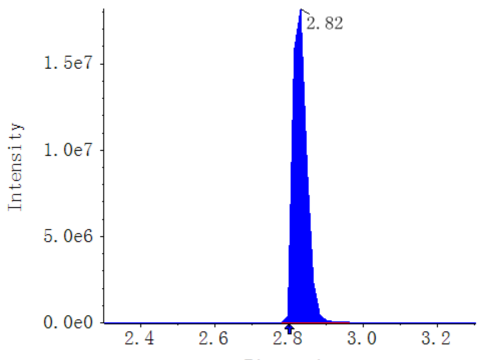

A21233261b_b
Nicotiflorin AREA:2.69e7
S/N:343.4

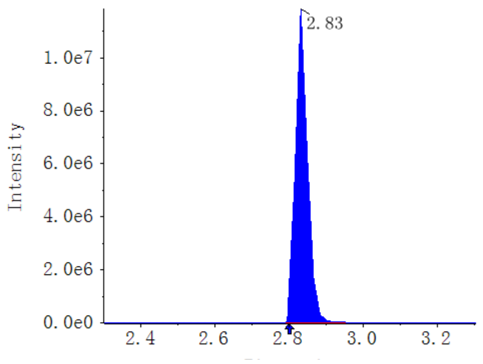

A21233262b_b
Nicotiflorin AREA:2.40e7
S/N:330.1

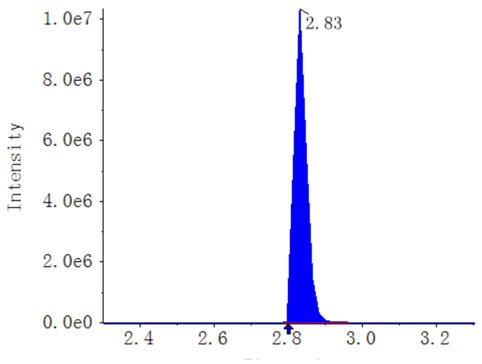

A21233263b_b
Nicotiflorin AREA:2.25e7
S/N:465.5

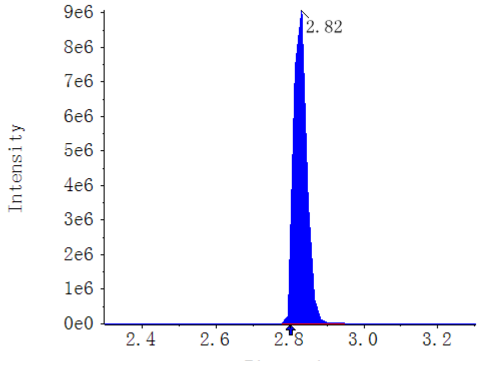

A21233264b_b
Nicotiflorin AREA:1.30e7
S/N:431.0

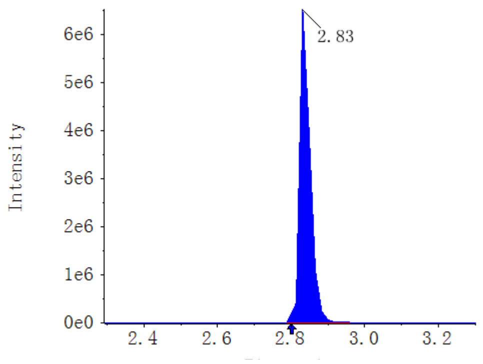

A21233265b_b
Nicotiflorin AREA:3.64e7
S/N:451.8

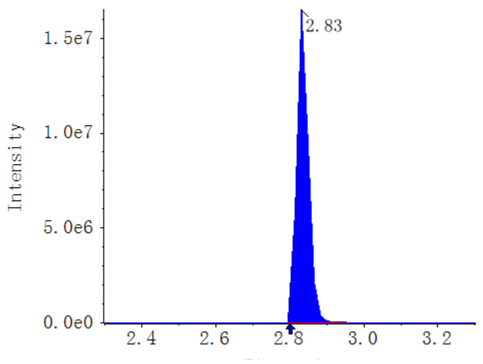

A21233266b_b
Nicotiflorin AREA:1.86e7
S/N:410.5

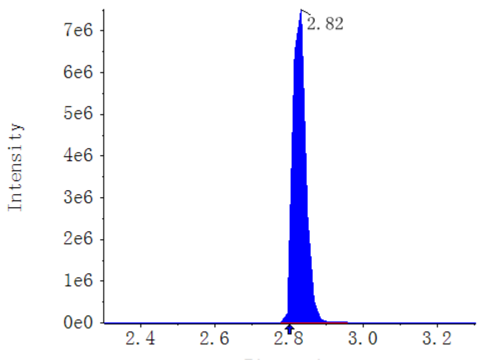

A21233267b_b
Nicotiflorin AREA:2.11e7
S/N:390.4

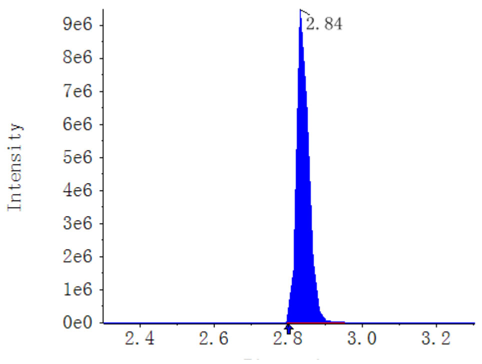

**Result Table**	MWXS-21-2101D-3_18_WH6500-5_A20-3_V1.0_TY_20211028	**Algorithm Used**	MQ4
**Acquisition Method**	Flavonoids_V1.0_WH6500-5_LT_20211025.dam	**Instrument Name**	QTRAP 6500+ Low Mass
**Project**	N/A	**Analytes QTY**	204:45

Compound name: Rutin (609.1 / 300.0)

Sample Name	Sample Type	Area (cps)	Is Area (cps)	RT (min)	S/N	Target Conc	Calculated Conc.()
STD_0.5nM	Standard	3.92e4	N/A	2.64	58.1	0.5000	4.615796e-1
STD_1nM	Standard	4.42e4	N/A	2.63	68.4	1.0000	8.993578e-1
STD_5nM	Standard	1.09e5	N/A	2.63	97.8	5.0000	6.573130e0
STD_10nM	Standard	1.41e5	N/A	2.63	143.8	10.0000	9.385678e0
STD_20nM	Standard	2.52e5	N/A	2.63	141.8	20.0000	1.910764e1
STD_50nM	Standard	5.88e5	N/A	2.63	187.0	50.0000	4.867384e1
STD_100nM	Standard	1.15e6	N/A	2.64	212.0	100.0000	9.768732e1
STD_200nM	Standard	2.35e6	N/A	2.63	200.6	200.0000	2.037115e2
STD_500nM	Standard	N/A	N/A	N/A	N/A	500.0000	N/A
STD_1000nM	Standard	N/A	N/A	N/A	N/A	1000.0000	N/A
STD_2000nM	Standard	N/A	N/A	N/A	N/A	2000.0000	N/A
V1.0_MW_RQC1_20211018	Quality Control	8.20e5	N/A	2.63	137.7	0.0000	6.901856e1
Blank	Unknown	N/A	N/A	N/A	N/A	N/A	N/A
V1.0_MWMS_20211021_1	Unknown	1.97e6	N/A	2.64	219.5	N/A	1.700358e2
MWXS212101D3_R1	Quality Control	1.92e6	N/A	2.63	225.7	0.0000	1.657062e2
MWXS212101D3_R2	Quality Control	1.86e6	N/A	2.64	239.2	0.0000	1.604887e2
MWXS212101D3_R3	Quality Control	1.98e6	N/A	2.63	257.7	0.0000	1.708168e2
A21233250b_b	Unknown	9.53e7	N/A	2.63	401.0	N/A	8.366871e3
A21233251b_b	Unknown	1.54e8	N/A	2.64	379.4	N/A	1.353091e4
A21233252b_b	Unknown	1.38e8	N/A	2.64	348.9	N/A	1.211033e4
A21233253b_b	Unknown	1.39e8	N/A	2.64	320.4	N/A	1.217566e4
A21233254b_b	Unknown	1.21e8	N/A	2.63	339.7	N/A	1.058986e4
A21233255b_b	Unknown	1.17e8	N/A	2.64	382.7	N/A	1.025373e4
A21233256b_b	Unknown	1.13e8	N/A	2.64	378.7	N/A	9.909270e3
A21233257b_b	Unknown	1.23e8	N/A	2.64	346.9	N/A	1.081551e4
A21233258b_b	Unknown	9.90e7	N/A	2.64	371.4	N/A	8.687132e3
A21233259b_b	Unknown	3.74e7	N/A	2.64	401.6	N/A	3.282487e3
A21233260b_b	Unknown	1.05e8	N/A	2.64	396.0	N/A	9.225258e3
A21233261b_b	Unknown	7.87e7	N/A	2.64	384.3	N/A	6.908030e3
A21233262b_b	Unknown	6.94e7	N/A	2.64	374.3	N/A	6.090564e3
A21233263b_b	Unknown	1.19e8	N/A	2.63	386.0	N/A	1.045570e4
A21233264b_b	Unknown	9.22e7	N/A	2.64	366.4	N/A	8.090494e3
A21233265b_b	Unknown	1.19e8	N/A	2.64	331.0	N/A	1.044263e4
A21233266b_b	Unknown	1.02e8	N/A	2.63	356.4	N/A	8.911181e3
A21233267b_b	Unknown	9.83e7	N/A	2.64	367.3	N/A	8.629191e3

Compound name: Rutin

Regression Equation: y = 11389.72468 x + 3.39152e4 (r = 0.99899) (weighting: 1 / x)

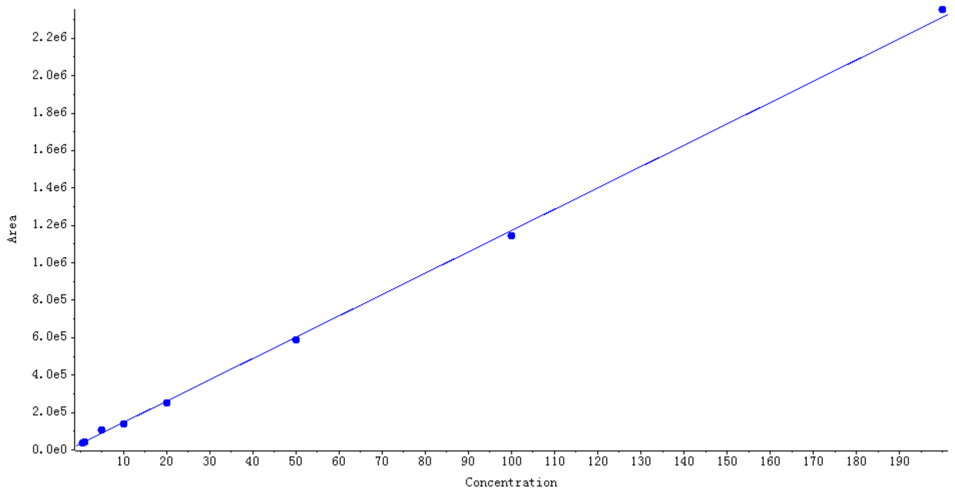

Peak Review

Blank
Rutin AREA:N/A S/N:N/A

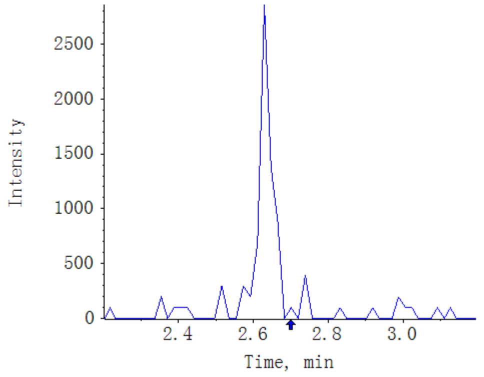

V1.0_MWMS_20211021_1
Rutin AREA:1.97e6 S/N:219.5

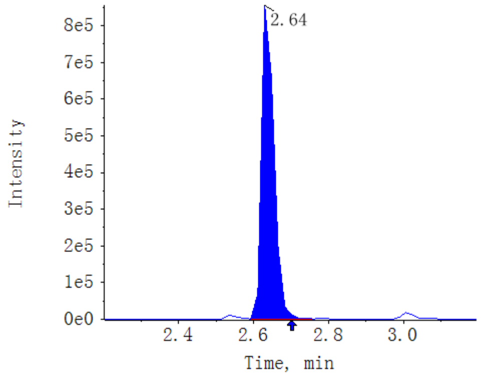

A21233250b_b
Rutin AREA:9.53e7 S/N:401.0

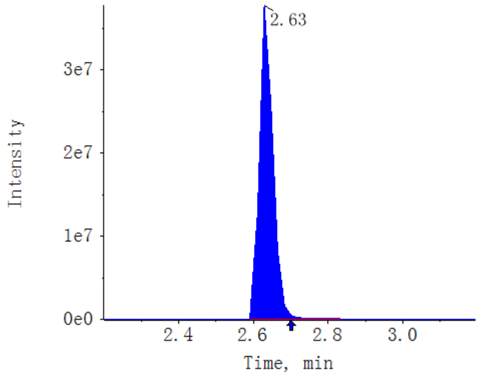

A21233251b_b
Rutin AREA:1.54e8 S/N:379.4

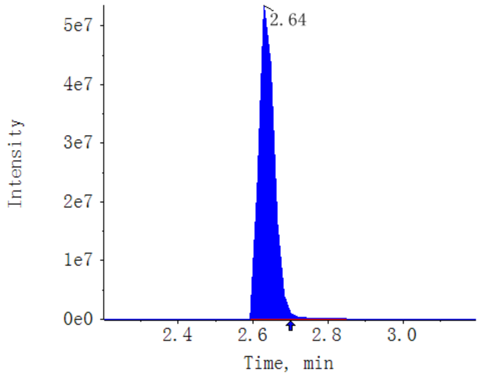

A21233252b_b
Rutin AREA:1.38e8 S/N:348.9

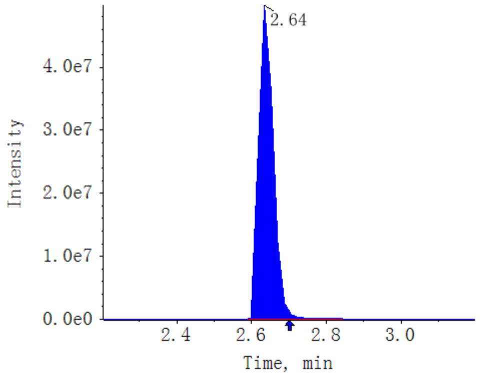

A21233253b_b
Rutin AREA:1.39e8 S/N:320.4

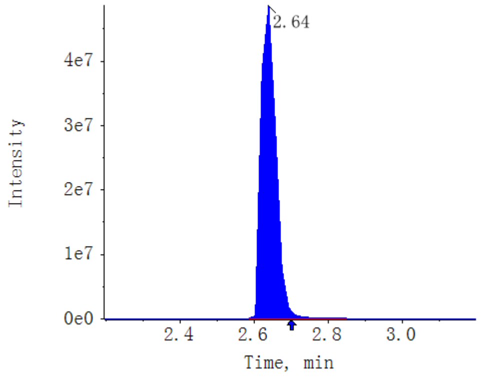

A21233254b_b
Rutin AREA:1.21e8 S/N:339.7

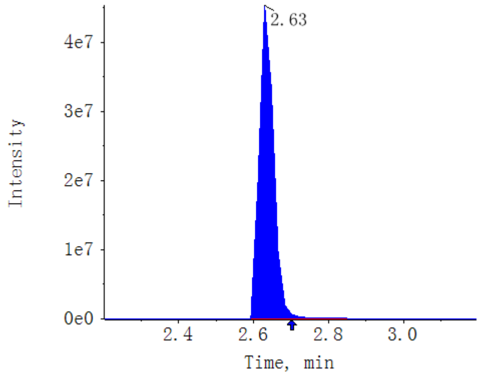

A21233255b_b
Rutin AREA:1.17e8 S/N:382.7

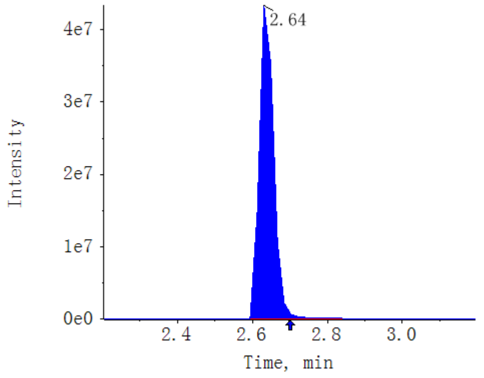

A21233256b_b
Rutin AREA:1.13e8 S/N:378.7

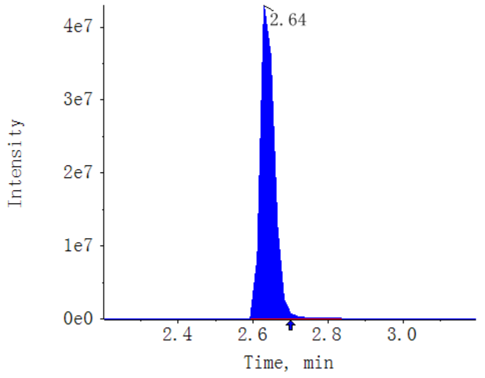

A21233257b_b
Rutin AREA:1.23e8 S/N:346.9

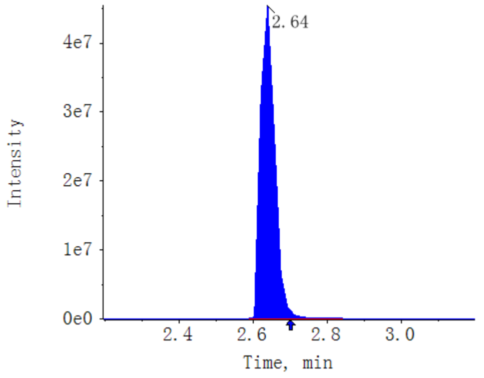

A21233258b_b
Rutin AREA:9.90e7 S/N:371.4

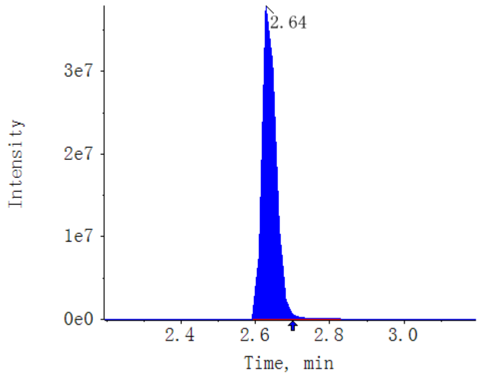

A21233259b_b
Rutin AREA:3.74e7 S/N:401.6

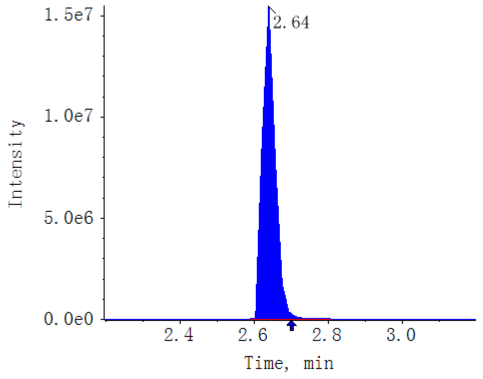

A21233260b_b
Rutin AREA:1.05e8 S/N:396.0

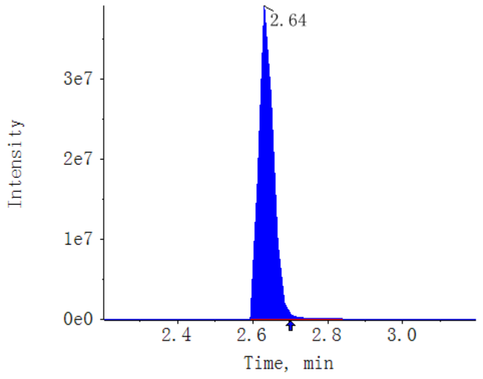

A21233261b_b
Rutin AREA:7.87e7 S/N:384.3

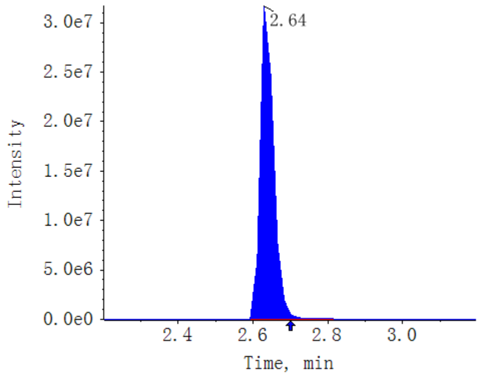

A21233262b_b
Rutin AREA:6.94e7 S/N:374.3

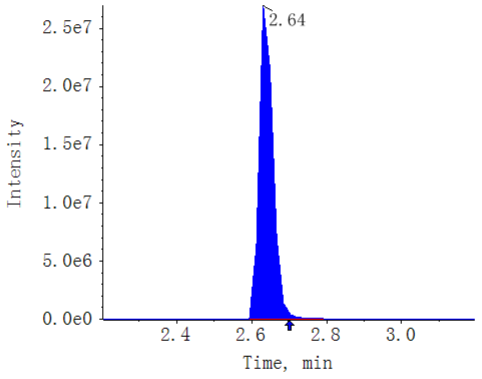

A21233263b_b
Rutin AREA:1.19e8 S/N:386.0

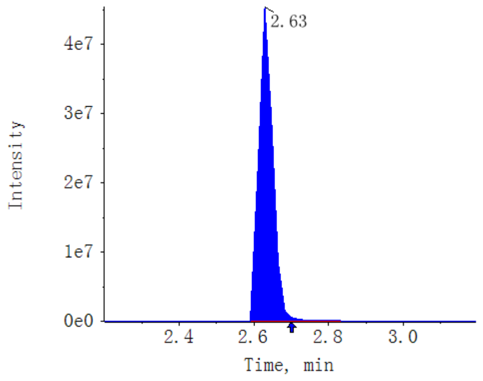

A21233264b_b
Rutin AREA:9.22e7 S/N:366.4

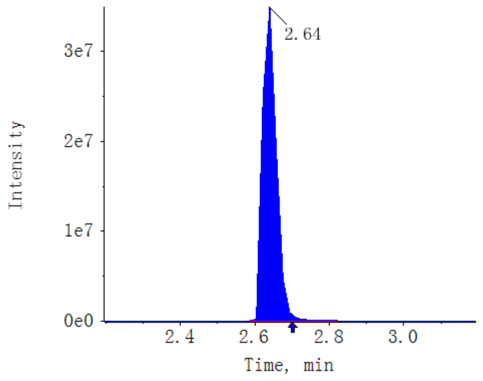

A21233265b_b
Rutin AREA:1.19e8 S/N:331.0

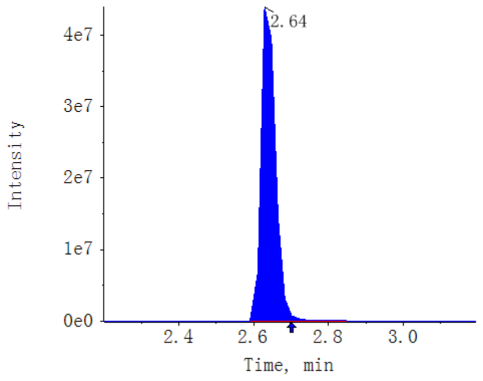

A21233266b_b
Rutin AREA:1.02e8 S/N:356.4

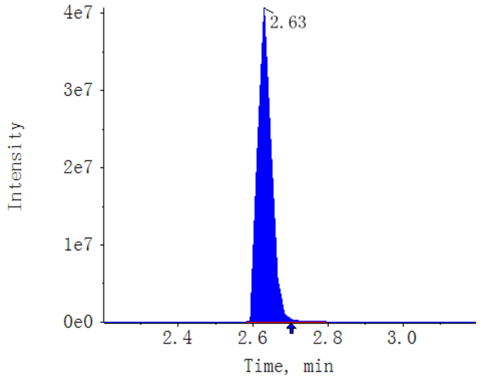

A21233267b_b
Rutin AREA:9.83e7 S/N:367.3

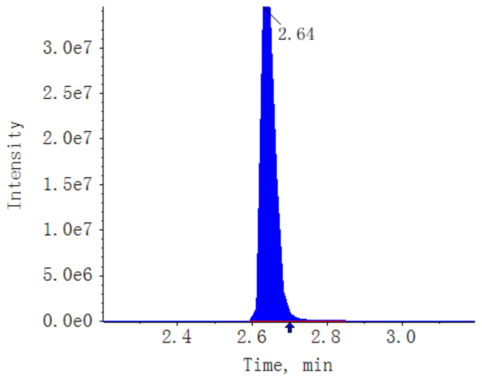

Result Table	MWXS-21-2101D-3_18_WH6500-5_A20-3_V1.0_TY_20211028	Algorithm Used	MQ4
Acquisition Method	Flavonoids_V1.0_WH6500-5_LT_20211025.dam	Instrument Name	QTRAP 6500+ Low Mass
Project	N/A	Analytes QTY	204:53

Compound name: Phlorizin (435.1 / 273.1)

Sample Name	Sample Type	Area (cps)	Is Area (cps)	RT (min)	S/N	Target Conc	Calculated Conc.()
STD_0.5nM	Standard	7.41e3	N/A	3.27	34.4	0.5000	3.382499e-1
STD_1nM	Standard	1.61e4	N/A	3.27	44.4	1.0000	8.166601e-1
STD_5nM	Standard	1.36e5	N/A	3.27	88.4	5.0000	7.411817e0
STD_10nM	Standard	1.82e5	N/A	3.27	150.0	10.0000	9.953311e0
STD_20nM	Standard	3.76e5	N/A	3.26	161.3	20.0000	2.063693e1
STD_50nM	Standard	9.29e5	N/A	3.27	208.7	50.0000	5.105564e1
STD_100nM	Standard	1.80e6	N/A	3.28	157.6	100.0000	9.894983e1
STD_200nM	Standard	3.59e6	N/A	3.27	144.7	200.0000	1.973376e2
STD_500nM	Standard	N/A	N/A	N/A	N/A	500.0000	N/A
STD_1000nM	Standard	N/A	N/A	N/A	N/A	1000.0000	N/A
STD_2000nM	Standard	N/A	N/A	N/A	N/A	2000.0000	N/A
V1.0_MW_RQC1_20211018	Quality Control	1.71e4	N/A	3.27	4.4	0.0000	8.733864e-1
Blank	Unknown	N/A	N/A	N/A	N/A	N/A	N/A
V1.0_MWMS_20211021_1	Unknown	3.00e6	N/A	3.28	210.4	N/A	1.648067e2
MWXS212101D3_R1	Quality Control	3.14e6	N/A	3.28	181.0	0.0000	1.725797e2
MWXS212101D3_R2	Quality Control	3.08e6	N/A	3.28	176.4	0.0000	1.694434e2
MWXS212101D3_R3	Quality Control	2.94e6	N/A	3.28	193.6	0.0000	1.618024e2
A21233250b_b	Unknown	1.30e4	N/A	3.28	5.3	N/A	6.469493e-1
A21233251b_b	Unknown	1.87e4	N/A	3.28	10.5	N/A	9.590136e-1
A21233252b_b	Unknown	3.33e4	N/A	3.28	15.8	N/A	1.764722e0
A21233253b_b	Unknown	9.72e4	N/A	3.28	10.5	N/A	5.276954e0
A21233254b_b	Unknown	7.11e4	N/A	3.28	9.4	N/A	3.844152e0
A21233255b_b	Unknown	9.11e4	N/A	3.28	10.3	N/A	4.944571e0
A21233256b_b	Unknown	1.73e5	N/A	3.28	9.7	N/A	9.429290e0
A21233257b_b	Unknown	1.89e5	N/A	3.28	8.2	N/A	1.034601e1
A21233258b_b	Unknown	1.77e5	N/A	3.28	10.8	N/A	9.692834e0
A21233259b_b	Unknown	6.27e3	N/A	3.28	3.8	N/A	2.754643e-1
A21233260b_b	Unknown	1.14e4	N/A	3.28	8.4	N/A	5.555841e-1
A21233261b_b	Unknown	6.17e3	N/A	3.28	3.0	N/A	2.700232e-1
A21233262b_b	Unknown	2.22e4	N/A	3.28	6.8	N/A	1.151978e0
A21233263b_b	Unknown	9.88e4	N/A	3.28	8.5	N/A	5.367983e0
A21233264b_b	Unknown	6.87e4	N/A	3.28	9.7	N/A	3.708383e0
A21233265b_b	Unknown	2.13e5	N/A	3.28	9.6	N/A	1.164980e1
A21233266b_b	Unknown	1.84e5	N/A	3.27	11.0	N/A	1.005688e1
A21233267b_b	Unknown	2.62e5	N/A	3.29	12.7	N/A	1.432242e1

Compound name: Phlorizin
Regression Equation: y = 18173.36711 x + 1265.23169 (r = 0.99818) (weighting: 1 / x)

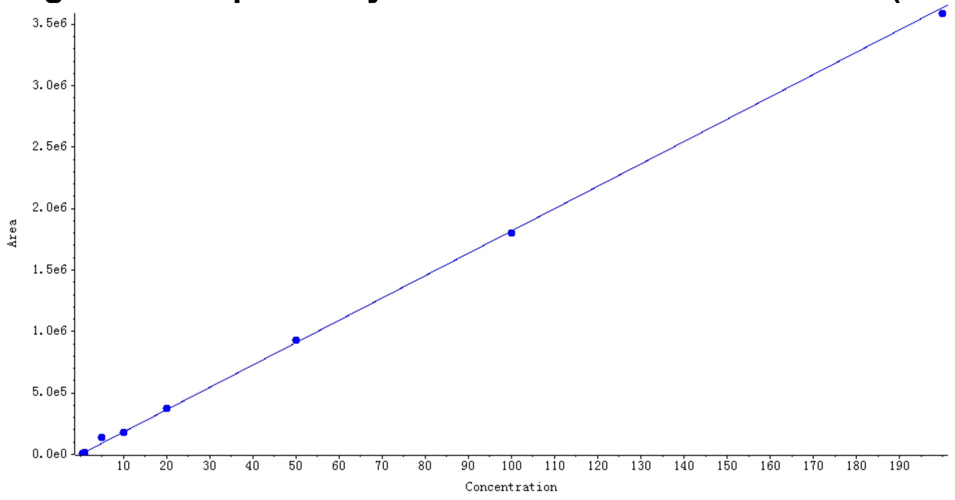

Peak Review

Blank
Phlorizin AREA:N/A S/N:N/A

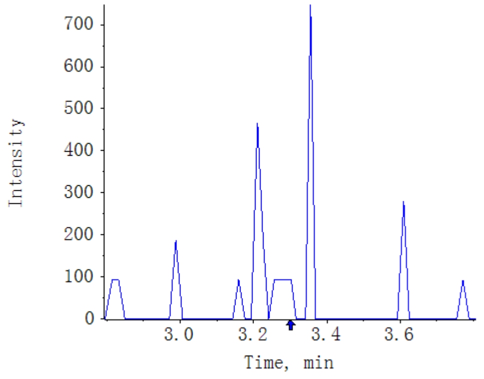

V1.0_MWMS_20211021_1
Phlorizin AREA:3.00e6 S/N:210.4

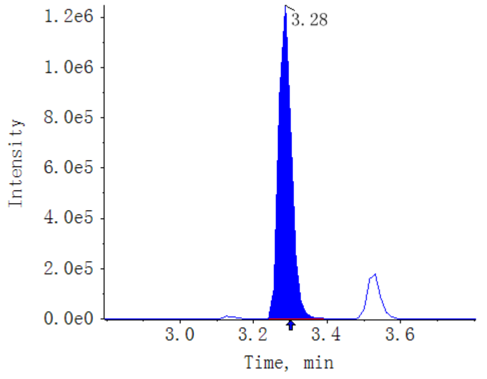

A21233250b_b
Phlorizin AREA:1.30e4 S/N:5.3

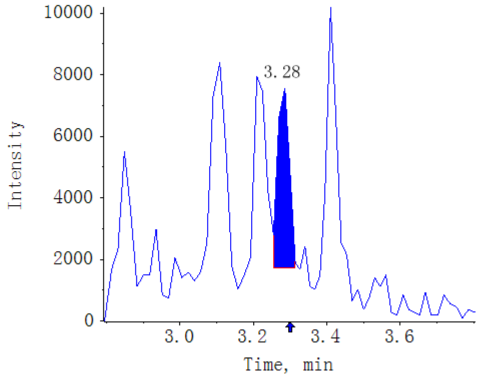

A21233251b_b
Phlorizin AREA:1.87e4 S/N:10.5

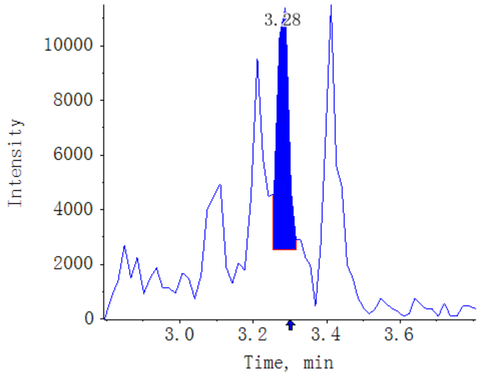

A21233252b_b
Phlorizin AREA:3.33e4 S/N:15.8

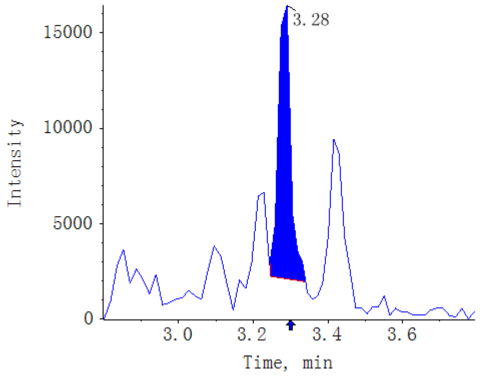

A21233253b_b
Phlorizin AREA:9.72e4 S/N:10.5

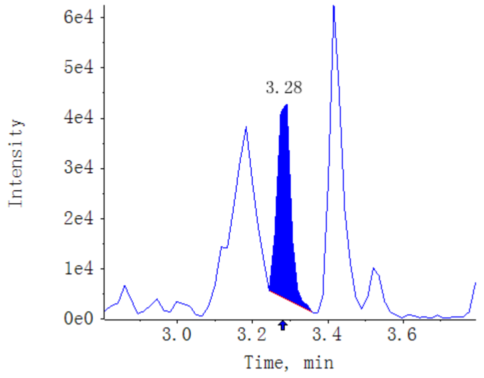

A21233254b_b
Phlorizin AREA:7.11e4 S/N:9.4

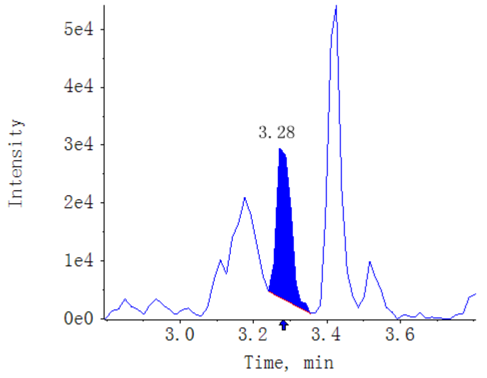

A21233255b_b
Phlorizin AREA:9.11e4 S/N:10.3

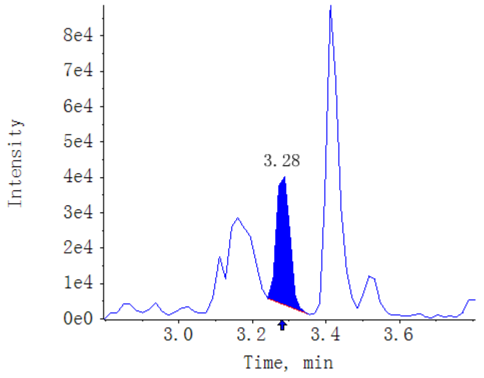

A21233256b_b
Phlorizin AREA:1.73e5 S/N:9.7

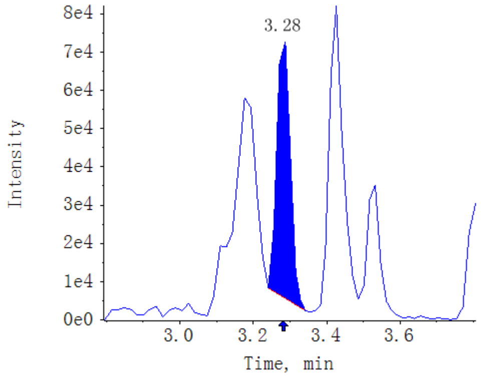

A21233257b_b
Phlorizin AREA:1.89e5 S/N:8.2

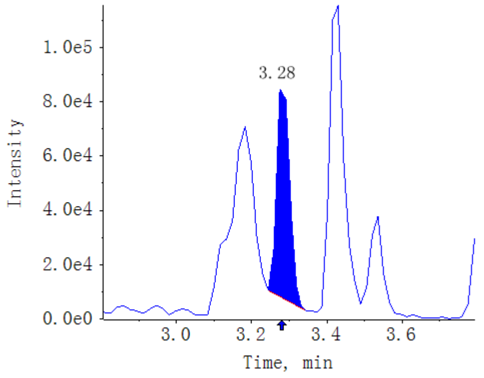

A21233258b_b
Phlorizin AREA:1.77e5 S/N:10.8

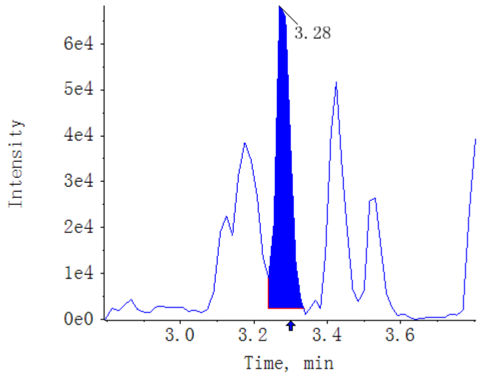

A21233259b_b
Phlorizin AREA:6.27e3 S/N:3.8

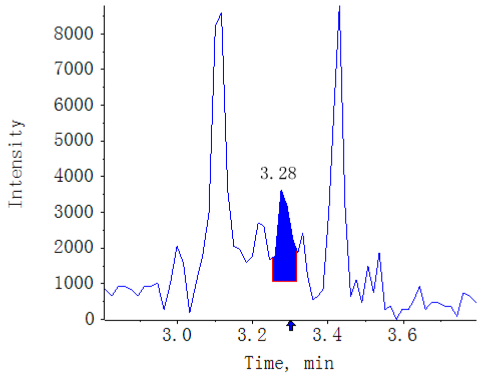

A21233260b_b
Phlorizin AREA:1.14e4 S/N:8.4

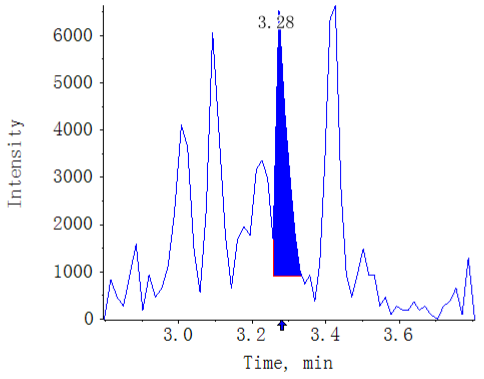

A21233261b_b
Phlorizin AREA:6.17e3 S/N:3.0

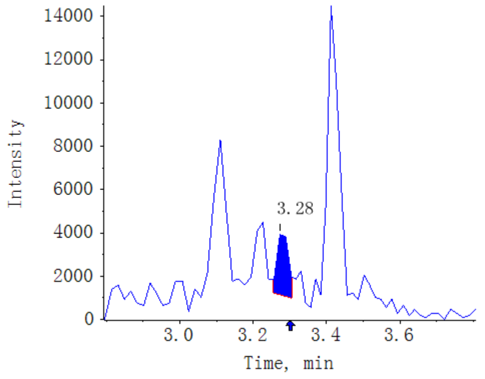

A21233262b_b
Phlorizin AREA:2.22e4 S/N:6.8

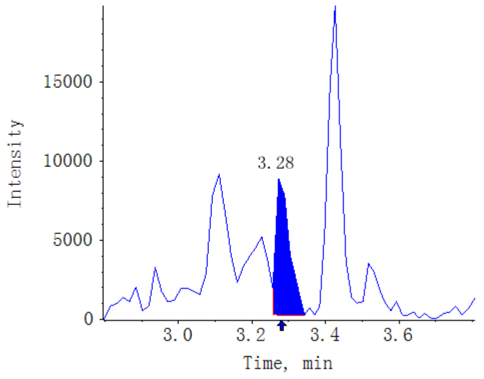

A21233263b_b
Phlorizin AREA:9.88e4 S/N:8.5

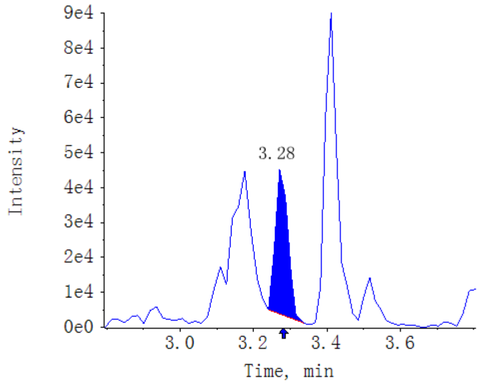

A21233264b_b
Phlorizin AREA:6.87e4 S/N:9.7

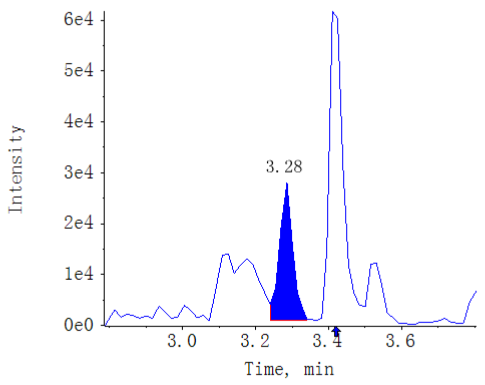

A21233265b_b
Phlorizin AREA:2.13e5 S/N:9.6

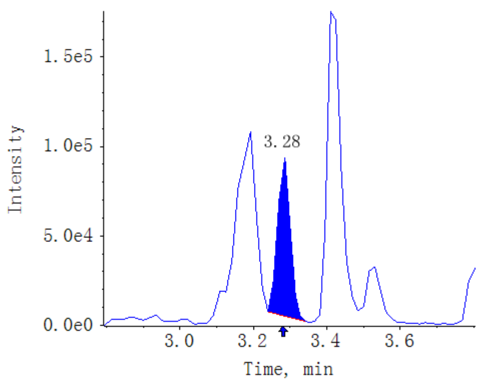

A21233266b_b
Phlorizin AREA:1.84e5 S/N:11.0

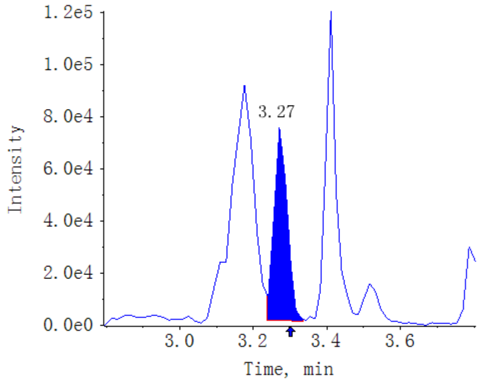

A21233267b_b
Phlorizin AREA:2.62e5 S/N:12.7

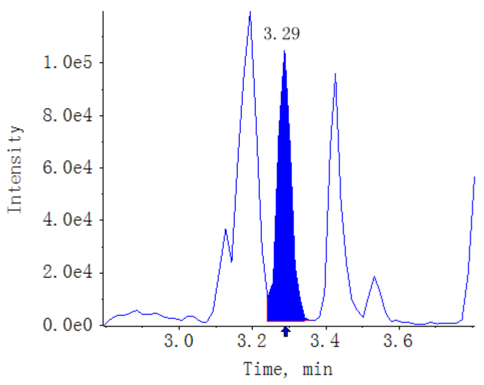

Result Table	MWXS-21-2101D-3_18_WH6500-5_A20-3_V1.0_TY_20211028	Algorithm Used	MQ4
Acquisition Method	Flavonoids_V1.0_WH6500-5_LT_20211025.dam	Instrument Name	QTRAP 6500+ Low Mass
Project	N/A	Analytes QTY	204:57

Compound name: Liquiritin (417.1 / 255.1)

Sample Name	Sample Type	Area (cps)	Is Area (cps)	RT (min)	S/N	Target Conc	Calculated Conc.()
STD_0.5nM	Standard	1.01e4	N/A	2.79	43.6	0.5000	4.429427e-1
STD_1nM	Standard	1.37e4	N/A	2.78	51.7	1.0000	6.563204e-1
STD_5nM	Standard	1.20e5	N/A	2.79	150.4	5.0000	7.066923e0
STD_10nM	Standard	1.71e5	N/A	2.79	231.7	10.0000	1.015961e1
STD_20nM	Standard	3.37e5	N/A	2.79	312.9	20.0000	2.016430e1
STD_50nM	Standard	8.75e5	N/A	2.79	342.9	50.0000	5.269262e1
STD_100nM	Standard	1.62e6	N/A	2.79	496.8	100.0000	9.795901e1
STD_200nM	Standard	3.27e6	N/A	2.78	487.7	200.0000	1.973583e2
STD_500nM	Standard	N/A	N/A	N/A	N/A	500.0000	N/A
STD_1000nM	Standard	N/A	N/A	N/A	N/A	1000.0000	N/A
STD_2000nM	Standard	N/A	N/A	N/A	N/A	2000.0000	N/A
V1.0_MW_RQC1_20211018	Quality Control	N/A	N/A	N/A	N/A	0.0000	N/A
Blank	Unknown	N/A	N/A	N/A	N/A	N/A	N/A
V1.0_MWMS_20211021_1	Unknown	2.68e6	N/A	2.80	463.7	N/A	1.615042e2
MWXS212101D3_R1	Quality Control	2.76e6	N/A	2.79	503.3	0.0000	1.663836e2
MWXS212101D3_R2	Quality Control	2.67e6	N/A	2.80	430.3	0.0000	1.609183e2
MWXS212101D3_R3	Quality Control	2.61e6	N/A	2.79	386.9	0.0000	1.572545e2
A21233250b_b	Unknown	1.32e4	N/A	2.72	8.1	N/A	6.307933e-1
A21233251b_b	Unknown	6.24e3	N/A	2.72	5.5	N/A	2.076943e-1
A21233252b_b	Unknown	8.05e3	N/A	2.72	5.3	N/A	3.172114e-1
A21233253b_b	Unknown	2.02e4	N/A	2.71	8.9	N/A	1.052724e0
A21233254b_b	Unknown	1.88e4	N/A	2.72	8.7	N/A	9.670657e-1
A21233255b_b	Unknown	2.34e4	N/A	2.71	10.0	N/A	1.245643e0
A21233256b_b	Unknown	2.14e4	N/A	2.72	4.5	N/A	1.125849e0
A21233257b_b	Unknown	3.18e4	N/A	2.73	3.4	N/A	1.749890e0
A21233258b_b	Unknown	1.48e4	N/A	2.72	1.8	N/A	7.259021e-1
A21233259b_b	Unknown	7.09e3	N/A	2.73	5.1	N/A	2.592523e-1
A21233260b_b	Unknown	8.31e3	N/A	2.70	8.6	N/A	3.328778e-1
A21233261b_b	Unknown	1.04e4	N/A	2.72	8.1	N/A	4.568581e-1
A21233262b_b	Unknown	1.46e4	N/A	2.72	9.1	N/A	7.111704e-1
A21233263b_b	Unknown	2.06e4	N/A	2.71	9.5	N/A	1.072169e0
A21233264b_b	Unknown	1.92e4	N/A	2.71	7.7	N/A	9.874053e-1
A21233265b_b	Unknown	2.44e4	N/A	2.71	5.7	N/A	1.304340e0
A21233266b_b	Unknown	2.14e4	N/A	2.71	6.0	N/A	1.120571e0
A21233267b_b	Unknown	2.47e4	N/A	2.71	3.5	N/A	1.323846e0

Compound name: Liquiritin

Regression Equation: y = 16559.77809 x + 2799.66502 (r = 0.99837) (weighting: 1 / x)

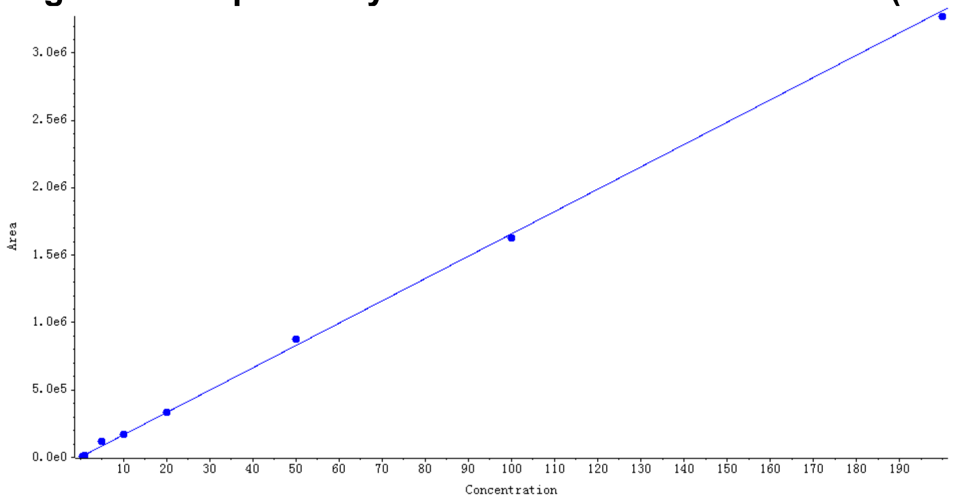

Peak Review

Blank
Liquiritin AREA:N/A S/N:N/A

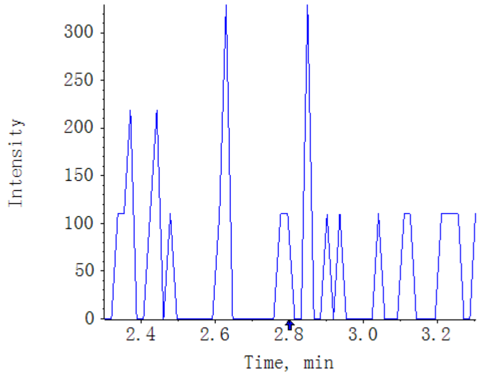

V1.0_MWMS_20211021_1
Liquiritin AREA:2.68e6 S/N:463.7

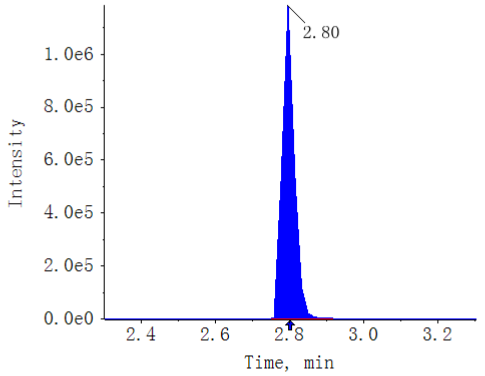

A21233250b_b
Liquiritin AREA:1.32e4 S/N:8.1

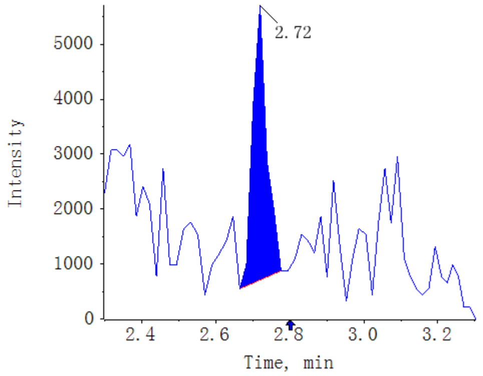

A21233251b_b
Liquiritin AREA:6.24e3 S/N:5.5

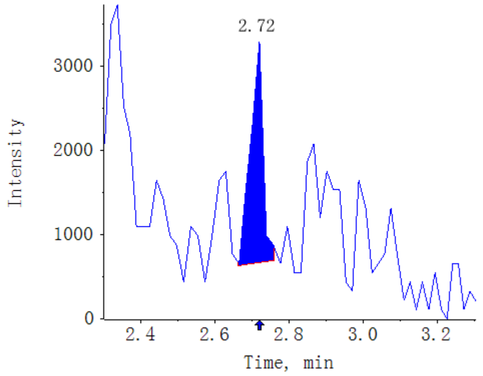

A21233252b_b
Liquiritin AREA:8.05e3 S/N:5.3

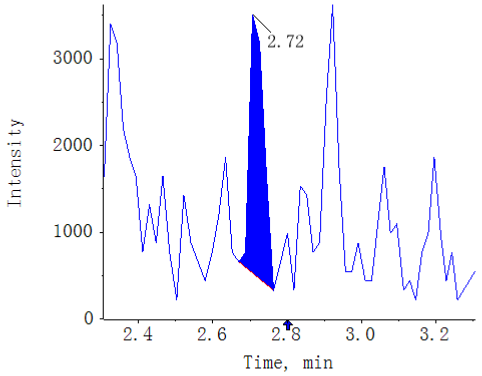

A21233253b_b
Liquiritin AREA:2.02e4 S/N:8.9

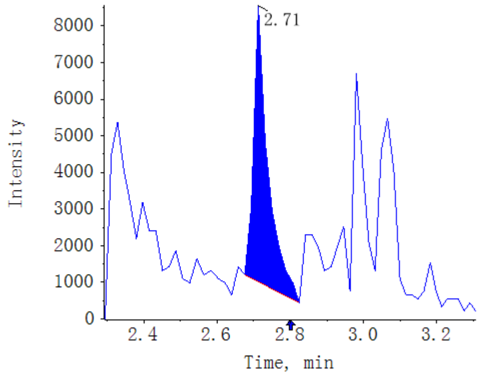

A21233254b_b
Liquiritin AREA:1.88e4 S/N:8.7

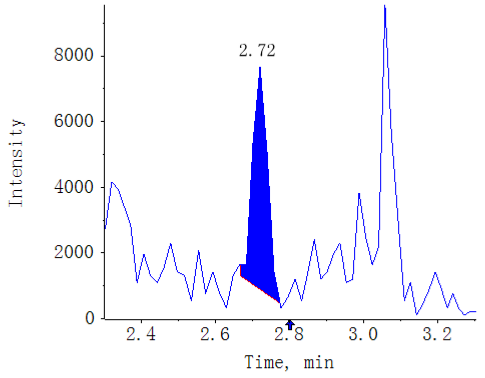

A21233255b_b
Liquiritin AREA:2.34e4 S/N:10.0

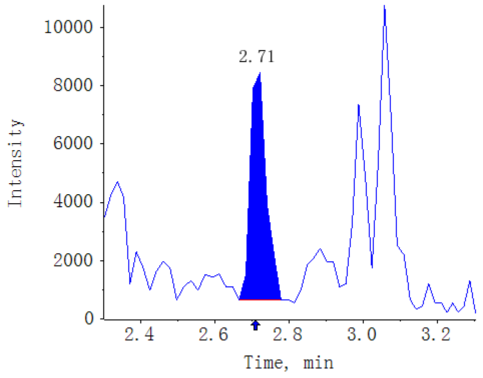

A21233256b_b
Liquiritin AREA:2.14e4 S/N:4.5

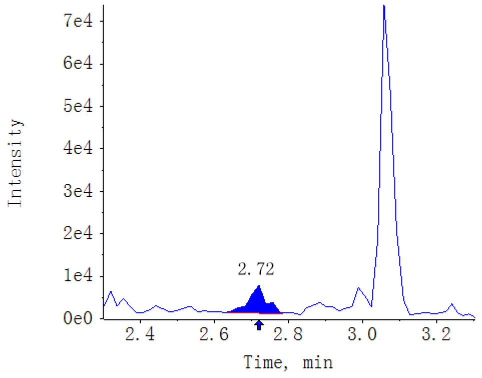

A21233257b_b
Liquiritin AREA:3.18e4 S/N:3.4

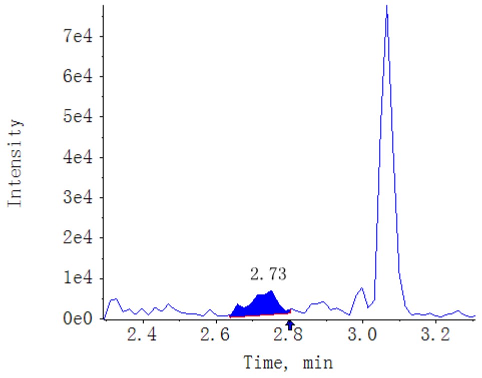

A21233258b_b
Liquiritin AREA:1.48e4 S/N:1.8

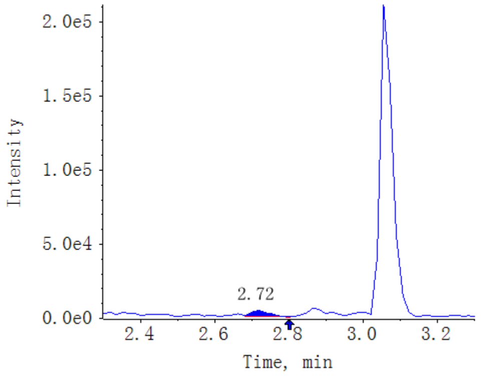

A21233259b_b
Liquiritin AREA:7.09e3 S/N:5.1

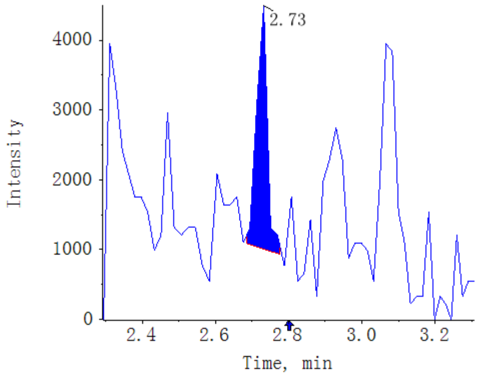

A21233260b_b
Liquiritin AREA:8.31e3 S/N:8.6

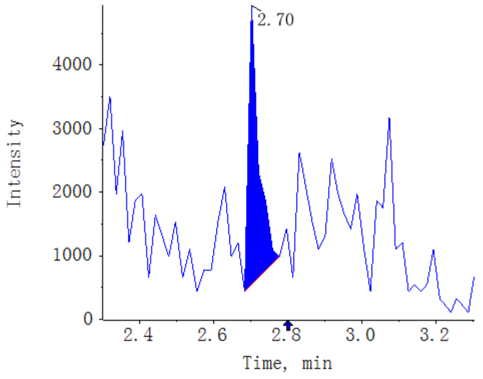

A21233261b_b
Liquiritin AREA:1.04e4 S/N:8.1

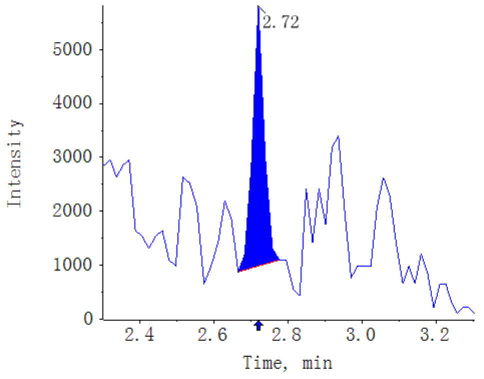

A21233262b_b
Liquiritin AREA:1.46e4 S/N:9.1

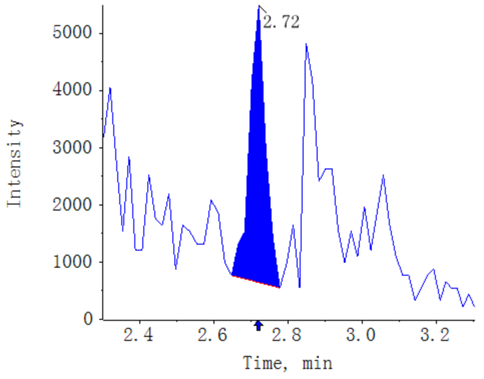

A21233263b_b
Liquiritin AREA:2.06e4 S/N:9.5

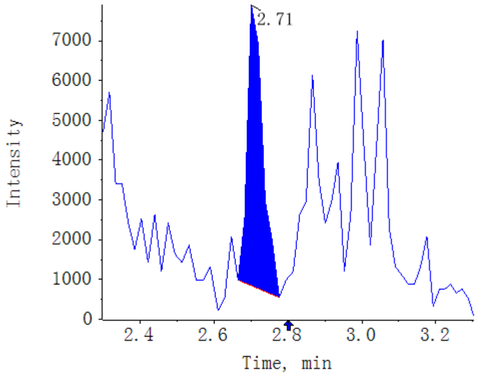

A21233264b_b
Liquiritin AREA:1.92e4 S/N:7.7

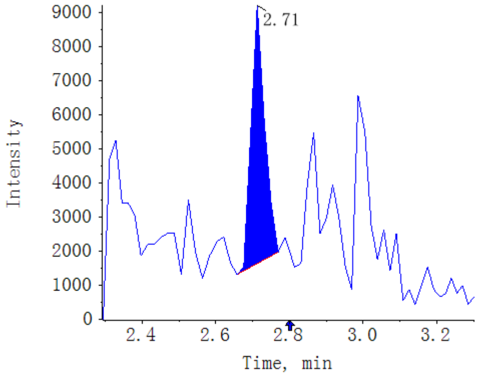

A21233265b_b
Liquiritin AREA:2.44e4 S/N:5.7

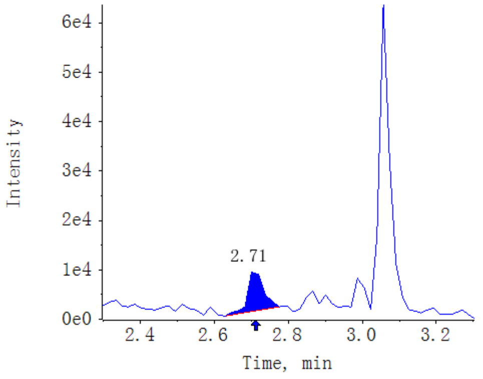

A21233266b_b
Liquiritin AREA:2.14e4 S/N:6.0

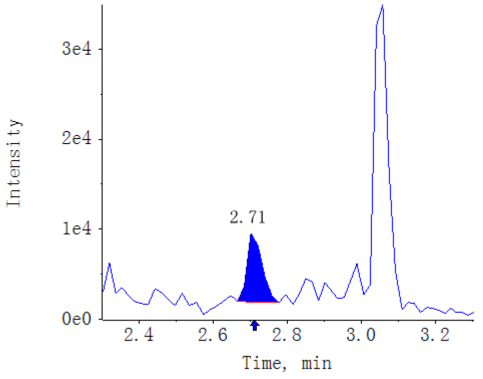

A21233267b_b
Liquiritin AREA:2.47e4 S/N:3.5

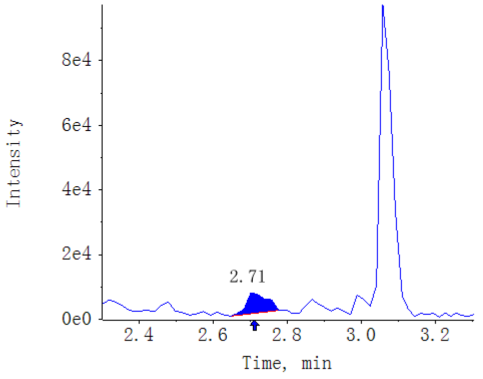

**Result Table**	MWXS-21-2101D-3_18_WH6500-5_A20-3_V1.0_TY_20211028	**Algorithm Used**	MQ4
**Acquisition Method**	Flavonoids_V1.0_WH6500-5_LT_20211025.dam	**Instrument Name**	QTRAP 6500+ Low Mass
**Project**	N/A	**Analytes QTY**	204:67

Compound name: (-)-Gallocatechin gallate (457.1 / 169.0)

Sample Name	Sample Type	Area (cps)	Is Area (cps)	RT (min)	S/N	Target Conc	Calculated Conc.()
STD_0.5nM	Standard	2.39e3	N/A	2.48	16.3	0.5000	3.442252e-1
STD_1nM	Standard	6.07e3	N/A	2.46	21.8	1.0000	1.172144e0
STD_5nM	Standard	3.20e4	N/A	2.46	75.4	5.0000	7.003963e0
STD_10nM	Standard	3.57e4	N/A	2.46	114.8	10.0000	7.841150e0
STD_20nM	Standard	8.03e4	N/A	2.46	145.9	20.0000	1.786485e1
STD_50nM	Standard	2.43e5	N/A	2.47	182.3	50.0000	5.436523e1
STD_100nM	Standard	4.32e5	N/A	2.47	198.1	100.0000	9.688181e1
STD_200nM	Standard	8.95e5	N/A	2.46	237.2	200.0000	2.010266e2
STD_500nM	Standard	N/A	N/A	N/A	N/A	500.0000	N/A
STD_1000nM	Standard	N/A	N/A	N/A	N/A	1000.0000	N/A
STD_2000nM	Standard	N/A	N/A	N/A	N/A	2000.0000	N/A
V1.0_MW_RQC1_20211018	Quality Control	N/A	N/A	N/A	N/A	0.0000	N/A
Blank	Unknown	N/A	N/A	N/A	N/A	N/A	N/A
V1.0_MWMS_20211021_1	Unknown	8.72e5	N/A	2.47	257.2	N/A	1.958754e2
MWXS212101D3_R1	Quality Control	8.72e5	N/A	2.46	242.7	0.0000	1.957943e2
MWXS212101D3_R2	Quality Control	8.67e5	N/A	2.47	206.4	0.0000	1.947021e2
MWXS212101D3_R3	Quality Control	8.78e5	N/A	2.47	227.7	0.0000	1.972904e2
A21233250b_b	Unknown	6.18e4	N/A	2.40	82.4	N/A	1.369487e1
A21233251b_b	Unknown	3.02e4	N/A	2.40	44.1	N/A	6.602019e0
A21233252b_b	Unknown	1.04e4	N/A	2.40	17.7	N/A	2.143998e0
A21233253b_b	Unknown	3.76e4	N/A	2.40	45.1	N/A	8.252535e0
A21233254b_b	Unknown	2.98e4	N/A	2.40	40.3	N/A	6.508010e0
A21233255b_b	Unknown	7.50e3	N/A	2.39	8.1	N/A	1.492935e0
A21233256b_b	Unknown	9.88e4	N/A	2.41	73.4	N/A	2.201878e1
A21233257b_b	Unknown	1.53e4	N/A	2.41	20.0	N/A	3.240187e0
A21233258b_b	Unknown	2.26e4	N/A	2.41	22.8	N/A	4.879153e0
A21233259b_b	Unknown	1.30e5	N/A	2.40	110.7	N/A	2.908768e1
A21233260b_b	Unknown	4.76e5	N/A	2.40	205.2	N/A	1.069284e2
A21233261b_b	Unknown	1.27e6	N/A	2.40	278.9	N/A	2.842444e2
A21233262b_b	Unknown	5.19e3	N/A	2.40	11.9	N/A	9.729736e-1
A21233263b_b	Unknown	4.33e4	N/A	2.40	55.5	N/A	9.541979e0
A21233264b_b	Unknown	1.44e5	N/A	2.40	84.7	N/A	3.216876e1
A21233265b_b	Unknown	1.69e4	N/A	2.41	24.3	N/A	3.603405e0
A21233266b_b	Unknown	3.11e4	N/A	2.40	39.1	N/A	6.800104e0
A21233267b_b	Unknown	1.98e4	N/A	2.40	26.2	N/A	4.248885e0

Compound name: (-)-Gallocatechin gallate
Regression Equation: y = 4447.50939 x + 860.04244 (r = 0.99721) (weighting: 1 / x)

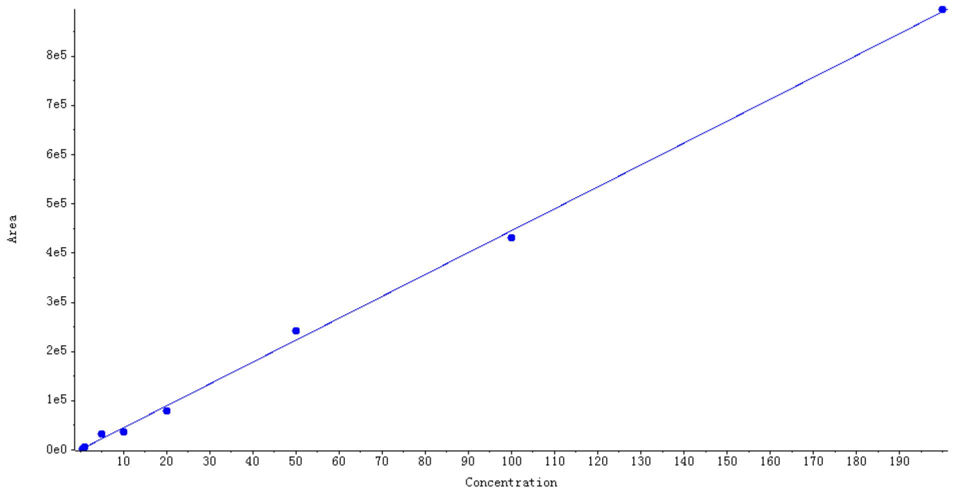

Peak Review

Blank
(-)-Gallocatechin gallate AREA:N/A
S/N:N/A

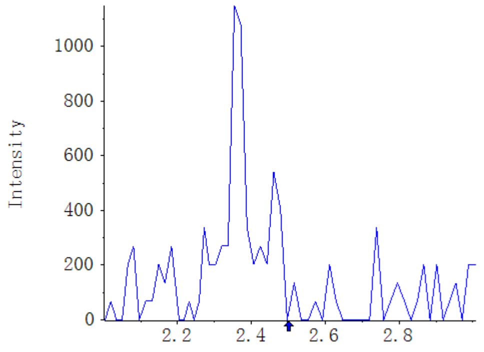

V1.0_MWMS_20211021_1
(-)-Gallocatechin gallate
AREA:8.72e5 S/N:257.2

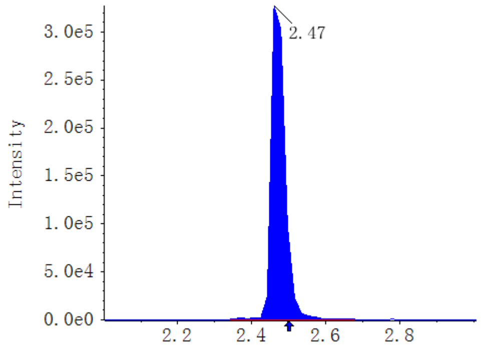

A21233250b_b
(-)-Gallocatechin gallate
AREA:6.18e4 S/N:82.4

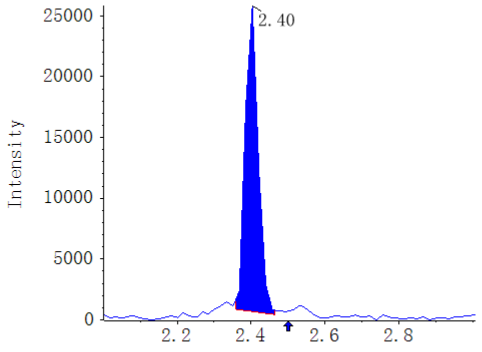

A21233251b_b
(-)-Gallocatechin gallate
AREA:3.02e4 S/N:44.1

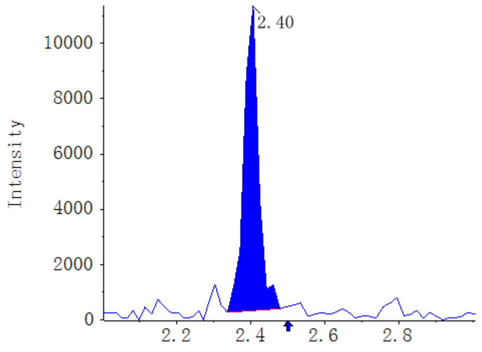

A21233252b_b
(-)-Gallocatechin gallate
AREA:1.04e4 S/N:17.7

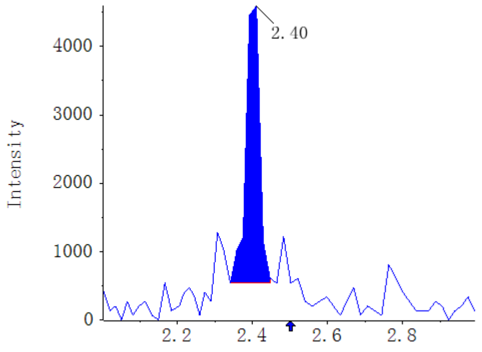

A21233253b_b
(-)-Gallocatechin gallate
AREA:3.76e4 S/N:45.1

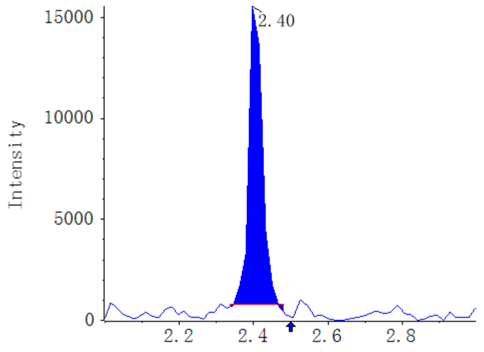

A21233254b_b
(-)-Gallocatechin gallate
AREA:2.98e4 S/N:40.3

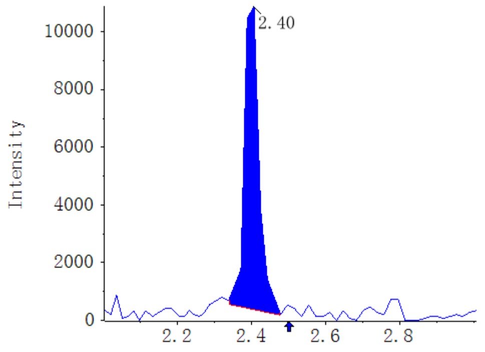

A21233255b_b
(-)-Gallocatechin gallate
AREA:7.50e3 S/N:8.1

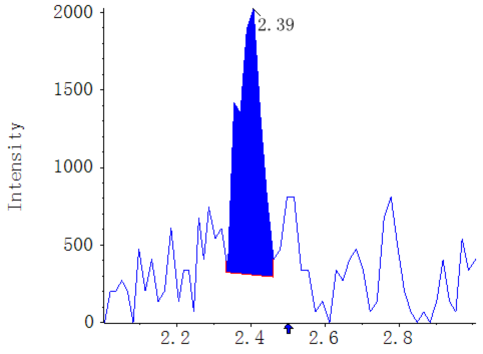

A21233256b_b
(-)-Gallocatechin gallate
AREA:9.88e4 S/N:73.4

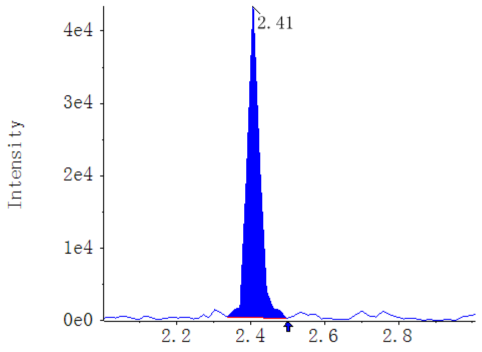

A21233257b_b
(-)-Gallocatechin gallate
AREA:1.53e4 S/N:20.0

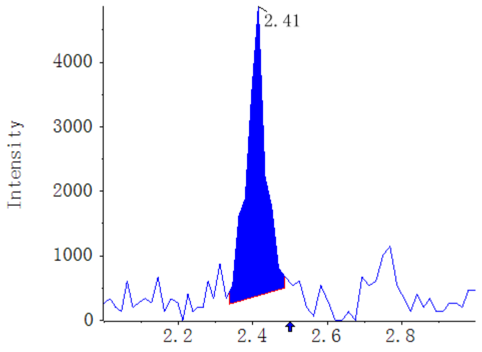

A21233258b_b
(-)-Gallocatechin gallate
AREA:2.26e4 S/N:22.8

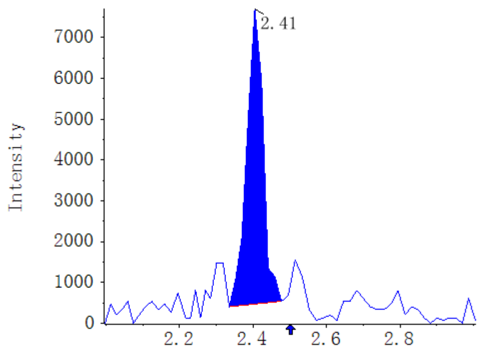

A21233259b_b
(-)-Gallocatechin gallate
AREA:1.30e5 S/N:110.7

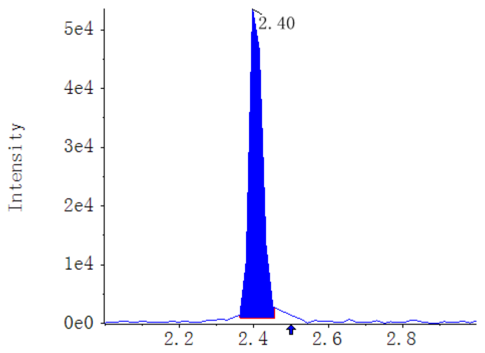

A21233260b_b
(-)-Gallocatechin gallate
AREA:4.76e5 S/N:205.2

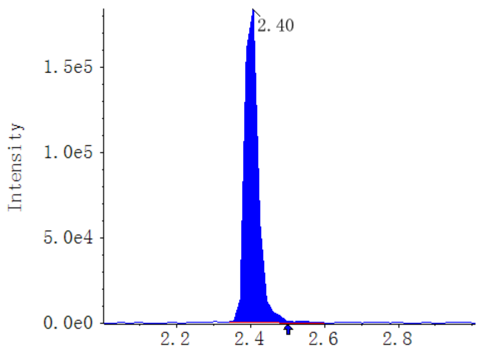

A21233261b_b
(-)-Gallocatechin gallate
AREA:1.27e6 S/N:278.9

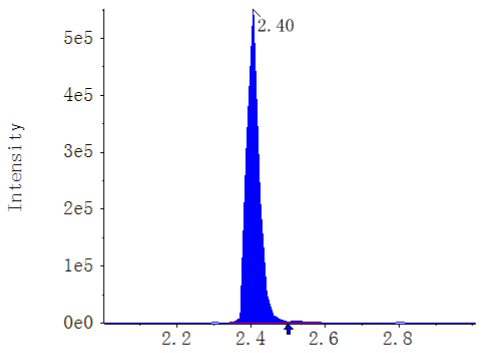

A21233262b_b
(-)-Gallocatechin gallate
AREA:5.19e3 S/N:11.9

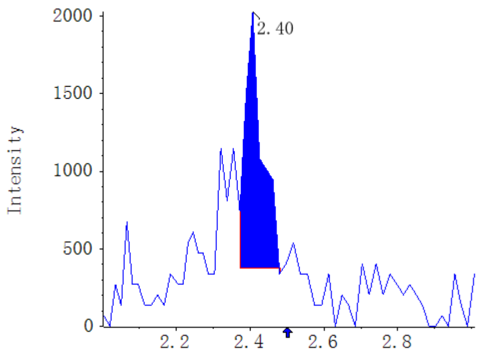

A21233263b_b
(-)-Gallocatechin gallate
AREA:4.33e4 S/N:55.5

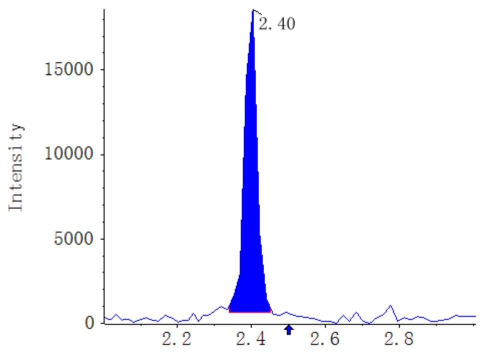

A21233264b_b
(-)-Gallocatechin gallate
AREA:1.44e5 S/N:84.7

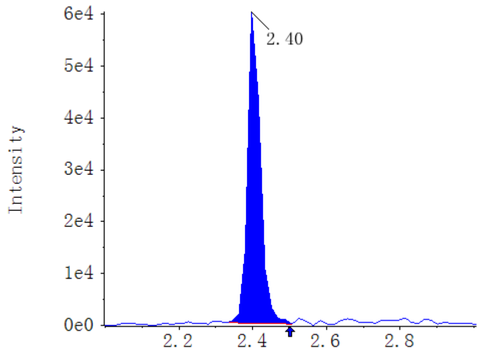

A21233265b_b
(-)-Gallocatechin gallate
AREA:1.69e4 S/N:24.3

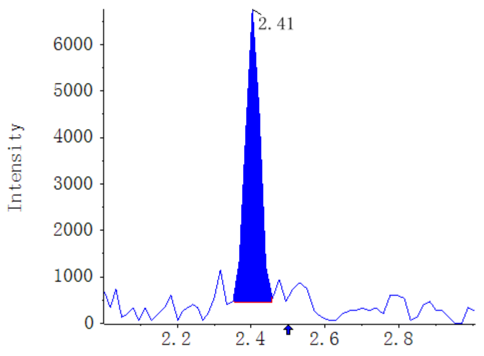

A21233266b_b
(-)-Gallocatechin gallate
AREA:3.11e4 S/N:39.1

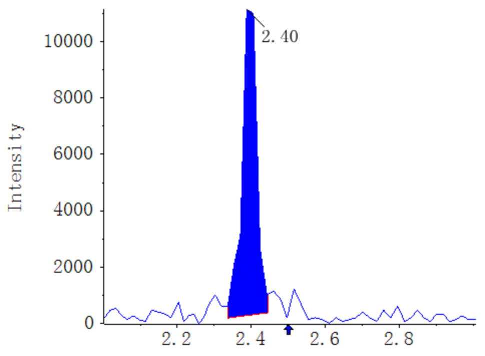

A21233267b_b
(-)-Gallocatechin gallate
AREA:1.98e4 S/N:26.2

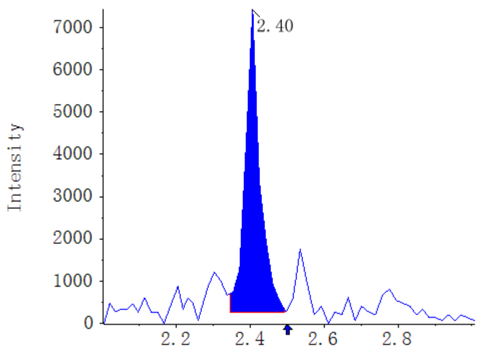

**Result Table**	MWXS-21-2101D-3_18_WH6500-5_A20-3_V1.0_TY_20211028	**Algorithm Used**	MQ4
**Acquisition Method**	Flavonoids_V1.0_WH6500-5_LT_20211025.dam	**Instrument Name**	QTRAP 6500+ Low Mass
**Project**	N/A	**Analytes QTY**	204:72

Compound name: Cynaroside (447.1 / 285.0)

Sample Name	Sample Type	Area (cps)	Is Area (cps)	RT (min)	S/N	Target Conc	Calculated Conc.()
STD_0.5nM	Standard	5.42e3	N/A	2.78	15.4	0.5000	3.679276e-1
STD_1nM	Standard	9.51e3	N/A	2.76	31.1	1.0000	8.137083e-1
STD_5nM	Standard	6.75e4	N/A	2.77	40.5	5.0000	7.143554e0
STD_10nM	Standard	9.83e4	N/A	2.77	40.6	10.0000	1.049888e1
STD_20nM	Standard	1.81e5	N/A	2.77	55.9	20.0000	1.952823e1
STD_50nM	Standard	4.59e5	N/A	2.78	50.8	50.0000	4.991449e1
STD_100nM	Standard	9.29e5	N/A	2.78	41.4	100.0000	1.011942e2
STD_200nM	Standard	1.81e6	N/A	2.77	57.7	200.0000	1.970390e2
STD_500nM	Standard	N/A	N/A	N/A	N/A	500.0000	N/A
STD_1000nM	Standard	N/A	N/A	N/A	N/A	1000.0000	N/A
STD_2000nM	Standard	N/A	N/A	N/A	N/A	2000.0000	N/A
V1.0_MW_RQC1_20211018	Quality Control	9.96e7	N/A	2.76	52.7	0.0000	1.087153e4
Blank	Unknown	N/A	N/A	N/A	N/A	N/A	N/A
V1.0_MWMS_20211021_1	Unknown	1.51e6	N/A	2.78	58.0	N/A	1.647122e2
MWXS212101D3_R1	Quality Control	1.53e6	N/A	2.77	55.0	0.0000	1.668221e2
MWXS212101D3_R2	Quality Control	1.54e6	N/A	2.78	63.6	0.0000	1.676668e2
MWXS212101D3_R3	Quality Control	1.54e6	N/A	2.78	60.9	0.0000	1.678479e2
A21233250b_b	Unknown	1.26e5	N/A	2.76	10.7	N/A	1.352708e1
A21233251b_b	Unknown	N/A	N/A	N/A	N/A	N/A	N/A
A21233252b_b	Unknown	N/A	N/A	N/A	N/A	N/A	N/A
A21233253b_b	Unknown	N/A	N/A	N/A	N/A	N/A	N/A
A21233254b_b	Unknown	N/A	N/A	N/A	N/A	N/A	N/A
A21233255b_b	Unknown	N/A	N/A	N/A	N/A	N/A	N/A
A21233256b_b	Unknown	N/A	N/A	N/A	N/A	N/A	N/A
A21233257b_b	Unknown	N/A	N/A	N/A	N/A	N/A	N/A
A21233258b_b	Unknown	N/A	N/A	N/A	N/A	N/A	N/A
A21233259b_b	Unknown	1.52e5	N/A	2.75	16.8	N/A	1.631934e1
A21233260b_b	Unknown	1.10e5	N/A	2.76	9.6	N/A	1.173353e1
A21233261b_b	Unknown	1.42e5	N/A	2.76	13.7	N/A	1.528784e1
A21233262b_b	Unknown	7.32e4	N/A	2.75	8.9	N/A	7.759179e0
A21233263b_b	Unknown	7.49e4	N/A	2.75	4.2	N/A	7.951200e0
A21233264b_b	Unknown	N/A	N/A	N/A	N/A	N/A	N/A
A21233265b_b	Unknown	N/A	N/A	N/A	N/A	N/A	N/A
A21233266b_b	Unknown	N/A	N/A	N/A	N/A	N/A	N/A
A21233267b_b	Unknown	N/A	N/A	N/A	N/A	N/A	N/A

Compound name: Cynaroside
Regression Equation: y = 9163.75687 x + 2049.74881 (r = 0.99853) (weighting: 1 / x)

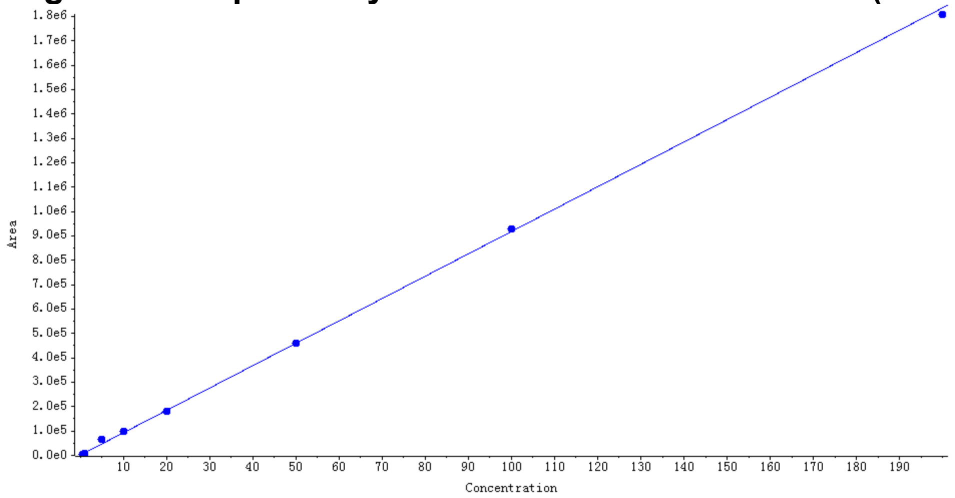

Peak Review

Blank
Cynaroside AREA:N/A S/N:N/A

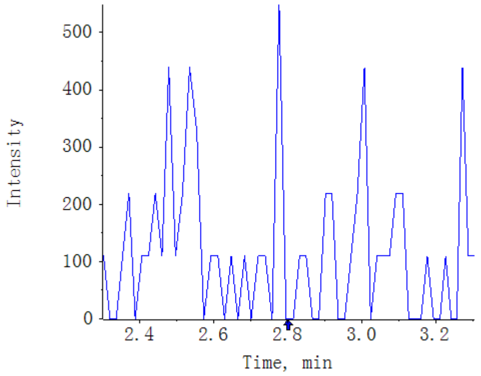

V1.0_MWMS_20211021_1
Cynaroside AREA:1.51e6
S/N:58.0

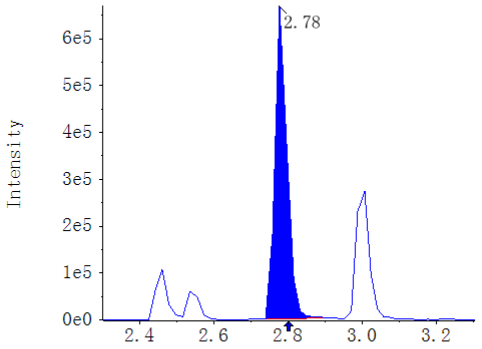

A21233250b_b
Cynaroside AREA:1.26e5
S/N:10.7

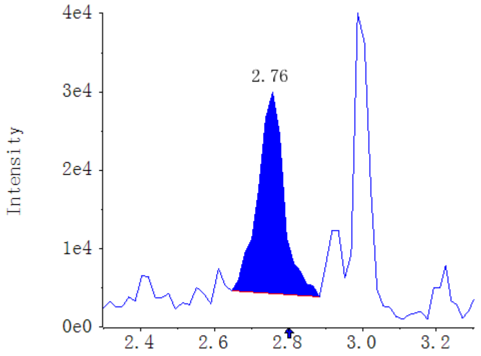

A21233251b_b
Cynaroside AREA:N/A S/N:N/A

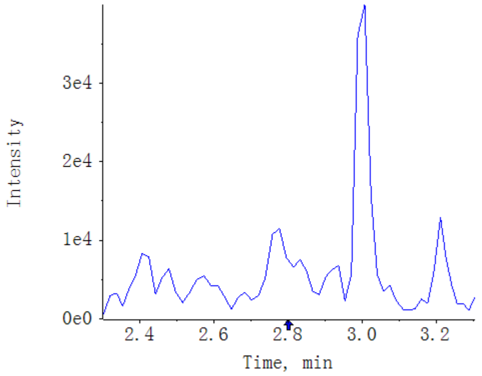

A21233252b_b
Cynaroside AREA:N/A S/N:N/A

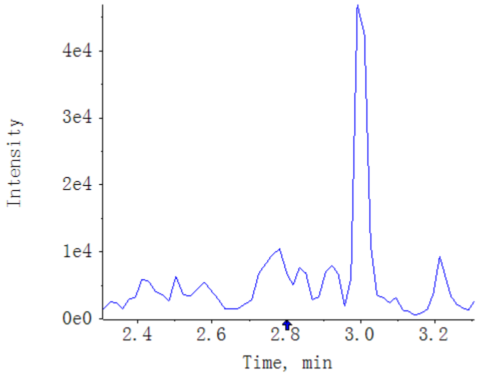

A21233253b_b
Cynaroside AREA:N/A S/N:N/A

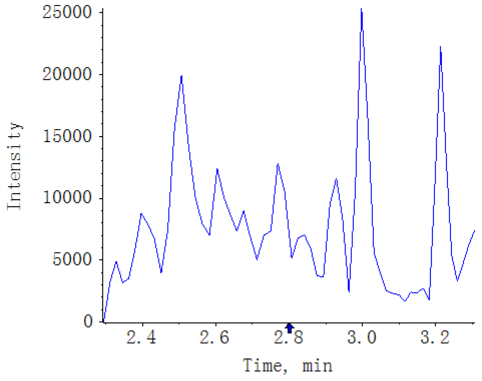

A21233254b_b
Cynaroside AREA:N/A S/N:N/A

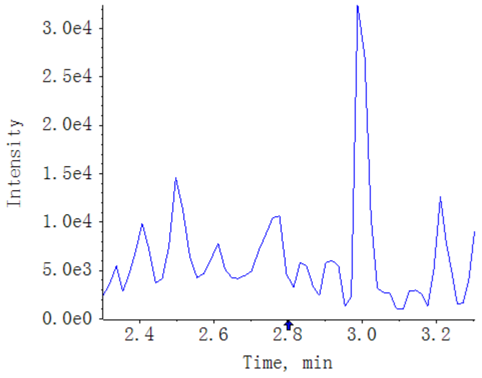

A21233255b_b
Cynaroside AREA:N/A S/N:N/A

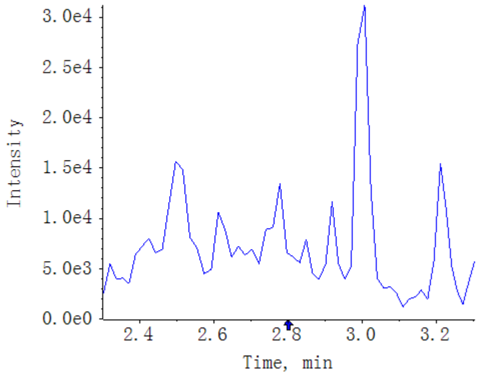

A21233256b_b
Cynaroside AREA:N/A S/N:N/A

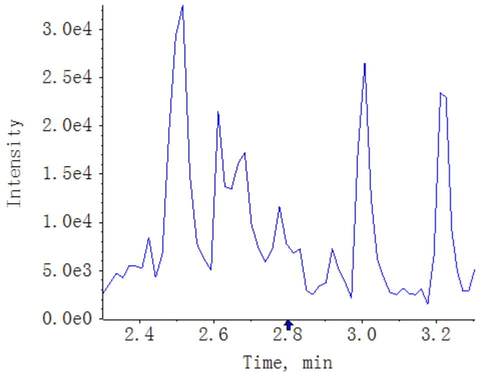

A21233257b_b
Cynaroside AREA:N/A S/N:N/A

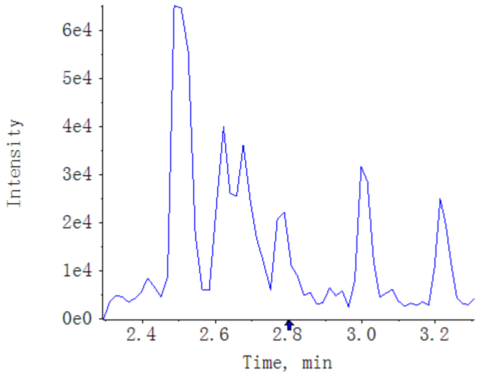

A21233258b_b
Cynaroside AREA:N/A S/N:N/A

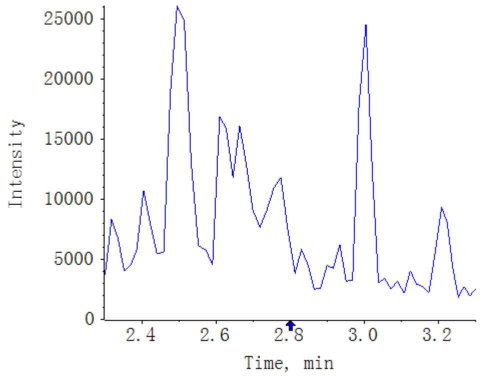

A21233259b_b
Cynaroside AREA:1.52e5 S/N:16.8

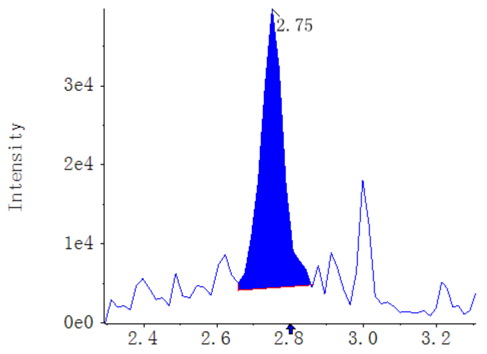

A21233260b_b
Cynaroside AREA:1.10e5 S/N:9.6

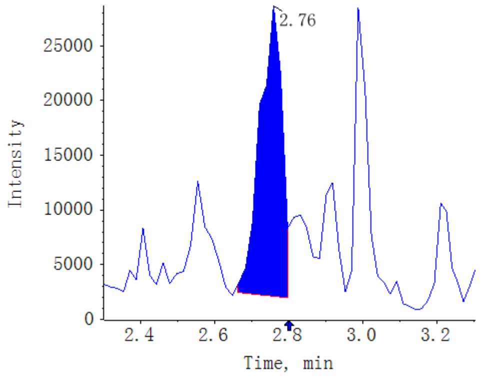

A21233261b_b
Cynaroside AREA:1.42e5 S/N:13.7

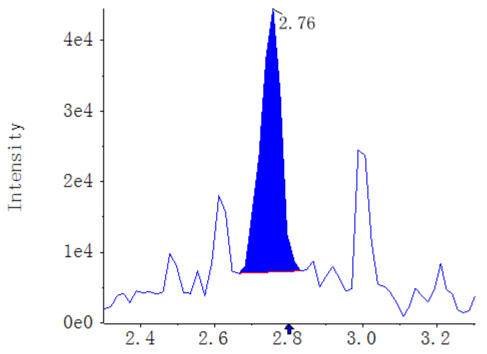

A21233262b_b
Cynaroside AREA:7.32e4 S/N:8.9

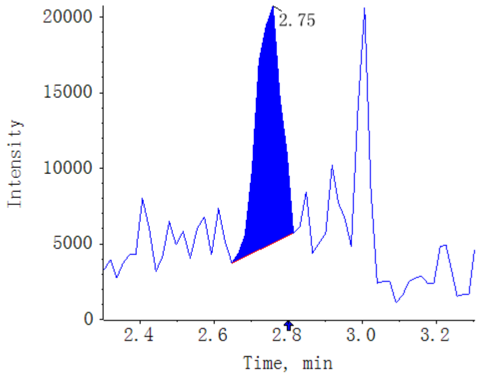

A21233263b_b
Cynaroside AREA:7.49e4 S/N:4.2

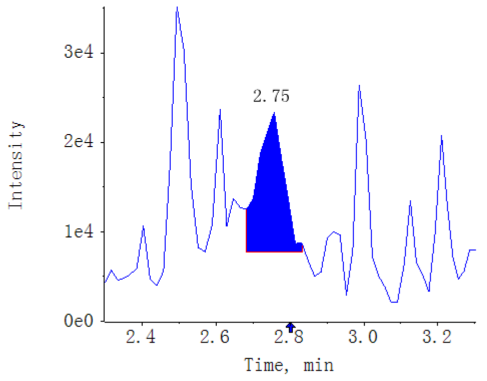

A21233264b_b
Cynaroside AREA:N/A S/N:N/A

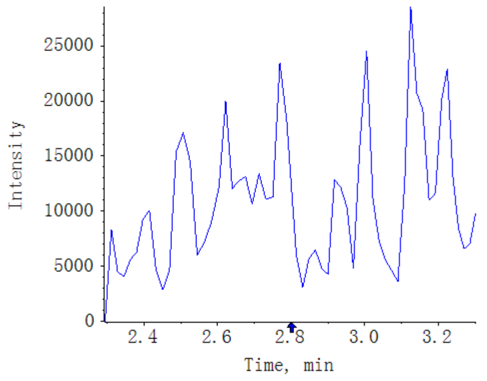

A21233265b_b
Cynaroside AREA:N/A S/N:N/A

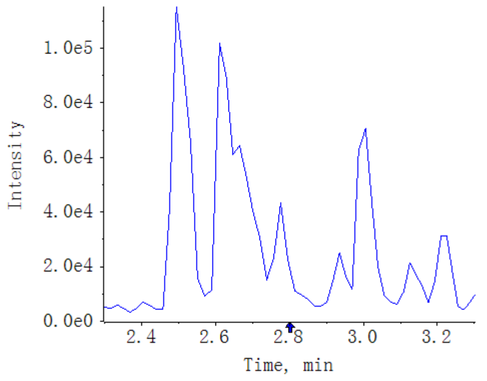

A21233266b_b
Cynaroside AREA:N/A S/N:N/A

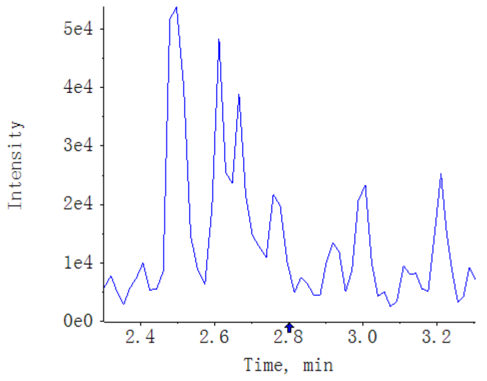

A21233267b_b
Cynaroside AREA:N/A S/N:N/A

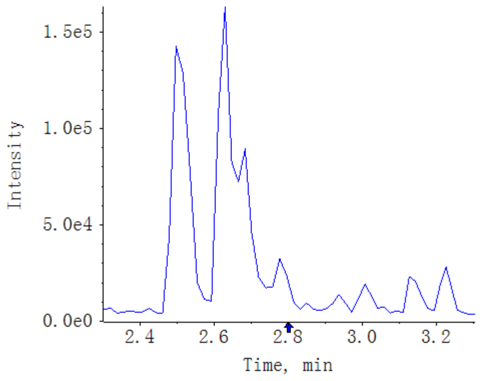

Result Table	MWXS-21-2101D-3_18_WH6500-5_A20-3_V1.0_TY_20211028	Algorithm Used	MQ4
Acquisition Method	Flavonoids_V1.0_WH6500-5_LT_20211025.dam	Instrument Name	QTRAP 6500+ Low Mass
Project	N/A	Analytes QTY	204:77

Compound name: Calycosin-7-O-β-D-glucoside (491.1 / 283.1)

Sample Name	Sample Type	Area (cps)	Is Area (cps)	RT (min)	S/N	Target Conc	Calculated Conc.()
STD_0.5nM	Standard	1.97e3	N/A	2.73	7.6	0.5000	3.033290e-1
STD_1nM	Standard	4.18e3	N/A	2.71	12.7	1.0000	8.117155e-1
STD_5nM	Standard	3.28e4	N/A	2.71	34.3	5.0000	7.402803e0
STD_10nM	Standard	4.23e4	N/A	2.72	75.3	10.0000	9.596905e0
STD_20nM	Standard	9.55e4	N/A	2.71	120.3	20.0000	2.187440e1
STD_50nM	Standard	2.47e5	N/A	2.72	116.0	50.0000	5.681336e1
STD_100nM	Standard	4.02e5	N/A	2.72	155.7	100.0000	9.258016e1
STD_200nM	Standard	8.56e5	N/A	2.71	164.3	200.0000	1.971173e2
STD_500nM	Standard	N/A	N/A	N/A	N/A	500.0000	N/A
STD_1000nM	Standard	N/A	N/A	N/A	N/A	1000.0000	N/A
STD_2000nM	Standard	N/A	N/A	N/A	N/A	2000.0000	N/A
V1.0_MW_RQC1_20211018	Quality Control	7.02e3	N/A	2.77	4.0	0.0000	1.467161e0
Blank	Unknown	N/A	N/A	N/A	N/A	N/A	N/A
V1.0_MWMS_20211021_1	Unknown	6.75e5	N/A	2.72	127.2	N/A	1.555360e2
MWXS212101D3_R1	Quality Control	6.46e5	N/A	2.72	180.5	0.0000	1.487279e2
MWXS212101D3_R2	Quality Control	6.94e5	N/A	2.72	145.8	0.0000	1.599095e2
MWXS212101D3_R3	Quality Control	6.42e5	N/A	2.72	155.9	0.0000	1.479109e2
A21233250b_b	Unknown	N/A	N/A	N/A	N/A	N/A	N/A
A21233251b_b	Unknown	N/A	N/A	N/A	N/A	N/A	N/A
A21233252b_b	Unknown	N/A	N/A	N/A	N/A	N/A	N/A
A21233253b_b	Unknown	5.03e3	N/A	2.73	4.5	N/A	1.008405e0
A21233254b_b	Unknown	N/A	N/A	N/A	N/A	N/A	N/A
A21233255b_b	Unknown	6.02e3	N/A	2.73	4.9	N/A	1.236133e0
A21233256b_b	Unknown	N/A	N/A	N/A	N/A	N/A	N/A
A21233257b_b	Unknown	N/A	N/A	N/A	N/A	N/A	N/A
A21233258b_b	Unknown	N/A	N/A	N/A	N/A	N/A	N/A
A21233259b_b	Unknown	1.39e4	N/A	2.73	11.0	N/A	3.046213e0
A21233260b_b	Unknown	8.00e3	N/A	2.72	8.7	N/A	1.693446e0
A21233261b_b	Unknown	5.51e3	N/A	2.72	2.8	N/A	1.118137e0
A21233262b_b	Unknown	1.33e4	N/A	2.73	5.4	N/A	2.923231e0
A21233263b_b	Unknown	1.40e4	N/A	2.72	6.5	N/A	3.066686e0
A21233264b_b	Unknown	N/A	N/A	N/A	N/A	N/A	N/A
A21233265b_b	Unknown	1.29e4	N/A	2.73	2.7	N/A	2.820635e0
A21233266b_b	Unknown	1.20e4	N/A	2.73	4.2	N/A	2.616339e0
A21233267b_b	Unknown	1.26e4	N/A	2.75	3.0	N/A	2.744846e0

Compound name: Calycosin-7-O-β-D-glucoside
Regression Equation: y = 4337.23759 x + 657.66151 (r = 0.99597) (weighting: 1 / x)

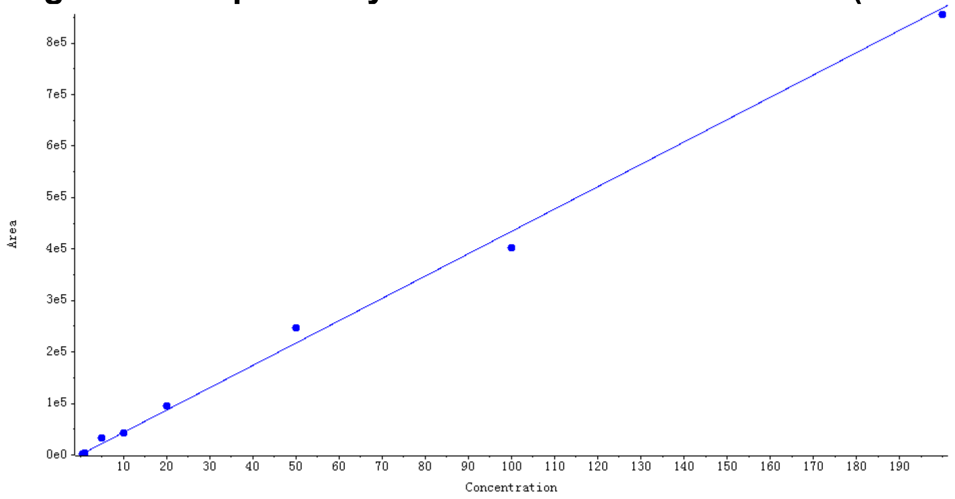

Peak Review

Blank
Calycosin-7-O- β -D-glucoside
AREA:N/A S/N:N/A

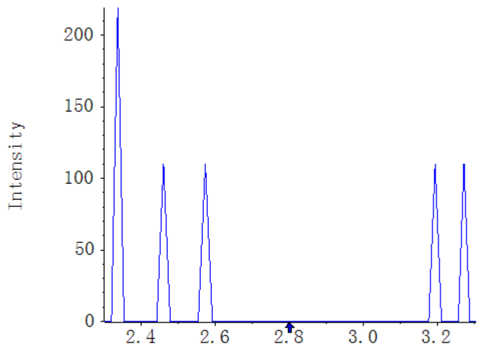

V1.0_MWMS_20211021_1
Calycosin-7-O- β -D-glucoside
AREA:6.75e5 S/N:127.2

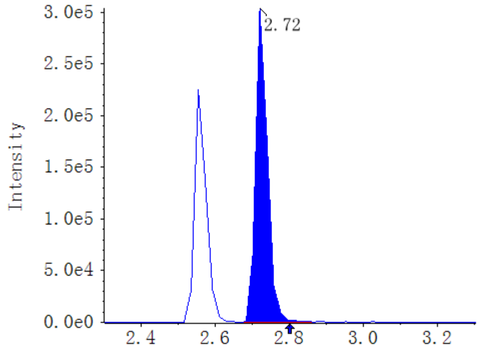

A21233250b_b
Calycosin-7-O- β -D-glucoside
AREA:N/A S/N:N/A

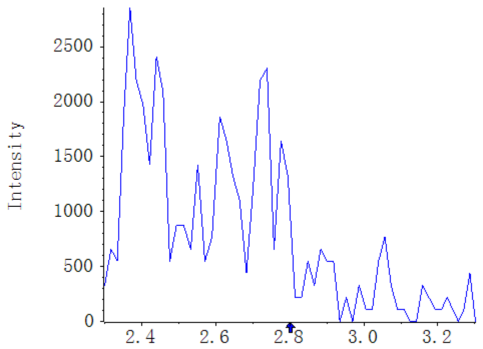

A21233251b_b
Calycosin-7-O- β -D-glucoside
AREA:N/A S/N:N/A

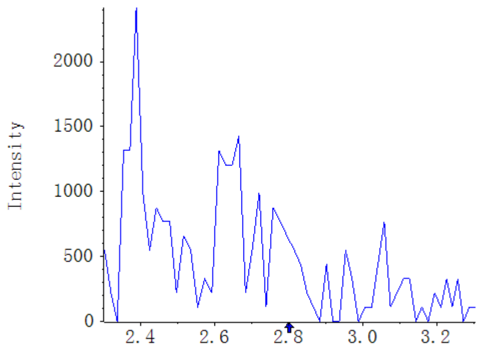

A21233252b_b
Calycosin-7-O- β -D-glucoside
AREA:N/A S/N:N/A

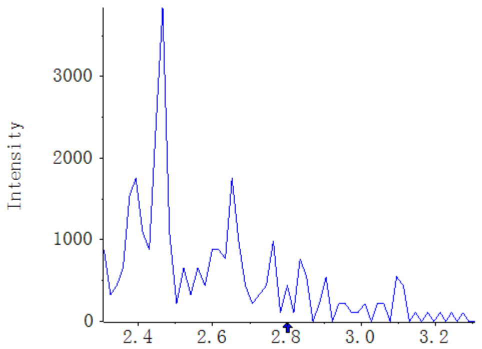

A21233253b_b
Calycosin-7-O- β -D-glucoside
AREA:5.03e3 S/N:4.5

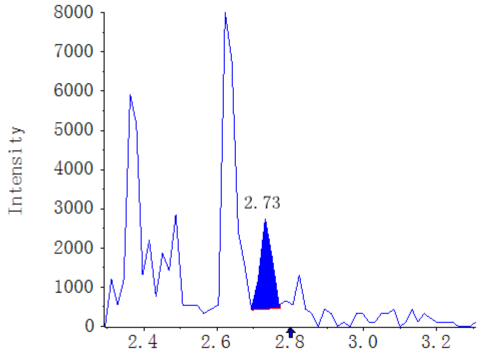

A21233254b_b
Calycosin-7-O- β -D-glucoside
AREA:N/A S/N:N/A

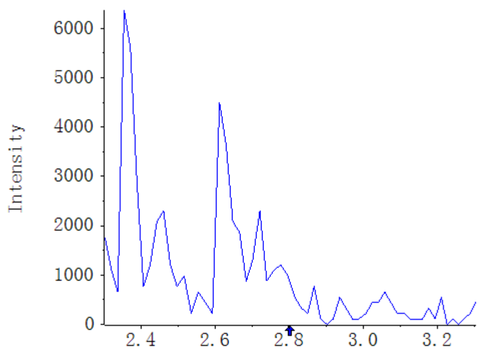

A21233255b_b
Calycosin-7-O- β -D-glucoside
AREA:6.02e3 S/N:4.9

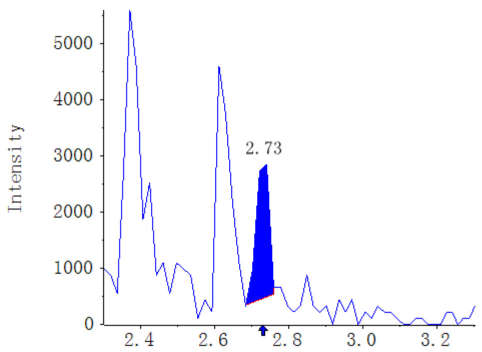

A21233256b_b
Calycosin-7-O- β -D-glucoside
AREA:N/A S/N:N/A

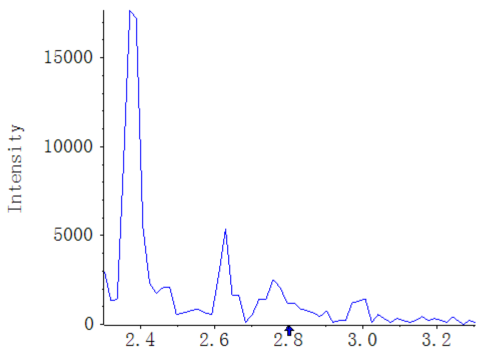

A21233257b_b
Calycosin-7-O- β -D-glucoside
AREA:N/A S/N:N/A

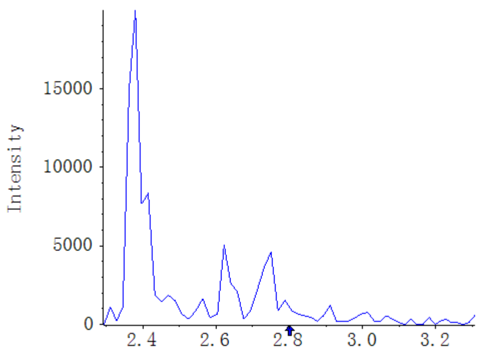

A21233258b_b
Calycosin-7-O- β -D-glucoside
AREA:N/A S/N:N/A

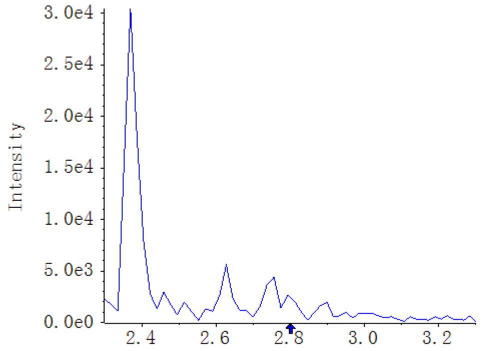

A21233259b_b
Calycosin-7-O- β -D-glucoside
AREA:1.39e4 S/N:11.0

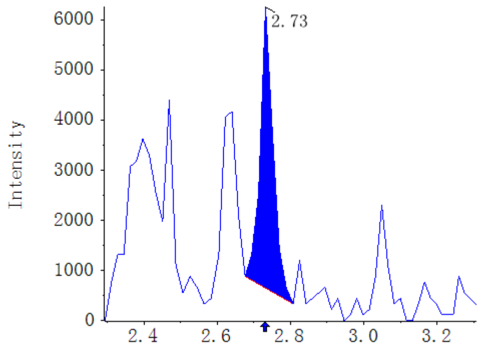

A21233260b_b
Calycosin-7-O- β -D-glucoside
AREA:8.00e3 S/N:8.7

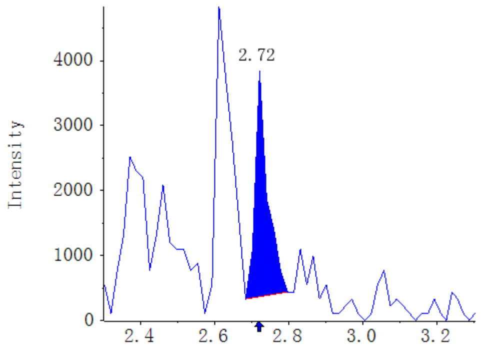

A21233261b_b
Calycosin-7-O- β -D-glucoside
AREA:5.51e3 S/N:2.8

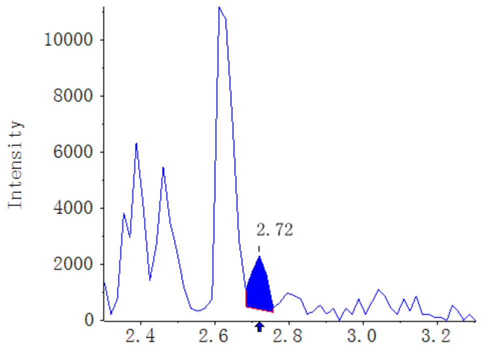

A21233262b_b
Calycosin-7-O- β -D-glucoside
AREA:1.33e4 S/N:5.4

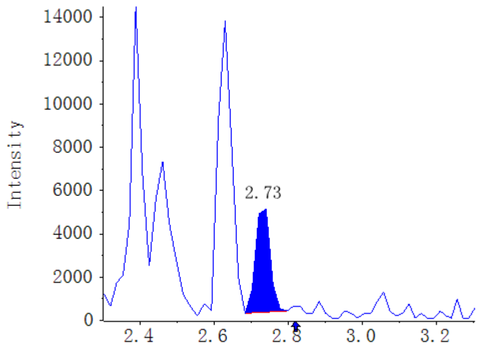

A21233263b_b
Calycosin-7-O- β -D-glucoside
AREA:1.40e4 S/N:6.5

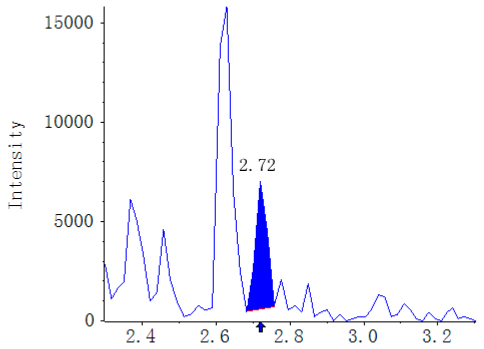

A21233264b_b
Calycosin-7-O- β -D-glucoside
AREA:N/A S/N:N/A

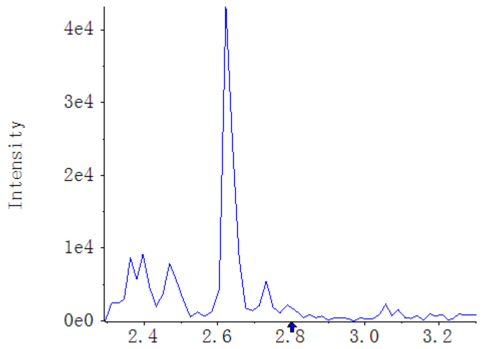

A21233265b_b
Calycosin-7-O- β -D-glucoside
AREA:1.29e4 S/N:2.7

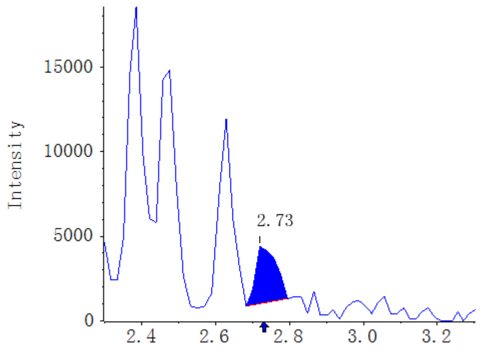

A21233266b_b
Calycosin-7-O- β -D-glucoside
AREA:1.20e4 S/N:4.2

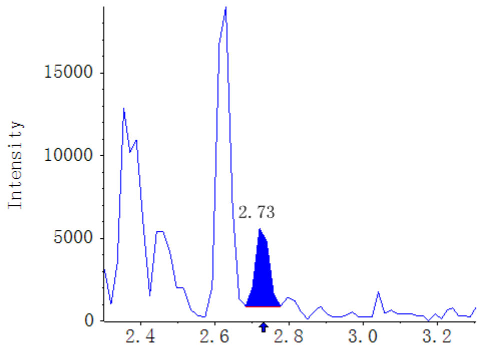

A21233267b_b
Calycosin-7-O- β -D-glucoside
AREA:1.26e4 S/N:3.0

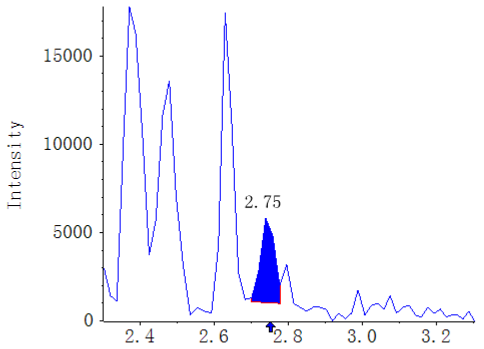

Result Table	MWXS-21-2101D-3_18_WH6500-5_A20-3_V1.0_TY_20211028	Algorithm Used	MQ4
Acquisition Method	Flavonoids_V1.0_WH6500-5_LT_20211025.dam	Instrument Name	QTRAP 6500+ Low Mass
Project	N/A	Analytes QTY	204:84

Compound name: Typhaneoside (769.2 / 314.0)

Sample Name	Sample Type	Area (cps)	Is Area (cps)	RT (min)	S/N	Target Conc	Calculated Conc.()
STD_0.5nM	Standard	1.27e3	N/A	2.52	19.3	0.5000	3.953665e-1
STD_1nM	Standard	2.34e3	N/A	2.52	31.2	1.0000	8.135263e-1
STD_5nM	Standard	1.84e4	N/A	2.52	45.2	5.0000	7.098839e0
STD_10nM	Standard	2.66e4	N/A	2.52	39.0	10.0000	1.030599e1
STD_20nM	Standard	4.88e4	N/A	2.52	35.0	20.0000	1.897026e1
STD_50nM	Standard	1.27e5	N/A	2.52	153.7	50.0000	4.937076e1
STD_100nM	Standard	2.62e5	N/A	2.53	287.6	100.0000	1.023440e2
STD_200nM	Standard	5.05e5	N/A	2.52	471.6	200.0000	1.972013e2
STD_500nM	Standard	N/A	N/A	N/A	N/A	500.0000	N/A
STD_1000nM	Standard	N/A	N/A	N/A	N/A	1000.0000	N/A
STD_2000nM	Standard	N/A	N/A	N/A	N/A	2000.0000	N/A
V1.0_MW_RQC1_20211018	Quality Control	N/A	N/A	N/A	N/A	0.0000	N/A
Blank	Unknown	N/A	N/A	N/A	N/A	N/A	N/A
V1.0_MWMS_20211021_1	Unknown	4.58e5	N/A	2.53	513.2	N/A	1.785616e2
MWXS212101D3_R1	Quality Control	4.77e5	N/A	2.53	276.9	0.0000	1.860908e2
MWXS212101D3_R2	Quality Control	4.44e5	N/A	2.53	416.2	0.0000	1.732765e2
MWXS212101D3_R3	Quality Control	4.75e5	N/A	2.53	337.2	0.0000	1.854157e2
A21233250b_b	Unknown	N/A	N/A	N/A	N/A	N/A	N/A
A21233251b_b	Unknown	3.91e3	N/A	2.50	27.2	N/A	1.428591e0
A21233252b_b	Unknown	1.45e3	N/A	2.50	9.0	N/A	4.657044e-1
A21233253b_b	Unknown	1.36e4	N/A	2.50	33.8	N/A	5.219735e0
A21233254b_b	Unknown	7.29e3	N/A	2.51	25.7	N/A	2.744944e0
A21233255b_b	Unknown	1.68e4	N/A	2.51	27.8	N/A	6.443453e0
A21233256b_b	Unknown	5.41e3	N/A	2.50	19.5	N/A	2.013626e0
A21233257b_b	Unknown	N/A	N/A	N/A	N/A	N/A	N/A
A21233258b_b	Unknown	7.67e3	N/A	2.51	23.1	N/A	2.894047e0
A21233259b_b	Unknown	N/A	N/A	N/A	N/A	N/A	N/A
A21233260b_b	Unknown	7.11e3	N/A	2.50	26.2	N/A	2.677355e0
A21233261b_b	Unknown	N/A	N/A	N/A	N/A	N/A	N/A
A21233262b_b	Unknown	N/A	N/A	N/A	N/A	N/A	N/A
A21233263b_b	Unknown	1.61e4	N/A	2.50	23.0	N/A	6.191469e0
A21233264b_b	Unknown	N/A	N/A	N/A	N/A	N/A	N/A
A21233265b_b	Unknown	3.11e3	N/A	2.51	8.4	N/A	1.115762e0
A21233266b_b	Unknown	2.56e3	N/A	2.49	7.2	N/A	8.978757e-1
A21233267b_b	Unknown	1.10e4	N/A	2.50	22.5	N/A	4.208072e0

Compound name: Typhaneoside

Regression Equation: y = 2560.71134 x + 256.37551 (r = 0.99850) (weighting: 1 / x)

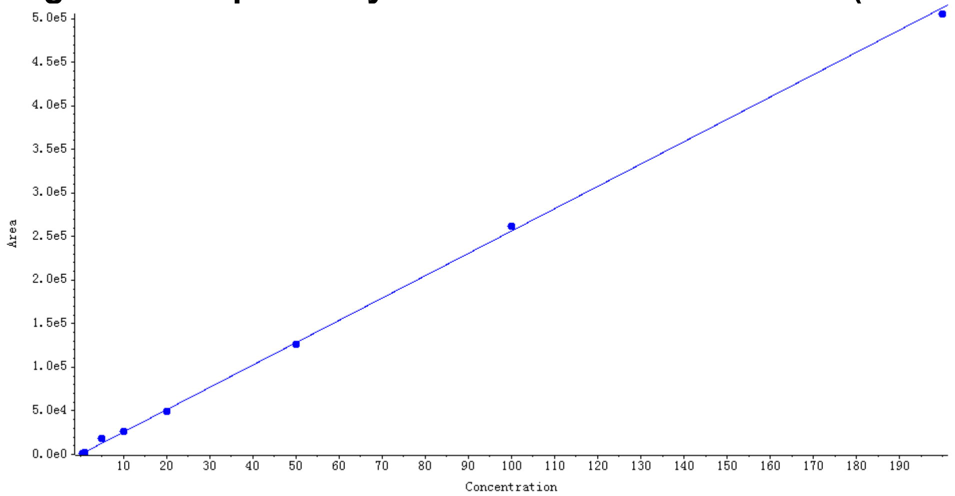

Peak Review

Blank
Typhaneoside AREA:N/A S/N:N/A

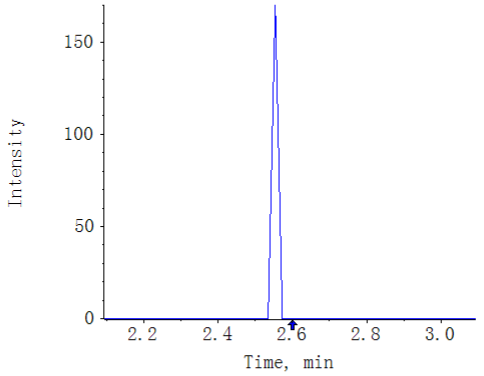

V1.0_MWMS_20211021_1
Typhaneoside AREA:4.58e5
S/N:513.2

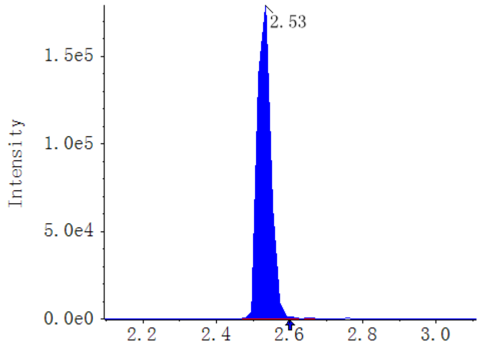

A21233250b_b
Typhaneoside AREA:N/A S/N:N/A

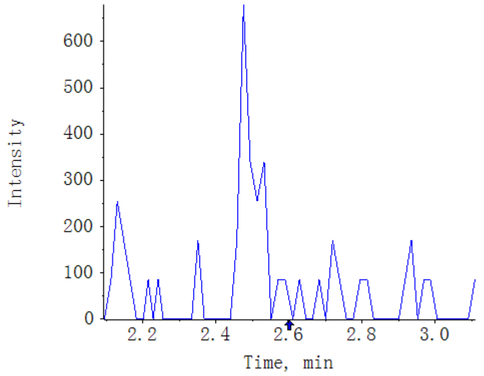

A21233251b_b
Typhaneoside AREA:3.91e3
S/N:27.2

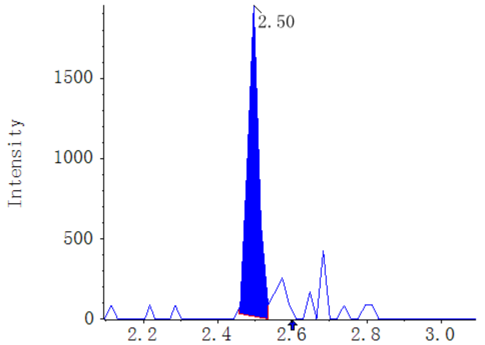

A21233252b_b
Typhaneoside AREA:1.45e3
S/N:9.0

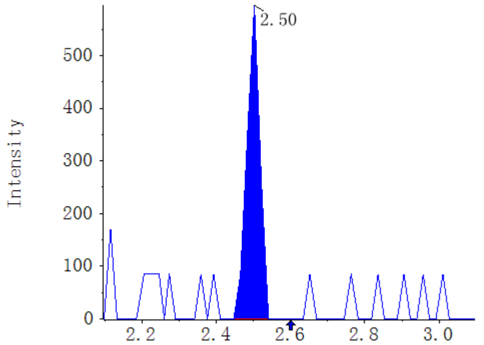

A21233253b_b
Typhaneoside AREA:1.36e4
S/N:33.8

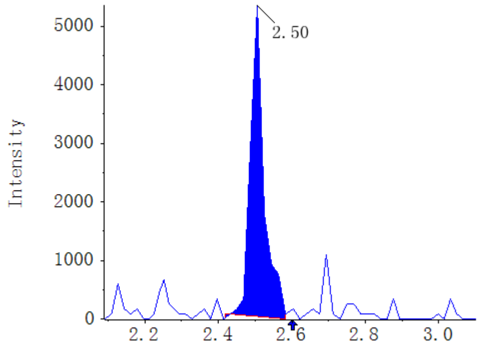

A21233254b_b
Typhaneoside AREA:7.29e3
S/N:25.7

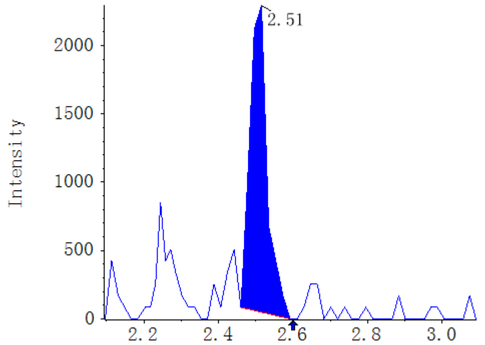

A21233255b_b
Typhaneoside AREA:1.68e4
S/N:27.8

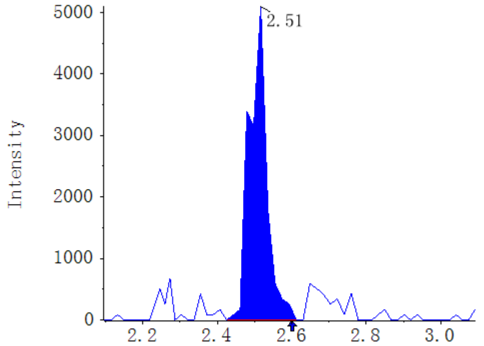

A21233256b_b
Typhaneoside AREA:5.41e3
S/N:19.5

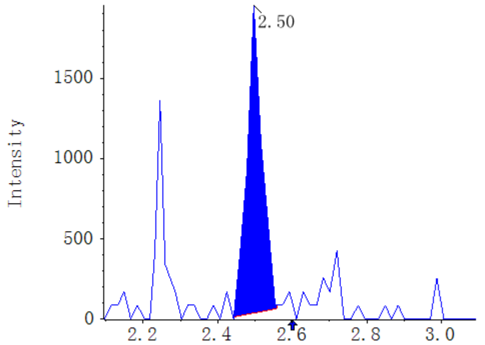

A21233257b_b
Typhaneoside AREA:N/A S/N:N/A

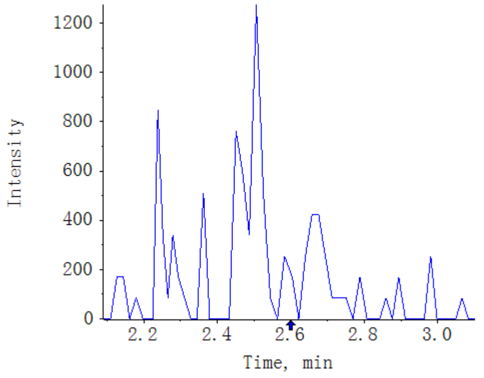

A21233258b_b
Typhaneoside AREA:7.67e3 S/N:23.1

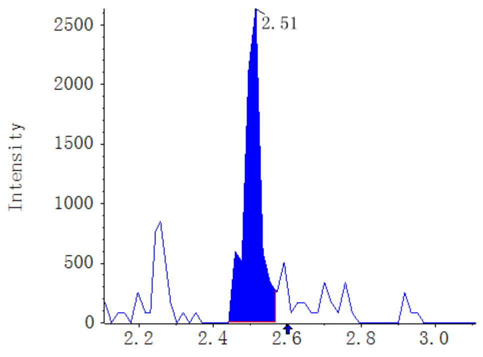

A21233259b_b
Typhaneoside AREA:N/A S/N:N/A

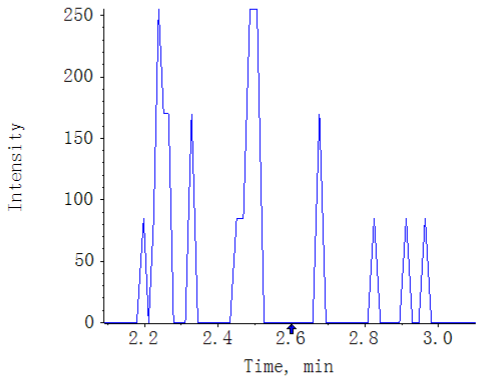

A21233260b_b
Typhaneoside AREA:7.11e3 S/N:26.2

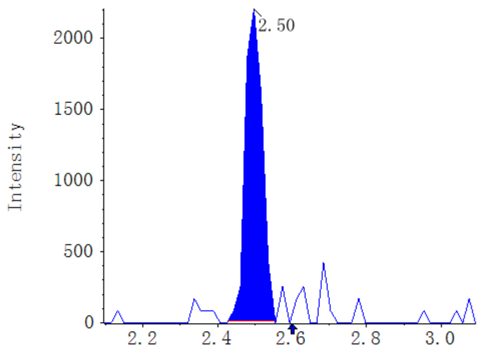

A21233261b_b
Typhaneoside AREA:N/A S/N:N/A

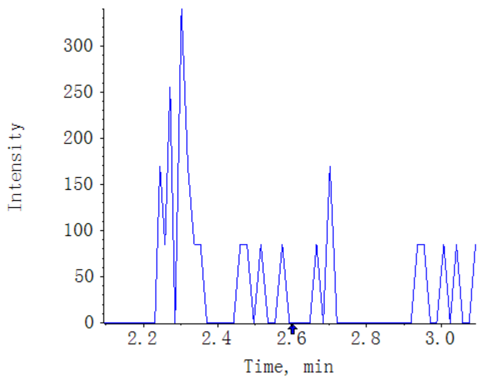

A21233262b_b
Typhaneoside AREA:N/A S/N:N/A

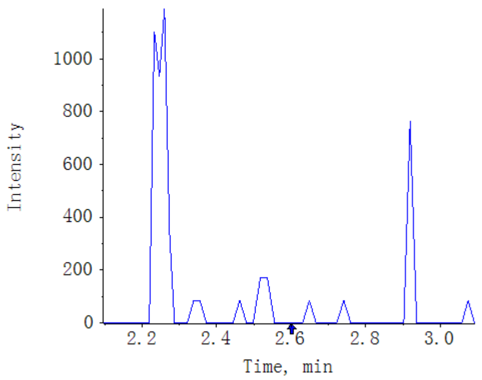

A21233263b_b
Typhaneoside AREA:1.61e4 S/N:23.0

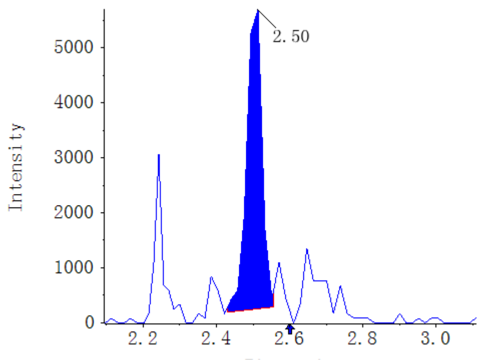

A21233264b_b
Typhaneoside AREA:N/A S/N:N/A

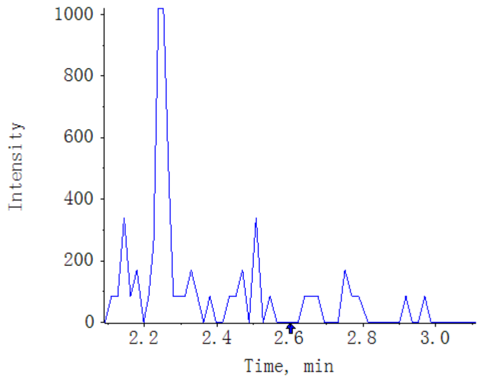

A21233265b_b
Typhaneoside AREA:3.11e3 S/N:8.4

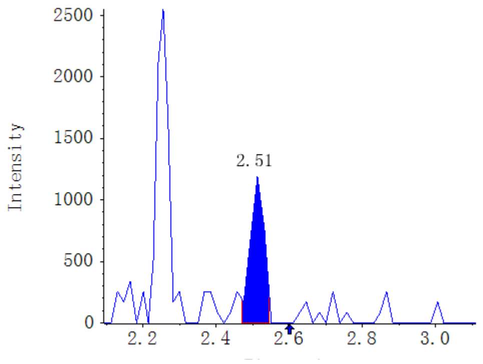

A21233266b_b
Typhaneoside AREA:2.56e3 S/N:7.2

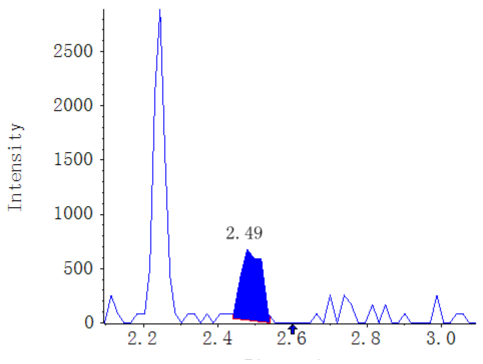

A21233267b_b
Typhaneoside AREA:1.10e4 S/N:22.5

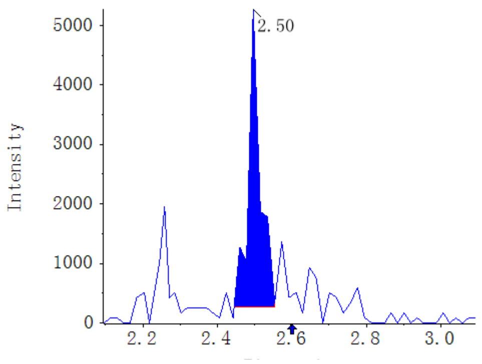

Result Table	MWXS-21-2101D-3_18_WH6500-5_A20-3_V1.0_TY_20211028	Algorithm Used	MQ4
Acquisition Method	Flavonoids_V1.0_WH6500-5_LT_20211025.dam	Instrument Name	QTRAP 6500+ Low Mass
Project	N/A	Analytes QTY	204:91

Compound name: Quercitrin (447.1 / 300.0)

Sample Name	Sample Type	Area (cps)	Is Area (cps)	RT (min)	S/N	Target Conc	Calculated Conc.()
STD_0.5nM	Standard	2.76e3	N/A	3.04	23.1	0.5000	3.035567e-1
STD_1nM	Standard	7.40e3	N/A	3.03	33.8	1.0000	1.117109e0
STD_5nM	Standard	3.64e4	N/A	3.03	95.9	5.0000	6.199276e0
STD_10nM	Standard	6.26e4	N/A	3.03	128.1	10.0000	1.079422e1
STD_20nM	Standard	1.09e5	N/A	3.03	192.8	20.0000	1.898660e1
STD_50nM	Standard	2.95e5	N/A	3.03	146.1	50.0000	5.151102e1
STD_100nM	Standard	5.59e5	N/A	3.04	180.5	100.0000	9.780161e1
STD_200nM	Standard	1.14e6	N/A	3.03	202.7	200.0000	1.997866e2
STD_500nM	Standard	N/A	N/A	N/A	N/A	500.0000	N/A
STD_1000nM	Standard	N/A	N/A	N/A	N/A	1000.0000	N/A
STD_2000nM	Standard	N/A	N/A	N/A	N/A	2000.0000	N/A
V1.0_MW_RQC1_20211018	Quality Control	N/A	N/A	N/A	N/A	0.0000	N/A
Blank	Unknown	N/A	N/A	N/A	N/A	N/A	N/A
V1.0_MWMS_20211021_1	Unknown	1.00e6	N/A	3.05	238.9	N/A	1.758412e2
MWXS212101D3_R1	Quality Control	1.04e6	N/A	3.04	229.0	0.0000	1.826871e2
MWXS212101D3_R2	Quality Control	9.79e5	N/A	3.05	210.5	0.0000	1.713469e2
MWXS212101D3_R3	Quality Control	1.01e6	N/A	3.05	205.6	0.0000	1.767022e2
A21233250b_b	Unknown	1.93e4	N/A	3.04	67.9	N/A	3.199920e0
A21233251b_b	Unknown	5.16e3	N/A	3.05	15.0	N/A	7.247634e-1
A21233252b_b	Unknown	1.39e3	N/A	3.06	8.7	N/A	6.286767e-2
A21233253b_b	Unknown	5.05e3	N/A	3.04	20.3	N/A	7.057488e-1
A21233254b_b	Unknown	2.13e3	N/A	3.05	10.6	N/A	1.931427e-1
A21233255b_b	Unknown	N/A	N/A	N/A	N/A	N/A	N/A
A21233256b_b	Unknown	3.18e3	N/A	3.05	14.4	N/A	3.773837e-1
A21233257b_b	Unknown	2.64e3	N/A	3.05	16.6	N/A	2.833795e-1
A21233258b_b	Unknown	N/A	N/A	N/A	N/A	N/A	N/A
A21233259b_b	Unknown	8.53e3	N/A	3.05	23.5	N/A	1.314393e0
A21233260b_b	Unknown	4.94e4	N/A	3.04	94.4	N/A	8.484925e0
A21233261b_b	Unknown	9.50e4	N/A	3.05	115.5	N/A	1.645880e1
A21233262b_b	Unknown	N/A	N/A	N/A	N/A	N/A	N/A
A21233263b_b	Unknown	N/A	N/A	N/A	N/A	N/A	N/A
A21233264b_b	Unknown	6.18e3	N/A	3.04	14.6	N/A	9.025556e-1
A21233265b_b	Unknown	N/A	N/A	N/A	N/A	N/A	N/A
A21233266b_b	Unknown	N/A	N/A	N/A	N/A	N/A	N/A
A21233267b_b	Unknown	9.66e3	N/A	3.06	44.8	N/A	1.512393e0

Compound name: Quercitrin
Regression Equation: y = 5706.55627 x + 1027.38360 (r = 0.99920) (weighting: 1 / x)

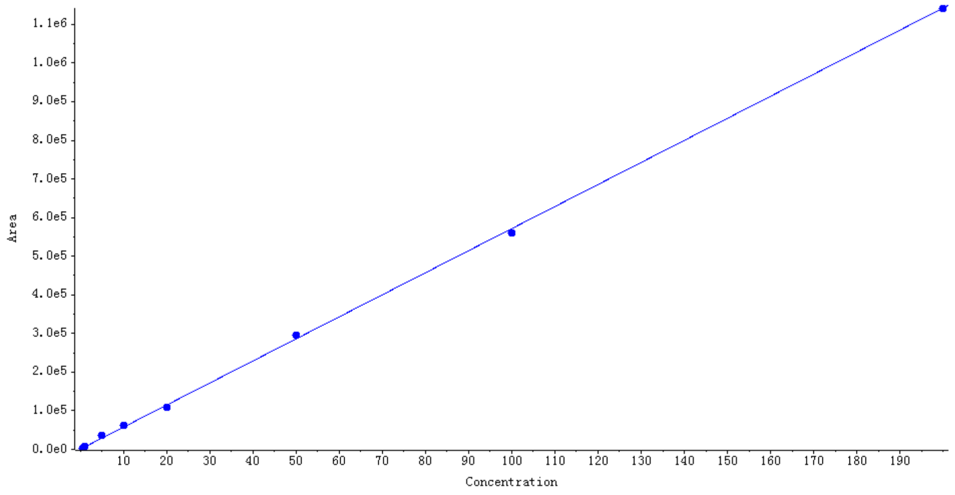

Peak Review

Blank
Quercitrin AREA:N/A S/N:N/A

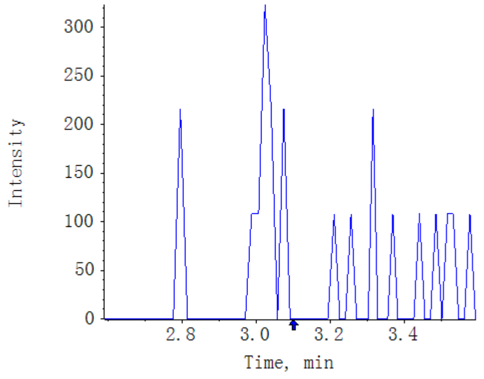

V1.0_MWMS_20211021_1
Quercitrin AREA:1.00e6 S/N:238.9

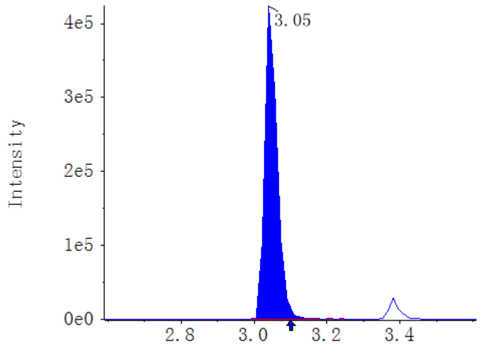

A21233250b_b
Quercitrin AREA:1.93e4 S/N:67.9

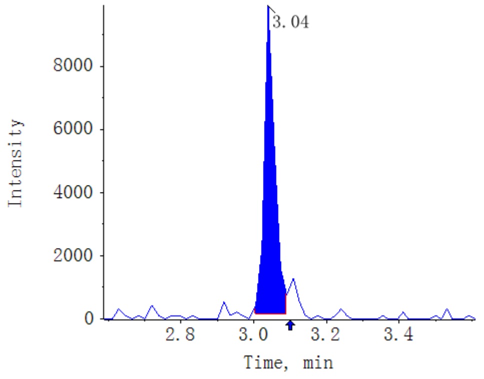

A21233251b_b
Quercitrin AREA:5.16e3 S/N:15.0

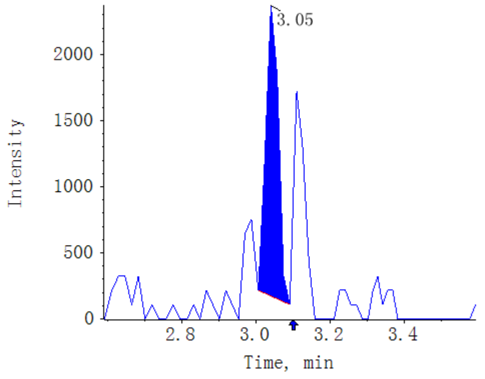

A21233252b_b
Quercitrin AREA:1.39e3 S/N:8.7

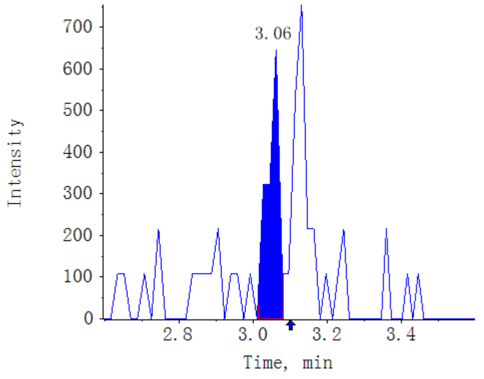

A21233253b_b
Quercitrin AREA:5.05e3 S/N:20.3

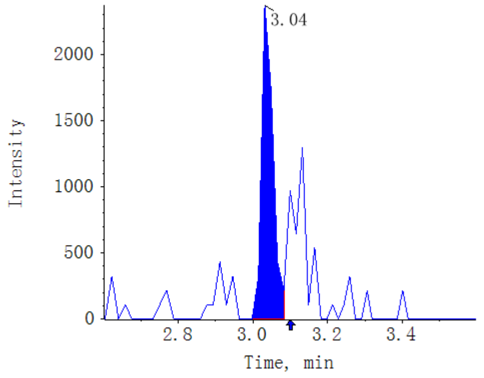

A21233254b_b
Quercitrin AREA:2.13e3 S/N:10.6

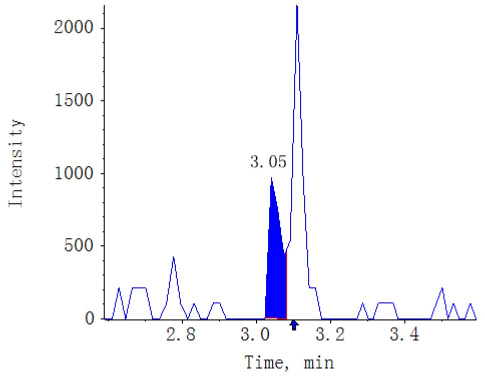

A21233255b_b
Quercitrin AREA:N/A S/N:N/A

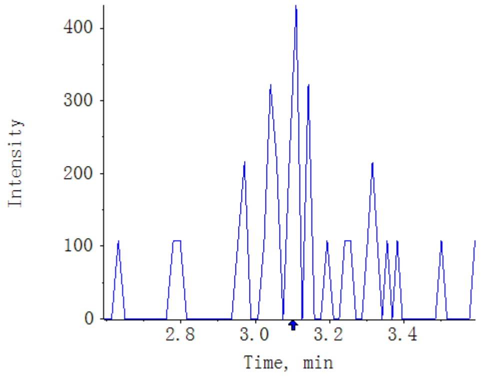

A21233256b_b
Quercitrin AREA:3.18e3 S/N:14.4

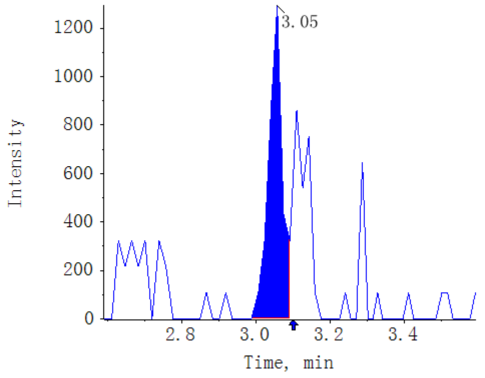

A21233257b_b
Quercitrin AREA:2.64e3 S/N:16.6

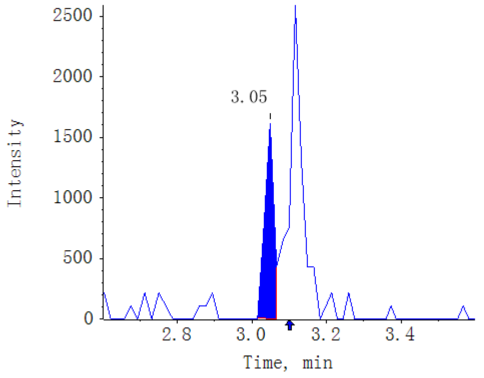

A21233258b_b
Quercitrin AREA:N/A S/N:N/A

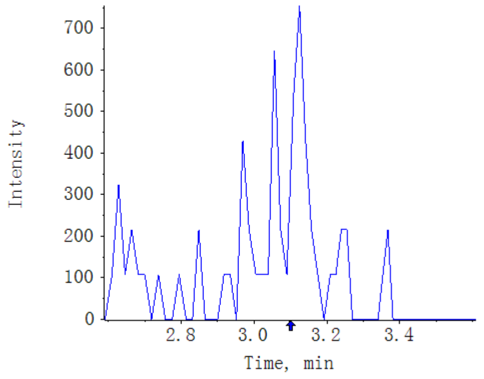

A21233259b_b
Quercitrin AREA:8.53e3 S/N:23.5

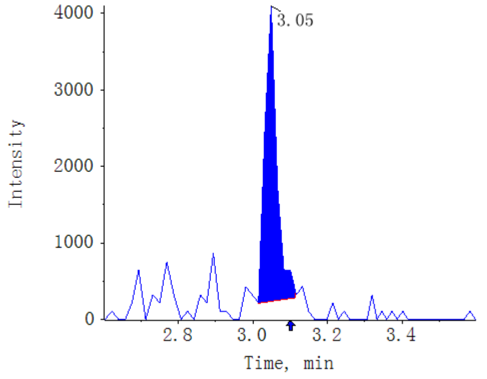

A21233260b_b
Quercitrin AREA:4.94e4 S/N:94.4

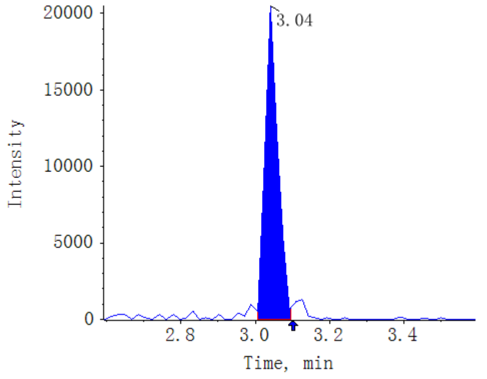

A21233261b_b
Quercitrin AREA:9.50e4 S/N:115.5

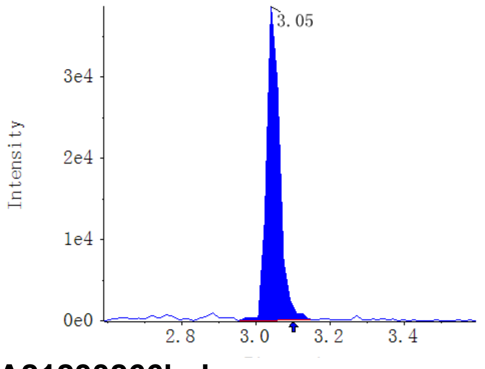

A21233262b_b
Quercitrin AREA:N/A S/N:N/A

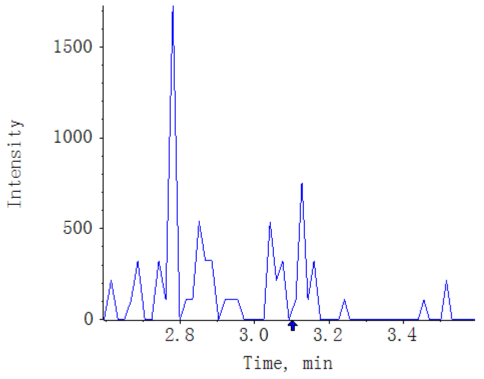

A21233263b_b
Quercitrin AREA:N/A S/N:N/A

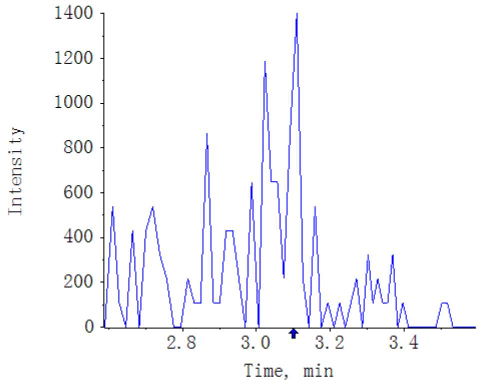

A21233264b_b
Quercitrin AREA:6.18e3 S/N:14.6

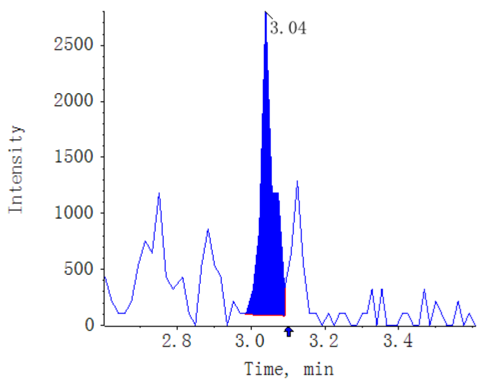

A21233265b_b
Quercitrin AREA:N/A S/N:N/A

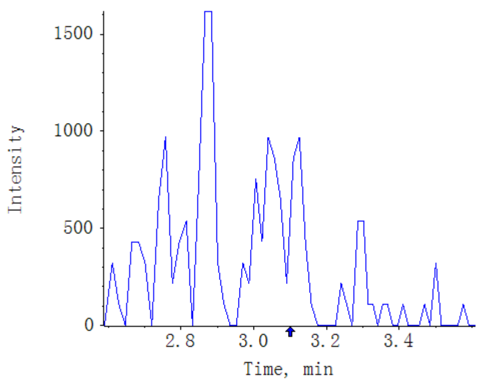

A21233266b_b
Quercitrin AREA:N/A S/N:N/A

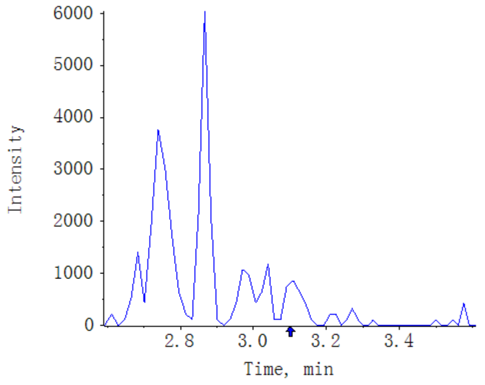

A21233267b_b
Quercitrin AREA:9.66e3 S/N:44.8

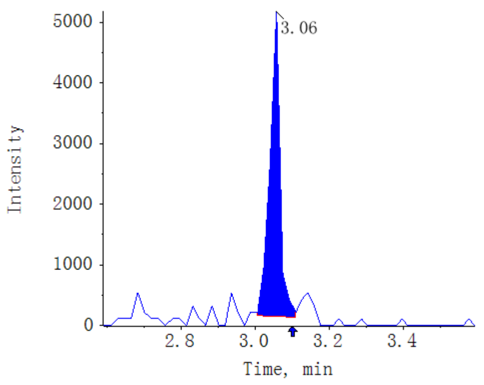

Result Table	MWXS-21-2101D-3_18_WH6500-5_A20-3_V1.0_TY_20211028	Algorithm Used	MQ4
Acquisition Method	Flavonoids_V1.0_WH6500-5_LT_20211025.dam	Instrument Name	QTRAP 6500+ Low Mass
Project	N/A	Analytes QTY	204:93

Compound name: Engeletin (433.1 / 269.0)

Sample Name	Sample Type	Area (cps)	Is Area (cps)	RT (min)	S/N	Target Conc	Calculated Conc.()
STD_0.5nM	Standard	N/A	N/A	N/A	N/A	0.5000	N/A
STD_1nM	Standard	N/A	N/A	N/A	N/A	1.0000	N/A
STD_5nM	Standard	2.30e4	N/A	3.16	78.7	5.0000	6.057708e0
STD_10nM	Standard	3.08e4	N/A	3.16	58.4	10.0000	8.909377e0
STD_20nM	Standard	5.55e4	N/A	3.16	78.4	20.0000	1.789671e1
STD_50nM	Standard	1.42e5	N/A	3.17	95.2	50.0000	4.944071e1
STD_100nM	Standard	2.82e5	N/A	3.17	133.3	100.0000	1.000787e2
STD_200nM	Standard	5.64e5	N/A	3.16	100.5	200.0000	2.026168e2
STD_500nM	Standard	N/A	N/A	N/A	N/A	500.0000	N/A
STD_1000nM	Standard	N/A	N/A	N/A	N/A	1000.0000	N/A
STD_2000nM	Standard	N/A	N/A	N/A	N/A	2000.0000	N/A
V1.0_MW_RQC1_20211018	Quality Control	N/A	N/A	N/A	N/A	0.0000	N/A
Blank	Unknown	N/A	N/A	N/A	N/A	N/A	N/A
V1.0_MWMS_20211021_1	Unknown	5.21e5	N/A	3.18	121.9	N/A	1.872336e2
MWXS212101D3_R1	Quality Control	4.73e5	N/A	3.17	131.6	0.0000	1.696093e2
MWXS212101D3_R2	Quality Control	4.77e5	N/A	3.18	110.3	0.0000	1.712170e2
MWXS212101D3_R3	Quality Control	4.68e5	N/A	3.18	106.0	0.0000	1.676677e2
A21233250b_b	Unknown	N/A	N/A	N/A	N/A	N/A	N/A
A21233251b_b	Unknown	N/A	N/A	N/A	N/A	N/A	N/A
A21233252b_b	Unknown	N/A	N/A	N/A	N/A	N/A	N/A
A21233253b_b	Unknown	1.07e4	N/A	3.15	6.6	N/A	1.609031e0
A21233254b_b	Unknown	8.89e3	N/A	3.14	11.5	N/A	9.419230e-1
A21233255b_b	Unknown	1.14e4	N/A	3.15	11.5	N/A	1.850715e0
A21233256b_b	Unknown	8.11e3	N/A	3.15	8.2	N/A	6.607587e-1
A21233257b_b	Unknown	1.02e4	N/A	3.15	7.8	N/A	1.433898e0
A21233258b_b	Unknown	6.44e3	N/A	3.15	7.3	N/A	5.171781e-2
A21233259b_b	Unknown	N/A	N/A	N/A	N/A	N/A	N/A
A21233260b_b	Unknown	N/A	N/A	N/A	N/A	N/A	N/A
A21233261b_b	Unknown	N/A	N/A	N/A	N/A	N/A	N/A
A21233262b_b	Unknown	N/A	N/A	N/A	N/A	N/A	N/A
A21233263b_b	Unknown	1.18e4	N/A	3.15	5.5	N/A	2.015040e0
A21233264b_b	Unknown	N/A	N/A	N/A	N/A	N/A	N/A
A21233265b_b	Unknown	1.05e4	N/A	3.16	3.8	N/A	1.536654e0
A21233266b_b	Unknown	7.96e3	N/A	3.15	4.6	N/A	6.035617e-1
A21233267b_b	Unknown	N/A	N/A	N/A	N/A	N/A	N/A

Compound name: Engeletin
Regression Equation: y = 2750.71378 x + 6296.33056 (r = 0.99896) (weighting: 1 / x)

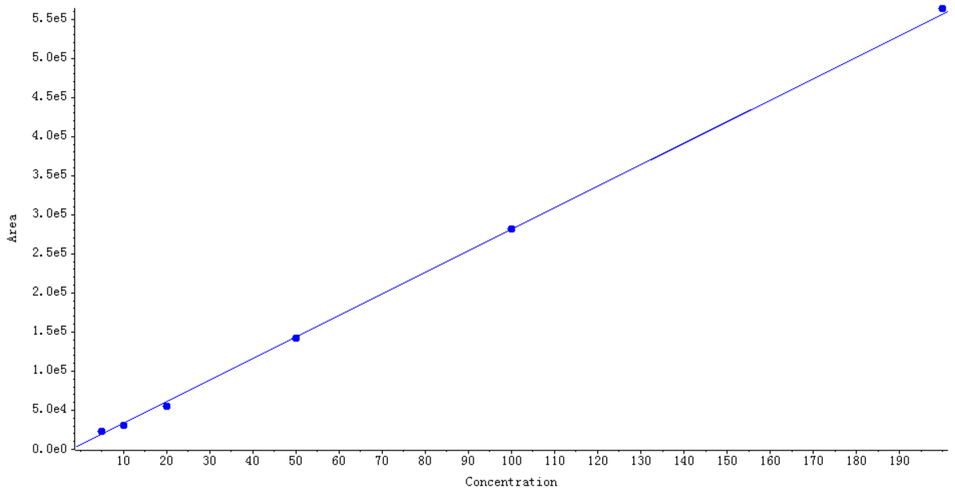

Peak Review

Blank
Engeletin AREA:N/A S/N:N/A

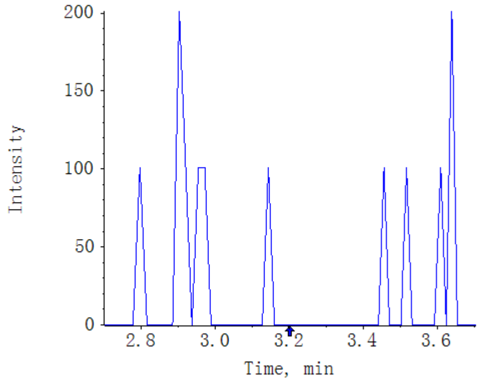

V1.0_MWMS_20211021_1
Engeletin AREA:5.21e5 S/N:121.9

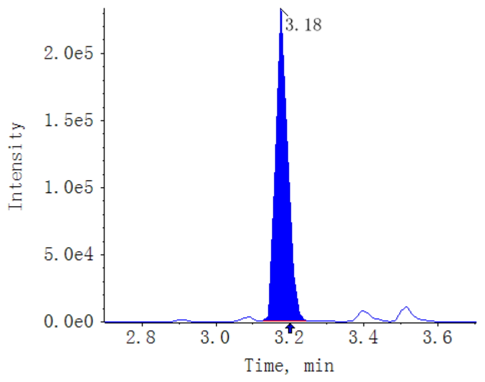

A21233250b_b
Engeletin AREA:N/A S/N:N/A

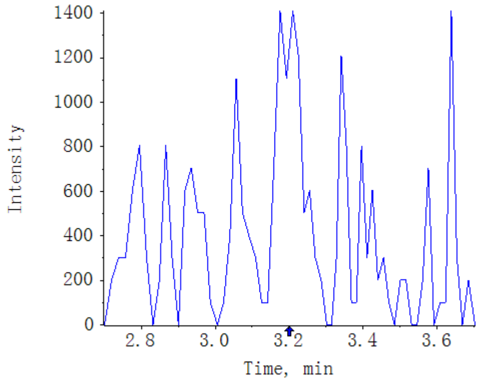

A21233251b_b
Engeletin AREA:N/A S/N:N/A

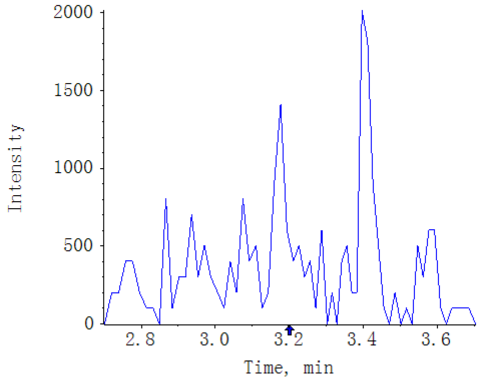

A21233252b_b
Engeletin AREA:N/A S/N:N/A

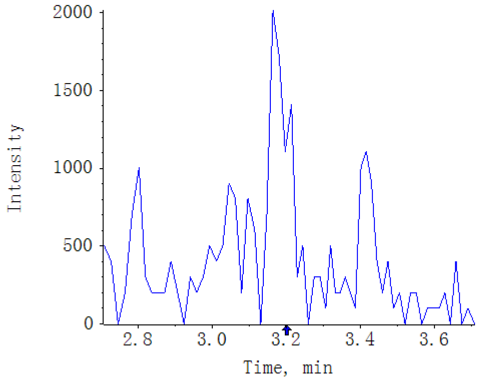

A21233253b_b
Engeletin AREA:1.07e4 S/N:6.6

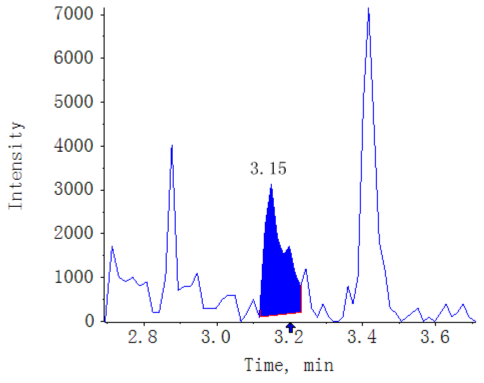

A21233254b_b
Engeletin AREA:8.89e3 S/N:11.5

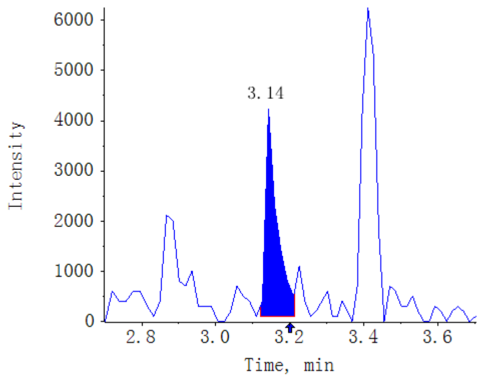

A21233255b_b
Engeletin AREA:1.14e4 S/N:11.5

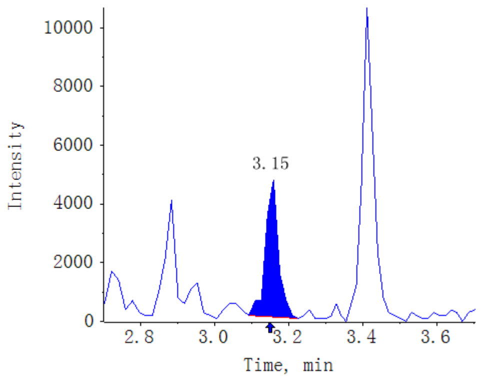

A21233256b_b
Engeletin AREA:8.11e3 S/N:8.2

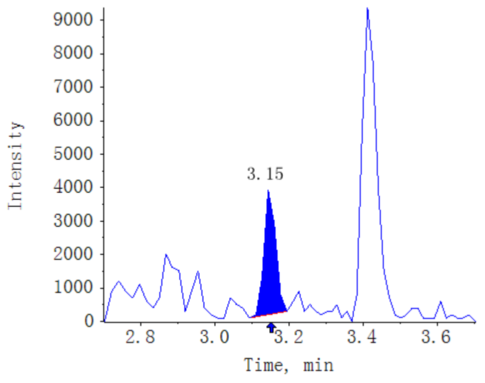

A21233257b_b
Engeletin AREA:1.02e4 S/N:7.8

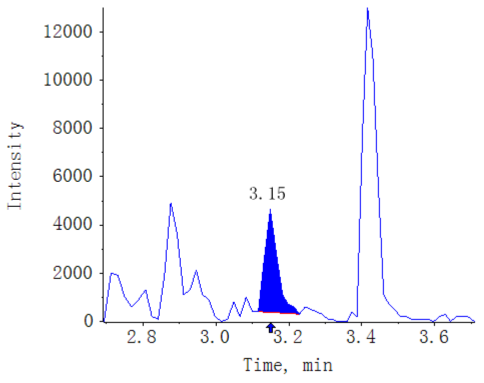

A21233258b_b
Engeletin AREA:6.44e3 S/N:7.3

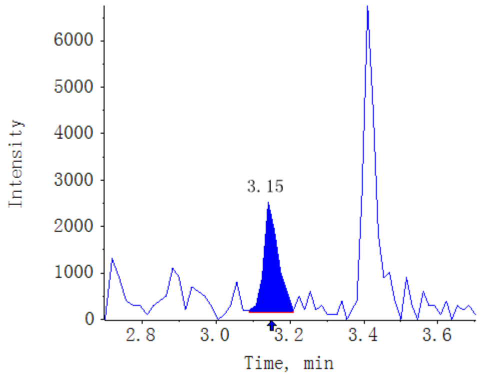

A21233259b_b
Engeletin AREA:N/A S/N:N/A

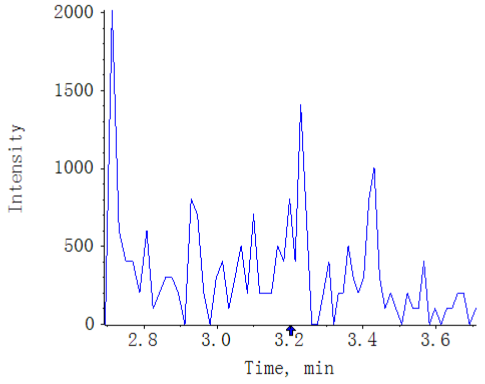

A21233260b_b
Engeletin AREA:N/A S/N:N/A

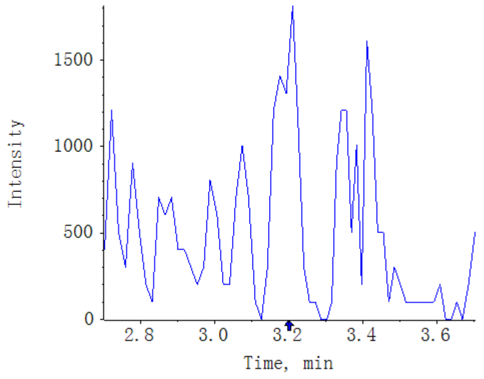

A21233261b_b
Engeletin AREA:N/A S/N:N/A

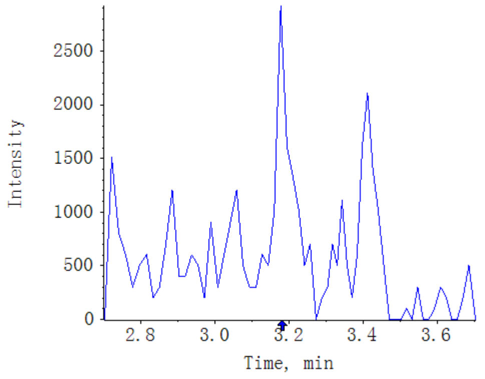

A21233262b_b
Engeletin AREA:N/A S/N:N/A

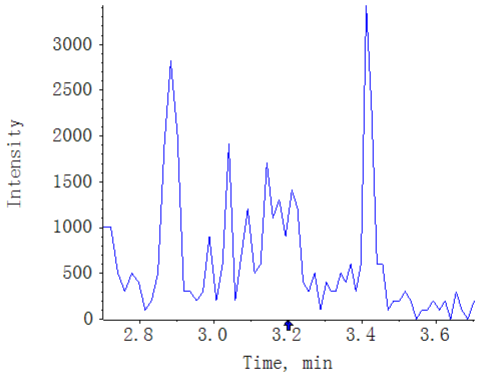

A21233263b_b
Engeletin AREA:1.18e4 S/N:5.5

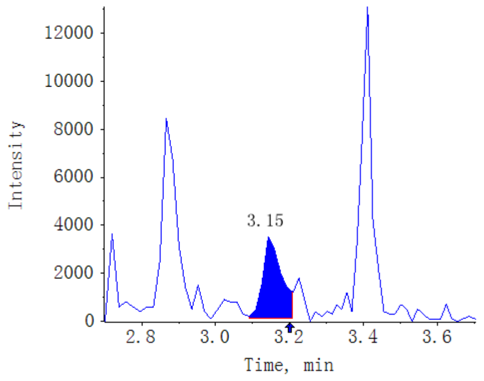

A21233264b_b
Engeletin AREA:N/A S/N:N/A

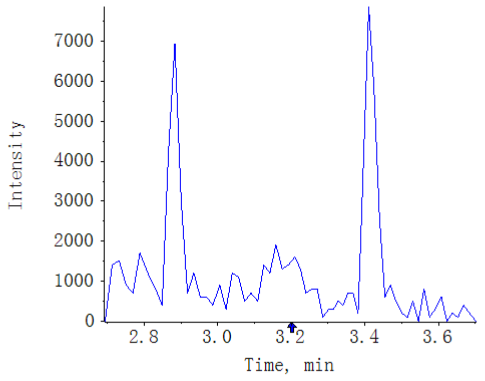

A21233265b_b
Engeletin AREA:1.05e4 S/N:3.8

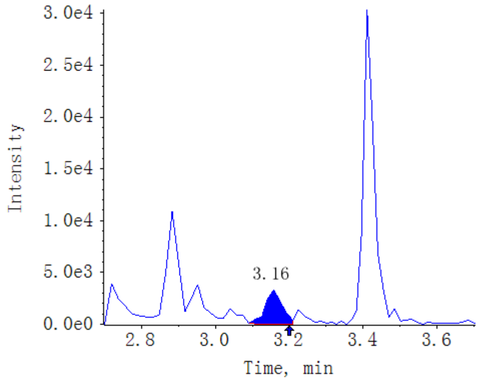

A21233266b_b
Engeletin AREA:7.96e3 S/N:4.6

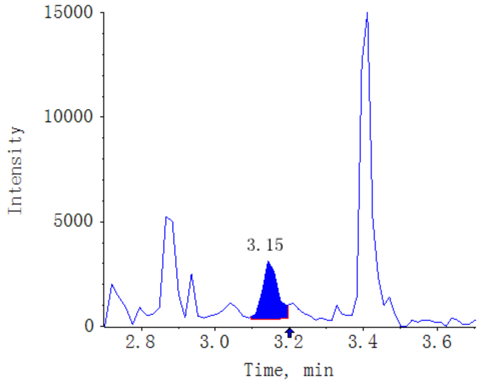

A21233267b_b
Engeletin AREA:N/A S/N:N/A

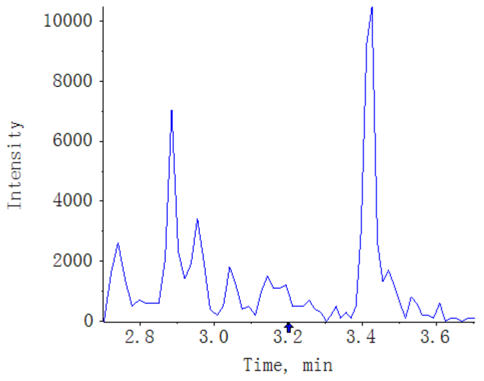

Result Table	MWXS-21-2101D-3_18_WH6500-5_A20-3_V1.0_TY_20211028	Algorithm Used	MQ4
Acquisition Method	Flavonoids_V1.0_WH6500-5_LT_20211025.dam	Instrument Name	QTRAP 6500+ Low Mass
Project	N/A	Analytes QTY	204:94

Compound name: Narcissin (623.2 / 315.1)

Sample Name	Sample Type	Area (cps)	Is Area (cps)	RT (min)	S/N	Target Conc	Calculated Conc.()
STD_0.5nM	Standard	3.35e3	N/A	2.87	25.1	0.5000	5.836912e-1
STD_1nM	Standard	7.17e3	N/A	2.86	21.7	1.0000	1.136665e0
STD_5nM	Standard	4.94e4	N/A	2.86	123.9	5.0000	7.254035e0
STD_10nM	Standard	7.19e4	N/A	2.86	120.1	10.0000	1.051355e1
STD_20nM	Standard	1.25e5	N/A	2.86	105.9	20.0000	1.816957e1
STD_50nM	Standard	3.45e5	N/A	2.86	154.2	50.0000	5.004327e1
STD_100nM	Standard	6.72e5	N/A	2.87	143.0	100.0000	9.741983e1
STD_200nM	Standard	1.39e6	N/A	2.86	171.7	200.0000	2.013794e2
STD_500nM	Standard	N/A	N/A	N/A	N/A	500.0000	N/A
STD_1000nM	Standard	N/A	N/A	N/A	N/A	1000.0000	N/A
STD_2000nM	Standard	N/A	N/A	N/A	N/A	2000.0000	N/A
V1.0_MW_RQC1_20211018	Quality Control	1.58e6	N/A	2.85	150.3	0.0000	2.293237e2
Blank	Unknown	N/A	N/A	N/A	N/A	N/A	N/A
V1.0_MWMS_20211021_1	Unknown	1.14e6	N/A	2.87	199.2	N/A	1.649882e2
MWXS212101D3_R1	Quality Control	1.21e6	N/A	2.87	154.3	0.0000	1.757288e2
MWXS212101D3_R2	Quality Control	1.17e6	N/A	2.87	165.0	0.0000	1.701694e2
MWXS212101D3_R3	Quality Control	1.18e6	N/A	2.87	201.3	0.0000	1.709834e2
A21233250b_b	Unknown	7.53e5	N/A	2.87	253.2	N/A	1.092459e2
A21233251b_b	Unknown	5.75e5	N/A	2.87	219.6	N/A	8.342214e1
A21233252b_b	Unknown	4.64e5	N/A	2.87	240.6	N/A	6.730327e1
A21233253b_b	Unknown	1.74e6	N/A	2.87	345.8	N/A	2.521510e2
A21233254b_b	Unknown	3.14e5	N/A	2.87	162.8	N/A	4.560505e1
A21233255b_b	Unknown	2.86e5	N/A	2.87	174.2	N/A	4.154211e1
A21233256b_b	Unknown	3.05e5	N/A	2.87	169.5	N/A	4.429247e1
A21233257b_b	Unknown	1.99e5	N/A	2.87	160.6	N/A	2.896388e1
A21233258b_b	Unknown	2.22e5	N/A	2.87	168.5	N/A	3.226761e1
A21233259b_b	Unknown	2.77e5	N/A	2.87	208.5	N/A	4.022332e1
A21233260b_b	Unknown	4.14e5	N/A	2.87	202.0	N/A	6.015648e1
A21233261b_b	Unknown	3.47e5	N/A	2.87	214.2	N/A	5.034762e1
A21233262b_b	Unknown	3.83e5	N/A	2.87	246.5	N/A	5.563878e1
A21233263b_b	Unknown	4.97e5	N/A	2.86	222.8	N/A	7.214726e1
A21233264b_b	Unknown	6.19e5	N/A	2.87	266.6	N/A	8.982567e1
A21233265b_b	Unknown	3.64e5	N/A	2.87	238.3	N/A	5.280559e1
A21233266b_b	Unknown	3.44e5	N/A	2.86	281.7	N/A	4.991199e1
A21233267b_b	Unknown	2.94e5	N/A	2.88	155.9	N/A	4.273172e1

Compound name: Narcissin
Regression Equation: y = 6901.58193 x + -675.92267 (r = 0.99975) (weighting: None)

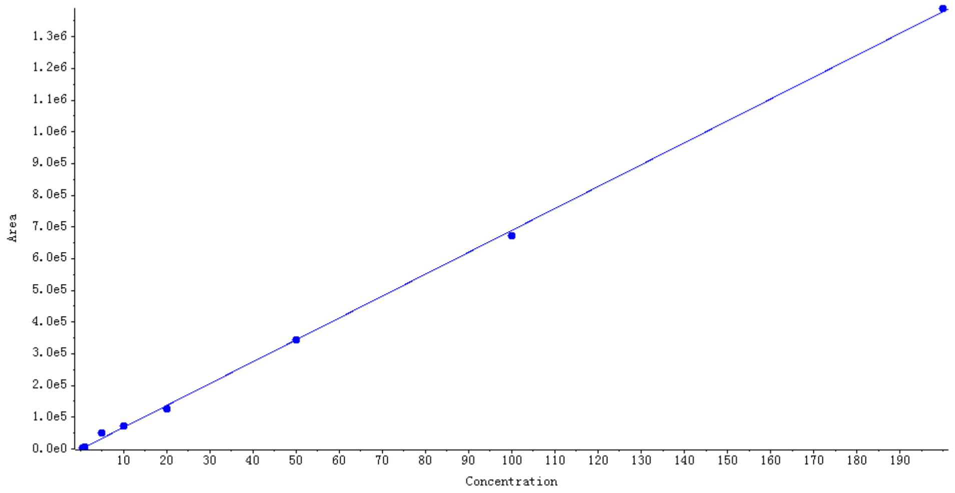

Peak Review

Blank
Narcissin AREA:N/A S/N:N/A

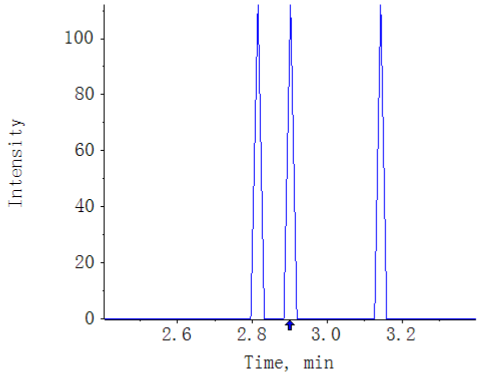

V1.0_MWMS_20211021_1
Narcissin AREA:1.14e6 S/N:199.2

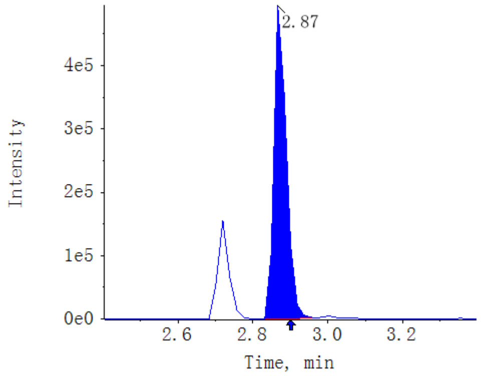

A21233250b_b
Narcissin AREA:7.53e5 S/N:253.2

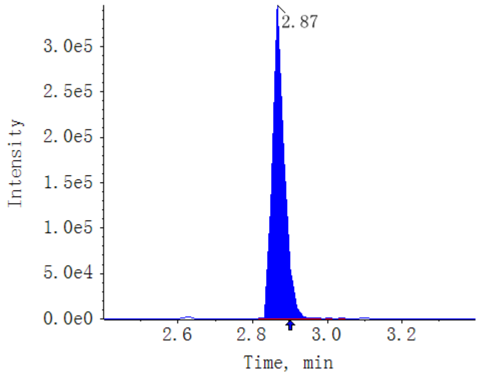

A21233251b_b
Narcissin AREA:5.75e5 S/N:219.6

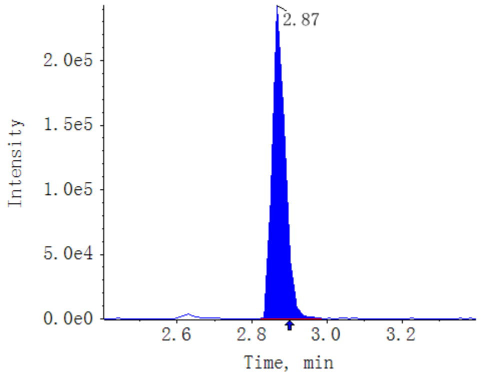

A21233252b_b
Narcissin AREA:4.64e5 S/N:240.6

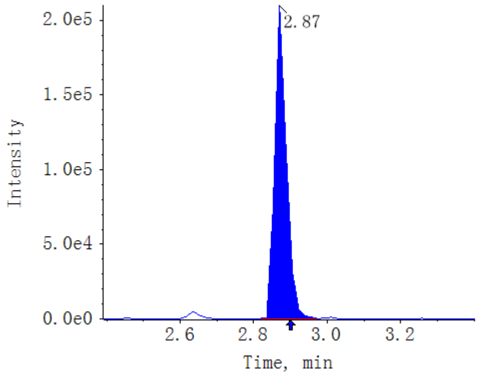

A21233253b_b
Narcissin AREA:1.74e6 S/N:345.8

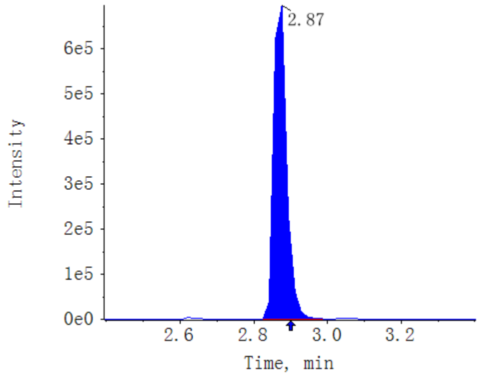

A21233254b_b
Narcissin AREA:3.14e5 S/N:162.8

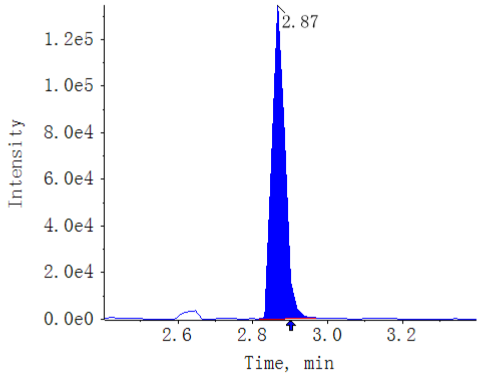

A21233255b_b
Narcissin AREA:2.86e5 S/N:174.2

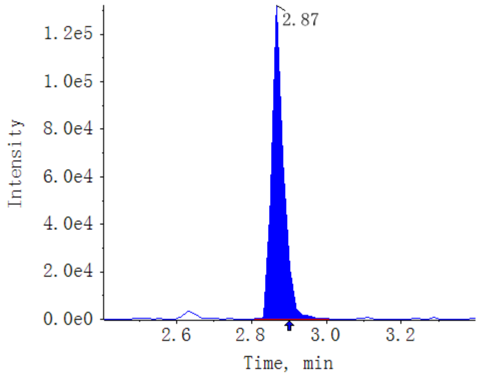

A21233256b_b
Narcissin AREA:3.05e5 S/N:169.5

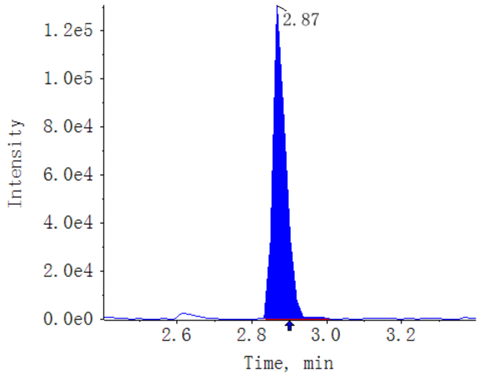

A21233257b_b
Narcissin AREA:1.99e5 S/N:160.6

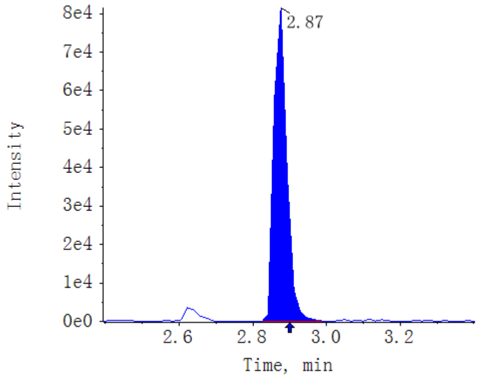

A21233258b_b
Narcissin AREA:2.22e5 S/N:168.5

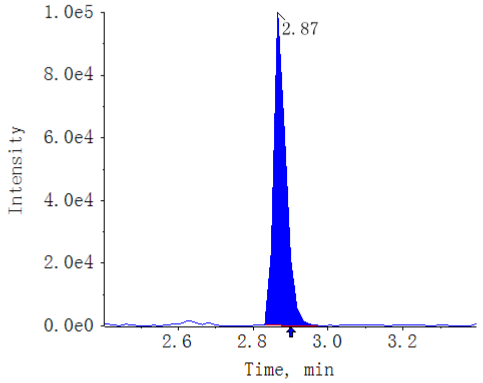

A21233259b_b
Narcissin AREA:2.77e5 S/N:208.5

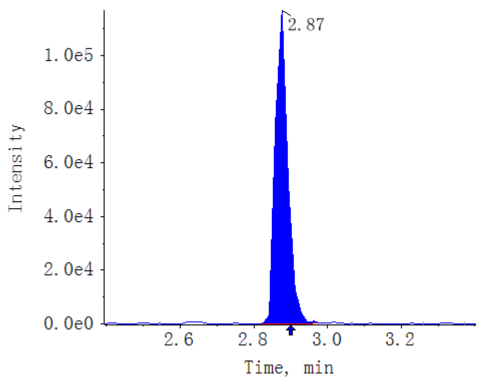

A21233260b_b
Narcissin AREA:4.14e5 S/N:202.0

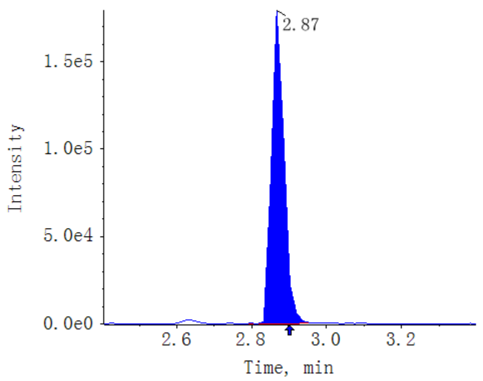

A21233261b_b
Narcissin AREA:3.47e5 S/N:214.2

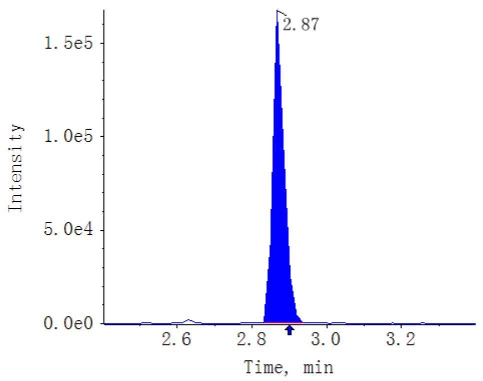

A21233262b_b
Narcissin AREA:3.83e5 S/N:246.5

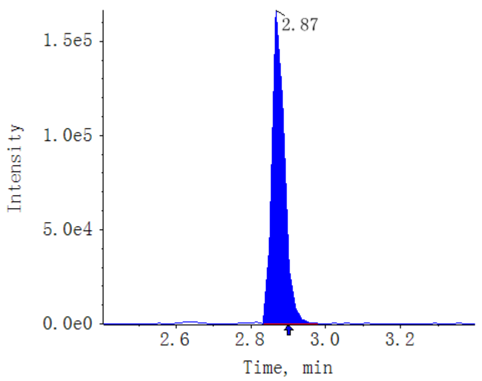

A21233263b_b
Narcissin AREA:4.97e5 S/N:222.8

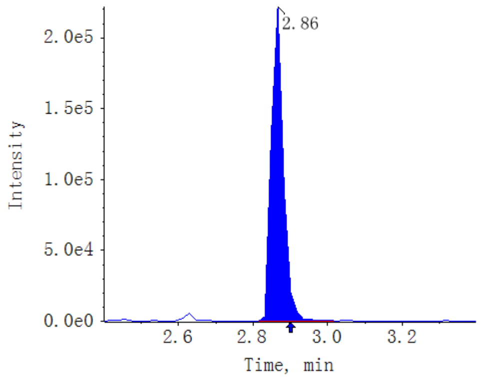

A21233264b_b
Narcissin AREA:6.19e5 S/N:266.6

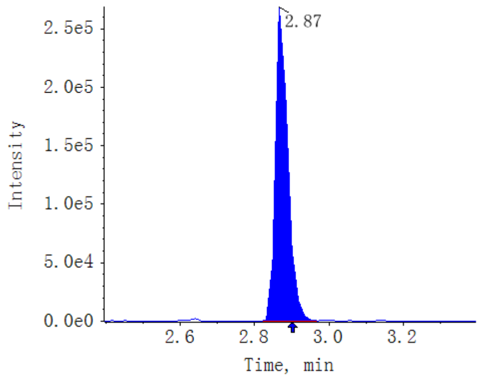

A21233265b_b
Narcissin AREA:3.64e5 S/N:238.3

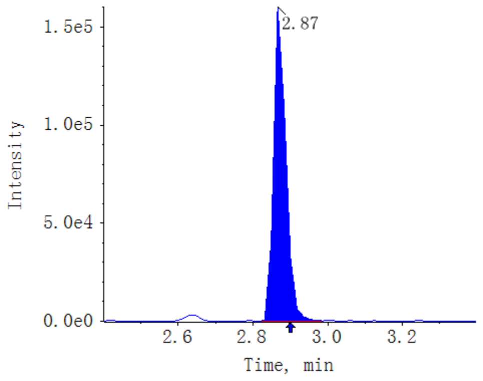

A21233266b_b
Narcissin AREA:3.44e5 S/N:281.7

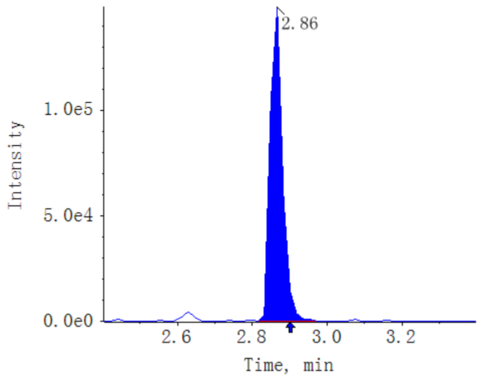

A21233267b_b
Narcissin AREA:2.94e5 S/N:155.9

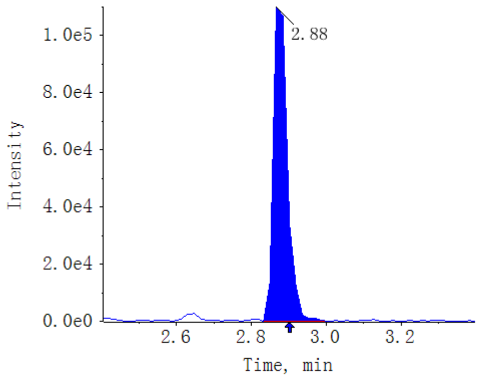

Result Table	MWXS-21-2101D-3_18_WH6500-5_A20-3_V1.0_TY_20211028	Algorithm Used	MQ4
Acquisition Method	Flavonoids_V1.0_WH6500-5_LT_20211025.dam	Instrument Name	QTRAP 6500+ Low Mass
Project	N/A	Analytes QTY	204:95

Compound name: Astragalin (447.1 / 284.0)

Sample Name	Sample Type	Area (cps)	Is Area (cps)	RT (min)	S/N	Target Conc	Calculated Conc.()
STD_0.5nM	Standard	6.36e3	N/A	3.01	37.7	0.5000	4.115691e-1
STD_1nM	Standard	9.84e3	N/A	2.98	43.5	1.0000	7.446589e-1
STD_5nM	Standard	7.48e4	N/A	2.99	107.8	5.0000	6.963325e0
STD_10nM	Standard	1.08e5	N/A	2.98	120.8	10.0000	1.016999e1
STD_20nM	Standard	2.14e5	N/A	2.98	94.9	20.0000	2.028975e1
STD_50nM	Standard	5.43e5	N/A	2.99	149.3	50.0000	5.186810e1
STD_100nM	Standard	1.03e6	N/A	2.99	141.4	100.0000	9.808537e1
STD_200nM	Standard	2.07e6	N/A	2.99	172.8	200.0000	1.979672e2
STD_500nM	Standard	N/A	N/A	N/A	N/A	500.0000	N/A
STD_1000nM	Standard	N/A	N/A	N/A	N/A	1000.0000	N/A
STD_2000nM	Standard	N/A	N/A	N/A	N/A	2000.0000	N/A
V1.0_MW_RQC1_20211018	Quality Control	N/A	N/A	N/A	N/A	0.0000	N/A
Blank	Unknown	N/A	N/A	N/A	N/A	N/A	N/A
V1.0_MWMS_20211021_1	Unknown	1.79e6	N/A	3.00	140.7	N/A	1.715397e2
MWXS212101D3_R1	Quality Control	1.83e6	N/A	2.99	168.5	0.0000	1.748287e2
MWXS212101D3_R2	Quality Control	1.72e6	N/A	3.00	145.7	0.0000	1.646547e2
MWXS212101D3_R3	Quality Control	1.74e6	N/A	3.00	132.1	0.0000	1.663075e2
A21233250b_b	Unknown	3.07e5	N/A	2.99	55.5	N/A	2.922150e1
A21233251b_b	Unknown	1.82e5	N/A	2.99	54.5	N/A	1.720590e1
A21233252b_b	Unknown	2.59e5	N/A	3.00	72.4	N/A	2.461874e1
A21233253b_b	Unknown	7.19e4	N/A	3.00	32.3	N/A	6.690672e0
A21233254b_b	Unknown	1.10e5	N/A	2.99	44.4	N/A	1.036509e1
A21233255b_b	Unknown	1.49e5	N/A	3.00	40.5	N/A	1.406952e1
A21233256b_b	Unknown	6.87e4	N/A	3.00	30.5	N/A	6.380573e0
A21233257b_b	Unknown	1.57e5	N/A	3.00	38.7	N/A	1.485250e1
A21233258b_b	Unknown	3.87e4	N/A	3.00	18.1	N/A	3.511016e0
A21233259b_b	Unknown	4.51e4	N/A	3.00	19.8	N/A	4.124700e0
A21233260b_b	Unknown	1.33e5	N/A	3.00	34.9	N/A	1.250321e1
A21233261b_b	Unknown	1.41e5	N/A	3.00	29.4	N/A	1.330817e1
A21233262b_b	Unknown	8.07e4	N/A	3.00	31.4	N/A	7.535651e0
A21233263b_b	Unknown	1.22e5	N/A	2.99	43.0	N/A	1.145482e1
A21233264b_b	Unknown	8.06e4	N/A	3.00	25.8	N/A	7.526193e0
A21233265b_b	Unknown	4.54e5	N/A	3.00	43.2	N/A	4.331828e1
A21233266b_b	Unknown	1.18e5	N/A	2.99	26.7	N/A	1.110618e1
A21233267b_b	Unknown	5.67e4	N/A	3.00	10.2	N/A	5.233165e0

Compound name: Astragalin
Regression Equation: y = 10438.36926 x + 2065.53246 (r = 0.99866) (weighting: 1 / x)

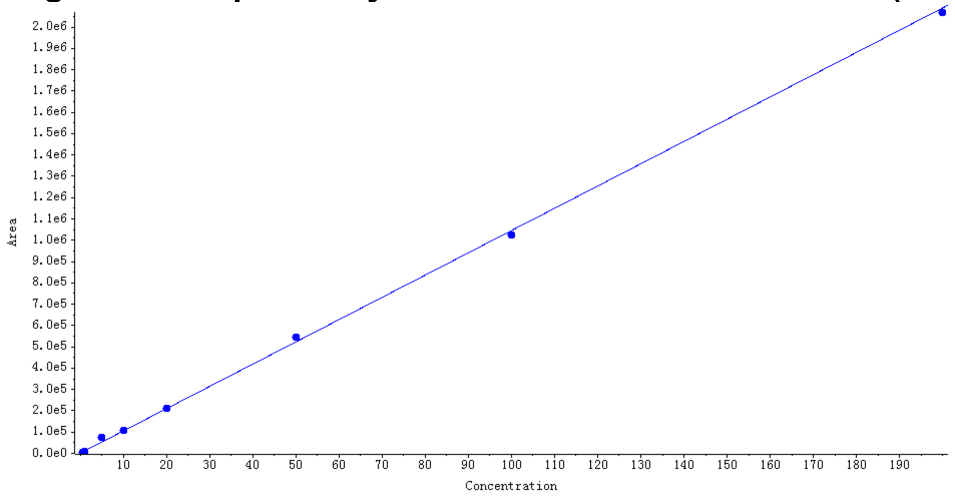

Peak Review

Blank
Astragalin AREA:N/A S/N:N/A

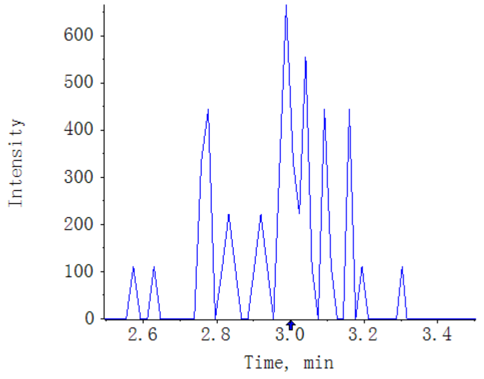

V1.0_MWMS_20211021_1
Astragalin AREA:1.79e6 S/N:140.7

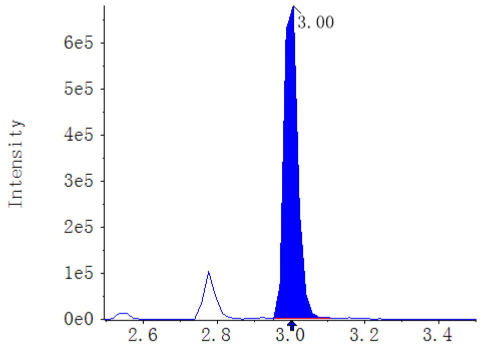

A21233250b_b
Astragalin AREA:3.07e5 S/N:55.5

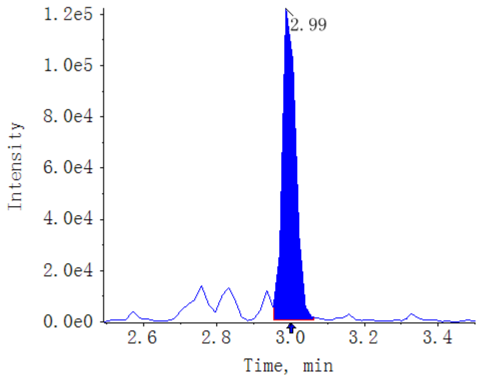

A21233251b_b
Astragalin AREA:1.82e5 S/N:54.5

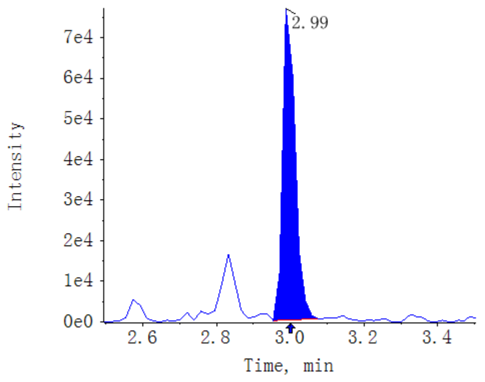

A21233252b_b
Astragalin AREA:2.59e5 S/N:72.4

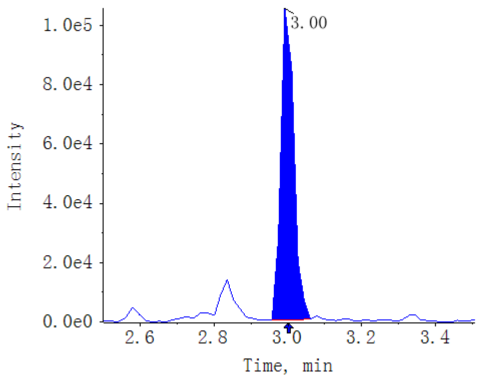

A21233253b_b
Astragalin AREA:7.19e4 S/N:32.3

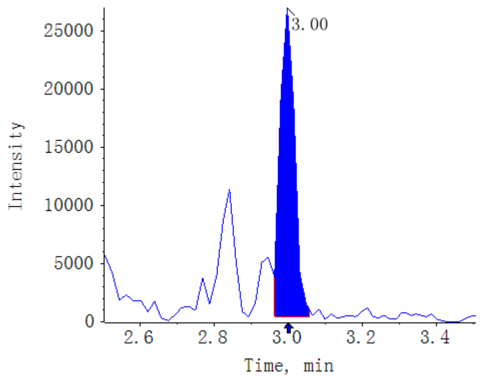

A21233254b_b
Astragalin AREA:1.10e5 S/N:44.4

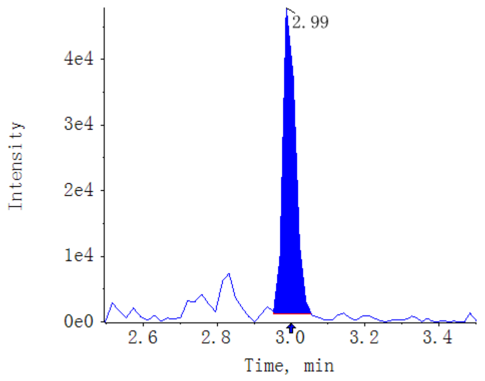

A21233255b_b
Astragalin AREA:1.49e5 S/N:40.5

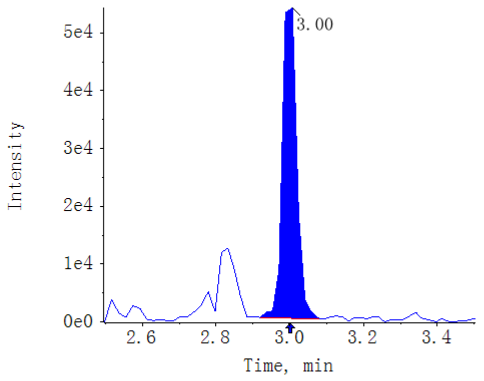

A21233256b_b
Astragalin AREA:6.87e4 S/N:30.5

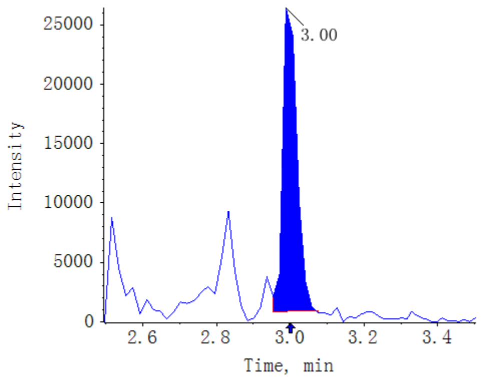

A21233257b_b
Astragalin AREA:1.57e5 S/N:38.7

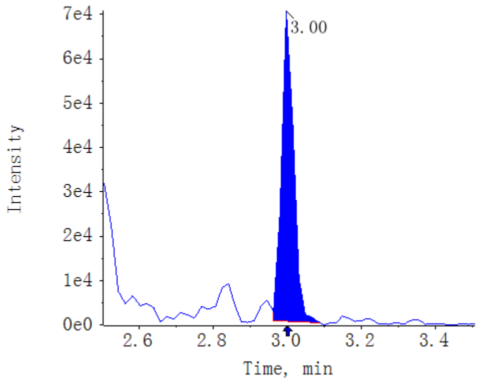

A21233258b_b
Astragalin AREA:3.87e4 S/N:18.1

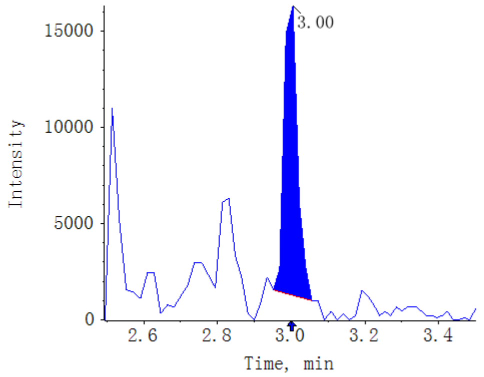

A21233259b_b
Astragalin AREA:4.51e4 S/N:19.8

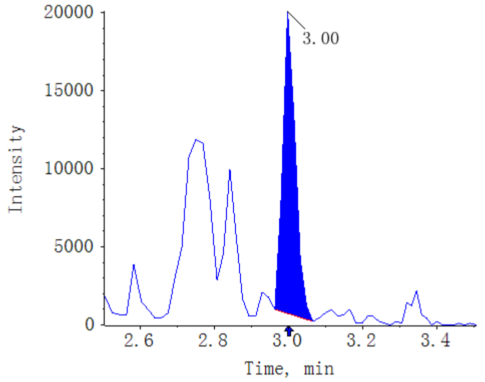

A21233260b_b
Astragalin AREA:1.33e5 S/N:34.9

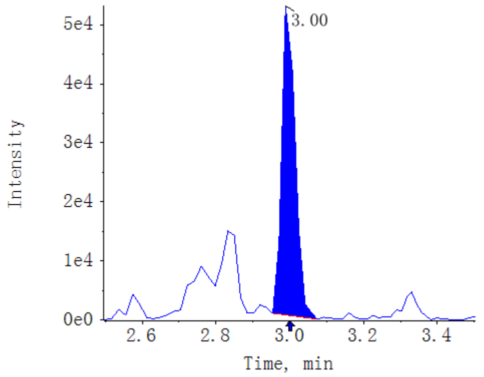

A21233261b_b
Astragalin AREA:1.41e5 S/N:29.4

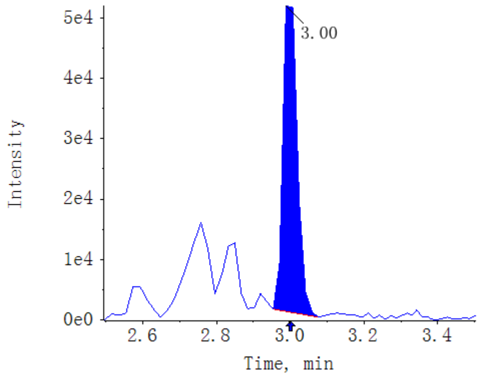

A21233262b_b
Astragalin AREA:8.07e4 S/N:31.4

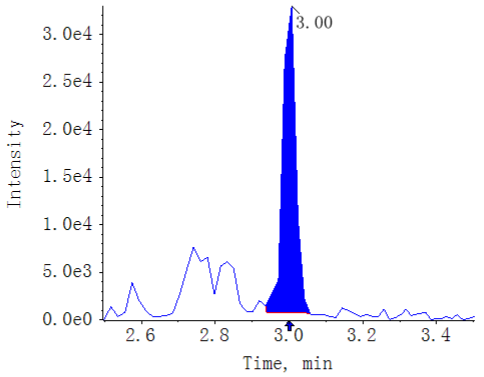

A21233263b_b
Astragalin AREA:1.22e5 S/N:43.0

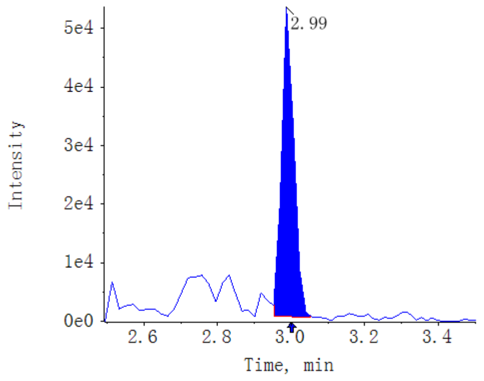

A21233264b_b
Astragalin AREA:8.06e4 S/N:25.8

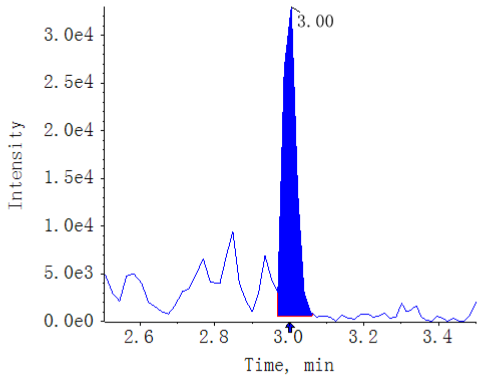

A21233265b_b
Astragalin AREA:4.54e5 S/N:43.2

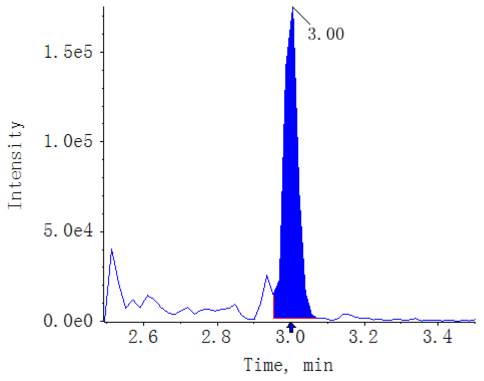

A21233266b_b
Astragalin AREA:1.18e5 S/N:26.7

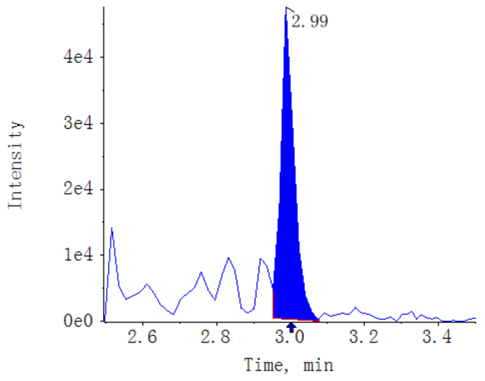

A21233267b_b
Astragalin AREA:5.67e4 S/N:10.2

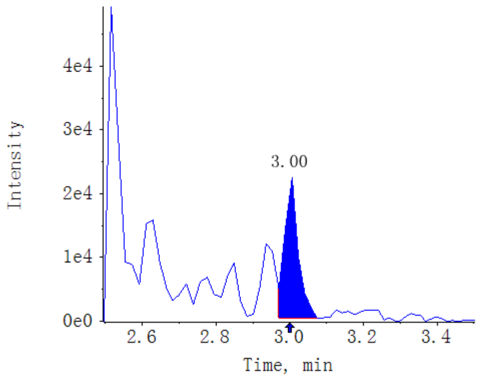

**Result Table**	MWXS-21-2101D-3_18_WH6500-5_A20-3_V1.0_TY_20211028	**Algorithm Used**	MQ4
**Acquisition Method**	Flavonoids_V1.0_WH6500-5_LT_20211025.dam	**Instrument Name**	QTRAP 6500+ Low Mass
**Project**	N/A	**Analytes QTY**	204:100

Compound name: Silychristin (481.1 / 125.0)

Sample Name	Sample Type	Area (cps)	Is Area (cps)	RT (min)	S/N	Target Conc	Calculated Conc.()
STD_0.5nM	Standard	1.64e3	N/A	3.60	13.1	0.5000	3.200131e-1
STD_1nM	Standard	4.00e3	N/A	3.59	25.1	1.0000	8.543609e-1
STD_5nM	Standard	3.26e4	N/A	3.60	142.7	5.0000	7.350802e0
STD_10nM	Standard	4.84e4	N/A	3.60	158.6	10.0000	1.093336e1
STD_20nM	Standard	8.30e4	N/A	3.60	158.8	20.0000	1.877133e1
STD_50nM	Standard	2.30e5	N/A	3.60	218.1	50.0000	5.200960e1
STD_100nM	Standard	4.25e5	N/A	3.61	352.7	100.0000	9.641101e1
STD_200nM	Standard	8.81e5	N/A	3.59	290.0	200.0000	1.998495e2
STD_500nM	Standard	N/A	N/A	N/A	N/A	500.0000	N/A
STD_1000nM	Standard	N/A	N/A	N/A	N/A	1000.0000	N/A
STD_2000nM	Standard	N/A	N/A	N/A	N/A	2000.0000	N/A
V1.0_MW_RQC1_20211018	Quality Control	4.55e4	N/A	3.51	27.6	0.0000	1.027043e1
Blank	Unknown	N/A	N/A	N/A	N/A	N/A	N/A
V1.0_MWMS_20211021_1	Unknown	7.43e5	N/A	3.61	369.7	N/A	1.684876e2
MWXS212101D3_R1	Quality Control	7.65e5	N/A	3.61	371.0	0.0000	1.734005e2
MWXS212101D3_R2	Quality Control	7.68e5	N/A	3.61	308.5	0.0000	1.741906e2
MWXS212101D3_R3	Quality Control	7.36e5	N/A	3.61	343.6	0.0000	1.669507e2
A21233250b_b	Unknown	N/A	N/A	N/A	N/A	N/A	N/A
A21233251b_b	Unknown	N/A	N/A	N/A	N/A	N/A	N/A
A21233252b_b	Unknown	N/A	N/A	N/A	N/A	N/A	N/A
A21233253b_b	Unknown	N/A	N/A	N/A	N/A	N/A	N/A
A21233254b_b	Unknown	8.69e3	N/A	3.55	21.0	N/A	1.918978e0
A21233255b_b	Unknown	6.59e3	N/A	3.53	19.3	N/A	1.442377e0
A21233256b_b	Unknown	1.16e4	N/A	3.55	16.1	N/A	2.579400e0
A21233257b_b	Unknown	1.71e4	N/A	3.55	31.7	N/A	3.831713e0
A21233258b_b	Unknown	7.15e3	N/A	3.55	14.3	N/A	1.570513e0
A21233259b_b	Unknown	N/A	N/A	N/A	N/A	N/A	N/A
A21233260b_b	Unknown	N/A	N/A	N/A	N/A	N/A	N/A
A21233261b_b	Unknown	3.76e3	N/A	3.53	11.4	N/A	8.002980e-1
A21233262b_b	Unknown	3.39e3	N/A	3.54	8.6	N/A	7.178951e-1
A21233263b_b	Unknown	N/A	N/A	N/A	N/A	N/A	N/A
A21233264b_b	Unknown	N/A	N/A	N/A	N/A	N/A	N/A
A21233265b_b	Unknown	1.33e4	N/A	3.55	16.6	N/A	2.961493e0
A21233266b_b	Unknown	5.08e3	N/A	3.53	10.2	N/A	1.100529e0
A21233267b_b	Unknown	N/A	N/A	N/A	N/A	N/A	N/A

Compound name: Silychristin
Regression Equation: y = 4409.39289 x + 227.96057 (r = 0.99788) (weighting: 1 / x)

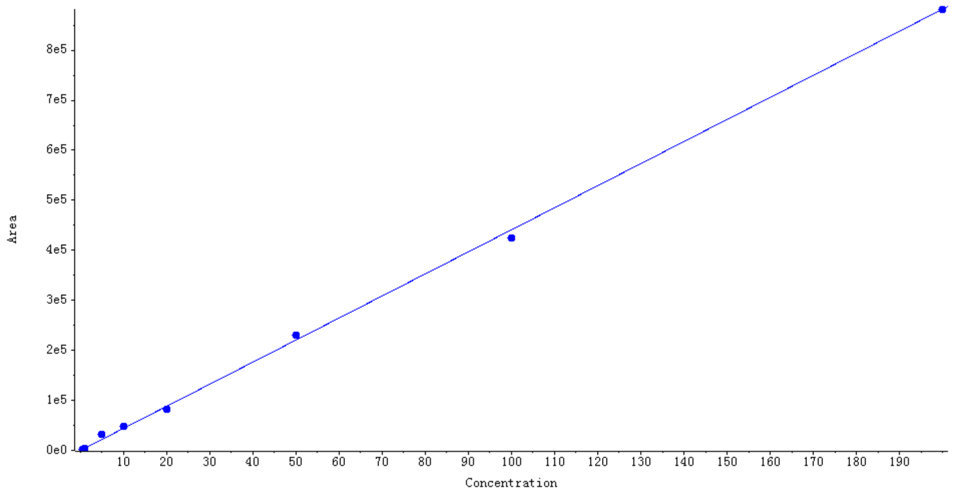

Peak Review

Blank
Silychristin AREA:N/A S/N:N/A

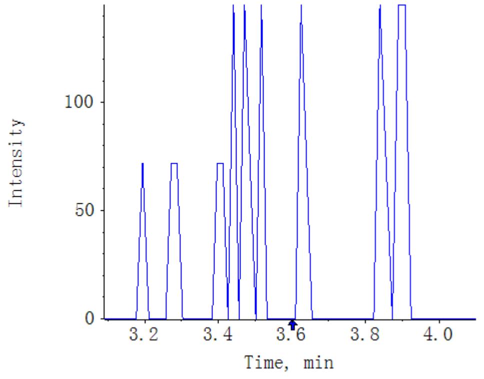

V1.0_MWMS_20211021_1
Silychristin AREA:7.43e5 S/N:369.7

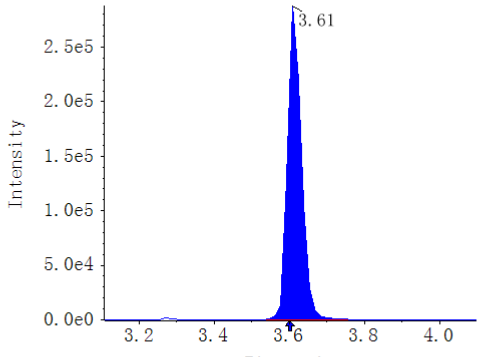

A21233250b_b
Silychristin AREA:N/A S/N:N/A

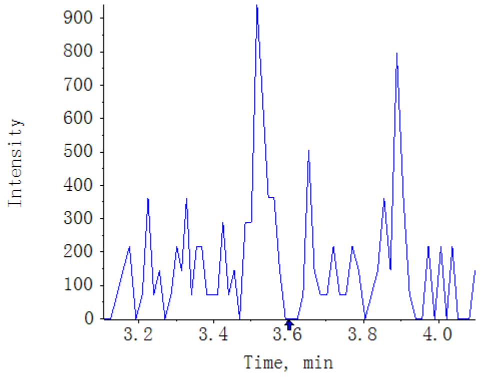

A21233251b_b
Silychristin AREA:N/A S/N:N/A

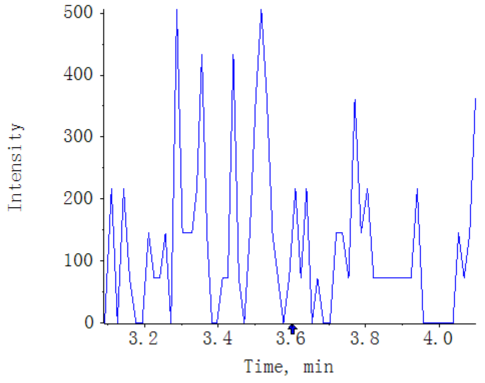

A21233252b_b
Silychristin AREA:N/A S/N:N/A

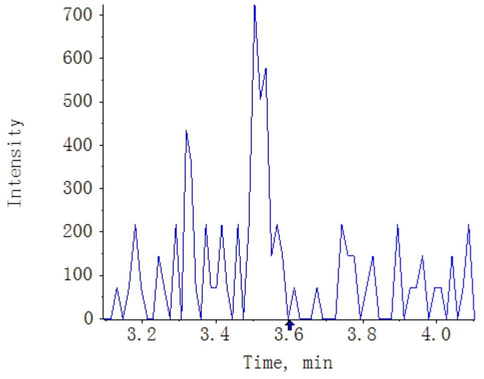

A21233253b_b
Silychristin AREA:N/A S/N:N/A

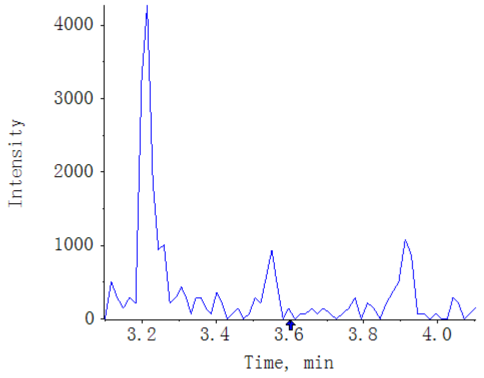

A21233254b_b
Silychristin AREA:8.69e3 S/N:21.0

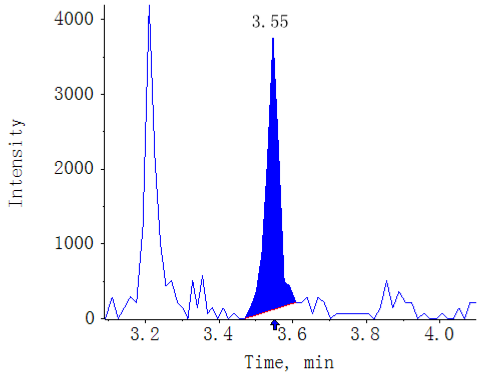

A21233255b_b
Silychristin AREA:6.59e3 S/N:19.3

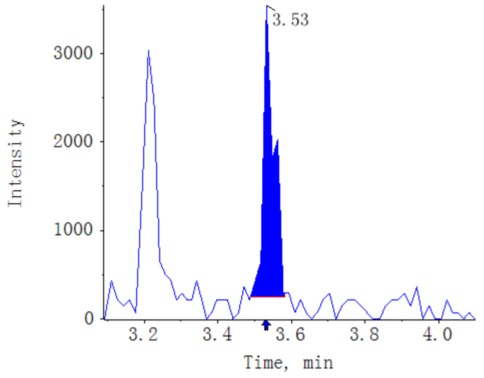

A21233256b_b
Silychristin AREA:1.16e4 S/N:16.1

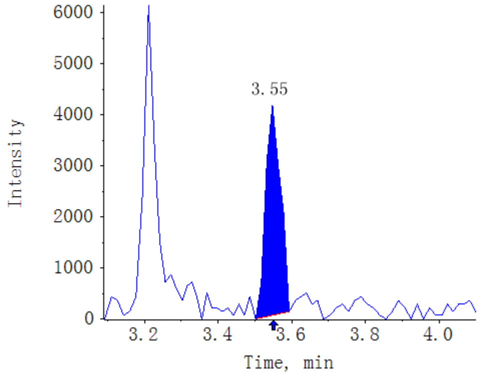

A21233257b_b
Silychristin AREA:1.71e4 S/N:31.7

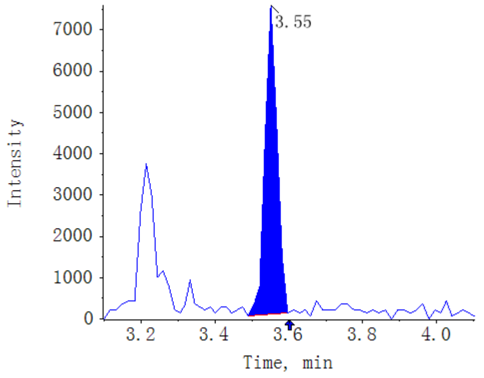

A21233258b_b
Silychristin AREA:7.15e3 S/N:14.3

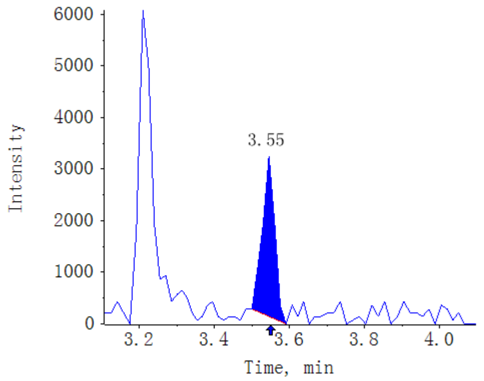

A21233259b_b
Silychristin AREA:N/A S/N:N/A

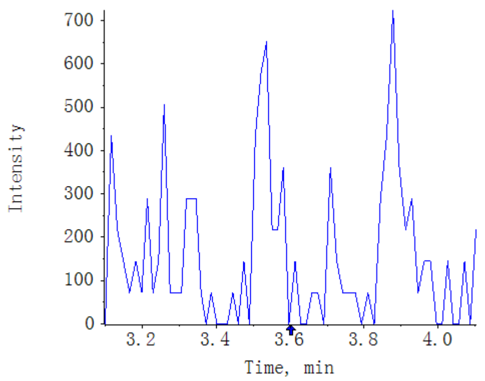

A21233260b_b
Silychristin AREA:N/A S/N:N/A

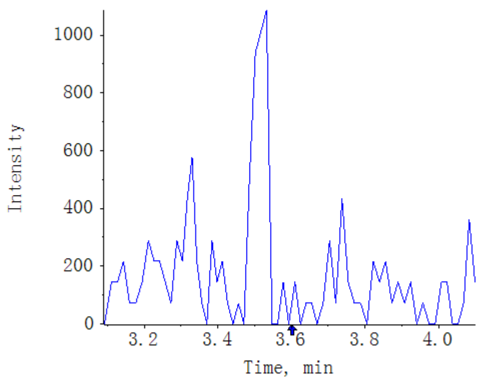

A21233261b_b
Silychristin AREA:3.76e3 S/N:11.4

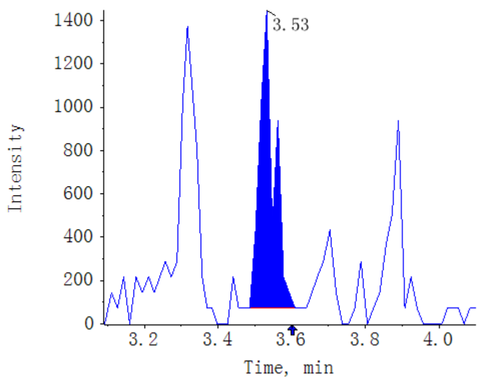

A21233262b_b
Silychristin AREA:3.39e3 S/N:8.6

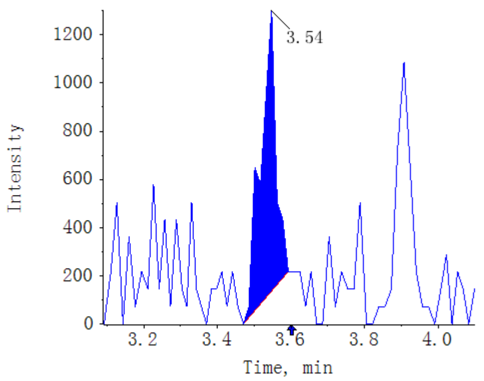

A21233263b_b
Silychristin AREA:N/A S/N:N/A

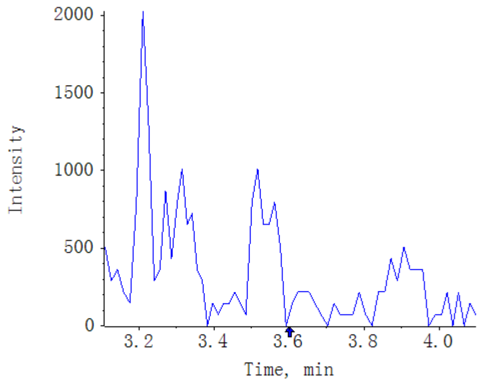

A21233264b_b
Silychristin AREA:N/A S/N:N/A

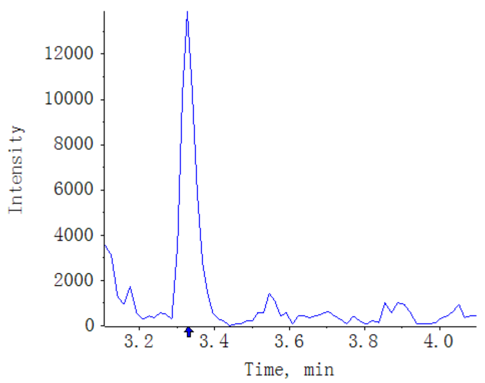

A21233265b_b
Silychristin AREA:1.33e4 S/N:16.6

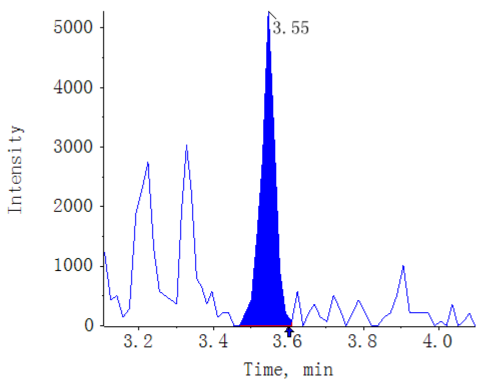

A21233266b_b
Silychristin AREA:5.08e3 S/N:10.2

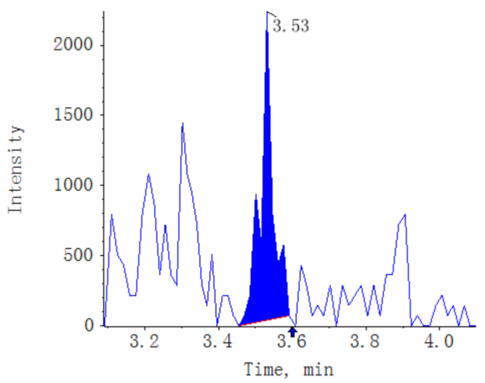

A21233267b_b
Silychristin AREA:N/A S/N:N/A

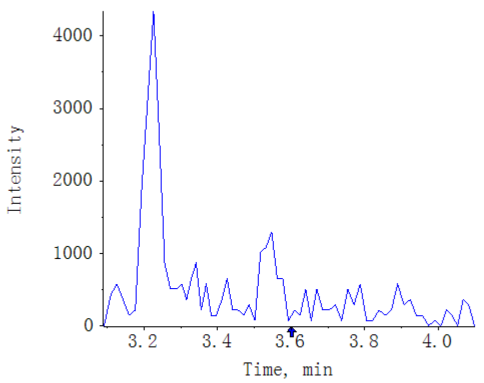

Result Table	MWXS-21-2101D-3_18_WH6500-5_A20-3_V1.0_TY_20211028	Algorithm Used	MQ4
Acquisition Method	Flavonoids_V1.0_WH6500-5_LT_20211025.dam	Instrument Name	QTRAP 6500+ Low Mass
Project	N/A	Analytes QTY	204:102

Compound name: Eriodictyol (287.1 / 135.0)

Sample Name	Sample Type	Area (cps)	Is Area (cps)	RT (min)	S/N	Target Conc	Calculated Conc.()
STD_0.5nM	Standard	2.16e3	N/A	4.06	8.7	0.5000	5.023487e-1
STD_1nM	Standard	3.32e3	N/A	4.04	14.9	1.0000	9.035317e-1
STD_5nM	Standard	2.09e4	N/A	4.05	64.7	5.0000	6.960468e0
STD_10nM	Standard	2.66e4	N/A	4.05	74.5	10.0000	8.937993e0
STD_20nM	Standard	5.42e4	N/A	4.05	165.8	20.0000	1.844424e1
STD_50nM	Standard	1.44e5	N/A	4.05	163.0	50.0000	4.937618e1
STD_100nM	Standard	2.97e5	N/A	4.05	209.3	100.0000	1.021183e2
STD_200nM	Standard	5.79e5	N/A	4.05	353.5	200.0000	1.992570e2
STD_500nM	Standard	N/A	N/A	N/A	N/A	500.0000	N/A
STD_1000nM	Standard	N/A	N/A	N/A	N/A	1000.0000	N/A
STD_2000nM	Standard	N/A	N/A	N/A	N/A	2000.0000	N/A
V1.0_MW_RQC1_20211018	Quality Control	1.93e5	N/A	4.04	122.8	0.0000	6.639319e1
Blank	Unknown	N/A	N/A	N/A	N/A	N/A	N/A
V1.0_MWMS_20211021_1	Unknown	4.45e5	N/A	4.06	221.1	N/A	1.529802e2
MWXS212101D3_R1	Quality Control	4.47e5	N/A	4.06	303.3	0.0000	1.536557e2
MWXS212101D3_R2	Quality Control	4.30e5	N/A	4.06	262.3	0.0000	1.477970e2
MWXS212101D3_R3	Quality Control	4.34e5	N/A	4.06	306.5	0.0000	1.494652e2
A21233250b_b	Unknown	5.64e3	N/A	4.06	35.4	N/A	1.702587e0
A21233251b_b	Unknown	8.49e3	N/A	4.06	38.6	N/A	2.685105e0
A21233252b_b	Unknown	7.29e3	N/A	4.06	36.9	N/A	2.271094e0
A21233253b_b	Unknown	1.38e6	N/A	4.06	77.5	N/A	4.742460e2
A21233254b_b	Unknown	1.48e6	N/A	4.06	110.9	N/A	5.111886e2
A21233255b_b	Unknown	3.05e6	N/A	4.06	95.7	N/A	1.051658e3
A21233256b_b	Unknown	1.87e6	N/A	4.06	127.0	N/A	6.450971e2
A21233257b_b	Unknown	2.90e6	N/A	4.06	138.9	N/A	1.000575e3
A21233258b_b	Unknown	9.13e5	N/A	4.06	189.8	N/A	3.142893e2
A21233259b_b	Unknown	4.50e4	N/A	4.06	90.4	N/A	1.528099e1
A21233260b_b	Unknown	4.37e3	N/A	4.07	20.3	N/A	1.266975e0
A21233261b_b	Unknown	4.90e4	N/A	4.06	113.0	N/A	1.664484e1
A21233262b_b	Unknown	5.01e5	N/A	4.06	289.7	N/A	1.724673e2
A21233263b_b	Unknown	4.51e6	N/A	4.05	116.8	N/A	1.554617e3
A21233264b_b	Unknown	3.74e6	N/A	4.06	110.1	N/A	1.286894e3
A21233265b_b	Unknown	5.50e6	N/A	4.06	168.6	N/A	1.894317e3
A21233266b_b	Unknown	3.32e6	N/A	4.05	110.4	N/A	1.142704e3
A21233267b_b	Unknown	5.24e6	N/A	4.07	142.6	N/A	1.806656e3

Compound name: Eriodictyol
Regression Equation: y = 2901.87645 x + 698.14968 (r = 0.99981) (weighting: None)

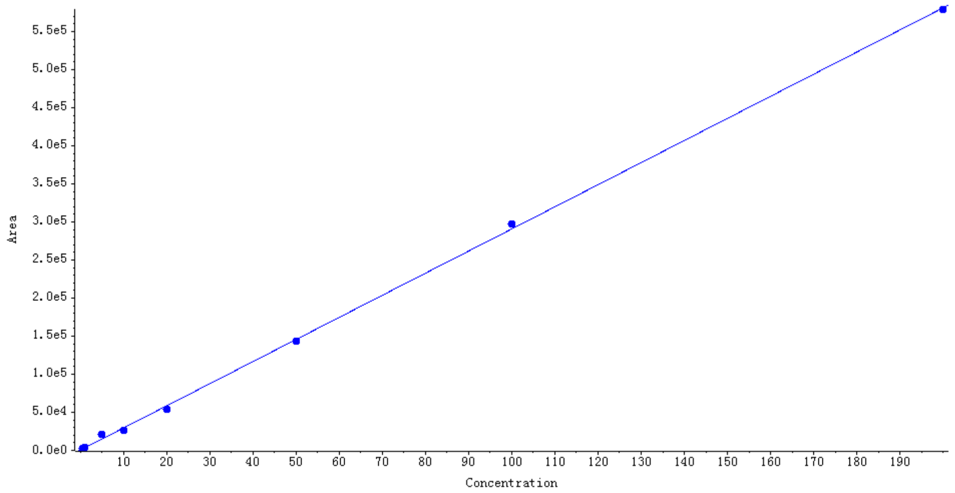

Peak Review

Blank
Eriodictyol AREA:N/A S/N:N/A

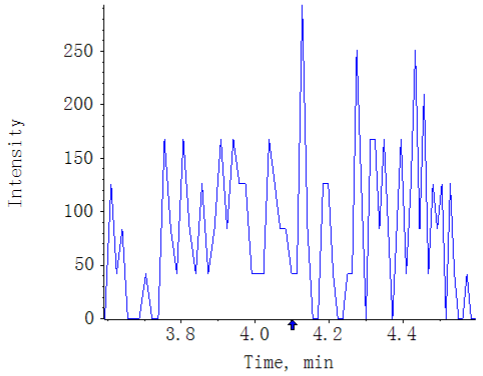

V1.0_MWMS_20211021_1
Eriodictyol AREA:4.45e5 S/N:221.1

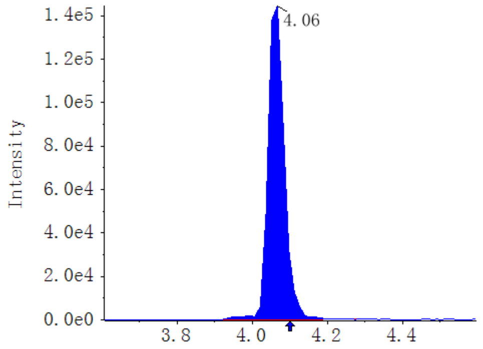

A21233250b_b
Eriodictyol AREA:5.64e3 S/N:35.4

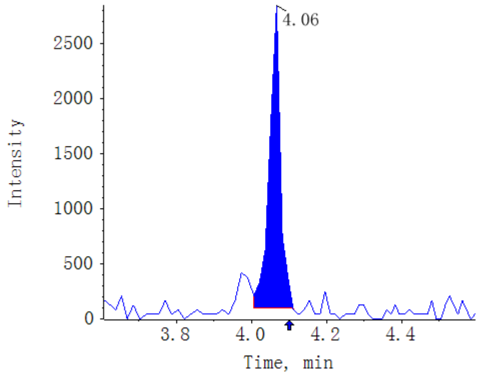

A21233251b_b
Eriodictyol AREA:8.49e3 S/N:38.6

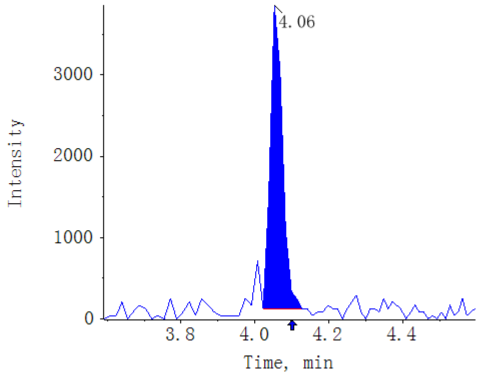

A21233252b_b
Eriodictyol AREA:7.29e3 S/N:36.9

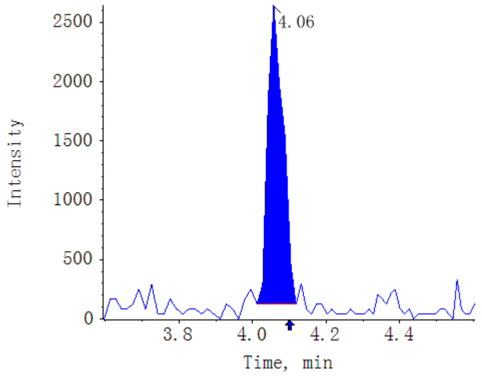

A21233253b_b
Eriodictyol AREA:1.38e6 S/N:77.5

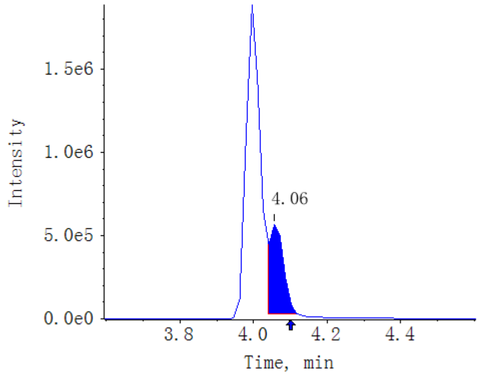

A21233254b_b
Eriodictyol AREA:1.48e6 S/N:110.9

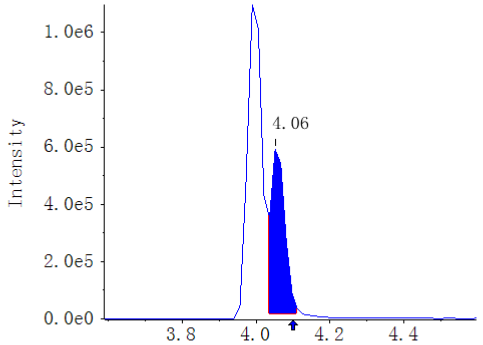

A21233255b_b
Eriodictyol AREA:3.05e6 S/N:95.7

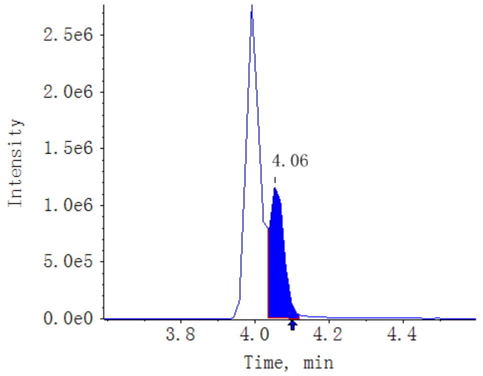

A21233256b_b
Eriodictyol AREA:1.87e6 S/N:127.0

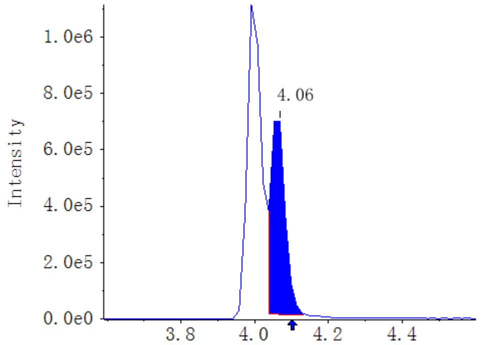

A21233257b_b
Eriodictyol AREA:2.90e6 S/N:138.9

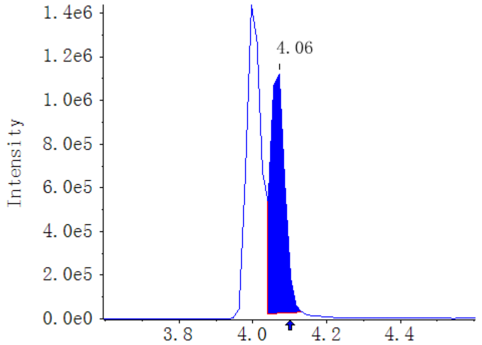

A21233258b_b
Eriodictyol AREA:9.13e5
S/N:189.8

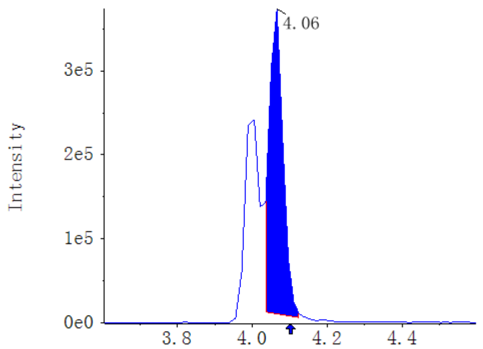

A21233259b_b
Eriodictyol AREA:4.50e4 S/N:90.4

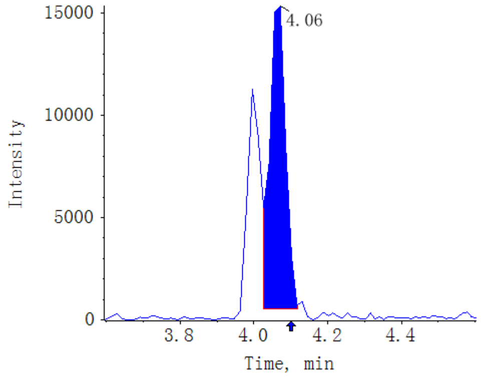

A21233260b_b
Eriodictyol AREA:4.37e3 S/N:20.3

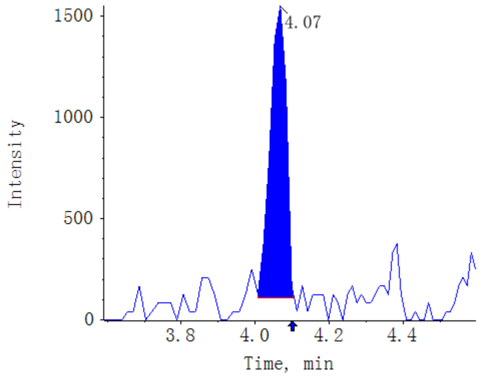

A21233261b_b
Eriodictyol AREA:4.90e4
S/N:113.0

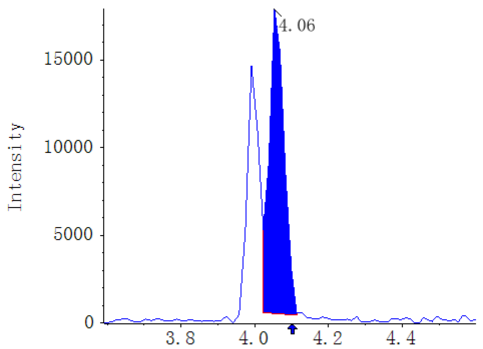

A21233262b_b
Eriodictyol AREA:5.01e5
S/N:289.7

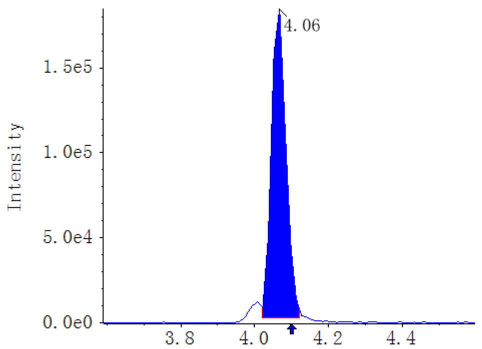

A21233263b_b
Eriodictyol AREA:4.51e6
S/N:116.8

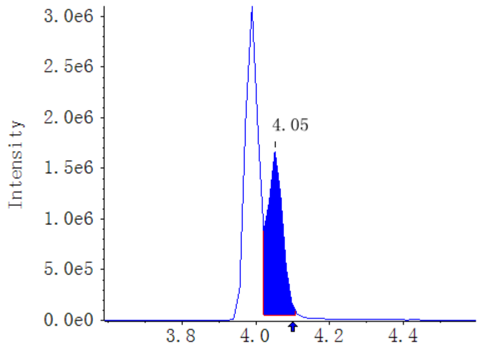

A21233264b_b
Eriodictyol AREA:3.74e6
S/N:110.1

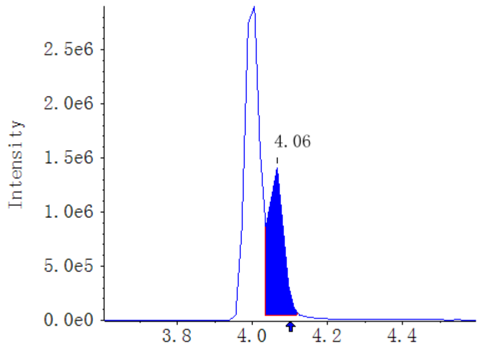

A21233265b_b
Eriodictyol AREA:5.50e6
S/N:168.6

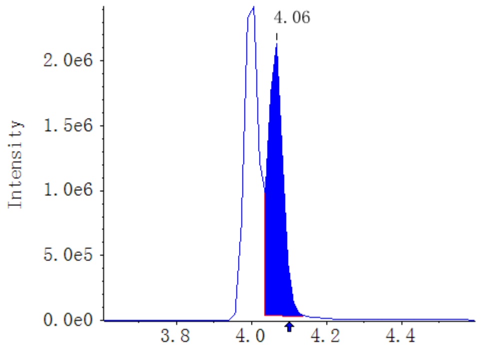

A21233266b_b
Eriodictyol AREA:3.32e6
S/N:110.4

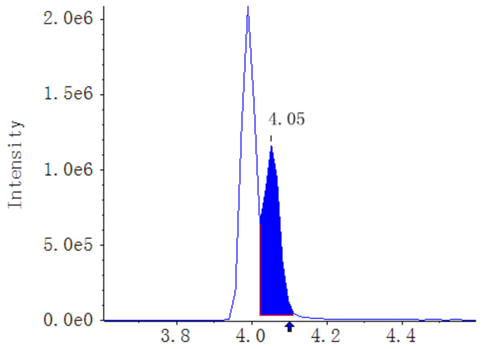

A21233267b_b
Eriodictyol AREA:5.24e6
S/N:142.6

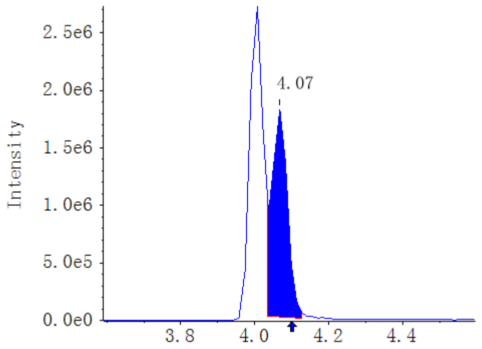

Result Table	MWXS-21-2101D-3_18_WH6500-5_A20-3_V1.0_TY_20211028	Algorithm Used	MQ4
Acquisition Method	Flavonoids_V1.0_WH6500-5_LT_20211025.dam	Instrument Name	QTRAP 6500+ Low Mass
Project	N/A	Analytes QTY	204:109

Compound name: Theaflavin 3,3'-digallate (867.2 / 697.1)

Sample Name	Sample Type	Area (cps)	Is Area (cps)	RT (min)	S/N	Target Conc	Calculated Conc.()
STD_0.5nM	Standard	N/A	N/A	N/A	N/A	0.5000	N/A
STD_1nM	Standard	N/A	N/A	N/A	N/A	1.0000	N/A
STD_5nM	Standard	N/A	N/A	N/A	N/A	5.0000	N/A
STD_10nM	Standard	7.88e2	N/A	3.79	10.1	10.0000	1.248717e1
STD_20nM	Standard	1.26e3	N/A	3.76	21.6	20.0000	1.556886e1
STD_50nM	Standard	5.88e3	N/A	3.78	47.6	50.0000	4.549660e1
STD_100nM	Standard	1.52e4	N/A	3.78	67.5	100.0000	1.061341e2
STD_200nM	Standard	2.98e4	N/A	3.77	97.3	200.0000	2.003133e2
STD_500nM	Standard	N/A	N/A	N/A	N/A	500.0000	N/A
STD_1000nM	Standard	N/A	N/A	N/A	N/A	1000.0000	N/A
STD_2000nM	Standard	N/A	N/A	N/A	N/A	2000.0000	N/A
V1.0_MW_RQC1_20211018	Quality Control	N/A	N/A	N/A	N/A	0.0000	N/A
Blank	Unknown	N/A	N/A	N/A	N/A	N/A	N/A
V1.0_MWMS_20211021_1	Unknown	4.87e4	N/A	3.79	158.4	N/A	3.228268e2
MWXS212101D3_R1	Quality Control	4.94e4	N/A	3.79	128.8	0.0000	3.279544e2
MWXS212101D3_R2	Quality Control	5.14e4	N/A	3.79	159.0	0.0000	3.407413e2
MWXS212101D3_R3	Quality Control	5.27e4	N/A	3.79	141.1	0.0000	3.492442e2
A21233250b_b	Unknown	N/A	N/A	N/A	N/A	N/A	N/A
A21233251b_b	Unknown	N/A	N/A	N/A	N/A	N/A	N/A
A21233252b_b	Unknown	N/A	N/A	N/A	N/A	N/A	N/A
A21233253b_b	Unknown	-2.61e1	N/A	3.70	N/A	N/A	7.208013e0
A21233254b_b	Unknown	N/A	N/A	N/A	N/A	N/A	N/A
A21233255b_b	Unknown	N/A	N/A	N/A	N/A	N/A	N/A
A21233256b_b	Unknown	N/A	N/A	N/A	N/A	N/A	N/A
A21233257b_b	Unknown	N/A	N/A	N/A	N/A	N/A	N/A
A21233258b_b	Unknown	N/A	N/A	N/A	N/A	N/A	N/A
A21233259b_b	Unknown	N/A	N/A	N/A	N/A	N/A	N/A
A21233260b_b	Unknown	N/A	N/A	N/A	N/A	N/A	N/A
A21233261b_b	Unknown	N/A	N/A	N/A	N/A	N/A	N/A
A21233262b_b	Unknown	N/A	N/A	N/A	N/A	N/A	N/A
A21233263b_b	Unknown	N/A	N/A	N/A	N/A	N/A	N/A
A21233264b_b	Unknown	N/A	N/A	N/A	N/A	N/A	N/A
A21233265b_b	Unknown	N/A	N/A	N/A	N/A	N/A	N/A
A21233266b_b	Unknown	N/A	N/A	N/A	N/A	N/A	N/A
A21233267b_b	Unknown	N/A	N/A	N/A	N/A	N/A	N/A

Compound name: Theaflavin 3,3'-digallate
Regression Equation: y = 154.23305 x + -1137.78655 (r = 0.99517) (weighting: 1 / x)

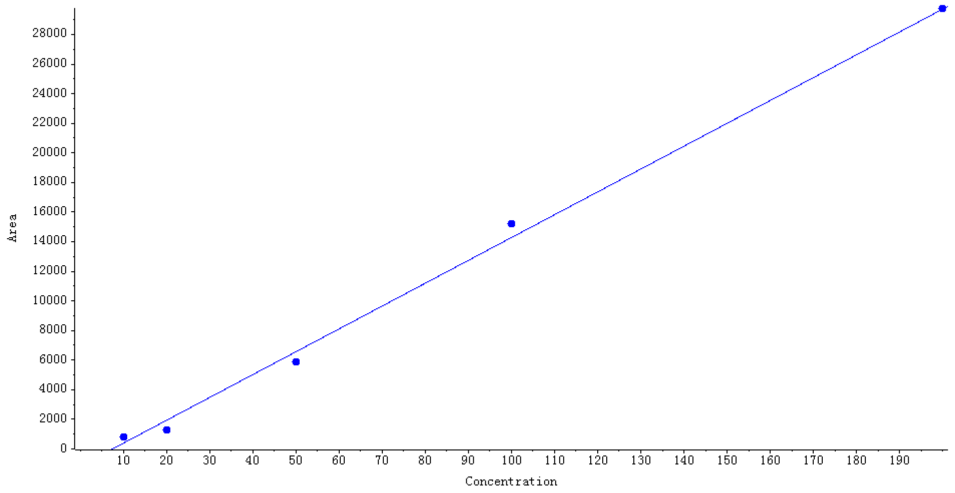

Peak Review

Blank
Theaflavin 3,3'-digallate AREA:N/A
S/N:N/A

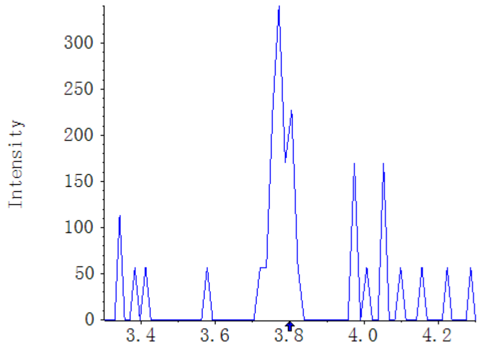

V1.0_MWMS_20211021_1
Theaflavin 3,3'-digallate
AREA:4.87e4 S/N:158.4

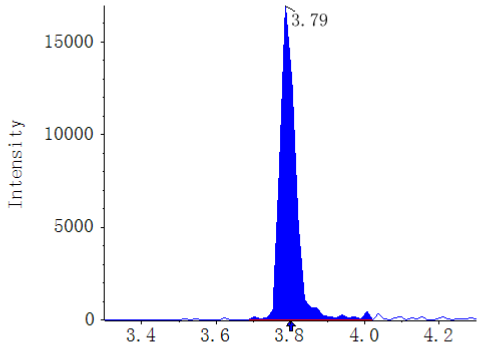

A21233250b_b
Theaflavin 3,3'-digallate AREA:N/A
S/N:N/A

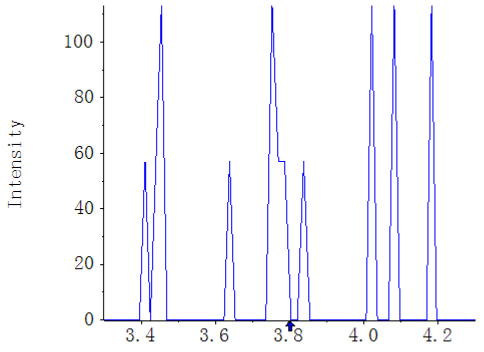

A21233251b_b
Theaflavin 3,3'-digallate AREA:N/A
S/N:N/A

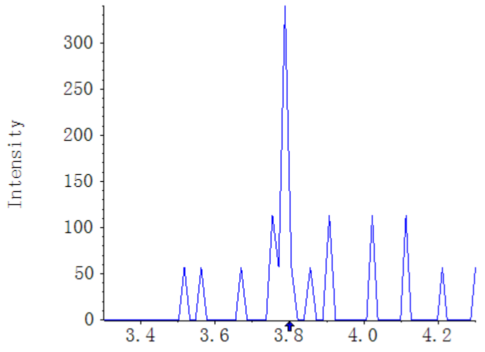

A21233252b_b
Theaflavin 3,3'-digallate AREA:N/A
S/N:N/A

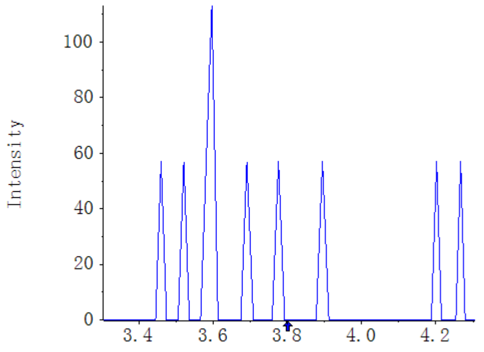

A21233253b_b
Theaflavin 3,3'-digallate AREA:-
2.61e1 S/N:N/A

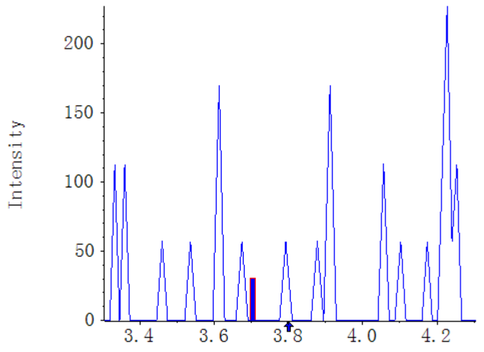

A21233254b_b
Theaflavin 3,3'-digallate AREA:N/A
S/N:N/A

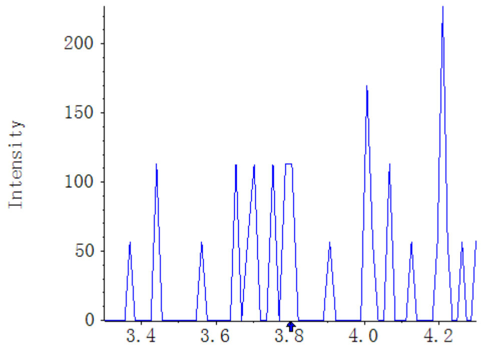

A21233255b_b
Theaflavin 3,3'-digallate AREA:N/A
S/N:N/A

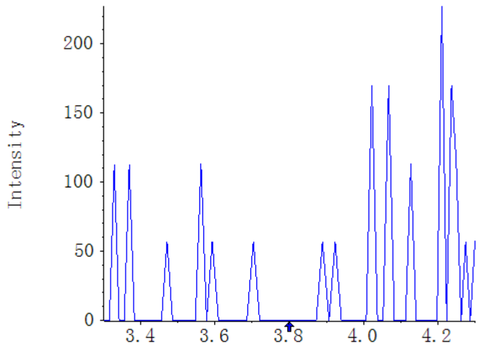

A21233256b_b
Theaflavin 3,3'-digallate AREA:N/A
S/N:N/A

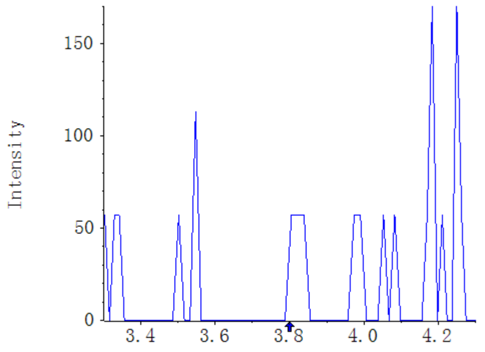

A21233257b_b
Theaflavin 3,3'-digallate AREA:N/A
S/N:N/A

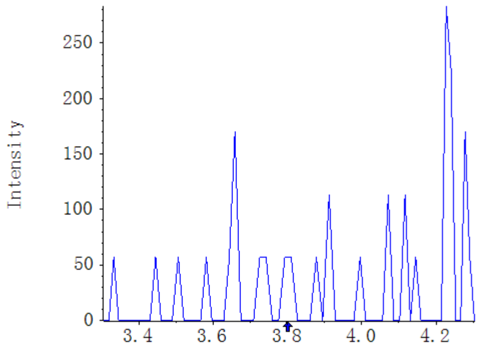

A21233258b_b
Theaflavin 3,3'-digallate AREA:N/A
S/N:N/A

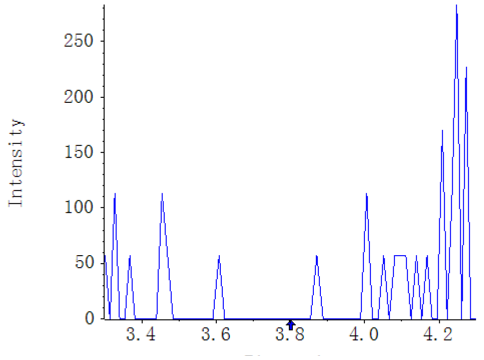

A21233259b_b
Theaflavin 3,3'-digallate AREA:N/A
S/N:N/A

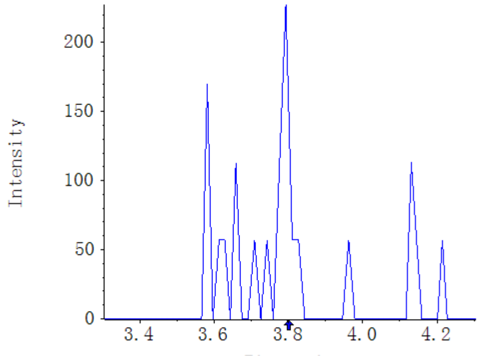

A21233260b_b
Theaflavin 3,3'-digallate AREA:N/A
S/N:N/A

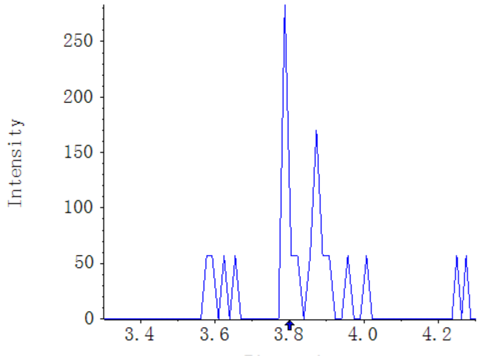

A21233261b_b
Theaflavin 3,3'-digallate AREA:N/A
S/N:N/A

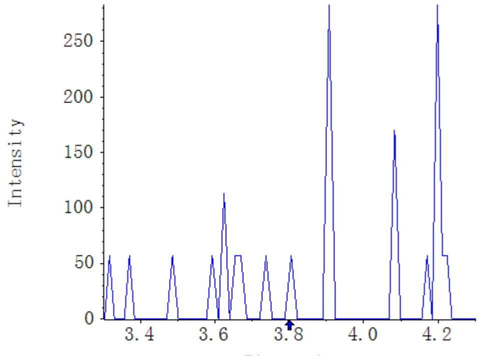

A21233262b_b
Theaflavin 3,3'-digallate AREA:N/A
S/N:N/A

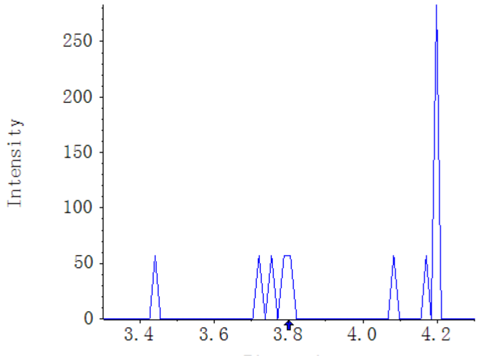

A21233263b_b
Theaflavin 3,3'-digallate AREA:N/A
S/N:N/A

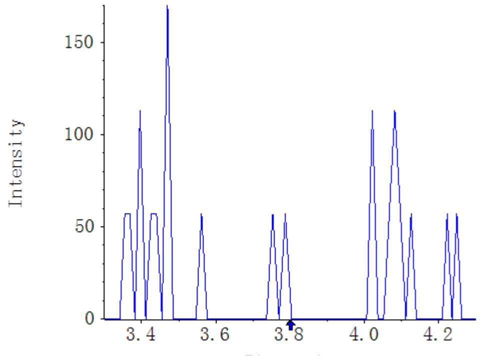

A21233264b_b
Theaflavin 3,3'-digallate AREA:N/A
S/N:N/A

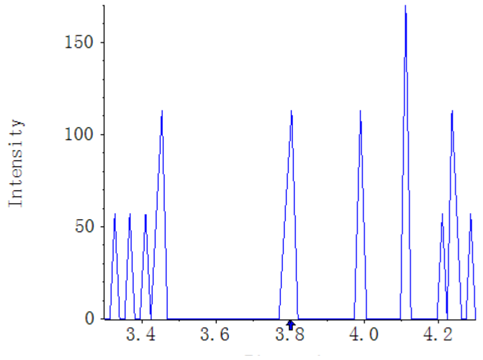

A21233265b_b
Theaflavin 3,3'-digallate AREA:N/A
S/N:N/A

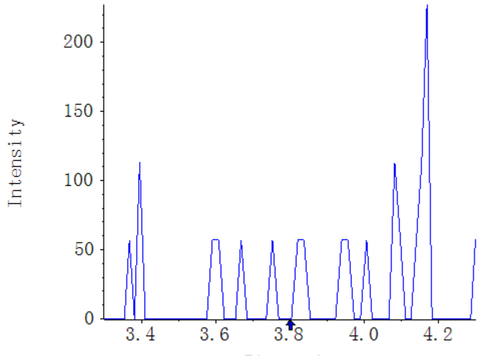

A21233266b_b
Theaflavin 3,3'-digallate AREA:N/A
S/N:N/A

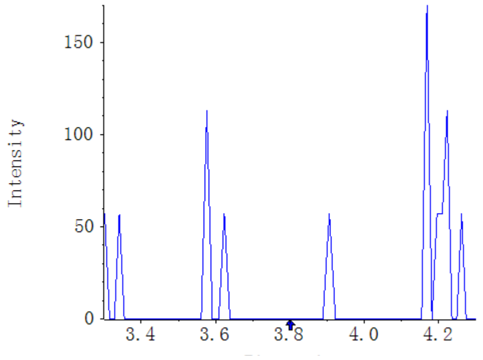

A21233267b_b
Theaflavin 3,3'-digallate AREA:N/A
S/N:N/A

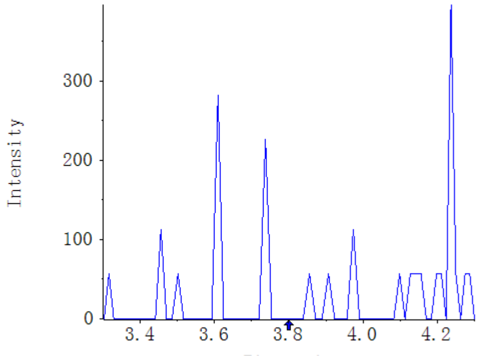

**Result Table**	MWXS-21-2101D-3_18_WH6500-5_A20-3_V1.0_TY_20211028	**Algorithm Used**	MQ4
**Acquisition Method**	Flavonoids_V1.0_WH6500-5_LT_20211025.dam	**Instrument Name**	QTRAP 6500+ Low Mass
**Project**	N/A	**Analytes QTY**	204:110

Compound name: 6,2'-Dihydroxyflavone (253.1 / 117.0)

Sample Name	Sample Type	Area (cps)	Is Area (cps)	RT (min)	S/N	Target Conc	Calculated Conc.()
STD_0.5nM	Standard	4.86e3	N/A	4.69	5.8	0.5000	3.480938e-1
STD_1nM	Standard	1.10e4	N/A	4.68	11.4	1.0000	1.077177e0
STD_5nM	Standard	5.38e4	N/A	4.68	50.5	5.0000	6.197314e0
STD_10nM	Standard	8.45e4	N/A	4.68	62.8	10.0000	9.874910e0
STD_20nM	Standard	1.67e5	N/A	4.68	85.8	20.0000	1.971358e1
STD_50nM	Standard	4.37e5	N/A	4.69	176.7	50.0000	5.200193e1
STD_100nM	Standard	8.17e5	N/A	4.69	266.4	100.0000	9.750578e1
STD_200nM	Standard	1.67e6	N/A	4.68	267.0	200.0000	1.997812e2
STD_500nM	Standard	N/A	N/A	N/A	N/A	500.0000	N/A
STD_1000nM	Standard	N/A	N/A	N/A	N/A	1000.0000	N/A
STD_2000nM	Standard	N/A	N/A	N/A	N/A	2000.0000	N/A
V1.0_MW_RQC1_20211018	Quality Control	N/A	N/A	N/A	N/A	0.0000	N/A
Blank	Unknown	N/A	N/A	N/A	N/A	N/A	N/A
V1.0_MWMS_20211021_1	Unknown	1.35e6	N/A	4.70	338.3	N/A	1.607538e2
MWXS212101D3_R1	Quality Control	1.30e6	N/A	4.69	311.2	0.0000	1.550935e2
MWXS212101D3_R2	Quality Control	1.30e6	N/A	4.70	252.1	0.0000	1.552890e2
MWXS212101D3_R3	Quality Control	1.34e6	N/A	4.69	257.0	0.0000	1.599191e2
A21233250b_b	Unknown	N/A	N/A	N/A	N/A	N/A	N/A
A21233251b_b	Unknown	N/A	N/A	N/A	N/A	N/A	N/A
A21233252b_b	Unknown	N/A	N/A	N/A	N/A	N/A	N/A
A21233253b_b	Unknown	2.61e4	N/A	4.71	30.7	N/A	2.886807e0
A21233254b_b	Unknown	2.56e4	N/A	4.70	33.4	N/A	2.828131e0
A21233255b_b	Unknown	2.81e4	N/A	4.70	36.8	N/A	3.127482e0
A21233256b_b	Unknown	1.38e4	N/A	4.71	25.3	N/A	1.414361e0
A21233257b_b	Unknown	1.53e4	N/A	4.71	21.3	N/A	1.600322e0
A21233258b_b	Unknown	5.52e3	N/A	4.71	11.3	N/A	4.277561e-1
A21233259b_b	Unknown	N/A	N/A	N/A	N/A	N/A	N/A
A21233260b_b	Unknown	N/A	N/A	N/A	N/A	N/A	N/A
A21233261b_b	Unknown	N/A	N/A	N/A	N/A	N/A	N/A
A21233262b_b	Unknown	N/A	N/A	N/A	N/A	N/A	N/A
A21233263b_b	Unknown	2.85e4	N/A	4.70	29.7	N/A	3.173571e0
A21233264b_b	Unknown	3.30e4	N/A	4.71	43.4	N/A	3.718846e0
A21233265b_b	Unknown	2.27e4	N/A	4.70	31.0	N/A	2.484993e0
A21233266b_b	Unknown	2.41e4	N/A	4.70	29.2	N/A	2.644343e0
A21233267b_b	Unknown	1.70e4	N/A	4.71	23.8	N/A	1.802653e0

Compound name: 6,2'-Dihydroxyflavone
Regression Equation: y = 8363.60692 x + 1943.87321 (r = 0.99934) (weighting: 1 / x)

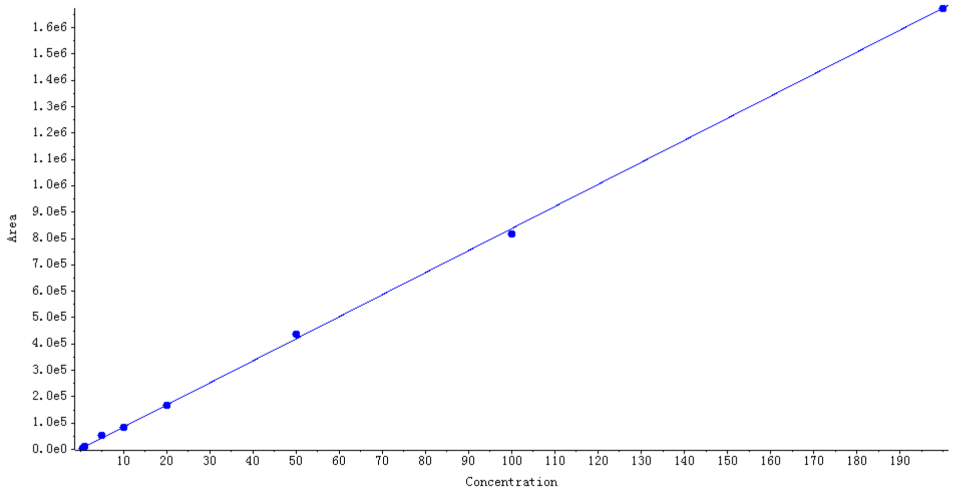

Peak Review

Blank
6,2'-Dihydroxyflavone AREA:N/A
S/N:N/A

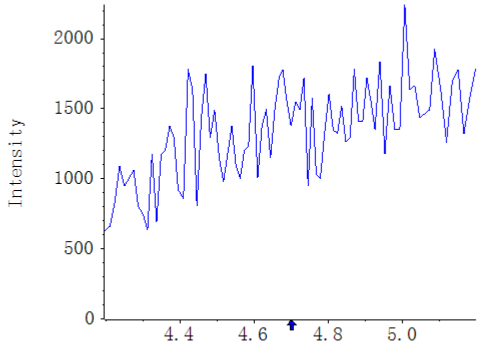

V1.0_MWMS_20211021_1
6,2'-Dihydroxyflavone AREA:1.35e6
S/N:338.3

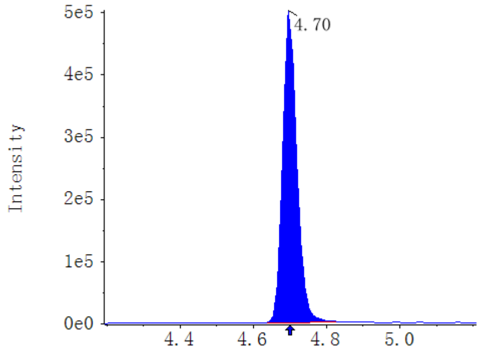

A21233250b_b
6,2'-Dihydroxyflavone AREA:N/A
S/N:N/A

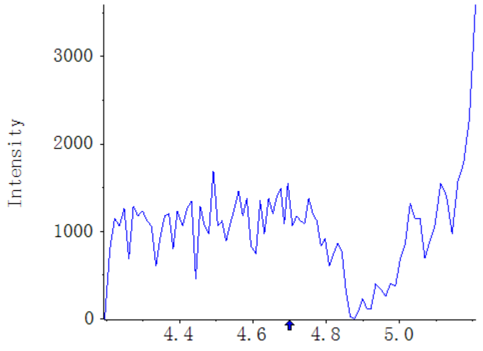

A21233251b_b
6,2'-Dihydroxyflavone AREA:N/A
S/N:N/A

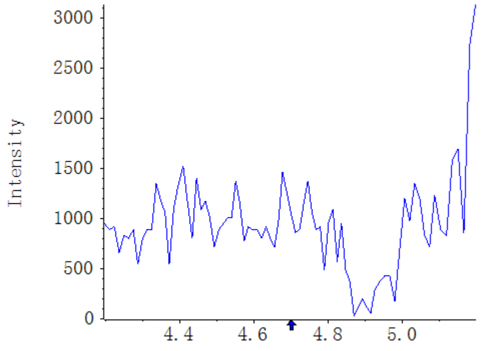

A21233252b_b
6,2'-Dihydroxyflavone AREA:N/A
S/N:N/A

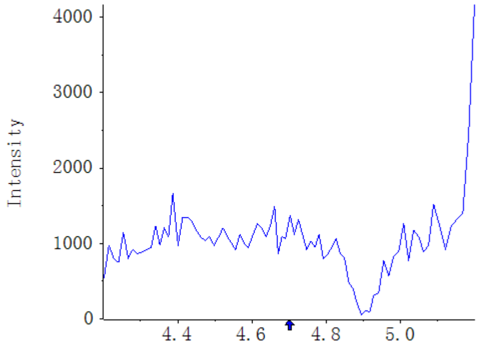

A21233253b_b
6,2'-Dihydroxyflavone AREA:2.61e4
S/N:30.7

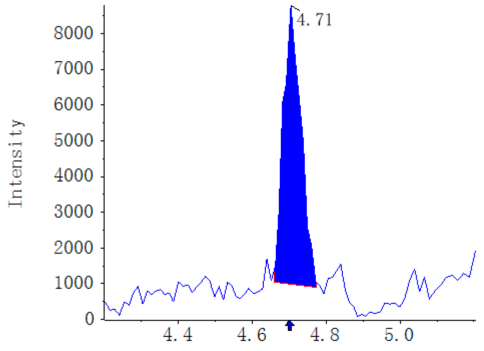

A21233254b_b
6,2'-Dihydroxyflavone AREA:2.56e4
S/N:33.4

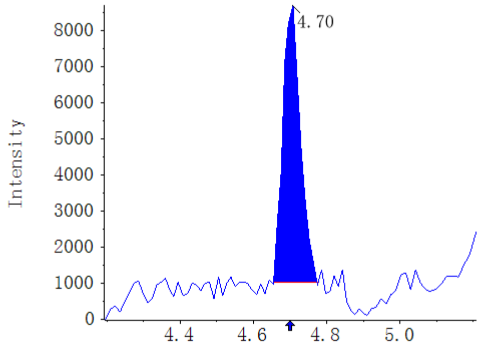

A21233255b_b
6,2'-Dihydroxyflavone AREA:2.81e4
S/N:36.8

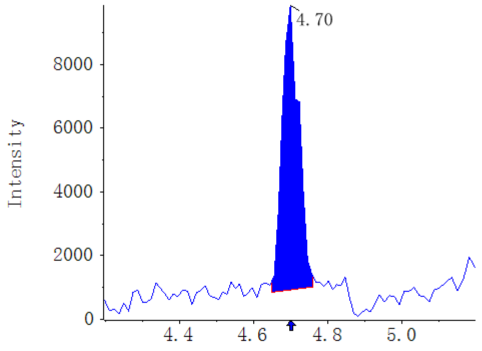

A21233256b_b
6,2'-Dihydroxyflavone AREA:1.38e4
S/N:25.3

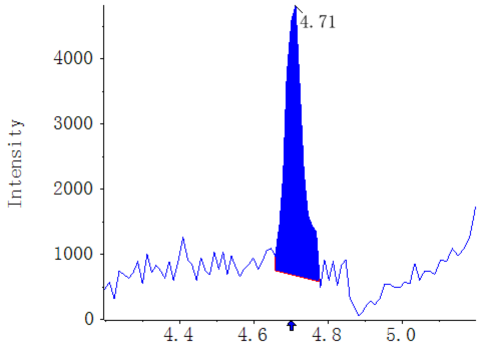

A21233257b_b
6,2'-Dihydroxyflavone AREA:1.53e4
S/N:21.3

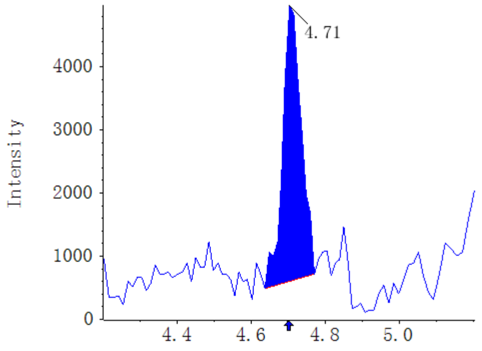

A21233258b_b
6,2'-Dihydroxyflavone AREA:5.52e3
S/N:11.3

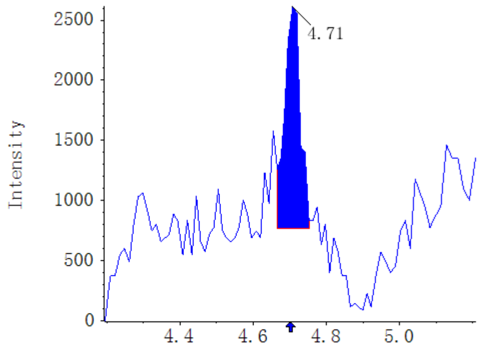

A21233259b_b
6,2'-Dihydroxyflavone AREA:N/A
S/N:N/A

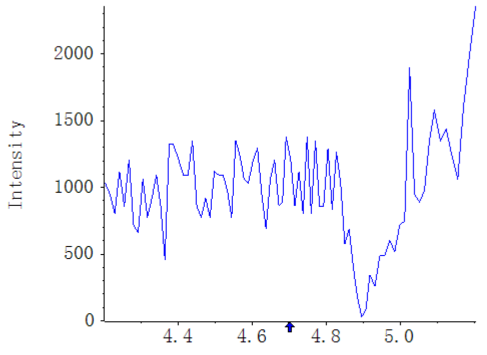

A21233260b_b
6,2'-Dihydroxyflavone AREA:N/A
S/N:N/A

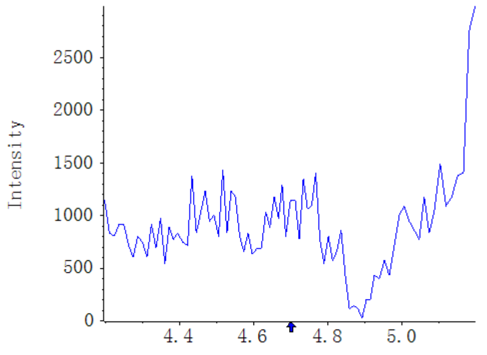

A21233261b_b
6,2'-Dihydroxyflavone AREA:N/A
S/N:N/A

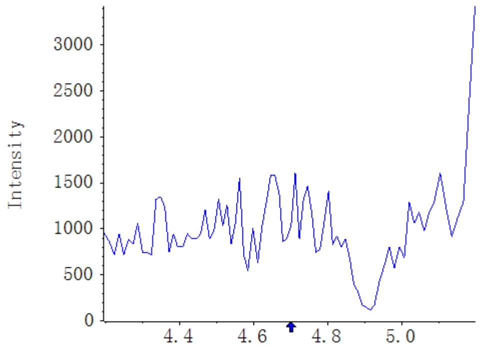

A21233262b_b
6,2'-Dihydroxyflavone AREA:N/A
S/N:N/A

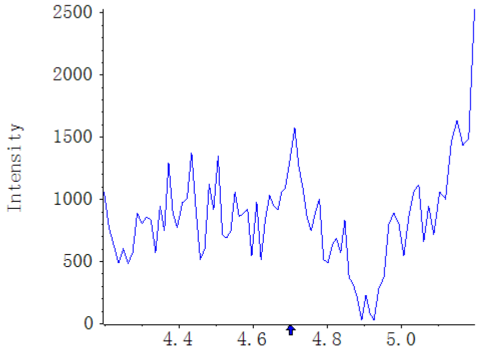

A21233263b_b
6,2'-Dihydroxyflavone AREA:2.85e4
S/N:29.7

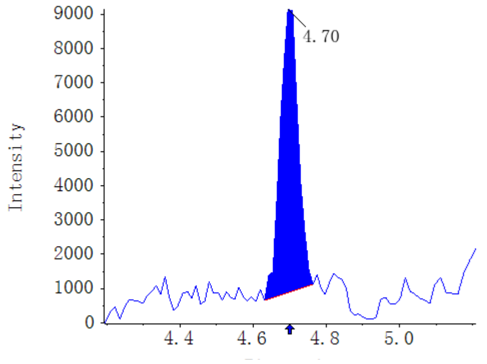

A21233264b_b
6,2'-Dihydroxyflavone AREA:3.30e4
S/N:43.4

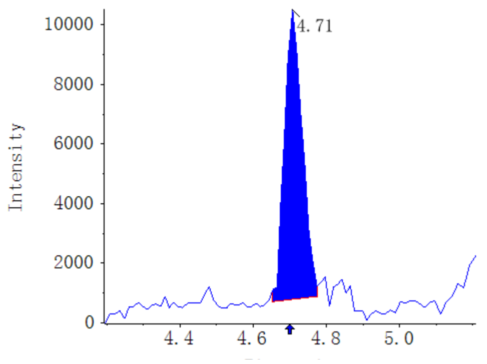

A21233265b_b
6,2'-Dihydroxyflavone AREA:2.27e4
S/N:31.0

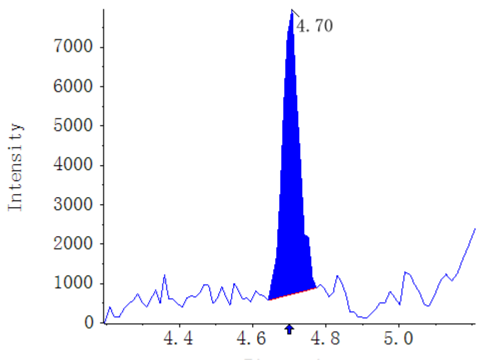

A21233266b_b
6,2'-Dihydroxyflavone AREA:2.41e4
S/N:29.2

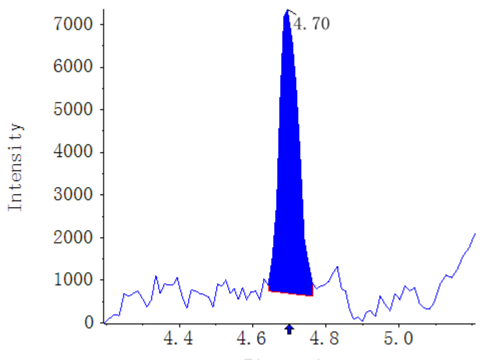

A21233267b_b
6,2'-Dihydroxyflavone AREA:1.70e4
S/N:23.8

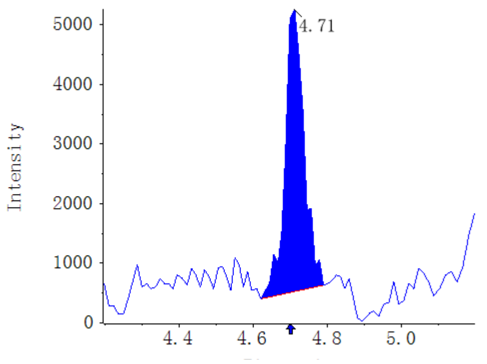

Result Table	MWXS-21-2101D-3_18_WH6500-5_A20-3_V1.0_TY_20211028	Algorithm Used	MQ4
Acquisition Method	Flavonoids_V1.0_WH6500-5_LT_20211025.dam	Instrument Name	QTRAP 6500+ Low Mass
Project	N/A	Analytes QTY	204:112

Compound name: Baimaside (625.1 / 300.0)

Sample Name	Sample Type	Area (cps)	Is Area (cps)	RT (min)	S/N	Target Conc	Calculated Conc.()
STD_0.5nM	Standard	3.06e3	N/A	2.41	19.4	0.5000	3.314550e-1
STD_1nM	Standard	7.85e3	N/A	2.40	29.1	1.0000	9.336469e-1
STD_5nM	Standard	5.92e4	N/A	2.40	196.7	5.0000	7.375877e0
STD_10nM	Standard	7.57e4	N/A	2.40	242.4	10.0000	9.458774e0
STD_20nM	Standard	1.53e5	N/A	2.40	245.7	20.0000	1.922136e1
STD_50nM	Standard	4.15e5	N/A	2.40	223.6	50.0000	5.204684e1
STD_100nM	Standard	7.88e5	N/A	2.41	257.7	100.0000	9.894509e1
STD_200nM	Standard	1.58e6	N/A	2.40	272.9	200.0000	1.981870e2
STD_500nM	Standard	N/A	N/A	N/A	N/A	500.0000	N/A
STD_1000nM	Standard	N/A	N/A	N/A	N/A	1000.0000	N/A
STD_2000nM	Standard	N/A	N/A	N/A	N/A	2000.0000	N/A
V1.0_MW_RQC1_20211018	Quality Control	N/A	N/A	N/A	N/A	0.0000	N/A
Blank	Unknown	N/A	N/A	N/A	N/A	N/A	N/A
V1.0_MWMS_20211021_1	Unknown	1.35e6	N/A	2.41	270.4	N/A	1.699094e2
MWXS212101D3_R1	Quality Control	1.43e6	N/A	2.40	295.0	0.0000	1.791701e2
MWXS212101D3_R2	Quality Control	1.41e6	N/A	2.41	292.8	0.0000	1.775556e2
MWXS212101D3_R3	Quality Control	1.37e6	N/A	2.40	289.8	0.0000	1.723093e2
A21233250b_b	Unknown	8.97e5	N/A	2.40	217.6	N/A	1.125780e2
A21233251b_b	Unknown	6.27e3	N/A	2.40	10.6	N/A	7.344108e-1
A21233252b_b	Unknown	4.81e3	N/A	2.40	9.7	N/A	5.509163e-1
A21233253b_b	Unknown	5.39e5	N/A	2.41	168.1	N/A	6.765729e1
A21233254b_b	Unknown	2.15e5	N/A	2.40	155.4	N/A	2.698091e1
A21233255b_b	Unknown	2.35e3	N/A	2.41	4.9	N/A	2.426046e-1
A21233256b_b	Unknown	3.66e3	N/A	2.40	4.4	N/A	4.065616e-1
A21233257b_b	Unknown	4.03e3	N/A	2.40	6.0	N/A	4.534945e-1
A21233258b_b	Unknown	2.38e3	N/A	2.40	3.2	N/A	2.456955e-1
A21233259b_b	Unknown	9.46e4	N/A	2.41	94.6	N/A	1.182572e1
A21233260b_b	Unknown	1.13e4	N/A	2.39	21.9	N/A	1.370307e0
A21233261b_b	Unknown	6.88e4	N/A	2.41	71.7	N/A	8.582343e0
A21233262b_b	Unknown	9.22e4	N/A	2.41	111.5	N/A	1.153048e1
A21233263b_b	Unknown	1.45e4	N/A	2.40	18.6	N/A	1.772319e0
A21233264b_b	Unknown	4.87e3	N/A	2.40	8.8	N/A	5.593477e-1
A21233265b_b	Unknown	2.41e3	N/A	2.41	6.0	N/A	2.502649e-1
A21233266b_b	Unknown	3.01e4	N/A	2.40	39.0	N/A	3.723679e0
A21233267b_b	Unknown	2.59e3	N/A	2.40	4.6	N/A	2.732563e-1

Compound name: Baimaside
Regression Equation: y = 7962.77841 x + 418.84858 (r = 0.99815) (weighting: 1 / x)

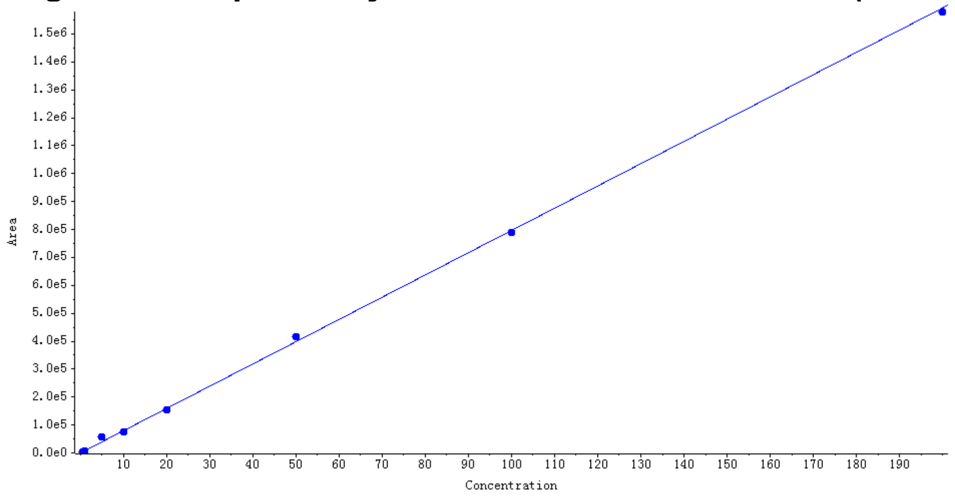

Peak Review

Blank
Baimaside AREA:N/A S/N:N/A

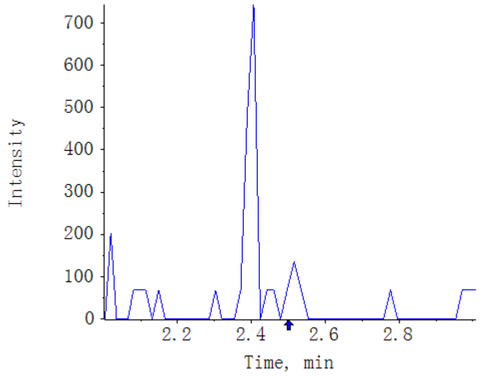

V1.0_MWMS_20211021_1
Baimaside AREA:1.35e6 S/N:270.4

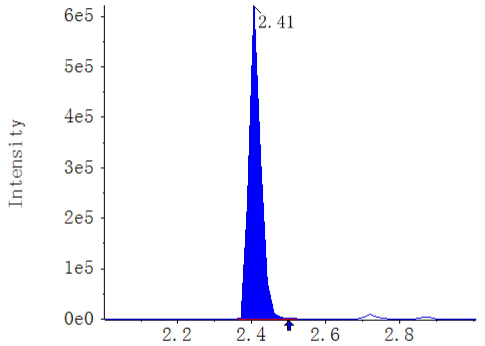

A21233250b_b
Baimaside AREA:8.97e5 S/N:217.6

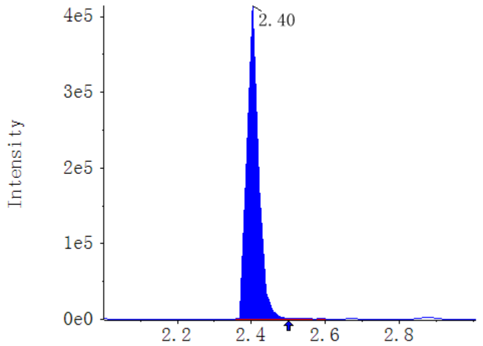

A21233251b_b
Baimaside AREA:6.27e3 S/N:10.6

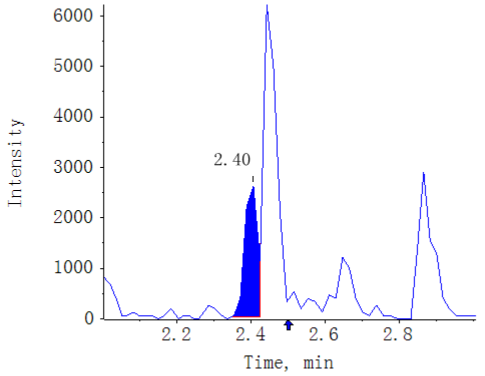

A21233252b_b
Baimaside AREA:4.81e3 S/N:9.7

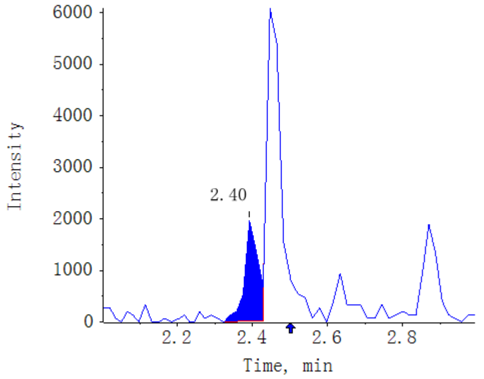

A21233253b_b
Baimaside AREA:5.39e5 S/N:168.1

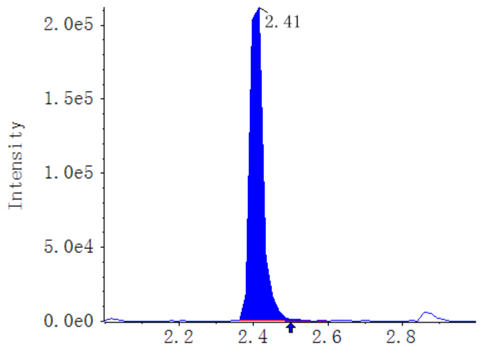

A21233254b_b
Baimaside AREA:2.15e5 S/N:155.4

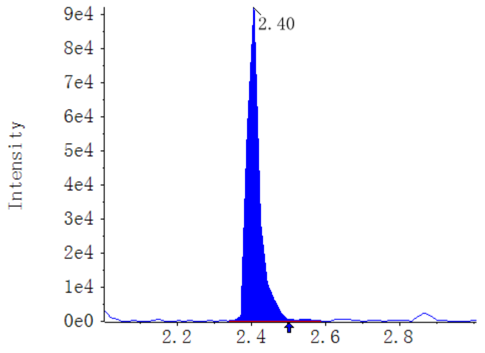

A21233255b_b
Baimaside AREA:2.35e3 S/N:4.9

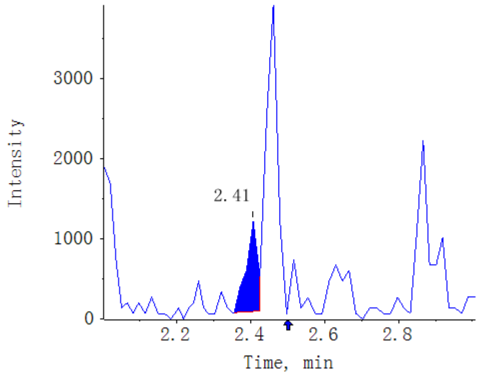

A21233256b_b
Baimaside AREA:3.66e3 S/N:4.4

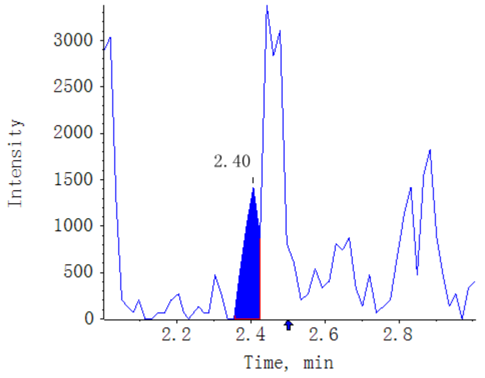

A21233257b_b
Baimaside AREA:4.03e3 S/N:6.0

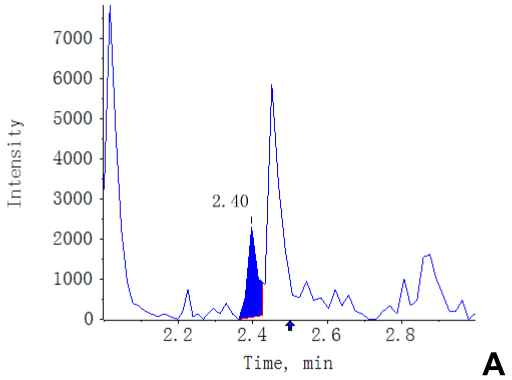

A

[illegible]

A21233258b_b
Baimaside AREA:2.38e3 S/N:3.2

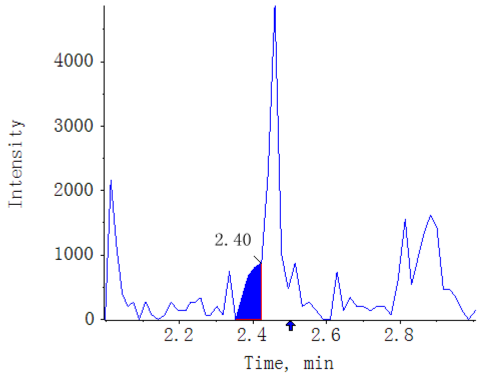

A21233259b_b
Baimaside AREA:9.46e4 S/N:94.6

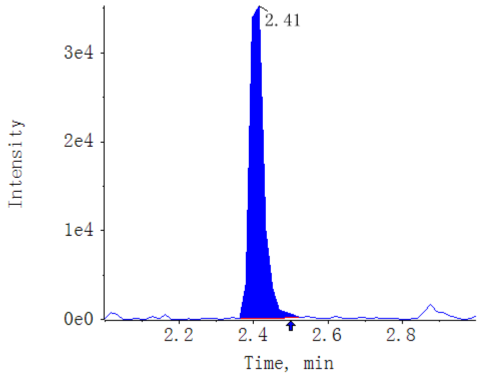

A21233260b_b
Baimaside AREA:1.13e4 S/N:21.9

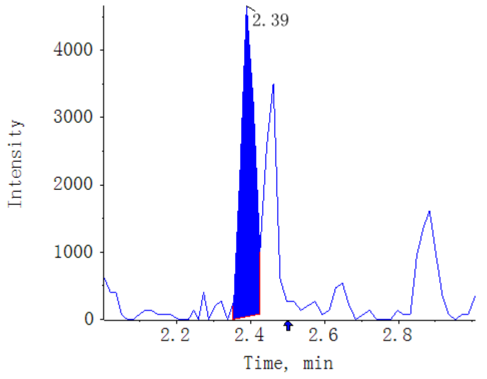

A21233261b_b
Baimaside AREA:6.88e4 S/N:71.7

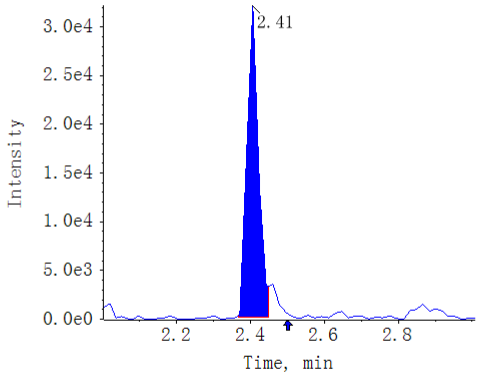

A21233262b_b
Baimaside AREA:9.22e4
S/N:111.5

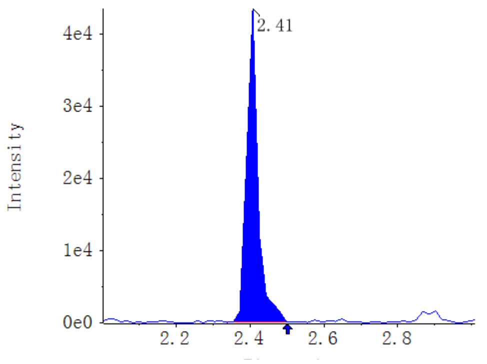

A21233263b_b
Baimaside AREA:1.45e4 S/N:18.6

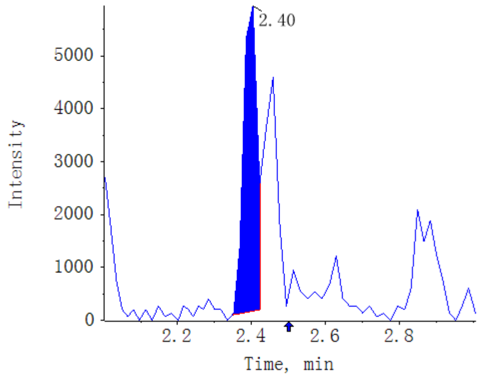

A21233264b_b
Baimaside AREA:4.87e3 S/N:8.8

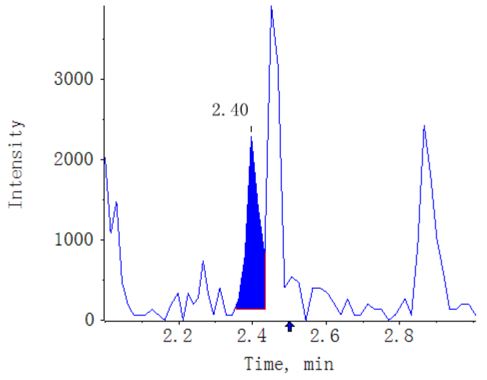

A21233265b_b
Baimaside AREA:2.41e3 S/N:6.0

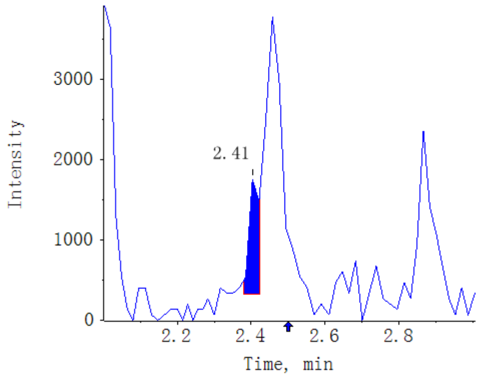

A21233266b_b
Baimaside AREA:3.01e4 S/N:39.0

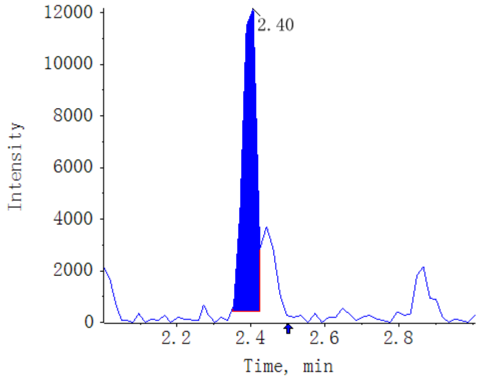

A21233267b_b
Baimaside AREA:2.59e3 S/N:4.6

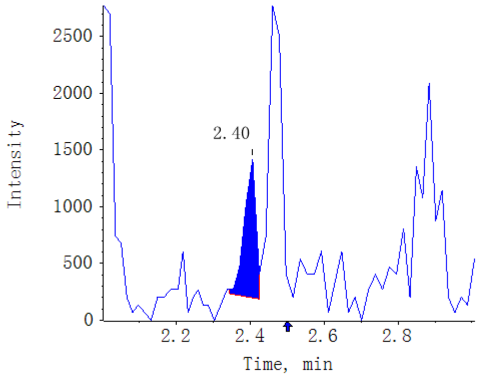

Result Table	MWXS-21-2101D-3_18_WH6500-5_A20-3_V1.0_TY_20211028	Algorithm Used	MQ4
Acquisition Method	Flavonoids_V1.0_WH6500-5_LT_20211025.dam	Instrument Name	QTRAP 6500+ Low Mass
Project	N/A	Analytes QTY	204:113

Compound name: Dihydrokaempferol (287.1 / 125.0)

Sample Name	Sample Type	Area (cps)	Is Area (cps)	RT (min)	S/N	Target Conc	Calculated Conc.()
STD_0.5nM	Standard	2.49e3	N/A	3.53	5.6	0.5000	3.386008e-1
STD_1nM	Standard	6.35e3	N/A	3.52	20.1	1.0000	8.328315e-1
STD_5nM	Standard	5.80e4	N/A	3.53	93.0	5.0000	7.456397e0
STD_10nM	Standard	7.52e4	N/A	3.53	105.8	10.0000	9.661809e0
STD_20nM	Standard	1.60e5	N/A	3.52	158.1	20.0000	2.053584e1
STD_50nM	Standard	4.00e5	N/A	3.53	151.6	50.0000	5.125116e1
STD_100nM	Standard	7.78e5	N/A	3.53	202.6	100.0000	9.971496e1
STD_200nM	Standard	1.53e6	N/A	3.52	207.8	200.0000	1.967084e2
STD_500nM	Standard	N/A	N/A	N/A	N/A	500.0000	N/A
STD_1000nM	Standard	N/A	N/A	N/A	N/A	1000.0000	N/A
STD_2000nM	Standard	N/A	N/A	N/A	N/A	2000.0000	N/A
V1.0_MW_RQC1_20211018	Quality Control	5.34e3	N/A	3.52	7.0	0.0000	7.037668e-1
Blank	Unknown	N/A	N/A	N/A	N/A	N/A	N/A
V1.0_MWMS_20211021_1	Unknown	1.17e6	N/A	3.54	233.4	N/A	1.497839e2
MWXS212101D3_R1	Quality Control	1.20e6	N/A	3.53	213.9	0.0000	1.536434e2
MWXS212101D3_R2	Quality Control	1.24e6	N/A	3.54	206.2	0.0000	1.588441e2
MWXS212101D3_R3	Quality Control	1.22e6	N/A	3.54	203.6	0.0000	1.565350e2
A21233250b_b	Unknown	4.95e3	N/A	3.54	9.2	N/A	6.533179e-1
A21233251b_b	Unknown	3.54e3	N/A	3.55	4.2	N/A	4.734929e-1
A21233252b_b	Unknown	4.49e3	N/A	3.53	6.2	N/A	5.953943e-1
A21233253b_b	Unknown	5.76e5	N/A	3.53	41.5	N/A	7.390473e1
A21233254b_b	Unknown	6.83e5	N/A	3.53	68.4	N/A	8.762392e1
A21233255b_b	Unknown	7.91e5	N/A	3.53	49.4	N/A	1.014510e2
A21233256b_b	Unknown	1.99e5	N/A	3.54	23.9	N/A	2.557938e1
A21233257b_b	Unknown	2.62e5	N/A	3.54	23.3	N/A	3.361223e1
A21233258b_b	Unknown	1.44e5	N/A	3.54	36.1	N/A	1.851658e1
A21233259b_b	Unknown	7.31e3	N/A	3.55	15.8	N/A	9.568670e-1
A21233260b_b	Unknown	3.22e3	N/A	3.54	4.4	N/A	4.322690e-1
A21233261b_b	Unknown	5.25e3	N/A	3.54	8.5	N/A	6.917752e-1
A21233262b_b	Unknown	2.23e5	N/A	3.54	99.7	N/A	2.862885e1
A21233263b_b	Unknown	6.99e5	N/A	3.53	44.3	N/A	8.967587e1
A21233264b_b	Unknown	7.43e5	N/A	3.54	42.3	N/A	9.523166e1
A21233265b_b	Unknown	3.93e5	N/A	3.54	24.8	N/A	5.040240e1
A21233266b_b	Unknown	4.77e5	N/A	3.53	37.8	N/A	6.118776e1
A21233267b_b	Unknown	1.58e5	N/A	3.54	17.9	N/A	2.025675e1

Compound name: Dihydrokaempferol
Regression Equation: y = 7800.13496 x + -149.89212 (r = 0.99810) (weighting: 1 / x)

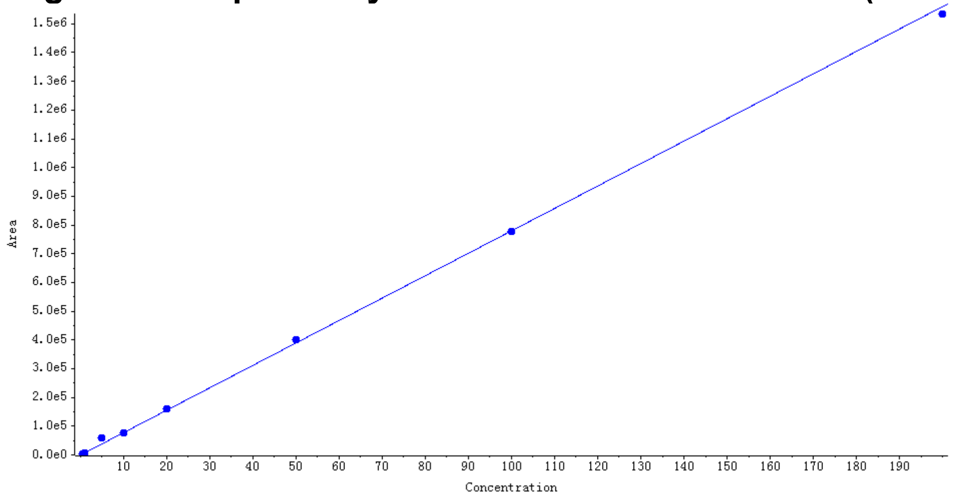

Peak Review

Blank
Dihydrokaempferol AREA:N/A
S/N:N/A

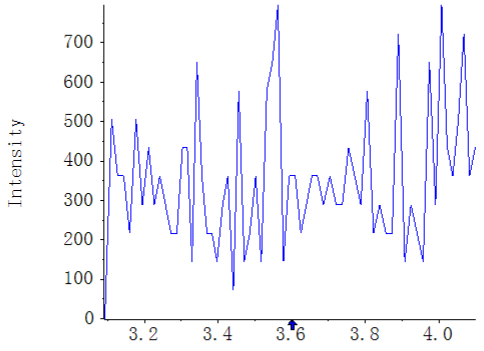

V1.0_MWMS_20211021_1
Dihydrokaempferol AREA:1.17e6
S/N:233.4

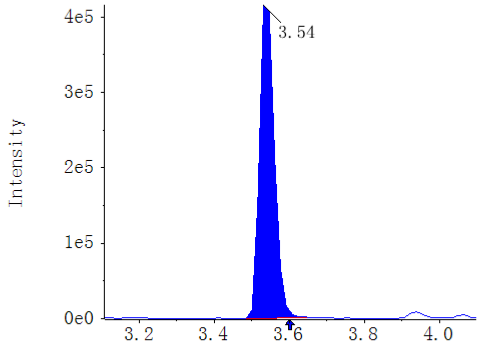

A21233250b_b
Dihydrokaempferol AREA:4.95e3
S/N:9.2

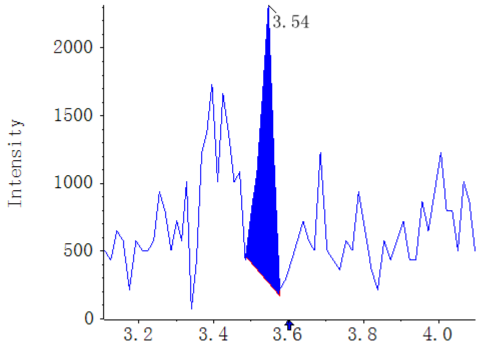

A21233251b_b
Dihydrokaempferol AREA:3.54e3
S/N:4.2

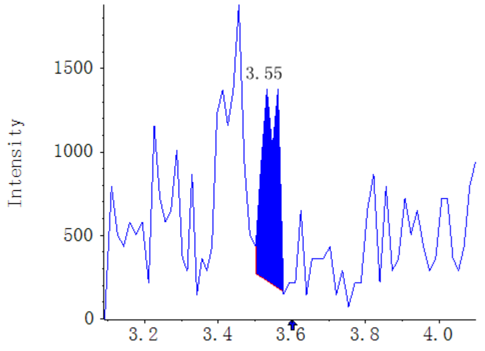

A21233252b_b
Dihydrokaempferol AREA:4.49e3
S/N:6.2

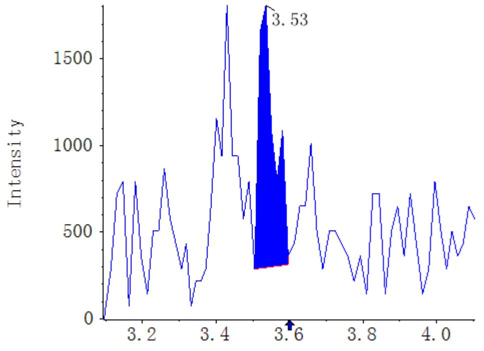

A21233253b_b
Dihydrokaempferol AREA:5.76e5
S/N:41.5

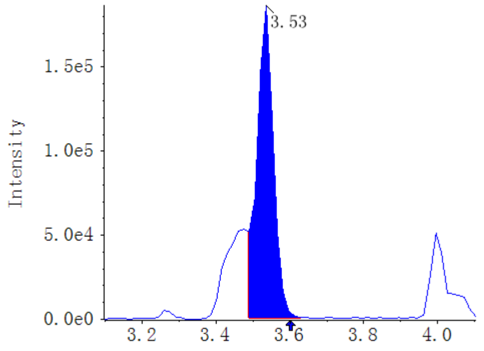

A21233254b_b
Dihydrokaempferol AREA:6.83e5
S/N:68.4

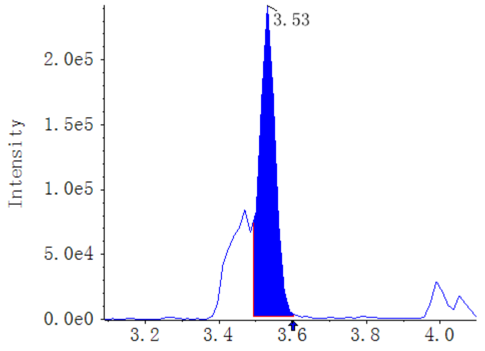

A21233255b_b
Dihydrokaempferol AREA:7.91e5
S/N:49.4

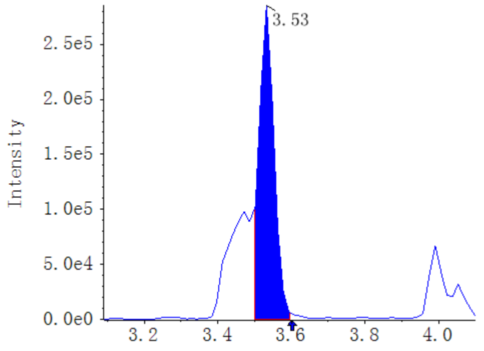

A21233256b_b
Dihydrokaempferol AREA:1.99e5
S/N:23.9

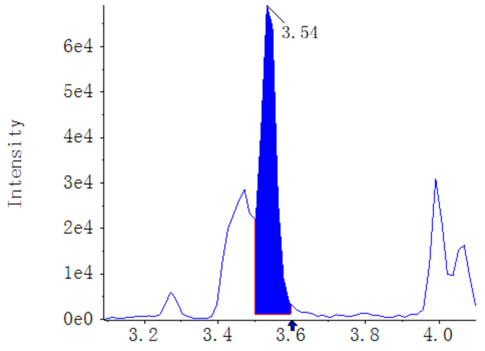

A21233257b_b
Dihydrokaempferol AREA:2.62e5
S/N:23.3

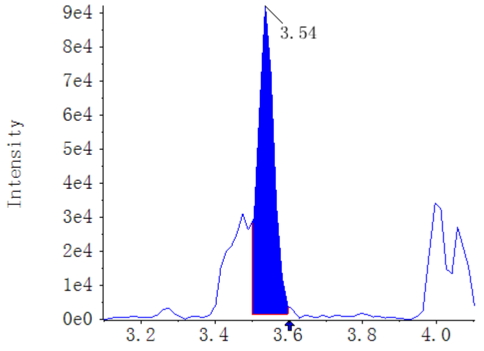

A21233258b_b
Dihydrokaempferol AREA:1.44e5
S/N:36.1

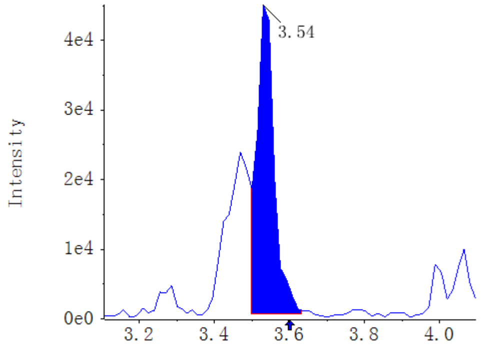

A21233259b_b
Dihydrokaempferol AREA:7.31e3
S/N:15.8

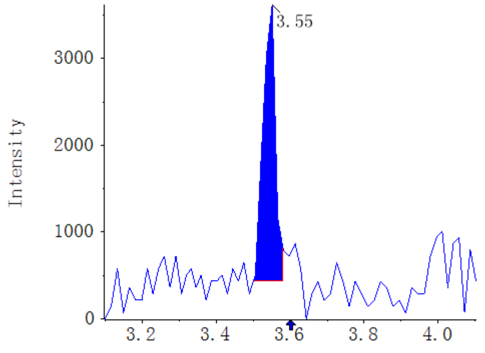

A21233260b_b
Dihydrokaempferol AREA:3.22e3
S/N:4.4

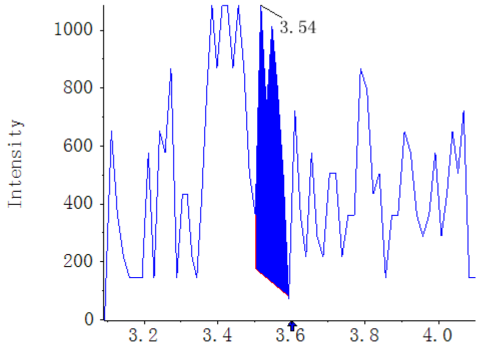

A21233261b_b
Dihydrokaempferol AREA:5.25e3
S/N:8.5

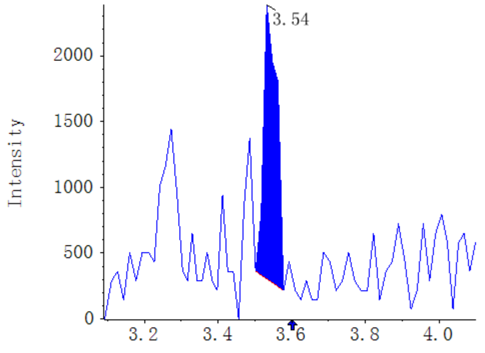

A21233262b_b
Dihydrokaempferol AREA:2.23e5
S/N:99.7

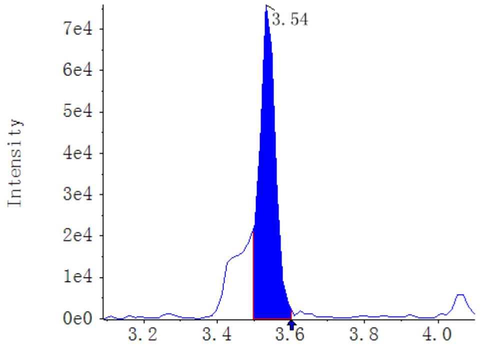

A21233263b_b
Dihydrokaempferol AREA:6.99e5
S/N:44.3

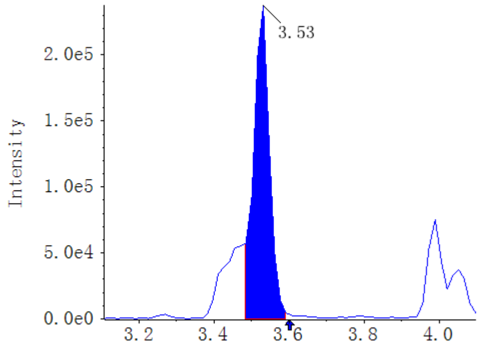

A21233264b_b
Dihydrokaempferol AREA:7.43e5
S/N:42.3

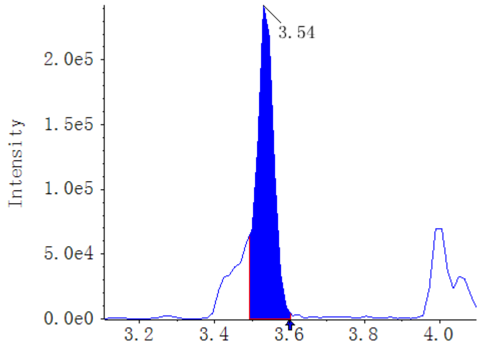

A21233265b_b
Dihydrokaempferol AREA:3.93e5
S/N:24.8

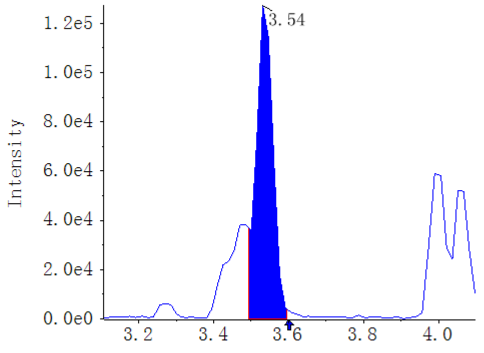

A21233266b_b
Dihydrokaempferol AREA:4.77e5
S/N:37.8

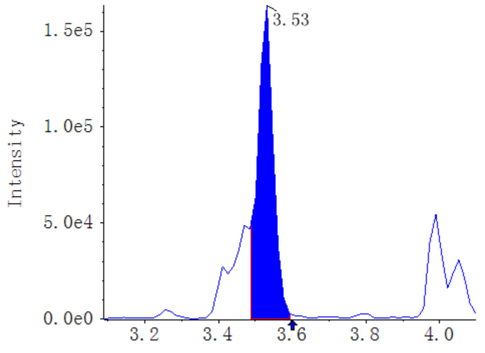

A21233267b_b
Dihydrokaempferol AREA:1.58e5
S/N:17.9

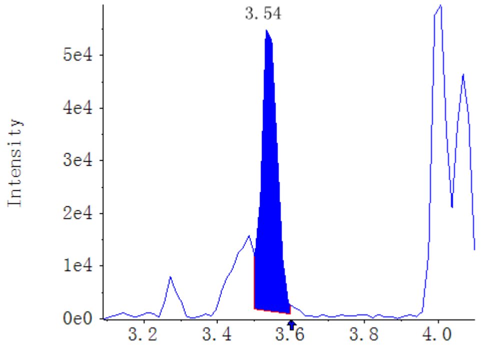

Result Table	MWXS-21-2101D-3_18_WH6500-5_A20-3_V1.0_TY_20211028	Algorithm Used	MQ4
Acquisition Method	Flavonoids_V1.0_WH6500-5_LT_20211025.dam	Instrument Name	QTRAP 6500+ Low Mass
Project	N/A	Analytes QTY	204:117

Compound name: Procyanidin B2 (577.1 / 407.1)

Sample Name	Sample Type	Area (cps)	Is Area (cps)	RT (min)	S/N	Target Conc	Calculated Conc.()
STD_0.5nM	Standard	1.25e3	N/A	2.21	4.7	0.5000	4.387243e-1
STD_1nM	Standard	1.78e3	N/A	2.22	9.9	1.0000	8.843911e-1
STD_5nM	Standard	8.86e3	N/A	2.22	51.8	5.0000	6.769898e0
STD_10nM	Standard	1.26e4	N/A	2.22	49.9	10.0000	9.873861e0
STD_20nM	Standard	2.19e4	N/A	2.22	72.2	20.0000	1.757610e1
STD_50nM	Standard	6.28e4	N/A	2.22	129.5	50.0000	5.164126e1
STD_100nM	Standard	1.18e5	N/A	2.23	182.0	100.0000	9.771708e1
STD_200nM	Standard	2.43e5	N/A	2.22	279.8	200.0000	2.015987e2
STD_500nM	Standard	N/A	N/A	N/A	N/A	500.0000	N/A
STD_1000nM	Standard	N/A	N/A	N/A	N/A	1000.0000	N/A
STD_2000nM	Standard	N/A	N/A	N/A	N/A	2000.0000	N/A
V1.0_MW_RQC1_20211018	Quality Control	N/A	N/A	N/A	N/A	0.0000	N/A
Blank	Unknown	N/A	N/A	N/A	N/A	N/A	N/A
V1.0_MWMS_20211021_1	Unknown	1.92e5	N/A	2.22	122.8	N/A	1.593647e2
MWXS212101D3_R1	Quality Control	1.95e5	N/A	2.22	118.7	0.0000	1.619343e2
MWXS212101D3_R2	Quality Control	1.90e5	N/A	2.22	123.1	0.0000	1.571268e2
MWXS212101D3_R3	Quality Control	2.04e5	N/A	2.22	140.3	0.0000	1.692067e2
A21233250b_b	Unknown	3.38e3	N/A	2.22	11.0	N/A	2.210904e0
A21233251b_b	Unknown	2.12e3	N/A	2.22	7.1	N/A	1.161958e0
A21233252b_b	Unknown	N/A	N/A	N/A	N/A	N/A	N/A
A21233253b_b	Unknown	3.40e3	N/A	2.22	16.0	N/A	2.226297e0
A21233254b_b	Unknown	3.78e3	N/A	2.22	9.0	N/A	2.542628e0
A21233255b_b	Unknown	N/A	N/A	N/A	N/A	N/A	N/A
A21233256b_b	Unknown	6.01e3	N/A	2.23	13.6	N/A	4.395992e0
A21233257b_b	Unknown	5.26e3	N/A	2.22	16.7	N/A	3.772568e0
A21233258b_b	Unknown	N/A	N/A	N/A	N/A	N/A	N/A
A21233259b_b	Unknown	2.15e4	N/A	2.22	41.6	N/A	1.731506e1
A21233260b_b	Unknown	4.70e4	N/A	2.22	61.4	N/A	3.848137e1
A21233261b_b	Unknown	2.36e4	N/A	2.23	28.7	N/A	1.906031e1
A21233262b_b	Unknown	N/A	N/A	N/A	N/A	N/A	N/A
A21233263b_b	Unknown	4.83e3	N/A	2.22	10.6	N/A	3.422076e0
A21233264b_b	Unknown	4.69e3	N/A	2.22	14.2	N/A	3.303977e0
A21233265b_b	Unknown	N/A	N/A	N/A	N/A	N/A	N/A
A21233266b_b	Unknown	N/A	N/A	N/A	N/A	N/A	N/A
A21233267b_b	Unknown	7.54e3	N/A	2.22	12.1	N/A	5.675382e0

Compound name: Procyanidin B2
Regression Equation: y = 1202.43685 x + 719.73578 (r = 0.99856) (weighting: 1 / x)

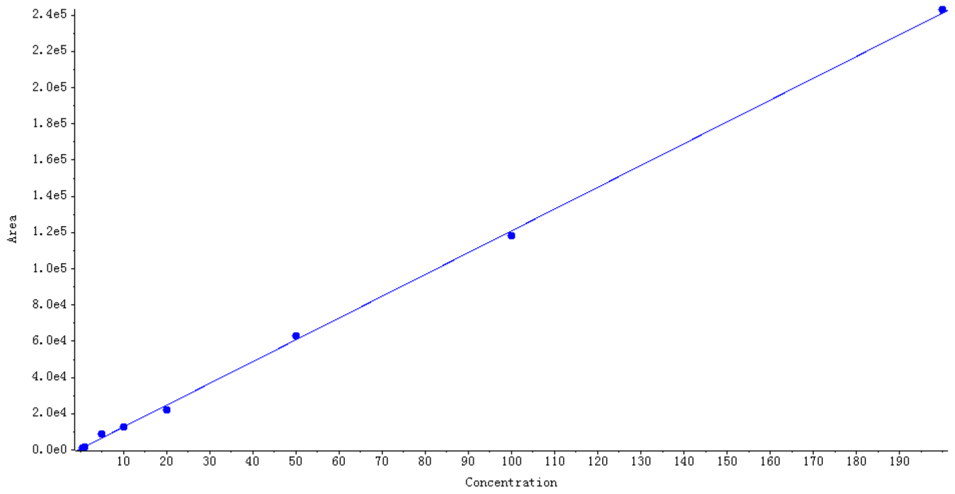

Peak Review

Blank
Procyanidin B2 AREA:N/A
S/N:N/A

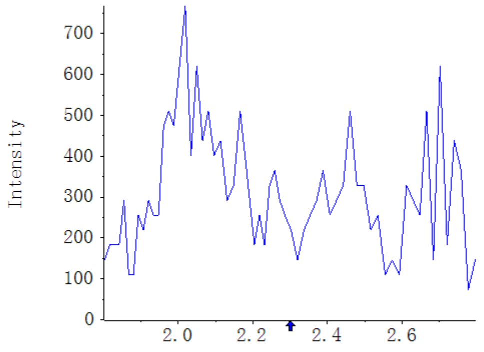

V1.0_MWMS_20211021_1
Procyanidin B2 AREA:1.92e5
S/N:122.8

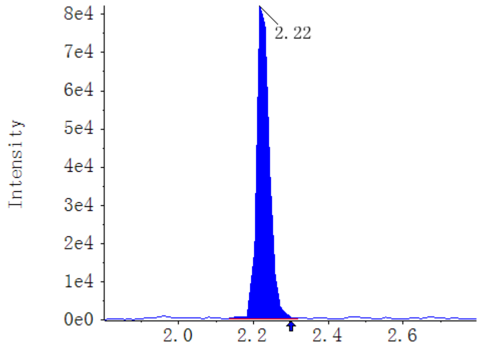

A21233250b_b
Procyanidin B2 AREA:3.38e3
S/N:11.0

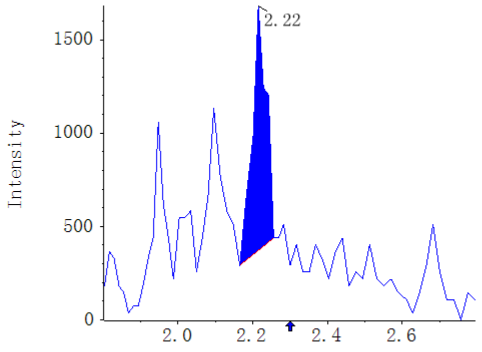

A21233251b_b
Procyanidin B2 AREA:2.12e3
S/N:7.1

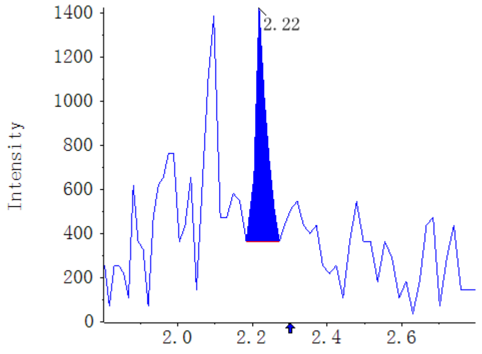

A21233252b_b
Procyanidin B2 AREA:N/A
S/N:N/A

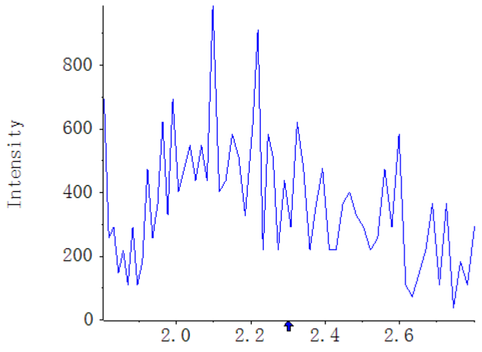

A21233253b_b
Procyanidin B2 AREA:3.40e3
S/N:16.0

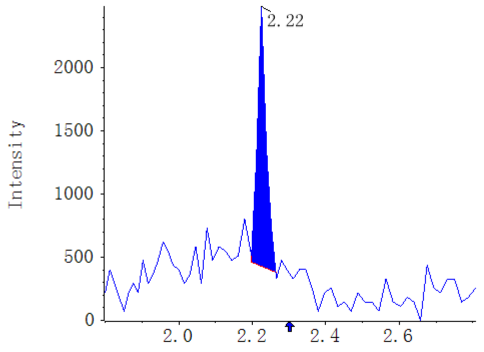

A21233254b_b
Procyanidin B2 AREA:3.78e3
S/N:9.0

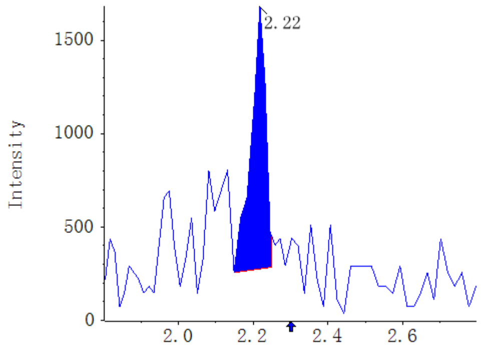

A21233255b_b
Procyanidin B2 AREA:N/A
S/N:N/A

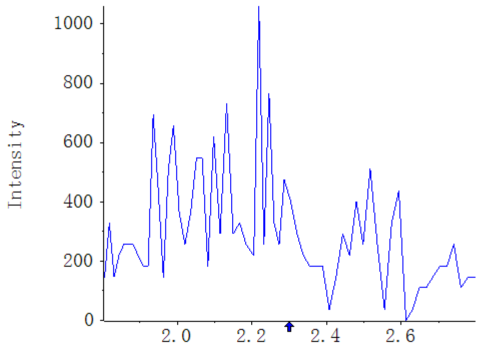

A21233256b_b
Procyanidin B2 AREA:6.01e3
S/N:13.6

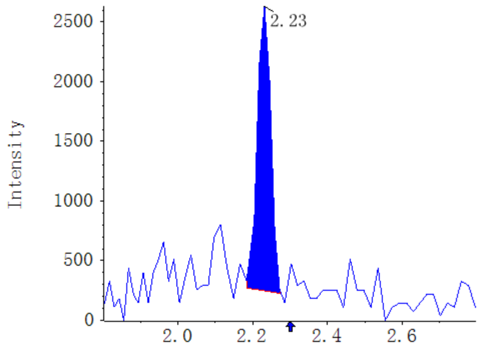

A21233257b_b
Procyanidin B2 AREA:5.26e3
S/N:16.7

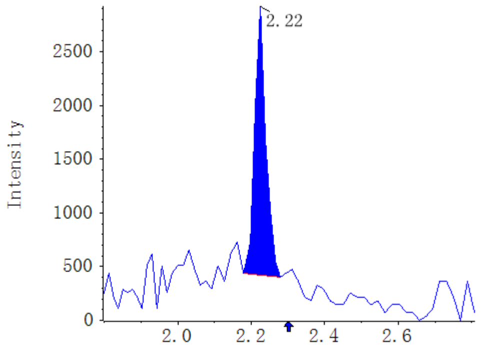

A21233258b_b
Procyanidin B2 AREA:N/A
S/N:N/A

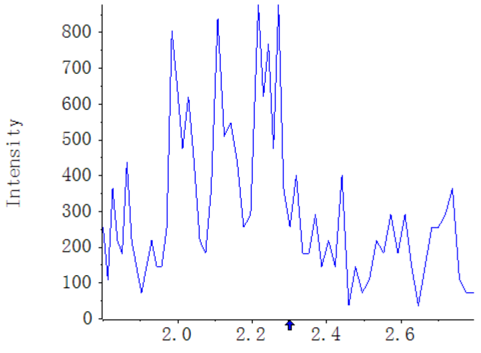

A21233259b_b
Procyanidin B2 AREA:2.15e4
S/N:41.6

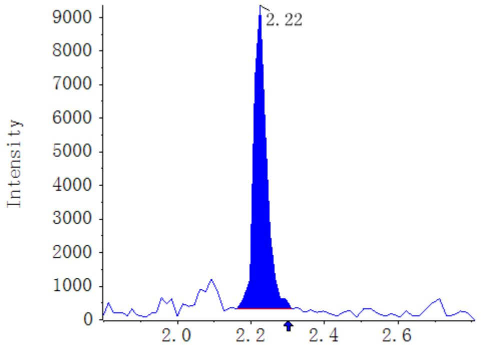

A21233260b_b
Procyanidin B2 AREA:4.70e4
S/N:61.4

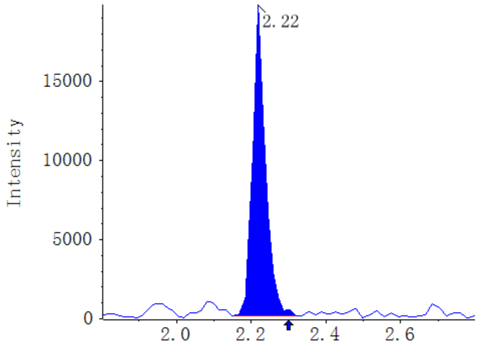

A21233261b_b
Procyanidin B2 AREA:2.36e4
S/N:28.7

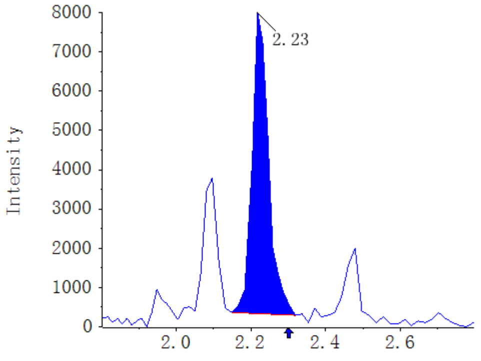

A21233262b_b
Procyanidin B2 AREA:N/A
S/N:N/A

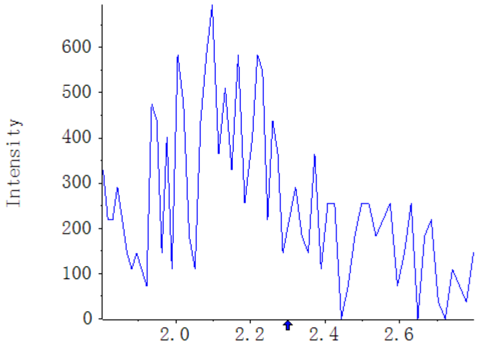

A21233263b_b
Procyanidin B2 AREA:4.83e3
S/N:10.6

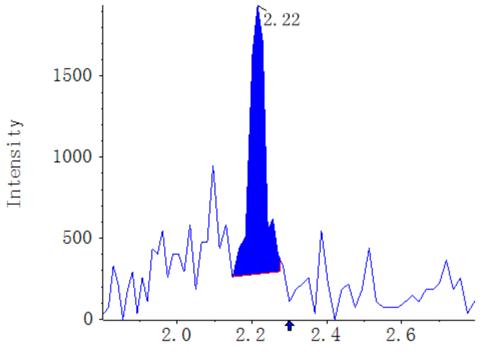

A21233264b_b
Procyanidin B2 AREA:4.69e3
S/N:14.2

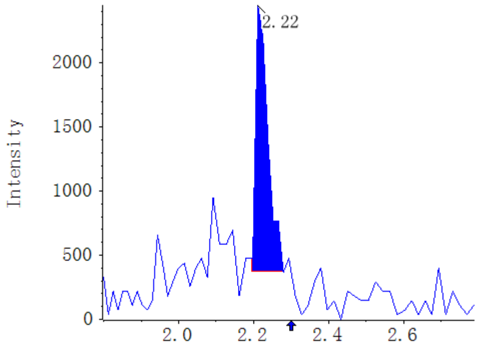

A21233265b_b
Procyanidin B2 AREA:N/A
S/N:N/A

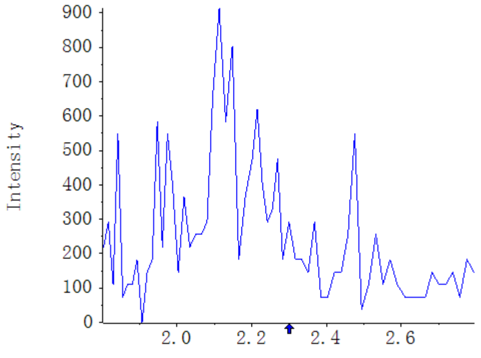

A21233266b_b
Procyanidin B2 AREA:N/A
S/N:N/A

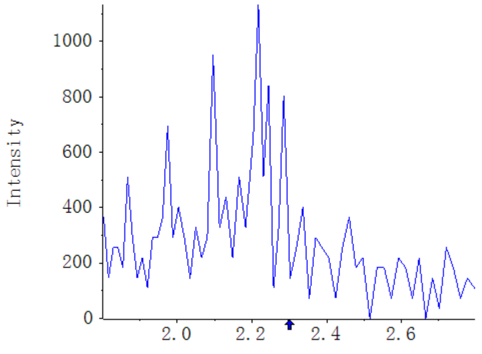

A21233267b_b
Procyanidin B2 AREA:7.54e3
S/N:12.1

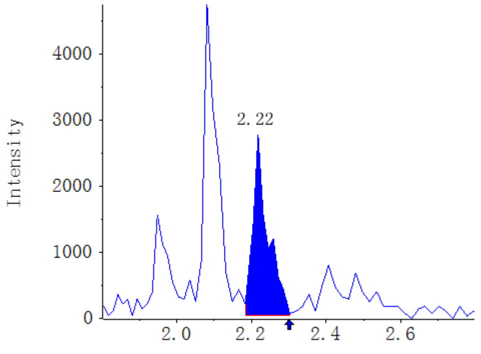

**Result Table**	MWXS-21-2101D-3_18_WH6500-5_A20-3_V1.0_TY_20211028	**Algorithm Used**	MQ4
**Acquisition Method**	Flavonoids_V1.0_WH6500-5_LT_20211025.dam	**Instrument Name**	QTRAP 6500+ Low Mass
**Project**	N/A	**Analytes QTY**	204:119

Compound name: (-)-Epicatechin (289.1 / 109.0)

Sample Name	Sample Type	Area (cps)	Is Area (cps)	RT (min)	S/N	Target Conc	Calculated Conc.()
STD_0.5nM	Standard	2.14e3	N/A	2.36	5.1	0.5000	3.637130e-1
STD_1nM	Standard	3.21e3	N/A	2.38	8.0	1.0000	1.026735e0
STD_5nM	Standard	1.26e4	N/A	2.39	21.0	5.0000	6.800297e0
STD_10nM	Standard	1.70e4	N/A	2.39	28.3	10.0000	9.503262e0
STD_20nM	Standard	3.30e4	N/A	2.38	39.5	20.0000	1.938113e1
STD_50nM	Standard	8.07e4	N/A	2.39	63.2	50.0000	4.880587e1
STD_100nM	Standard	1.60e5	N/A	2.39	63.0	100.0000	9.743692e1
STD_200nM	Standard	3.31e5	N/A	2.38	87.0	200.0000	2.031821e2
STD_500nM	Standard	N/A	N/A	N/A	N/A	500.0000	N/A
STD_1000nM	Standard	N/A	N/A	N/A	N/A	1000.0000	N/A
STD_2000nM	Standard	N/A	N/A	N/A	N/A	2000.0000	N/A
V1.0_MW_RQC1_20211018	Quality Control	N/A	N/A	N/A	N/A	0.0000	N/A
Blank	Unknown	N/A	N/A	N/A	N/A	N/A	N/A
V1.0_MWMS_20211021_1	Unknown	2.57e5	N/A	2.39	69.4	N/A	1.577497e2
MWXS212101D3_R1	Quality Control	2.71e5	N/A	2.39	67.6	0.0000	1.664434e2
MWXS212101D3_R2	Quality Control	2.68e5	N/A	2.39	81.2	0.0000	1.643503e2
MWXS212101D3_R3	Quality Control	2.62e5	N/A	2.39	70.9	0.0000	1.605789e2
A21233250b_b	Unknown	1.98e4	N/A	2.40	14.7	N/A	1.125748e1
A21233251b_b	Unknown	1.43e4	N/A	2.40	11.5	N/A	7.881598e0
A21233252b_b	Unknown	5.09e3	N/A	2.40	4.4	N/A	2.182918e0
A21233253b_b	Unknown	1.10e4	N/A	2.39	6.2	N/A	5.844982e0
A21233254b_b	Unknown	1.30e4	N/A	2.40	8.6	N/A	7.068088e0
A21233255b_b	Unknown	5.62e3	N/A	2.40	4.1	N/A	2.510533e0
A21233256b_b	Unknown	1.70e4	N/A	2.39	6.4	N/A	9.545901e0
A21233257b_b	Unknown	1.46e4	N/A	2.39	2.8	N/A	8.049653e0
A21233258b_b	Unknown	8.81e3	N/A	2.40	4.2	N/A	4.478551e0
A21233259b_b	Unknown	2.63e4	N/A	2.39	23.8	N/A	1.524820e1
A21233260b_b	Unknown	6.38e4	N/A	2.39	41.8	N/A	3.837825e1
A21233261b_b	Unknown	2.04e5	N/A	2.39	46.0	N/A	1.249132e2
A21233262b_b	Unknown	6.20e3	N/A	2.40	5.5	N/A	2.872868e0
A21233263b_b	Unknown	9.91e3	N/A	2.39	6.3	N/A	5.157025e0
A21233264b_b	Unknown	1.08e4	N/A	2.40	4.9	N/A	5.732591e0
A21233265b_b	Unknown	5.66e3	N/A	2.41	2.7	N/A	2.537508e0
A21233266b_b	Unknown	7.33e3	N/A	2.40	3.4	N/A	3.565371e0
A21233267b_b	Unknown	1.71e4	N/A	2.39	4.4	N/A	9.600235e0

Compound name: (-)-Epicatechin
Regression Equation: y = 1621.16703 x + 1547.17300 (r = 0.99881) (weighting: 1 / x)

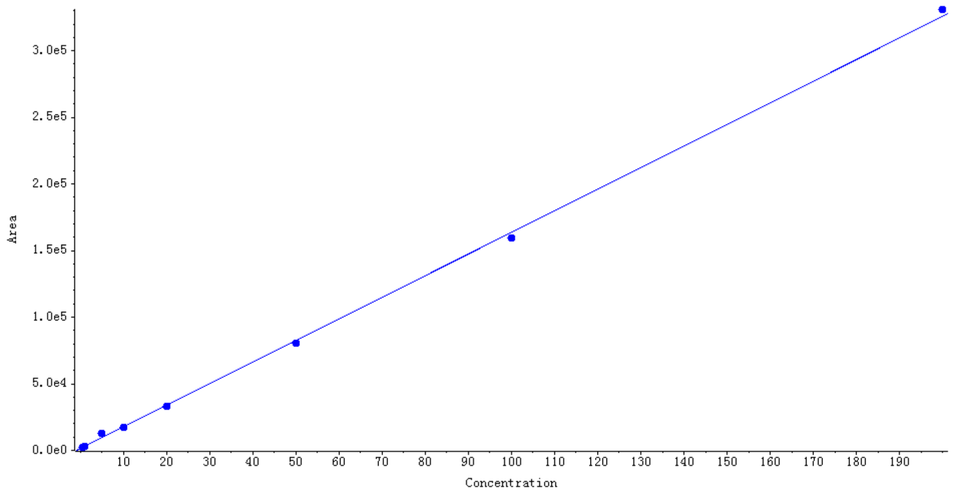

Peak Review

Blank
(-)-Epicatechin AREA:N/A S/N:N/A

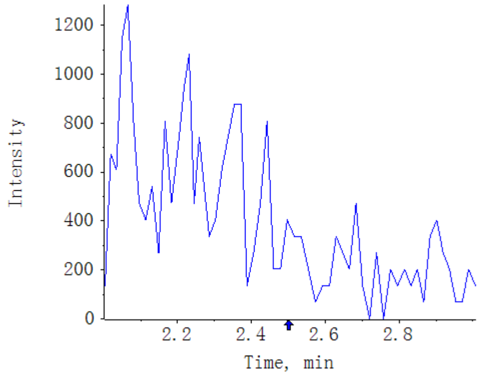

V1.0_MWMS_20211021_1
(-)-Epicatechin AREA:2.57e5 S/N:69.4

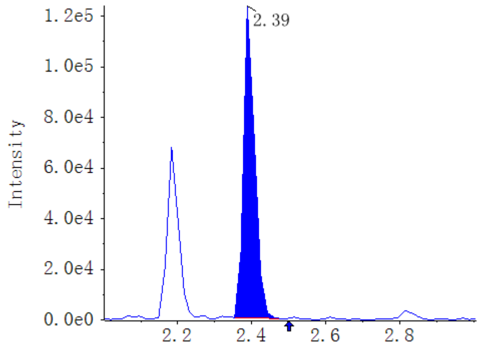

A21233250b_b
(-)-Epicatechin AREA:1.98e4 S/N:14.7

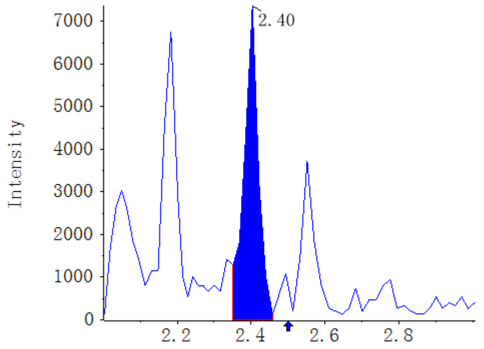

A21233251b_b
(-)-Epicatechin AREA:1.43e4 S/N:11.5

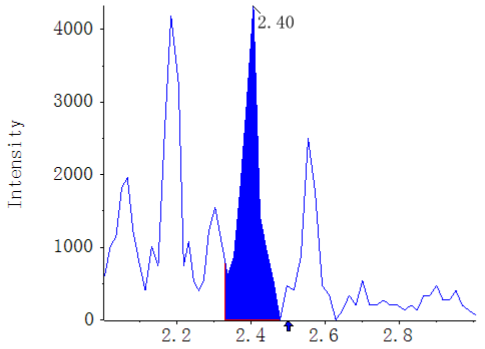

A21233252b_b
(-)-Epicatechin AREA:5.09e3 S/N:4.4

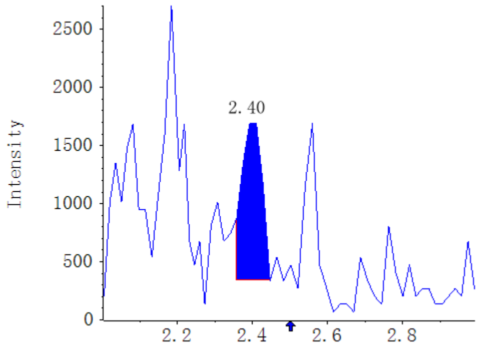

A21233253b_b
(-)-Epicatechin AREA:1.10e4 S/N:6.2

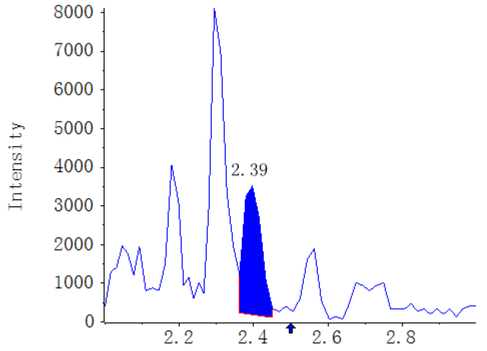

A21233254b_b
(-)-Epicatechin AREA:1.30e4 S/N:8.6

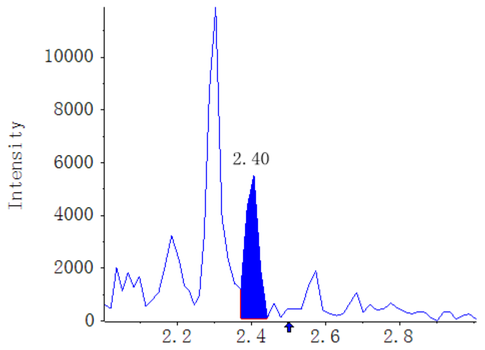

A21233255b_b
(-)-Epicatechin AREA:5.62e3 S/N:4.1

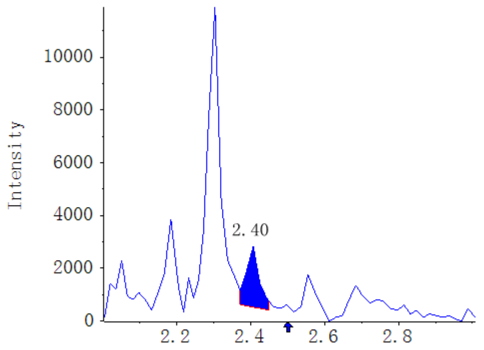

A21233256b_b
(-)-Epicatechin AREA:1.70e4 S/N:6.4

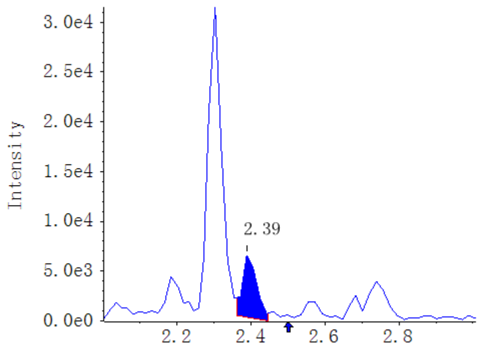

A21233257b_b
(-)-Epicatechin AREA:1.46e4 S/N:2.8

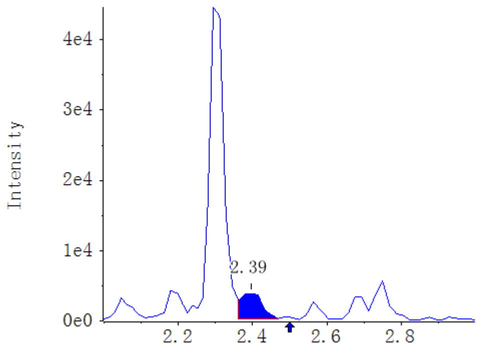

A21233258b_b
(-)-Epicatechin AREA:8.81e3
S/N:4.2

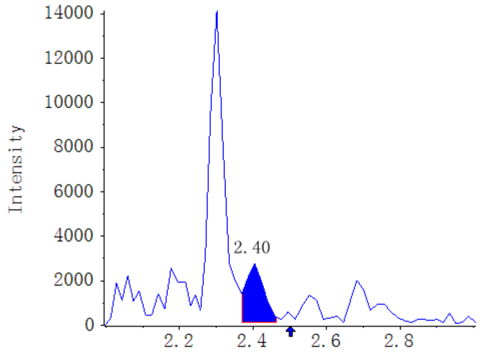

A21233259b_b
(-)-Epicatechin AREA:2.63e4
S/N:23.8

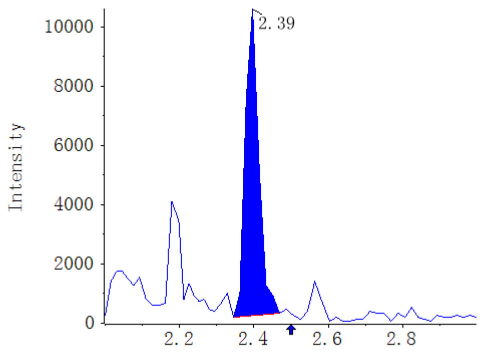

A21233260b_b
(-)-Epicatechin AREA:6.38e4
S/N:41.8

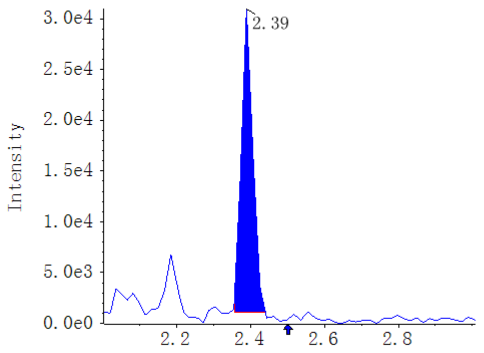

A21233261b_b
(-)-Epicatechin AREA:2.04e5
S/N:46.0

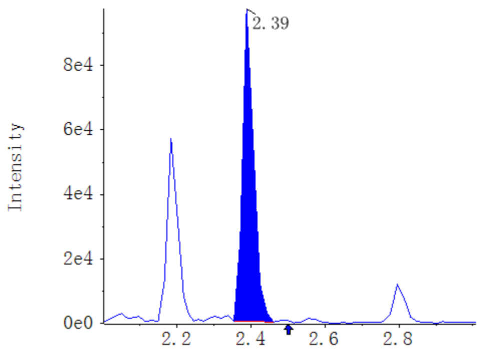

A21233262b_b
(-)-Epicatechin AREA:6.20e3
S/N:5.5

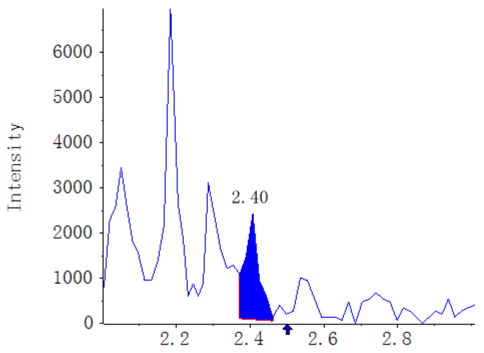

A21233263b_b
(-)-Epicatechin AREA:9.91e3
S/N:6.3

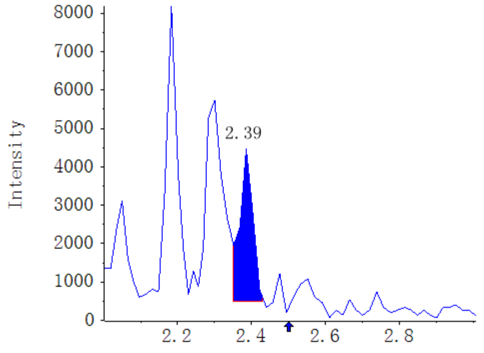

A21233264b_b
(-)-Epicatechin AREA:1.08e4
S/N:4.9

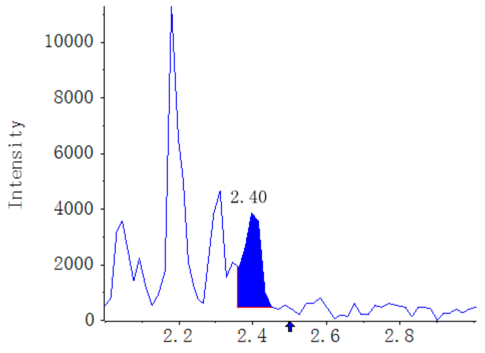

A21233265b_b
(-)-Epicatechin AREA:5.66e3
S/N:2.7

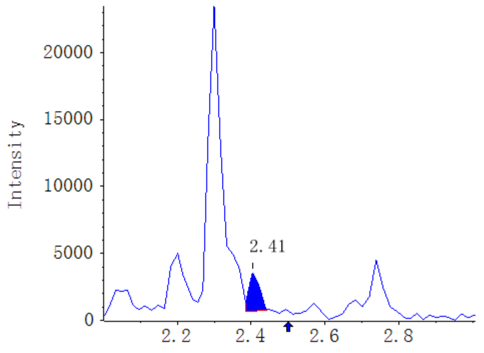

A21233266b_b
(-)-Epicatechin AREA:7.33e3
S/N:3.4

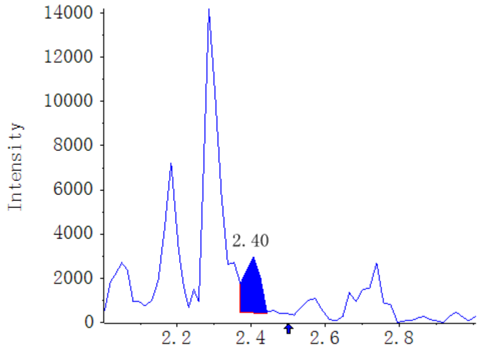

A21233267b_b
(-)-Epicatechin AREA:1.71e4
S/N:4.4

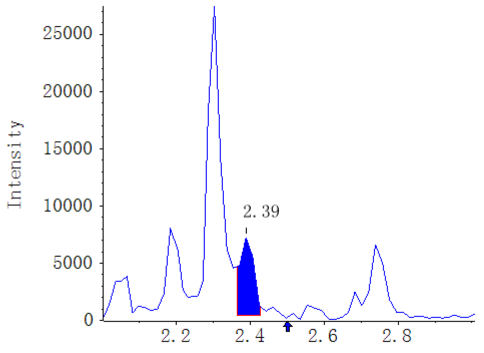

**Result Table**	MWXS-21-2101D-3_18_WH6500-5_A20-3_V1.0_TY_20211028	**Algorithm Used**	MQ4
**Acquisition Method**	Flavonoids_V1.0_WH6500-5_LT_20211025.dam	**Instrument Name**	QTRAP 6500+ Low Mass
**Project**	N/A	**Analytes QTY**	204:122

Compound name: (-)-Catechin gallate (441.1 / 169.0)

Sample Name	Sample Type	Area (cps)	Is Area (cps)	RT (min)	S/N	Target Conc	Calculated Conc.()
STD_0.5nM	Standard	9.17e3	N/A	2.80	34.3	0.5000	3.713242e-1
STD_1nM	Standard	1.48e4	N/A	2.80	31.5	1.0000	1.072626e0
STD_5nM	Standard	5.62e4	N/A	2.81	80.5	5.0000	6.206785e0
STD_10nM	Standard	8.28e4	N/A	2.81	109.1	10.0000	9.512935e0
STD_20nM	Standard	1.64e5	N/A	2.81	193.5	20.0000	1.961963e1
STD_50nM	Standard	4.20e5	N/A	2.81	181.9	50.0000	5.142877e1
STD_100nM	Standard	7.97e5	N/A	2.82	192.3	100.0000	9.821576e1
STD_200nM	Standard	1.62e6	N/A	2.81	196.7	200.0000	2.000722e2
STD_500nM	Standard	N/A	N/A	N/A	N/A	500.0000	N/A
STD_1000nM	Standard	N/A	N/A	N/A	N/A	1000.0000	N/A
STD_2000nM	Standard	N/A	N/A	N/A	N/A	2000.0000	N/A
V1.0_MW_RQC1_20211018	Quality Control	2.38e4	N/A	2.79	17.8	0.0000	2.187240e0
Blank	Unknown	N/A	N/A	N/A	N/A	N/A	N/A
V1.0_MWMS_20211021_1	Unknown	1.38e6	N/A	2.82	261.4	N/A	1.700481e2
MWXS212101D3_R1	Quality Control	1.42e6	N/A	2.81	326.5	0.0000	1.759931e2
MWXS212101D3_R2	Quality Control	1.40e6	N/A	2.82	261.2	0.0000	1.736689e2
MWXS212101D3_R3	Quality Control	1.37e6	N/A	2.82	232.8	0.0000	1.695664e2
A21233250b_b	Unknown	2.57e4	N/A	2.79	24.7	N/A	2.425192e0
A21233251b_b	Unknown	1.34e4	N/A	2.80	21.4	N/A	8.966594e-1
A21233252b_b	Unknown	1.11e4	N/A	2.79	11.2	N/A	6.124825e-1
A21233253b_b	Unknown	1.80e4	N/A	2.80	14.4	N/A	1.470894e0
A21233254b_b	Unknown	1.48e4	N/A	2.80	13.9	N/A	1.069717e0
A21233255b_b	Unknown	6.77e3	N/A	2.79	6.2	N/A	7.263458e-2
A21233256b_b	Unknown	4.87e4	N/A	2.80	29.7	N/A	5.280764e0
A21233257b_b	Unknown	6.80e3	N/A	2.79	6.1	N/A	7.624027e-2
A21233258b_b	Unknown	1.03e4	N/A	2.80	5.5	N/A	5.148400e-1
A21233259b_b	Unknown	7.49e4	N/A	2.80	36.0	N/A	8.539600e0
A21233260b_b	Unknown	1.79e5	N/A	2.80	68.0	N/A	2.144302e1
A21233261b_b	Unknown	3.06e6	N/A	2.80	245.0	N/A	3.792351e2
A21233262b_b	Unknown	7.03e3	N/A	2.79	8.0	N/A	1.053520e-1
A21233263b_b	Unknown	1.72e4	N/A	2.79	14.5	N/A	1.366088e0
A21233264b_b	Unknown	3.57e4	N/A	2.80	19.7	N/A	3.663833e0
A21233265b_b	Unknown	1.77e4	N/A	2.79	11.1	N/A	1.435023e0
A21233266b_b	Unknown	1.04e4	N/A	2.79	8.1	N/A	5.197340e-1
A21233267b_b	Unknown	2.77e4	N/A	2.80	15.9	N/A	2.670438e0

Compound name: (-)-Catechin gallate
Regression Equation: y = 8051.04169 x + 6183.34194 (r = 0.99941) (weighting: 1 / x)

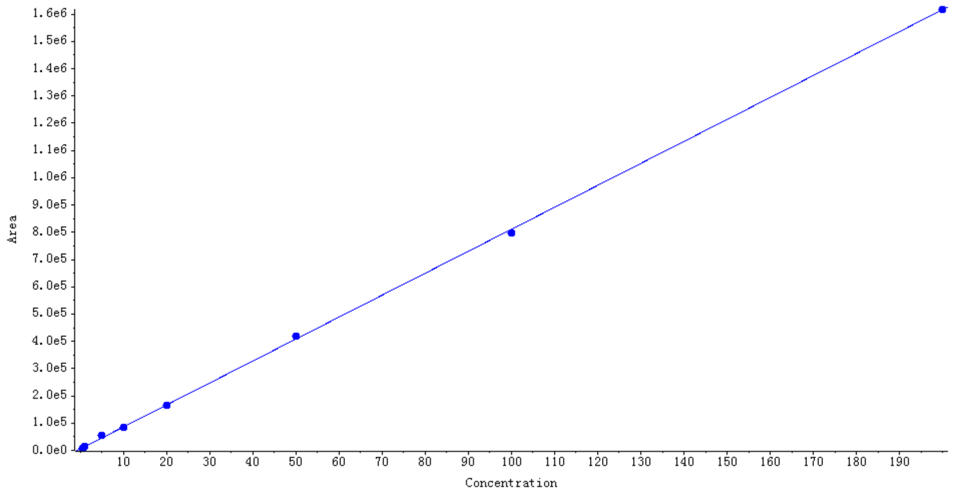

Peak Review

Blank
(-)-Catechin gallate AREA:N/A
S/N:N/A

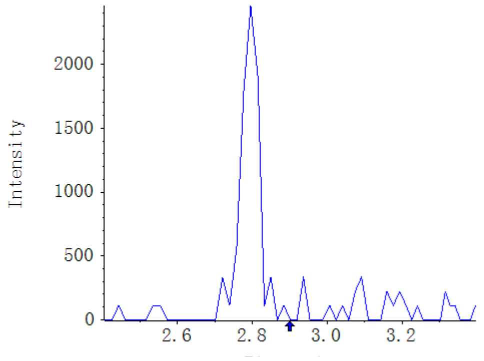

V1.0_MWMS_20211021_1
(-)-Catechin gallate AREA:1.38e6
S/N:261.4

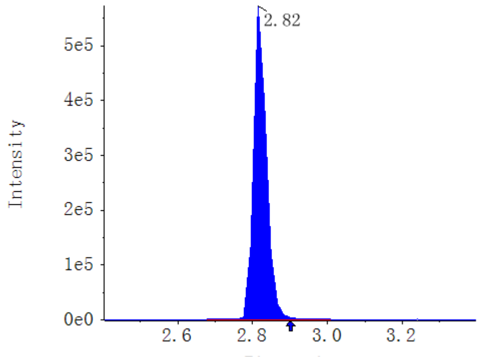

A21233250b_b
(-)-Catechin gallate AREA:2.57e4
S/N:24.7

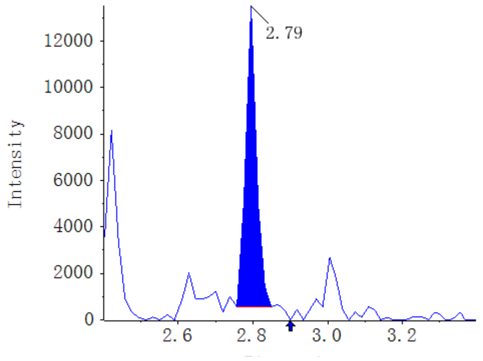

A21233251b_b
(-)-Catechin gallate AREA:1.34e4
S/N:21.4

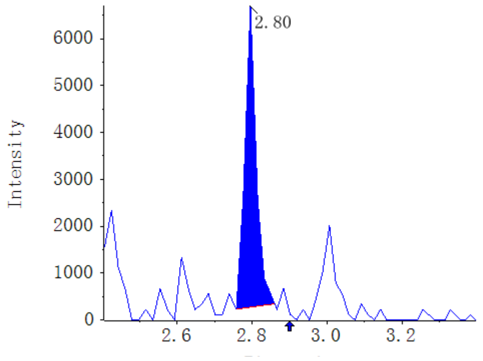

A21233252b_b
(-)-Catechin gallate AREA:1.11e4
S/N:11.2

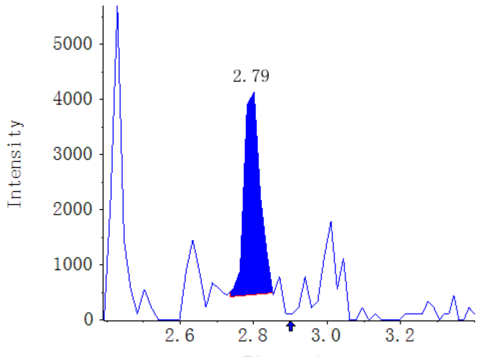

A21233253b_b
(-)-Catechin gallate AREA:1.80e4
S/N:14.4

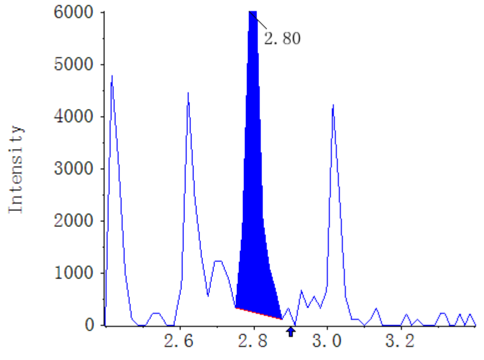

A21233254b_b
(-)-Catechin gallate AREA:1.48e4
S/N:13.9

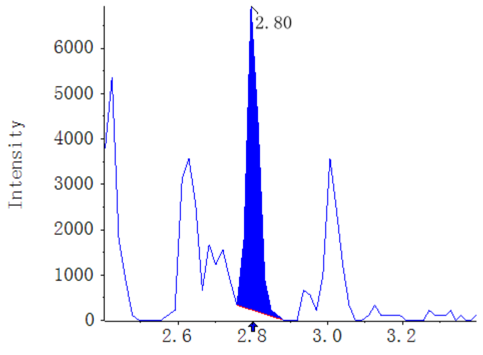

A21233255b_b
(-)-Catechin gallate AREA:6.77e3
S/N:6.2

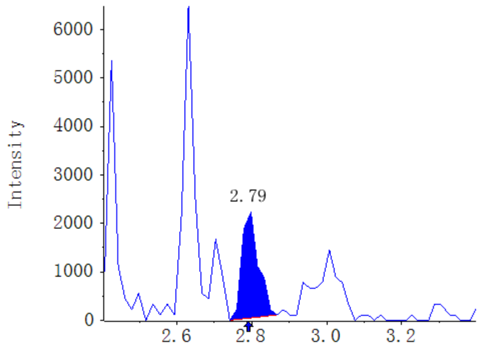

A21233256b_b
(-)-Catechin gallate AREA:4.87e4
S/N:29.7

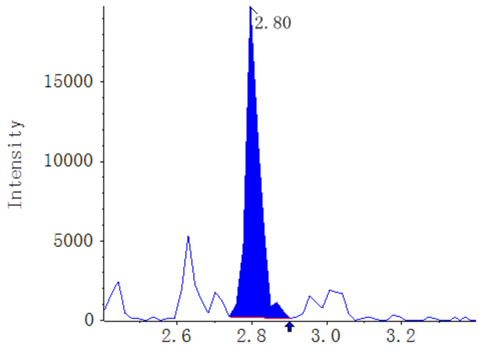

A21233257b_b
(-)-Catechin gallate AREA:6.80e3
S/N:6.1

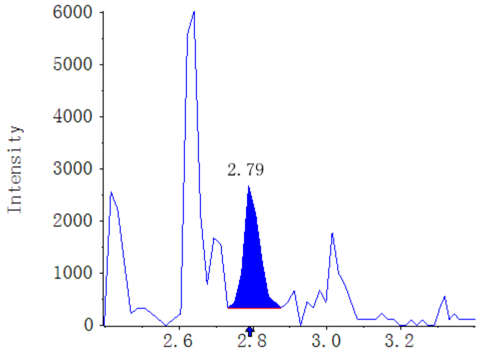

A21233258b_b
(-)-Catechin gallate AREA:1.03e4
S/N:5.5

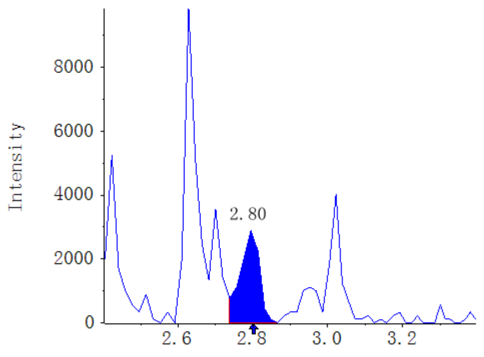

A21233259b_b
(-)-Catechin gallate AREA:7.49e4
S/N:36.0

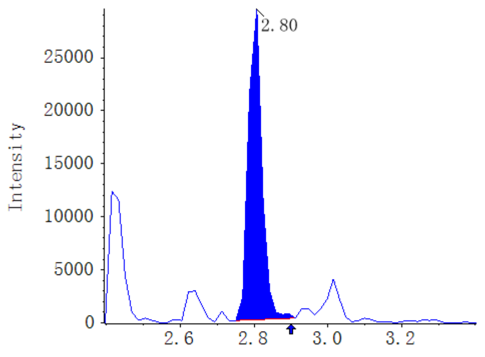

A21233260b_b
(-)-Catechin gallate AREA:1.79e5
S/N:68.0

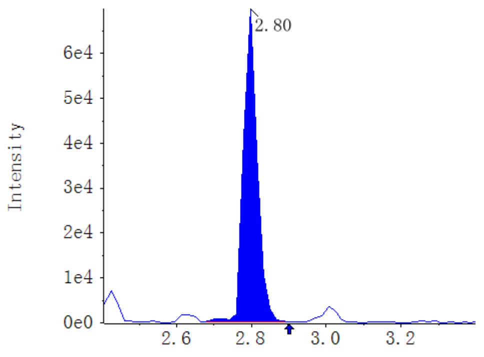

A21233261b_b
(-)-Catechin gallate AREA:3.06e6
S/N:245.0

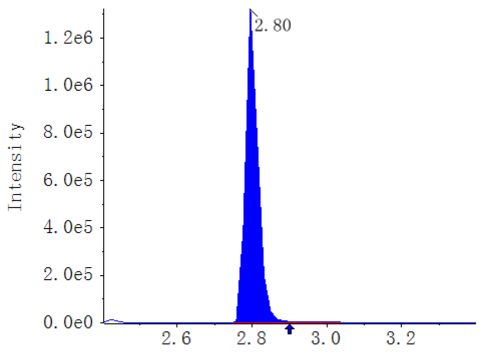

A21233262b_b
(-)-Catechin gallate AREA:7.03e3
S/N:8.0

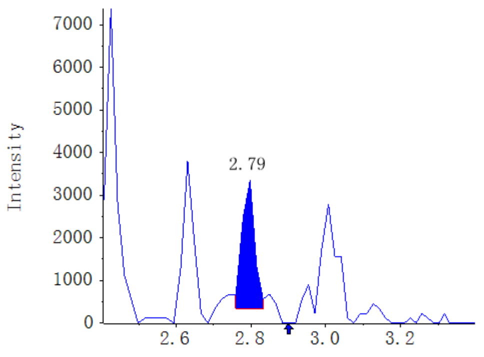

A21233263b_b
(-)-Catechin gallate AREA:1.72e4
S/N:14.5

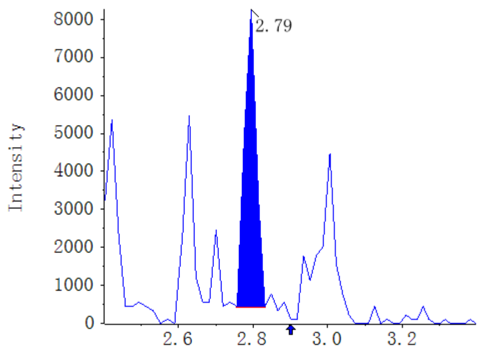

A21233264b_b
(-)-Catechin gallate AREA:3.57e4
S/N:19.7

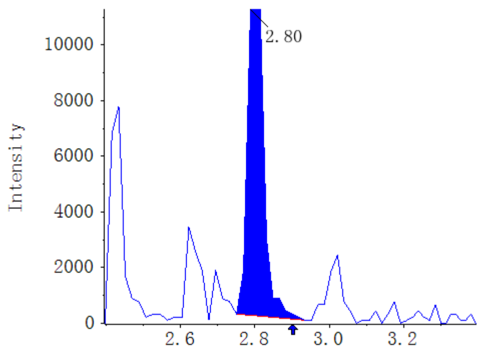

A21233265b_b
(-)-Catechin gallate AREA:1.77e4
S/N:11.1

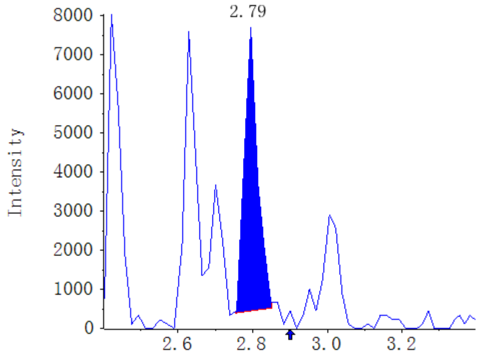

A21233266b_b
(-)-Catechin gallate AREA:1.04e4
S/N:8.1

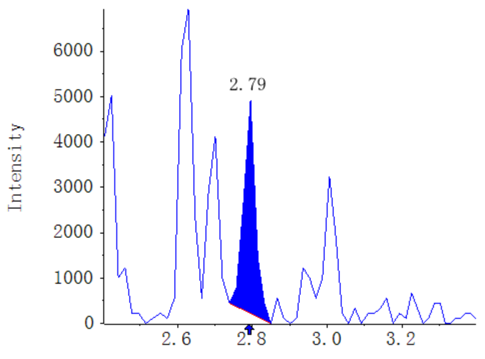

A21233267b_b
(-)-Catechin gallate AREA:2.77e4
S/N:15.9

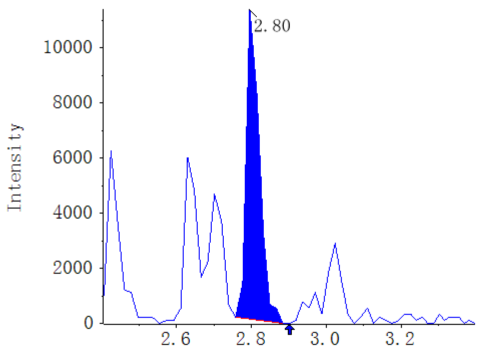

**Result Table**	MWXS-21-2101D-3_18_WH6500-5_A20-3_V1.0_TY_20211028	**Algorithm Used**	MQ4
**Acquisition Method**	Flavonoids_V1.0_WH6500-5_LT_20211025.dam	**Instrument Name**	QTRAP 6500+ Low Mass
**Project**	N/A	**Analytes QTY**	204:124

Compound name: Glycitin (491.1 / 283.1)

Sample Name	Sample Type	Area (cps)	Is Area (cps)	RT (min)	S/N	Target Conc	Calculated Conc.()
STD_0.5nM	Standard	1.75e3	N/A	2.55	17.6	0.5000	3.118867e-1
STD_1nM	Standard	3.71e3	N/A	2.54	31.5	1.0000	8.814235e-1
STD_5nM	Standard	2.59e4	N/A	2.55	62.2	5.0000	7.360354e0
STD_10nM	Standard	3.31e4	N/A	2.55	67.0	10.0000	9.458455e0
STD_20nM	Standard	7.03e4	N/A	2.55	117.6	20.0000	2.031328e1
STD_50nM	Standard	1.91e5	N/A	2.55	178.5	50.0000	5.560798e1
STD_100nM	Standard	3.34e5	N/A	2.56	117.3	100.0000	9.724606e1
STD_200nM	Standard	6.70e5	N/A	2.54	122.8	200.0000	1.953206e2
STD_500nM	Standard	N/A	N/A	N/A	N/A	500.0000	N/A
STD_1000nM	Standard	N/A	N/A	N/A	N/A	1000.0000	N/A
STD_2000nM	Standard	N/A	N/A	N/A	N/A	2000.0000	N/A
V1.0_MW_RQC1_20211018	Quality Control	N/A	N/A	N/A	N/A	0.0000	N/A
Blank	Unknown	N/A	N/A	N/A	N/A	N/A	N/A
V1.0_MWMS_20211021_1	Unknown	5.09e5	N/A	2.55	135.1	N/A	1.483464e2
MWXS212101D3_R1	Quality Control	5.78e5	N/A	2.55	165.4	0.0000	1.684759e2
MWXS212101D3_R2	Quality Control	4.90e5	N/A	2.55	150.7	0.0000	1.428343e2
MWXS212101D3_R3	Quality Control	5.54e5	N/A	2.55	133.6	0.0000	1.616138e2
A21233250b_b	Unknown	N/A	N/A	N/A	N/A	N/A	N/A
A21233251b_b	Unknown	N/A	N/A	N/A	N/A	N/A	N/A
A21233252b_b	Unknown	N/A	N/A	N/A	N/A	N/A	N/A
A21233253b_b	Unknown	1.83e4	N/A	2.62	13.9	N/A	5.143005e0
A21233254b_b	Unknown	1.20e4	N/A	2.61	8.3	N/A	3.294717e0
A21233255b_b	Unknown	1.27e4	N/A	2.62	8.0	N/A	3.500754e0
A21233256b_b	Unknown	1.09e4	N/A	2.62	5.0	N/A	2.985480e0
A21233257b_b	Unknown	1.13e4	N/A	2.63	6.8	N/A	3.094693e0
A21233258b_b	Unknown	1.20e4	N/A	2.62	4.8	N/A	3.302655e0
A21233259b_b	Unknown	9.63e3	N/A	2.62	5.7	N/A	2.610417e0
A21233260b_b	Unknown	1.07e4	N/A	2.62	7.7	N/A	2.934116e0
A21233261b_b	Unknown	3.10e4	N/A	2.62	15.1	N/A	8.855546e0
A21233262b_b	Unknown	3.10e4	N/A	2.62	11.9	N/A	8.855898e0
A21233263b_b	Unknown	4.27e4	N/A	2.62	17.3	N/A	1.225308e1
A21233264b_b	Unknown	8.35e4	N/A	2.63	27.3	N/A	2.417925e1
A21233265b_b	Unknown	3.08e4	N/A	2.63	10.7	N/A	8.786663e0
A21233266b_b	Unknown	4.45e4	N/A	2.62	13.2	N/A	1.278314e1
A21233267b_b	Unknown	3.41e4	N/A	2.63	8.3	N/A	9.763036e0

Compound name: Glycitin
Regression Equation: y = 3425.51416 x + 686.41018 (r = 0.99723) (weighting: 1 / x)

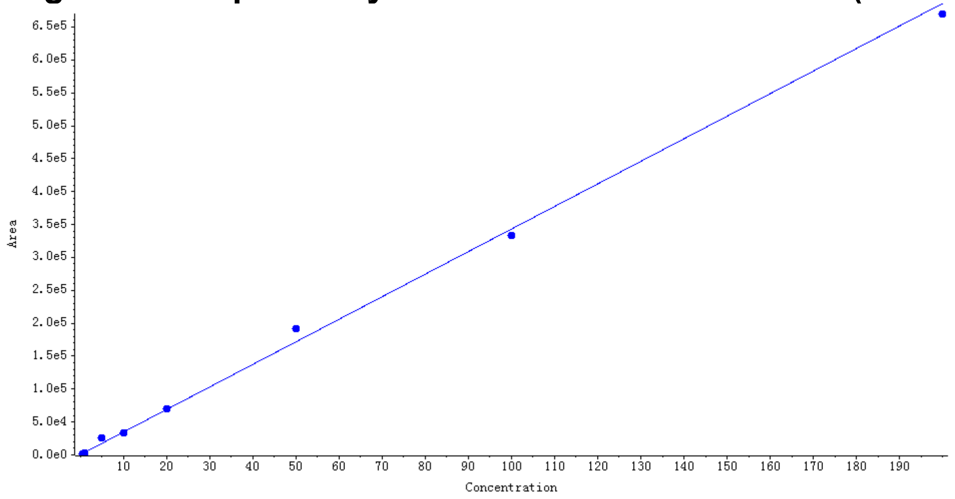

Peak Review

Blank
Glycitin AREA:N/A S/N:N/A

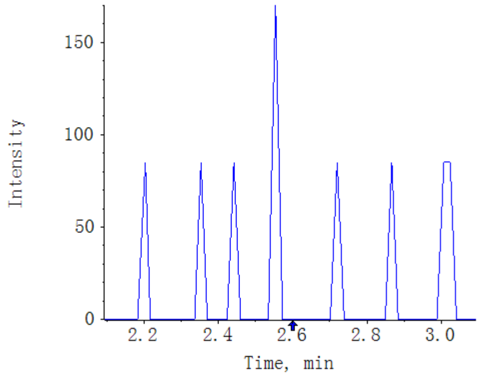

V1.0_MWMS_20211021_1
Glycitin AREA:5.09e5 S/N:135.1

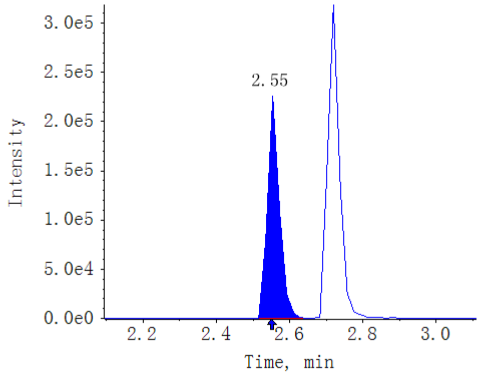

A21233250b_b
Glycitin AREA:N/A S/N:N/A

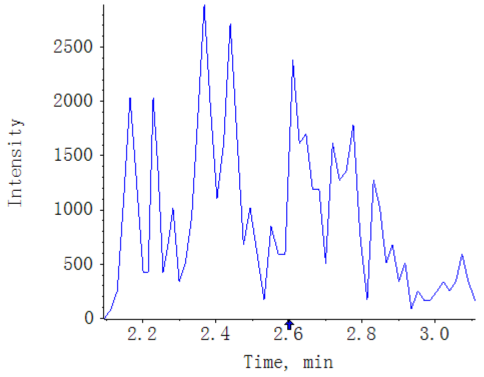

A21233251b_b
Glycitin AREA:N/A S/N:N/A

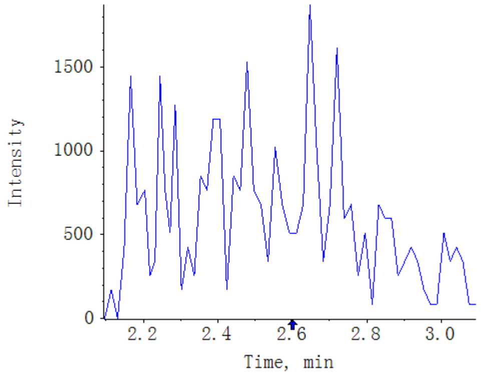

A21233252b_b
Glycitin AREA:N/A S/N:N/A

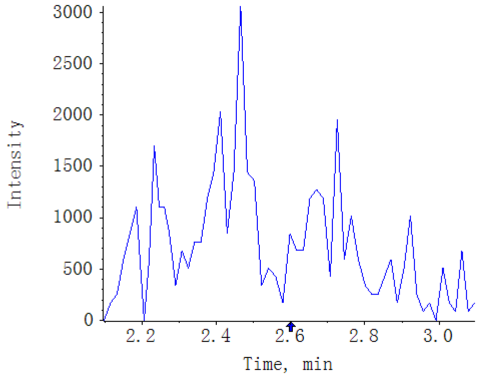

A21233253b_b
Glycitin AREA:1.83e4 S/N:13.9

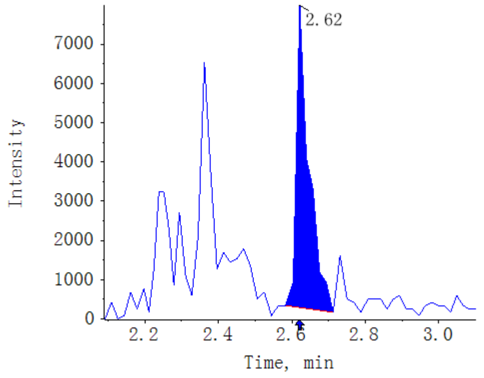

A21233254b_b
Glycitin AREA:1.20e4 S/N:8.3

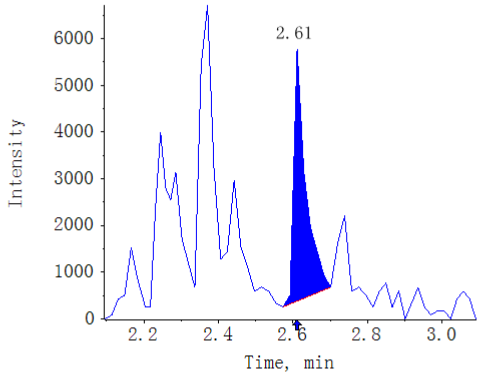

A21233255b_b
Glycitin AREA:1.27e4 S/N:8.0

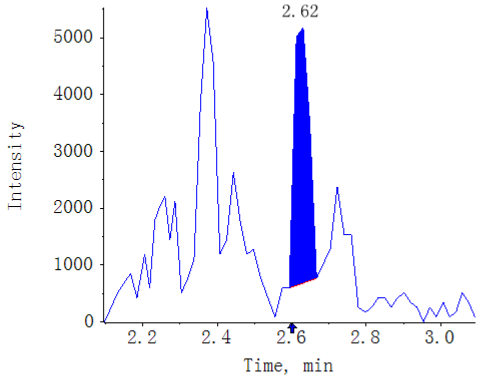

A21233256b_b
Glycitin AREA:1.09e4 S/N:5.0

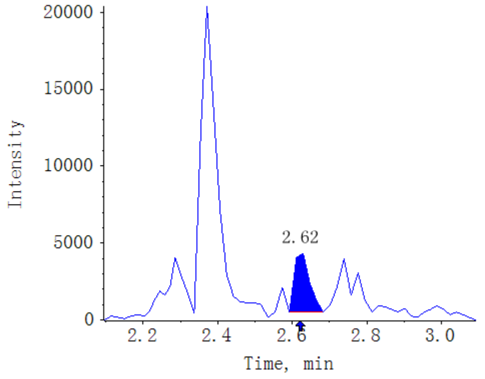

A21233257b_b
Glycitin AREA:1.13e4 S/N:6.8

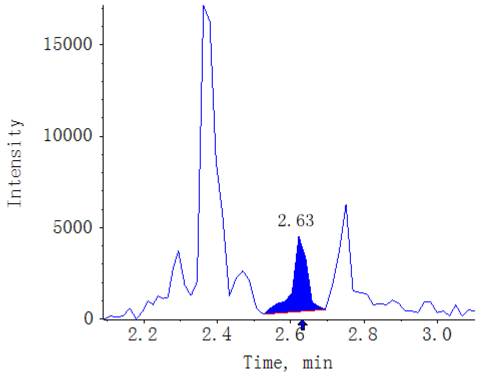

A21233258b_b
Glycitin AREA:1.20e4 S/N:4.8

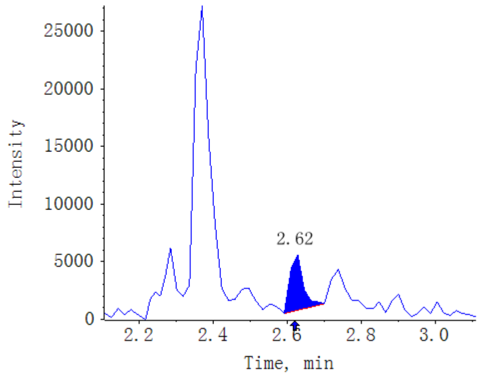

A21233259b_b
Glycitin AREA:9.63e3 S/N:5.7

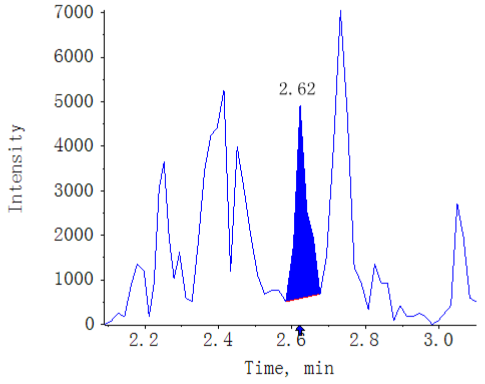

A21233260b_b
Glycitin AREA:1.07e4 S/N:7.7

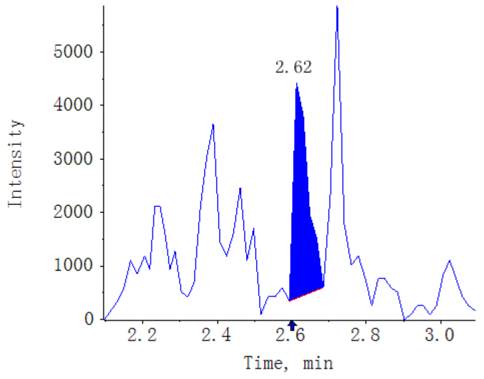

A21233261b_b
Glycitin AREA:3.10e4 S/N:15.1

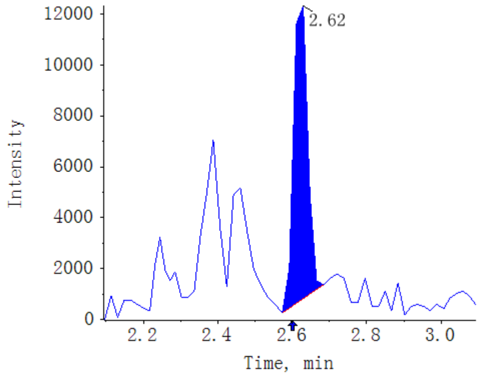

A21233262b_b
Glycitin AREA:3.10e4 S/N:11.9

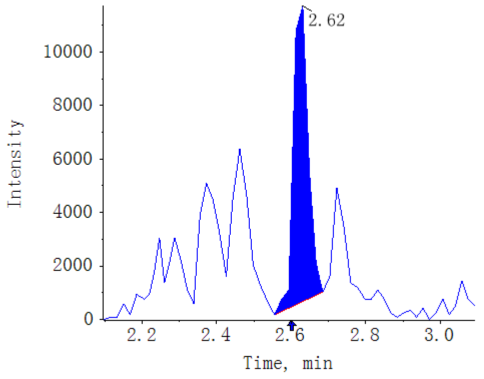

A21233263b_b
Glycitin AREA:4.27e4 S/N:17.3

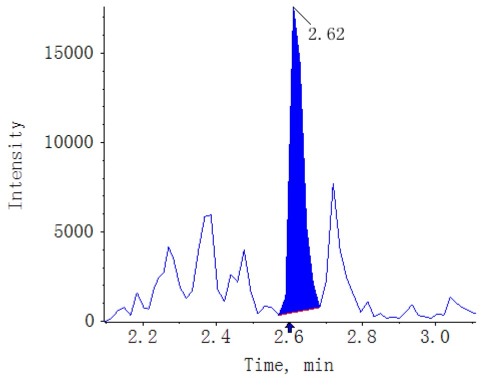

A21233264b_b
Glycitin AREA:8.35e4 S/N:27.3

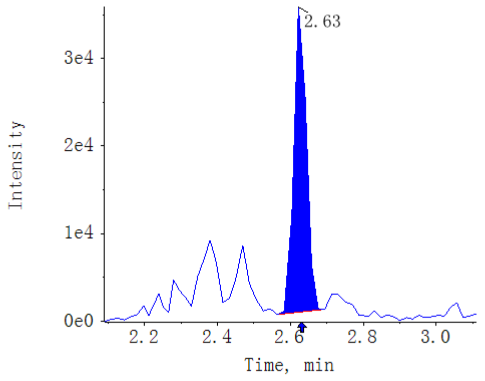

A21233265b_b
Glycitin AREA:3.08e4 S/N:10.7

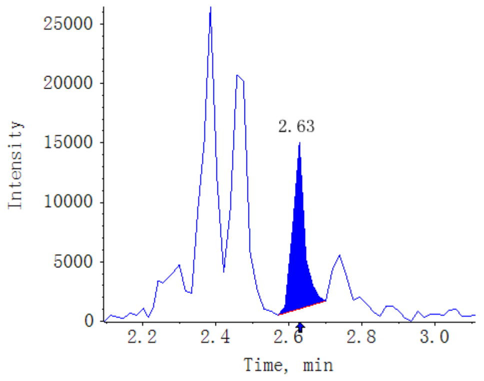

A21233266b_b
Glycitin AREA:4.45e4 S/N:13.2

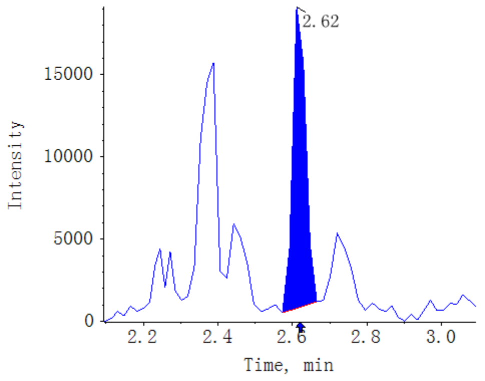

A21233267b_b
Glycitin AREA:3.41e4 S/N:8.3

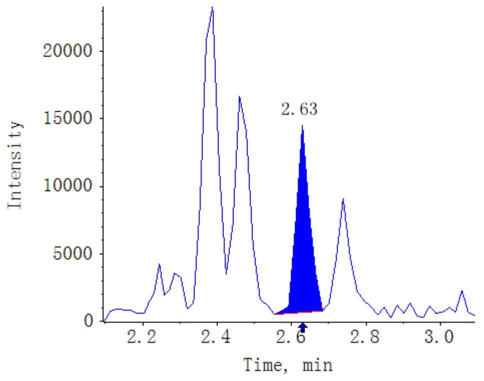

Result Table	MWXS-21-2101D-3_18_WH6500-5_A20-3_V1.0_TY_20211028	Algorithm Used	MQ4
Acquisition Method	Flavonoids_V1.0_WH6500-5_LT_20211025.dam	Instrument Name	QTRAP 6500+ Low Mass
Project	N/A	Analytes QTY	204:128

Compound name: Isomangiferin (421.1 / 301.0)

Sample Name	Sample Type	Area (cps)	Is Area (cps)	RT (min)	S/N	Target Conc	Calculated Conc.()
STD_0.5nM	Standard	4.74e3	N/A	2.32	23.8	0.5000	3.731473e-1
STD_1nM	Standard	9.37e3	N/A	2.31	32.3	1.0000	8.118249e-1
STD_5nM	Standard	7.63e4	N/A	2.31	102.0	5.0000	7.139207e0
STD_10nM	Standard	1.08e5	N/A	2.31	115.8	10.0000	1.011979e1
STD_20nM	Standard	2.12e5	N/A	2.31	135.3	20.0000	2.000826e1
STD_50nM	Standard	5.31e5	N/A	2.31	150.7	50.0000	5.020498e1
STD_100nM	Standard	1.08e6	N/A	2.32	144.9	100.0000	1.016667e2
STD_200nM	Standard	2.07e6	N/A	2.30	149.2	200.0000	1.961761e2
STD_500nM	Standard	N/A	N/A	N/A	N/A	500.0000	N/A
STD_1000nM	Standard	N/A	N/A	N/A	N/A	1000.0000	N/A
STD_2000nM	Standard	N/A	N/A	N/A	N/A	2000.0000	N/A
V1.0_MW_RQC1_20211018	Quality Control	N/A	N/A	N/A	N/A	0.0000	N/A
Blank	Unknown	N/A	N/A	N/A	N/A	N/A	N/A
V1.0_MWMS_20211021_1	Unknown	1.82e6	N/A	2.32	157.5	N/A	1.716883e2
MWXS212101D3_R1	Quality Control	1.78e6	N/A	2.31	145.9	0.0000	1.678804e2
MWXS212101D3_R2	Quality Control	1.84e6	N/A	2.31	172.8	0.0000	1.742429e2
MWXS212101D3_R3	Quality Control	1.85e6	N/A	2.31	149.6	0.0000	1.746694e2
A21233250b_b	Unknown	9.00e3	N/A	2.40	6.8	N/A	7.765562e-1
A21233251b_b	Unknown	7.71e3	N/A	2.40	6.1	N/A	6.544820e-1
A21233252b_b	Unknown	1.17e3	N/A	2.40	2.2	N/A	3.567472e-2
A21233253b_b	Unknown	2.57e3	N/A	2.40	3.3	N/A	1.680264e-1
A21233254b_b	Unknown	1.64e3	N/A	2.39	3.7	N/A	8.060954e-2
A21233255b_b	Unknown	3.62e3	N/A	2.40	3.3	N/A	2.675996e-1
A21233256b_b	Unknown	4.04e3	N/A	2.40	3.7	N/A	3.074384e-1
A21233257b_b	Unknown	5.90e3	N/A	2.41	5.8	N/A	4.832434e-1
A21233258b_b	Unknown	3.08e3	N/A	2.40	4.0	N/A	2.165061e-1
A21233259b_b	Unknown	5.94e3	N/A	2.40	6.2	N/A	4.871210e-1
A21233260b_b	Unknown	3.37e3	N/A	2.39	4.0	N/A	2.437807e-1
A21233261b_b	Unknown	7.86e3	N/A	2.40	7.2	N/A	6.685222e-1
A21233262b_b	Unknown	3.76e3	N/A	2.39	5.6	N/A	2.810973e-1
A21233263b_b	Unknown	6.83e3	N/A	2.39	5.1	N/A	5.708138e-1
A21233264b_b	Unknown	7.06e3	N/A	2.40	6.1	N/A	5.933336e-1
A21233265b_b	Unknown	6.05e3	N/A	2.40	6.4	N/A	4.975237e-1
A21233266b_b	Unknown	5.60e3	N/A	2.39	4.6	N/A	4.551344e-1
A21233267b_b	Unknown	9.63e3	N/A	2.41	8.7	N/A	8.362376e-1

Compound name: Isomangiferin
Regression Equation: y = 10569.52166 x + 792.17049 (r = 0.99853) (weighting: 1 / x)

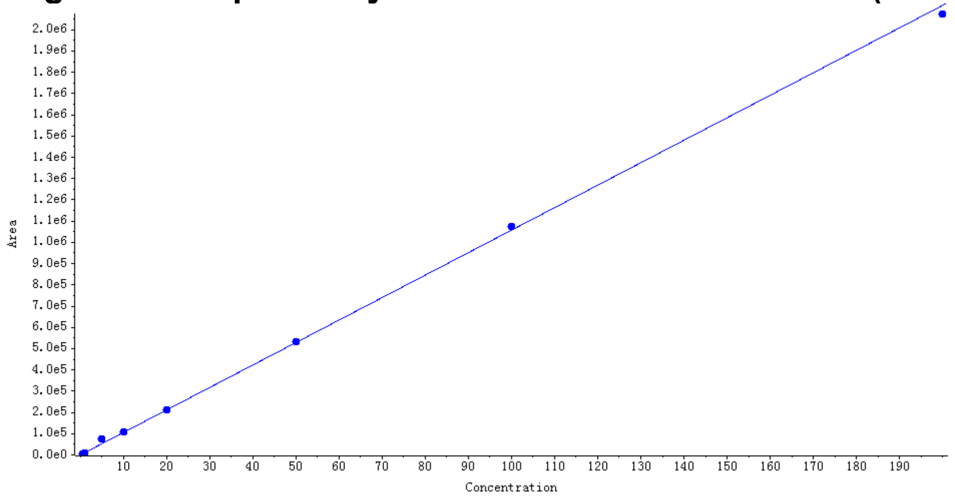

Peak Review

Blank
Isomangiferin AREA:N/A S/N:N/A

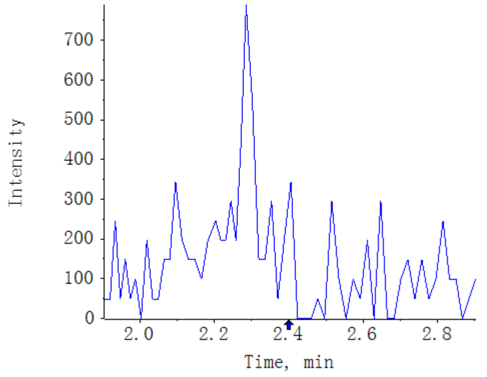

V1.0_MWMS_20211021_1
Isomangiferin AREA:1.82e6
S/N:157.5

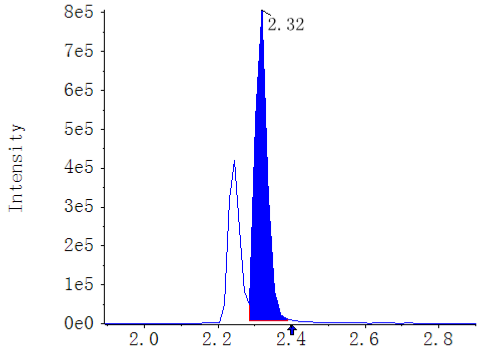

A21233250b_b
Isomangiferin AREA:9.00e3
S/N:6.8

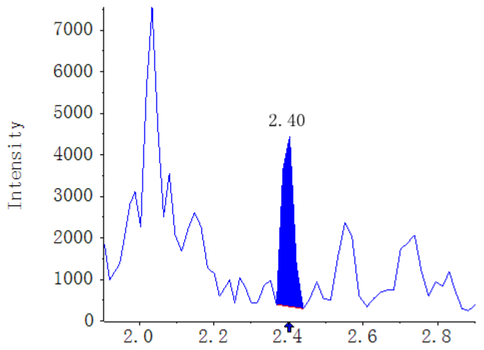

A21233251b_b
Isomangiferin AREA:7.71e3
S/N:6.1

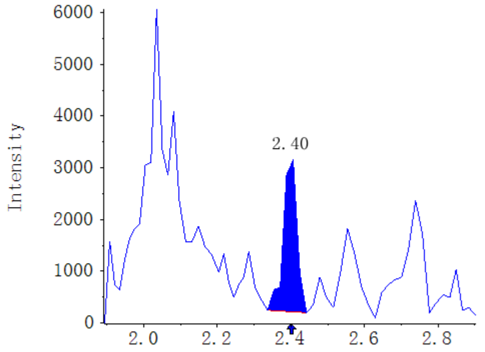

A21233252b_b
Isomangiferin AREA:1.17e3
S/N:2.2

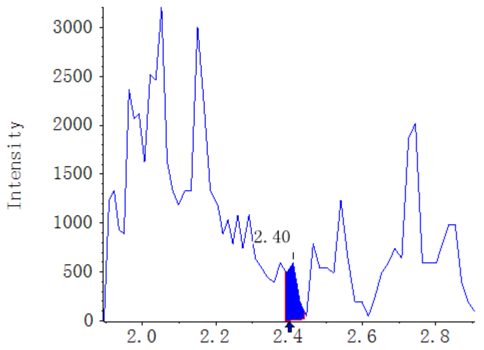

A21233253b_b
Isomangiferin AREA:2.57e3
S/N:3.3

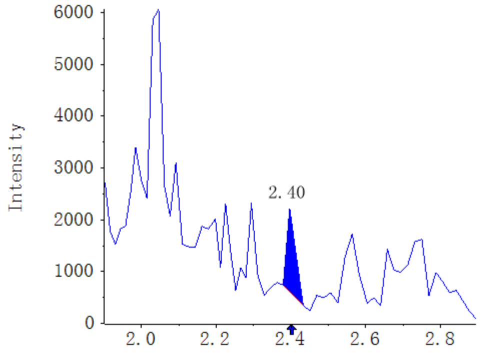

A21233254b_b
Isomangiferin AREA:1.64e3
S/N:3.7

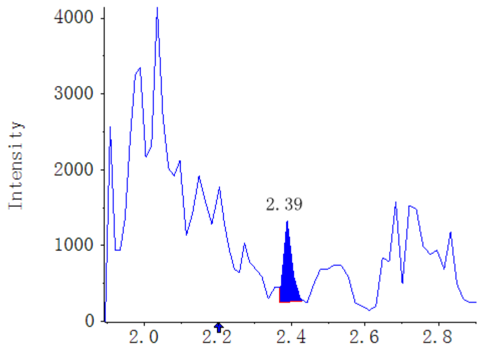

A21233255b_b
Isomangiferin AREA:3.62e3
S/N:3.3

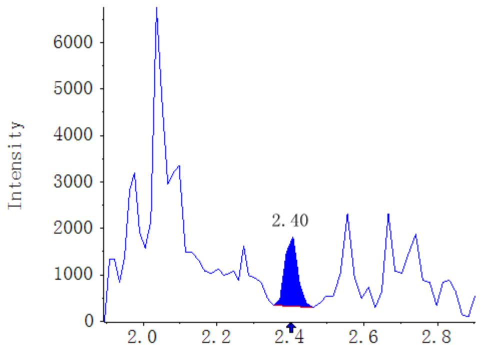

A21233256b_b
Isomangiferin AREA:4.04e3
S/N:3.7

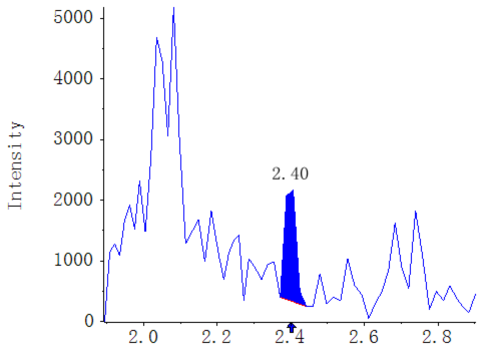

A21233257b_b
Isomangiferin AREA:5.90e3
S/N:5.8

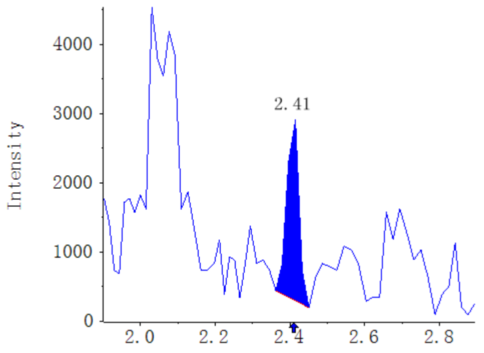

A21233258b_b
Isomangiferin AREA:3.08e3
S/N:4.0

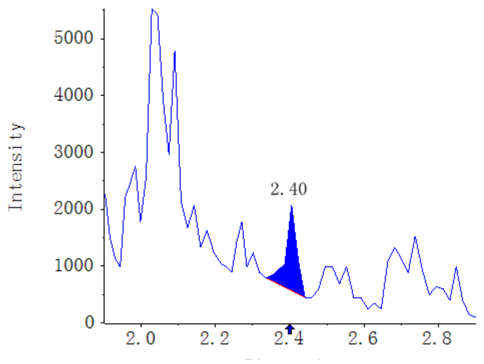

A21233259b_b
Isomangiferin AREA:5.94e3
S/N:6.2

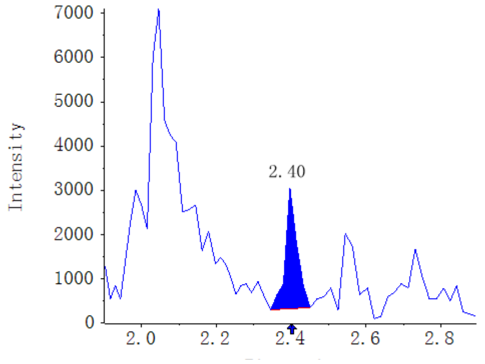

A21233260b_b
Isomangiferin AREA:3.37e3
S/N:4.0

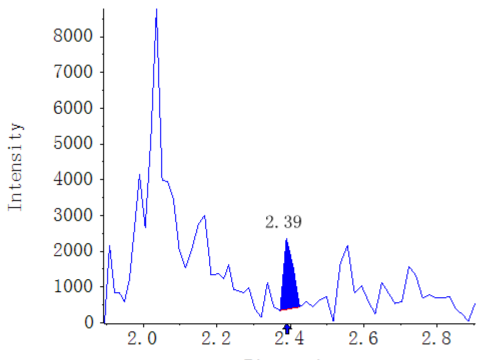

A21233261b_b
Isomangiferin AREA:7.86e3
S/N:7.2

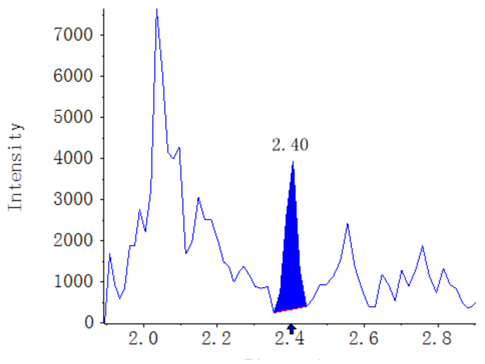

A21233262b_b
Isomangiferin AREA:3.76e3
S/N:5.6

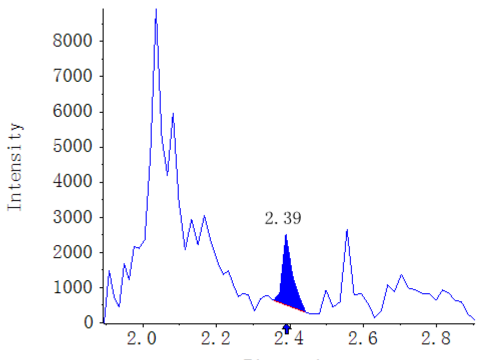

A21233263b_b
Isomangiferin AREA:6.83e3
S/N:5.1

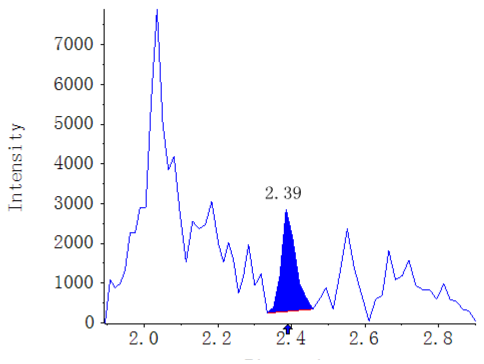

A21233264b_b
Isomangiferin AREA:7.06e3
S/N:6.1

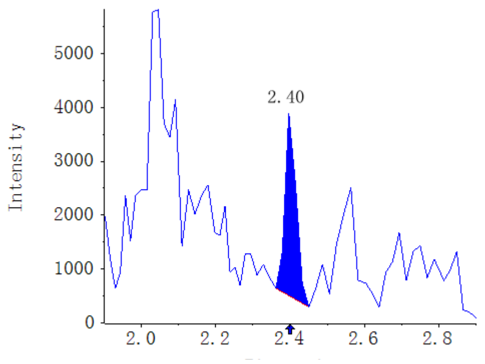

A21233265b_b
Isomangiferin AREA:6.05e3
S/N:6.4

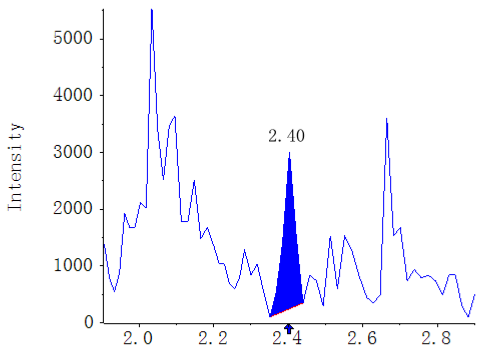

A21233266b_b
Isomangiferin AREA:5.60e3
S/N:4.6

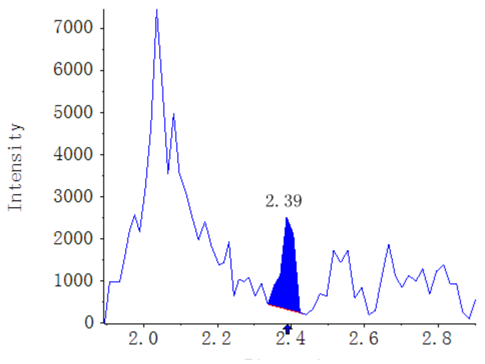

A21233267b_b
Isomangiferin AREA:9.63e3
S/N:8.7

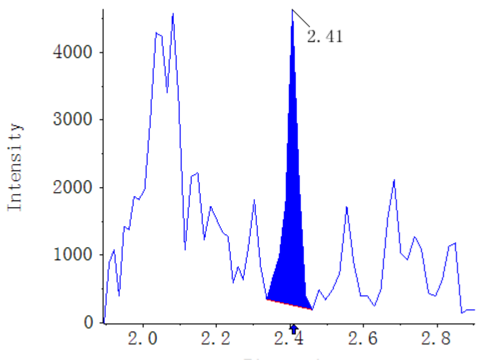

**Result Table**	MWXS-21-2101D-3_18_WH6500-5_A20-3_V1.0_TY_20211028	**Algorithm Used**	MQ4
**Acquisition Method**	Flavonoids_V1.0_WH6500-5_LT_20211025.dam	**Instrument Name**	QTRAP 6500+ Low Mass
**Project**	N/A	**Analytes QTY**	204:131

Compound name: Apigenin 7-glucoside (431.1 / 268.0)

Sample Name	Sample Type	Area (cps)	Is Area (cps)	RT (min)	S/N	Target Conc	Calculated Conc.()
STD_0.5nM	Standard	5.28e3	N/A	3.07	27.8	0.5000	3.125393e-1
STD_1nM	Standard	1.20e4	N/A	3.06	32.7	1.0000	9.837439e-1
STD_5nM	Standard	7.02e4	N/A	3.07	66.3	5.0000	6.835246e0
STD_10nM	Standard	9.70e4	N/A	3.07	122.1	10.0000	9.527789e0
STD_20nM	Standard	2.08e5	N/A	3.06	119.5	20.0000	2.064892e1
STD_50nM	Standard	5.33e5	N/A	3.07	100.8	50.0000	5.336594e1
STD_100nM	Standard	9.92e5	N/A	3.07	96.9	100.0000	9.949110e1
STD_200nM	Standard	1.95e6	N/A	3.06	123.0	200.0000	1.953347e2
STD_500nM	Standard	N/A	N/A	N/A	N/A	500.0000	N/A
STD_1000nM	Standard	N/A	N/A	N/A	N/A	1000.0000	N/A
STD_2000nM	Standard	N/A	N/A	N/A	N/A	2000.0000	N/A
V1.0_MW_RQC1_20211018	Quality Control	3.37e6	N/A	3.06	168.3	0.0000	3.383757e2
Blank	Unknown	N/A	N/A	N/A	N/A	N/A	N/A
V1.0_MWMS_20211021_1	Unknown	1.65e6	N/A	3.08	127.1	N/A	1.656165e2
MWXS212101D3_R1	Quality Control	1.70e6	N/A	3.08	106.0	0.0000	1.702638e2
MWXS212101D3_R2	Quality Control	1.62e6	N/A	3.08	127.7	0.0000	1.624150e2
MWXS212101D3_R3	Quality Control	1.66e6	N/A	3.08	129.0	0.0000	1.670893e2
A21233250b_b	Unknown	N/A	N/A	N/A	N/A	N/A	N/A
A21233251b_b	Unknown	N/A	N/A	N/A	N/A	N/A	N/A
A21233252b_b	Unknown	N/A	N/A	N/A	N/A	N/A	N/A
A21233253b_b	Unknown	N/A	N/A	N/A	N/A	N/A	N/A
A21233254b_b	Unknown	N/A	N/A	N/A	N/A	N/A	N/A
A21233255b_b	Unknown	N/A	N/A	N/A	N/A	N/A	N/A
A21233256b_b	Unknown	1.65e4	N/A	3.08	6.7	N/A	1.440254e0
A21233257b_b	Unknown	N/A	N/A	N/A	N/A	N/A	N/A
A21233258b_b	Unknown	1.81e4	N/A	3.08	12.6	N/A	1.604016e0
A21233259b_b	Unknown	N/A	N/A	N/A	N/A	N/A	N/A
A21233260b_b	Unknown	N/A	N/A	N/A	N/A	N/A	N/A
A21233261b_b	Unknown	N/A	N/A	N/A	N/A	N/A	N/A
A21233262b_b	Unknown	N/A	N/A	N/A	N/A	N/A	N/A
A21233263b_b	Unknown	N/A	N/A	N/A	N/A	N/A	N/A
A21233264b_b	Unknown	N/A	N/A	N/A	N/A	N/A	N/A
A21233265b_b	Unknown	2.06e4	N/A	3.08	6.4	N/A	1.850455e0
A21233266b_b	Unknown	8.74e3	N/A	3.07	5.8	N/A	6.601395e-1
A21233267b_b	Unknown	1.34e4	N/A	3.09	4.7	N/A	1.123645e0

Compound name: Apigenin 7-glucoside
Regression Equation: y = 9951.59186 x + 2168.77254 (r = 0.99847) (weighting: 1 / x)

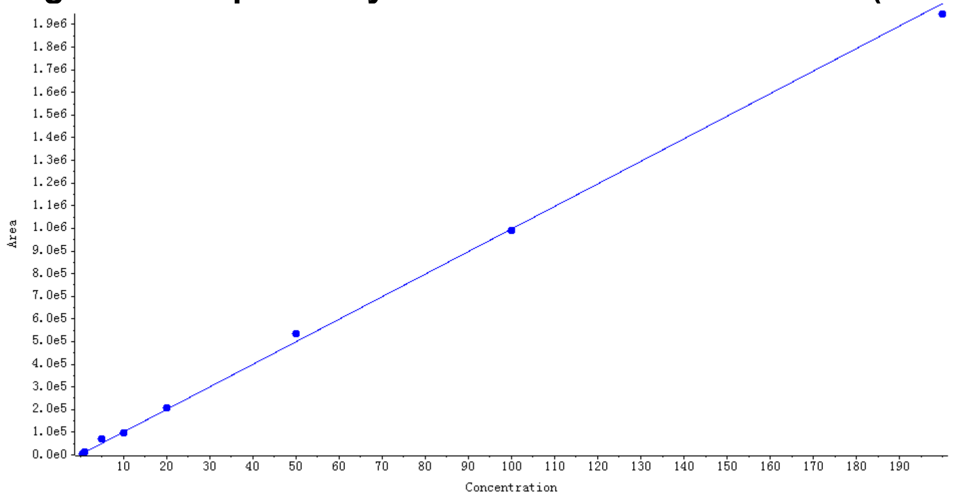

Peak Review

Blank
Apigenin 7-glucoside AREA:N/A
S/N:N/A

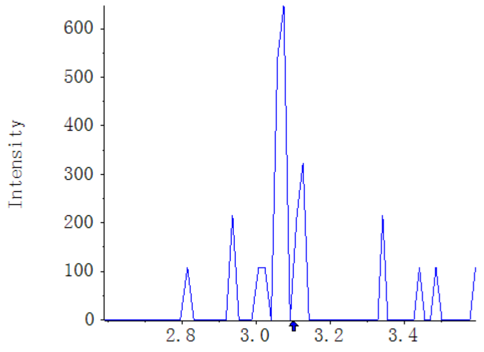

V1.0_MWMS_20211021_1
Apigenin 7-glucoside AREA:1.65e6
S/N:127.1

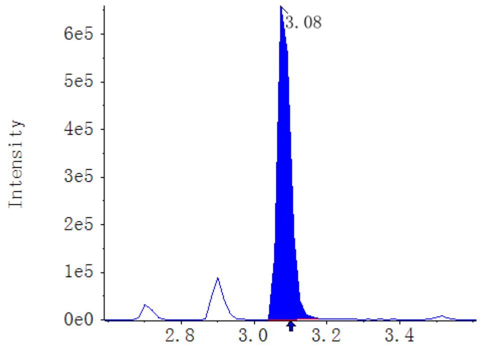

A21233250b_b
Apigenin 7-glucoside AREA:N/A
S/N:N/A

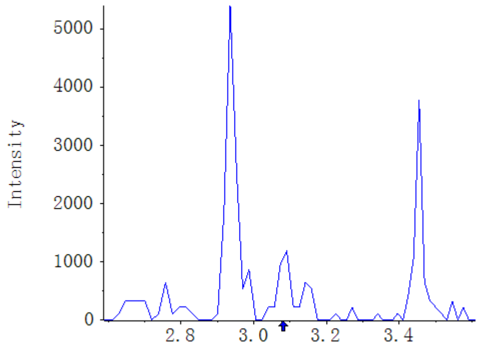

A21233251b_b
Apigenin 7-glucoside AREA:N/A
S/N:N/A

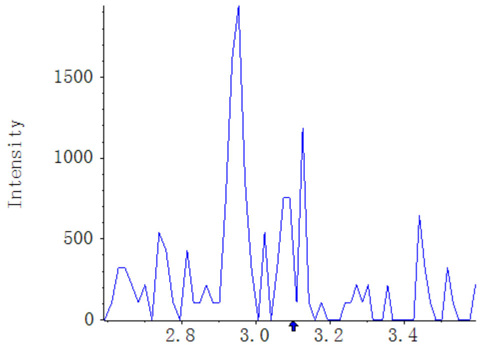

A21233252b_b
Apigenin 7-glucoside AREA:N/A
S/N:N/A

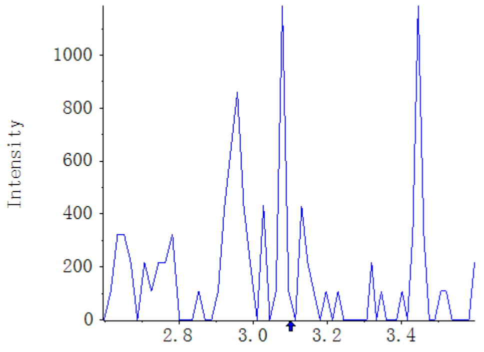

A21233253b_b
Apigenin 7-glucoside AREA:N/A
S/N:N/A

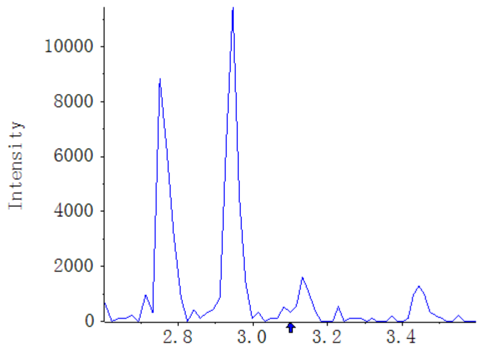

A21233254b_b
Apigenin 7-glucoside AREA:N/A
S/N:N/A

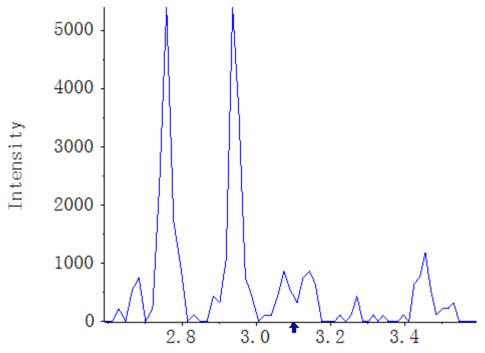

A21233255b_b
Apigenin 7-glucoside AREA:N/A
S/N:N/A

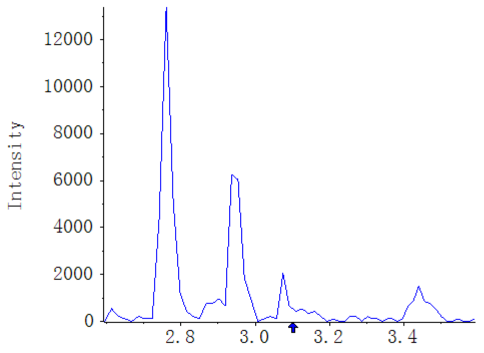

A21233256b_b
Apigenin 7-glucoside AREA:1.65e4
S/N:6.7

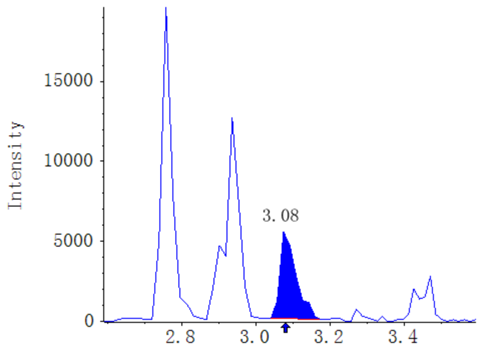

A21233257b_b
Apigenin 7-glucoside AREA:N/A
S/N:N/A

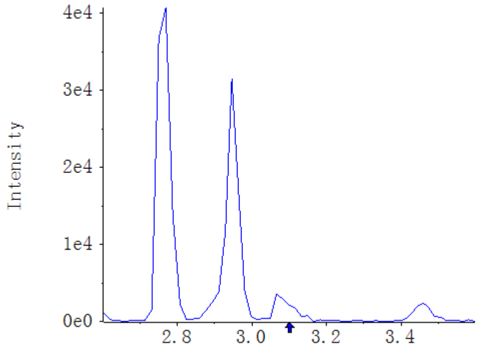

A21233258b_b
Apigenin 7-glucoside AREA:1.81e4
S/N:12.6

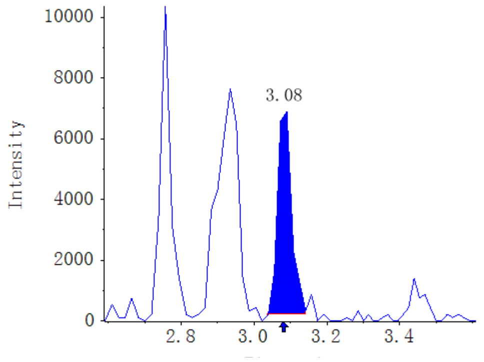

A21233259b_b
Apigenin 7-glucoside AREA:N/A
S/N:N/A

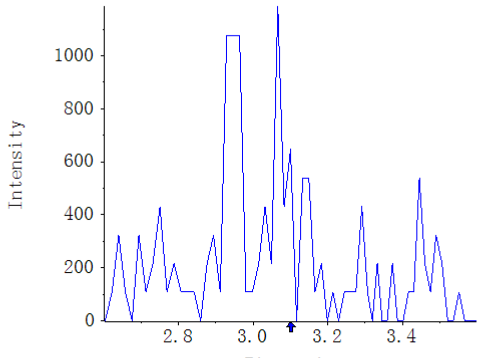

A21233260b_b
Apigenin 7-glucoside AREA:N/A
S/N:N/A

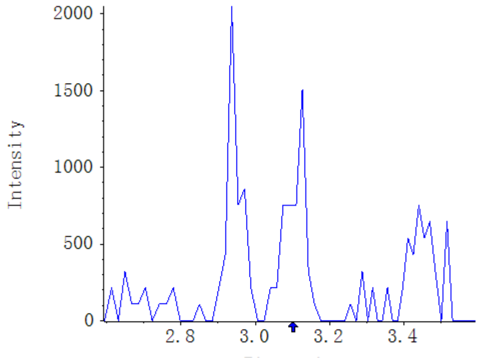

A21233261b_b
Apigenin 7-glucoside AREA:N/A
S/N:N/A

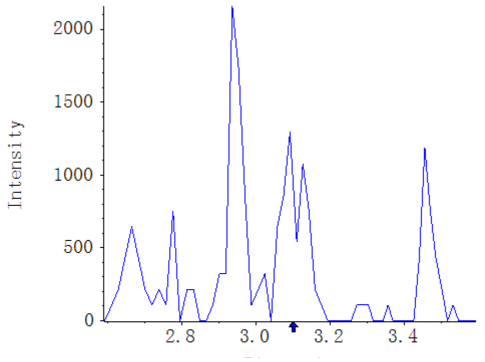

A21233262b_b
Apigenin 7-glucoside AREA:N/A
S/N:N/A

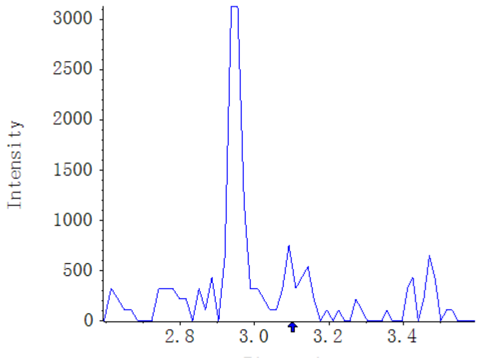

A21233263b_b
Apigenin 7-glucoside AREA:N/A
S/N:N/A

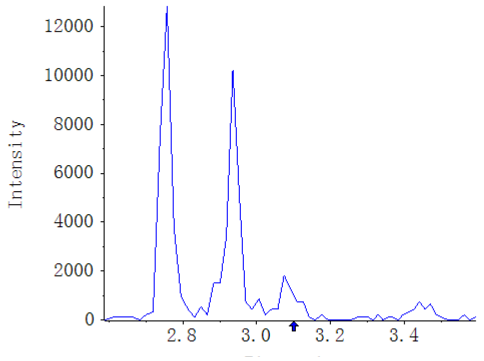

A21233264b_b
Apigenin 7-glucoside AREA:N/A
S/N:N/A

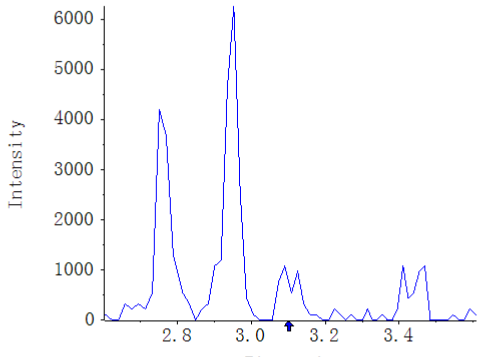

A21233265b_b
Apigenin 7-glucoside AREA:2.06e4
S/N:6.4

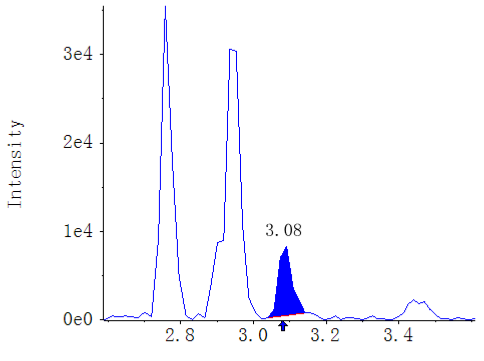

A21233266b_b
Apigenin 7-glucoside AREA:8.74e3
S/N:5.8

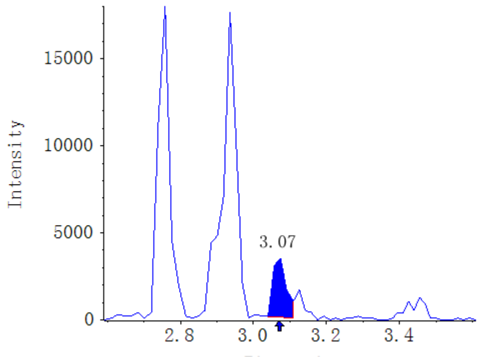

A21233267b_b
Apigenin 7-glucoside AREA:1.34e4
S/N:4.7

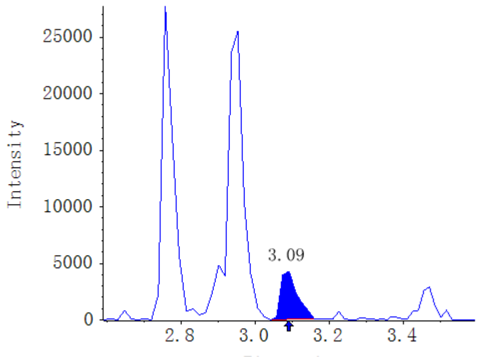

Result Table	MWXS-21-2101D-3_18_WH6500-5_A20-3_V1.0_TY_20211028	Algorithm Used	MQ4
Acquisition Method	Flavonoids_V1.0_WH6500-5_LT_20211025.dam	Instrument Name	QTRAP 6500+ Low Mass
Project	N/A	Analytes QTY	204:137

Compound name: Quercetin 3-O-(6"-galloyl)-β-D-galactopyranoside (615.1 / 463.1)

Sample Name	Sample Type	Area (cps)	Is Area (cps)	RT (min)	S/N	Target Conc	Calculated Conc.()
STD_0.5nM	Standard	3.32e3	N/A	2.62	20.9	0.5000	5.157168e-1
STD_1nM	Standard	4.24e3	N/A	2.62	22.3	1.0000	6.547170e-1
STD_5nM	Standard	4.75e4	N/A	2.61	134.0	5.0000	7.185440e0
STD_10nM	Standard	6.51e4	N/A	2.61	141.3	10.0000	9.839159e0
STD_20nM	Standard	1.20e5	N/A	2.61	230.5	20.0000	1.806653e1
STD_50nM	Standard	3.36e5	N/A	2.62	269.6	50.0000	5.070522e1
STD_100nM	Standard	6.33e5	N/A	2.62	258.4	100.0000	9.554966e1
STD_200nM	Standard	1.35e6	N/A	2.61	337.4	200.0000	2.039835e2
STD_500nM	Standard	N/A	N/A	N/A	N/A	500.0000	N/A
STD_1000nM	Standard	N/A	N/A	N/A	N/A	1000.0000	N/A
STD_2000nM	Standard	N/A	N/A	N/A	N/A	2000.0000	N/A
V1.0_MW_RQC1_20211018	Quality Control	5.19e3	N/A	2.61	9.4	0.0000	7.973028e-1
Blank	Unknown	N/A	N/A	N/A	N/A	N/A	N/A
V1.0_MWMS_20211021_1	Unknown	1.19e6	N/A	2.62	293.0	N/A	1.796550e2
MWXS212101D3_R1	Quality Control	1.19e6	N/A	2.61	290.5	0.0000	1.796615e2
MWXS212101D3_R2	Quality Control	1.11e6	N/A	2.62	247.3	0.0000	1.675930e2
MWXS212101D3_R3	Quality Control	1.20e6	N/A	2.62	242.5	0.0000	1.818460e2
A21233250b_b	Unknown	3.53e3	N/A	2.62	16.4	N/A	5.476120e-1
A21233251b_b	Unknown	N/A	N/A	N/A	N/A	N/A	N/A
A21233252b_b	Unknown	N/A	N/A	N/A	N/A	N/A	N/A
A21233253b_b	Unknown	N/A	N/A	N/A	N/A	N/A	N/A
A21233254b_b	Unknown	N/A	N/A	N/A	N/A	N/A	N/A
A21233255b_b	Unknown	N/A	N/A	N/A	N/A	N/A	N/A
A21233256b_b	Unknown	N/A	N/A	N/A	N/A	N/A	N/A
A21233257b_b	Unknown	N/A	N/A	N/A	N/A	N/A	N/A
A21233258b_b	Unknown	N/A	N/A	N/A	N/A	N/A	N/A
A21233259b_b	Unknown	N/A	N/A	N/A	N/A	N/A	N/A
A21233260b_b	Unknown	N/A	N/A	N/A	N/A	N/A	N/A
A21233261b_b	Unknown	N/A	N/A	N/A	N/A	N/A	N/A
A21233262b_b	Unknown	N/A	N/A	N/A	N/A	N/A	N/A
A21233263b_b	Unknown	N/A	N/A	N/A	N/A	N/A	N/A
A21233264b_b	Unknown	N/A	N/A	N/A	N/A	N/A	N/A
A21233265b_b	Unknown	N/A	N/A	N/A	N/A	N/A	N/A
A21233266b_b	Unknown	N/A	N/A	N/A	N/A	N/A	N/A
A21233267b_b	Unknown	N/A	N/A	N/A	N/A	N/A	N/A

Compound name: Quercetin 3-O-(6''-galloyl)-β-D-galactopyranoside
Regression Equation: y = 6624.18579 x + -95.36664 (r = 0.99790) (weighting: 1 / x)

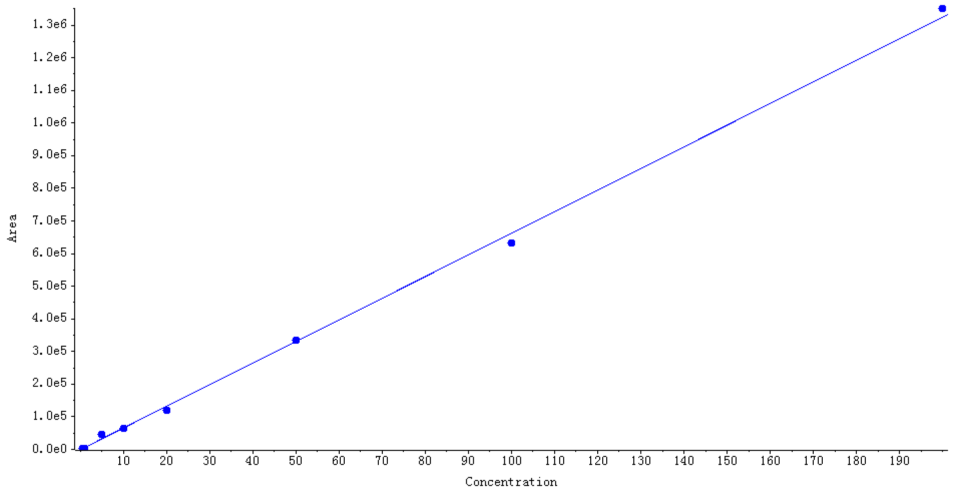

Peak Review

Blank
Quercetin 3-O-(6''-galloyl)- β -D-galactopyranoside AREA:N/A
S/N:N/A

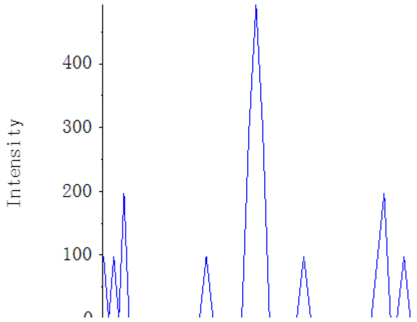

V1.0_MWMS_20211021_1
Quercetin 3-O-(6''-galloyl)- β -D-galactopyranoside AREA:1.19e6
S/N:293.0

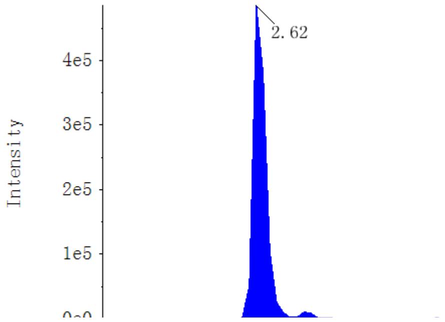

A21233250b_b
Quercetin 3-O-(6''-galloyl)- β -D-galactopyranoside AREA:3.53e3
S/N:16.4

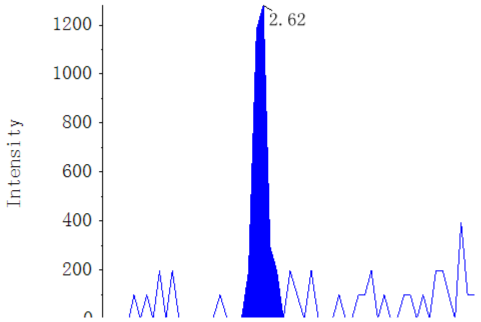

A21233251b_b
Quercetin 3-O-(6''-galloyl)- β -D-galactopyranoside AREA:N/A
S/N:N/A

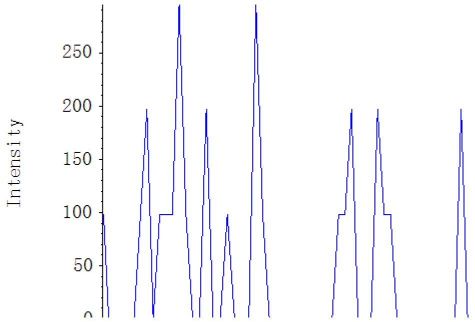

A21233252b_b
Quercetin 3-O-(6''-galloyl)- β -D-galactopyranoside AREA:N/A
S/N:N/A

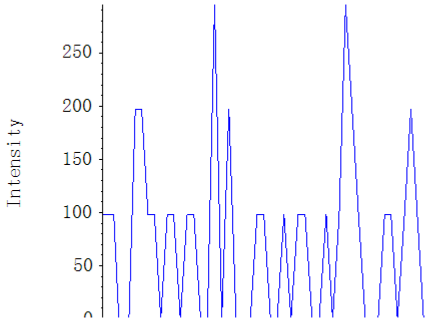

A21233253b_b
Quercetin 3-O-(6''-galloyl)- β -D-galactopyranoside AREA:N/A
S/N:N/A

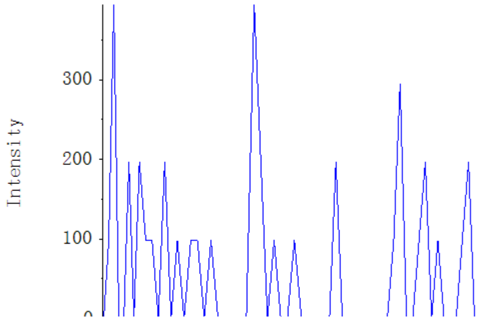

A21233254b_b
Quercetin 3-O-(6''-galloyl)- β -D-galactopyranoside AREA:N/A
S/N:N/A

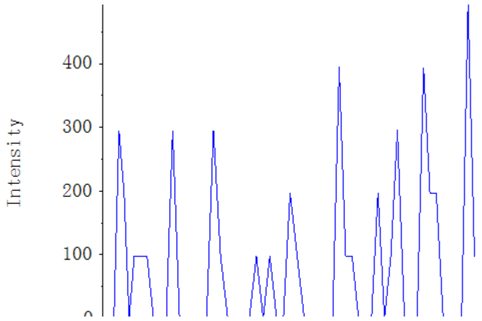

A21233255b_b
Quercetin 3-O-(6''-galloyl)- β -D-galactopyranoside AREA:N/A
S/N:N/A

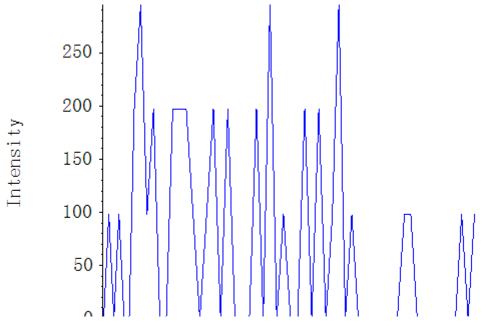

A21233256b_b
Quercetin 3-O-(6''-galloyl)- β -D-galactopyranoside AREA:N/A
S/N:N/A

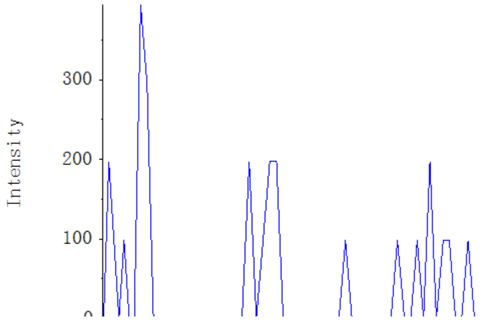

A21233257b_b
Quercetin 3-O-(6''-galloyl)- β -D-galactopyranoside AREA:N/A
S/N:N/A

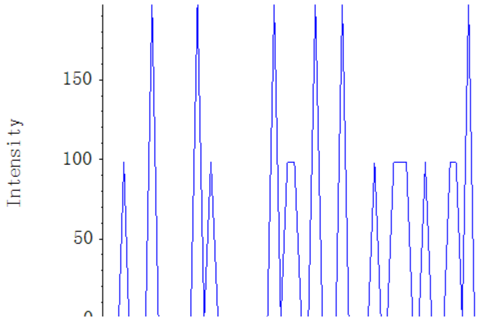

A21233258b_b
Quercetin 3-O-(6''-galloyl)-β-D-galactopyranoside AREA:N/A S/N:N/A

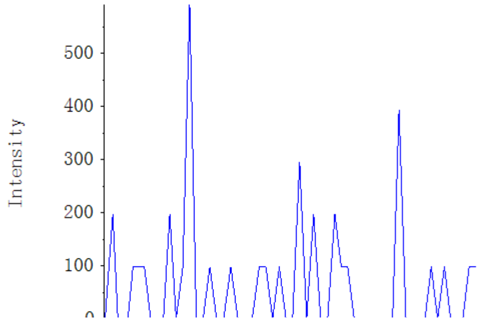

A21233259b_b
Quercetin 3-O-(6''-galloyl)-β-D-galactopyranoside AREA:N/A S/N:N/A

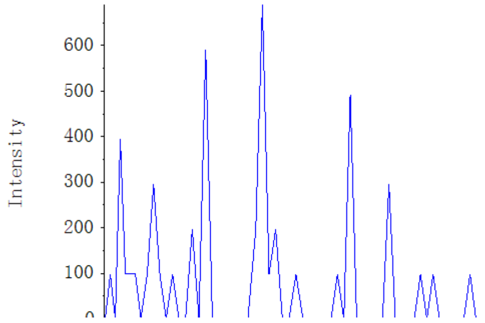

A21233260b_b
Quercetin 3-O-(6''-galloyl)-β-D-galactopyranoside AREA:N/A S/N:N/A

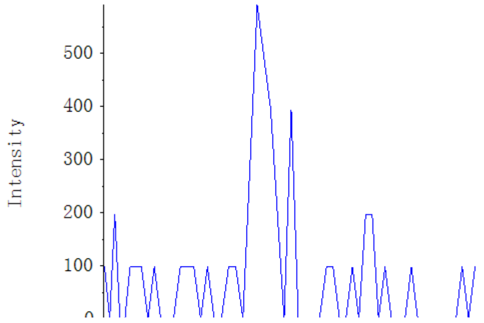

A21233261b_b
Quercetin 3-O-(6''-galloyl)-β-D-galactopyranoside AREA:N/A S/N:N/A

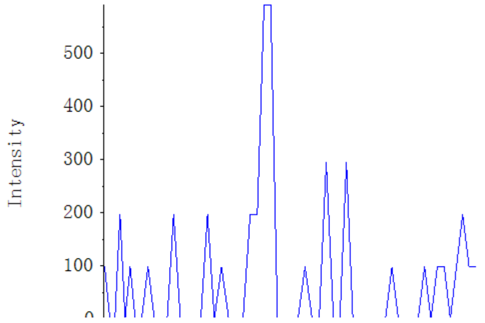

A21233262b_b
Quercetin 3-O-(6''-galloyl)-β-D-galactopyranoside AREA:N/A S/N:N/A

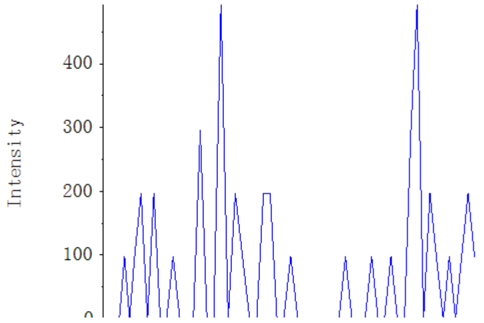

A21233263b_b
Quercetin 3-O-(6''-galloyl)-β-D-galactopyranoside AREA:N/A S/N:N/A

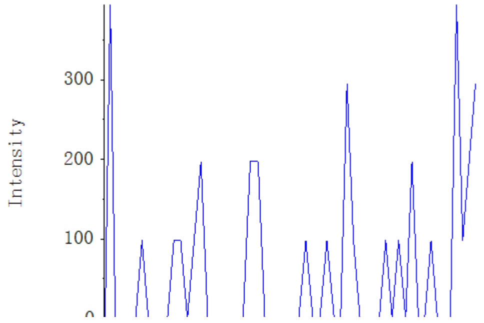

A21233264b_b
Quercetin 3-O-(6''-galloyl)-β-D-galactopyranoside AREA:N/A S/N:N/A

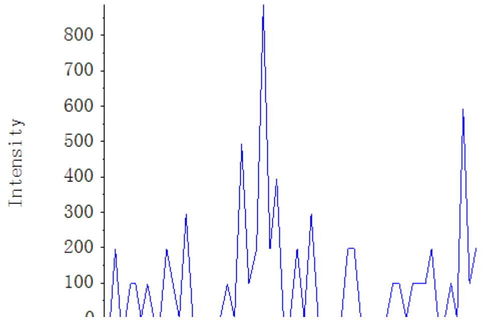

A21233265b_b
Quercetin 3-O-(6''-galloyl)-β-D-galactopyranoside AREA:N/A S/N:N/A

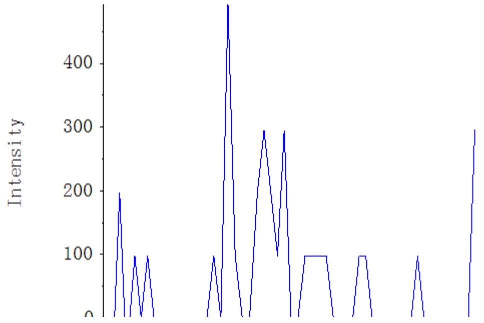

A21233266b_b
Quercetin 3-O-(6''-galloyl)-β-D-galactopyranoside AREA:N/A S/N:N/A

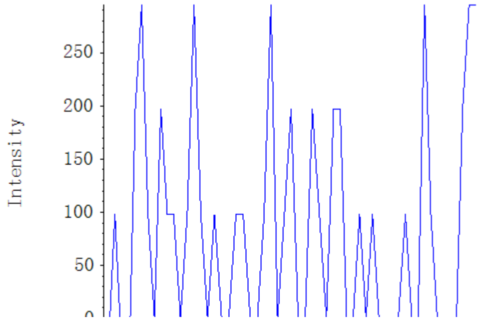

A21233267b_b
Quercetin 3-O-(6''-galloyl)-β-D-galactopyranoside AREA:N/A S/N:N/A

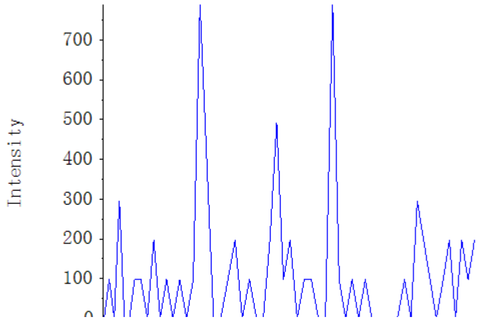

Result Table	MWXS-21-2101D-3_18_WH6500-5_A20-3_V1.0_TY_20211028	Algorithm Used	MQ4
Acquisition Method	Flavonoids_V1.0_WH6500-5_LT_20211025.dam	Instrument Name	QTRAP 6500+ Low Mass
Project	N/A	Analytes QTY	204:143

Compound name: Naringenin-7-glucoside (433.1 / 271.1)

Sample Name	Sample Type	Area (cps)	Is Area (cps)	RT (min)	S/N	Target Conc	Calculated Conc.()
STD_0.5nM	Standard	3.38e3	N/A	3.12	13.1	0.5000	3.574036e-1
STD_1nM	Standard	8.89e3	N/A	3.11	24.9	1.0000	9.909569e-1
STD_5nM	Standard	6.02e4	N/A	3.11	87.2	5.0000	6.889699e0
STD_10nM	Standard	8.14e4	N/A	3.11	99.3	10.0000	9.327238e0
STD_20nM	Standard	1.75e5	N/A	3.11	141.8	20.0000	2.003827e1
STD_50nM	Standard	4.35e5	N/A	3.11	138.5	50.0000	5.001533e1
STD_100nM	Standard	8.48e5	N/A	3.12	157.3	100.0000	9.738928e1
STD_200nM	Standard	1.75e6	N/A	3.11	147.6	200.0000	2.014918e2
STD_500nM	Standard	N/A	N/A	N/A	N/A	500.0000	N/A
STD_1000nM	Standard	N/A	N/A	N/A	N/A	1000.0000	N/A
STD_2000nM	Standard	N/A	N/A	N/A	N/A	2000.0000	N/A
V1.0_MW_RQC1_20211018	Quality Control	7.12e5	N/A	3.15	7.0	0.0000	8.177172e1
Blank	Unknown	N/A	N/A	N/A	N/A	N/A	N/A
V1.0_MWMS_20211021_1	Unknown	1.35e6	N/A	3.12	181.9	N/A	1.550653e2
MWXS212101D3_R1	Quality Control	1.34e6	N/A	3.12	158.3	0.0000	1.534507e2
MWXS212101D3_R2	Quality Control	1.35e6	N/A	3.12	190.6	0.0000	1.546317e2
MWXS212101D3_R3	Quality Control	1.32e6	N/A	3.12	207.7	0.0000	1.516821e2
A21233250b_b	Unknown	1.38e5	N/A	3.12	5.4	N/A	1.578425e1
A21233251b_b	Unknown	2.81e4	N/A	3.11	3.2	N/A	3.195797e0
A21233252b_b	Unknown	2.02e4	N/A	3.12	1.4	N/A	2.289951e0
A21233253b_b	Unknown	2.07e6	N/A	3.14	9.1	N/A	2.381581e2
A21233254b_b	Unknown	1.45e6	N/A	3.14	10.4	N/A	1.662930e2
A21233255b_b	Unknown	2.68e6	N/A	3.14	10.1	N/A	3.080498e2
A21233256b_b	Unknown	2.85e6	N/A	3.13	8.0	N/A	3.275771e2
A21233257b_b	Unknown	4.68e6	N/A	3.14	8.4	N/A	5.374901e2
A21233258b_b	Unknown	2.29e6	N/A	3.13	8.6	N/A	2.629950e2
A21233259b_b	Unknown	4.43e4	N/A	3.12	2.2	N/A	5.060799e0
A21233260b_b	Unknown	1.37e4	N/A	3.12	1.2	N/A	1.539768e0
A21233261b_b	Unknown	5.41e4	N/A	3.12	1.5	N/A	6.186214e0
A21233262b_b	Unknown	3.51e5	N/A	3.14	4.1	N/A	4.026867e1
A21233263b_b	Unknown	1.88e6	N/A	3.13	6.0	N/A	2.161953e2
A21233264b_b	Unknown	1.20e6	N/A	3.14	5.3	N/A	1.380269e2
A21233265b_b	Unknown	3.12e6	N/A	3.14	4.9	N/A	3.586214e2
A21233266b_b	Unknown	2.68e6	N/A	3.13	6.8	N/A	3.082297e2
A21233267b_b	Unknown	2.99e6	N/A	3.12	7.8	N/A	3.434060e2

Compound name: Naringenin-7-glucoside
Regression Equation: y = 8700.91348 x + 266.54093 (r = 0.99881) (weighting: 1 / x)

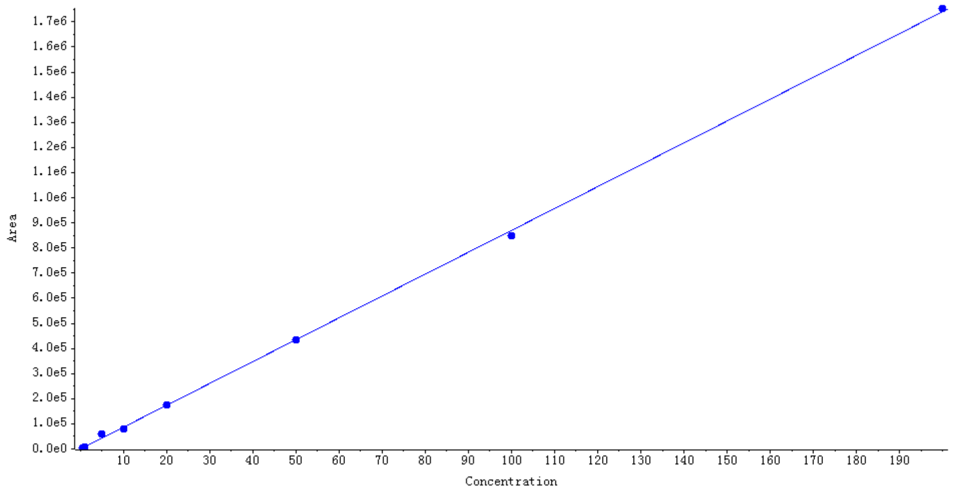

Peak Review

Blank
Naringenin-7-glucoside AREA:N/A
S/N:N/A

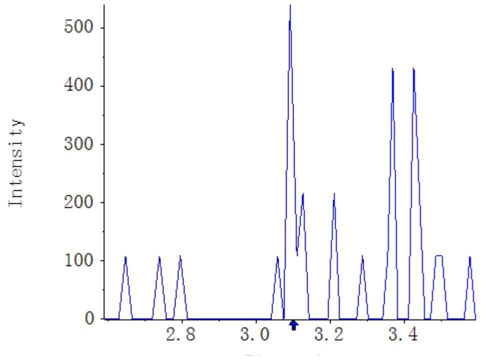

V1.0_MWMS_20211021_1
Naringenin-7-glucoside
AREA:1.35e6 S/N:181.9

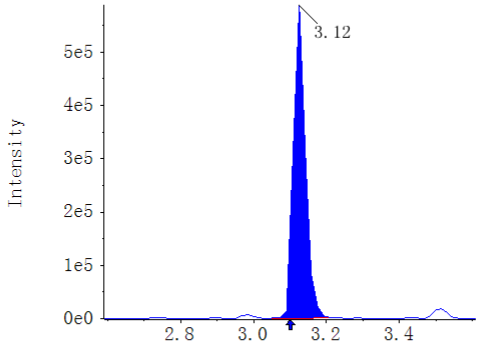

A21233250b_b
Naringenin-7-glucoside
AREA:1.38e5 S/N:5.4

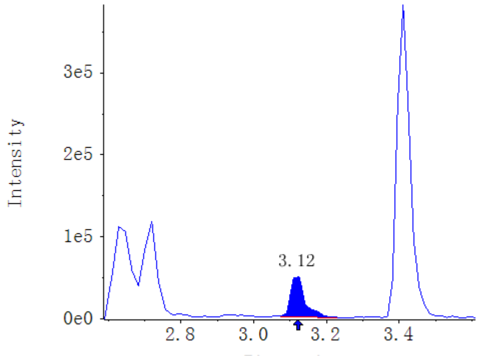

A21233251b_b
Naringenin-7-glucoside
AREA:2.81e4 S/N:3.2

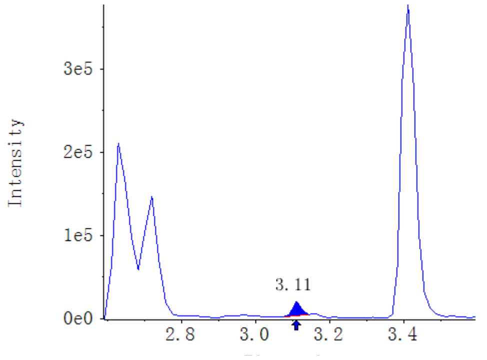

A21233252b_b
Naringenin-7-glucoside
AREA:2.02e4 S/N:1.4

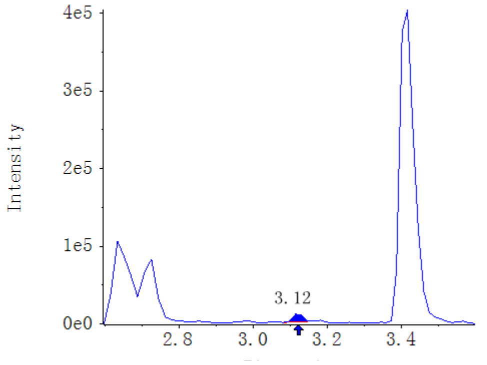

A21233253b_b
Naringenin-7-glucoside
AREA:2.07e6 S/N:9.1

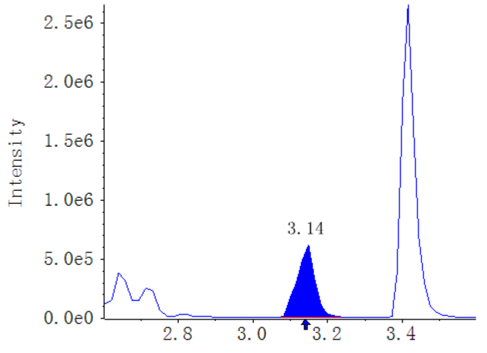

A21233254b_b
Naringenin-7-glucoside
AREA:1.45e6 S/N:10.4

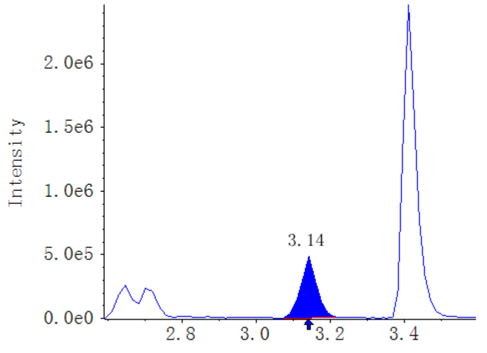

A21233255b_b
Naringenin-7-glucoside
AREA:2.68e6 S/N:10.1

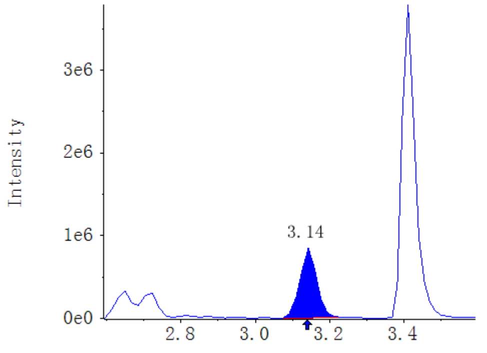

A21233256b_b
Naringenin-7-glucoside
AREA:2.85e6 S/N:8.0

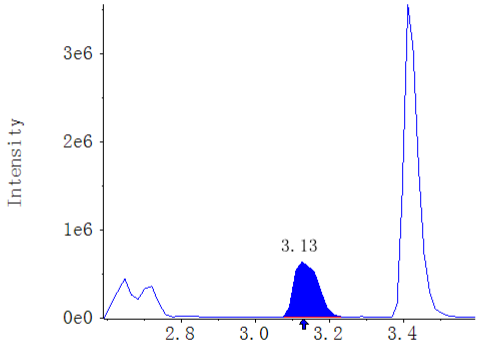

A21233257b_b
Naringenin-7-glucoside
AREA:4.68e6 S/N:8.4

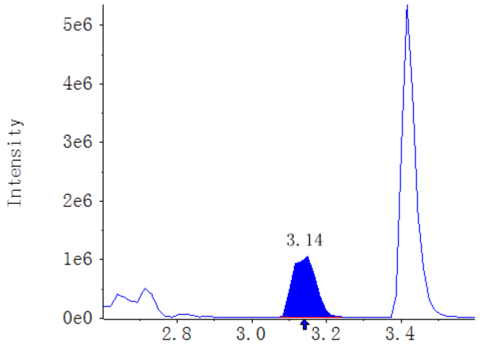

A21233258b_b
Naringenin-7-glucoside
AREA:2.29e6 S/N:8.6

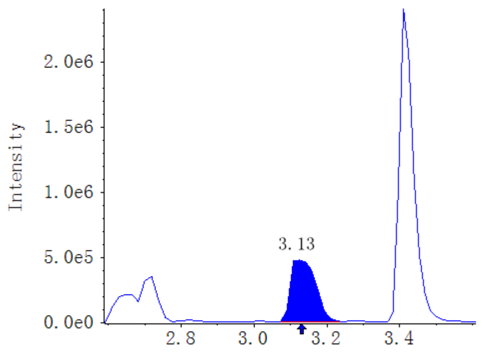

A21233259b_b
Naringenin-7-glucoside
AREA:4.43e4 S/N:2.2

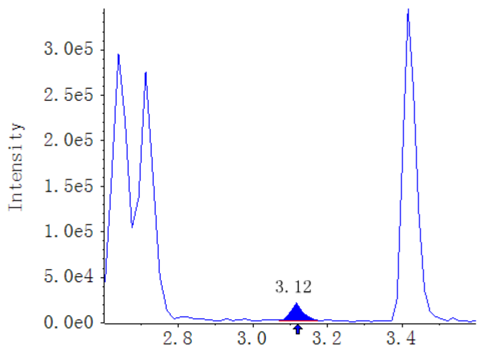

A21233260b_b
Naringenin-7-glucoside
AREA:1.37e4 S/N:1.2

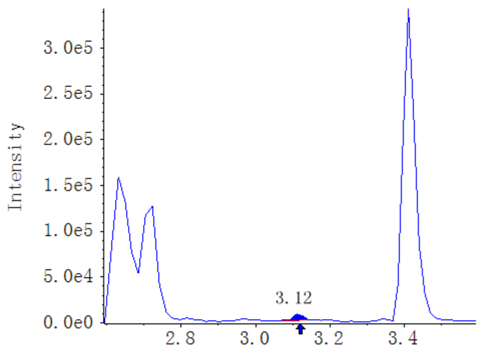

A21233261b_b
Naringenin-7-glucoside
AREA:5.41e4 S/N:1.5

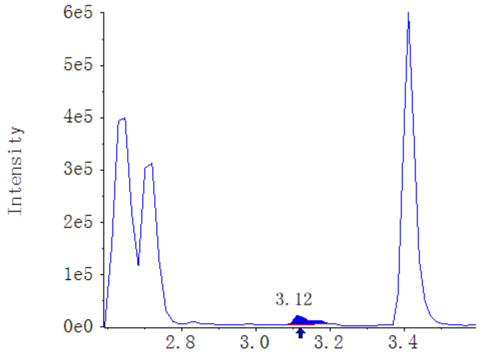

A21233262b_b
Naringenin-7-glucoside
AREA:3.51e5 S/N:4.1

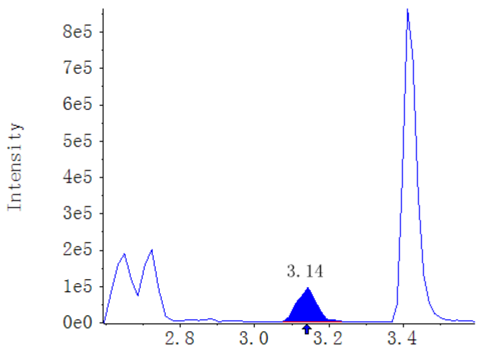

A21233263b_b
Naringenin-7-glucoside
AREA:1.88e6 S/N:6.0

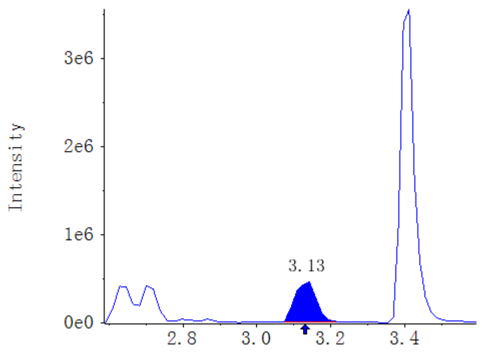

A21233264b_b
Naringenin-7-glucoside
AREA:1.20e6 S/N:5.3

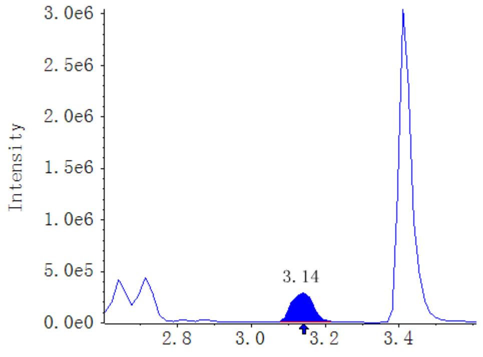

A21233265b_b
Naringenin-7-glucoside
AREA:3.12e6 S/N:4.9

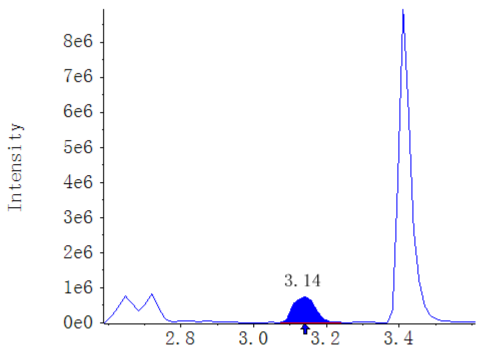

A21233266b_b
Naringenin-7-glucoside
AREA:2.68e6 S/N:6.8

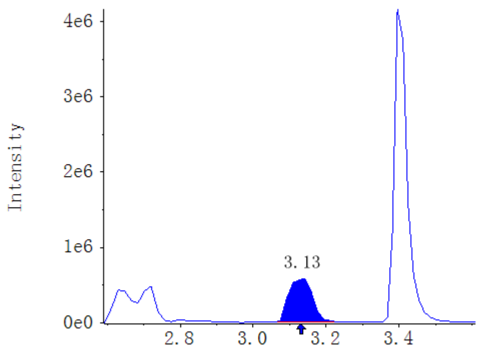

A21233267b_b
Naringenin-7-glucoside
AREA:2.99e6 S/N:7.8

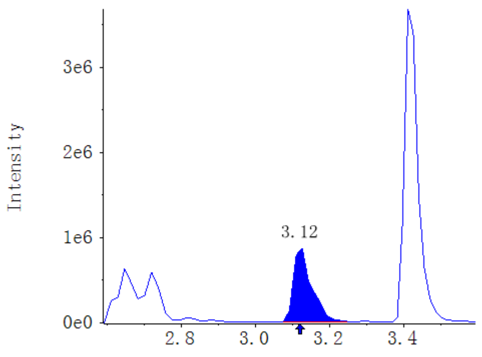

Result Table	MWXS-21-2101D-3_18_WH6500-5_A20-3_V1.0_TY_20211028	Algorithm Used	MQ4
Acquisition Method	Flavonoids_V1.0_WH6500-5_LT_20211025.dam	Instrument Name	QTRAP 6500+ Low Mass
Project	N/A	Analytes QTY	204:144

Compound name: Phloretin (273.1 / 167.0)

Sample Name	Sample Type	Area (cps)	Is Area (cps)	RT (min)	S/N	Target Conc	Calculated Conc.()
STD_0.5nM	Standard	1.44e4	N/A	4.77	34.9	0.5000	3.454327e-1
STD_1nM	Standard	3.41e4	N/A	4.76	71.2	1.0000	9.723479e-1
STD_5nM	Standard	2.16e5	N/A	4.76	287.4	5.0000	6.762120e0
STD_10nM	Standard	3.15e5	N/A	4.76	271.4	10.0000	9.934399e0
STD_20nM	Standard	6.16e5	N/A	4.76	362.9	20.0000	1.951587e1
STD_50nM	Standard	1.61e6	N/A	4.76	538.2	50.0000	5.112822e1
STD_100nM	Standard	3.16e6	N/A	4.77	545.9	100.0000	1.006713e2
STD_200nM	Standard	6.19e6	N/A	4.76	448.7	200.0000	1.971703e2
STD_500nM	Standard	N/A	N/A	N/A	N/A	500.0000	N/A
STD_1000nM	Standard	N/A	N/A	N/A	N/A	1000.0000	N/A
STD_2000nM	Standard	N/A	N/A	N/A	N/A	2000.0000	N/A
V1.0_MW_RQC1_20211018	Quality Control	N/A	N/A	N/A	N/A	0.0000	N/A
Blank	Unknown	N/A	N/A	N/A	N/A	N/A	N/A
V1.0_MWMS_20211021_1	Unknown	5.09e6	N/A	4.78	608.3	N/A	1.620100e2
MWXS212101D3_R1	Quality Control	5.12e6	N/A	4.77	543.4	0.0000	1.630662e2
MWXS212101D3_R2	Quality Control	5.05e6	N/A	4.77	383.5	0.0000	1.608577e2
MWXS212101D3_R3	Quality Control	5.07e6	N/A	4.77	496.9	0.0000	1.615869e2
A21233250b_b	Unknown	N/A	N/A	N/A	N/A	N/A	N/A
A21233251b_b	Unknown	N/A	N/A	N/A	N/A	N/A	N/A
A21233252b_b	Unknown	N/A	N/A	N/A	N/A	N/A	N/A
A21233253b_b	Unknown	5.94e6	N/A	4.78	405.7	N/A	1.892080e2
A21233254b_b	Unknown	9.09e6	N/A	4.77	385.1	N/A	2.894115e2
A21233255b_b	Unknown	9.64e6	N/A	4.77	456.7	N/A	3.071694e2
A21233256b_b	Unknown	2.30e6	N/A	4.78	369.1	N/A	7.320717e1
A21233257b_b	Unknown	2.09e6	N/A	4.78	342.7	N/A	6.635316e1
A21233258b_b	Unknown	8.38e5	N/A	4.78	260.2	N/A	2.658620e1
A21233259b_b	Unknown	7.69e4	N/A	4.78	135.1	N/A	2.336126e0
A21233260b_b	Unknown	N/A	N/A	N/A	N/A	N/A	N/A
A21233261b_b	Unknown	3.80e3	N/A	4.77	12.1	N/A	6.499808e-3
A21233262b_b	Unknown	1.89e6	N/A	4.78	365.1	N/A	6.005550e1
A21233263b_b	Unknown	4.77e6	N/A	4.77	393.3	N/A	1.518853e2
A21233264b_b	Unknown	7.94e6	N/A	4.78	381.9	N/A	2.529327e2
A21233265b_b	Unknown	2.19e6	N/A	4.77	338.9	N/A	6.960408e1
A21233266b_b	Unknown	3.50e6	N/A	4.77	349.2	N/A	1.113551e2
A21233267b_b	Unknown	1.02e6	N/A	4.78	214.8	N/A	3.238315e1

Compound name: Phloretin
Regression Equation: y = 3.13837e4 x + 3599.36185 (r = 0.99898) (weighting: 1 / x)

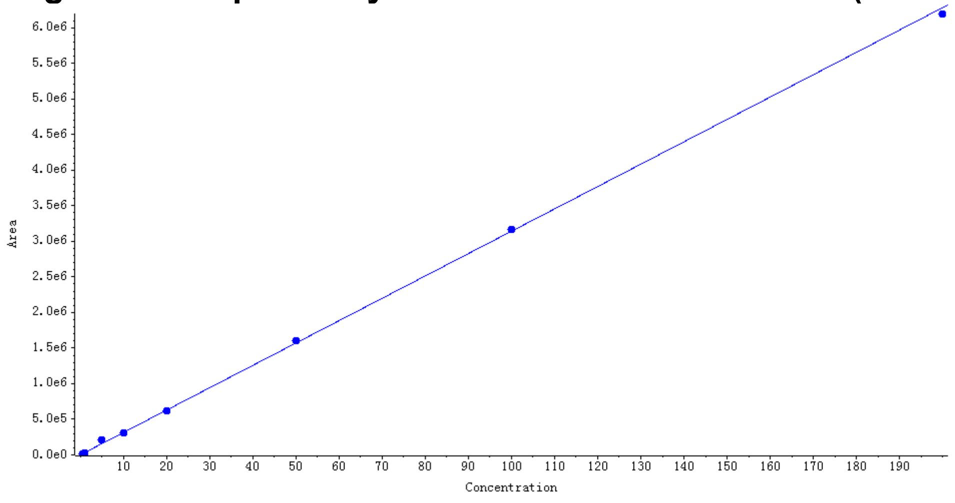

Peak Review

Blank
Phloretin AREA:N/A S/N:N/A

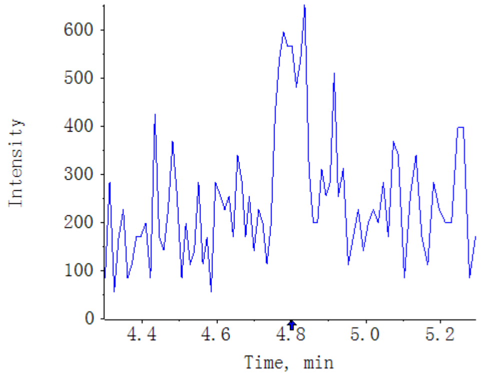

V1.0_MWMS_20211021_1
Phloretin AREA:5.09e6 S/N:608.3

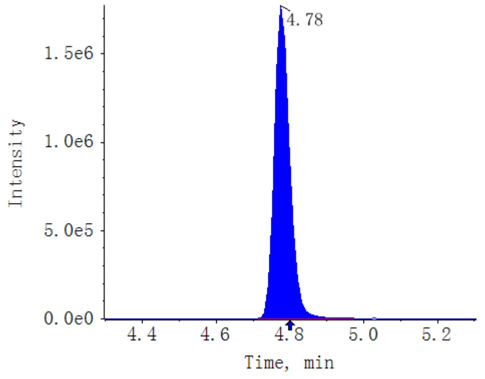

A21233250b_b
Phloretin AREA:N/A S/N:N/A

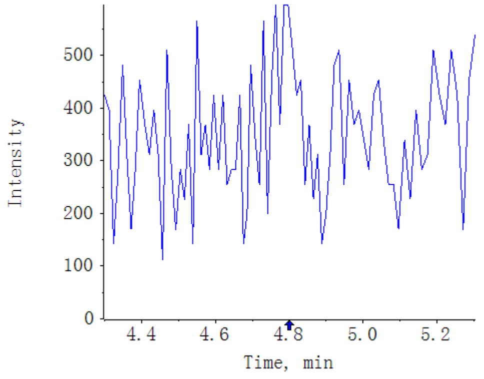

A21233251b_b
Phloretin AREA:N/A S/N:N/A

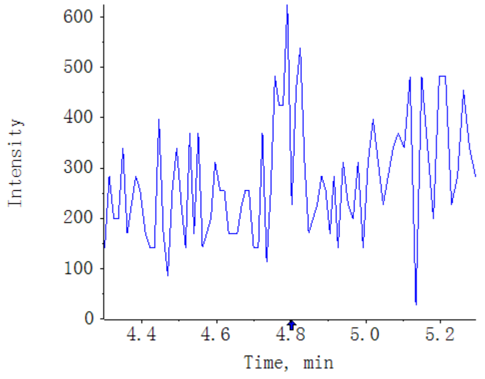

A21233252b_b
Phloretin AREA:N/A S/N:N/A

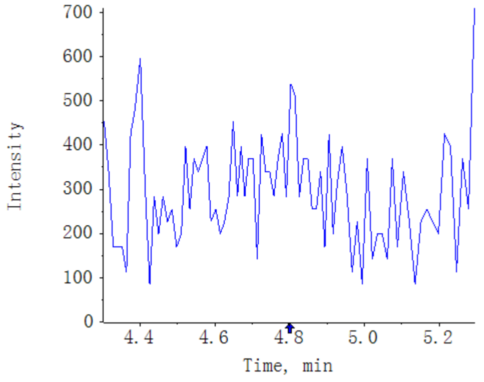

A21233253b_b
Phloretin AREA:5.94e6 S/N:405.7

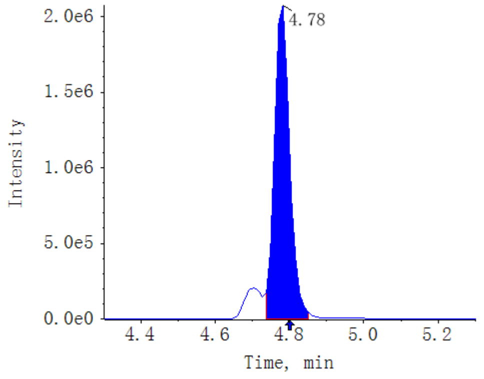

A21233254b_b
Phloretin AREA:9.09e6 S/N:385.1

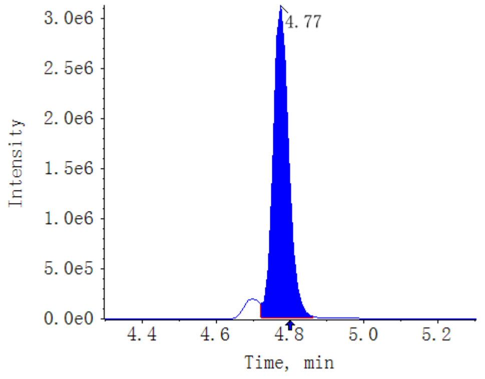

A21233255b_b
Phloretin AREA:9.64e6 S/N:456.7

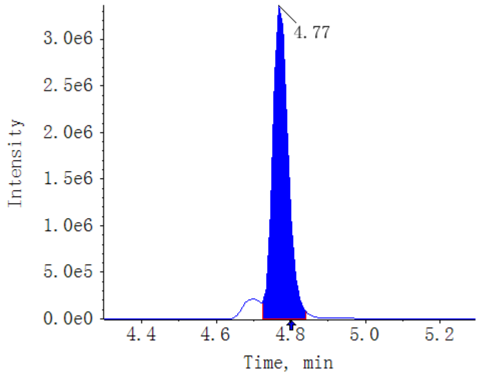

A21233256b_b
Phloretin AREA:2.30e6 S/N:369.1

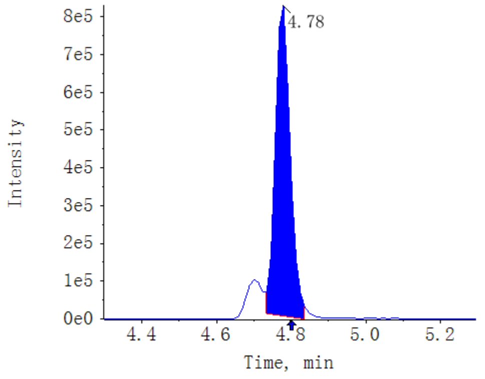

A21233257b_b
Phloretin AREA:2.09e6 S/N:342.7

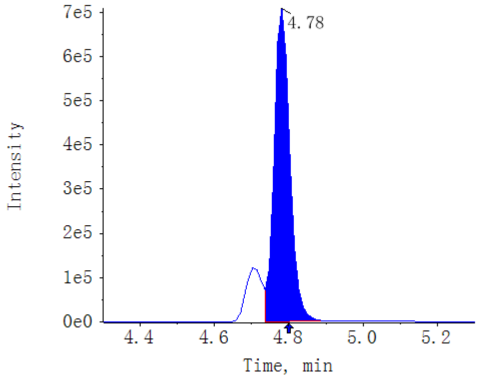

A21233258b_b
Phloretin AREA:8.38e5 S/N:260.2

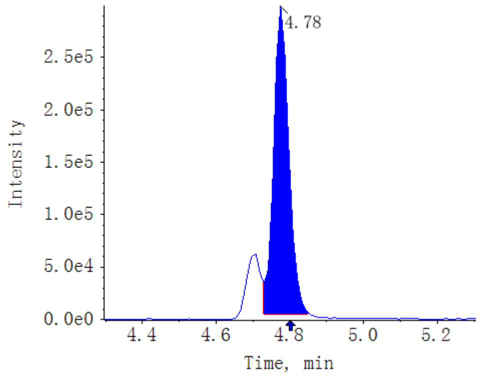

A21233259b_b
Phloretin AREA:7.69e4 S/N:135.1

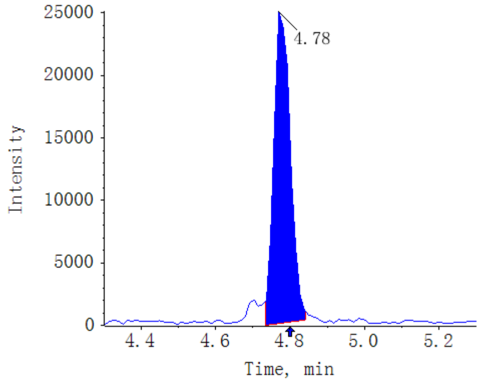

A21233260b_b
Phloretin AREA:N/A S/N:N/A

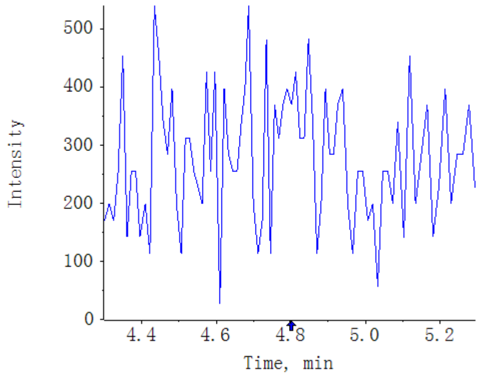

A21233261b_b
Phloretin AREA:3.80e3 S/N:12.1

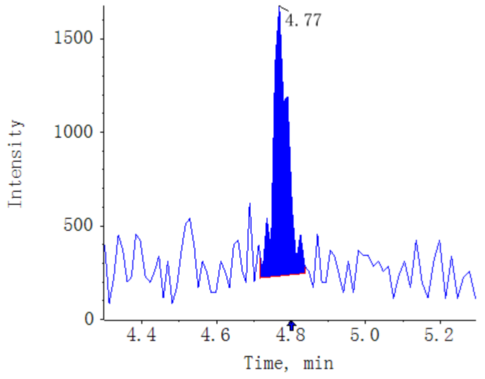

A21233262b_b
Phloretin AREA:1.89e6 S/N:365.1

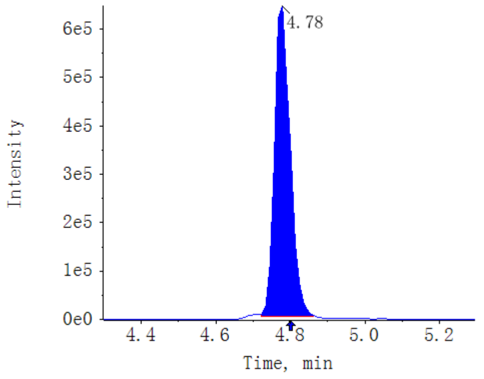

A21233263b_b
Phloretin AREA:4.77e6 S/N:393.3

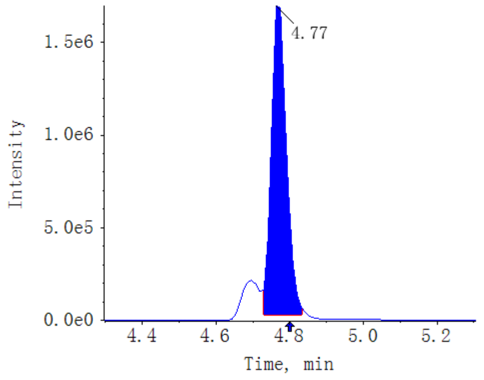

A21233264b_b
Phloretin AREA:7.94e6 S/N:381.9

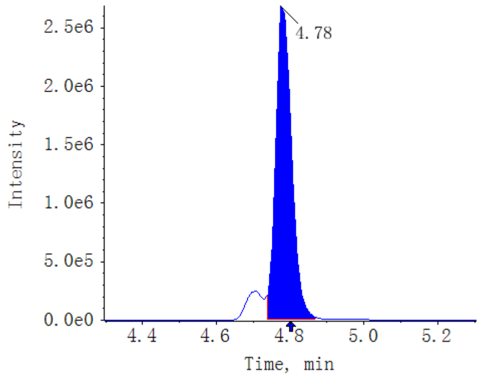

A21233265b_b
Phloretin AREA:2.19e6 S/N:338.9

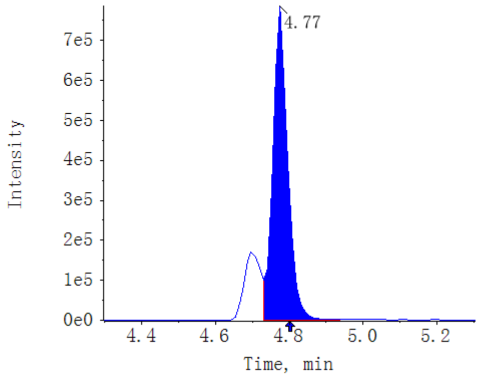

A21233266b_b
Phloretin AREA:3.50e6 S/N:349.2

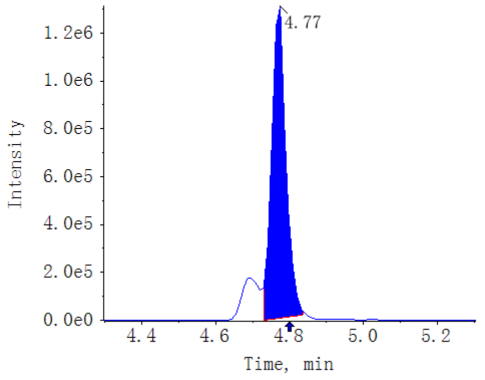

A21233267b_b
Phloretin AREA:1.02e6 S/N:214.8

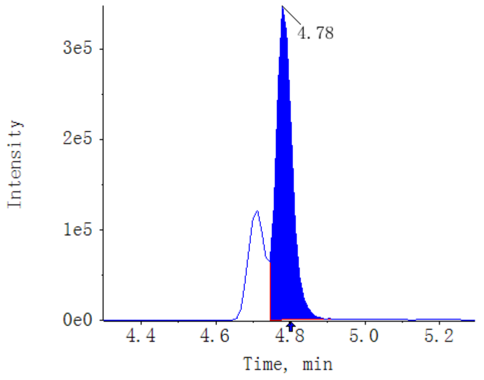

**Result Table**	MWXS-21-2101D-3_18_WH6500-5_A20-3_V1.0_TY_20211028	**Algorithm Used**	MQ4
**Acquisition Method**	Flavonoids_V1.0_WH6500-5_LT_20211025.dam	**Instrument Name**	QTRAP 6500+ Low Mass
**Project**	N/A	**Analytes QTY**	204:150

Compound name: 2'-Hydroxygenistein (285.0 / 217.1)

Sample Name	Sample Type	Area (cps)	Is Area (cps)	RT (min)	S/N	Target Conc	Calculated Conc.()
STD_0.5nM	Standard	N/A	N/A	N/A	N/A	0.5000	N/A
STD_1nM	Standard	1.54e4	N/A	3.93	14.5	1.0000	6.743100e-1
STD_5nM	Standard	1.09e5	N/A	3.94	40.4	5.0000	6.097807e0
STD_10nM	Standard	1.84e5	N/A	3.94	46.3	10.0000	1.037800e1
STD_20nM	Standard	3.55e5	N/A	3.93	73.0	20.0000	2.027102e1
STD_50nM	Standard	9.29e5	N/A	3.94	108.3	50.0000	5.330769e1
STD_100nM	Standard	1.78e6	N/A	3.95	153.2	100.0000	1.024537e2
STD_200nM	Standard	3.35e6	N/A	3.94	188.8	200.0000	1.928175e2
STD_500nM	Standard	N/A	N/A	N/A	N/A	500.0000	N/A
STD_1000nM	Standard	N/A	N/A	N/A	N/A	1000.0000	N/A
STD_2000nM	Standard	N/A	N/A	N/A	N/A	2000.0000	N/A
V1.0_MW_RQC1_20211018	Quality Control	N/A	N/A	N/A	N/A	0.0000	N/A
Blank	Unknown	N/A	N/A	N/A	N/A	N/A	N/A
V1.0_MWMS_20211021_1	Unknown	2.72e6	N/A	3.95	171.8	N/A	1.564257e2
MWXS212101D3_R1	Quality Control	2.68e6	N/A	3.95	190.0	0.0000	1.545370e2
MWXS212101D3_R2	Quality Control	2.74e6	N/A	3.95	165.1	0.0000	1.575790e2
MWXS212101D3_R3	Quality Control	2.76e6	N/A	3.95	181.7	0.0000	1.590474e2
A21233250b_b	Unknown	N/A	N/A	N/A	N/A	N/A	N/A
A21233251b_b	Unknown	N/A	N/A	N/A	N/A	N/A	N/A
A21233252b_b	Unknown	N/A	N/A	N/A	N/A	N/A	N/A
A21233253b_b	Unknown	1.55e4	N/A	3.99	17.3	N/A	6.777220e-1
A21233254b_b	Unknown	1.38e4	N/A	3.99	19.8	N/A	5.838434e-1
A21233255b_b	Unknown	2.37e4	N/A	3.99	21.4	N/A	1.151848e0
A21233256b_b	Unknown	1.63e4	N/A	4.00	14.3	N/A	7.260531e-1
A21233257b_b	Unknown	1.57e4	N/A	4.00	15.7	N/A	6.894020e-1
A21233258b_b	Unknown	N/A	N/A	N/A	N/A	N/A	N/A
A21233259b_b	Unknown	N/A	N/A	N/A	N/A	N/A	N/A
A21233260b_b	Unknown	N/A	N/A	N/A	N/A	N/A	N/A
A21233261b_b	Unknown	N/A	N/A	N/A	N/A	N/A	N/A
A21233262b_b	Unknown	N/A	N/A	N/A	N/A	N/A	N/A
A21233263b_b	Unknown	2.46e4	N/A	3.99	16.1	N/A	1.202948e0
A21233264b_b	Unknown	2.02e4	N/A	4.00	19.7	N/A	9.535194e-1
A21233265b_b	Unknown	1.54e4	N/A	3.99	18.4	N/A	6.724102e-1
A21233266b_b	Unknown	1.20e4	N/A	3.99	16.0	N/A	4.759983e-1
A21233267b_b	Unknown	2.12e4	N/A	4.00	20.6	N/A	1.006407e0

Compound name: 2'-Hydroxygenistein
Regression Equation: y = 17349.32894 x + 3704.57035 (r = 0.99872) (weighting: 1 / x)

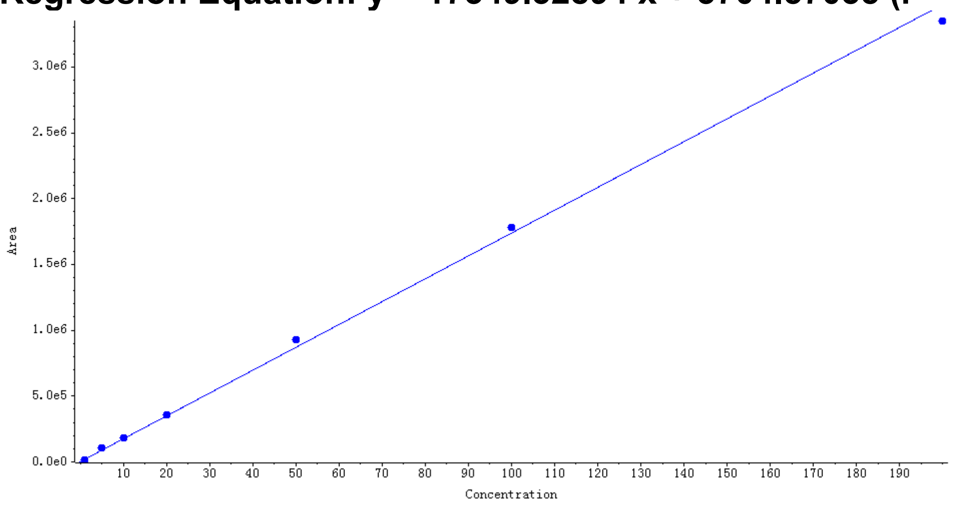

Peak Review

Blank
2'-Hydroxygenistein AREA:N/A
S/N:N/A

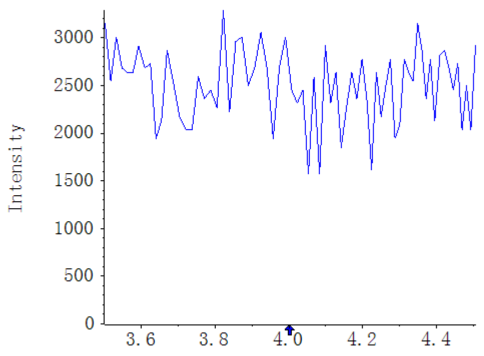

V1.0_MWMS_20211021_1
2'-Hydroxygenistein AREA:2.72e6
S/N:171.8

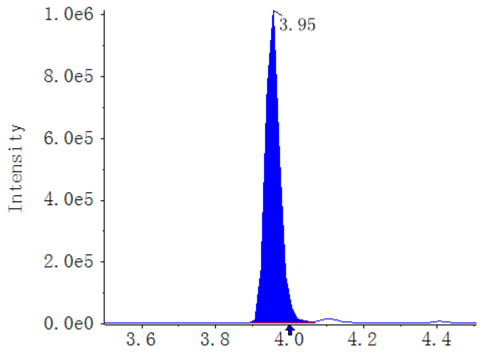

A21233250b_b
2'-Hydroxygenistein AREA:N/A
S/N:N/A

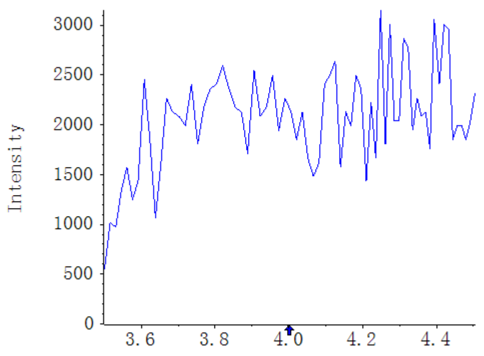

A21233251b_b
2'-Hydroxygenistein AREA:N/A
S/N:N/A

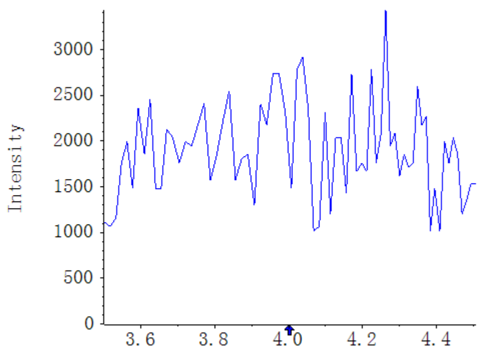

A21233252b_b
2'-Hydroxygenistein AREA:N/A
S/N:N/A

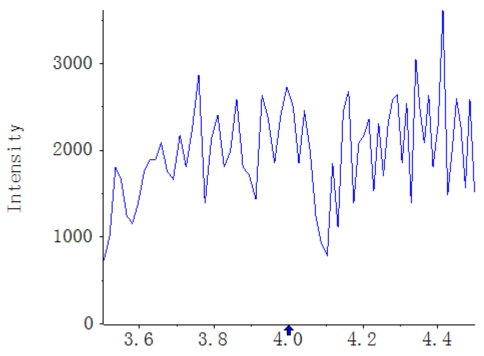

A21233253b_b
2'-Hydroxygenistein AREA:1.55e4
S/N:17.3

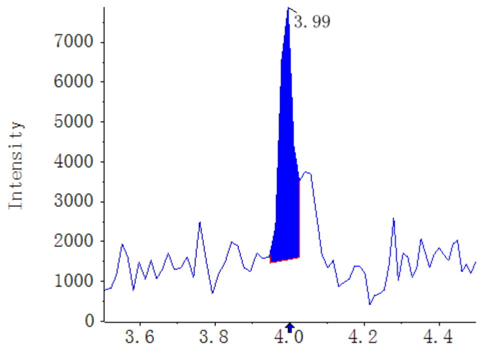

A21233254b_b
2'-Hydroxygenistein AREA:1.38e4
S/N:19.8

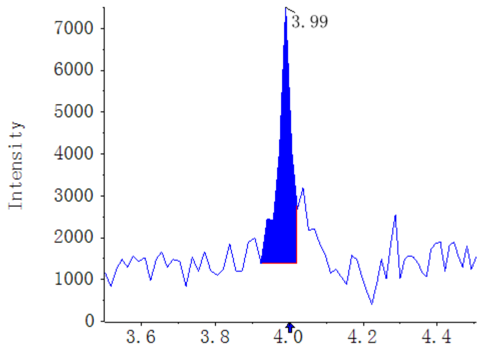

A21233255b_b
2'-Hydroxygenistein AREA:2.37e4
S/N:21.4

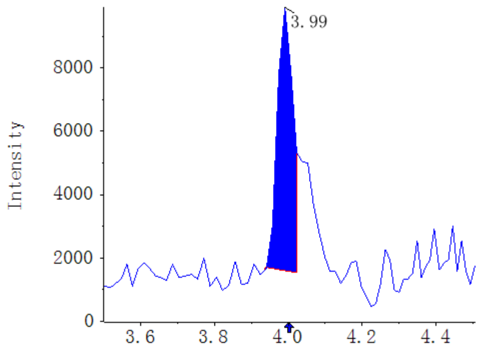

A21233256b_b
2'-Hydroxygenistein AREA:1.63e4
S/N:14.3

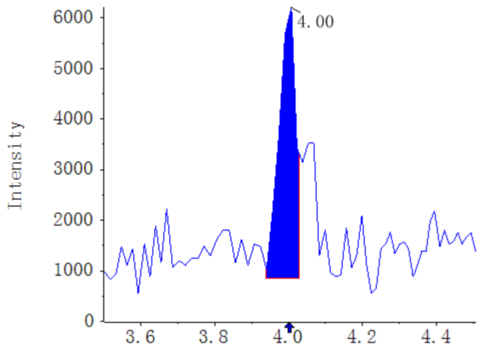

A21233257b_b
2'-Hydroxygenistein AREA:1.57e4
S/N:15.7

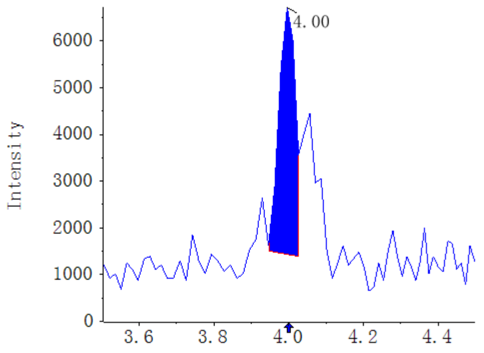

A21233258b_b
2'-Hydroxygenistein AREA:N/A
S/N:N/A

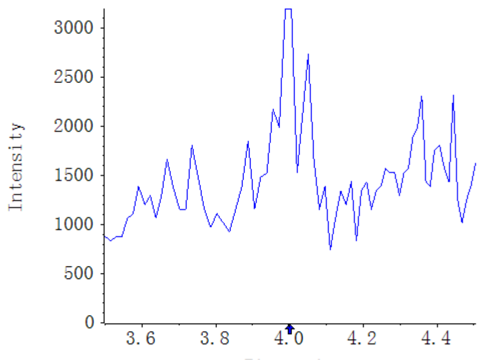

A21233259b_b
2'-Hydroxygenistein AREA:N/A
S/N:N/A

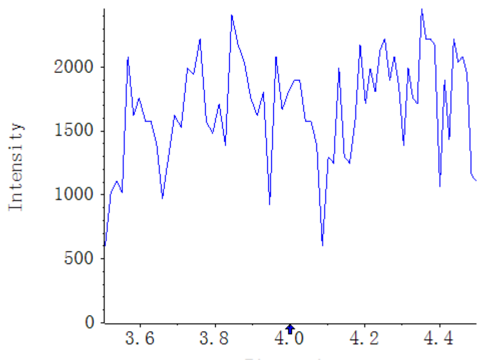

A21233260b_b
2'-Hydroxygenistein AREA:N/A
S/N:N/A

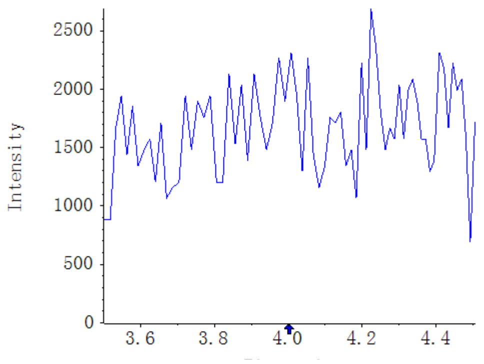

A21233261b_b
2'-Hydroxygenistein AREA:N/A
S/N:N/A

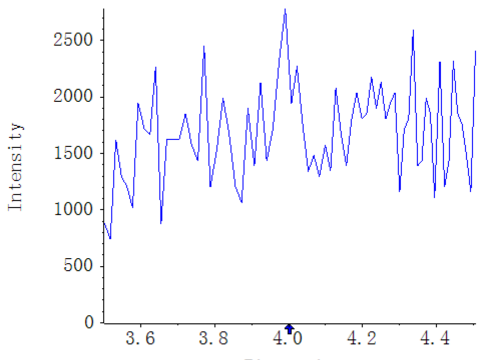

A21233262b_b
2'-Hydroxygenistein AREA:N/A
S/N:N/A

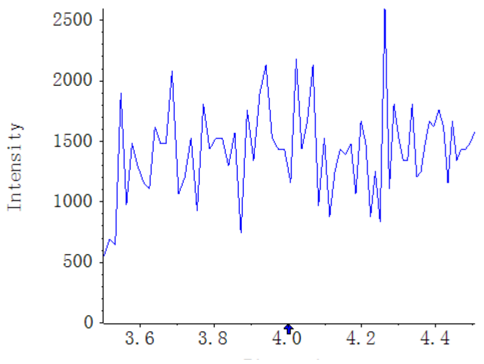

A21233263b_b
2'-Hydroxygenistein AREA:2.46e4
S/N:16.1

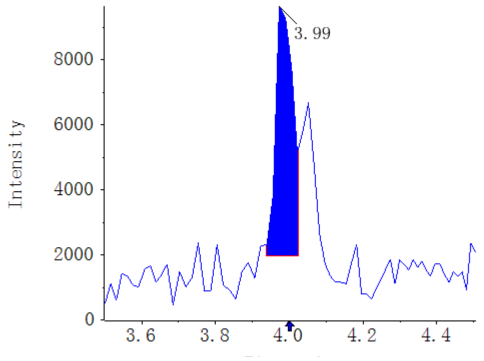

A21233264b_b
2'-Hydroxygenistein AREA:2.02e4
S/N:19.7

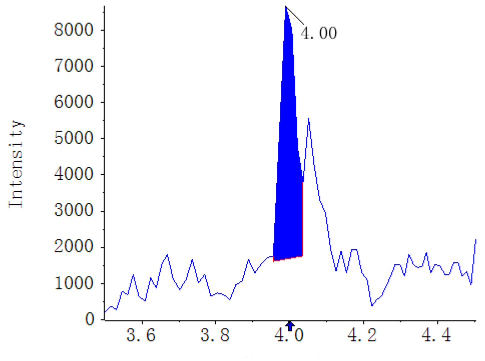

A21233265b_b
2'-Hydroxygenistein AREA:1.54e4
S/N:18.4

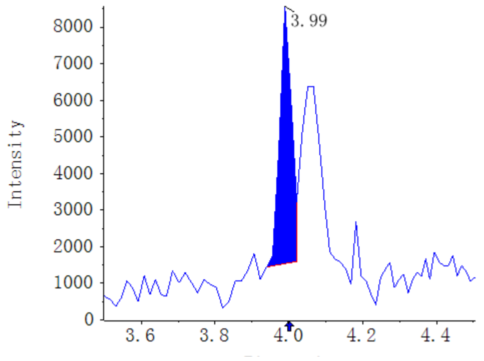

A21233266b_b
2'-Hydroxygenistein AREA:1.20e4
S/N:16.0

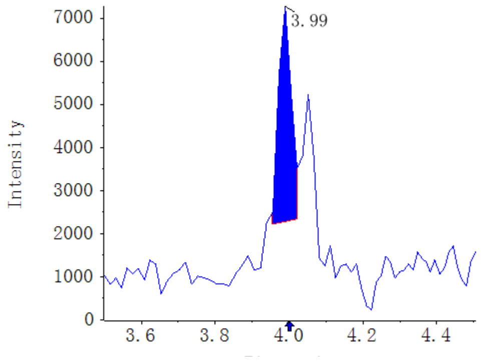

A21233267b_b
2'-Hydroxygenistein AREA:2.12e4
S/N:20.6

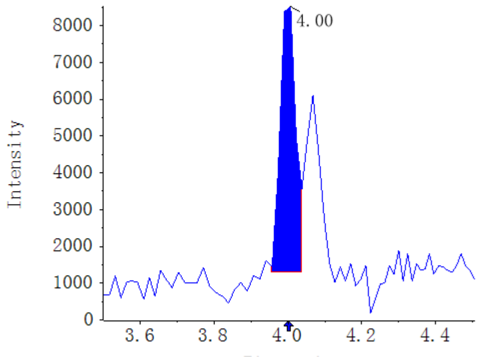

**Result Table**	MWXS-21-2101D-3_18_WH6500-5_A20-3_V1.0_TY_20211028	**Algorithm Used**	MQ4
**Acquisition Method**	Flavonoids_V1.0_WH6500-5_LT_20211025.dam	**Instrument Name**	QTRAP 6500+ Low Mass
**Project**	N/A	**Analytes QTY**	204:151

Compound name: Afzelechin (273.1 / 97.0)

Sample Name	Sample Type	Area (cps)	Is Area (cps)	RT (min)	S/N	Target Conc	Calculated Conc.()
STD_0.5nM	Standard	2.41e3	N/A	2.51	7.0	0.5000	5.873713e-1
STD_1nM	Standard	2.93e3	N/A	2.50	8.1	1.0000	8.034364e-1
STD_5nM	Standard	1.51e4	N/A	2.50	24.3	5.0000	5.929118e0
STD_10nM	Standard	2.18e4	N/A	2.50	35.7	10.0000	8.726105e0
STD_20nM	Standard	4.39e4	N/A	2.50	58.0	20.0000	1.803070e1
STD_50nM	Standard	1.29e5	N/A	2.50	112.7	50.0000	5.365338e1
STD_100nM	Standard	2.36e5	N/A	2.51	147.3	100.0000	9.898690e1
STD_200nM	Standard	4.76e5	N/A	2.50	189.8	200.0000	1.997830e2
STD_500nM	Standard	N/A	N/A	N/A	N/A	500.0000	N/A
STD_1000nM	Standard	N/A	N/A	N/A	N/A	1000.0000	N/A
STD_2000nM	Standard	N/A	N/A	N/A	N/A	2000.0000	N/A
V1.0_MW_RQC1_20211018	Quality Control	N/A	N/A	N/A	N/A	0.0000	N/A
Blank	Unknown	N/A	N/A	N/A	N/A	N/A	N/A
V1.0_MWMS_20211021_1	Unknown	3.74e5	N/A	2.50	164.5	N/A	1.566454e2
MWXS212101D3_R1	Quality Control	3.86e5	N/A	2.50	214.3	0.0000	1.616872e2
MWXS212101D3_R2	Quality Control	3.67e5	N/A	2.50	212.1	0.0000	1.538263e2
MWXS212101D3_R3	Quality Control	3.76e5	N/A	2.50	171.5	0.0000	1.575193e2
A21233250b_b	Unknown	N/A	N/A	N/A	N/A	N/A	N/A
A21233251b_b	Unknown	2.99e4	N/A	2.54	16.0	N/A	1.212205e1
A21233252b_b	Unknown	1.45e4	N/A	2.55	7.2	N/A	5.657142e0
A21233253b_b	Unknown	N/A	N/A	N/A	N/A	N/A	N/A
A21233254b_b	Unknown	N/A	N/A	N/A	N/A	N/A	N/A
A21233255b_b	Unknown	1.42e4	N/A	2.53	8.7	N/A	5.540977e0
A21233256b_b	Unknown	8.74e3	N/A	2.52	5.7	N/A	3.248910e0
A21233257b_b	Unknown	N/A	N/A	N/A	N/A	N/A	N/A
A21233258b_b	Unknown	1.30e4	N/A	2.52	9.0	N/A	5.048716e0
A21233259b_b	Unknown	N/A	N/A	N/A	N/A	N/A	N/A
A21233260b_b	Unknown	2.20e4	N/A	2.54	7.7	N/A	8.812834e0
A21233261b_b	Unknown	N/A	N/A	N/A	N/A	N/A	N/A
A21233262b_b	Unknown	N/A	N/A	N/A	N/A	N/A	N/A
A21233263b_b	Unknown	N/A	N/A	N/A	N/A	N/A	N/A
A21233264b_b	Unknown	N/A	N/A	N/A	N/A	N/A	N/A
A21233265b_b	Unknown	N/A	N/A	N/A	N/A	N/A	N/A
A21233266b_b	Unknown	1.04e4	N/A	2.52	5.2	N/A	3.953060e0
A21233267b_b	Unknown	N/A	N/A	N/A	N/A	N/A	N/A

Compound name: Afzelechin
Regression Equation: y = 2378.79838 x + 1016.17421 (r = 0.99883) (weighting: 1 / x)

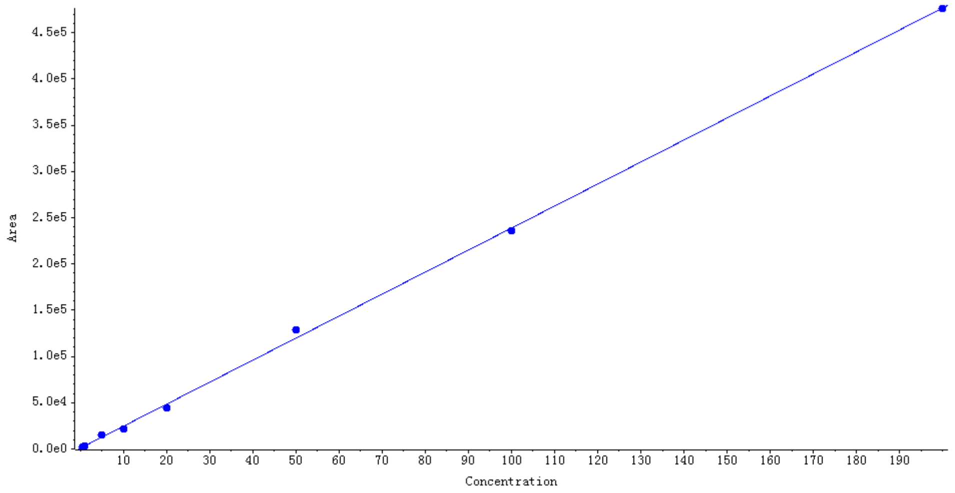

Peak Review

Blank
Afzelechin AREA:N/A S/N:N/A

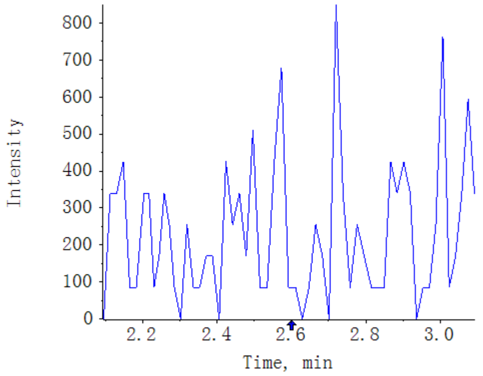

V1.0_MWMS_20211021_1
Afzelechin AREA:3.74e5 S/N:164.5

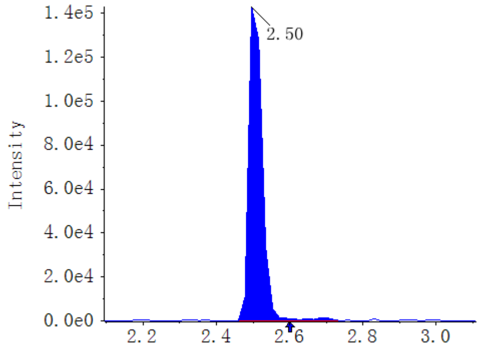

A21233250b_b
Afzelechin AREA:N/A S/N:N/A

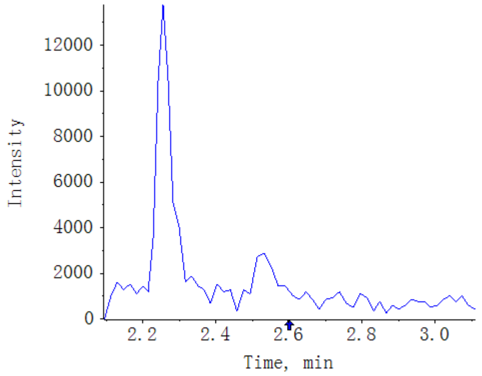

A21233251b_b
Afzelechin AREA:2.99e4 S/N:16.0

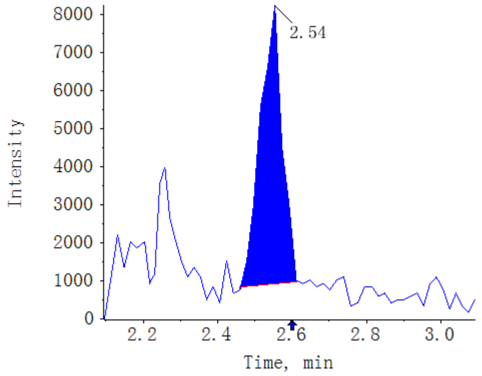

A21233252b_b
Afzelechin AREA:1.45e4 S/N:7.2

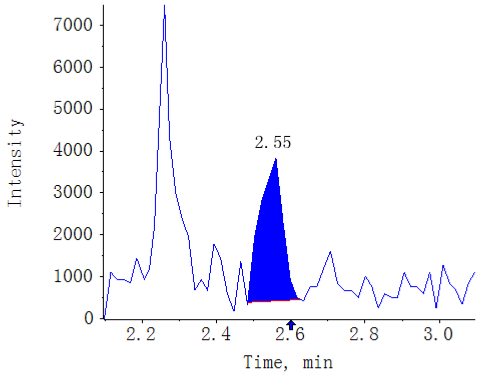

A21233253b_b
Afzelechin AREA:N/A S/N:N/A

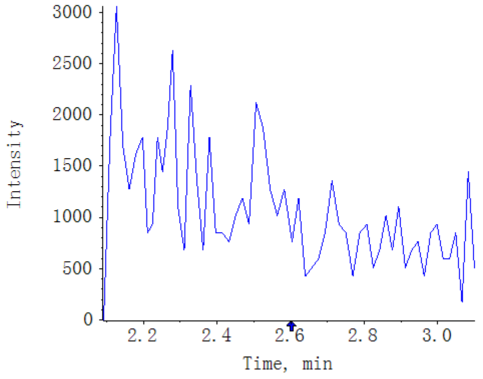

A21233254b_b
Afzelechin AREA:N/A S/N:N/A

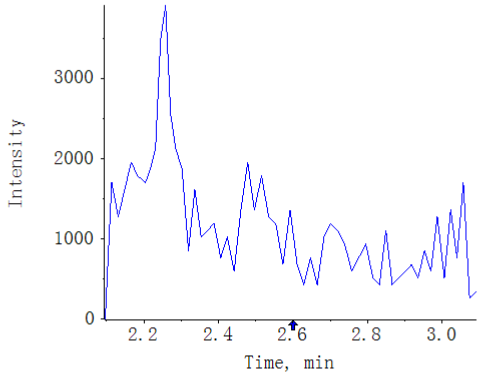

A21233255b_b
Afzelechin AREA:1.42e4 S/N:8.7

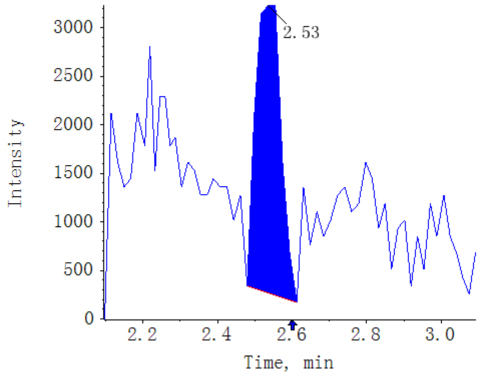

A21233256b_b
Afzelechin AREA:8.74e3 S/N:5.7

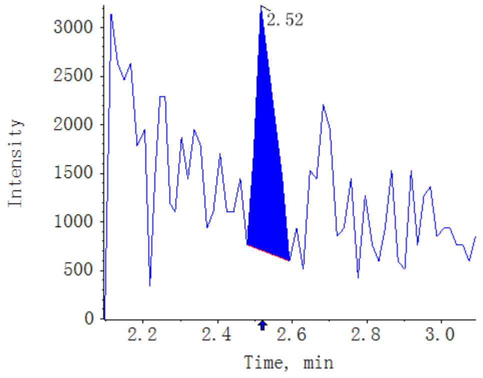

A21233257b_b
Afzelechin AREA:N/A S/N:N/A

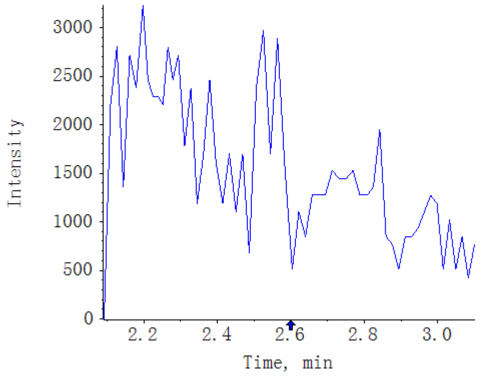

A21233258b_b
Afzelechin AREA:1.30e4 S/N:9.0

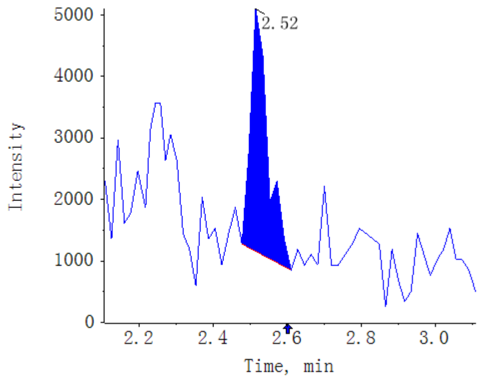

A21233259b_b
Afzelechin AREA:N/A S/N:N/A

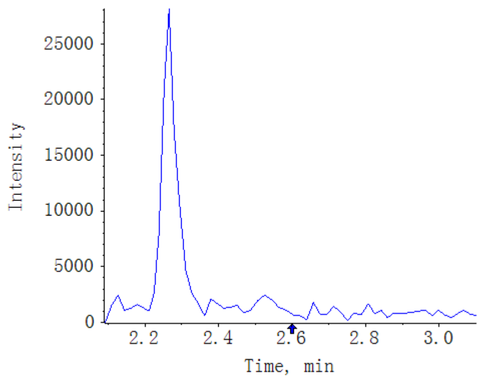

A21233260b_b
Afzelechin AREA:2.20e4 S/N:7.7

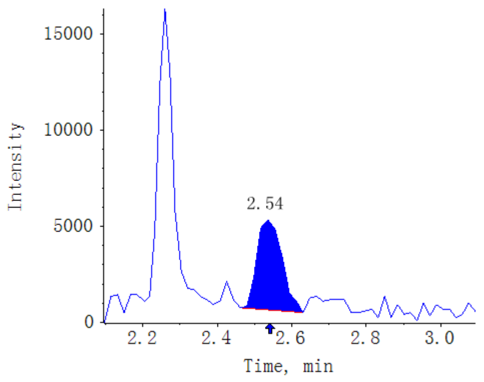

A21233261b_b
Afzelechin AREA:N/A S/N:N/A

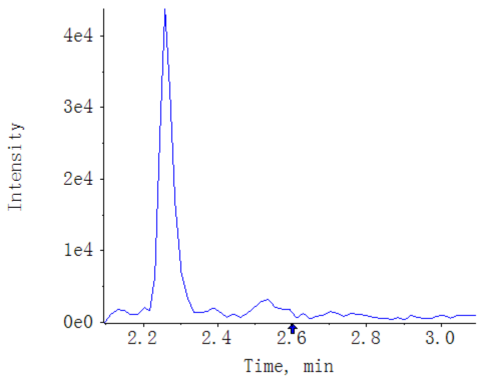

A21233262b_b
Afzelechin AREA:N/A S/N:N/A

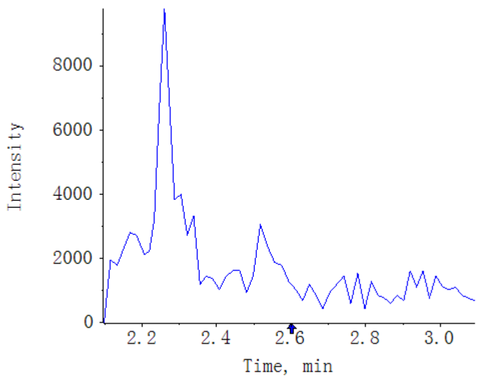

A21233263b_b
Afzelechin AREA:N/A S/N:N/A

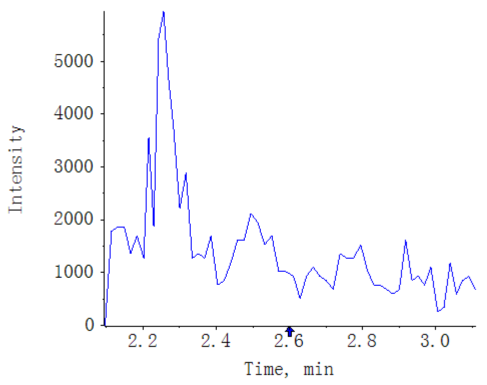

A21233264b_b
Afzelechin AREA:N/A S/N:N/A

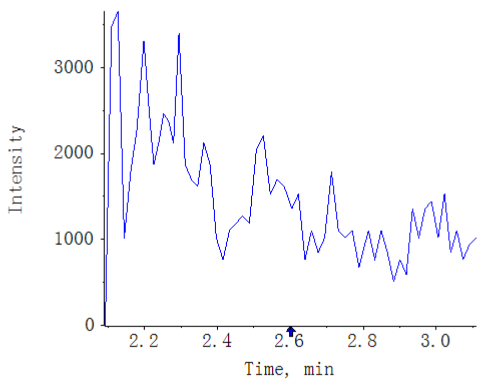

A21233265b_b
Afzelechin AREA:N/A S/N:N/A

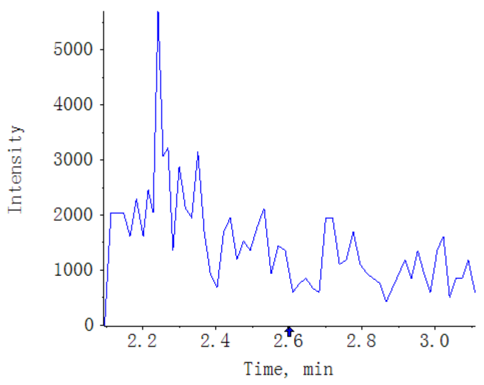

A21233266b_b
Afzelechin AREA:1.04e4 S/N:5.2

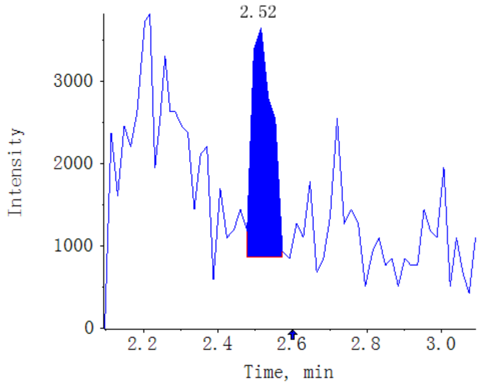

A21233267b_b
Afzelechin AREA:N/A S/N:N/A

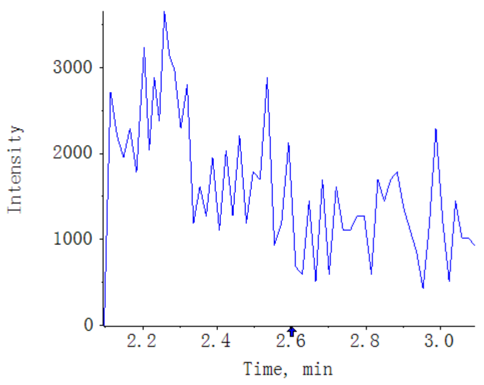

Result Table	MWXS-21-2101D-3_18_WH6500-5_A20-3_V1.0_TY_20211028	Algorithm Used	MQ4
Acquisition Method	Flavonoids_V1.0_WH6500-5_LT_20211025.dam	Instrument Name	QTRAP 6500+ Low Mass
Project	N/A	Analytes QTY	204:158

Compound name: Naringenin chalcone (271.1 / 151.0)

Sample Name	Sample Type	Area (cps)	Is Area (cps)	RT (min)	S/N	Target Conc	Calculated Conc.()
STD_0.5nM	Standard	5.31e3	N/A	4.84	14.0	0.5000	3.231434e-1
STD_1nM	Standard	1.21e4	N/A	4.83	28.5	1.0000	9.285855e-1
STD_5nM	Standard	7.92e4	N/A	4.83	63.0	5.0000	6.905083e0
STD_10nM	Standard	1.17e5	N/A	4.83	69.2	10.0000	1.025034e1
STD_20nM	Standard	2.28e5	N/A	4.82	76.8	20.0000	2.011255e1
STD_50nM	Standard	5.76e5	N/A	4.83	80.2	50.0000	5.113991e1
STD_100nM	Standard	1.14e6	N/A	4.84	80.7	100.0000	1.012898e2
STD_200nM	Standard	2.20e6	N/A	4.83	85.1	200.0000	1.955505e2
STD_500nM	Standard	N/A	N/A	N/A	N/A	500.0000	N/A
STD_1000nM	Standard	N/A	N/A	N/A	N/A	1000.0000	N/A
STD_2000nM	Standard	N/A	N/A	N/A	N/A	2000.0000	N/A
V1.0_MW_RQC1_20211018	Quality Control	1.79e5	N/A	4.82	93.6	0.0000	1.578834e1
Blank	Unknown	N/A	N/A	N/A	N/A	N/A	N/A
V1.0_MWMS_20211021_1	Unknown	1.43e6	N/A	4.84	69.4	N/A	1.275502e2
MWXS212101D3_R1	Quality Control	1.52e6	N/A	4.84	76.2	0.0000	1.355855e2
MWXS212101D3_R2	Quality Control	1.46e6	N/A	4.84	60.8	0.0000	1.299998e2
MWXS212101D3_R3	Quality Control	1.45e6	N/A	4.84	62.0	0.0000	1.289043e2
A21233250b_b	Unknown	1.97e4	N/A	4.83	18.7	N/A	1.606717e0
A21233251b_b	Unknown	1.76e4	N/A	4.84	17.2	N/A	1.417131e0
A21233252b_b	Unknown	7.06e3	N/A	4.84	9.8	N/A	4.790587e-1
A21233253b_b	Unknown	4.13e7	N/A	4.84	19.4	N/A	3.679800e3
A21233254b_b	Unknown	4.17e7	N/A	4.83	18.0	N/A	3.709934e3
A21233255b_b	Unknown	5.78e7	N/A	4.83	23.7	N/A	5.145515e3
A21233256b_b	Unknown	1.78e7	N/A	4.84	13.8	N/A	1.581106e3
A21233257b_b	Unknown	2.57e7	N/A	4.84	16.4	N/A	2.289083e3
A21233258b_b	Unknown	9.89e6	N/A	4.84	14.0	N/A	8.809063e2
A21233259b_b	Unknown	4.95e5	N/A	4.84	16.9	N/A	4.395808e1
A21233260b_b	Unknown	1.58e4	N/A	4.83	13.4	N/A	1.253330e0
A21233261b_b	Unknown	1.25e5	N/A	4.84	21.0	N/A	1.096486e1
A21233262b_b	Unknown	7.93e6	N/A	4.84	38.9	N/A	7.061854e2
A21233263b_b	Unknown	6.16e7	N/A	4.83	23.8	N/A	5.482672e3
A21233264b_b	Unknown	7.72e7	N/A	4.84	23.2	N/A	6.872233e3
A21233265b_b	Unknown	3.80e7	N/A	4.83	16.8	N/A	3.387472e3
A21233266b_b	Unknown	4.29e7	N/A	4.83	20.8	N/A	3.820293e3
A21233267b_b	Unknown	2.44e7	N/A	4.84	15.9	N/A	2.171246e3

Compound name: Naringenin chalcone
Regression Equation: y = 11228.95985 x + 1683.81489 (r = 0.99872) (weighting: 1 / x)

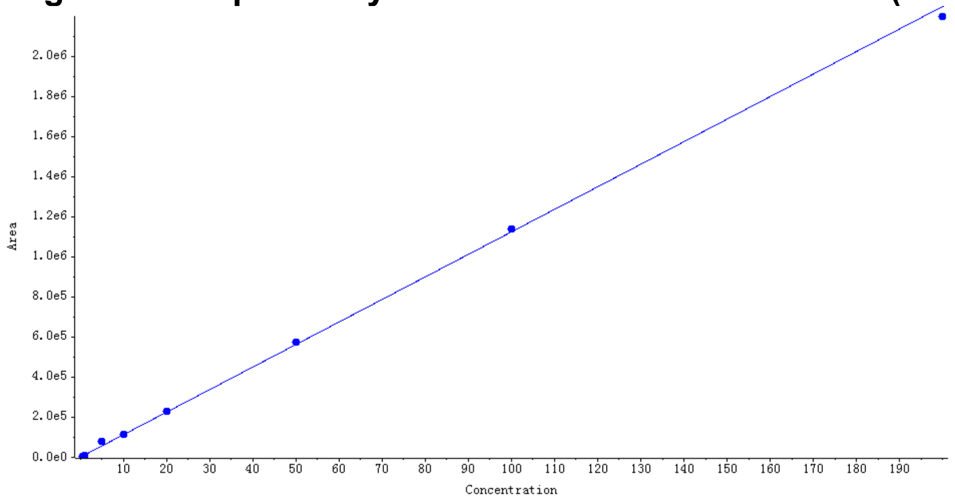

Peak Review

Blank
Naringenin chalcone AREA:N/A
S/N:N/A

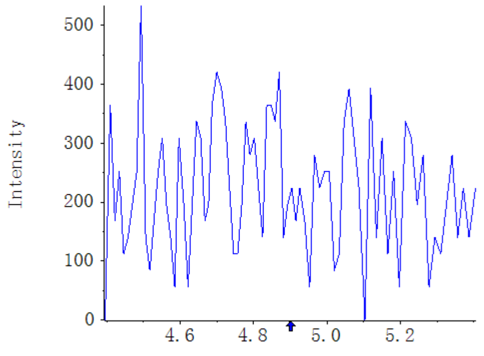

V1.0_MWMS_20211021_1
Naringenin chalcone AREA:1.43e6
S/N:69.4

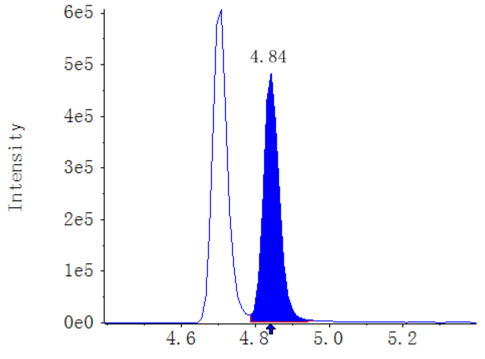

A21233250b_b
Naringenin chalcone AREA:1.97e4
S/N:18.7

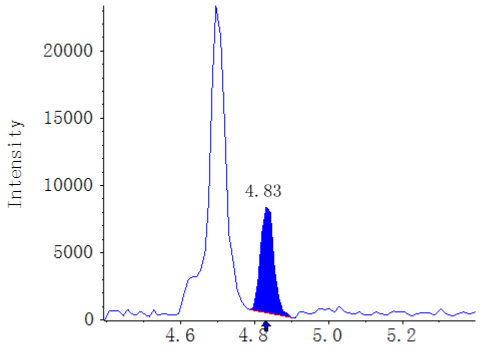

A21233251b_b
Naringenin chalcone AREA:1.76e4
S/N:17.2

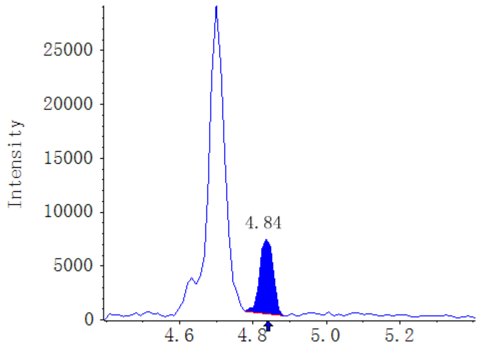

A21233252b_b
Naringenin chalcone AREA:7.06e3
S/N:9.8

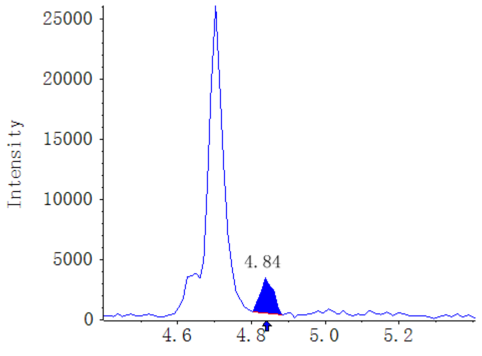

A21233253b_b
Naringenin chalcone AREA:4.13e7
S/N:19.4

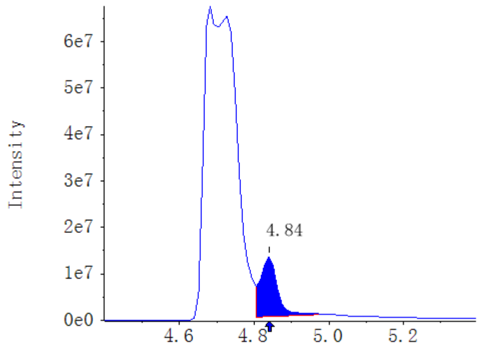

A21233254b_b
Naringenin chalcone AREA:4.17e7
S/N:18.0

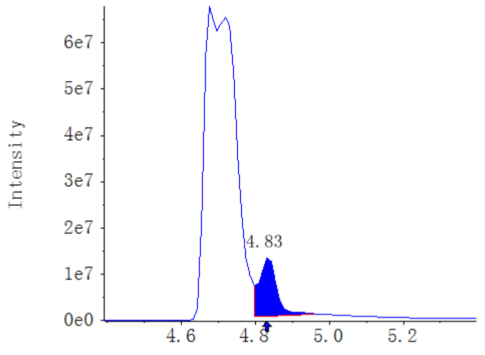

A21233255b_b
Naringenin chalcone AREA:5.78e7
S/N:23.7

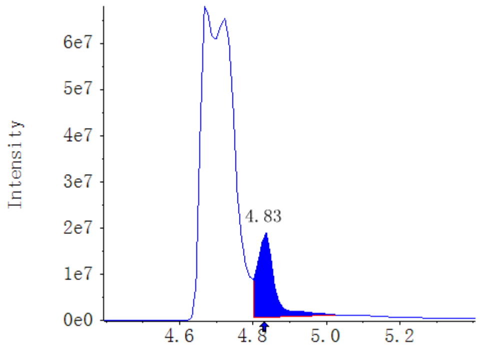

A21233256b_b
Naringenin chalcone AREA:1.78e7
S/N:13.8

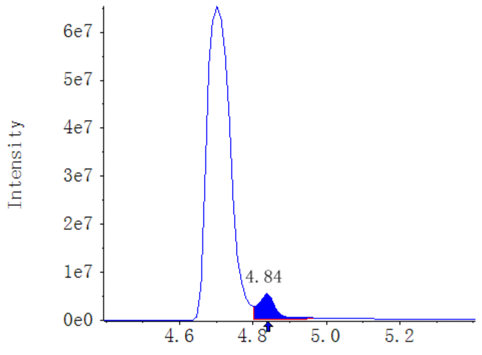

A21233257b_b
Naringenin chalcone AREA:2.57e7
S/N:16.4

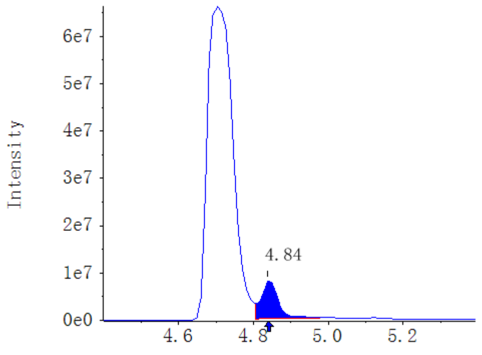

A21233258b_b
Naringenin chalcone AREA:9.89e6
S/N:14.0

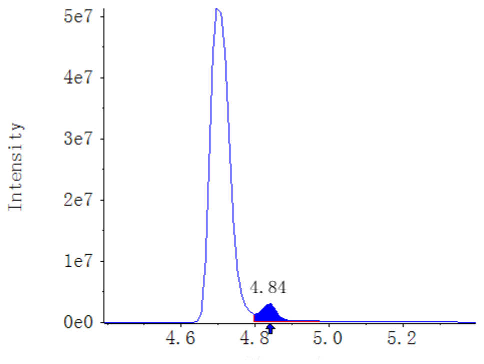

A21233259b_b
Naringenin chalcone AREA:4.95e5
S/N:16.9

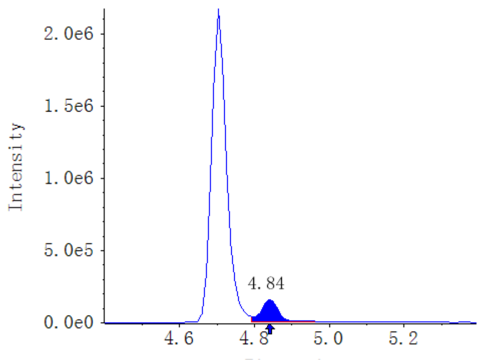

A21233260b_b
Naringenin chalcone AREA:1.58e4
S/N:13.4

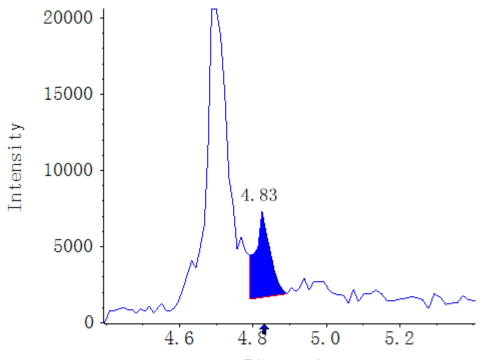

A21233261b_b
Naringenin chalcone AREA:1.25e5
S/N:21.0

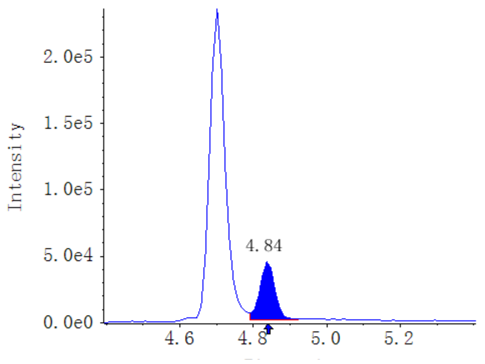

A21233262b_b
Naringenin chalcone AREA:7.93e6
S/N:38.9

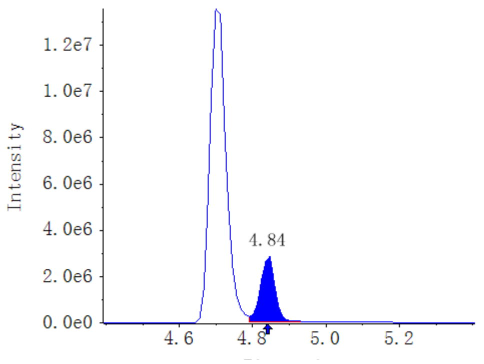

A21233263b_b
Naringenin chalcone AREA:6.16e7
S/N:23.8

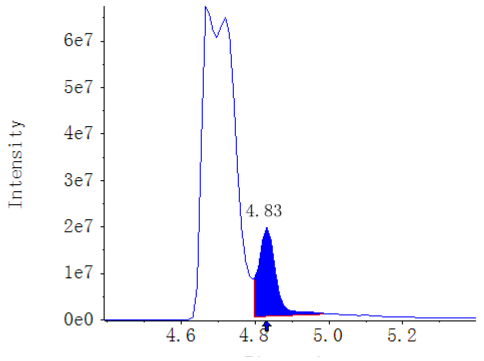

A21233264b_b
Naringenin chalcone AREA:7.72e7
S/N:23.2

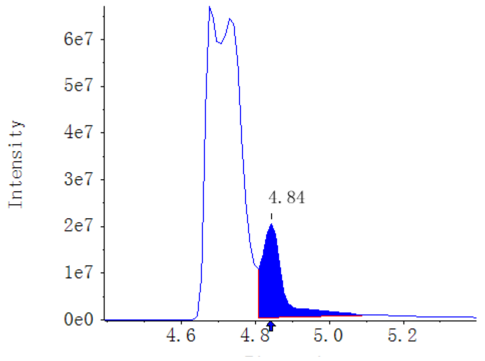

A21233265b_b
Naringenin chalcone AREA:3.80e7
S/N:16.8

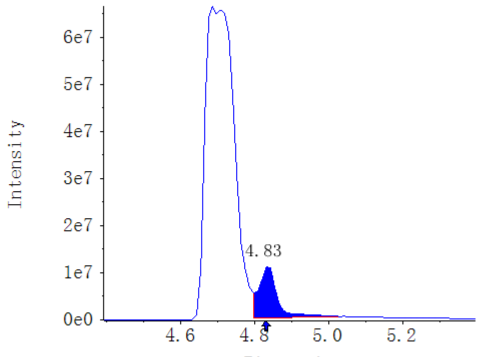

A21233266b_b
Naringenin chalcone AREA:4.29e7
S/N:20.8

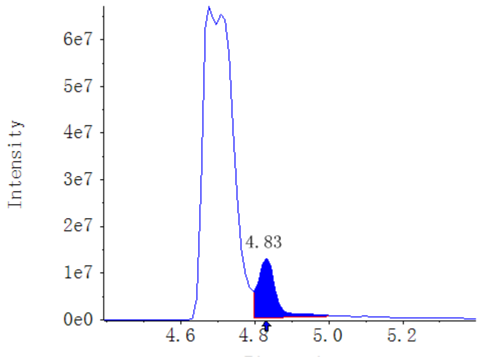

A21233267b_b
Naringenin chalcone AREA:2.44e7
S/N:15.9

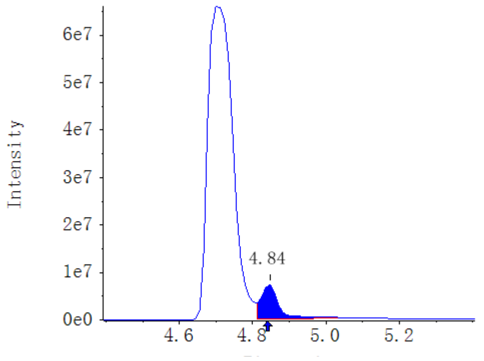

Result Table	MWXS-21-2101D-3_18_WH6500-5_A20-3_V1.0_TY_20211028	Algorithm Used	MQ4
Acquisition Method	Flavonoids_V1.0_WH6500-5_LT_20211025.dam	Instrument Name	QTRAP 6500+ Low Mass
Project	N/A	Analytes QTY	204:161

Compound name: (-)-Gallocatechin (305.1 / 125.0)

Sample Name	Sample Type	Area (cps)	Is Area (cps)	RT (min)	S/N	Target Conc	Calculated Conc.()
STD_0.5nM	Standard	2.17e3	N/A	1.67	9.6	0.5000	3.596021e-1
STD_1nM	Standard	3.50e3	N/A	1.67	11.1	1.0000	8.923617e-1
STD_5nM	Standard	1.91e4	N/A	1.66	40.7	5.0000	7.110249e0
STD_10nM	Standard	2.62e4	N/A	1.67	54.7	10.0000	9.979292e0
STD_20nM	Standard	4.93e4	N/A	1.66	63.3	20.0000	1.918175e1
STD_50nM	Standard	1.29e5	N/A	1.66	109.2	50.0000	5.097309e1
STD_100nM	Standard	2.51e5	N/A	1.67	88.5	100.0000	9.997751e1
STD_200nM	Standard	4.97e5	N/A	1.66	108.3	200.0000	1.980261e2
STD_500nM	Standard	N/A	N/A	N/A	N/A	500.0000	N/A
STD_1000nM	Standard	N/A	N/A	N/A	N/A	1000.0000	N/A
STD_2000nM	Standard	N/A	N/A	N/A	N/A	2000.0000	N/A
V1.0_MW_RQC1_20211018	Quality Control	9.32e3	N/A	1.66	6.6	0.0000	3.219723e0
Blank	Unknown	N/A	N/A	N/A	N/A	N/A	N/A
V1.0_MWMS_20211021_1	Unknown	4.93e5	N/A	1.67	108.8	N/A	1.966229e2
MWXS212101D3_R1	Quality Control	5.15e5	N/A	1.66	130.9	0.0000	2.053009e2
MWXS212101D3_R2	Quality Control	5.04e5	N/A	1.66	102.7	0.0000	2.007614e2
MWXS212101D3_R3	Quality Control	4.96e5	N/A	1.67	113.3	0.0000	1.978122e2
A21233250b_b	Unknown	3.23e4	N/A	1.66	27.0	N/A	1.240587e1
A21233251b_b	Unknown	9.56e3	N/A	1.66	11.3	N/A	3.313340e0
A21233252b_b	Unknown	5.62e3	N/A	1.66	11.4	N/A	1.740240e0
A21233253b_b	Unknown	1.42e4	N/A	1.69	9.2	N/A	5.165340e0
A21233254b_b	Unknown	1.94e4	N/A	1.69	12.3	N/A	7.241347e0
A21233255b_b	Unknown	1.81e4	N/A	1.69	15.1	N/A	6.716384e0
A21233256b_b	Unknown	2.49e4	N/A	1.69	10.3	N/A	9.457888e0
A21233257b_b	Unknown	1.79e4	N/A	1.69	11.1	N/A	6.649387e0
A21233258b_b	Unknown	3.73e4	N/A	1.69	16.4	N/A	1.438324e1
A21233259b_b	Unknown	2.66e4	N/A	1.67	21.4	N/A	1.014180e1
A21233260b_b	Unknown	3.36e4	N/A	1.66	21.3	N/A	1.290619e1
A21233261b_b	Unknown	1.34e5	N/A	1.67	37.5	N/A	5.318343e1
A21233262b_b	Unknown	1.37e4	N/A	1.69	8.5	N/A	4.984391e0
A21233263b_b	Unknown	1.84e4	N/A	1.68	8.5	N/A	6.831857e0
A21233264b_b	Unknown	1.73e4	N/A	1.67	8.9	N/A	6.390119e0
A21233265b_b	Unknown	1.62e4	N/A	1.69	10.1	N/A	5.983787e0
A21233266b_b	Unknown	1.05e4	N/A	1.68	8.6	N/A	3.680123e0
A21233267b_b	Unknown	1.84e4	N/A	1.69	11.4	N/A	6.834675e0

Compound name: (-)-Gallocatechin
Regression Equation: y = 2502.86675 x + 1265.98335 (r = 0.99862) (weighting: 1 / x)

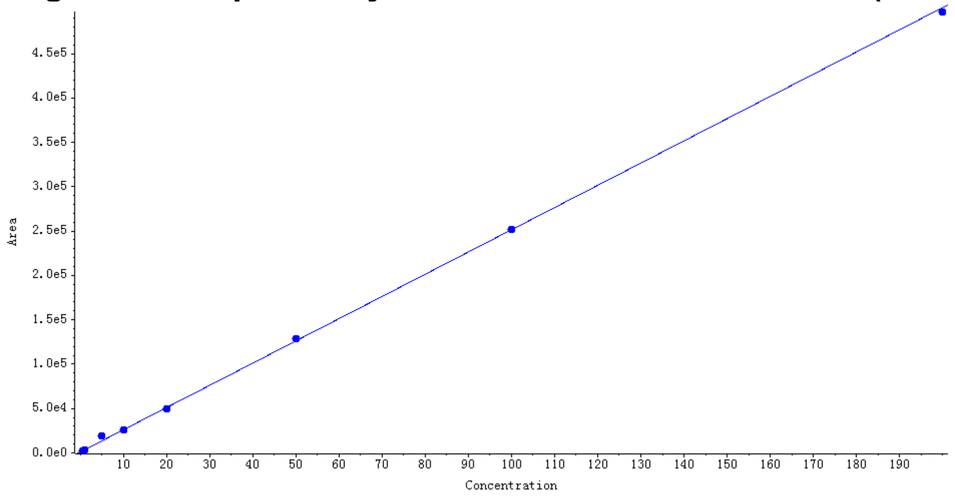

Peak Review

Blank
(-)-Gallocatechin AREA:N/A
S/N:N/A

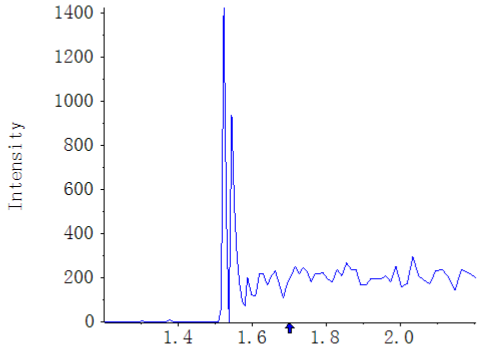

V1.0_MWMS_20211021_1
(-)-Gallocatechin AREA:4.93e5
S/N:108.8

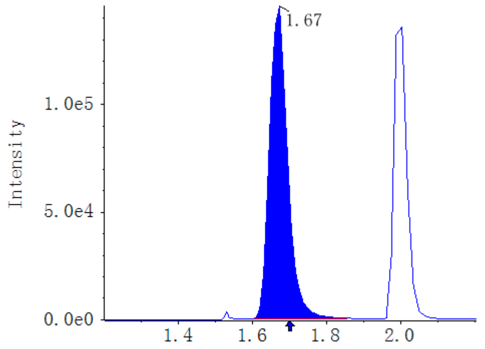

A21233250b_b
(-)-Gallocatechin AREA:3.23e4
S/N:27.0

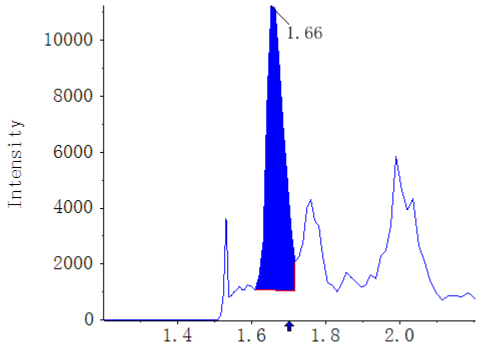

A21233251b_b
(-)-Gallocatechin AREA:9.56e3
S/N:11.3

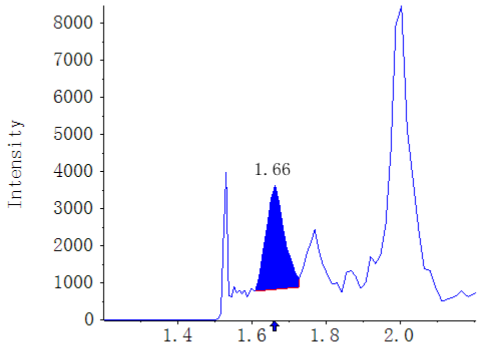

A21233252b_b
(-)-Gallocatechin AREA:5.62e3
S/N:11.4

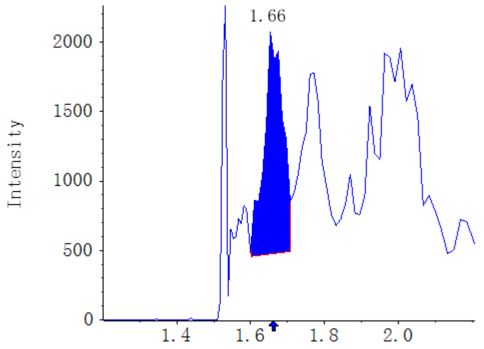

A21233253b_b
(-)-Gallocatechin AREA:1.42e4
S/N:9.2

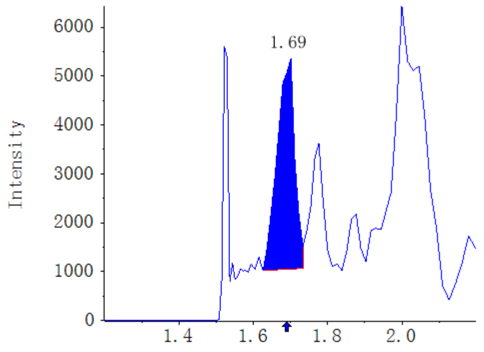

A21233254b_b
(-)-Gallocatechin AREA:1.94e4
S/N:12.3

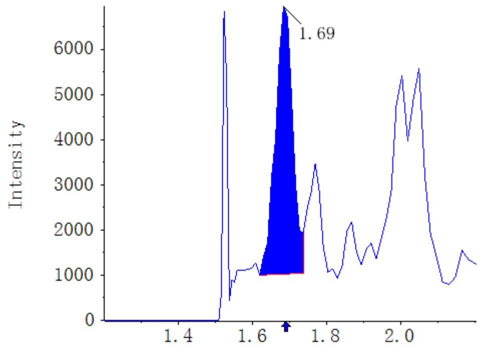

A21233255b_b
(-)-Gallocatechin AREA:1.81e4
S/N:15.1

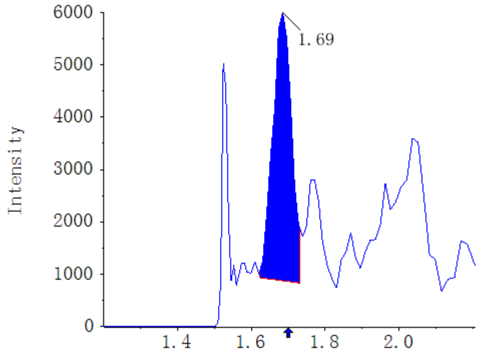

A21233256b_b
(-)-Gallocatechin AREA:2.49e4
S/N:10.3

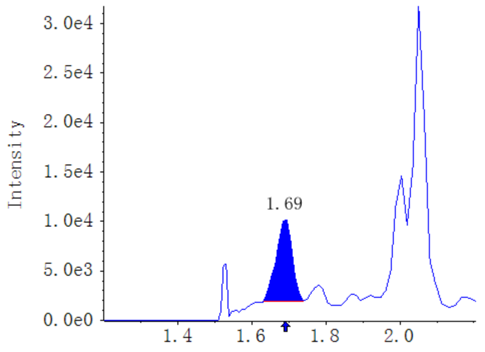

A21233257b_b
(-)-Gallocatechin AREA:1.79e4
S/N:11.1

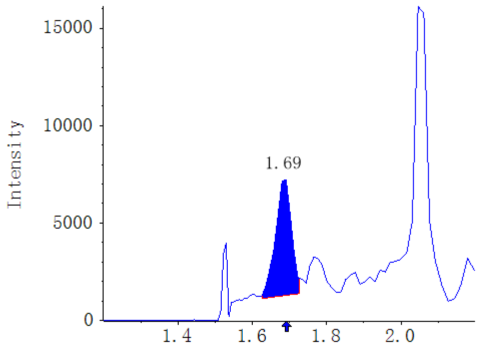

A21233258b_b
(-)-Gallocatechin AREA:3.73e4
S/N:16.4

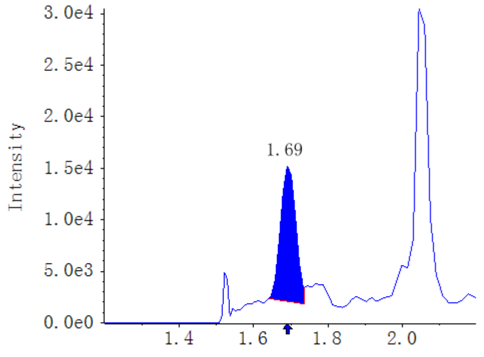

A21233259b_b
(-)-Gallocatechin AREA:2.66e4
S/N:21.4

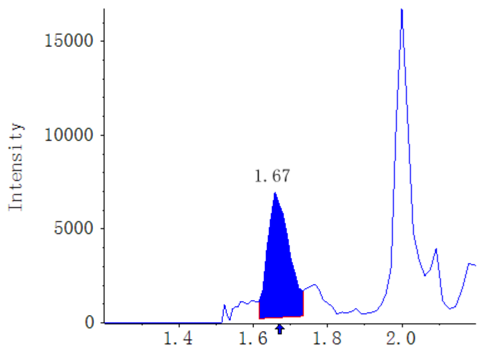

A21233260b_b
(-)-Gallocatechin AREA:3.36e4
S/N:21.3

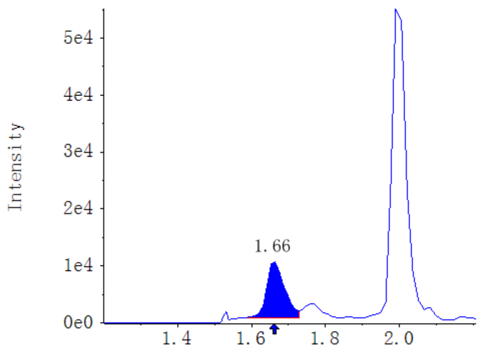

A21233261b_b
(-)-Gallocatechin AREA:1.34e5
S/N:37.5

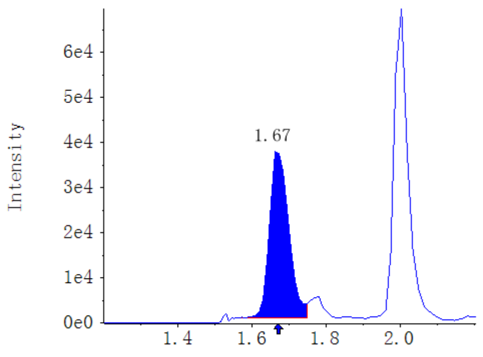

A21233262b_b
(-)-Gallocatechin AREA:1.37e4
S/N:8.5

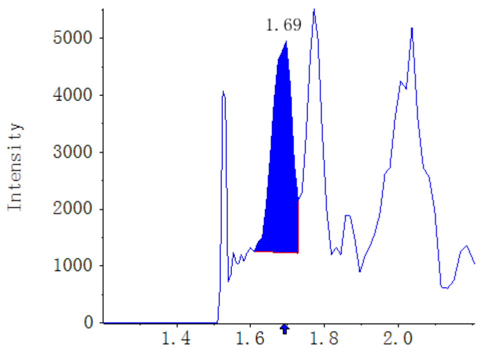

A21233263b_b
(-)-Gallocatechin AREA:1.84e4
S/N:8.5

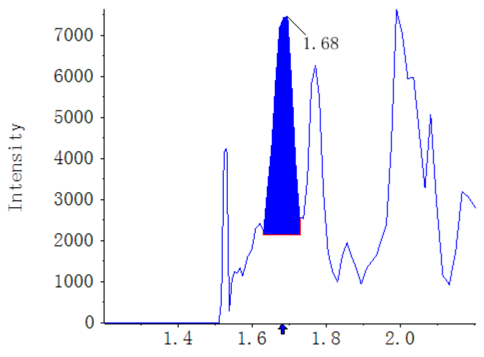

A21233264b_b
(-)-Gallocatechin AREA:1.73e4
S/N:8.9

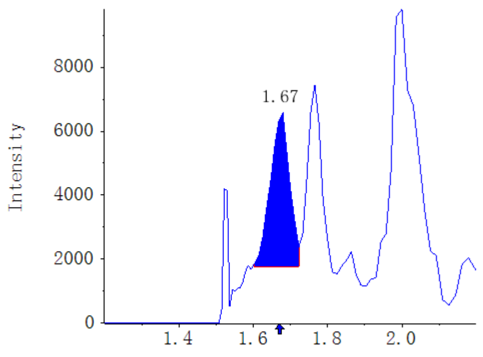

A21233265b_b
(-)-Gallocatechin AREA:1.62e4
S/N:10.1

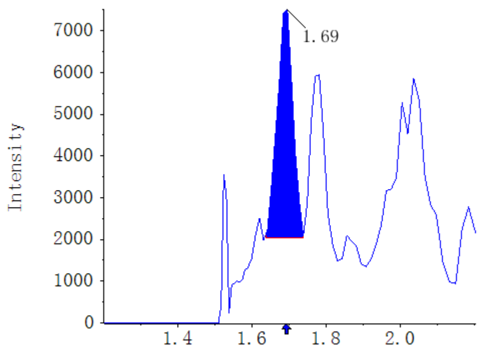

A21233266b_b
(-)-Gallocatechin AREA:1.05e4
S/N:8.6

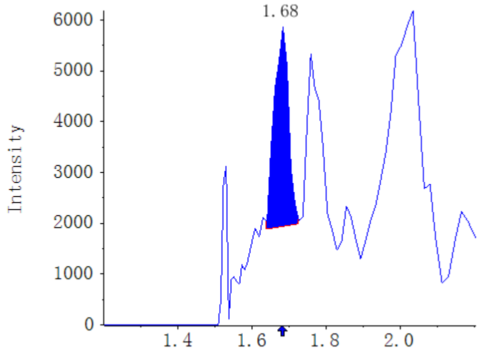

A21233267b_b
(-)-Gallocatechin AREA:1.84e4
S/N:11.4

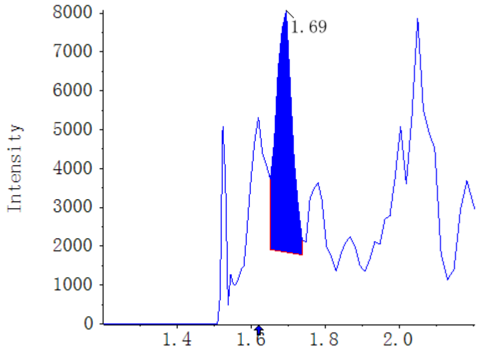

**Result Table**	MWXS-21-2101D-3_18_WH6500-5_A20-3_V1.0_TY_20211028	**Algorithm Used**	MQ4
**Acquisition Method**	Flavonoids_V1.0_WH6500-5_LT_20211025.dam	**Instrument Name**	QTRAP 6500+ Low Mass
**Project**	N/A	**Analytes QTY**	204:163

Compound name: Isorhamnetin 3-O-glucoside (477.1 / 314.1)

Sample Name	Sample Type	Area (cps)	Is Area (cps)	RT (min)	S/N	Target Conc	Calculated Conc.()
STD_0.5nM	Standard	3.85e3	N/A	3.04	33.0	0.5000	3.113017e-1
STD_1nM	Standard	1.23e4	N/A	3.03	64.4	1.0000	1.038809e0
STD_5nM	Standard	7.73e4	N/A	3.04	98.2	5.0000	6.651810e0
STD_10nM	Standard	1.16e5	N/A	3.03	197.3	10.0000	1.001918e1
STD_20nM	Standard	2.23e5	N/A	3.03	375.7	20.0000	1.920388e1
STD_50nM	Standard	6.19e5	N/A	3.03	323.5	50.0000	5.339997e1
STD_100nM	Standard	1.16e6	N/A	3.04	298.8	100.0000	9.974778e1
STD_200nM	Standard	2.27e6	N/A	3.03	292.7	200.0000	1.961273e2
STD_500nM	Standard	N/A	N/A	N/A	N/A	500.0000	N/A
STD_1000nM	Standard	N/A	N/A	N/A	N/A	1000.0000	N/A
STD_2000nM	Standard	N/A	N/A	N/A	N/A	2000.0000	N/A
V1.0_MW_RQC1_20211018	Quality Control	7.81e5	N/A	3.02	44.4	0.0000	6.734375e1
Blank	Unknown	N/A	N/A	N/A	N/A	N/A	N/A
V1.0_MWMS_20211021_1	Unknown	2.10e6	N/A	3.05	332.0	N/A	1.807994e2
MWXS212101D3_R1	Quality Control	2.14e6	N/A	3.04	326.8	0.0000	1.844823e2
MWXS212101D3_R2	Quality Control	2.09e6	N/A	3.05	378.3	0.0000	1.799764e2
MWXS212101D3_R3	Quality Control	1.98e6	N/A	3.05	322.7	0.0000	1.705918e2
A21233250b_b	Unknown	7.27e3	N/A	3.04	10.2	N/A	6.065366e-1
A21233251b_b	Unknown	2.53e3	N/A	3.06	9.4	N/A	1.979938e-1
A21233252b_b	Unknown	1.88e3	N/A	3.04	8.7	N/A	1.412089e-1
A21233253b_b	Unknown	1.20e4	N/A	3.04	19.6	N/A	1.012767e0
A21233254b_b	Unknown	N/A	N/A	N/A	N/A	N/A	N/A
A21233255b_b	Unknown	N/A	N/A	N/A	N/A	N/A	N/A
A21233256b_b	Unknown	5.29e3	N/A	3.05	10.1	N/A	4.357080e-1
A21233257b_b	Unknown	N/A	N/A	N/A	N/A	N/A	N/A
A21233258b_b	Unknown	N/A	N/A	N/A	N/A	N/A	N/A
A21233259b_b	Unknown	N/A	N/A	N/A	N/A	N/A	N/A
A21233260b_b	Unknown	3.69e3	N/A	3.04	9.5	N/A	2.980190e-1
A21233261b_b	Unknown	2.36e3	N/A	3.03	8.3	N/A	1.834247e-1
A21233262b_b	Unknown	3.08e3	N/A	3.04	7.5	N/A	2.452467e-1
A21233263b_b	Unknown	N/A	N/A	N/A	N/A	N/A	N/A
A21233264b_b	Unknown	N/A	N/A	N/A	N/A	N/A	N/A
A21233265b_b	Unknown	N/A	N/A	N/A	N/A	N/A	N/A
A21233266b_b	Unknown	N/A	N/A	N/A	N/A	N/A	N/A
A21233267b_b	Unknown	N/A	N/A	N/A	N/A	N/A	N/A

Compound name: Isorhamnetin 3-O-glucoside
Regression Equation: y = 11590.52408 x + 238.59017 (r = 0.99870) (weighting: 1 / x)

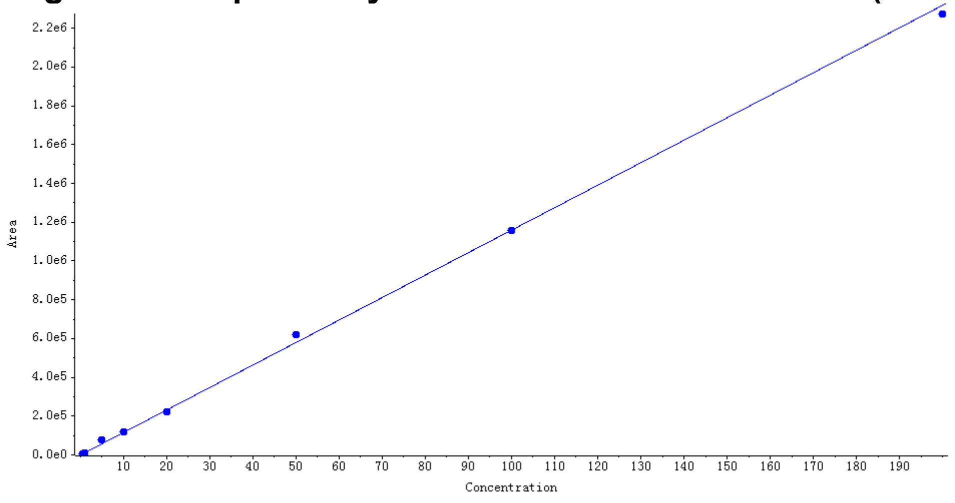

Peak Review

Blank
Isorhamnetin 3-O-glucoside
AREA:N/A S/N:N/A

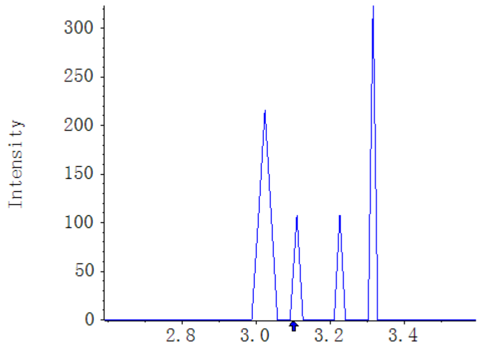

V1.0_MWMS_20211021_1
Isorhamnetin 3-O-glucoside
AREA:2.10e6 S/N:332.0

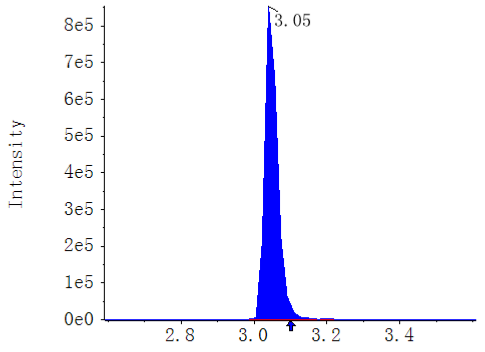

A21233250b_b
Isorhamnetin 3-O-glucoside
AREA:7.27e3 S/N:10.2

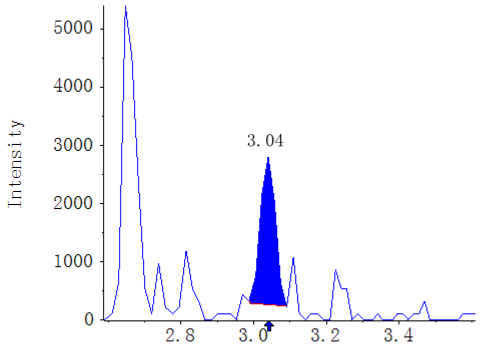

A21233251b_b
Isorhamnetin 3-O-glucoside
AREA:2.53e3 S/N:9.4

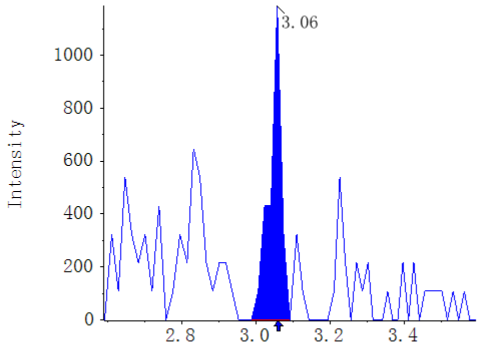

A21233252b_b
Isorhamnetin 3-O-glucoside
AREA:1.88e3 S/N:8.7

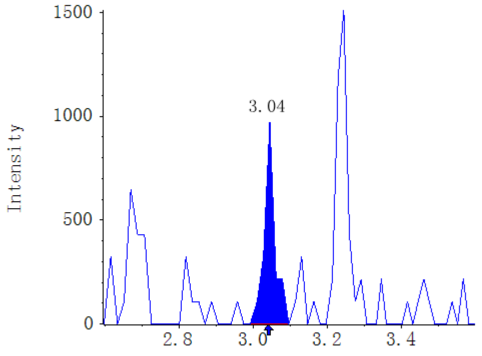

A21233253b_b
Isorhamnetin 3-O-glucoside
AREA:1.20e4 S/N:19.6

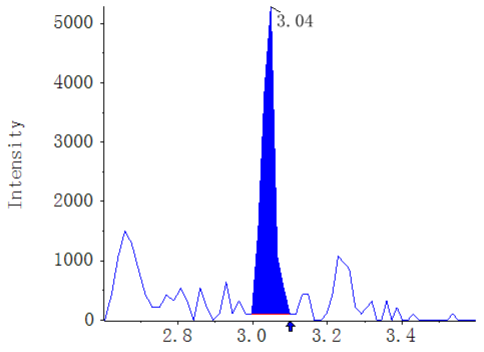

A21233254b_b
Isorhamnetin 3-O-glucoside
AREA:N/A S/N:N/A

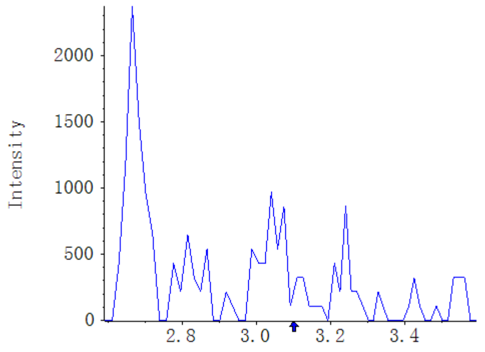

A21233255b_b
Isorhamnetin 3-O-glucoside
AREA:N/A S/N:N/A

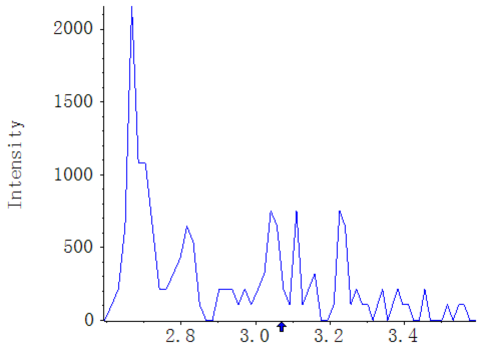

A21233256b_b
Isorhamnetin 3-O-glucoside
AREA:5.29e3 S/N:10.1

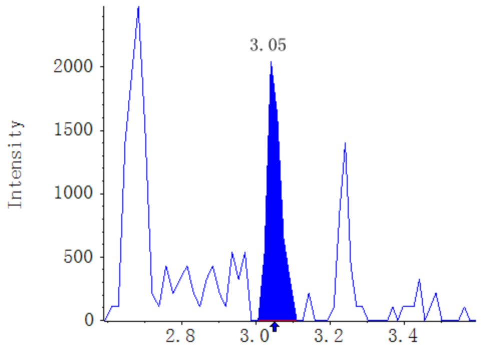

A21233257b_b
Isorhamnetin 3-O-glucoside
AREA:N/A S/N:N/A

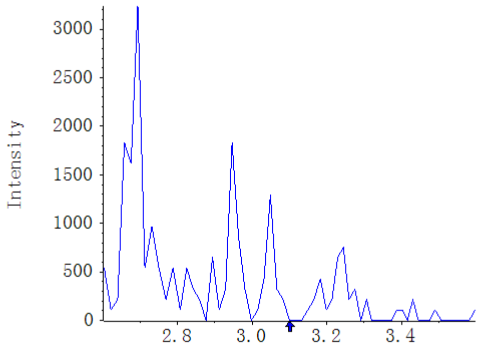

A21233258b_b
Isorhamnetin 3-O-glucoside
AREA:N/A S/N:N/A

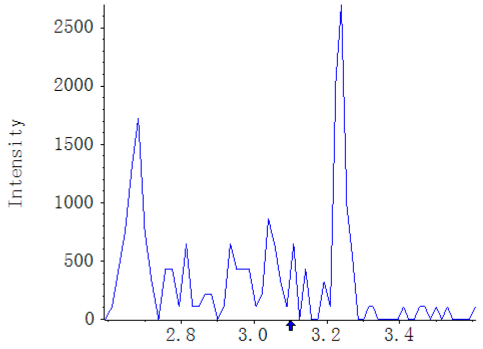

A21233259b_b
Isorhamnetin 3-O-glucoside
AREA:N/A S/N:N/A

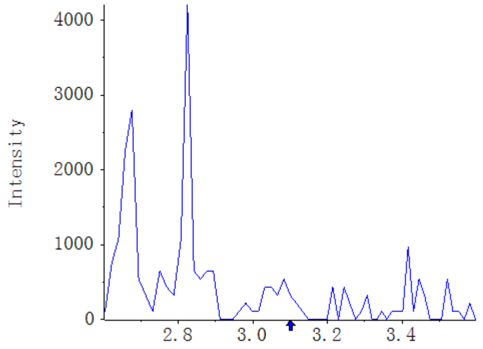

A21233260b_b
Isorhamnetin 3-O-glucoside
AREA:3.69e3 S/N:9.5

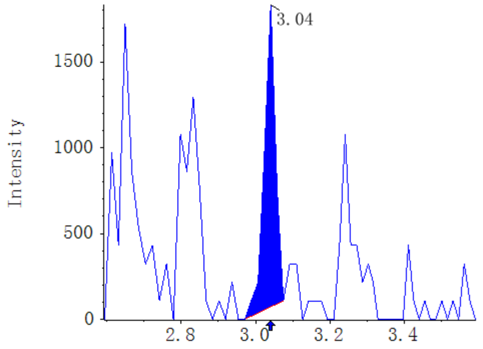

A21233261b_b
Isorhamnetin 3-O-glucoside
AREA:2.36e3 S/N:8.3

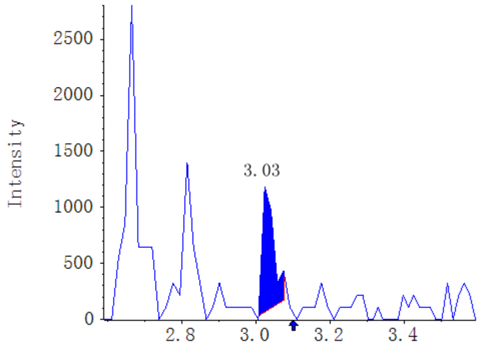

A21233262b_b
Isorhamnetin 3-O-glucoside
AREA:3.08e3 S/N:7.5

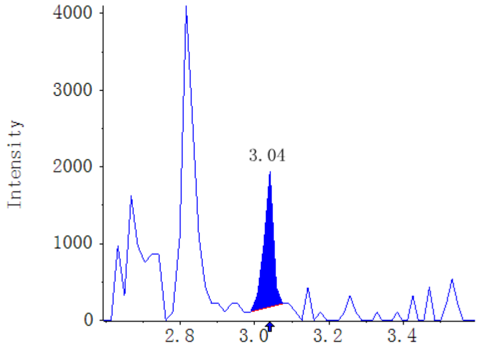

A21233263b_b
Isorhamnetin 3-O-glucoside
AREA:N/A S/N:N/A

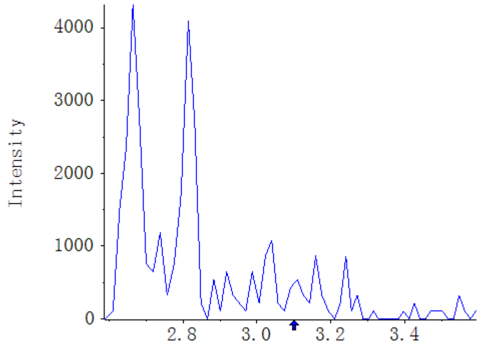

A21233264b_b
Isorhamnetin 3-O-glucoside
AREA:N/A S/N:N/A

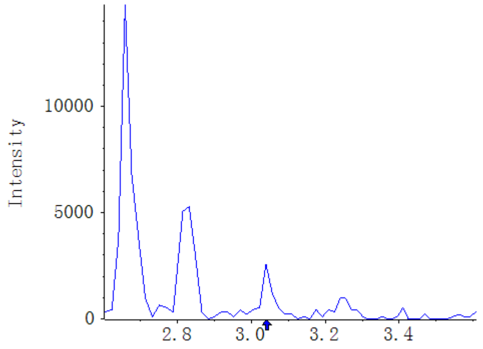

A21233265b_b
Isorhamnetin 3-O-glucoside
AREA:N/A S/N:N/A

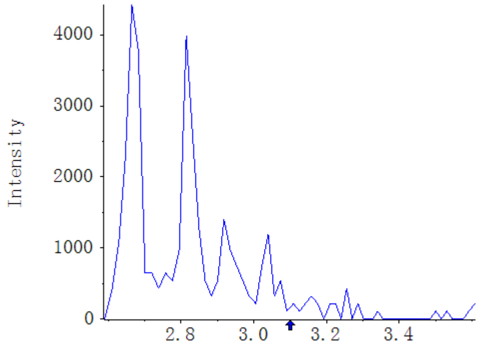

A21233266b_b
Isorhamnetin 3-O-glucoside
AREA:N/A S/N:N/A

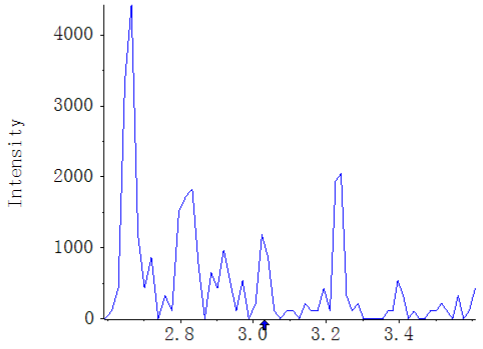

A21233267b_b
Isorhamnetin 3-O-glucoside
AREA:N/A S/N:N/A

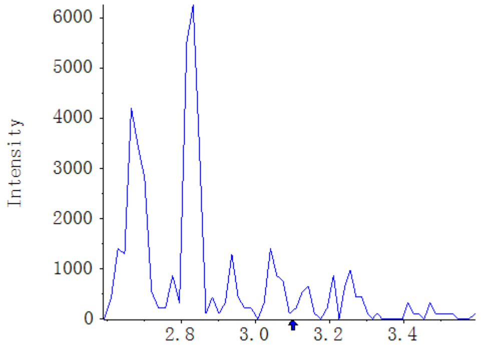

**Result Table**	MWXS-21-2101D-3_18_WH6500-5_A20-3_V1.0_TY_20211028	**Algorithm Used**	MQ4
**Acquisition Method**	Flavonoids_V1.0_WH6500-5_LT_20211025.dam	**Instrument Name**	QTRAP 6500+ Low Mass
**Project**	N/A	**Analytes QTY**	204:165

Compound name: Trilobatin (435.1 / 273.1)

Sample Name	Sample Type	Area (cps)	Is Area (cps)	RT (min)	S/N	Target Conc	Calculated Conc.()
STD_0.5nM	Standard	1.22e3	N/A	3.52	6.6	0.0500	3.179684e-2
STD_1nM	Standard	2.60e3	N/A	3.51	9.7	0.1000	8.266246e-2
STD_5nM	Standard	1.91e4	N/A	3.50	27.9	0.5000	6.879959e-1
STD_10nM	Standard	3.33e4	N/A	3.51	25.3	1.0000	1.213326e0
STD_20nM	Standard	5.43e4	N/A	3.50	28.6	2.0000	1.985017e0
STD_50nM	Standard	1.36e5	N/A	3.51	21.2	5.0000	5.007706e0
STD_100nM	Standard	2.57e5	N/A	3.51	21.6	10.0000	9.439926e0
STD_200nM	Standard	5.49e5	N/A	3.50	23.3	20.0000	2.020157e1
STD_500nM	Standard	N/A	N/A	N/A	N/A	50.0000	N/A
STD_1000nM	Standard	N/A	N/A	N/A	N/A	100.0000	N/A
STD_2000nM	Standard	N/A	N/A	N/A	N/A	200.0000	N/A
V1.0_MW_RQC1_20211018	Quality Control	1.10e4	N/A	3.50	4.4	0.0000	3.898937e-1
Blank	Unknown	N/A	N/A	N/A	N/A	N/A	N/A
V1.0_MWMS_20211021_1	Unknown	4.60e5	N/A	3.52	24.1	N/A	1.689768e1
MWXS212101D3_R1	Quality Control	4.68e5	N/A	3.52	30.7	0.0000	1.721566e1
MWXS212101D3_R2	Quality Control	4.59e5	N/A	3.52	28.7	0.0000	1.686847e1
MWXS212101D3_R3	Quality Control	4.68e5	N/A	3.51	35.0	0.0000	1.719868e1
A21233250b_b	Unknown	N/A	N/A	N/A	N/A	N/A	N/A
A21233251b_b	Unknown	N/A	N/A	N/A	N/A	N/A	N/A
A21233252b_b	Unknown	N/A	N/A	N/A	N/A	N/A	N/A
A21233253b_b	Unknown	1.95e4	N/A	3.52	2.4	N/A	7.035078e-1
A21233254b_b	Unknown	2.19e4	N/A	3.52	2.9	N/A	7.919260e-1
A21233255b_b	Unknown	2.20e4	N/A	3.51	2.6	N/A	7.981137e-1
A21233256b_b	Unknown	6.94e4	N/A	3.52	4.3	N/A	2.540351e0
A21233257b_b	Unknown	7.61e4	N/A	3.52	4.0	N/A	2.785671e0
A21233258b_b	Unknown	5.85e4	N/A	3.52	3.8	N/A	2.139559e0
A21233259b_b	Unknown	N/A	N/A	N/A	N/A	N/A	N/A
A21233260b_b	Unknown	N/A	N/A	N/A	N/A	N/A	N/A
A21233261b_b	Unknown	N/A	N/A	N/A	N/A	N/A	N/A
A21233262b_b	Unknown	7.20e3	N/A	3.52	2.2	N/A	2.517006e-1
A21233263b_b	Unknown	2.50e4	N/A	3.51	2.2	N/A	9.059338e-1
A21233264b_b	Unknown	2.10e4	N/A	3.52	3.1	N/A	7.605706e-1
A21233265b_b	Unknown	7.72e4	N/A	3.52	3.3	N/A	2.828626e0
A21233266b_b	Unknown	2.54e4	N/A	3.51	1.4	N/A	9.232907e-1
A21233267b_b	Unknown	3.29e4	N/A	3.53	1.9	N/A	1.198098e0

Compound name: Trilobatin
Regression Equation: y = 27176.15984 x + 357.05743 (r = 0.99784) (weighting: 1 / x)

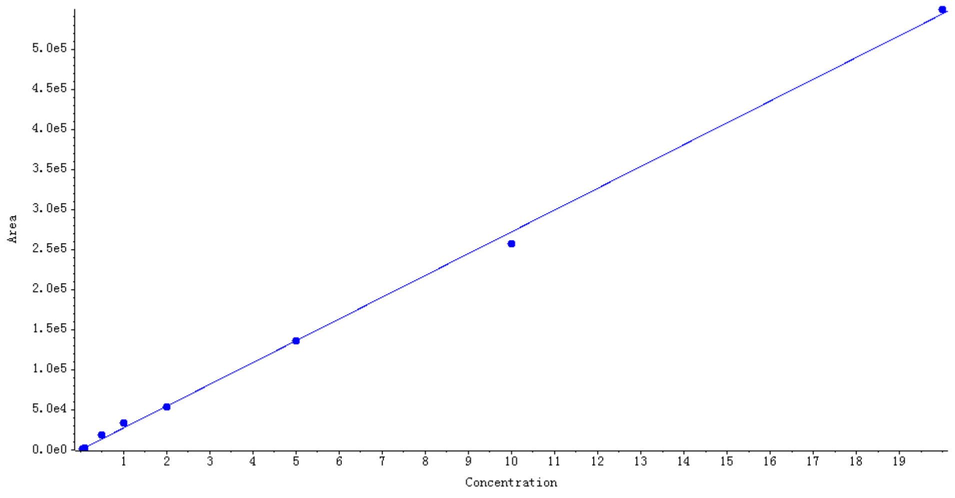

Peak Review

Blank
Trilobatin AREA:N/A S/N:N/A

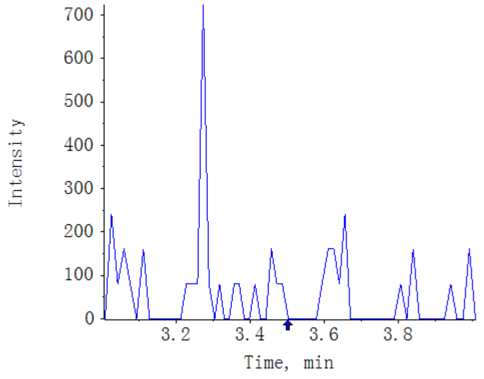

V1.0_MWMS_20211021_1
Trilobatin AREA:4.60e5 S/N:24.1

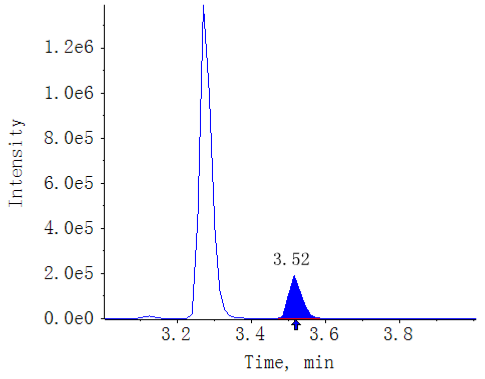

A21233250b_b
Trilobatin AREA:N/A S/N:N/A

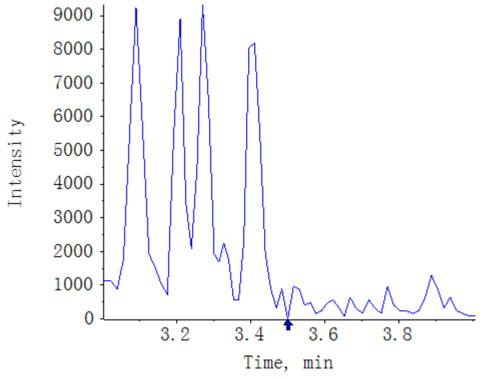

A21233251b_b
Trilobatin AREA:N/A S/N:N/A

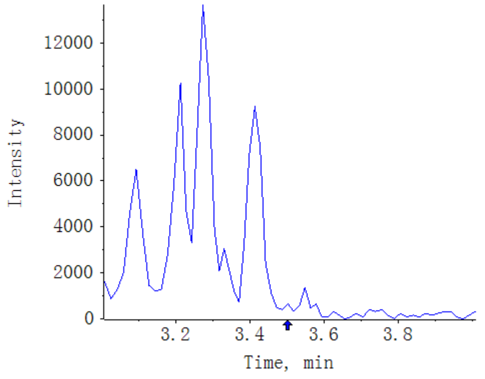

A21233252b_b
Trilobatin AREA:N/A S/N:N/A

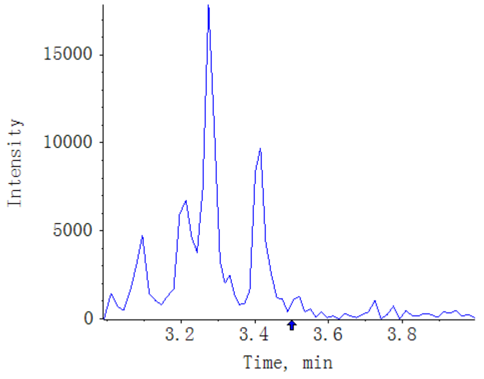

A21233253b_b
Trilobatin AREA:1.95e4 S/N:2.4

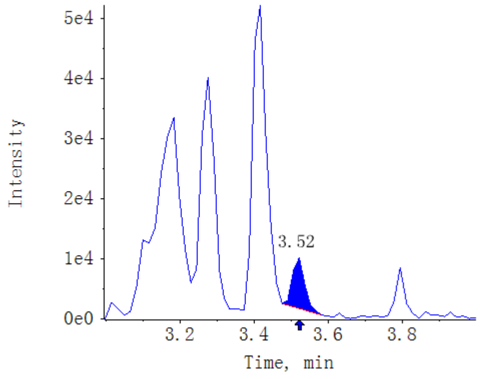

A21233254b_b
Trilobatin AREA:2.19e4 S/N:2.9

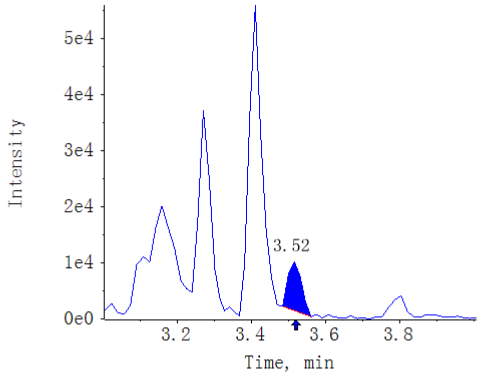

A21233255b_b
Trilobatin AREA:2.20e4 S/N:2.6

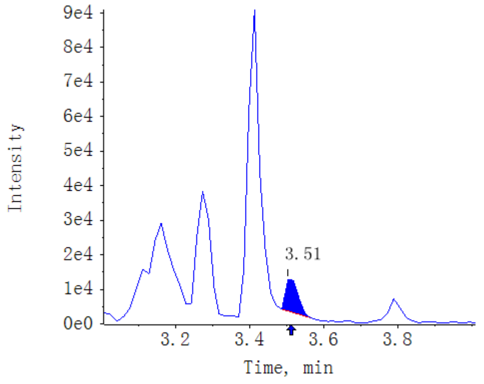

A21233256b_b
Trilobatin AREA:6.94e4 S/N:4.3

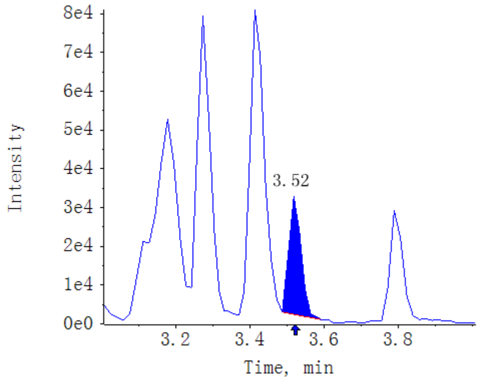

A21233257b_b
Trilobatin AREA:7.61e4 S/N:4.0

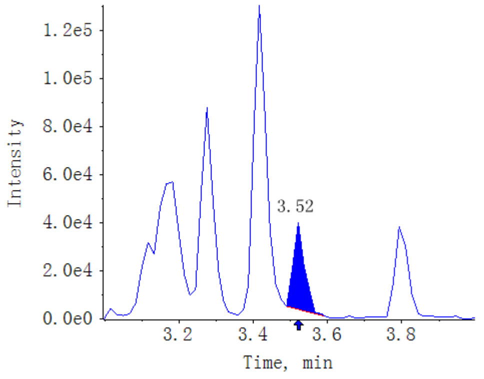

A21233258b_b
Trilobatin AREA:5.85e4 S/N:3.8

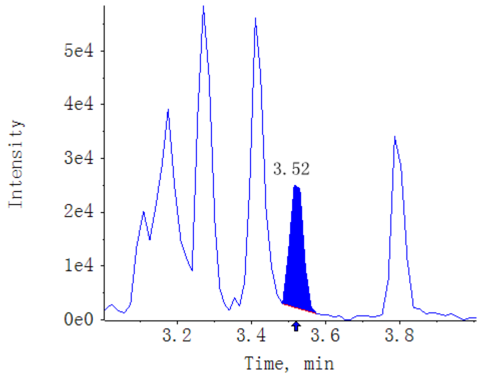

A21233259b_b
Trilobatin AREA:N/A S/N:N/A

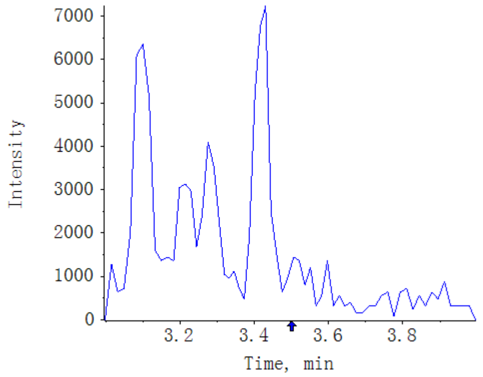

A21233260b_b
Trilobatin AREA:N/A S/N:N/A

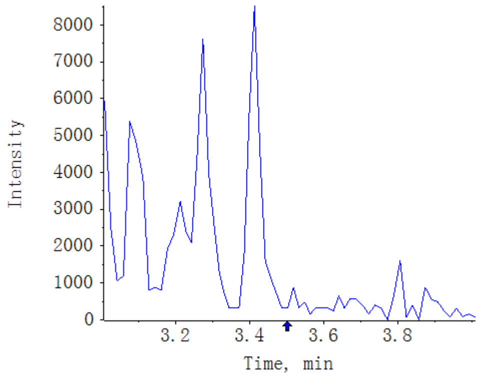

A21233261b_b
Trilobatin AREA:N/A S/N:N/A

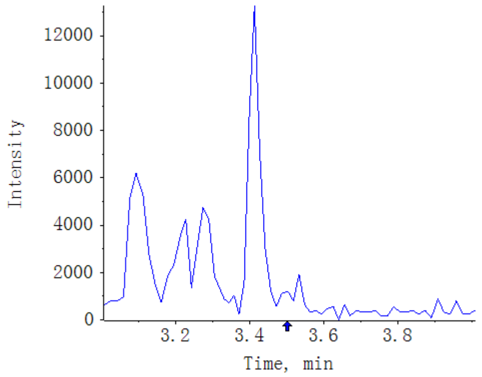

A21233262b_b
Trilobatin AREA:7.20e3 S/N:2.2

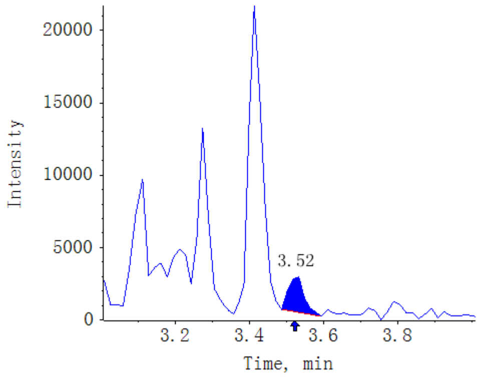

A21233263b_b
Trilobatin AREA:2.50e4 S/N:2.2

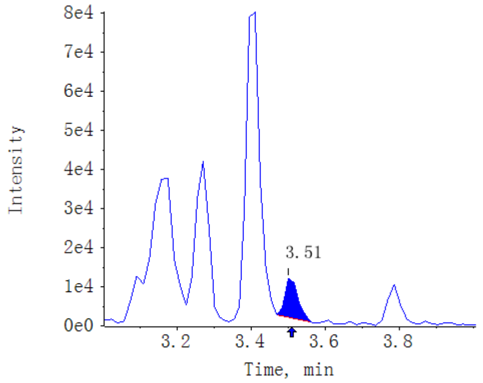

A21233264b_b
Trilobatin AREA:2.10e4 S/N:3.1

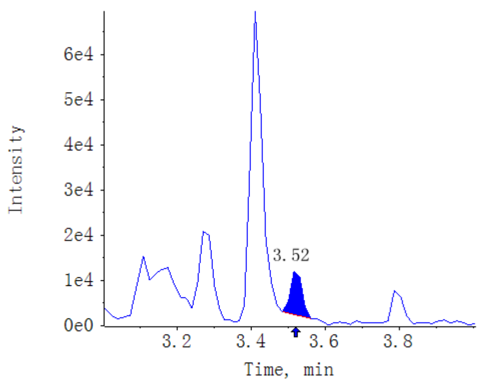

A21233265b_b
Trilobatin AREA:7.72e4 S/N:3.3

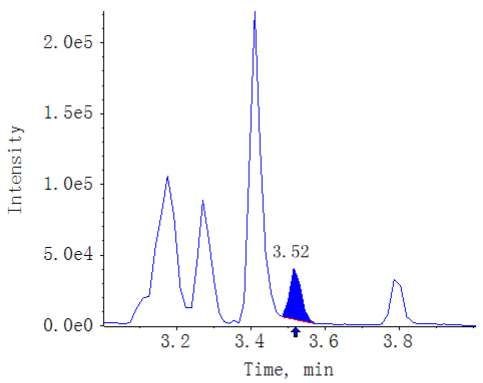

A21233266b_b
Trilobatin AREA:2.54e4 S/N:1.4

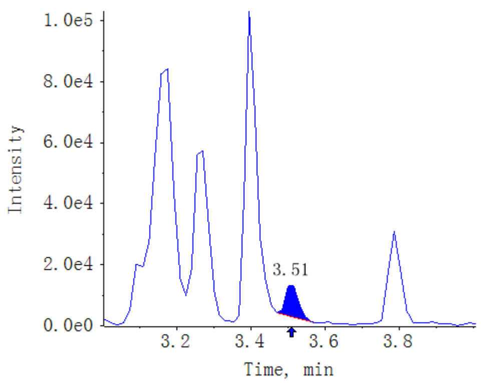

A21233267b_b
Trilobatin AREA:3.29e4 S/N:1.9

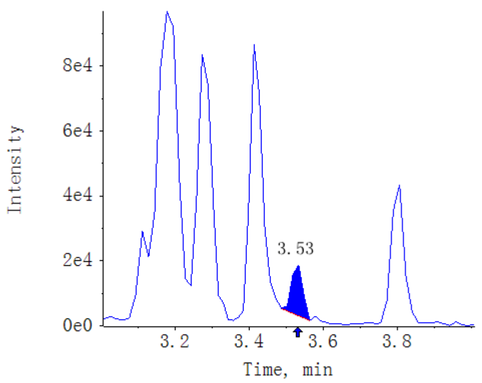

Result Table	MWXS-21-2101D-3_18_WH6500-5_A20-3_V1.0_TY_20211028	Algorithm Used	MQ4
Acquisition Method	Flavonoids_V1.0_WH6500-5_LT_20211025.dam	Instrument Name	QTRAP 6500+ Low Mass
Project	N/A	Analytes QTY	204:166

Compound name: Sieboldin (451.1 / 289.1)

Sample Name	Sample Type	Area (cps)	Is Area (cps)	RT (min)	S/N	Target Conc	Calculated Conc.()
STD_0.5nM	Standard	N/A	N/A	N/A	N/A	0.0250	N/A
STD_1nM	Standard	6.92e2	N/A	3.02	5.4	0.0500	4.008493e-2
STD_5nM	Standard	3.63e3	N/A	3.01	27.1	0.2500	3.498435e-1
STD_10nM	Standard	4.01e3	N/A	3.02	17.3	0.5000	3.894803e-1
STD_20nM	Standard	9.39e3	N/A	3.01	33.6	1.0000	9.558860e-1
STD_50nM	Standard	2.55e4	N/A	3.02	75.3	2.5000	2.650181e0
STD_100nM	Standard	4.90e4	N/A	3.02	115.6	5.0000	5.125559e0
STD_200nM	Standard	9.33e4	N/A	3.02	185.0	10.0000	9.788965e0
STD_500nM	Standard	N/A	N/A	N/A	N/A	25.0000	N/A
STD_1000nM	Standard	N/A	N/A	N/A	N/A	50.0000	N/A
STD_2000nM	Standard	N/A	N/A	N/A	N/A	100.0000	N/A
V1.0_MW_RQC1_20211018	Quality Control	N/A	N/A	N/A	N/A	0.0000	N/A
Blank	Unknown	N/A	N/A	N/A	N/A	N/A	N/A
V1.0_MWMS_20211021_1	Unknown	1.04e5	N/A	3.03	129.3	N/A	1.087916e1
MWXS212101D3_R1	Quality Control	1.05e5	N/A	3.03	147.2	0.0000	1.102168e1
MWXS212101D3_R2	Quality Control	1.15e5	N/A	3.03	138.4	0.0000	1.210400e1
MWXS212101D3_R3	Quality Control	1.10e5	N/A	3.03	151.3	0.0000	1.151412e1
A21233250b_b	Unknown	1.10e4	N/A	3.04	3.4	N/A	1.130458e0
A21233251b_b	Unknown	8.42e3	N/A	3.05	3.1	N/A	8.542608e-1
A21233252b_b	Unknown	N/A	N/A	N/A	N/A	N/A	N/A
A21233253b_b	Unknown	N/A	N/A	N/A	N/A	N/A	N/A
A21233254b_b	Unknown	N/A	N/A	N/A	N/A	N/A	N/A
A21233255b_b	Unknown	1.89e4	N/A	3.05	4.1	N/A	1.957854e0
A21233256b_b	Unknown	2.08e5	N/A	3.01	25.5	N/A	2.182624e1
A21233257b_b	Unknown	2.17e5	N/A	3.01	21.9	N/A	2.279695e1
A21233258b_b	Unknown	1.40e5	N/A	3.01	29.0	N/A	1.472782e1
A21233259b_b	Unknown	1.62e4	N/A	3.05	4.1	N/A	1.669916e0
A21233260b_b	Unknown	1.21e4	N/A	3.06	1.5	N/A	1.243929e0
A21233261b_b	Unknown	1.44e4	N/A	3.04	2.3	N/A	1.487044e0
A21233262b_b	Unknown	7.35e3	N/A	3.04	1.7	N/A	7.416132e-1
A21233263b_b	Unknown	1.74e4	N/A	3.03	3.4	N/A	1.803068e0
A21233264b_b	Unknown	1.44e4	N/A	3.03	3.5	N/A	1.483075e0
A21233265b_b	Unknown	1.17e5	N/A	3.01	9.8	N/A	1.223749e1
A21233266b_b	Unknown	7.72e4	N/A	3.00	8.3	N/A	8.094664e0
A21233267b_b	Unknown	1.48e5	N/A	3.01	16.8	N/A	1.559995e1

Compound name: Sieboldin
Regression Equation: y = 9496.31840 x + 310.98175 (r = 0.99759) (weighting: 1 / x)

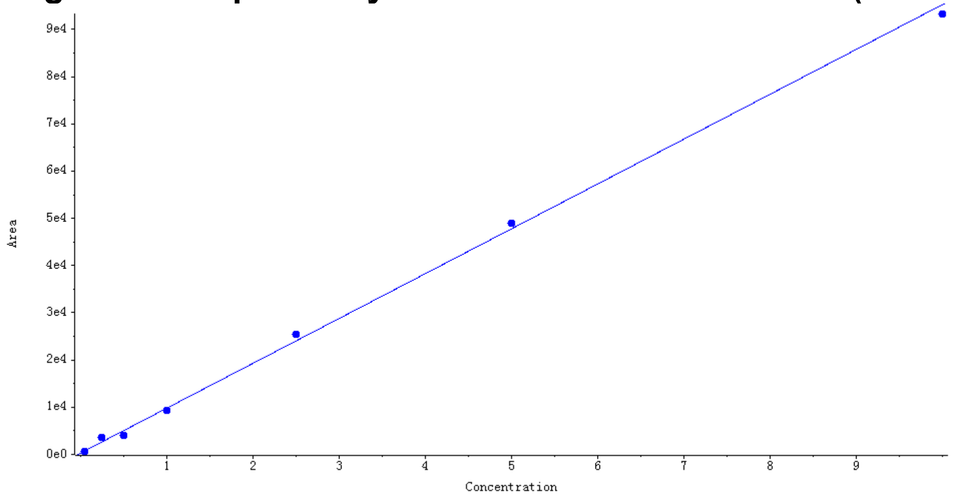

Peak Review

Blank
Sieboldin AREA:N/A S/N:N/A

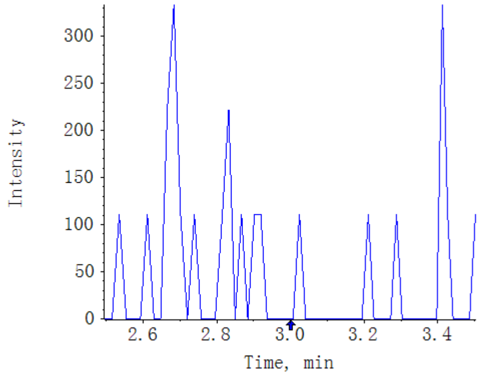

V1.0_MWMS_20211021_1
Sieboldin AREA:1.04e5 S/N:129.3

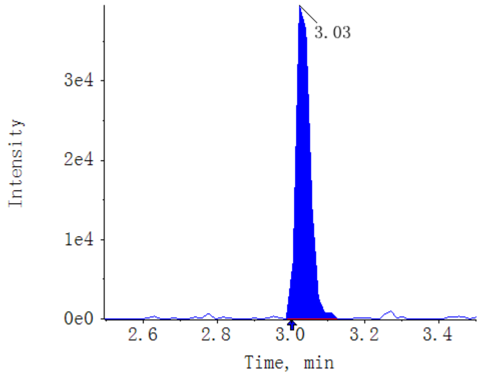

A21233250b_b
Sieboldin AREA:1.10e4 S/N:3.4

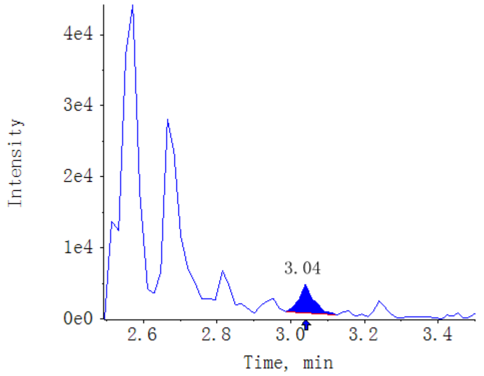

A21233251b_b
Sieboldin AREA:8.42e3 S/N:3.1

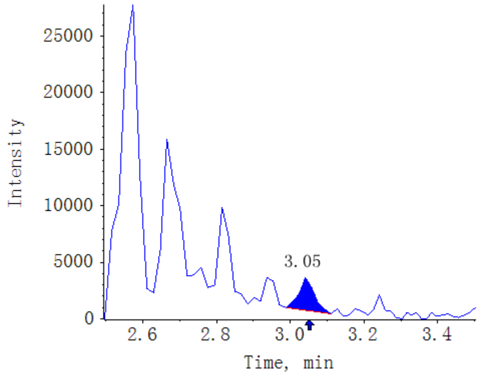

A21233252b_b
Sieboldin AREA:N/A S/N:N/A

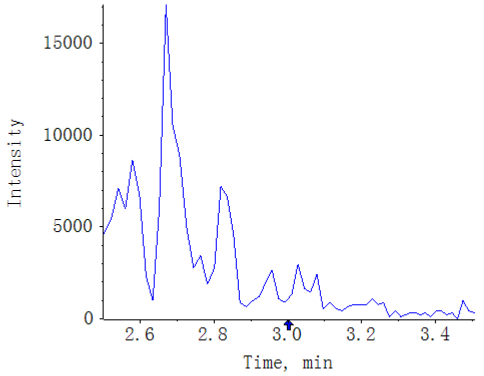

A21233253b_b
Sieboldin AREA:N/A S/N:N/A

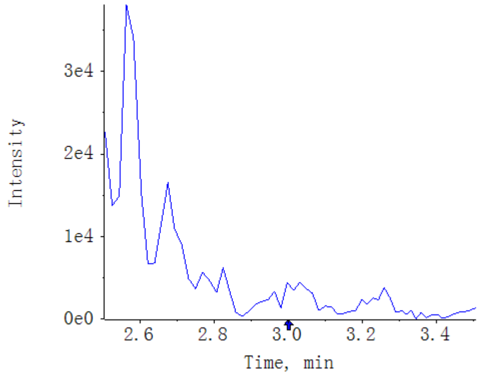

A21233254b_b
Sieboldin AREA:N/A S/N:N/A

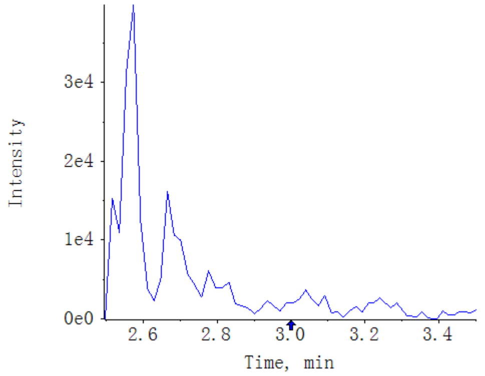

A21233255b_b
Sieboldin AREA:1.89e4 S/N:4.1

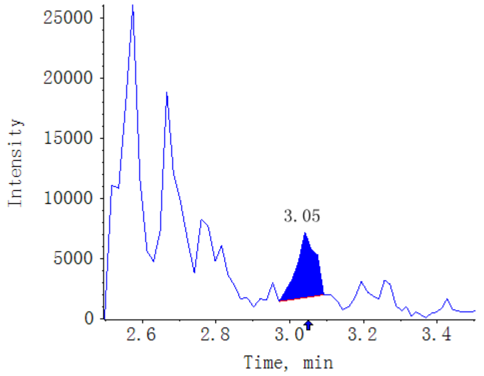

A21233256b_b
Sieboldin AREA:2.08e5 S/N:25.5

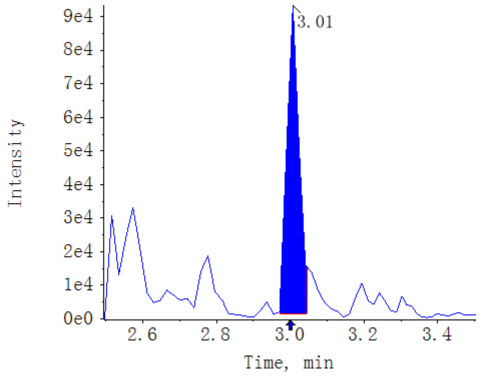

A21233257b_b
Sieboldin AREA:2.17e5 S/N:21.9

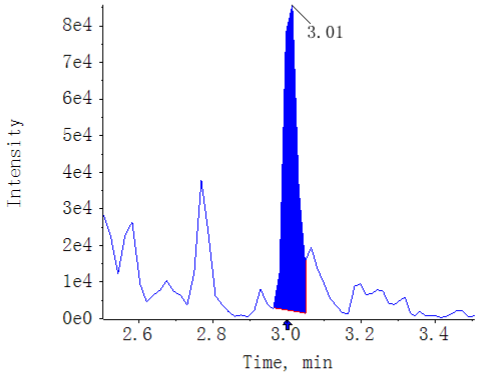

A21233258b_b
Sieboldin AREA:1.40e5 S/N:29.0

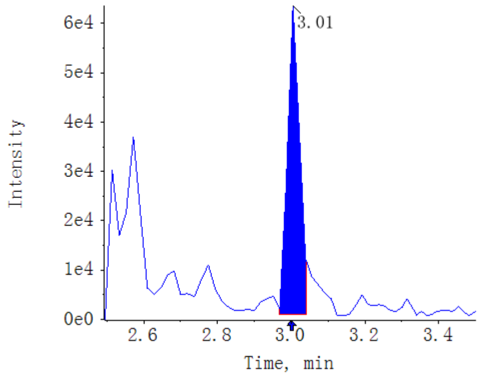

A21233259b_b
Sieboldin AREA:1.62e4 S/N:4.1

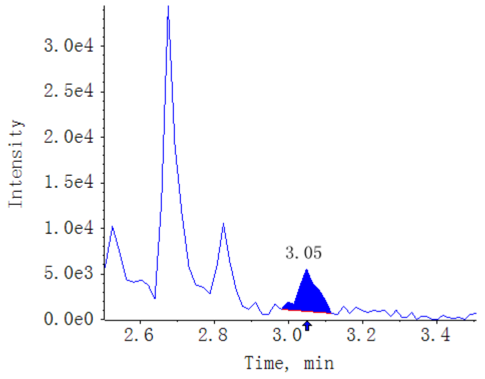

A21233260b_b
Sieboldin AREA:1.21e4 S/N:1.5

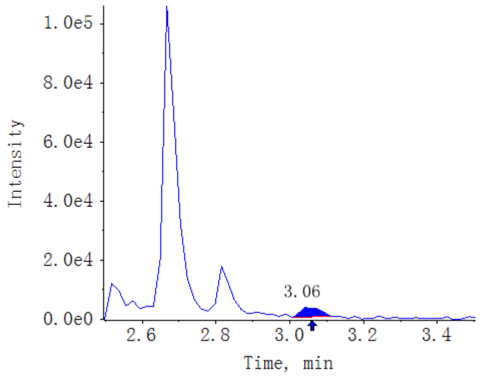

A21233261b_b
Sieboldin AREA:1.44e4 S/N:2.3

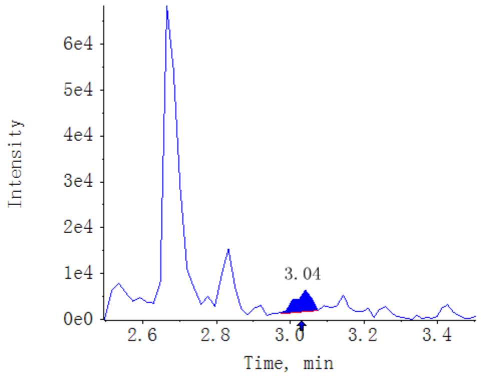

A21233262b_b
Sieboldin AREA:7.35e3 S/N:1.7

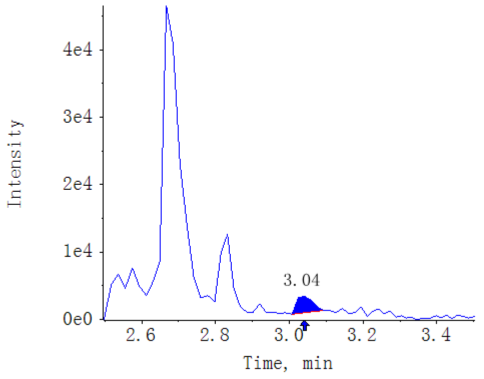

A21233263b_b
Sieboldin AREA:1.74e4 S/N:3.4

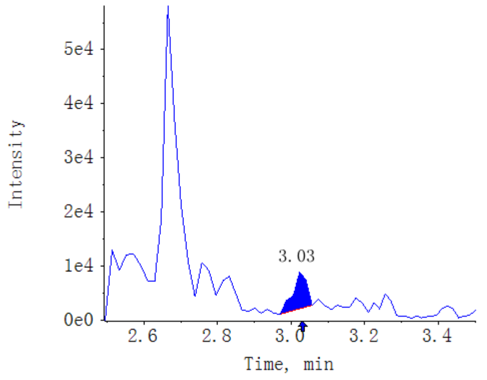

A21233264b_b
Sieboldin AREA:1.44e4 S/N:3.5

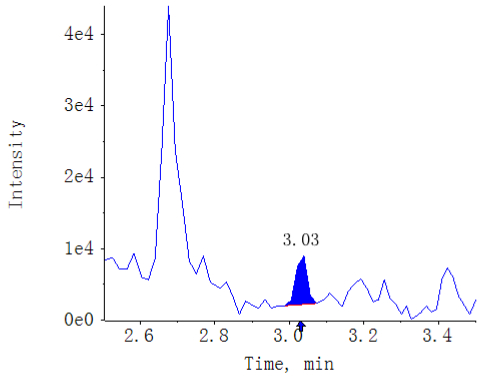

A21233265b_b
Sieboldin AREA:1.17e5 S/N:9.8

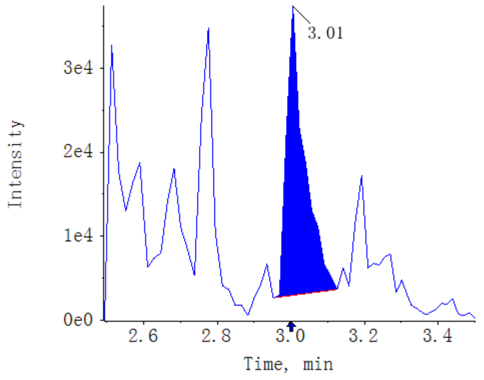

A21233266b_b
Sieboldin AREA:7.72e4 S/N:8.3

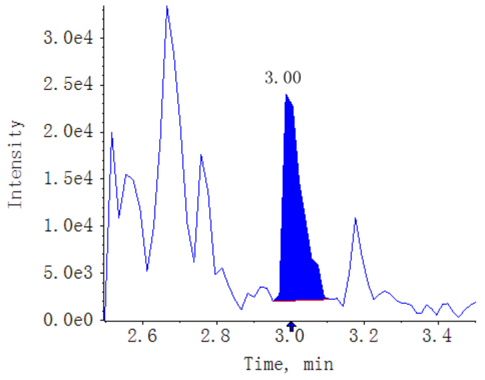

A21233267b_b
Sieboldin AREA:1.48e5 S/N:16.8

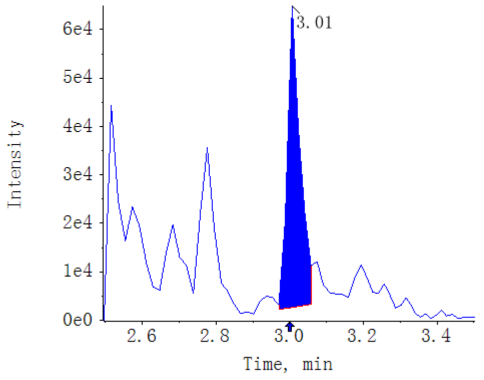

**Result Table**	MWXS-21-2101D-3_18_WH6500-5_A20-3_V1.0_TY_20211028	**Algorithm Used**	MQ4
**Acquisition Method**	Flavonoids_V1.0_WH6500-5_LT_20211025.dam	**Instrument Name**	QTRAP 6500+ Low Mass
**Project**	N/A	**Analytes QTY**	204:182

Compound name: (-)-Epigallocatechin (307.1 / 139.0)

Sample Name	Sample Type	Area (cps)	Is Area (cps)	RT (min)	S/N	Target Conc	Calculated Conc.()
STD_0.5nM	Standard	1.65e3	N/A	1.99	12.0	0.5000	4.225571e-1
STD_1nM	Standard	2.44e3	N/A	1.99	12.3	1.0000	7.405467e-1
STD_5nM	Standard	1.82e4	N/A	1.99	29.5	5.0000	7.113659e0
STD_10nM	Standard	2.53e4	N/A	2.00	36.2	10.0000	1.001431e1
STD_20nM	Standard	4.82e4	N/A	1.99	41.8	20.0000	1.926817e1
STD_50nM	Standard	1.28e5	N/A	2.00	44.9	50.0000	5.170905e1
STD_100nM	Standard	2.51e5	N/A	2.00	52.6	100.0000	1.012856e2
STD_200nM	Standard	4.84e5	N/A	1.99	49.6	200.0000	1.959461e2
STD_500nM	Standard	N/A	N/A	N/A	N/A	500.0000	N/A
STD_1000nM	Standard	N/A	N/A	N/A	N/A	1000.0000	N/A
STD_2000nM	Standard	N/A	N/A	N/A	N/A	2000.0000	N/A
V1.0_MW_RQC1_20211018	Quality Control	1.40e4	N/A	1.99	18.6	0.0000	5.442459e0
Blank	Unknown	N/A	N/A	N/A	N/A	N/A	N/A
V1.0_MWMS_20211021_1	Unknown	5.83e5	N/A	2.00	61.8	N/A	2.359297e2
MWXS212101D3_R1	Quality Control	5.96e5	N/A	1.99	62.4	0.0000	2.410853e2
MWXS212101D3_R2	Quality Control	5.90e5	N/A	2.00	61.8	0.0000	2.386751e2
MWXS212101D3_R3	Quality Control	5.83e5	N/A	2.00	62.9	0.0000	2.358256e2
A21233250b_b	Unknown	2.61e4	N/A	1.99	35.3	N/A	1.031279e1
A21233251b_b	Unknown	5.13e4	N/A	2.00	55.0	N/A	2.055935e1
A21233252b_b	Unknown	1.28e4	N/A	1.99	22.2	N/A	4.946128e0
A21233253b_b	Unknown	2.97e4	N/A	2.00	29.0	N/A	1.180743e1
A21233254b_b	Unknown	2.19e4	N/A	1.99	25.9	N/A	8.641375e0
A21233255b_b	Unknown	1.02e4	N/A	1.99	17.2	N/A	3.902611e0
A21233256b_b	Unknown	7.44e4	N/A	2.00	53.4	N/A	2.989093e1
A21233257b_b	Unknown	8.25e3	N/A	2.00	15.8	N/A	3.095583e0
A21233258b_b	Unknown	1.24e4	N/A	1.99	21.2	N/A	4.794099e0
A21233259b_b	Unknown	8.69e4	N/A	1.99	46.4	N/A	3.495406e1
A21233260b_b	Unknown	3.14e5	N/A	1.99	90.1	N/A	1.270691e2
A21233261b_b	Unknown	3.96e5	N/A	2.00	71.4	N/A	1.601714e2
A21233262b_b	Unknown	7.34e3	N/A	1.99	10.3	N/A	2.727290e0
A21233263b_b	Unknown	2.87e4	N/A	1.99	24.5	N/A	1.138773e1
A21233264b_b	Unknown	4.51e4	N/A	1.99	37.6	N/A	1.802859e1
A21233265b_b	Unknown	9.09e3	N/A	1.99	16.0	N/A	3.438428e0
A21233266b_b	Unknown	1.84e4	N/A	1.99	24.6	N/A	7.217075e0
A21233267b_b	Unknown	1.58e4	N/A	1.99	20.0	N/A	6.140334e0

Compound name: (-)-Epigallocatechin
Regression Equation: y = 2468.01720 x + 608.10898 (r = 0.99843) (weighting: 1 / x)

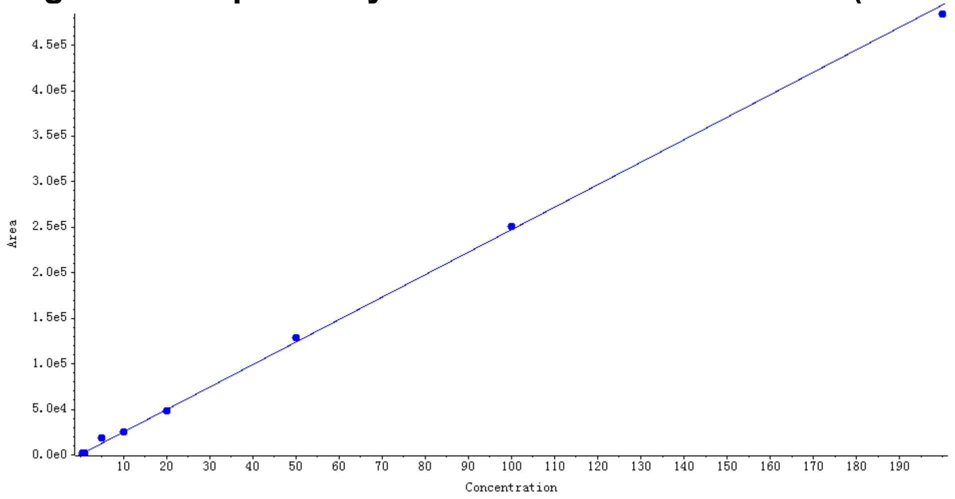

Peak Review

Blank
(-)-Epigallocatechin AREA:N/A
S/N:N/A

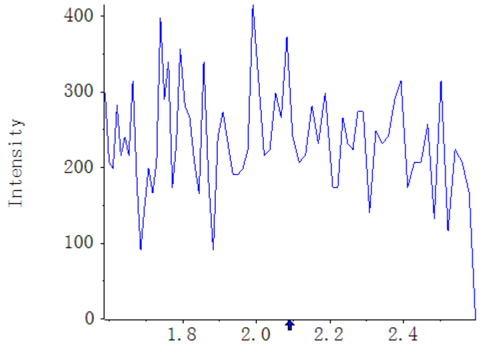

V1.0_MWMS_20211021_1
(-)-Epigallocatechin AREA:5.83e5
S/N:61.8

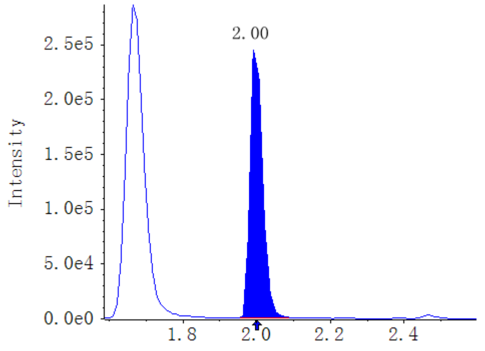

A21233250b_b
(-)-Epigallocatechin AREA:2.61e4
S/N:35.3

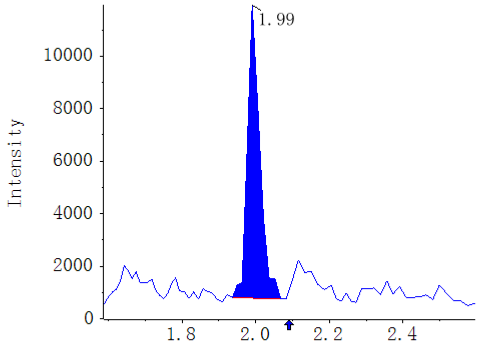

A21233251b_b
(-)-Epigallocatechin AREA:5.13e4
S/N:55.0

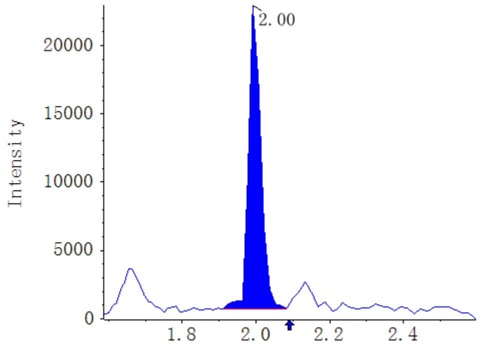

A21233252b_b
(-)-Epigallocatechin AREA:1.28e4
S/N:22.2

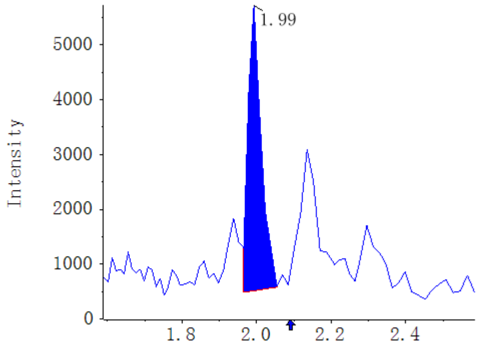

A21233253b_b
(-)-Epigallocatechin AREA:2.97e4
S/N:29.0

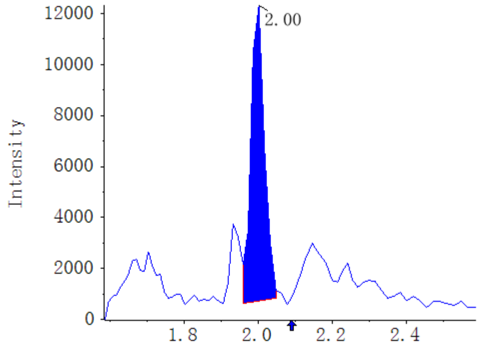

A21233254b_b
(-)-Epigallocatechin AREA:2.19e4
S/N:25.9

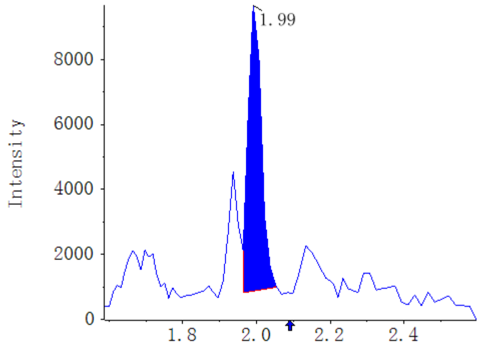

A21233255b_b
(-)-Epigallocatechin AREA:1.02e4
S/N:17.2

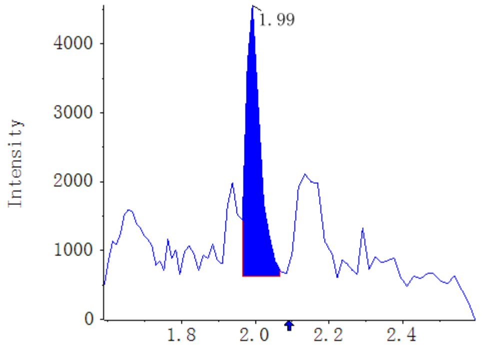

A21233256b_b
(-)-Epigallocatechin AREA:7.44e4
S/N:53.4

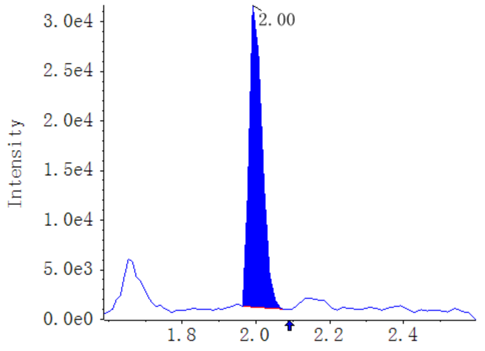

A21233257b_b
(-)-Epigallocatechin AREA:8.25e3
S/N:15.8

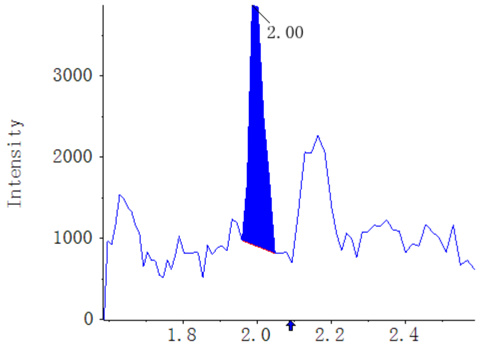

A21233258b_b
(-)-Epigallocatechin AREA:1.24e4
S/N:21.2

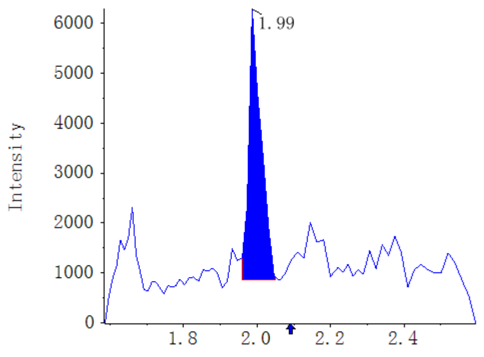

A21233259b_b
(-)-Epigallocatechin AREA:8.69e4
S/N:46.4

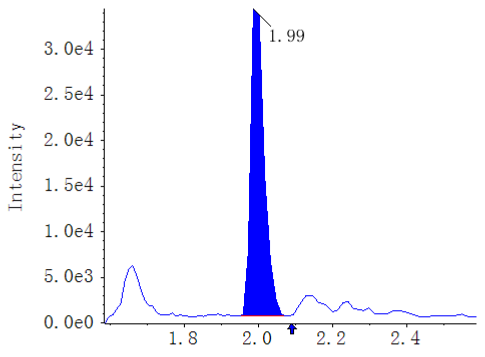

A21233260b_b
(-)-Epigallocatechin AREA:3.14e5
S/N:90.1

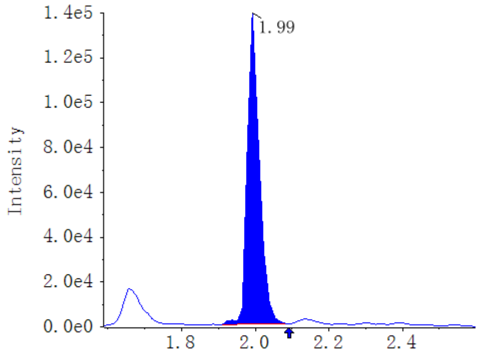

A21233261b_b
(-)-Epigallocatechin AREA:3.96e5
S/N:71.4

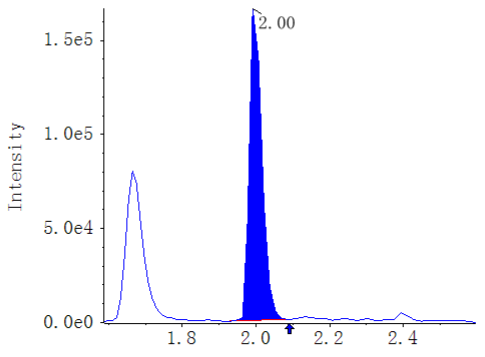

A21233262b_b
(-)-Epigallocatechin AREA:7.34e3
S/N:10.3

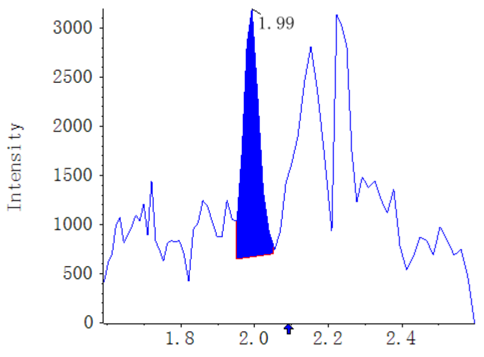

A21233263b_b
(-)-Epigallocatechin AREA:2.87e4
S/N:24.5

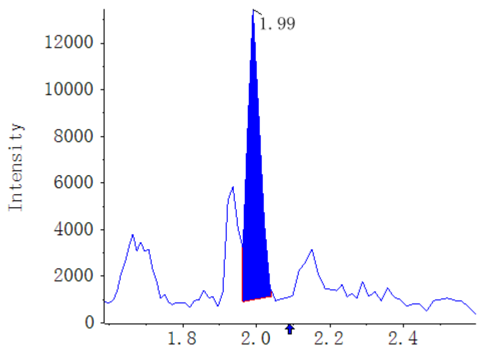

A21233264b_b
(-)-Epigallocatechin AREA:4.51e4
S/N:37.6

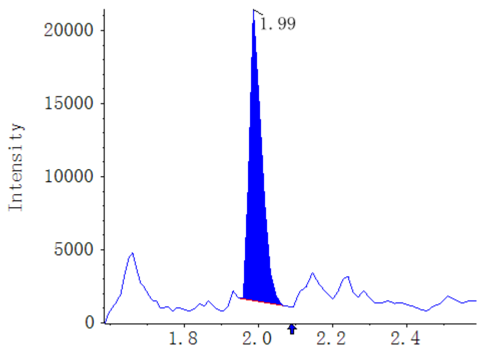

A21233265b_b
(-)-Epigallocatechin AREA:9.09e3
S/N:16.0

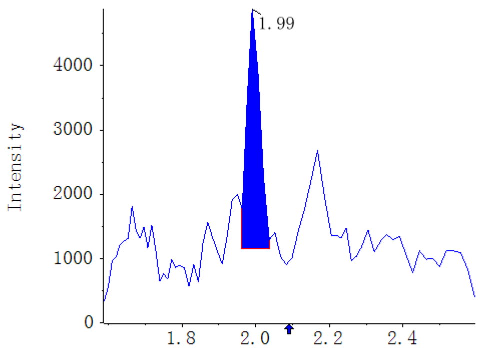

A21233266b_b
(-)-Epigallocatechin AREA:1.84e4
S/N:24.6

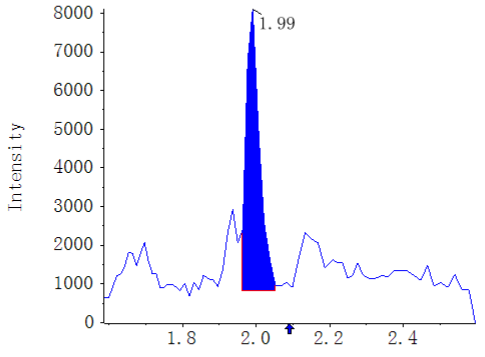

A21233267b_b
(-)-Epigallocatechin AREA:1.58e4
S/N:20.0

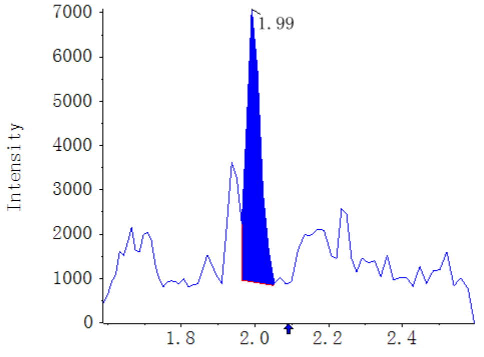

**Result Table**	MWXS-21-2101D-3_18_WH6500-5_A20-3_V1.0_TY_20211028	**Algorithm Used**	MQ4
**Acquisition Method**	Flavonoids_V1.0_WH6500-5_LT_20211025.dam	**Instrument Name**	QTRAP 6500+ Low Mass
**Project**	N/A	**Analytes QTY**	204:6

Compound name: Hydroxysafflor yellow A (611.2 / 491.1)

Sample Name	Sample Type	Area (cps)	Is Area (cps)	RT (min)	S/N	Target Conc	Calculated Conc.()
STD_0.5nM	Standard	4.79e2	N/A	2.03	13.9	0.5000	4.798493e-1
STD_1nM	Standard	6.38e2	N/A	2.02	21.9	1.0000	7.575171e-1
STD_5nM	Standard	4.40e3	N/A	2.02	90.1	5.0000	7.336975e0
STD_10nM	Standard	5.97e3	N/A	2.02	117.6	10.0000	1.007835e1
STD_20nM	Standard	9.94e3	N/A	2.02	117.9	20.0000	1.702306e1
STD_50nM	Standard	2.75e4	N/A	2.03	222.9	50.0000	4.774500e1
STD_100nM	Standard	5.58e4	N/A	2.03	214.4	100.0000	9.722099e1
STD_200nM	Standard	1.18e5	N/A	2.02	284.9	200.0000	2.058583e2
STD_500nM	Standard	N/A	N/A	N/A	N/A	500.0000	N/A
STD_1000nM	Standard	N/A	N/A	N/A	N/A	1000.0000	N/A
STD_2000nM	Standard	N/A	N/A	N/A	N/A	2000.0000	N/A
V1.0_MW_RQC1_20211018	Quality Control	1.14e5	N/A	2.08	3.4	0.0000	1.998614e2
Blank	Unknown	N/A	N/A	N/A	N/A	N/A	N/A
V1.0_MWMS_20211021_1	Unknown	9.66e4	N/A	2.02	294.1	N/A	1.685403e2
MWXS212101D3_R1	Quality Control	9.87e4	N/A	2.02	325.1	0.0000	1.723262e2
MWXS212101D3_R2	Quality Control	9.77e4	N/A	2.02	269.7	0.0000	1.704766e2
MWXS212101D3_R3	Quality Control	9.93e4	N/A	2.02	273.4	0.0000	1.734046e2
A21233250b_b	Unknown	5.86e3	N/A	2.02	9.4	N/A	9.895778e0
A21233251b_b	Unknown	2.81e3	N/A	2.02	4.6	N/A	4.549680e0
A21233252b_b	Unknown	1.05e3	N/A	2.02	3.3	N/A	1.485191e0
A21233253b_b	Unknown	1.04e4	N/A	2.02	9.4	N/A	1.781497e1
A21233254b_b	Unknown	4.12e3	N/A	2.01	7.6	N/A	6.842628e0
A21233255b_b	Unknown	5.37e3	N/A	2.02	6.1	N/A	9.035869e0
A21233256b_b	Unknown	4.04e3	N/A	2.02	5.6	N/A	6.701250e0
A21233257b_b	Unknown	5.24e3	N/A	2.02	5.1	N/A	8.806652e0
A21233258b_b	Unknown	2.75e3	N/A	2.02	3.3	N/A	4.460371e0
A21233259b_b	Unknown	1.47e3	N/A	2.02	2.9	N/A	2.210269e0
A21233260b_b	Unknown	3.83e3	N/A	2.02	3.7	N/A	6.344096e0
A21233261b_b	Unknown	4.36e3	N/A	2.02	4.2	N/A	7.266189e0
A21233262b_b	Unknown	8.68e3	N/A	2.02	10.7	N/A	1.483020e1
A21233263b_b	Unknown	7.91e3	N/A	2.01	7.0	N/A	1.347285e1
A21233264b_b	Unknown	7.51e3	N/A	2.02	6.0	N/A	1.278077e1
A21233265b_b	Unknown	1.25e4	N/A	2.02	6.1	N/A	2.153167e1
A21233266b_b	Unknown	7.22e3	N/A	2.01	3.8	N/A	1.227636e1
A21233267b_b	Unknown	4.85e3	N/A	2.02	3.8	N/A	8.129419e0

Compound name: Hydroxysafflor yellow A
Regression Equation: y = 571.68658 x + 204.97312 (r = 0.99736) (weighting: 1 / x)

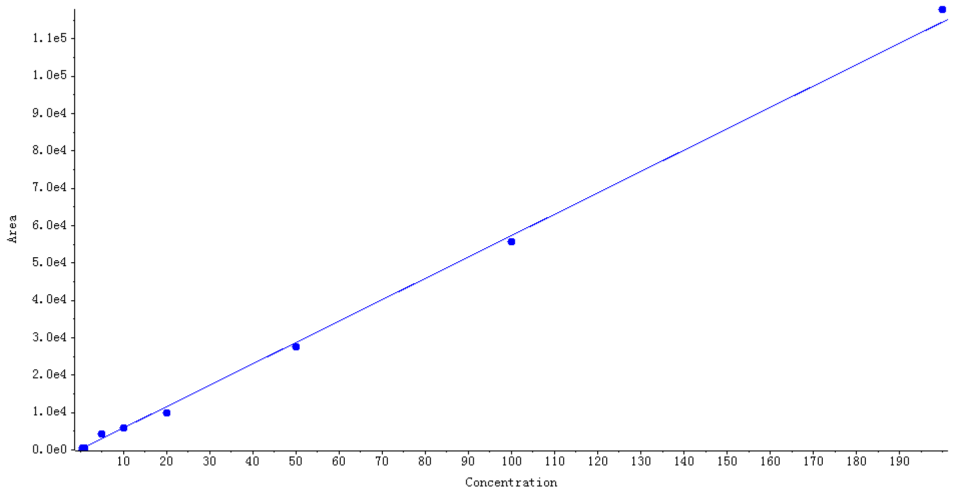

Peak Review

Blank
Hydroxysafflor yellow A   AREA:N/A
S/N:N/A

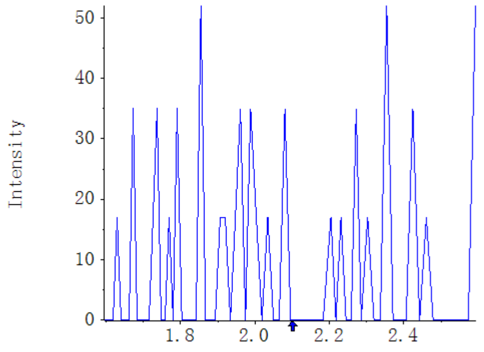

V1.0_MWMS_20211021_1
Hydroxysafflor yellow A
AREA:9.66e4   S/N:294.1

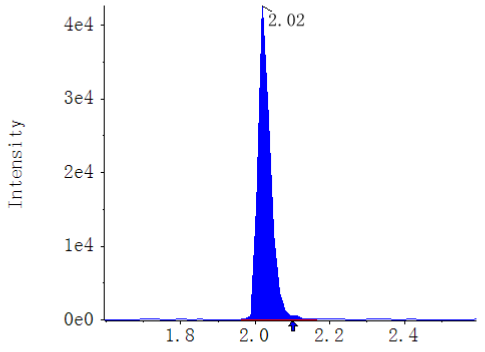

A21233250b_b
Hydroxysafflor yellow A
AREA:5.86e3   S/N:9.4

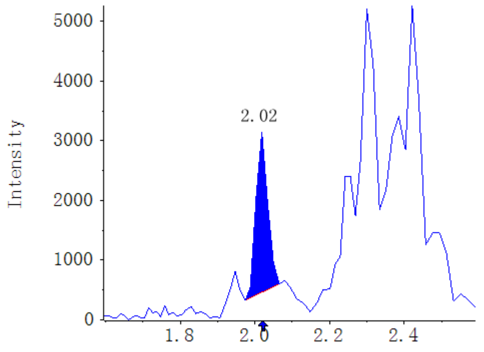

A21233251b_b
Hydroxysafflor yellow A
AREA:2.81e3   S/N:4.6

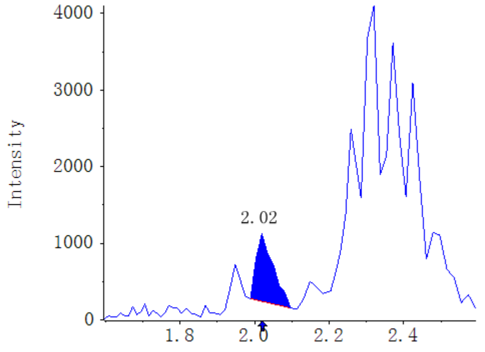

A21233252b_b
Hydroxysafflor yellow A
AREA:1.05e3   S/N:3.3

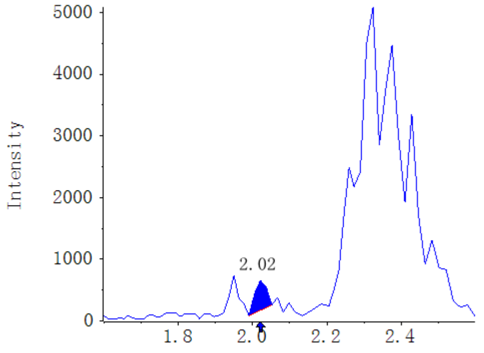

A21233253b_b
Hydroxysafflor yellow A
AREA:1.04e4   S/N:9.4

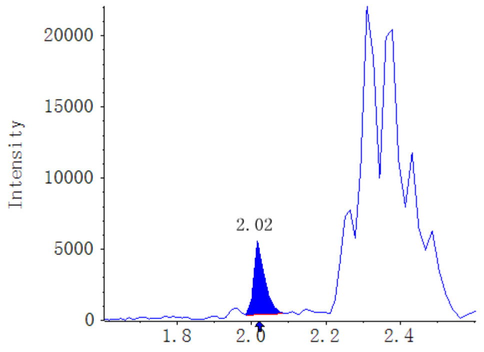

A21233254b_b
Hydroxysafflor yellow A
AREA:4.12e3   S/N:7.6

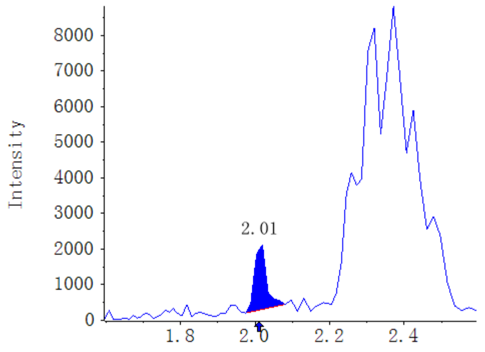

A21233255b_b
Hydroxysafflor yellow A
AREA:5.37e3   S/N:6.1

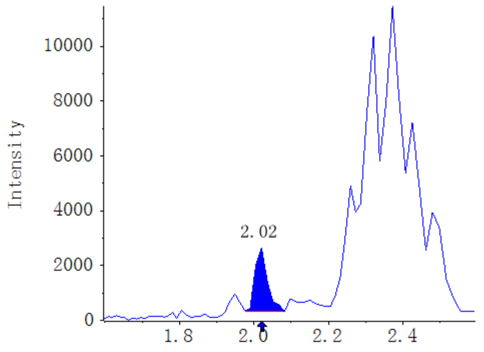

A21233256b_b
Hydroxysafflor yellow A
AREA:4.04e3   S/N:5.6

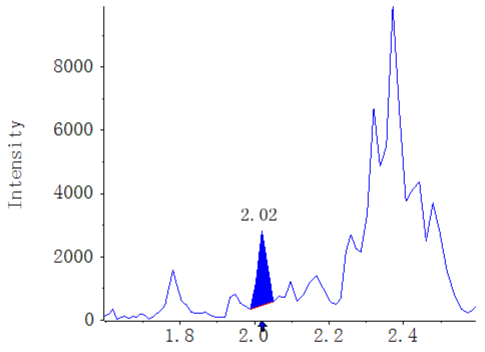

A21233257b_b
Hydroxysafflor yellow A
AREA:5.24e3   S/N:5.1

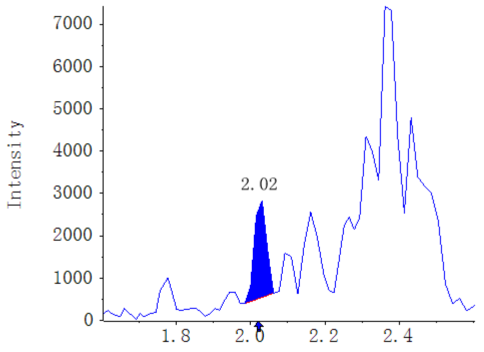

A21233258b_b
Hydroxysafflor yellow A
AREA:2.75e3 S/N:3.3

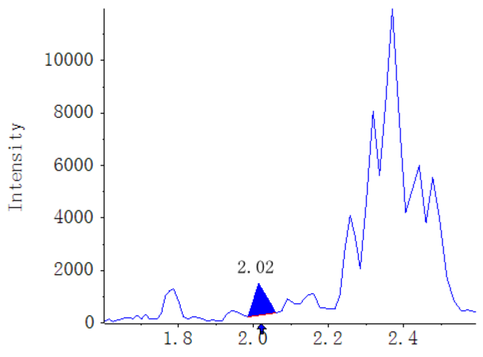

A21233259b_b
Hydroxysafflor yellow A
AREA:1.47e3 S/N:2.9

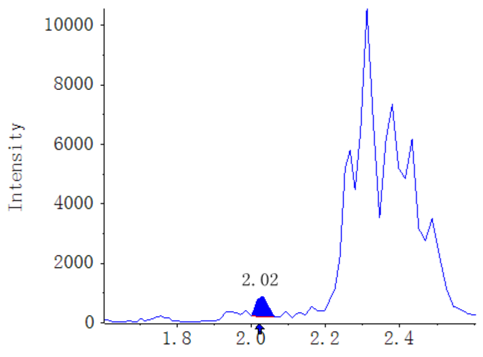

A21233260b_b
Hydroxysafflor yellow A
AREA:3.83e3 S/N:3.7

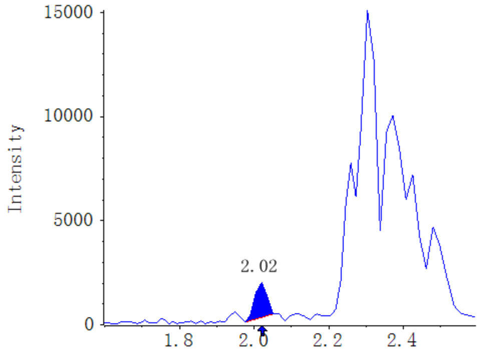

A21233261b_b
Hydroxysafflor yellow A
AREA:4.36e3 S/N:4.2

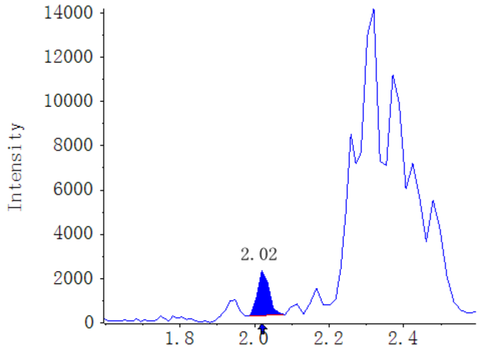

A21233262b_b
Hydroxysafflor yellow A
AREA:8.68e3 S/N:10.7

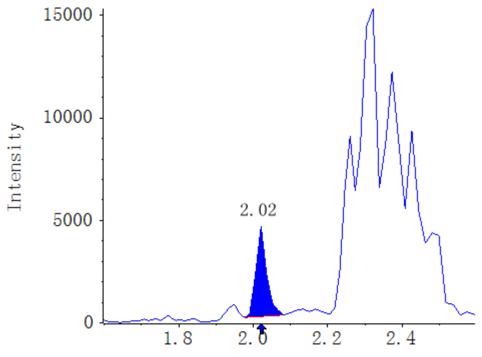

A21233263b_b
Hydroxysafflor yellow A
AREA:7.91e3 S/N:7.0

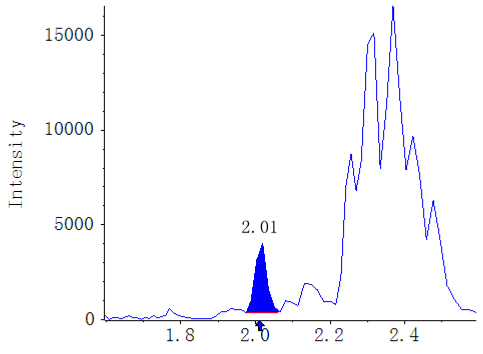

A21233264b_b
Hydroxysafflor yellow A
AREA:7.51e3 S/N:6.0

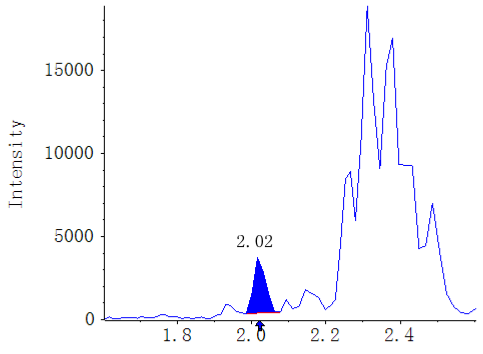

A21233265b_b
Hydroxysafflor yellow A
AREA:1.25e4 S/N:6.1

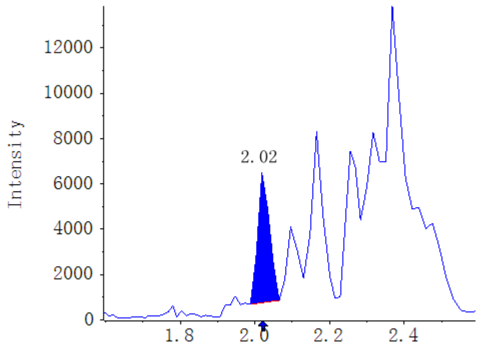

A21233266b_b
Hydroxysafflor yellow A
AREA:7.22e3 S/N:3.8

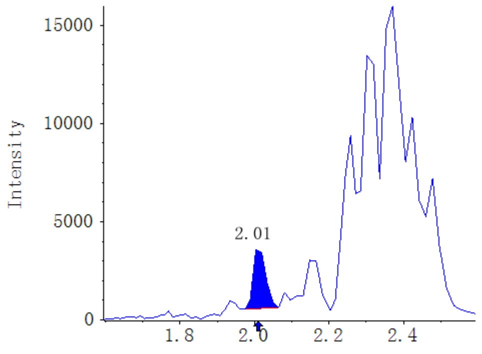

A21233267b_b
Hydroxysafflor yellow A
AREA:4.85e3 S/N:3.8

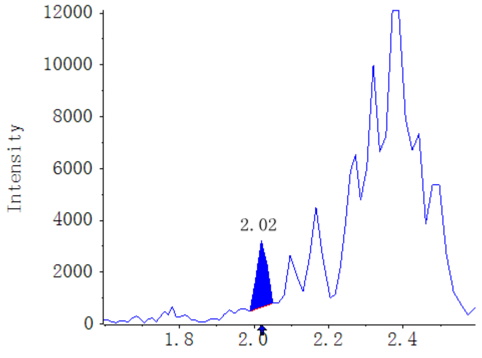

Result Table	MWXS-21-2101D-3_18_WH6500-5_A20-3_V1.0_TY_20211028	Algorithm Used	MQ4
Acquisition Method	Flavonoids_V1.0_WH6500-5_LT_20211025.dam	Instrument Name	QTRAP 6500+ Low Mass
Project	N/A	Analytes QTY	204:20

Compound name: Isoliquiritigenin (255.1 / 119.1)

Sample Name	Sample Type	Area (cps)	Is Area (cps)	RT (min)	S/N	Target Conc	Calculated Conc.()
STD_0.5nM	Standard	1.72e4	N/A	5.58	30.0	0.5000	3.341491e-1
STD_1nM	Standard	3.35e4	N/A	5.57	50.0	1.0000	8.348696e-1
STD_5nM	Standard	2.34e5	N/A	5.57	179.1	5.0000	7.005695e0
STD_10nM	Standard	3.44e5	N/A	5.57	182.6	10.0000	1.036083e1
STD_20nM	Standard	6.62e5	N/A	5.57	259.0	20.0000	2.013858e1
STD_50nM	Standard	1.76e6	N/A	5.58	236.5	50.0000	5.397148e1
STD_100nM	Standard	3.29e6	N/A	5.58	240.1	100.0000	1.007959e2
STD_200nM	Standard	6.29e6	N/A	5.57	235.0	200.0000	1.930585e2
STD_500nM	Standard	N/A	N/A	N/A	N/A	500.0000	N/A
STD_1000nM	Standard	N/A	N/A	N/A	N/A	1000.0000	N/A
STD_2000nM	Standard	N/A	N/A	N/A	N/A	2000.0000	N/A
V1.0_MW_RQC1_20211018	Quality Control	N/A	N/A	N/A	N/A	0.0000	N/A
Blank	Unknown	N/A	N/A	N/A	N/A	N/A	N/A
V1.0_MWMS_20211021_1	Unknown	4.68e6	N/A	5.59	246.1	N/A	1.435313e2
MWXS212101D3_R1	Quality Control	4.92e6	N/A	5.58	271.8	0.0000	1.508561e2
MWXS212101D3_R2	Quality Control	4.80e6	N/A	5.58	247.0	0.0000	1.473081e2
MWXS212101D3_R3	Quality Control	4.70e6	N/A	5.58	256.8	0.0000	1.442793e2
A21233250b_b	Unknown	N/A	N/A	N/A	N/A	N/A	N/A
A21233251b_b	Unknown	N/A	N/A	N/A	N/A	N/A	N/A
A21233252b_b	Unknown	N/A	N/A	N/A	N/A	N/A	N/A
A21233253b_b	Unknown	2.36e4	N/A	5.59	44.5	N/A	5.312470e-1
A21233254b_b	Unknown	4.12e4	N/A	5.58	70.1	N/A	1.070064e0
A21233255b_b	Unknown	2.91e4	N/A	5.58	61.0	N/A	6.981687e-1
A21233256b_b	Unknown	9.04e3	N/A	5.58	28.7	N/A	8.341050e-2
A21233257b_b	Unknown	9.59e3	N/A	5.59	23.4	N/A	1.003203e-1
A21233258b_b	Unknown	1.15e4	N/A	5.58	31.1	N/A	1.584105e-1
A21233259b_b	Unknown	N/A	N/A	N/A	N/A	N/A	N/A
A21233260b_b	Unknown	N/A	N/A	N/A	N/A	N/A	N/A
A21233261b_b	Unknown	N/A	N/A	N/A	N/A	N/A	N/A
A21233262b_b	Unknown	N/A	N/A	N/A	N/A	N/A	N/A
A21233263b_b	Unknown	3.56e4	N/A	5.58	77.9	N/A	8.988195e-1
A21233264b_b	Unknown	6.33e4	N/A	5.59	97.0	N/A	1.750796e0
A21233265b_b	Unknown	2.15e4	N/A	5.58	44.8	N/A	4.667258e-1
A21233266b_b	Unknown	2.91e4	N/A	5.58	59.3	N/A	7.005021e-1
A21233267b_b	Unknown	1.80e4	N/A	5.59	46.3	N/A	3.589496e-1

Compound name: Isoliquiritigenin
Regression Equation: y = 3.25665e4 x + 6324.48386 (r = 0.99802) (weighting: 1 / x)

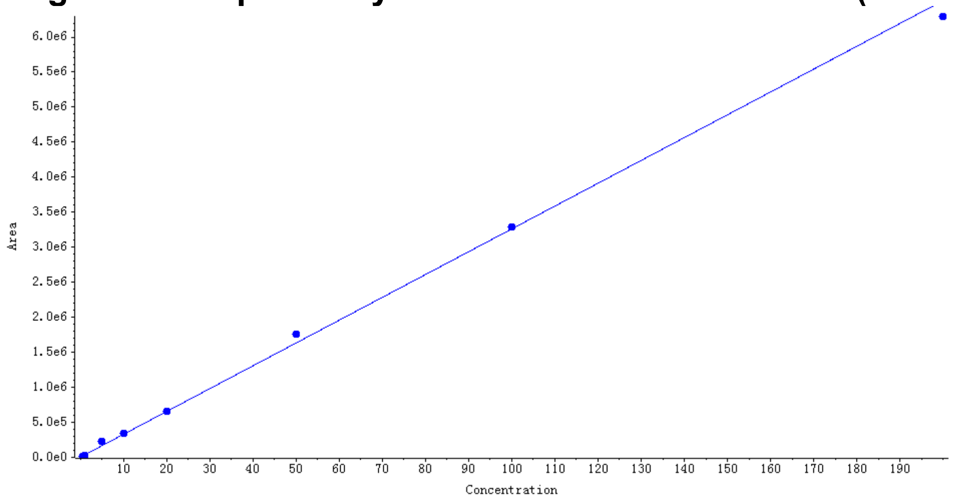

Peak Review

Blank
Isoliquiritigenin AREA:N/A
S/N:N/A

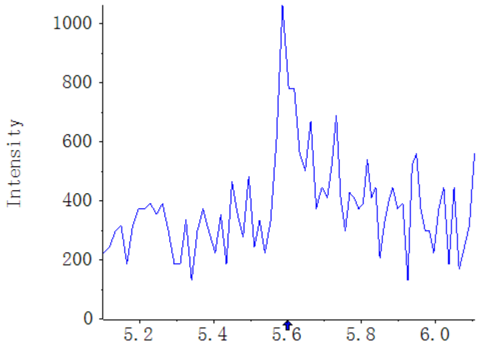

V1.0_MWMS_20211021_1
Isoliquiritigenin AREA:4.68e6
S/N:246.1

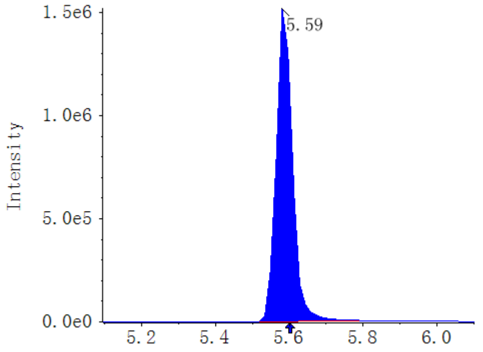

A21233250b_b
Isoliquiritigenin AREA:N/A
S/N:N/A

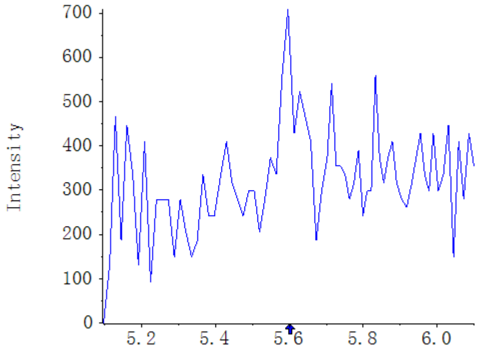

A21233251b_b
Isoliquiritigenin AREA:N/A
S/N:N/A

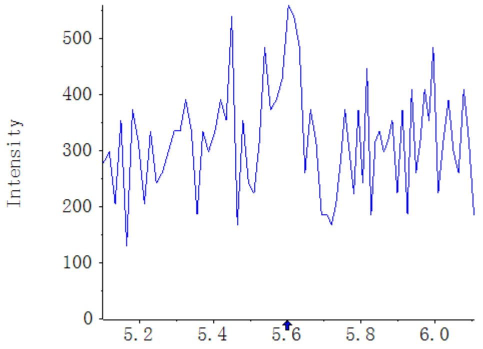

A21233252b_b
Isoliquiritigenin AREA:N/A
S/N:N/A

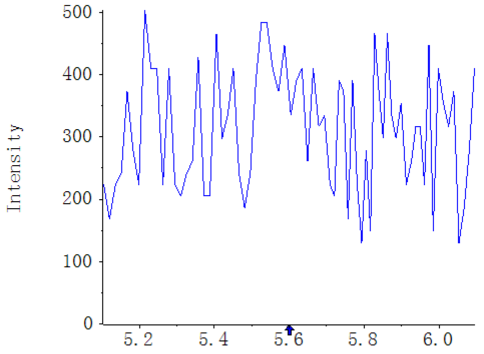

A21233253b_b
Isoliquiritigenin AREA:2.36e4
S/N:44.5

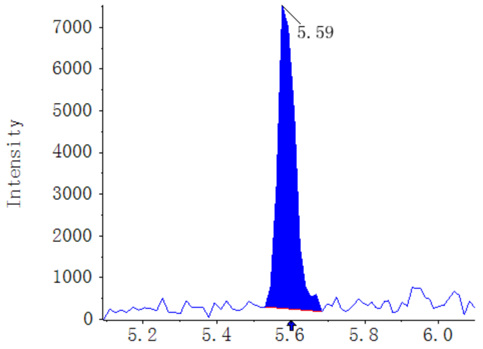

A21233254b_b
Isoliquiritigenin AREA:4.12e4
S/N:70.1

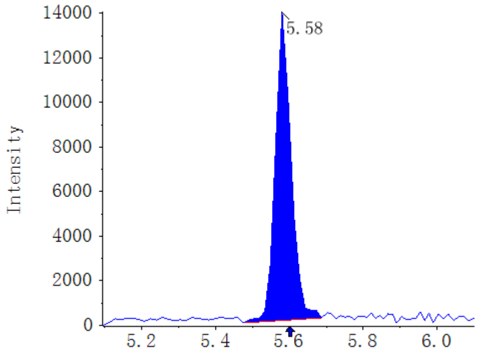

A21233255b_b
Isoliquiritigenin AREA:2.91e4
S/N:61.0

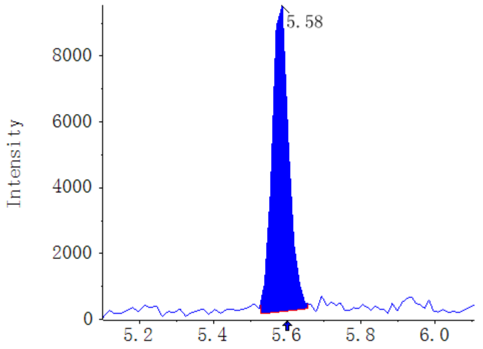

A21233256b_b
Isoliquiritigenin AREA:9.04e3
S/N:28.7

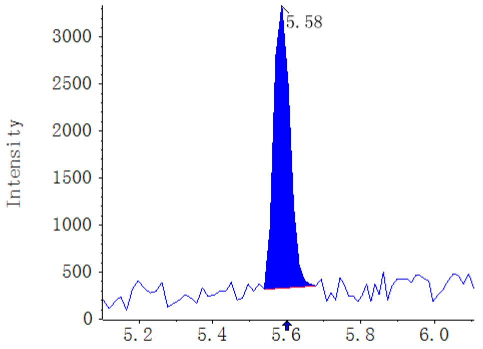

A21233257b_b
Isoliquiritigenin AREA:9.59e3
S/N:23.4

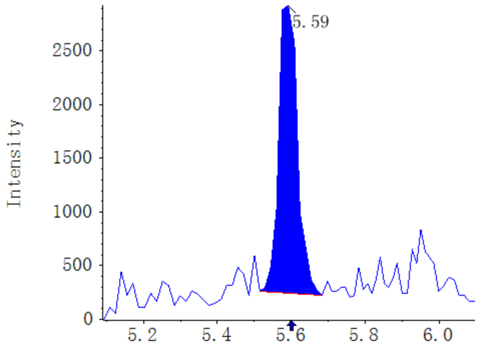

A21233258b_b
Isoliquiritigenin AREA:1.15e4
S/N:31.1

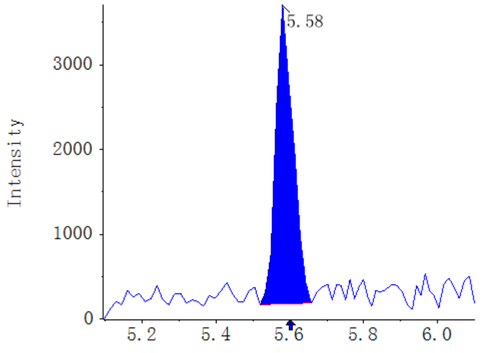

A21233259b_b
Isoliquiritigenin AREA:N/A
S/N:N/A

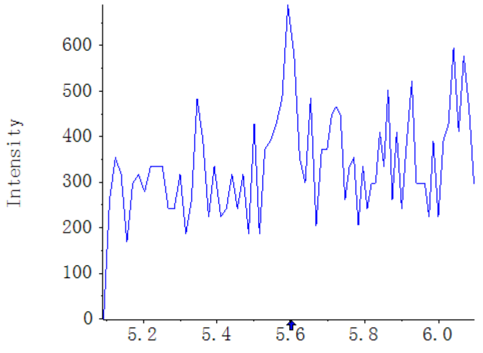

A21233260b_b
Isoliquiritigenin AREA:N/A
S/N:N/A

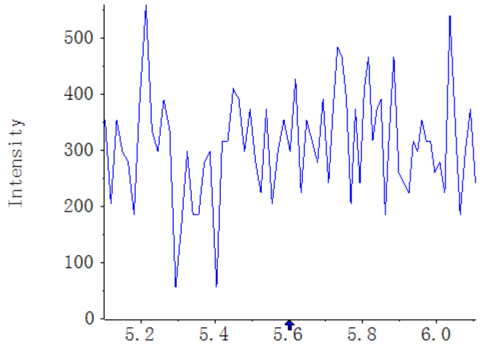

A21233261b_b
Isoliquiritigenin AREA:N/A
S/N:N/A

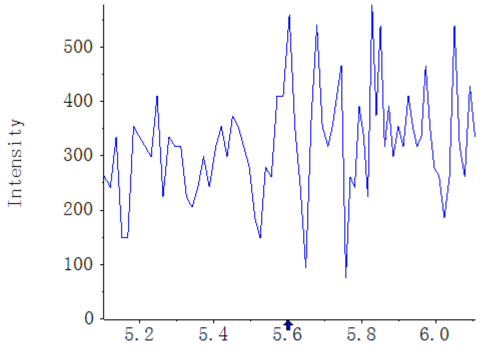

A21233262b_b
Isoliquiritigenin AREA:N/A
S/N:N/A

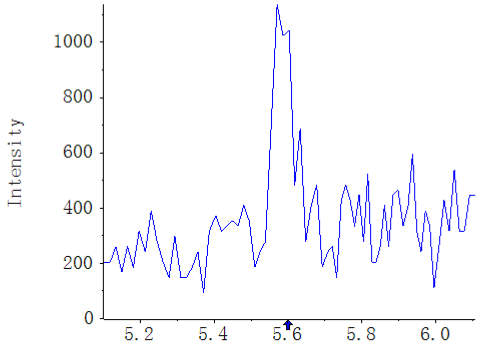

A21233263b_b
Isoliquiritigenin AREA:3.56e4
S/N:77.9

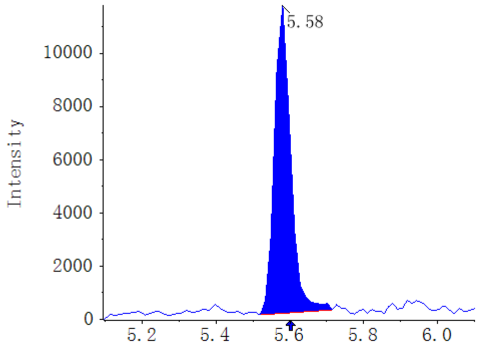

A21233264b_b
Isoliquiritigenin AREA:6.33e4
S/N:97.0

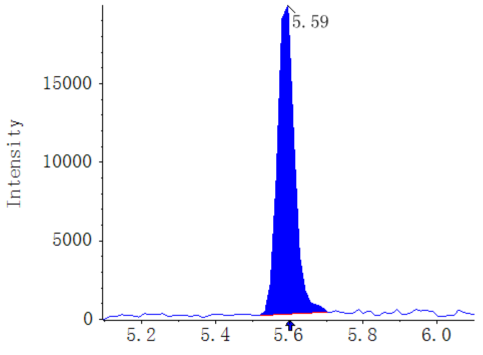

A21233265b_b
Isoliquiritigenin AREA:2.15e4
S/N:44.8

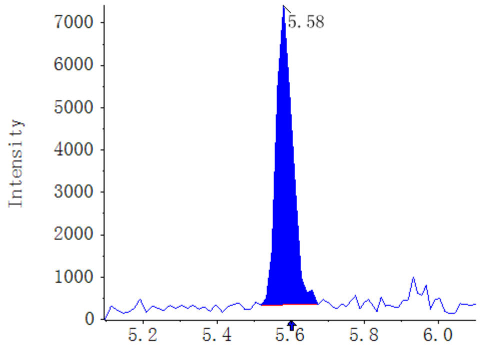

A21233266b_b
Isoliquiritigenin AREA:2.91e4
S/N:59.3

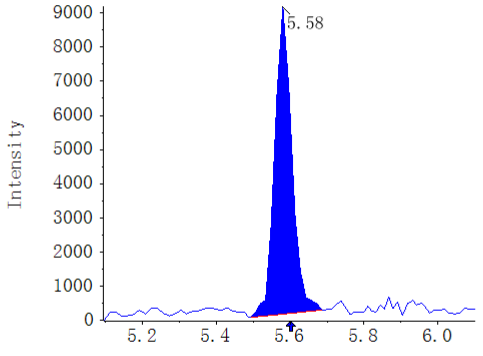

A21233267b_b
Isoliquiritigenin AREA:1.80e4
S/N:46.3

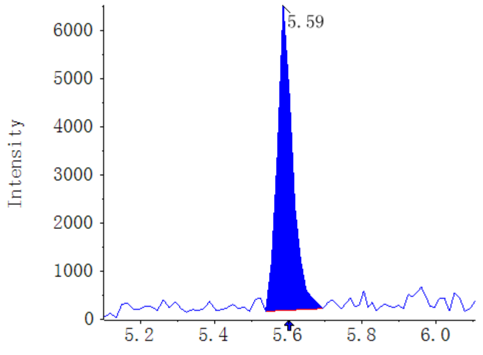

**Result Table**	MWXS-21-2101D-3_18_WH6500-5_A20-3_V1.0_TY_20211028	**Algorithm Used**	MQ4
**Acquisition Method**	Flavonoids_V1.0_WH6500-5_LT_20211025.dam	**Instrument Name**	QTRAP 6500+ Low Mass
**Project**	N/A	**Analytes QTY**	204:43

Compound name: (-)-Catechin (289.1 / 123.0)

Sample Name	Sample Type	Area (cps)	Is Area (cps)	RT (min)	S/N	Target Conc	Calculated Conc.()
STD_0.5nM	Standard	N/A	N/A	N/A	N/A	0.5000	N/A
STD_1nM	Standard	1.24e3	N/A	2.19	4.1	1.0000	6.110194e-1
STD_5nM	Standard	6.16e3	N/A	2.19	15.2	5.0000	6.342053e0
STD_10nM	Standard	1.02e4	N/A	2.19	20.9	10.0000	1.102913e1
STD_20nM	Standard	1.84e4	N/A	2.18	26.2	20.0000	2.062977e1
STD_50nM	Standard	4.55e4	N/A	2.19	48.5	50.0000	5.223146e1
STD_100nM	Standard	8.06e4	N/A	2.19	43.4	100.0000	9.315127e1
STD_200nM	Standard	1.74e5	N/A	2.18	68.8	200.0000	2.020053e2
STD_500nM	Standard	N/A	N/A	N/A	N/A	500.0000	N/A
STD_1000nM	Standard	N/A	N/A	N/A	N/A	1000.0000	N/A
STD_2000nM	Standard	N/A	N/A	N/A	N/A	2000.0000	N/A
V1.0_MW_RQC1_20211018	Quality Control	N/A	N/A	N/A	N/A	0.0000	N/A
Blank	Unknown	N/A	N/A	N/A	N/A	N/A	N/A
V1.0_MWMS_20211021_1	Unknown	1.31e5	N/A	2.19	49.5	N/A	1.521366e2
MWXS212101D3_R1	Quality Control	1.42e5	N/A	2.19	45.2	0.0000	1.643192e2
MWXS212101D3_R2	Quality Control	1.39e5	N/A	2.19	53.1	0.0000	1.614741e2
MWXS212101D3_R3	Quality Control	1.40e5	N/A	2.19	53.2	0.0000	1.625433e2
A21233250b_b	Unknown	1.30e4	N/A	2.18	13.7	N/A	1.433322e1
A21233251b_b	Unknown	1.04e4	N/A	2.19	14.0	N/A	1.124795e1
A21233252b_b	Unknown	4.95e3	N/A	2.20	5.3	N/A	4.931176e0
A21233253b_b	Unknown	8.79e3	N/A	2.19	8.7	N/A	9.412235e0
A21233254b_b	Unknown	5.98e3	N/A	2.18	8.2	N/A	6.131496e0
A21233255b_b	Unknown	7.77e3	N/A	2.19	8.2	N/A	8.220808e0
A21233256b_b	Unknown	6.30e3	N/A	2.20	4.8	N/A	6.509852e0
A21233257b_b	Unknown	6.19e3	N/A	2.20	5.4	N/A	6.379968e0
A21233258b_b	Unknown	6.29e3	N/A	2.19	5.1	N/A	6.494431e0
A21233259b_b	Unknown	9.94e3	N/A	2.19	6.2	N/A	1.075006e1
A21233260b_b	Unknown	1.33e4	N/A	2.18	10.6	N/A	1.466479e1
A21233261b_b	Unknown	9.55e4	N/A	2.19	28.7	N/A	1.105059e2
A21233262b_b	Unknown	1.47e4	N/A	2.19	10.0	N/A	1.633178e1
A21233263b_b	Unknown	1.55e4	N/A	2.19	14.6	N/A	1.724462e1
A21233264b_b	Unknown	2.65e4	N/A	2.20	13.6	N/A	3.007579e1
A21233265b_b	Unknown	9.21e3	N/A	2.20	6.2	N/A	9.901830e0
A21233266b_b	Unknown	1.45e4	N/A	2.18	14.2	N/A	1.606641e1
A21233267b_b	Unknown	1.90e4	N/A	2.20	12.0	N/A	2.126302e1

Compound name: (-)-Catechin
Regression Equation: y = 858.06877 x + 714.83349 (r = 0.99826) (weighting: 1 / x)

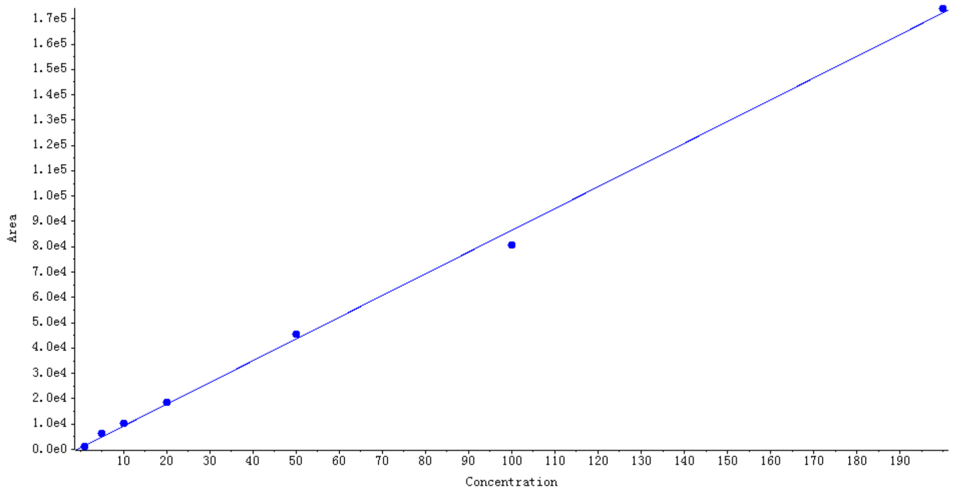

Peak Review

Blank
(-)-Catechin AREA:N/A S/N:N/A

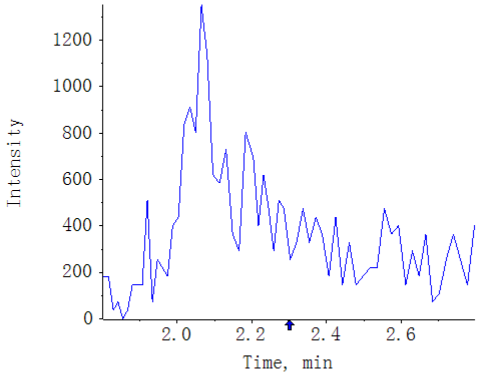

V1.0_MWMS_20211021_1
(-)-Catechin AREA:1.31e5 S/N:49.5

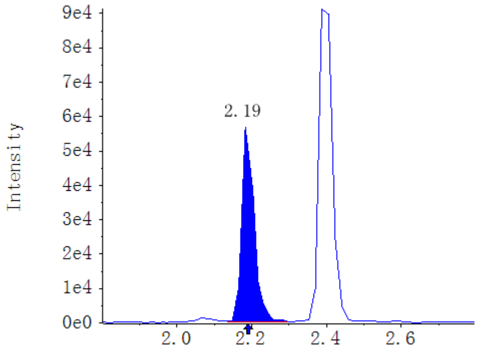

A21233250b_b
(-)-Catechin AREA:1.30e4 S/N:13.7

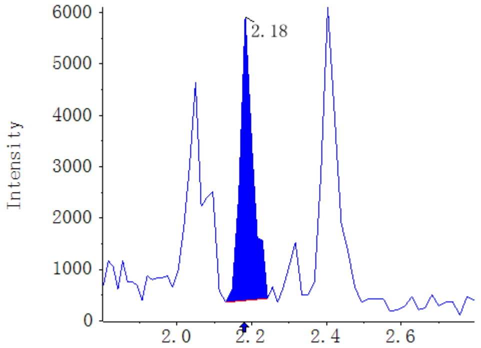

A21233251b_b
(-)-Catechin AREA:1.04e4 S/N:14.0

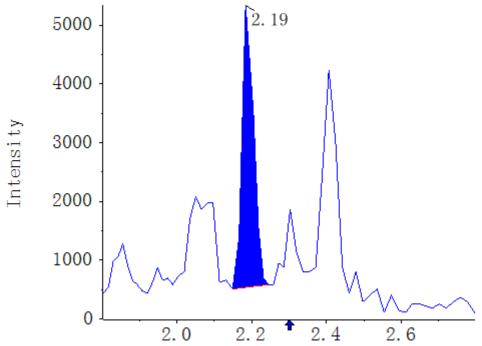

A21233252b_b
(-)-Catechin AREA:4.95e3 S/N:5.3

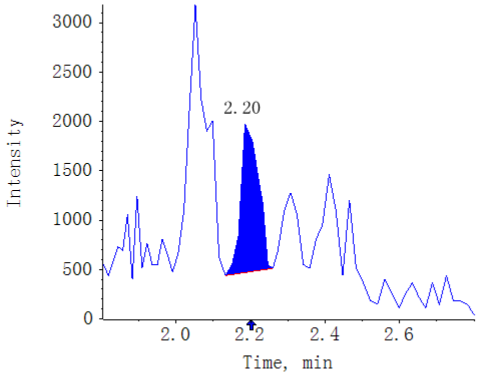

A21233253b_b
(-)-Catechin AREA:8.79e3 S/N:8.7

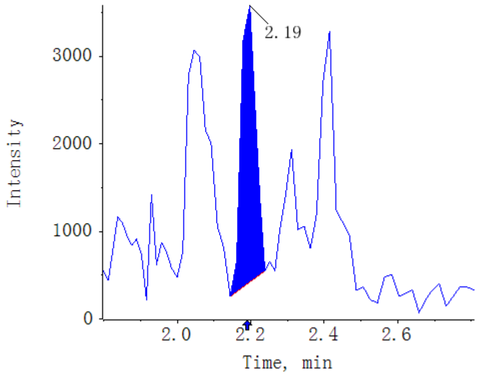

A21233254b_b
(-)-Catechin AREA:5.98e3 S/N:8.2

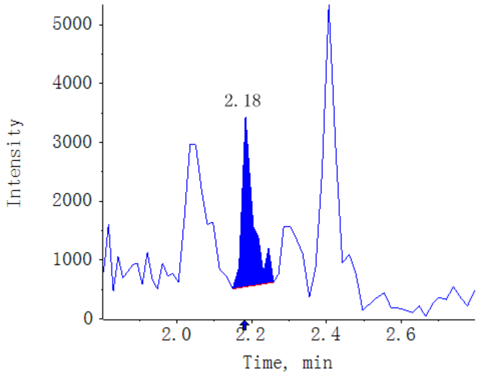

A21233255b_b
(-)-Catechin AREA:7.77e3 S/N:8.2

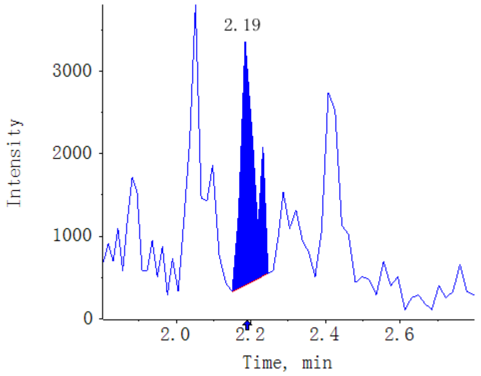

A21233256b_b
(-)-Catechin AREA:6.30e3 S/N:4.8

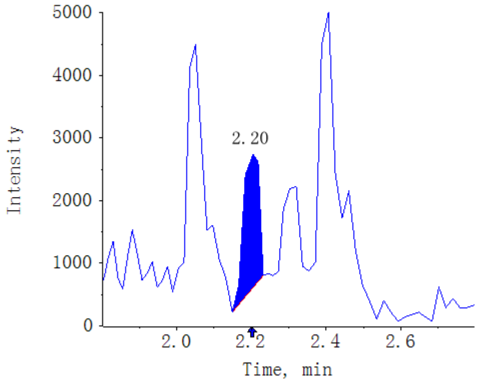

A21233257b_b
(-)-Catechin AREA:6.19e3 S/N:5.4

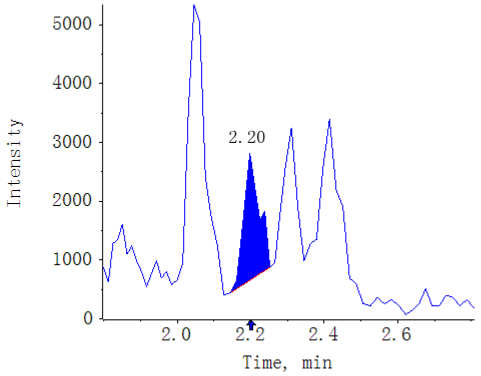

A21233258b_b
(-)-Catechin AREA:6.29e3 S/N:5.1

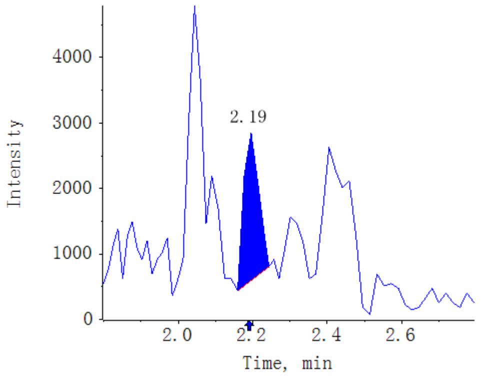

A21233259b_b
(-)-Catechin AREA:9.94e3 S/N:6.2

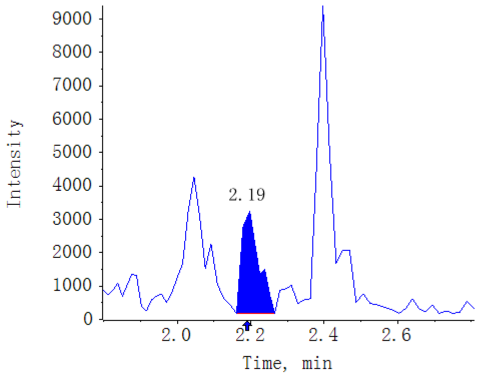

A21233260b_b
(-)-Catechin AREA:1.33e4
S/N:10.6

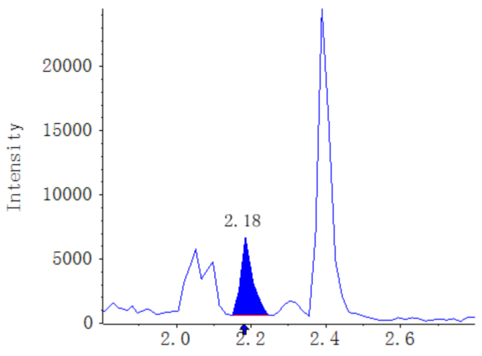

A21233261b_b
(-)-Catechin AREA:9.55e4
S/N:28.7

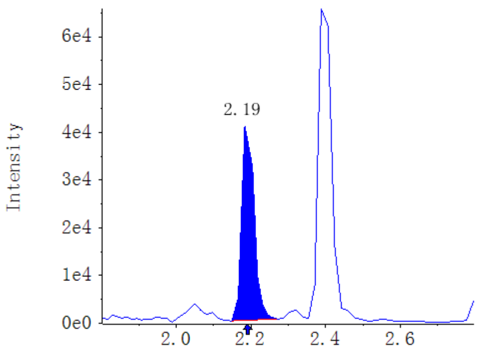

A21233262b_b
(-)-Catechin AREA:1.47e4
S/N:10.0

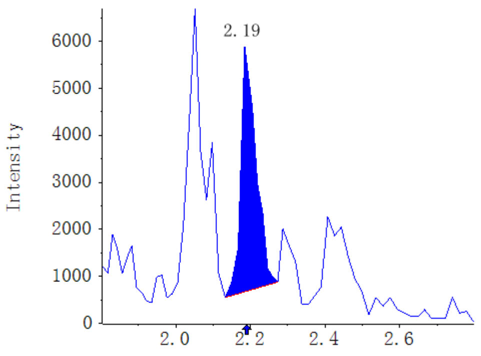

A21233263b_b
(-)-Catechin AREA:1.55e4
S/N:14.6

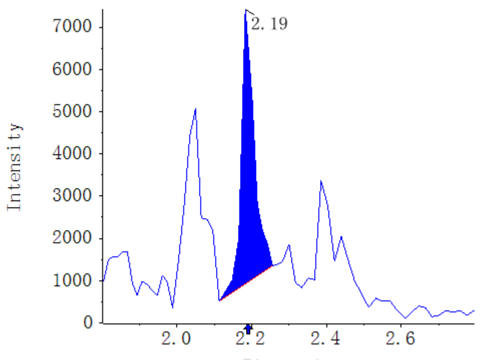

A21233264b_b
(-)-Catechin AREA:2.65e4
S/N:13.6

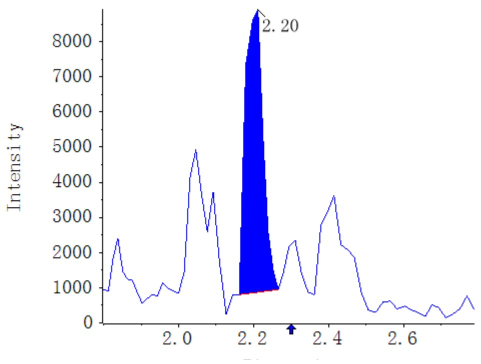

A21233265b_b
(-)-Catechin AREA:9.21e3 S/N:6.2

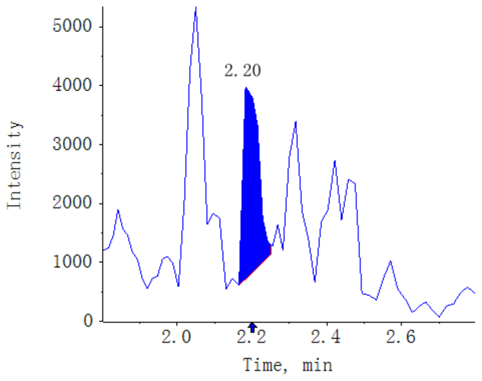

A21233266b_b
(-)-Catechin AREA:1.45e4
S/N:14.2

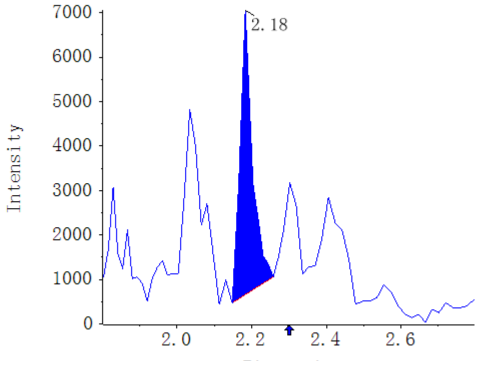

A21233267b_b
(-)-Catechin AREA:1.90e4
S/N:12.0

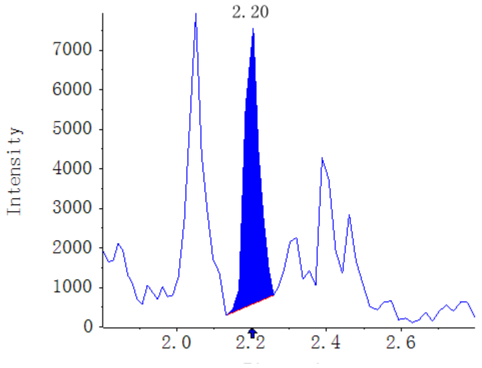

Result Table	MWXS-21-2101D-3_18_WH6500-5_A20-3_V1.0_TY_20211028	Algorithm Used	MQ4
Acquisition Method	Flavonoids_V1.0_WH6500-5_LT_20211025.dam	Instrument Name	QTRAP 6500+ Low Mass
Project	N/A	Analytes QTY	204:36

Compound name: Narirutin (579.2 / 271.1)

Sample Name	Sample Type	Area (cps)	Is Area (cps)	RT (min)	S/N	Target Conc	Calculated Conc.()
STD_0.5nM	Standard	6.24e3	N/A	2.89	32.7	0.5000	3.996285e-1
STD_1nM	Standard	9.82e3	N/A	2.88	43.5	1.0000	7.872362e-1
STD_5nM	Standard	6.92e4	N/A	2.89	143.6	5.0000	7.208858e0
STD_10nM	Standard	9.57e4	N/A	2.89	137.7	10.0000	1.007389e1
STD_20nM	Standard	1.78e5	N/A	2.89	94.0	20.0000	1.898543e1
STD_50nM	Standard	4.76e5	N/A	2.89	305.3	50.0000	5.122150e1
STD_100nM	Standard	9.30e5	N/A	2.90	338.0	100.0000	1.003056e2
STD_200nM	Standard	1.83e6	N/A	2.88	398.9	200.0000	1.975179e2
STD_500nM	Standard	N/A	N/A	N/A	N/A	500.0000	N/A
STD_1000nM	Standard	N/A	N/A	N/A	N/A	1000.0000	N/A
STD_2000nM	Standard	N/A	N/A	N/A	N/A	2000.0000	N/A
V1.0_MW_RQC1_20211018	Quality Control	2.67e4	N/A	2.88	37.4	0.0000	2.615789e0
Blank	Unknown	N/A	N/A	N/A	N/A	N/A	N/A
V1.0_MWMS_20211021_1	Unknown	1.57e6	N/A	2.90	365.7	N/A	1.691182e2
MWXS212101D3_R1	Quality Control	1.60e6	N/A	2.90	367.2	0.0000	1.724673e2
MWXS212101D3_R2	Quality Control	1.56e6	N/A	2.90	406.1	0.0000	1.689219e2
MWXS212101D3_R3	Quality Control	1.58e6	N/A	2.90	350.9	0.0000	1.709201e2
A21233250b_b	Unknown	2.54e3	N/A	2.90	3.0	N/A	4.664084e-5
A21233251b_b	Unknown	N/A	N/A	N/A	N/A	N/A	N/A
A21233252b_b	Unknown	N/A	N/A	N/A	N/A	N/A	N/A
A21233253b_b	Unknown	3.32e4	N/A	2.93	7.9	N/A	3.313381e0
A21233254b_b	Unknown	1.97e4	N/A	2.93	7.0	N/A	1.851450e0
A21233255b_b	Unknown	3.49e4	N/A	2.94	8.5	N/A	3.499138e0
A21233256b_b	Unknown	7.77e4	N/A	2.94	20.8	N/A	8.124785e0
A21233257b_b	Unknown	8.62e4	N/A	2.94	13.3	N/A	9.041739e0
A21233258b_b	Unknown	2.27e4	N/A	2.94	9.5	N/A	2.181428e0
A21233259b_b	Unknown	N/A	N/A	N/A	N/A	N/A	N/A
A21233260b_b	Unknown	N/A	N/A	N/A	N/A	N/A	N/A
A21233261b_b	Unknown	N/A	N/A	N/A	N/A	N/A	N/A
A21233262b_b	Unknown	N/A	N/A	N/A	N/A	N/A	N/A
A21233263b_b	Unknown	4.90e3	N/A	2.94	2.1	N/A	2.552990e-1
A21233264b_b	Unknown	N/A	N/A	N/A	N/A	N/A	N/A
A21233265b_b	Unknown	2.85e4	N/A	2.93	4.6	N/A	2.806609e0
A21233266b_b	Unknown	1.68e4	N/A	2.93	3.4	N/A	1.546727e0
A21233267b_b	Unknown	9.09e3	N/A	2.94	2.4	N/A	7.082204e-1

Compound name: Narirutin
Regression Equation: y = 9247.89141 x + 2541.55417 (r = 0.99843) (weighting: 1 / x)

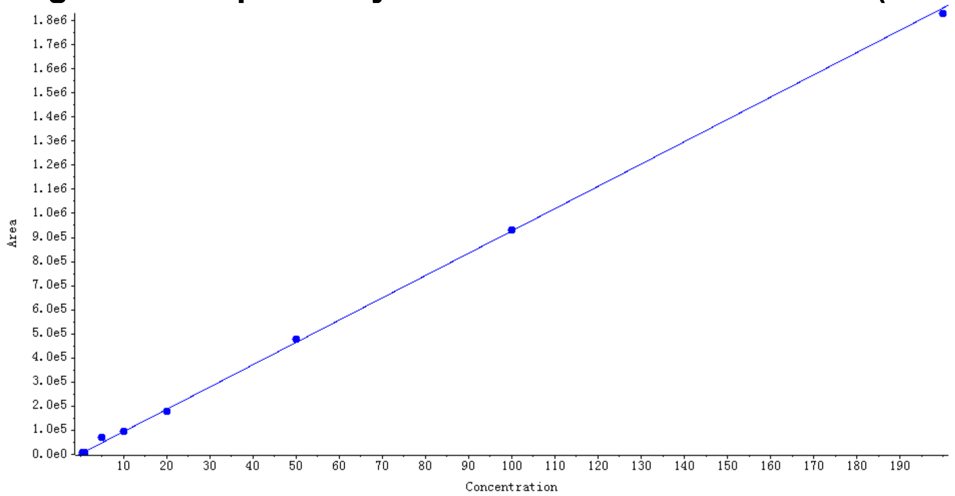

Peak Review

Blank
Narirutin AREA:N/A S/N:N/A

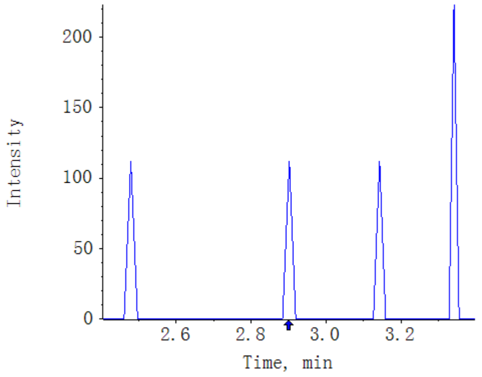

V1.0_MWMS_20211021_1
Narirutin AREA:1.57e6 S/N:365.7

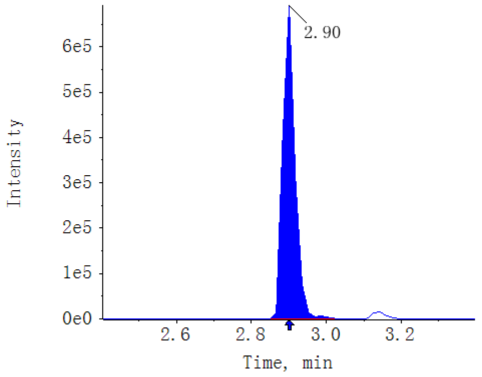

A21233250b_b
Narirutin AREA:2.54e3 S/N:3.0

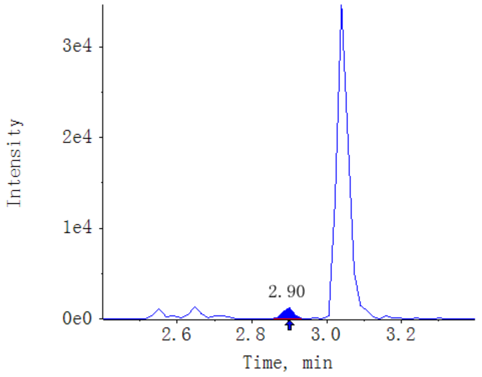

A21233251b_b
Narirutin AREA:N/A S/N:N/A

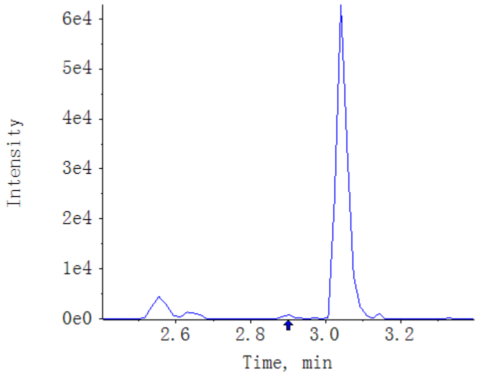

A21233252b_b
Narirutin AREA:N/A S/N:N/A

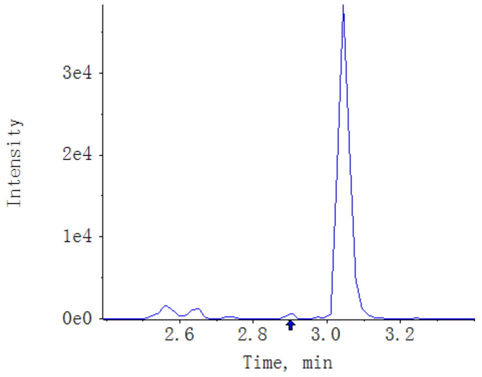

A21233253b_b
Narirutin AREA:3.32e4 S/N:7.9

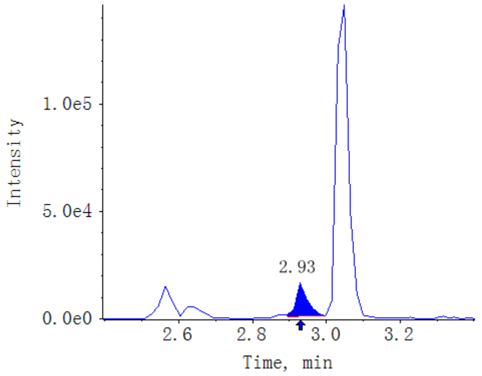

A21233254b_b
Narirutin AREA:1.97e4 S/N:7.0

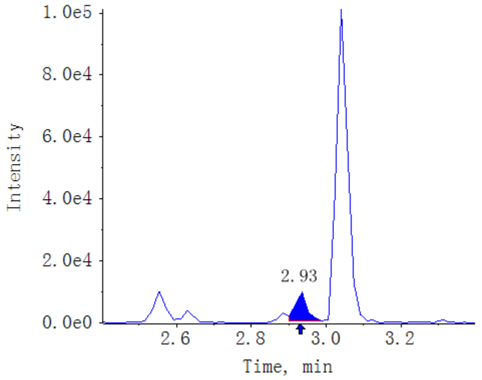

A21233255b_b
Narirutin AREA:3.49e4 S/N:8.5

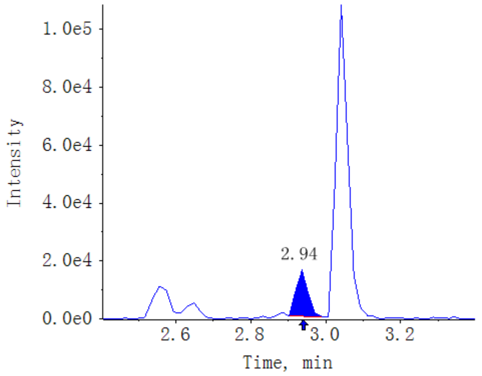

A21233256b_b
Narirutin AREA:7.77e4 S/N:20.8

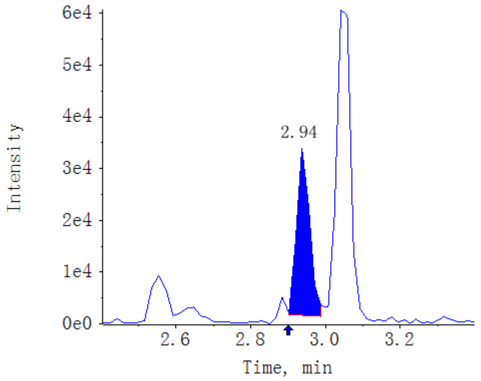

A21233257b_b
Narirutin AREA:8.62e4 S/N:13.3

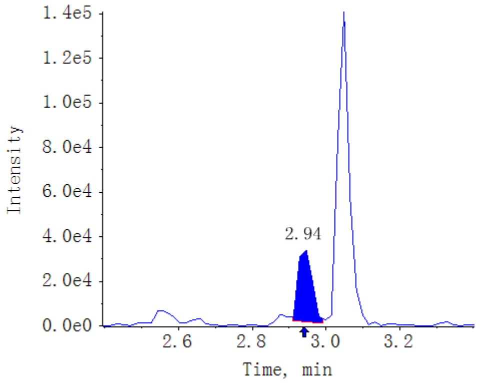

A21233258b_b
Narirutin AREA:2.27e4 S/N:9.5

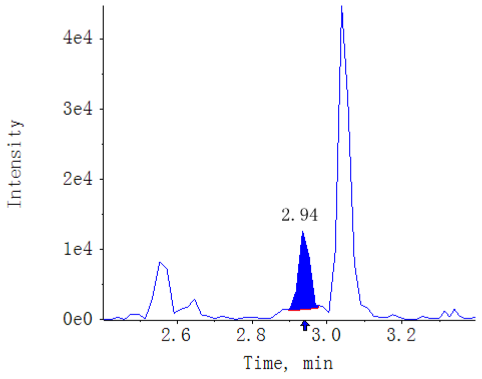

A21233259b_b
Narirutin AREA:N/A S/N:N/A

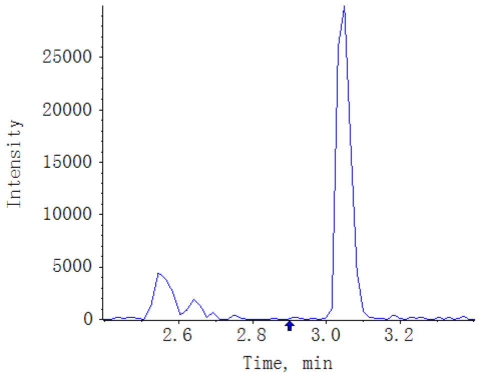

A21233260b_b
Narirutin AREA:N/A S/N:N/A

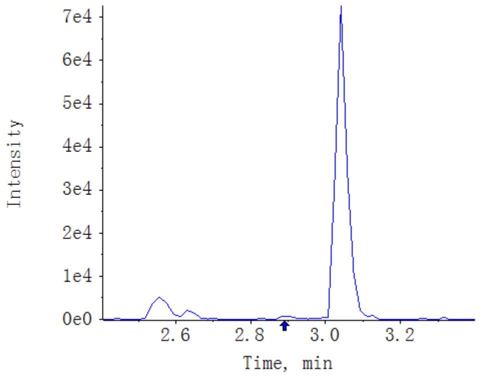

A21233261b_b
Narirutin AREA:N/A S/N:N/A

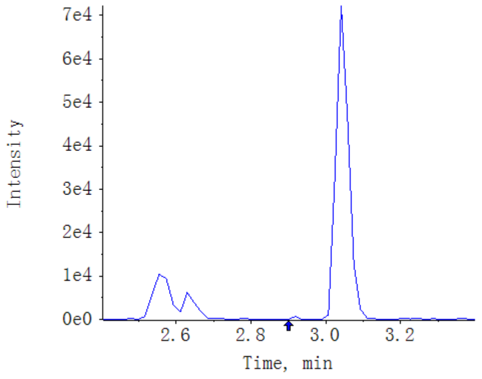

A21233262b_b
Narirutin AREA:N/A S/N:N/A

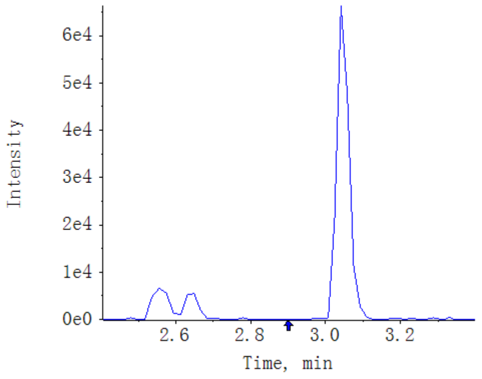

A21233263b_b
Narirutin AREA:4.90e3 S/N:2.1

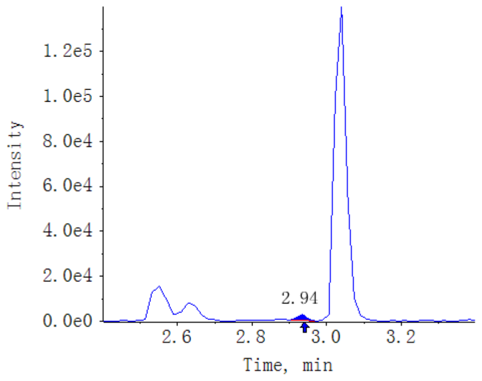

A21233264b_b
Narirutin AREA:N/A S/N:N/A

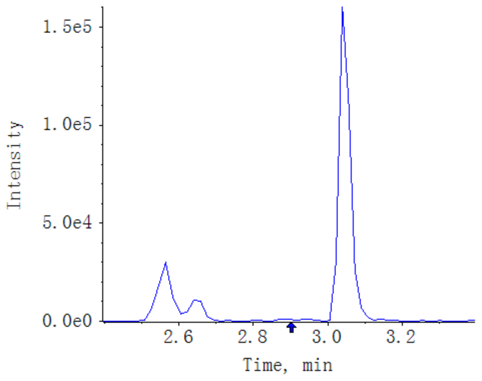

A21233265b_b
Narirutin AREA:2.85e4 S/N:4.6

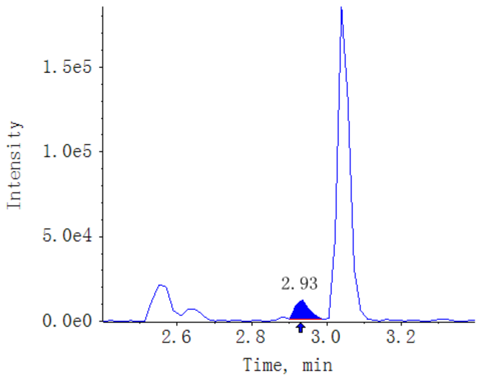

A21233266b_b
Narirutin AREA:1.68e4 S/N:3.4

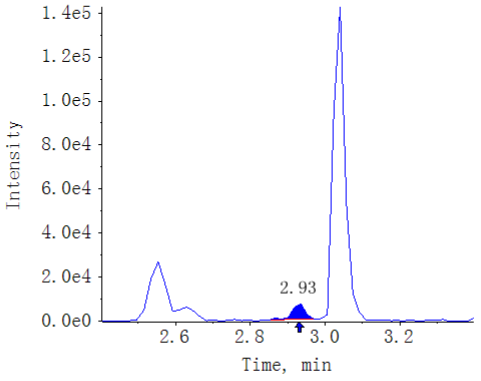

A21233267b_b
Narirutin AREA:9.09e3 S/N:2.4

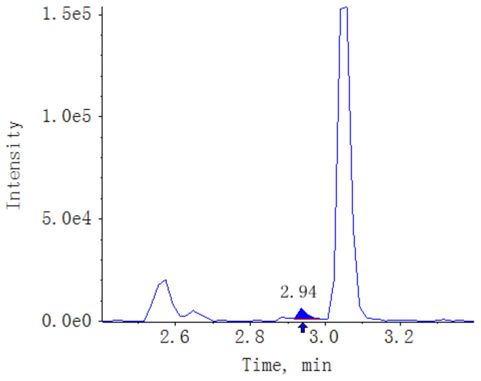

**Result Table**	MWXS-21-2101D-3_18_WH6500-5_A20-3_V1.0_TY_20211028	**Algorithm Used**	MQ4
**Acquisition Method**	Flavonoids_V1.0_WH6500-5_LT_20211025.dam	**Instrument Name**	QTRAP 6500+ Low Mass
**Project**	N/A	**Analytes QTY**	204:44

Compound name: Taxifolin (303.1 / 125.0)

Sample Name	Sample Type	Area (cps)	Is Area (cps)	RT (min)	S/N	Target Conc	Calculated Conc.()
STD_0.5nM	Standard	N/A	N/A	N/A	N/A	0.5000	N/A
STD_1nM	Standard	6.50e3	N/A	3.04	24.2	1.0000	7.982206e-1
STD_5nM	Standard	2.97e4	N/A	3.04	57.0	5.0000	6.833325e0
STD_10nM	Standard	3.61e4	N/A	3.04	50.1	10.0000	8.511472e0
STD_20nM	Standard	7.83e4	N/A	3.04	81.0	20.0000	1.947426e1
STD_50nM	Standard	2.02e5	N/A	3.04	140.9	50.0000	5.165133e1
STD_100nM	Standard	3.75e5	N/A	3.05	131.4	100.0000	9.671414e1
STD_200nM	Standard	7.80e5	N/A	3.04	118.1	200.0000	2.020172e2
STD_500nM	Standard	N/A	N/A	N/A	N/A	500.0000	N/A
STD_1000nM	Standard	N/A	N/A	N/A	N/A	1000.0000	N/A
STD_2000nM	Standard	N/A	N/A	N/A	N/A	2000.0000	N/A
V1.0_MW_RQC1_20211018	Quality Control	N/A	N/A	N/A	N/A	0.0000	N/A
Blank	Unknown	N/A	N/A	N/A	N/A	N/A	N/A
V1.0_MWMS_20211021_1	Unknown	6.08e5	N/A	3.05	105.4	N/A	1.573409e2
MWXS212101D3_R1	Quality Control	6.33e5	N/A	3.04	144.7	0.0000	1.637165e2
MWXS212101D3_R2	Quality Control	6.32e5	N/A	3.05	119.4	0.0000	1.634424e2
MWXS212101D3_R3	Quality Control	6.28e5	N/A	3.05	128.1	0.0000	1.625377e2
A21233250b_b	Unknown	N/A	N/A	N/A	N/A	N/A	N/A
A21233251b_b	Unknown	N/A	N/A	N/A	N/A	N/A	N/A
A21233252b_b	Unknown	N/A	N/A	N/A	N/A	N/A	N/A
A21233253b_b	Unknown	1.62e4	N/A	3.05	27.0	N/A	3.330992e0
A21233254b_b	Unknown	2.36e4	N/A	3.05	39.5	N/A	5.244302e0
A21233255b_b	Unknown	3.01e4	N/A	3.05	45.1	N/A	6.948939e0
A21233256b_b	Unknown	2.67e4	N/A	3.05	38.1	N/A	6.047158e0
A21233257b_b	Unknown	4.72e4	N/A	3.05	49.9	N/A	1.139216e1
A21233258b_b	Unknown	3.24e4	N/A	3.05	36.0	N/A	7.529071e0
A21233259b_b	Unknown	N/A	N/A	N/A	N/A	N/A	N/A
A21233260b_b	Unknown	N/A	N/A	N/A	N/A	N/A	N/A
A21233261b_b	Unknown	N/A	N/A	N/A	N/A	N/A	N/A
A21233262b_b	Unknown	1.13e4	N/A	3.05	18.8	N/A	2.045304e0
A21233263b_b	Unknown	4.94e4	N/A	3.04	55.6	N/A	1.196953e1
A21233264b_b	Unknown	2.41e4	N/A	3.05	32.8	N/A	5.386396e0
A21233265b_b	Unknown	5.50e4	N/A	3.05	49.0	N/A	1.342865e1
A21233266b_b	Unknown	4.19e4	N/A	3.04	52.5	N/A	1.000466e1
A21233267b_b	Unknown	4.91e4	N/A	3.05	45.3	N/A	1.189459e1

Compound name: Taxifolin
Regression Equation: y = 3843.11116 x + 3436.79163 (r = 0.99839) (weighting: 1 / x)

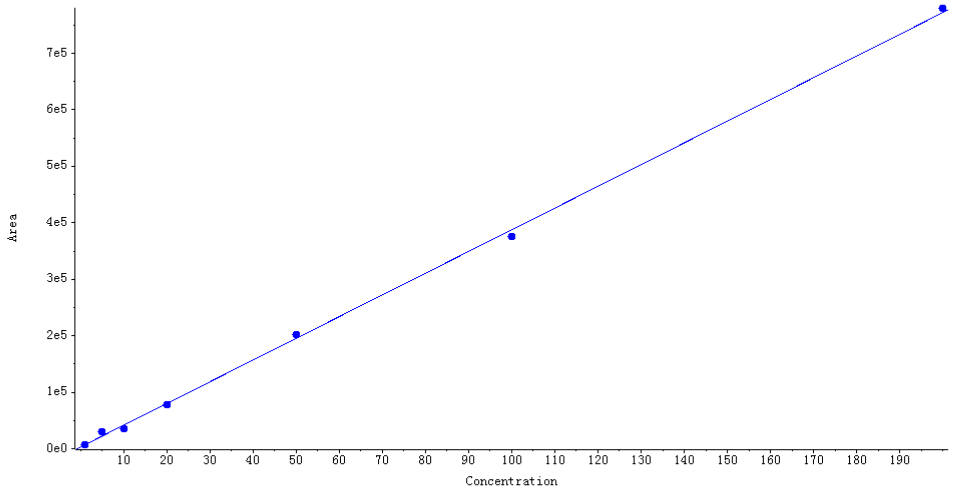

Peak Review

Blank
Taxifolin AREA:N/A S/N:N/A

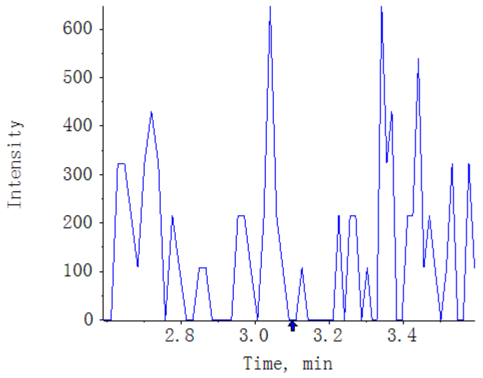

V1.0_MWMS_20211021_1
Taxifolin AREA:6.08e5 S/N:105.4

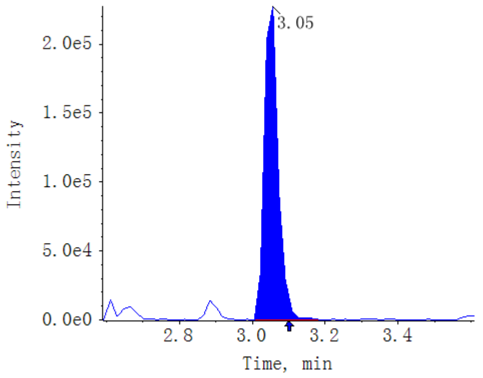

A21233250b_b
Taxifolin AREA:N/A S/N:N/A

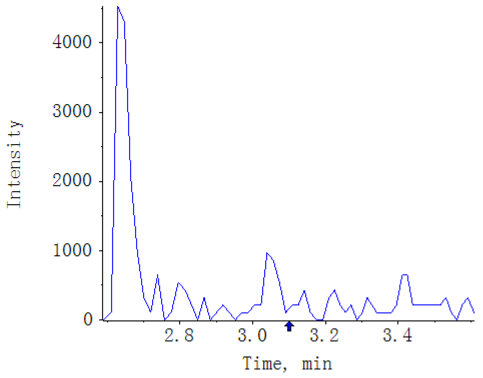

A21233251b_b
Taxifolin AREA:N/A S/N:N/A

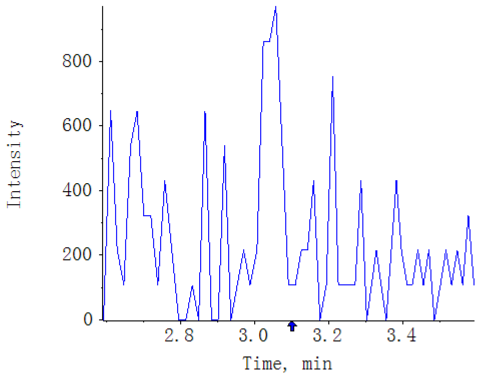

A21233252b_b
Taxifolin AREA:N/A S/N:N/A

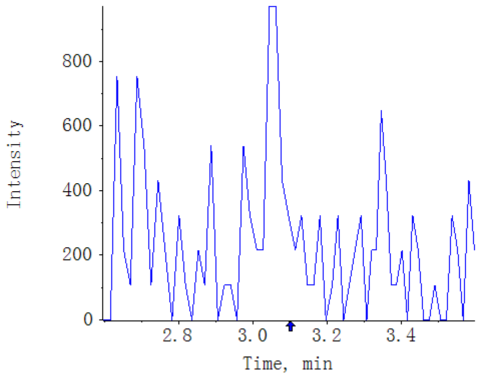

A21233253b_b
Taxifolin AREA:1.62e4 S/N:27.0

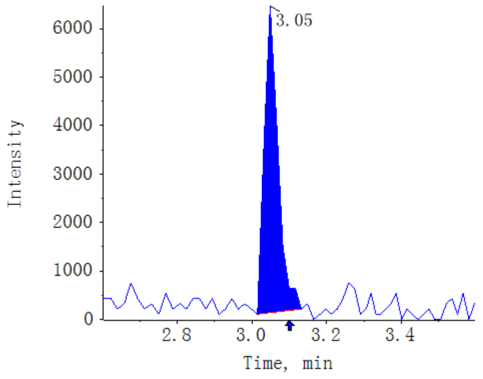

A21233254b_b
Taxifolin AREA:2.36e4 S/N:39.5

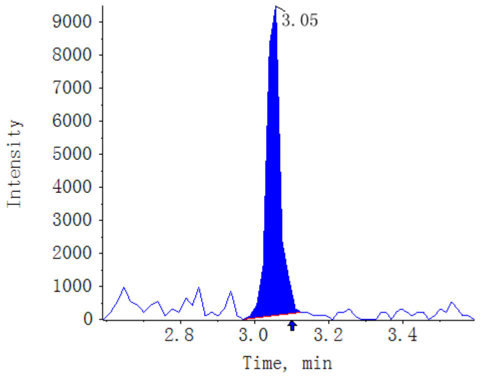

A21233255b_b
Taxifolin AREA:3.01e4 S/N:45.1

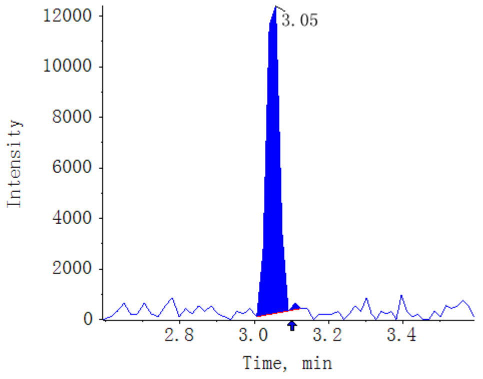

A21233256b_b
Taxifolin AREA:2.67e4 S/N:38.1

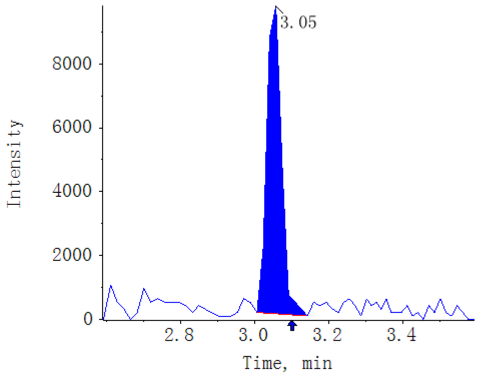

A21233257b_b
Taxifolin AREA:4.72e4 S/N:49.9

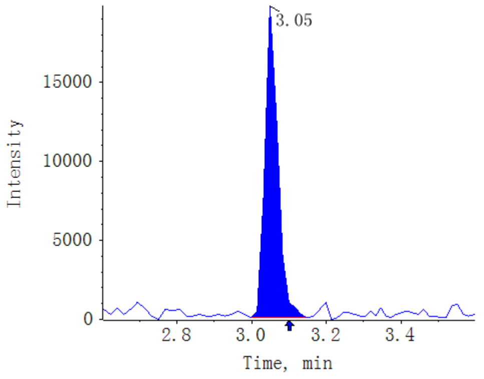

A21233258b_b
Taxifolin AREA:3.24e4 S/N:36.0

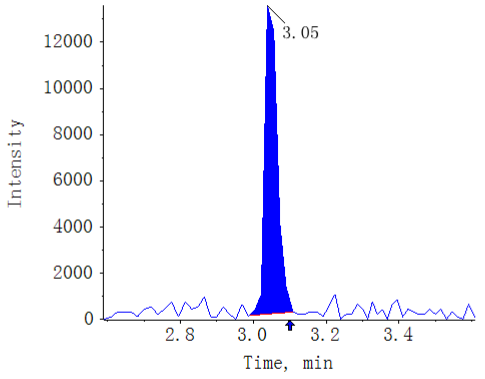

A21233259b_b
Taxifolin AREA:N/A S/N:N/A

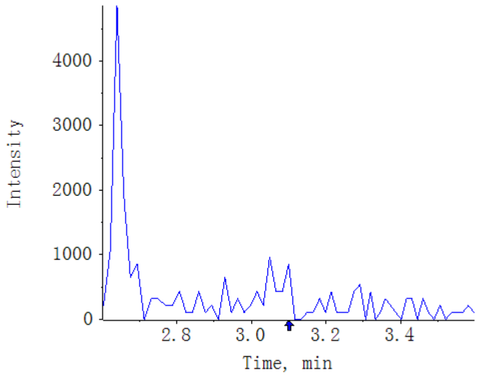

A21233260b_b
Taxifolin AREA:N/A S/N:N/A

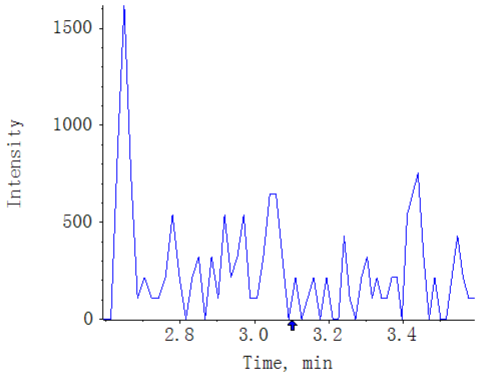

A21233261b_b
Taxifolin AREA:N/A S/N:N/A

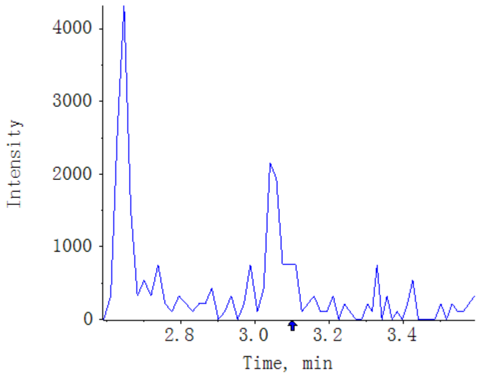

A21233262b_b
Taxifolin AREA:1.13e4 S/N:18.8

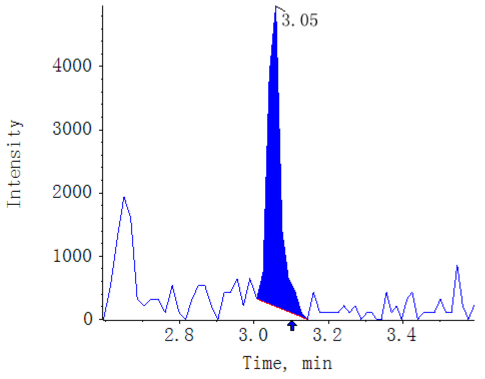

A21233263b_b
Taxifolin AREA:4.94e4 S/N:55.6

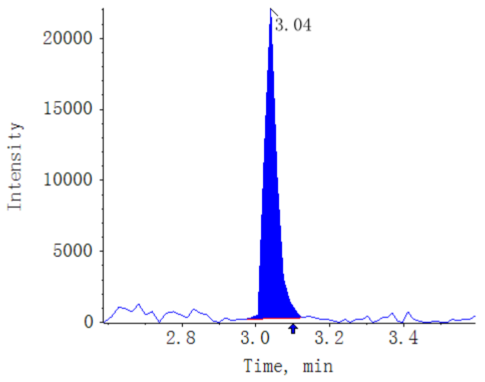

A21233264b_b
Taxifolin AREA:2.41e4 S/N:32.8

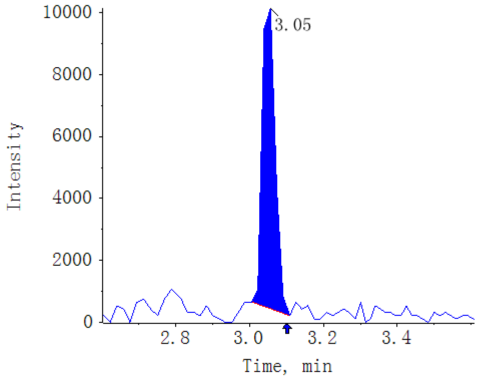

A21233265b_b
Taxifolin AREA:5.50e4 S/N:49.0

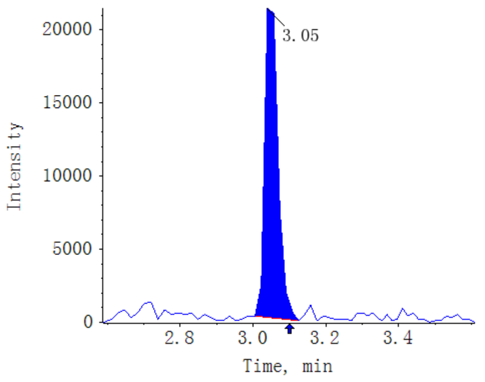

A21233266b_b
Taxifolin AREA:4.19e4 S/N:52.5

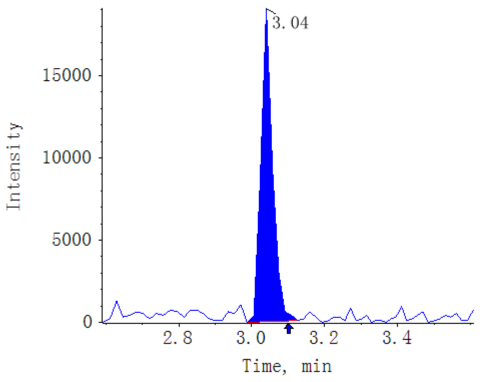

A21233267b_b
Taxifolin AREA:4.91e4 S/N:45.3

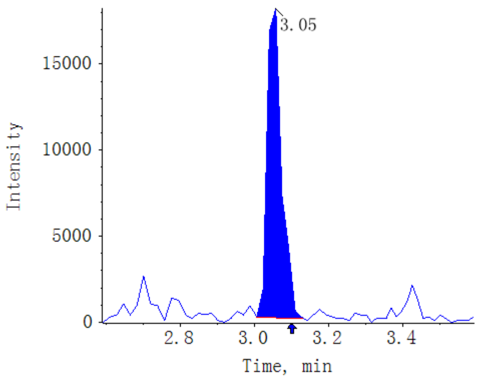

Result Table	MWXS-21-2101D-3_18_WH6500-5_A20-3_V1.0_TY_20211028	Algorithm Used	MQ4
Acquisition Method	Flavonoids_V1.0_WH6500-5_LT_20211025.dam	Instrument Name	QTRAP 6500+ Low Mass
Project	N/A	Analytes QTY	204:8

Compound name: Isoorientin (447.1 / 327.1)

Sample Name	Sample Type	Area (cps)	Is Area (cps)	RT (min)	S/N	Target Conc	Calculated Conc.()
STD_0.5nM	Standard	4.14e3	N/A	2.47	10.6	0.5000	3.056077e-1
STD_1nM	Standard	9.84e3	N/A	2.45	18.4	1.0000	9.171458e-1
STD_5nM	Standard	6.26e4	N/A	2.45	40.5	5.0000	6.580638e0
STD_10nM	Standard	9.79e4	N/A	2.46	41.7	10.0000	1.036990e1
STD_20nM	Standard	1.90e5	N/A	2.46	68.5	20.0000	2.027129e1
STD_50nM	Standard	5.24e5	N/A	2.46	79.3	50.0000	5.611406e1
STD_100nM	Standard	9.76e5	N/A	2.46	60.0	100.0000	1.045938e2
STD_200nM	Standard	1.75e6	N/A	2.45	55.1	200.0000	1.873475e2
STD_500nM	Standard	N/A	N/A	N/A	N/A	500.0000	N/A
STD_1000nM	Standard	N/A	N/A	N/A	N/A	1000.0000	N/A
STD_2000nM	Standard	N/A	N/A	N/A	N/A	2000.0000	N/A
V1.0_MW_RQC1_20211018	Quality Control	2.01e8	N/A	2.45	189.3	0.0000	2.152844e4
Blank	Unknown	N/A	N/A	N/A	N/A	N/A	N/A
V1.0_MWMS_20211021_1	Unknown	1.55e6	N/A	2.46	63.8	N/A	1.660239e2
MWXS212101D3_R1	Quality Control	1.80e6	N/A	2.46	66.4	0.0000	1.933496e2
MWXS212101D3_R2	Quality Control	1.73e6	N/A	2.46	82.6	0.0000	1.853495e2
MWXS212101D3_R3	Quality Control	1.82e6	N/A	2.46	78.0	0.0000	1.954700e2
A21233250b_b	Unknown	N/A	N/A	N/A	N/A	N/A	N/A
A21233251b_b	Unknown	N/A	N/A	N/A	N/A	N/A	N/A
A21233252b_b	Unknown	N/A	N/A	N/A	N/A	N/A	N/A
A21233253b_b	Unknown	N/A	N/A	N/A	N/A	N/A	N/A
A21233254b_b	Unknown	N/A	N/A	N/A	N/A	N/A	N/A
A21233255b_b	Unknown	N/A	N/A	N/A	N/A	N/A	N/A
A21233256b_b	Unknown	1.50e4	N/A	2.46	6.0	N/A	1.470054e0
A21233257b_b	Unknown	N/A	N/A	N/A	N/A	N/A	N/A
A21233258b_b	Unknown	N/A	N/A	N/A	N/A	N/A	N/A
A21233259b_b	Unknown	N/A	N/A	N/A	N/A	N/A	N/A
A21233260b_b	Unknown	N/A	N/A	N/A	N/A	N/A	N/A
A21233261b_b	Unknown	2.00e4	N/A	2.46	3.6	N/A	2.003869e0
A21233262b_b	Unknown	N/A	N/A	N/A	N/A	N/A	N/A
A21233263b_b	Unknown	N/A	N/A	N/A	N/A	N/A	N/A
A21233264b_b	Unknown	N/A	N/A	N/A	N/A	N/A	N/A
A21233265b_b	Unknown	4.15e4	N/A	2.47	6.0	N/A	4.320023e0
A21233266b_b	Unknown	1.23e4	N/A	2.47	3.5	N/A	1.177157e0
A21233267b_b	Unknown	4.38e4	N/A	2.50	7.5	N/A	4.564993e0

Compound name: Isoorientin
Regression Equation: y = 9318.16122 x + 1291.79077 (r = 0.99681) (weighting: 1 / x)

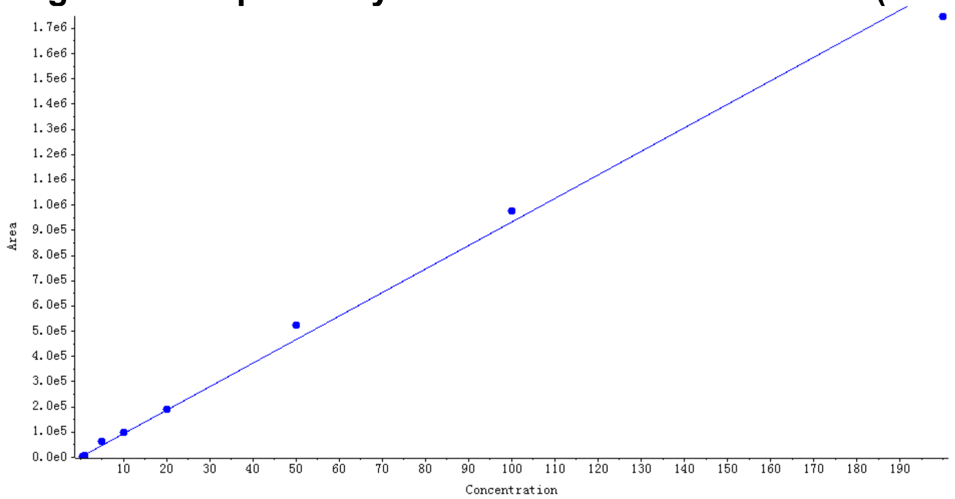

Peak Review

Blank
Isoorientin AREA:N/A S/N:N/A

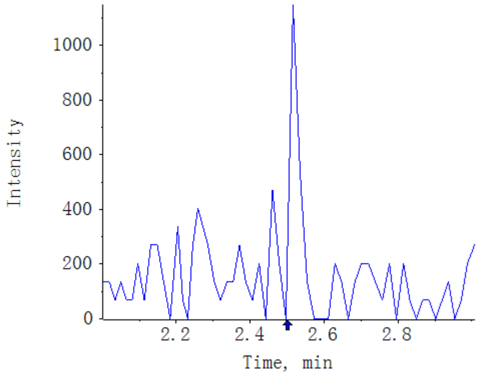

V1.0_MWMS_20211021_1
Isoorientin AREA:1.55e6 S/N:63.8

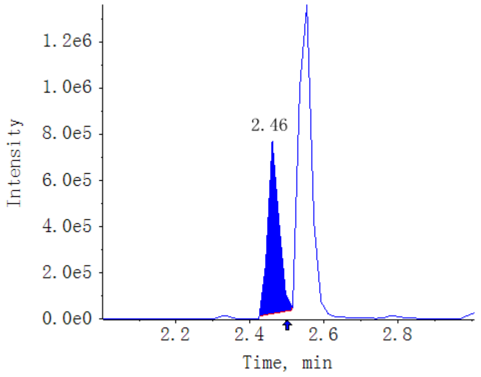

A21233250b_b
Isoorientin AREA:N/A S/N:N/A

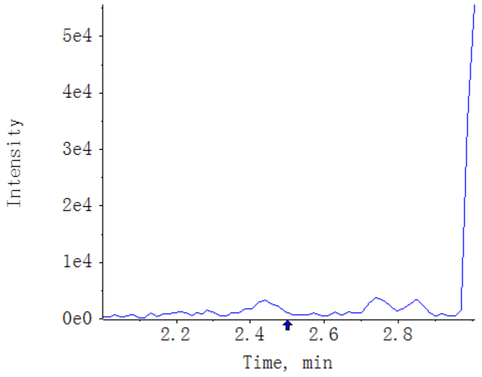

A21233251b_b
Isoorientin AREA:N/A S/N:N/A

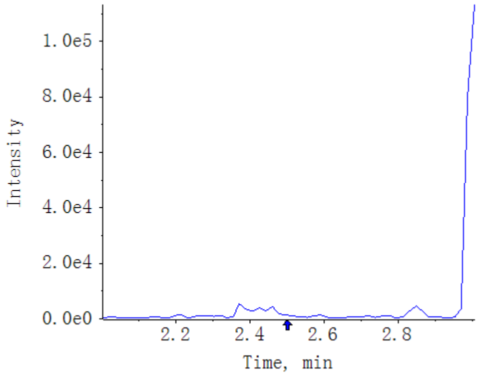

A21233252b_b
Isoorientin AREA:N/A S/N:N/A

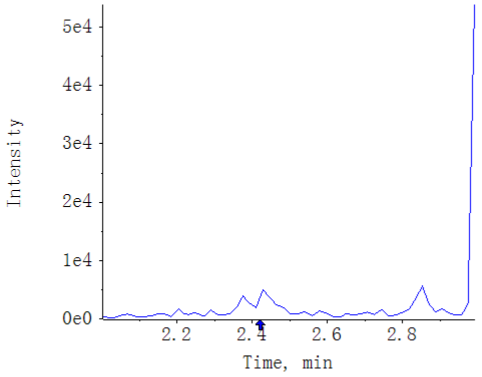

A21233253b_b
Isoorientin AREA:N/A S/N:N/A

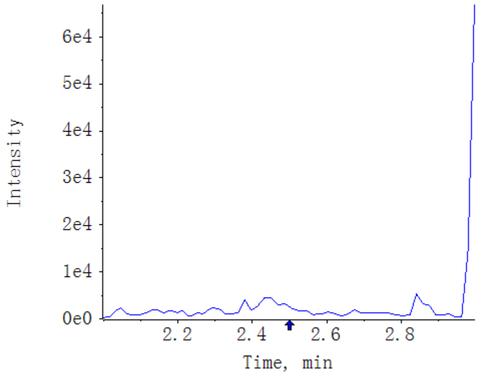

A21233254b_b
Isoorientin AREA:N/A S/N:N/A

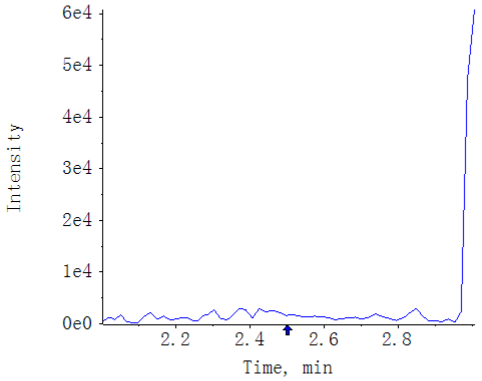

A21233255b_b
Isoorientin AREA:N/A S/N:N/A

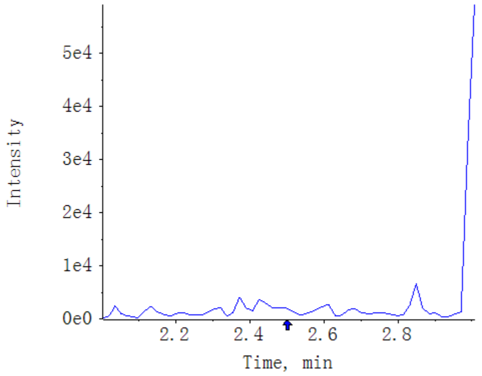

A21233256b_b
Isoorientin AREA:1.50e4 S/N:6.0

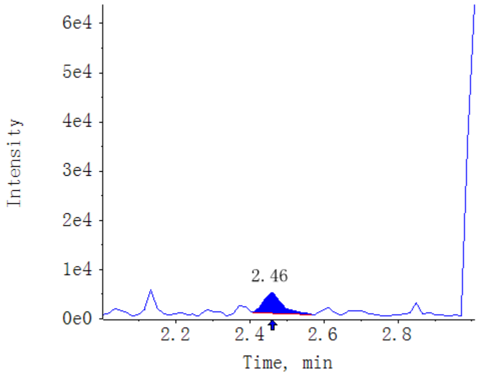

A21233257b_b
Isoorientin AREA:N/A S/N:N/A

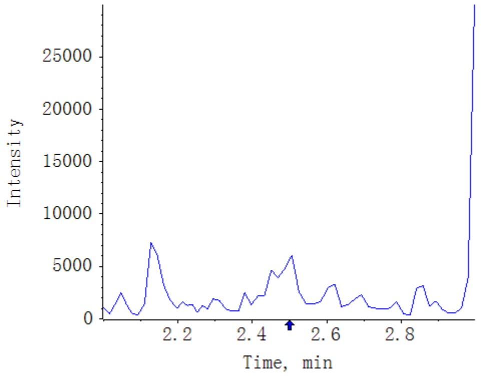

A21233258b_b
Isoorientin AREA:N/A S/N:N/A

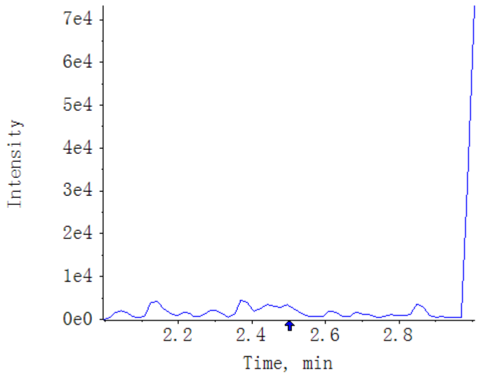

A21233259b_b
Isoorientin AREA:N/A S/N:N/A

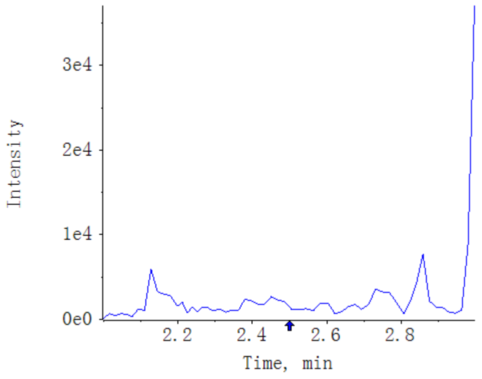

A21233260b_b
Isoorientin AREA:N/A S/N:N/A

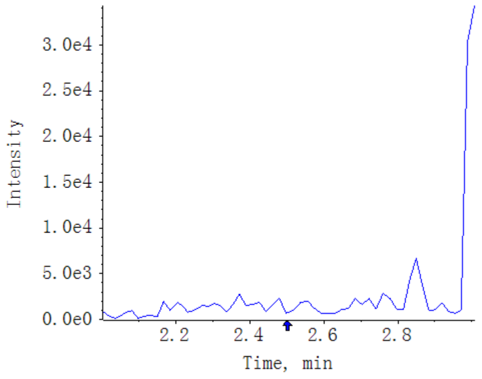

A21233261b_b
Isoorientin AREA:2.00e4 S/N:3.6

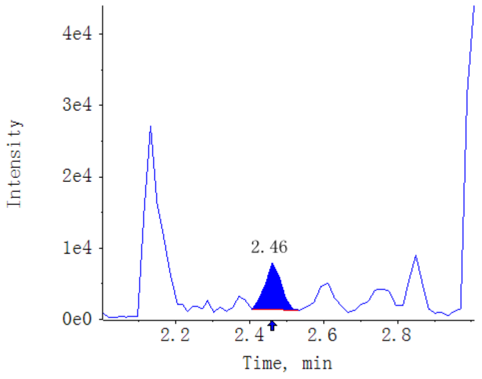

A21233262b_b
Isoorientin AREA:N/A S/N:N/A

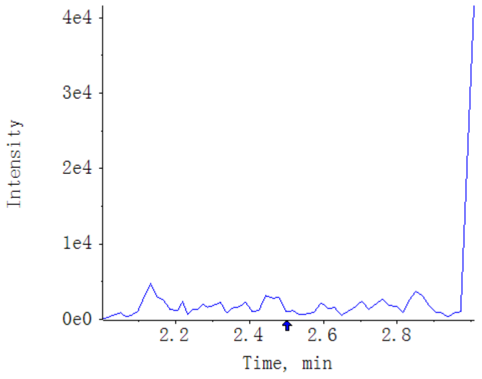

A21233263b_b
Isoorientin AREA:N/A S/N:N/A

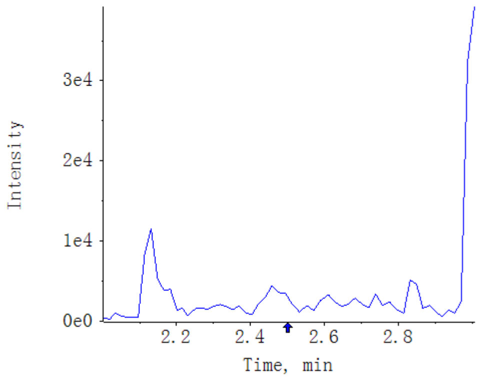

A21233264b_b
Isoorientin AREA:N/A S/N:N/A

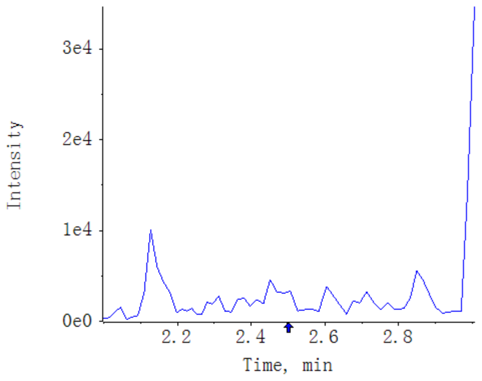

A21233265b_b
Isoorientin AREA:4.15e4 S/N:6.0

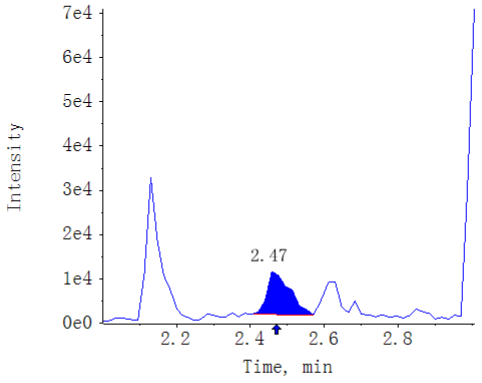

A21233266b_b
Isoorientin AREA:1.23e4 S/N:3.5

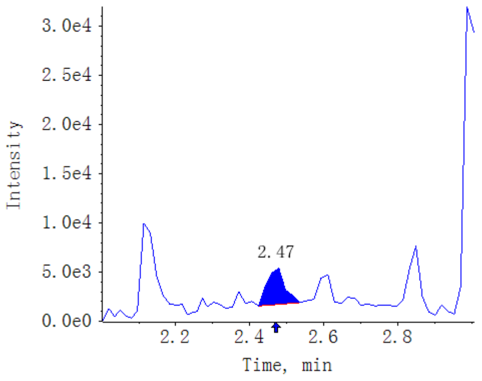

A21233267b_b
Isoorientin AREA:4.38e4 S/N:7.5

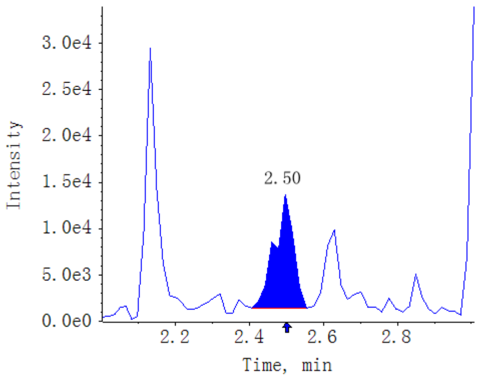

Result Table	MWXS-21-2101D-3_18_WH6500-5_A20-3_V1.0_TY_20211028	Algorithm Used	MQ4
Acquisition Method	Flavonoids_V1.0_WH6500-5_LT_20211025.dam	Instrument Name	QTRAP 6500+ Low Mass
Project	N/A	Analytes QTY	204:18

Compound name: Scutellarin (461.1 / 285.1)

Sample Name	Sample Type	Area (cps)	Is Area (cps)	RT (min)	S/N	Target Conc	Calculated Conc.()
STD_0.5nM	Standard	4.71e3	N/A	2.77	15.4	0.5000	3.060360e-1
STD_1nM	Standard	1.03e4	N/A	2.75	31.0	1.0000	1.051839e0
STD_5nM	Standard	5.43e4	N/A	2.76	73.7	5.0000	6.867594e0
STD_10nM	Standard	8.14e4	N/A	2.76	90.9	10.0000	1.044781e1
STD_20nM	Standard	1.44e5	N/A	2.75	122.0	20.0000	1.870851e1
STD_50nM	Standard	3.74e5	N/A	2.76	142.4	50.0000	4.911822e1
STD_100nM	Standard	N/A	N/A	N/A	N/A	100.0000	N/A
STD_200nM	Standard	N/A	N/A	N/A	N/A	200.0000	N/A
STD_500nM	Standard	N/A	N/A	N/A	N/A	500.0000	N/A
STD_1000nM	Standard	N/A	N/A	N/A	N/A	1000.0000	N/A
STD_2000nM	Standard	N/A	N/A	N/A	N/A	2000.0000	N/A
V1.0_MW_RQC1_20211018	Quality Control	1.34e5	N/A	2.78	8.2	0.0000	1.740114e1
Blank	Unknown	N/A	N/A	N/A	N/A	N/A	N/A
V1.0_MWMS_20211021_1	Unknown	1.23e6	N/A	2.76	182.6	N/A	1.625174e2
MWXS212101D3_R1	Quality Control	1.26e6	N/A	2.76	154.6	0.0000	1.662598e2
MWXS212101D3_R2	Quality Control	1.24e6	N/A	2.76	214.7	0.0000	1.635520e2
MWXS212101D3_R3	Quality Control	1.24e6	N/A	2.76	217.7	0.0000	1.642279e2
A21233250b_b	Unknown	N/A	N/A	N/A	N/A	N/A	N/A
A21233251b_b	Unknown	N/A	N/A	N/A	N/A	N/A	N/A
A21233252b_b	Unknown	N/A	N/A	N/A	N/A	N/A	N/A
A21233253b_b	Unknown	N/A	N/A	N/A	N/A	N/A	N/A
A21233254b_b	Unknown	N/A	N/A	N/A	N/A	N/A	N/A
A21233255b_b	Unknown	N/A	N/A	N/A	N/A	N/A	N/A
A21233256b_b	Unknown	N/A	N/A	N/A	N/A	N/A	N/A
A21233257b_b	Unknown	N/A	N/A	N/A	N/A	N/A	N/A
A21233258b_b	Unknown	N/A	N/A	N/A	N/A	N/A	N/A
A21233259b_b	Unknown	N/A	N/A	N/A	N/A	N/A	N/A
A21233260b_b	Unknown	3.94e4	N/A	2.77	55.5	N/A	4.889510e0
A21233261b_b	Unknown	N/A	N/A	N/A	N/A	N/A	N/A
A21233262b_b	Unknown	N/A	N/A	N/A	N/A	N/A	N/A
A21233263b_b	Unknown	N/A	N/A	N/A	N/A	N/A	N/A
A21233264b_b	Unknown	N/A	N/A	N/A	N/A	N/A	N/A
A21233265b_b	Unknown	N/A	N/A	N/A	N/A	N/A	N/A
A21233266b_b	Unknown	1.23e4	N/A	2.77	15.2	N/A	1.306075e0
A21233267b_b	Unknown	N/A	N/A	N/A	N/A	N/A	N/A

Compound name: Scutellarin
Regression Equation: y = 7559.63455 x + 2397.74094 (r = 0.99415) (weighting: 1 / x)

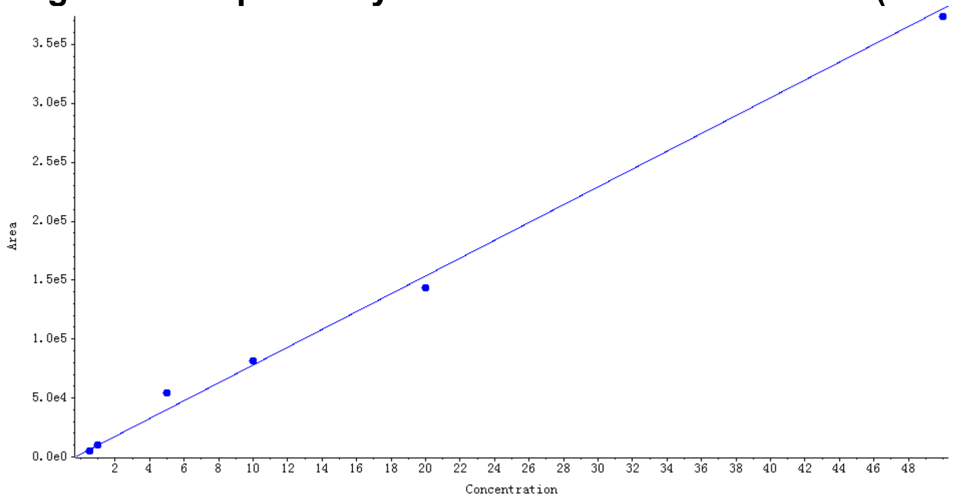

Peak Review

Blank
Scutellarin AREA:N/A S/N:N/A

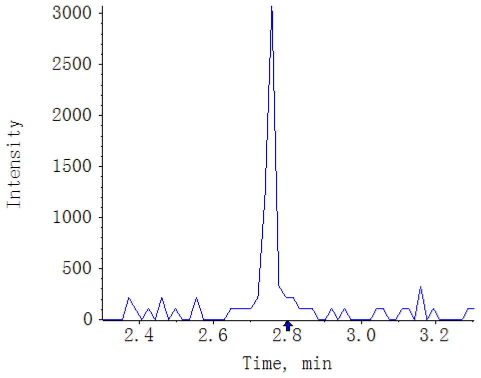

V1.0_MWMS_20211021_1
Scutellarin AREA:1.23e6 S/N:182.6

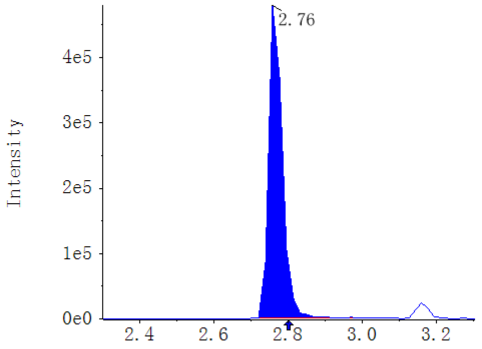

A21233250b_b
Scutellarin AREA:N/A S/N:N/A

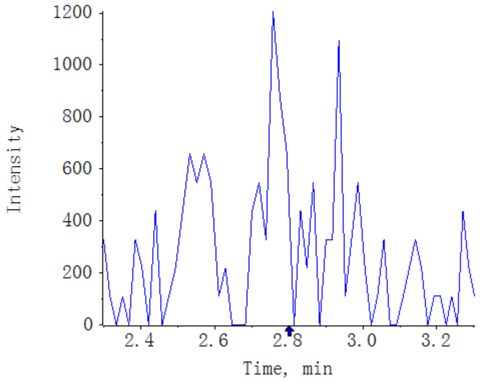

A21233251b_b
Scutellarin AREA:N/A S/N:N/A

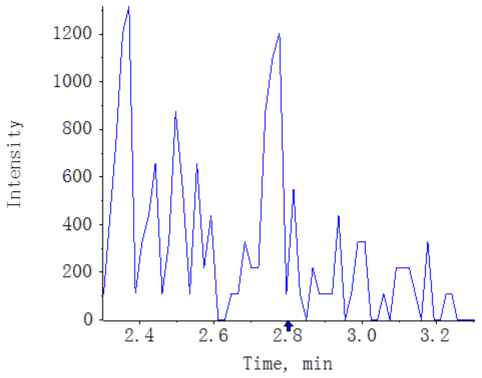

A21233252b_b
Scutellarin AREA:N/A S/N:N/A

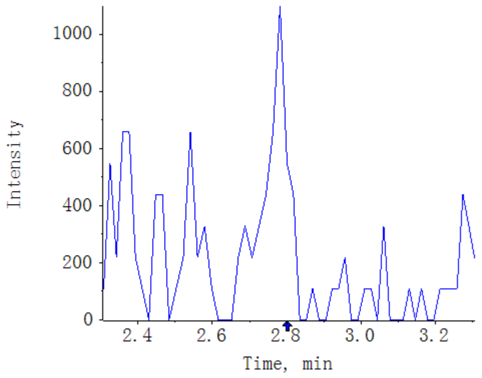

A21233253b_b
Scutellarin AREA:N/A S/N:N/A

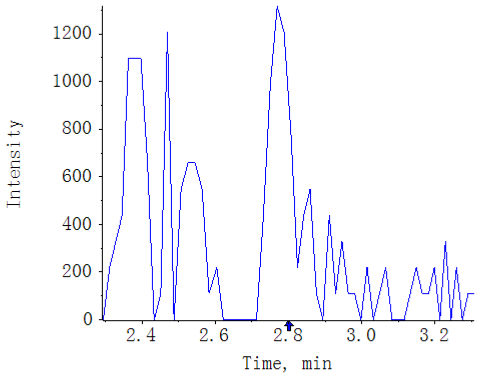

A21233254b_b
Scutellarin AREA:N/A S/N:N/A

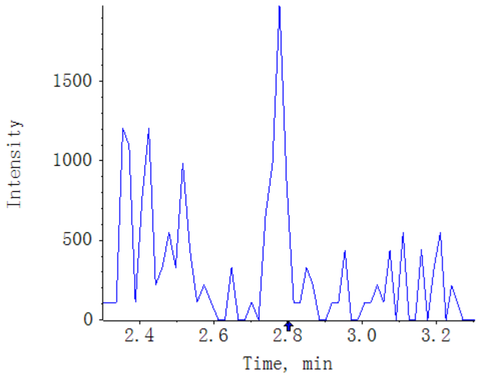

A21233255b_b
Scutellarin AREA:N/A S/N:N/A

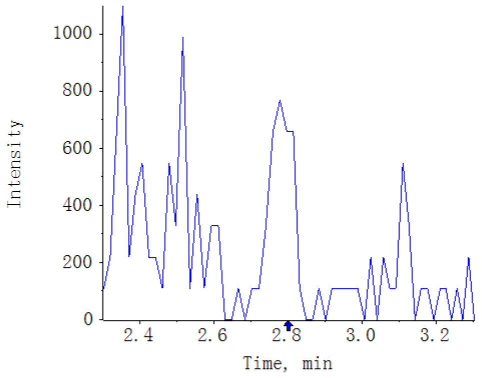

A21233256b_b
Scutellarin AREA:N/A S/N:N/A

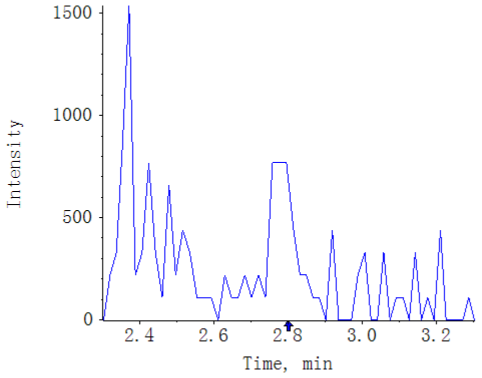

A21233257b_b
Scutellarin AREA:N/A S/N:N/A

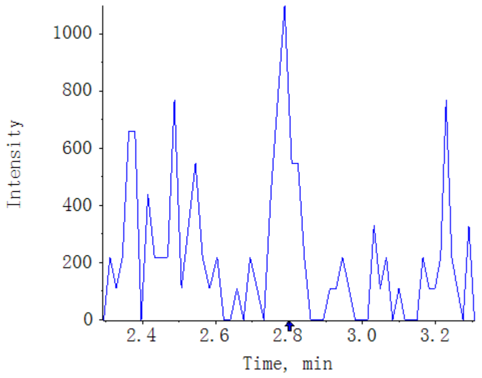

A21233258b_b
Scutellarin AREA:N/A S/N:N/A

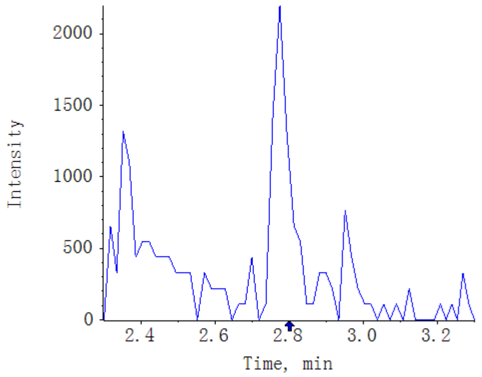

A21233259b_b
Scutellarin AREA:N/A S/N:N/A

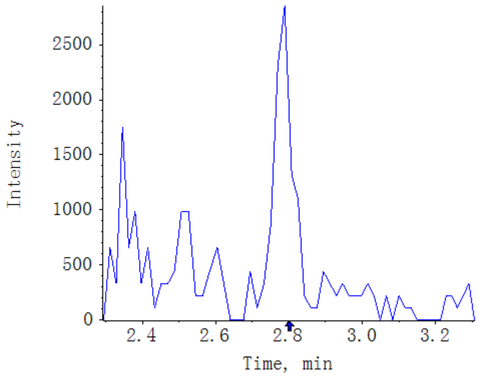

A21233260b_b
Scutellarin AREA:3.94e4 S/N:55.5

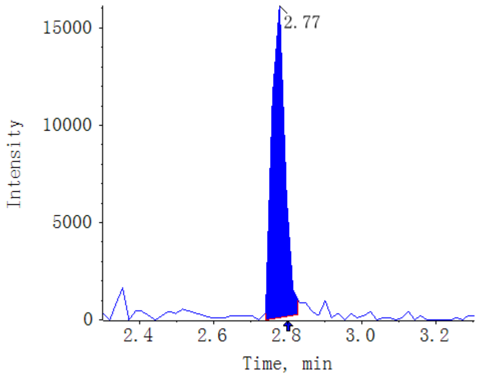

A21233261b_b
Scutellarin AREA:N/A S/N:N/A

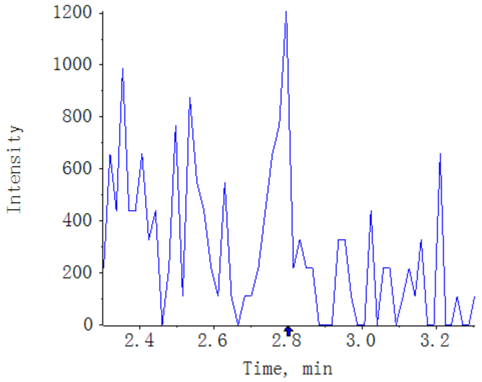

A21233262b_b
Scutellarin AREA:N/A S/N:N/A

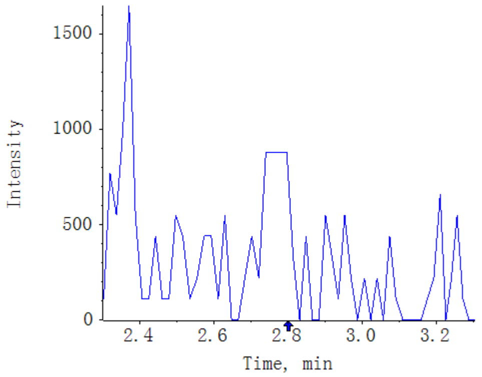

A21233263b_b
Scutellarin AREA:N/A S/N:N/A

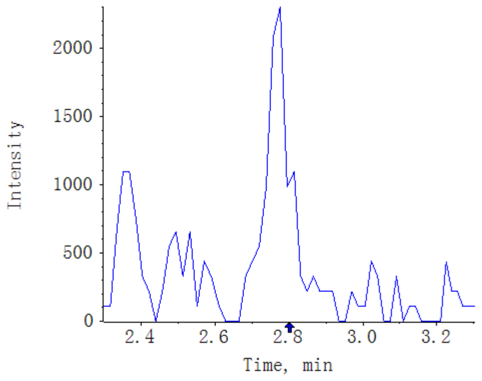

A21233264b_b
Scutellarin AREA:N/A S/N:N/A

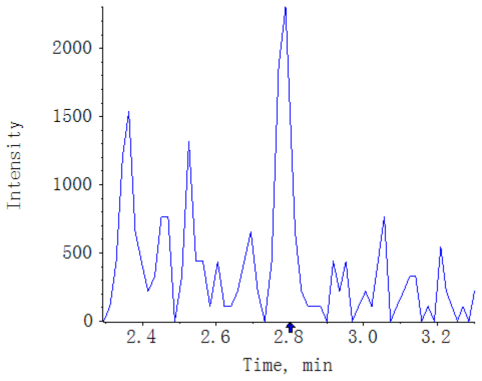

A21233265b_b
Scutellarin AREA:N/A S/N:N/A

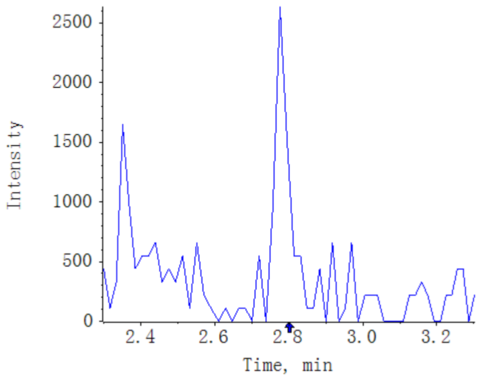

A21233266b_b
Scutellarin AREA:1.23e4 S/N:15.2

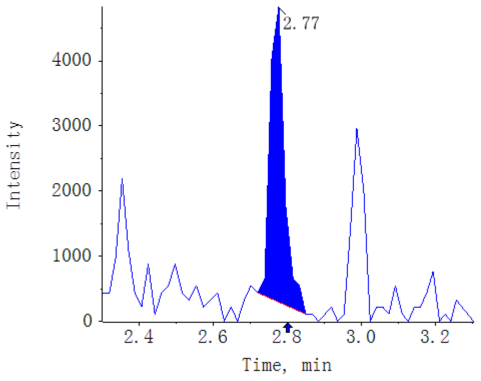

A21233267b_b
Scutellarin AREA:N/A S/N:N/A

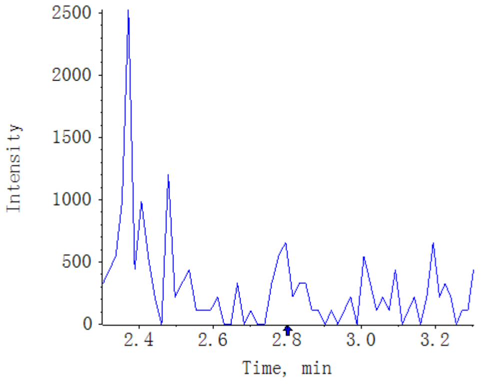

Result Table	MWXS-21-2101D-3_18_WH6500-5_A20-3_V1.0_TY_20211028	Algorithm Used	MQ4
Acquisition Method	Flavonoids_V1.0_WH6500-5_LT_20211025.dam	Instrument Name	QTRAP 6500+ Low Mass
Project	N/A	Analytes QTY	204:16

Compound name: Apigenin-7-glucuronide (445.1 / 269.1)

Sample Name	Sample Type	Area (cps)	Is Area (cps)	RT (min)	S/N	Target Conc	Calculated Conc.()
STD_0.5nM	Standard	4.89e3	N/A	3.09	10.4	0.5000	3.990195e-1
STD_1nM	Standard	8.45e3	N/A	3.09	19.8	1.0000	8.320382e-1
STD_5nM	Standard	6.15e4	N/A	3.09	46.0	5.0000	7.282781e0
STD_10nM	Standard	7.94e4	N/A	3.09	47.9	10.0000	9.453568e0
STD_20nM	Standard	1.59e5	N/A	3.08	56.8	20.0000	1.911185e1
STD_50nM	Standard	4.37e5	N/A	3.09	67.1	50.0000	5.291135e1
STD_100nM	Standard	7.77e5	N/A	3.09	68.7	100.0000	9.432869e1
STD_200nM	Standard	1.66e6	N/A	3.09	57.8	200.0000	2.021807e2
STD_500nM	Standard	N/A	N/A	N/A	N/A	500.0000	N/A
STD_1000nM	Standard	N/A	N/A	N/A	N/A	1000.0000	N/A
STD_2000nM	Standard	N/A	N/A	N/A	N/A	2000.0000	N/A
V1.0_MW_RQC1_20211018	Quality Control	N/A	N/A	N/A	N/A	0.0000	N/A
Blank	Unknown	N/A	N/A	N/A	N/A	N/A	N/A
V1.0_MWMS_20211021_1	Unknown	1.40e6	N/A	3.10	68.7	N/A	1.705808e2
MWXS212101D3_R1	Quality Control	1.43e6	N/A	3.09	69.1	0.0000	1.732445e2
MWXS212101D3_R2	Quality Control	1.42e6	N/A	3.10	70.9	0.0000	1.727081e2
MWXS212101D3_R3	Quality Control	1.37e6	N/A	3.10	66.4	0.0000	1.669639e2
A21233250b_b	Unknown	N/A	N/A	N/A	N/A	N/A	N/A
A21233251b_b	Unknown	N/A	N/A	N/A	N/A	N/A	N/A
A21233252b_b	Unknown	N/A	N/A	N/A	N/A	N/A	N/A
A21233253b_b	Unknown	N/A	N/A	N/A	N/A	N/A	N/A
A21233254b_b	Unknown	N/A	N/A	N/A	N/A	N/A	N/A
A21233255b_b	Unknown	N/A	N/A	N/A	N/A	N/A	N/A
A21233256b_b	Unknown	N/A	N/A	N/A	N/A	N/A	N/A
A21233257b_b	Unknown	N/A	N/A	N/A	N/A	N/A	N/A
A21233258b_b	Unknown	N/A	N/A	N/A	N/A	N/A	N/A
A21233259b_b	Unknown	N/A	N/A	N/A	N/A	N/A	N/A
A21233260b_b	Unknown	4.32e3	N/A	3.12	9.9	N/A	3.289928e-1
A21233261b_b	Unknown	4.06e3	N/A	3.12	9.3	N/A	2.979213e-1
A21233262b_b	Unknown	5.27e3	N/A	3.12	6.4	N/A	4.445803e-1
A21233263b_b	Unknown	5.96e3	N/A	3.11	9.7	N/A	5.282190e-1
A21233264b_b	Unknown	8.87e3	N/A	3.13	12.2	N/A	8.831108e-1
A21233265b_b	Unknown	4.26e3	N/A	3.12	10.9	N/A	3.223444e-1
A21233266b_b	Unknown	4.94e3	N/A	3.12	11.3	N/A	4.041865e-1
A21233267b_b	Unknown	N/A	N/A	N/A	N/A	N/A	N/A

Compound name: Apigenin-7-glucuronide
Regression Equation: y = 8223.25711 x + 1611.52973 (r = 0.99773) (weighting: 1 / x)

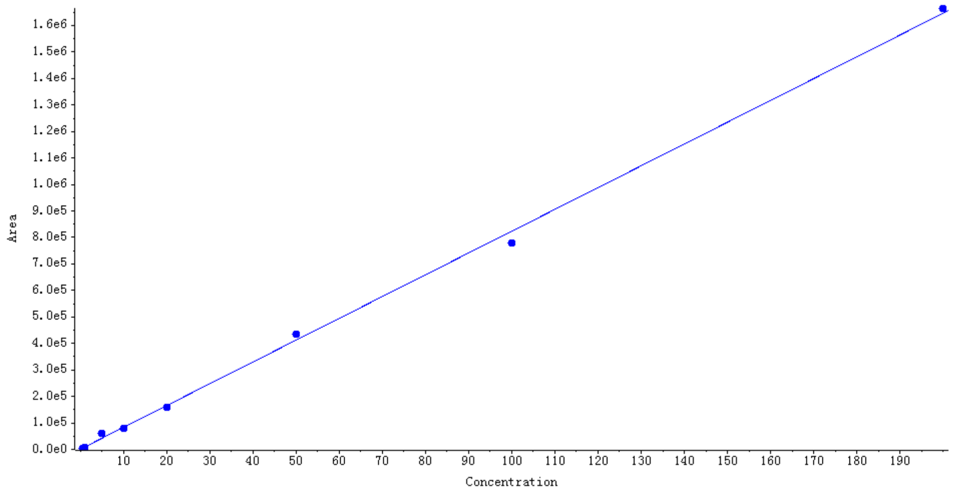

Peak Review

Blank
Apigenin-7-glucuronide AREA:N/A
S/N:N/A

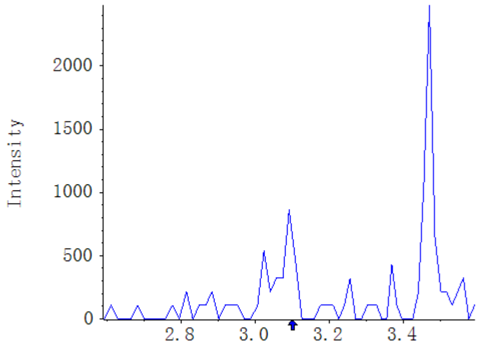

V1.0_MWMS_20211021_1
Apigenin-7-glucuronide
AREA:1.40e6 S/N:68.7

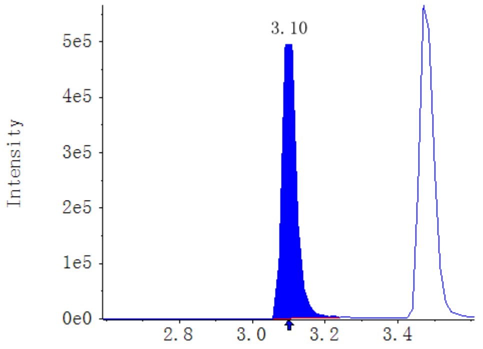

A21233250b_b
Apigenin-7-glucuronide AREA:N/A
S/N:N/A

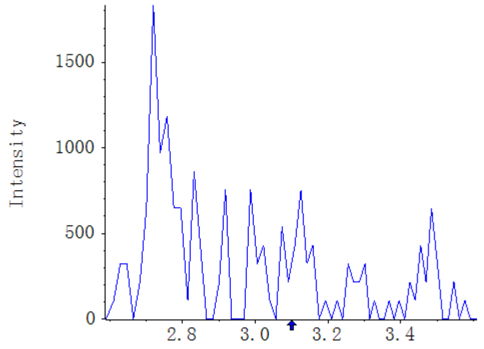

A21233251b_b
Apigenin-7-glucuronide AREA:N/A
S/N:N/A

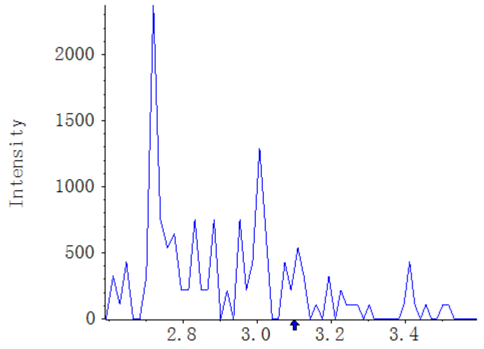

A21233252b_b
Apigenin-7-glucuronide AREA:N/A
S/N:N/A

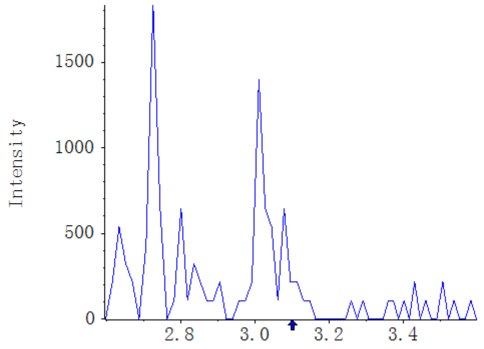

A21233253b_b
Apigenin-7-glucuronide AREA:N/A
S/N:N/A

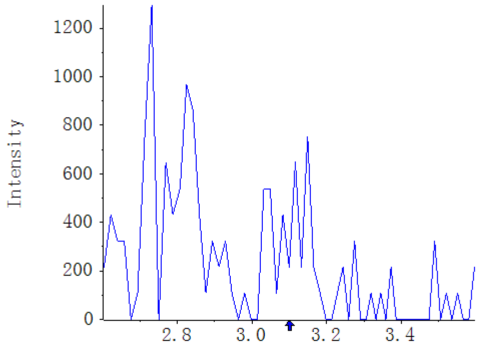

A21233254b_b
Apigenin-7-glucuronide AREA:N/A
S/N:N/A

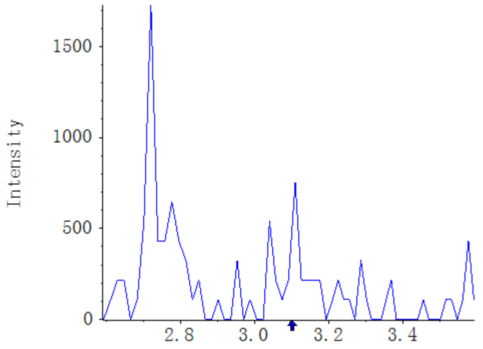

A21233255b_b
Apigenin-7-glucuronide AREA:N/A
S/N:N/A

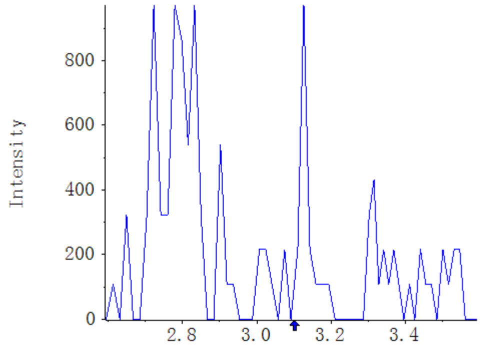

A21233256b_b
Apigenin-7-glucuronide AREA:N/A
S/N:N/A

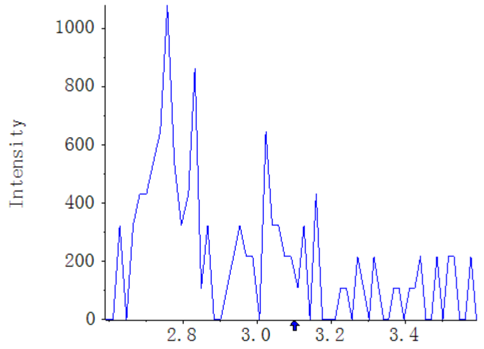

A21233257b_b
Apigenin-7-glucuronide AREA:N/A
S/N:N/A

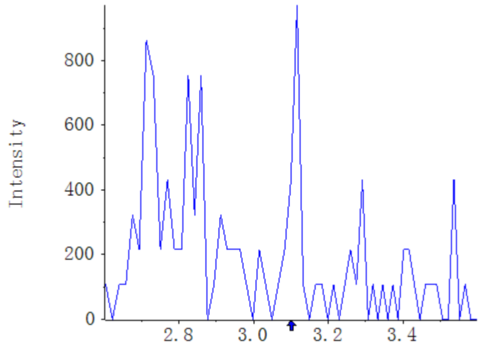

A21233258b_b
Apigenin-7-glucuronide AREA:N/A
S/N:N/A

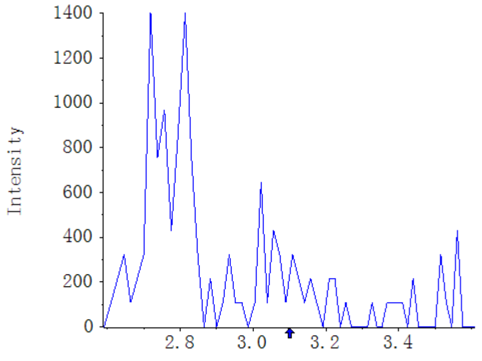

A21233259b_b
Apigenin-7-glucuronide AREA:N/A
S/N:N/A

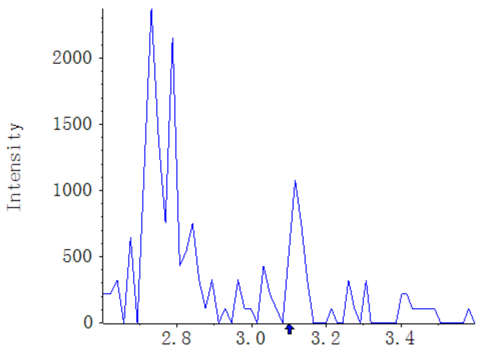

A21233260b_b
Apigenin-7-glucuronide
AREA:4.32e3 S/N:9.9

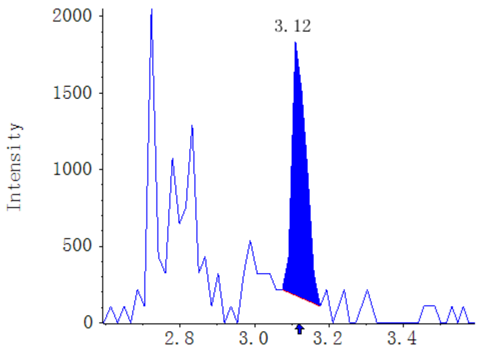

A21233261b_b
Apigenin-7-glucuronide
AREA:4.06e3 S/N:9.3

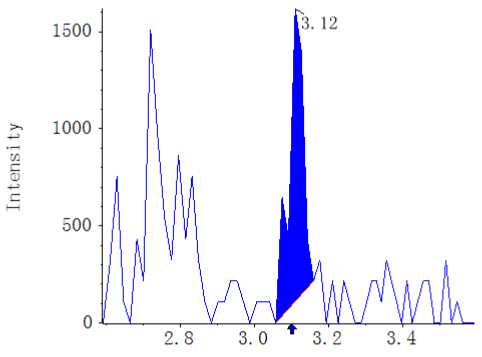

A21233262b_b
Apigenin-7-glucuronide
AREA:5.27e3 S/N:6.4

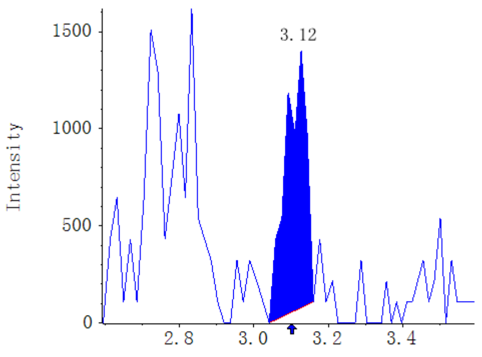

A21233263b_b
Apigenin-7-glucuronide
AREA:5.96e3 S/N:9.7

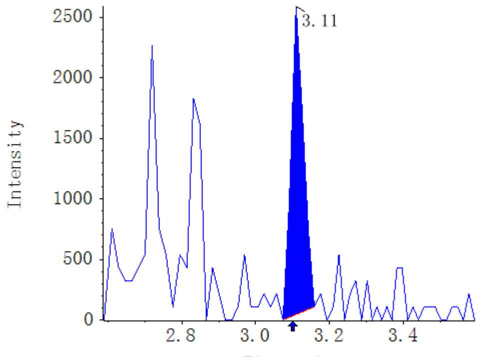

A21233264b_b
Apigenin-7-glucuronide
AREA:8.87e3 S/N:12.2

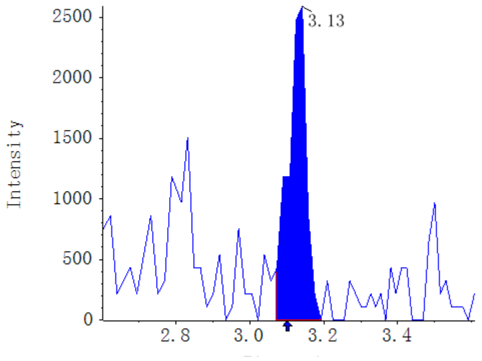

A21233265b_b
Apigenin-7-glucuronide
AREA:4.26e3 S/N:10.9

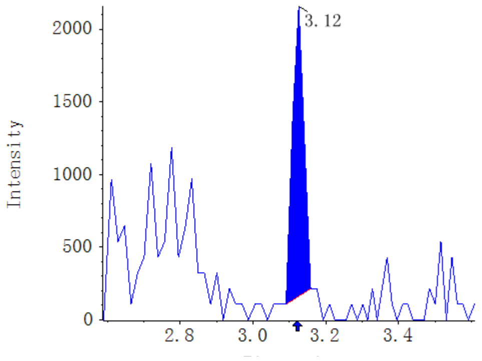

A21233266b_b
Apigenin-7-glucuronide
AREA:4.94e3 S/N:11.3

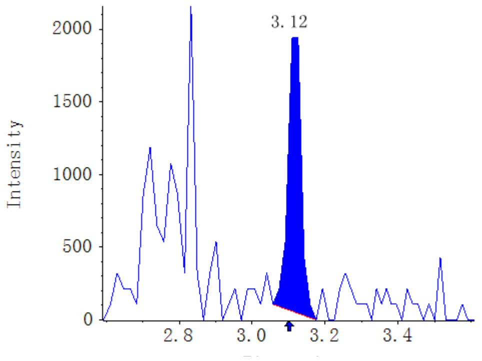

A21233267b_b
Apigenin-7-glucuronide AREA:N/A
S/N:N/A

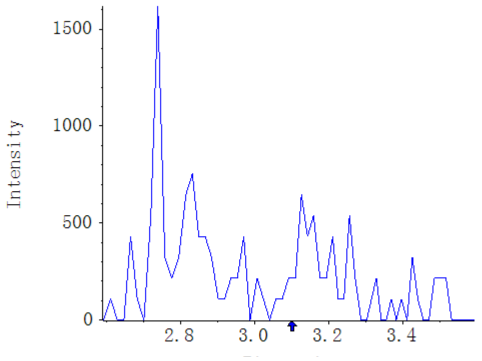

Result Table	MWXS-21-2101D-3_18_WH6500-5_A20-3_V1.0_TY_20211028	Algorithm Used	MQ4
Acquisition Method	Flavonoids_V1.0_WH6500-5_LT_20211025.dam	Instrument Name	QTRAP 6500+ Low Mass
Project	N/A	Analytes QTY	204:38

Compound name: Luteolin (285.0 / 133.0)

Sample Name	Sample Type	Area (cps)	Is Area (cps)	RT (min)	S/N	Target Conc	Calculated Conc.()
STD_0.5nM	Standard	7.32e3	N/A	4.11	16.8	0.5000	5.061871e-1
STD_1nM	Standard	1.11e4	N/A	4.10	19.9	1.0000	8.938487e-1
STD_5nM	Standard	7.09e4	N/A	4.10	51.1	5.0000	7.087917e0
STD_10nM	Standard	9.70e4	N/A	4.10	56.7	10.0000	9.798572e0
STD_20nM	Standard	1.87e5	N/A	4.10	58.1	20.0000	1.914406e1
STD_50nM	Standard	4.79e5	N/A	4.10	67.4	50.0000	4.934395e1
STD_100nM	Standard	9.59e5	N/A	4.11	79.1	100.0000	9.903494e1
STD_200nM	Standard	1.94e6	N/A	4.10	79.2	200.0000	2.006905e2
STD_500nM	Standard	N/A	N/A	N/A	N/A	500.0000	N/A
STD_1000nM	Standard	N/A	N/A	N/A	N/A	1000.0000	N/A
STD_2000nM	Standard	N/A	N/A	N/A	N/A	2000.0000	N/A
V1.0_MW_RQC1_20211018	Quality Control	4.09e5	N/A	4.09	125.8	0.0000	4.207151e1
Blank	Unknown	N/A	N/A	N/A	N/A	N/A	N/A
V1.0_MWMS_20211021_1	Unknown	1.47e6	N/A	4.11	76.4	N/A	1.517010e2
MWXS212101D3_R1	Quality Control	1.45e6	N/A	4.11	80.3	0.0000	1.497892e2
MWXS212101D3_R2	Quality Control	1.48e6	N/A	4.11	77.3	0.0000	1.527118e2
MWXS212101D3_R3	Quality Control	1.46e6	N/A	4.11	69.6	0.0000	1.514360e2
A21233250b_b	Unknown	N/A	N/A	N/A	N/A	N/A	N/A
A21233251b_b	Unknown	N/A	N/A	N/A	N/A	N/A	N/A
A21233252b_b	Unknown	N/A	N/A	N/A	N/A	N/A	N/A
A21233253b_b	Unknown	1.58e4	N/A	4.09	11.4	N/A	1.383480e0
A21233254b_b	Unknown	1.06e4	N/A	4.11	12.8	N/A	8.458341e-1
A21233255b_b	Unknown	1.80e4	N/A	4.10	17.6	N/A	1.609640e0
A21233256b_b	Unknown	2.49e4	N/A	4.11	27.9	N/A	2.332485e0
A21233257b_b	Unknown	3.11e4	N/A	4.12	37.3	N/A	2.965846e0
A21233258b_b	Unknown	4.57e3	N/A	4.11	8.7	N/A	2.221947e-1
A21233259b_b	Unknown	N/A	N/A	N/A	N/A	N/A	N/A
A21233260b_b	Unknown	N/A	N/A	N/A	N/A	N/A	N/A
A21233261b_b	Unknown	N/A	N/A	N/A	N/A	N/A	N/A
A21233262b_b	Unknown	N/A	N/A	N/A	N/A	N/A	N/A
A21233263b_b	Unknown	3.24e4	N/A	4.10	27.5	N/A	3.104900e0
A21233264b_b	Unknown	3.19e4	N/A	4.11	22.2	N/A	3.049338e0
A21233265b_b	Unknown	5.70e4	N/A	4.11	37.2	N/A	5.653376e0
A21233266b_b	Unknown	2.44e4	N/A	4.10	29.5	N/A	2.280720e0
A21233267b_b	Unknown	5.92e4	N/A	4.12	46.0	N/A	5.880323e0

Compound name: Luteolin
Regression Equation: y = 9654.82488 x + 2428.72465 (r = 0.99990) (weighting: None)

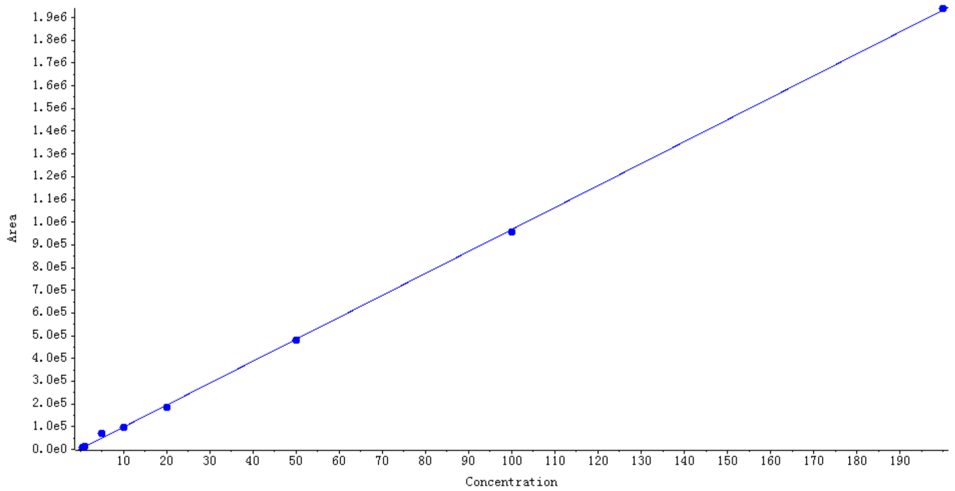

Peak Review

Blank
Luteolin AREA:N/A S/N:N/A

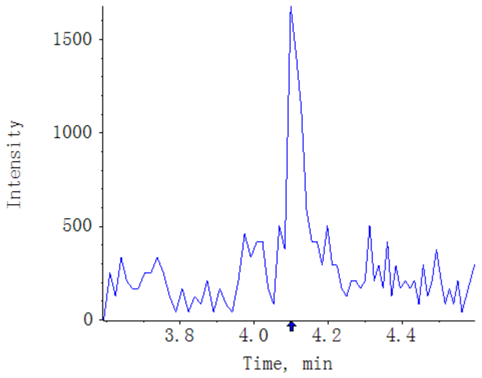

V1.0_MWMS_20211021_1
Luteolin AREA:1.47e6 S/N:76.4

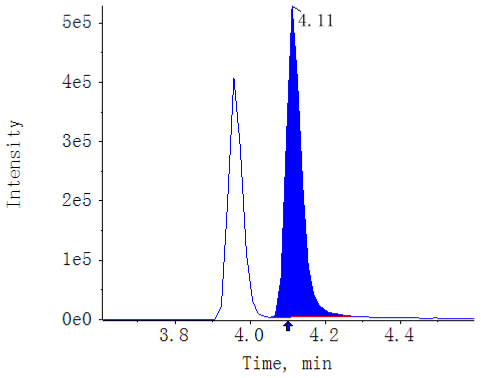

A21233250b_b
Luteolin AREA:N/A S/N:N/A

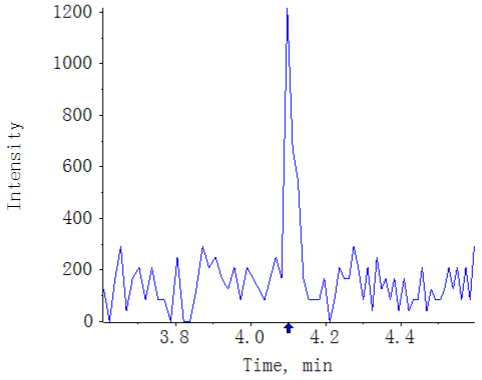

A21233251b_b
Luteolin AREA:N/A S/N:N/A

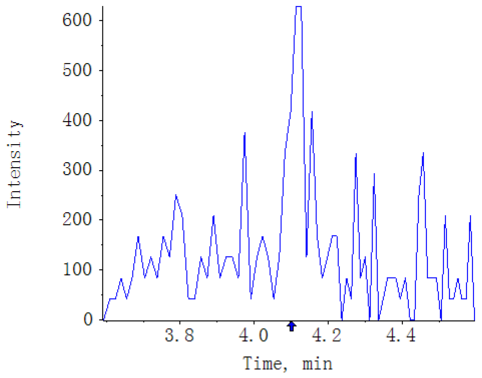

A21233252b_b
Luteolin AREA:N/A S/N:N/A

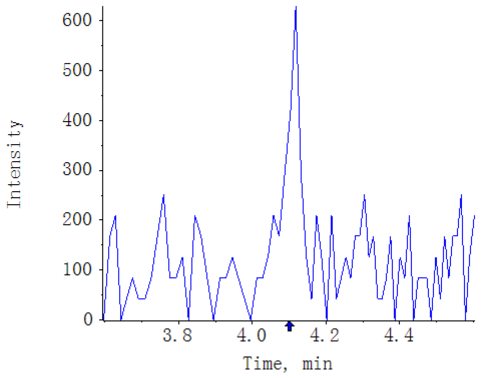

A21233253b_b
Luteolin AREA:1.58e4 S/N:11.4

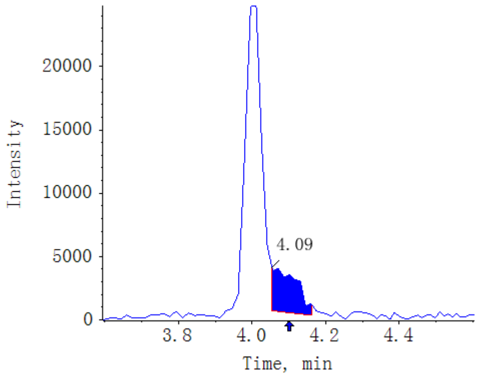

A21233254b_b
Luteolin AREA:1.06e4 S/N:12.8

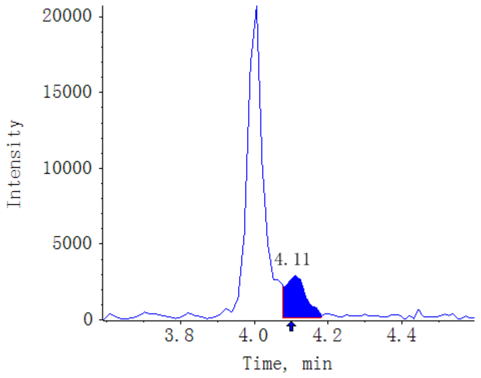

A21233255b_b
Luteolin AREA:1.80e4 S/N:17.6

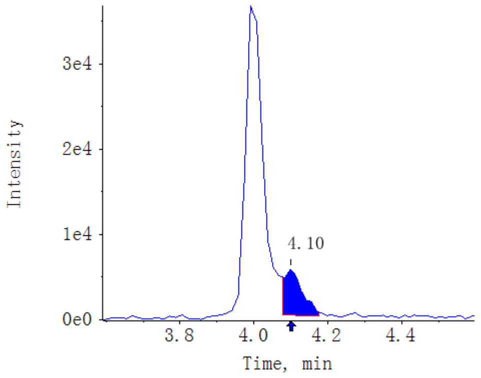

A21233256b_b
Luteolin AREA:2.49e4 S/N:27.9

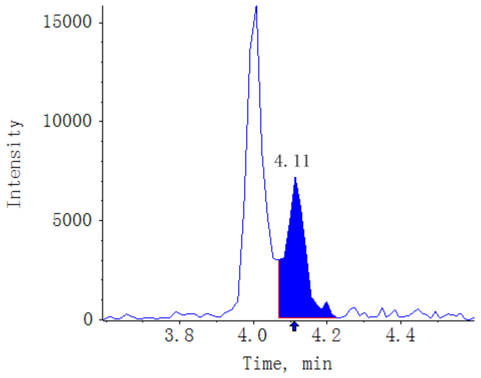

A21233257b_b
Luteolin AREA:3.11e4 S/N:37.3

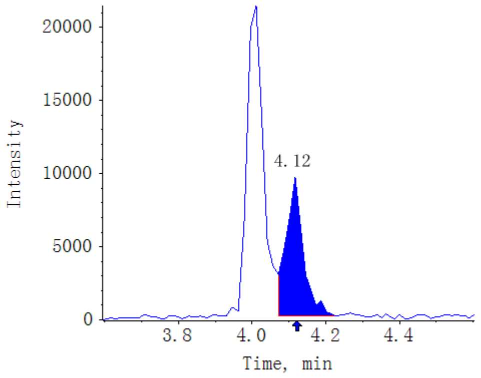

A21233258b_b
Luteolin AREA:4.57e3 S/N:8.7

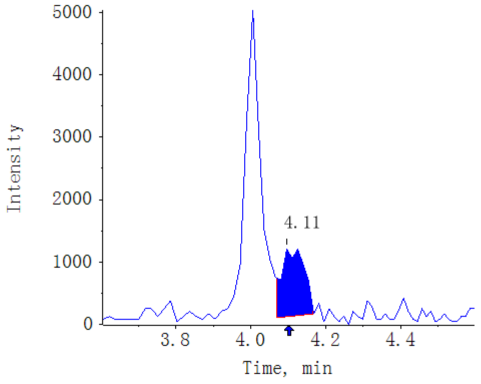

A21233259b_b
Luteolin AREA:N/A S/N:N/A

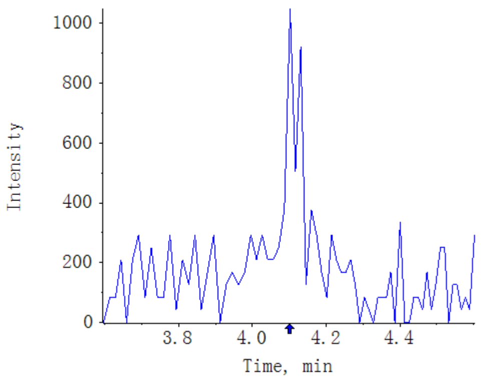

A21233260b_b
Luteolin AREA:N/A S/N:N/A

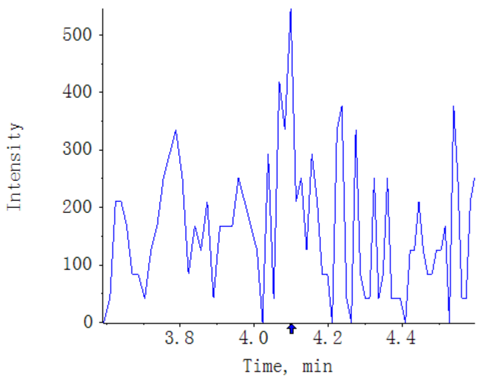

A21233261b_b
Luteolin AREA:N/A S/N:N/A

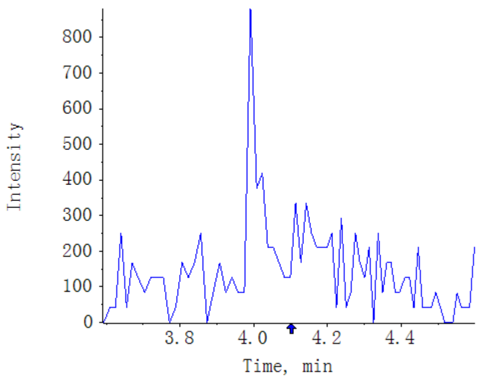

A21233262b_b
Luteolin AREA:N/A S/N:N/A

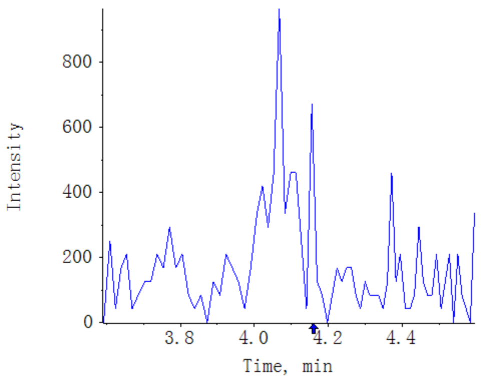

A21233263b_b
Luteolin AREA:3.24e4 S/N:27.5

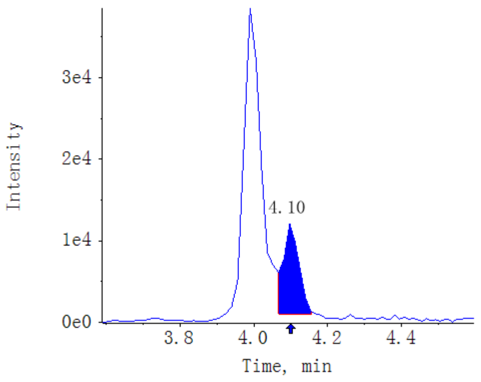

A21233264b_b
Luteolin AREA:3.19e4 S/N:22.2

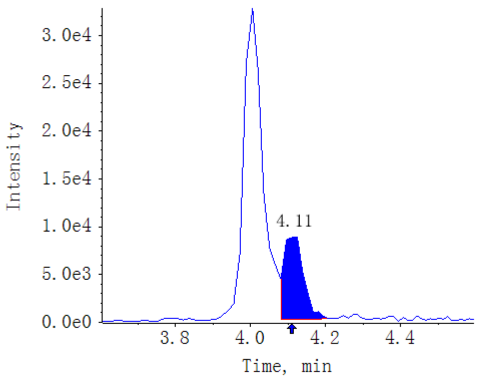

A21233265b_b
Luteolin AREA:5.70e4 S/N:37.2

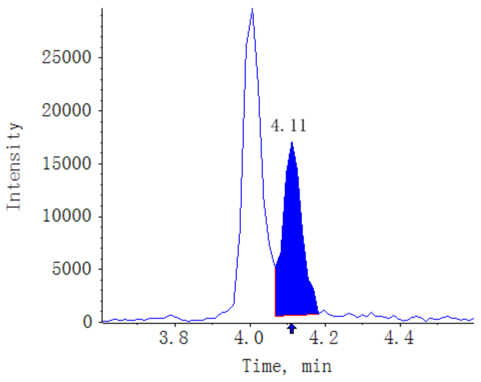

A21233266b_b
Luteolin AREA:2.44e4 S/N:29.5

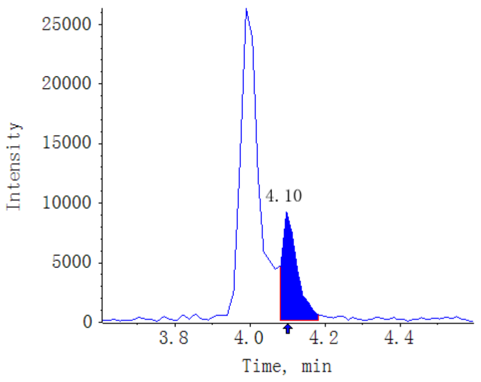

A21233267b_b
Luteolin AREA:5.92e4 S/N:46.0

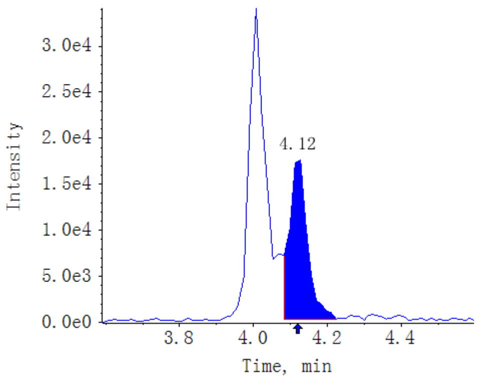

**Result Table**	MWXS-21-2101D-3_18_WH6500-5_A20-3_V1.0_TY_20211028	**Algorithm Used**	MQ4
**Acquisition Method**	Flavonoids_V1.0_WH6500-5_LT_20211025.dam	**Instrument Name**	QTRAP 6500+ Low Mass
**Project**	N/A	**Analytes QTY**	204:14

Compound name: Quercetin (301.1 / 151.0)

Sample Name	Sample Type	Area (cps)	Is Area (cps)	RT (min)	S/N	Target Conc	Calculated Conc.()
STD_0.5nM	Standard	6.09e3	N/A	4.14	17.8	0.5000	4.936760e-1
STD_1nM	Standard	8.65e3	N/A	4.13	23.6	1.0000	7.674545e-1
STD_5nM	Standard	6.19e4	N/A	4.14	86.7	5.0000	6.450162e0
STD_10nM	Standard	9.64e4	N/A	4.14	121.7	10.0000	1.013579e1
STD_20nM	Standard	1.79e5	N/A	4.13	141.6	20.0000	1.896701e1
STD_50nM	Standard	4.68e5	N/A	4.14	177.9	50.0000	4.980782e1
STD_100nM	Standard	9.33e5	N/A	4.15	167.9	100.0000	9.953699e1
STD_200nM	Standard	1.88e6	N/A	4.13	209.6	200.0000	2.003411e2
STD_500nM	Standard	N/A	N/A	N/A	N/A	500.0000	N/A
STD_1000nM	Standard	N/A	N/A	N/A	N/A	1000.0000	N/A
STD_2000nM	Standard	N/A	N/A	N/A	N/A	2000.0000	N/A
V1.0_MW_RQC1_20211018	Quality Control	1.21e4	N/A	4.13	19.5	0.0000	1.140130e0
Blank	Unknown	N/A	N/A	N/A	N/A	N/A	N/A
V1.0_MWMS_20211021_1	Unknown	1.76e6	N/A	4.15	189.8	N/A	1.872914e2
MWXS212101D3_R1	Quality Control	1.71e6	N/A	4.15	192.9	0.0000	1.826029e2
MWXS212101D3_R2	Quality Control	1.73e6	N/A	4.15	171.1	0.0000	1.844329e2
MWXS212101D3_R3	Quality Control	1.71e6	N/A	4.15	187.3	0.0000	1.827556e2
A21233250b_b	Unknown	N/A	N/A	N/A	N/A	N/A	N/A
A21233251b_b	Unknown	N/A	N/A	N/A	N/A	N/A	N/A
A21233252b_b	Unknown	N/A	N/A	N/A	N/A	N/A	N/A
A21233253b_b	Unknown	1.48e5	N/A	4.16	116.6	N/A	1.569020e1
A21233254b_b	Unknown	1.25e5	N/A	4.15	109.1	N/A	1.315622e1
A21233255b_b	Unknown	2.17e5	N/A	4.15	118.8	N/A	2.304047e1
A21233256b_b	Unknown	1.25e5	N/A	4.15	112.6	N/A	1.317362e1
A21233257b_b	Unknown	1.67e5	N/A	4.16	111.5	N/A	1.770215e1
A21233258b_b	Unknown	4.49e4	N/A	4.16	72.6	N/A	4.643420e0
A21233259b_b	Unknown	N/A	N/A	N/A	N/A	N/A	N/A
A21233260b_b	Unknown	N/A	N/A	N/A	N/A	N/A	N/A
A21233261b_b	Unknown	N/A	N/A	N/A	N/A	N/A	N/A
A21233262b_b	Unknown	N/A	N/A	N/A	N/A	N/A	N/A
A21233263b_b	Unknown	1.46e5	N/A	4.14	129.5	N/A	1.545775e1
A21233264b_b	Unknown	1.80e5	N/A	4.16	106.5	N/A	1.902245e1
A21233265b_b	Unknown	1.56e5	N/A	4.15	120.7	N/A	1.655200e1
A21233266b_b	Unknown	1.15e5	N/A	4.15	115.5	N/A	1.215282e1
A21233267b_b	Unknown	7.51e4	N/A	4.16	79.4	N/A	7.860888e0

Compound name: Quercetin
Regression Equation: y = 9363.61356 x + 1467.92020 (r = 0.99928) (weighting: 1 / x)

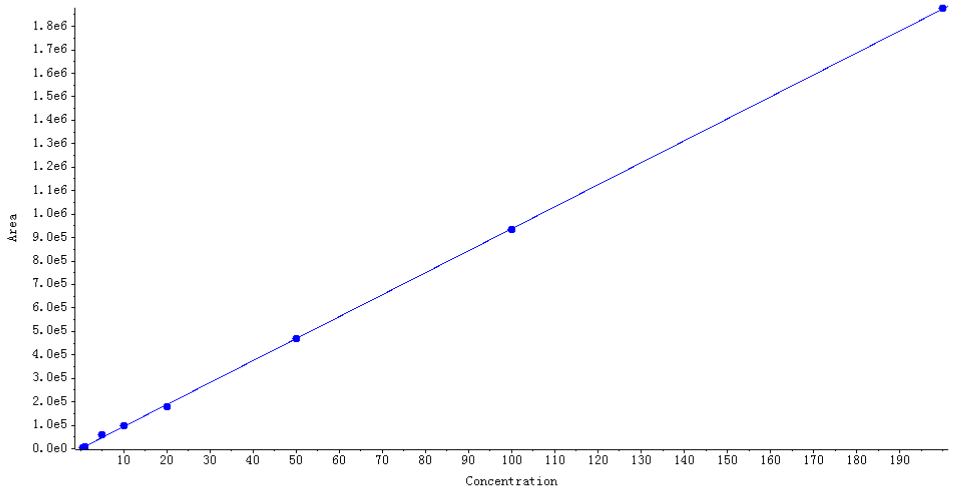

Peak Review

Blank
Quercetin AREA:N/A S/N:N/A

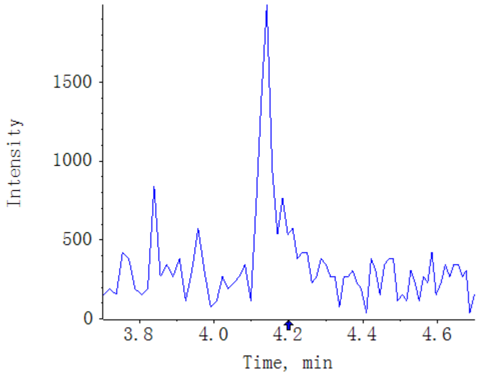

V1.0_MWMS_20211021_1
Quercetin AREA:1.76e6
S/N:189.8

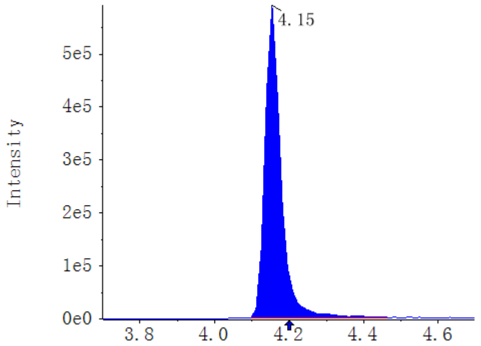

A21233250b_b
Quercetin AREA:N/A S/N:N/A

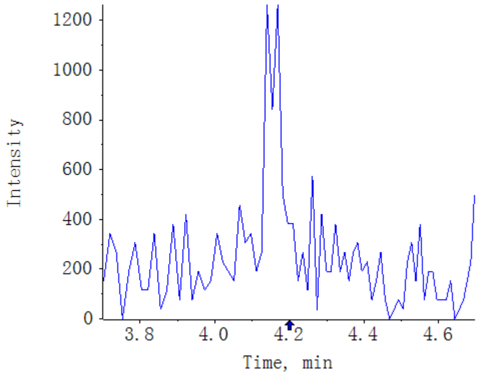

A21233251b_b
Quercetin AREA:N/A S/N:N/A

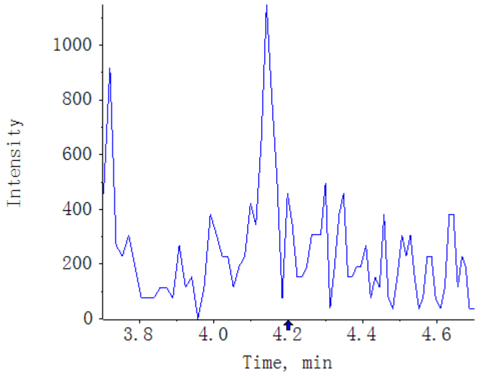

A21233252b_b
Quercetin AREA:N/A S/N:N/A

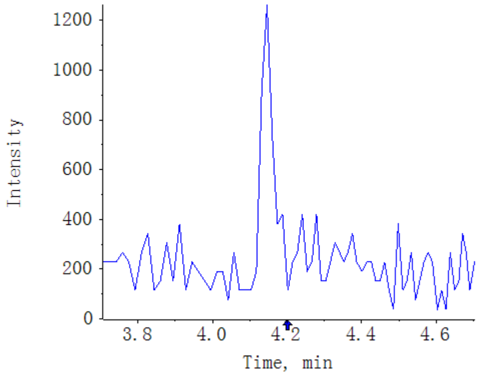

A21233253b_b
Quercetin AREA:1.48e5
S/N:116.6

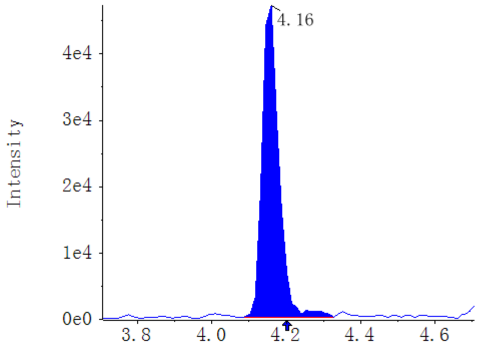

A21233254b_b
Quercetin AREA:1.25e5
S/N:109.1

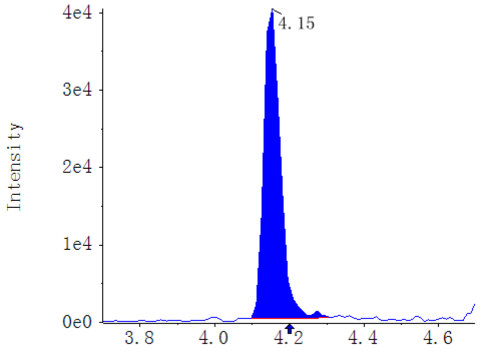

A21233255b_b
Quercetin AREA:2.17e5
S/N:118.8

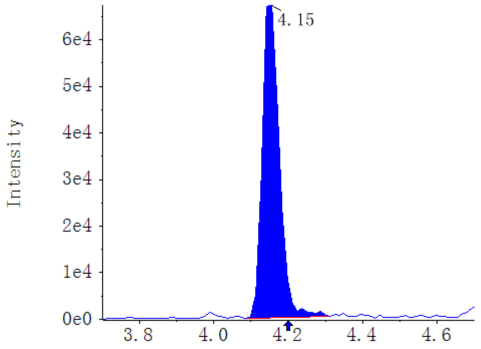

A21233256b_b
Quercetin AREA:1.25e5
S/N:112.6

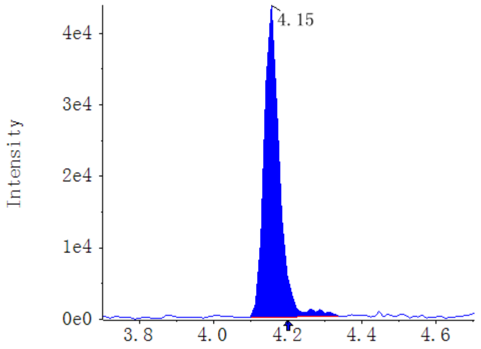

A21233257b_b
Quercetin AREA:1.67e5
S/N:111.5

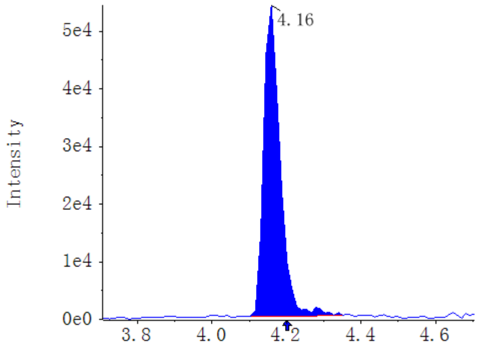

A21233258b_b
Quercetin AREA:4.49e4 S/N:72.6

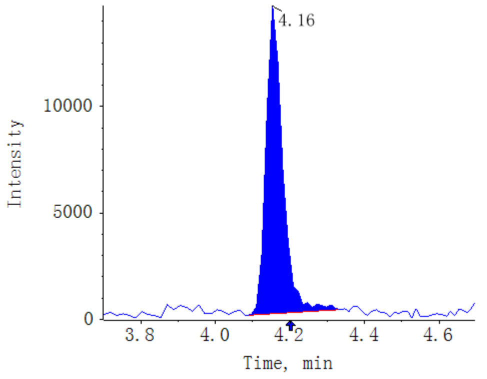

A21233259b_b
Quercetin AREA:N/A S/N:N/A

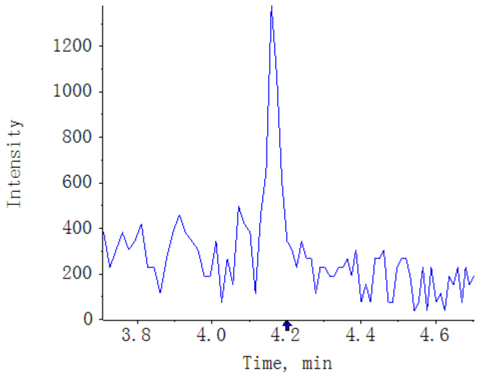

A21233260b_b
Quercetin AREA:N/A S/N:N/A

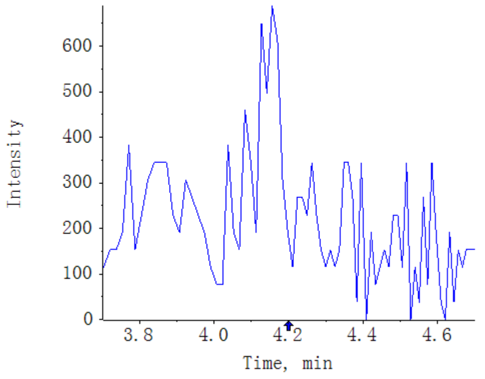

A21233261b_b
Quercetin AREA:N/A S/N:N/A

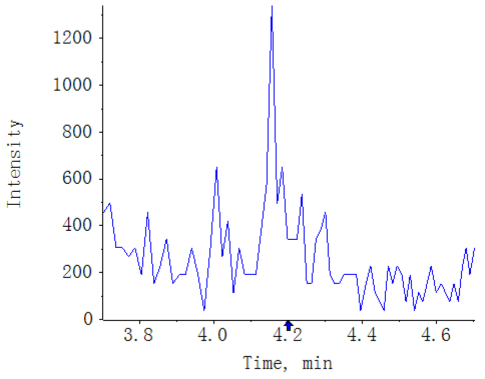

A21233262b_b
Quercetin AREA:N/A S/N:N/A

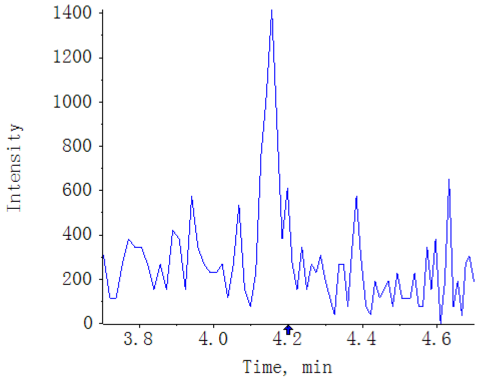

A21233263b_b
Quercetin AREA:1.46e5
S/N:129.5

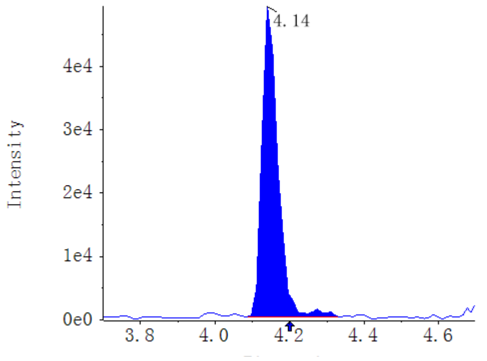

A21233264b_b
Quercetin AREA:1.80e5
S/N:106.5

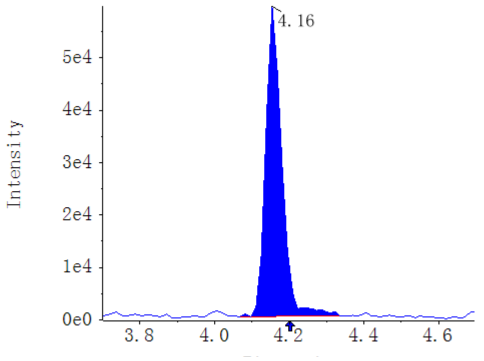

A21233265b_b
Quercetin AREA:1.56e5
S/N:120.7

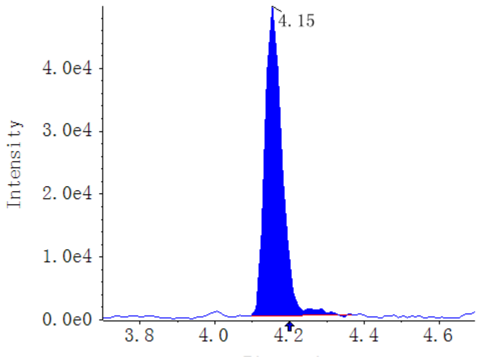

A21233266b_b
Quercetin AREA:1.15e5
S/N:115.5

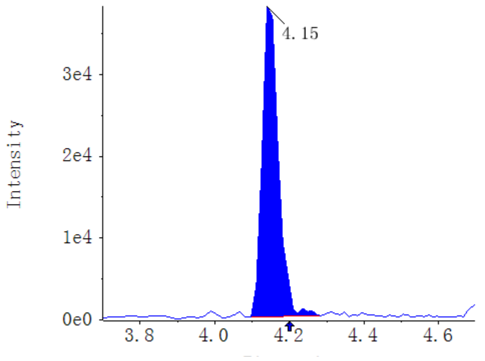

A21233267b_b
Quercetin AREA:7.51e4 S/N:79.4

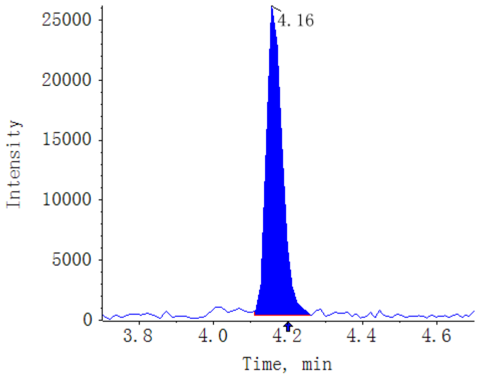

**Result Table**	MWXS-21-2101D-3_18_WH6500-5_A20-3_V1.0_TY_20211028	**Algorithm Used**	MQ4
**Acquisition Method**	Flavonoids_V1.0_WH6500-5_LT_20211025.dam	**Instrument Name**	QTRAP 6500+ Low Mass
**Project**	N/A	**Analytes QTY**	204:1

Compound name: Miquelianin (477.1 / 301.0)

Sample Name	Sample Type	Area (cps)	Is Area (cps)	RT (min)	S/N	Target Conc	Calculated Conc.()
STD_0.5nM	Standard	7.18e3	N/A	2.76	28.6	0.5000	6.226589e-1
STD_1nM	Standard	8.44e3	N/A	2.75	22.3	1.0000	7.504903e-1
STD_5nM	Standard	5.86e4	N/A	2.75	135.6	5.0000	5.846787e0
STD_10nM	Standard	9.39e4	N/A	2.75	180.0	10.0000	9.428521e0
STD_20nM	Standard	1.78e5	N/A	2.75	184.4	20.0000	1.796654e1
STD_50nM	Standard	4.94e5	N/A	2.76	184.7	50.0000	5.009470e1
STD_100nM	Standard	9.52e5	N/A	2.76	195.8	100.0000	9.656202e1
STD_200nM	Standard	2.02e6	N/A	2.75	222.5	200.0000	2.052283e2
STD_500nM	Standard	N/A	N/A	N/A	N/A	500.0000	N/A
STD_1000nM	Standard	N/A	N/A	N/A	N/A	1000.0000	N/A
STD_2000nM	Standard	N/A	N/A	N/A	N/A	2000.0000	N/A
V1.0_MW_RQC1_20211018	Quality Control	N/A	N/A	N/A	N/A	0.0000	N/A
Blank	Unknown	N/A	N/A	N/A	N/A	N/A	N/A
V1.0_MWMS_20211021_1	Unknown	1.67e6	N/A	2.76	223.5	N/A	1.690883e2
MWXS212101D3_R1	Quality Control	1.74e6	N/A	2.76	255.1	0.0000	1.768292e2
MWXS212101D3_R2	Quality Control	1.67e6	N/A	2.76	234.1	0.0000	1.693909e2
MWXS212101D3_R3	Quality Control	1.66e6	N/A	2.76	245.1	0.0000	1.684236e2
A21233250b_b	Unknown	9.48e3	N/A	2.75	8.3	N/A	8.560279e-1
A21233251b_b	Unknown	1.06e4	N/A	2.75	14.9	N/A	9.714955e-1
A21233252b_b	Unknown	1.16e4	N/A	2.75	12.7	N/A	1.071944e0
A21233253b_b	Unknown	1.64e4	N/A	2.76	16.8	N/A	1.564147e0
A21233254b_b	Unknown	1.26e4	N/A	2.75	17.8	N/A	1.171416e0
A21233255b_b	Unknown	1.73e4	N/A	2.76	16.3	N/A	1.650977e0
A21233256b_b	Unknown	1.70e4	N/A	2.76	25.6	N/A	1.614991e0
A21233257b_b	Unknown	1.48e4	N/A	2.76	14.7	N/A	1.399024e0
A21233258b_b	Unknown	2.35e4	N/A	2.76	25.2	N/A	2.278555e0
A21233259b_b	Unknown	8.97e3	N/A	2.76	13.5	N/A	8.044643e-1
A21233260b_b	Unknown	1.53e4	N/A	2.76	18.9	N/A	1.451240e0
A21233261b_b	Unknown	1.15e4	N/A	2.76	14.3	N/A	1.057247e0
A21233262b_b	Unknown	1.14e4	N/A	2.76	12.0	N/A	1.052575e0
A21233263b_b	Unknown	1.31e4	N/A	2.75	13.8	N/A	1.219934e0
A21233264b_b	Unknown	1.37e4	N/A	2.75	15.4	N/A	1.285843e0
A21233265b_b	Unknown	7.78e3	N/A	2.76	14.5	N/A	6.841462e-1
A21233266b_b	Unknown	1.33e5	N/A	2.75	81.3	N/A	1.338100e1
A21233267b_b	Unknown	8.58e3	N/A	2.76	12.3	N/A	7.647283e-1

Compound name: Miquelianin
Regression Equation: y = 9846.71313 x + 1047.96191 (r = 0.99901) (weighting: 1 / x)

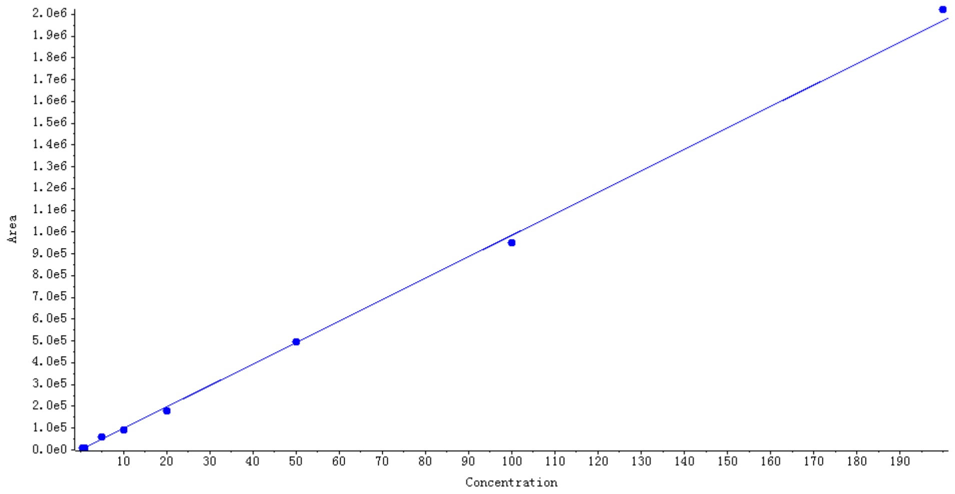

Peak Review

Blank
Miquelianin AREA:N/A S/N:N/A

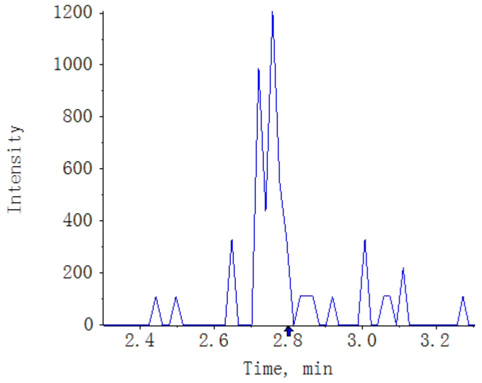

V1.0_MWMS_20211021_1
Miquelianin AREA:1.67e6
S/N:223.5

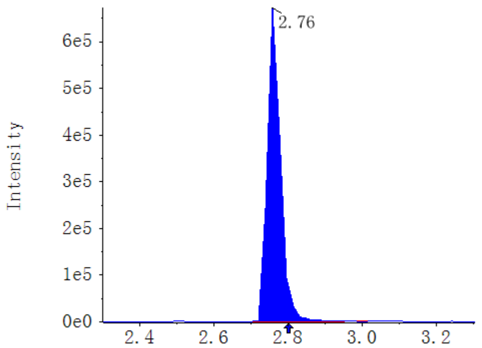

A21233250b_b
Miquelianin AREA:9.48e3 S/N:8.3

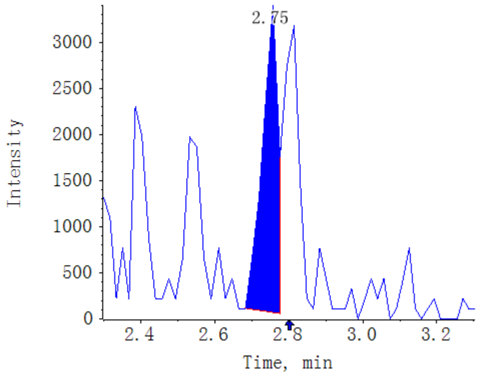

A21233251b_b
Miquelianin AREA:1.06e4
S/N:14.9

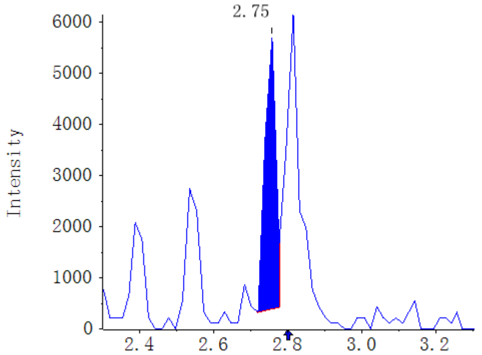

A21233252b_b
Miquelianin AREA:1.16e4
S/N:12.7

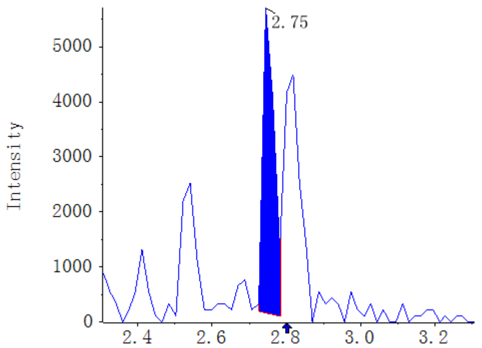

A21233253b_b
Miquelianin AREA:1.64e4
S/N:16.8

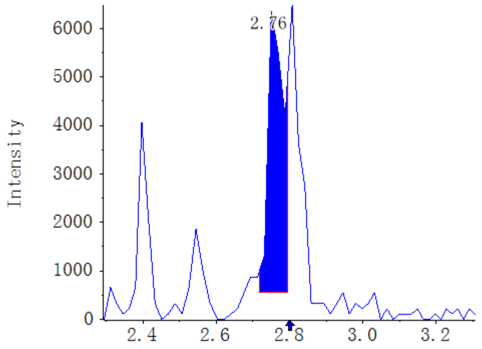

A21233254b_b
Miquelianin AREA:1.26e4
S/N:17.8

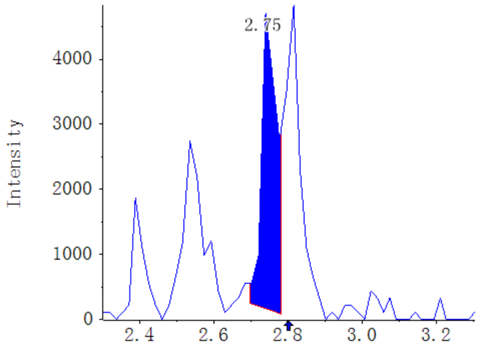

A21233255b_b
Miquelianin AREA:1.73e4
S/N:16.3

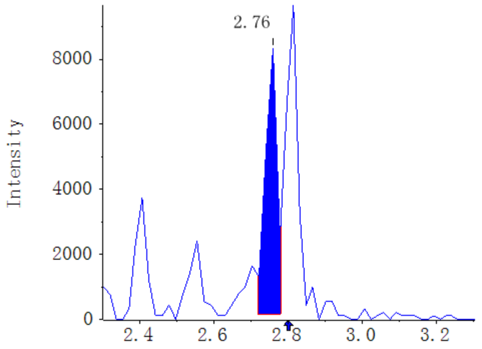

A21233256b_b
Miquelianin AREA:1.70e4
S/N:25.6

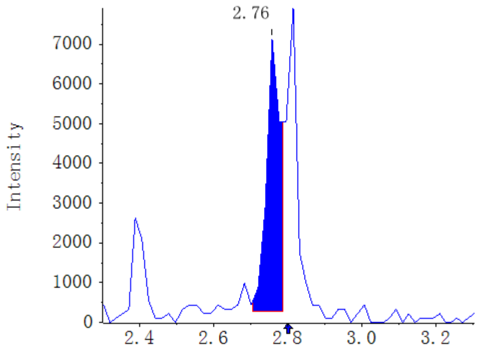

A21233257b_b
Miquelianin AREA:1.48e4
S/N:14.7

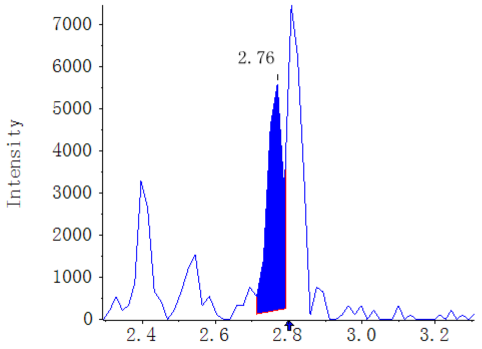

A21233258b_b
Miquelianin AREA:2.35e4
S/N:25.2

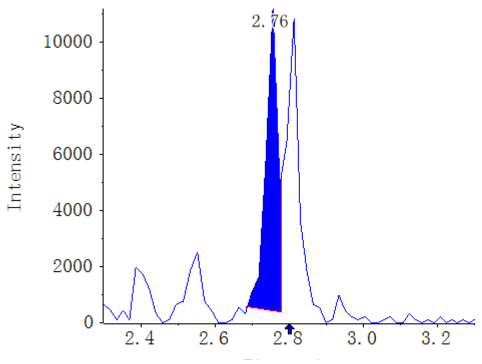

A21233259b_b
Miquelianin AREA:8.97e3
S/N:13.5

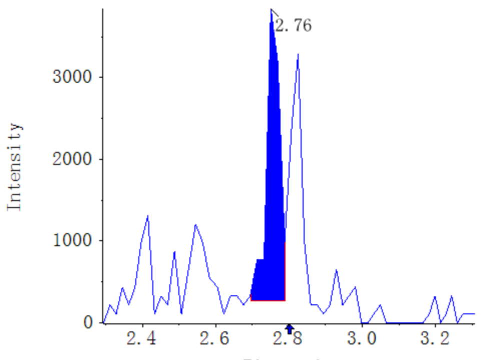

A21233260b_b
Miquelianin AREA:1.53e4
S/N:18.9

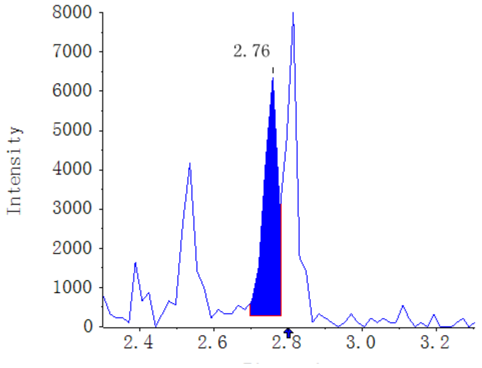

A21233261b_b
Miquelianin AREA:1.15e4
S/N:14.3

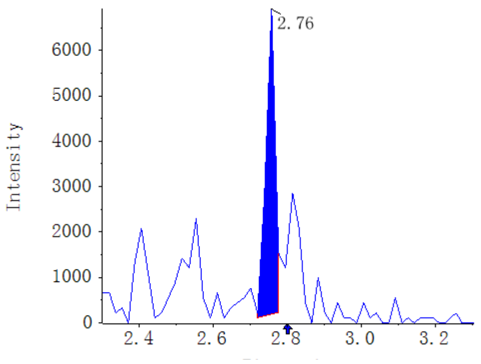

A21233262b_b
Miquelianin AREA:1.14e4
S/N:12.0

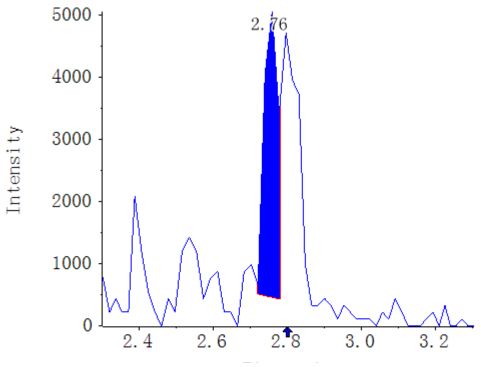

A21233263b_b
Miquelianin AREA:1.31e4
S/N:13.8

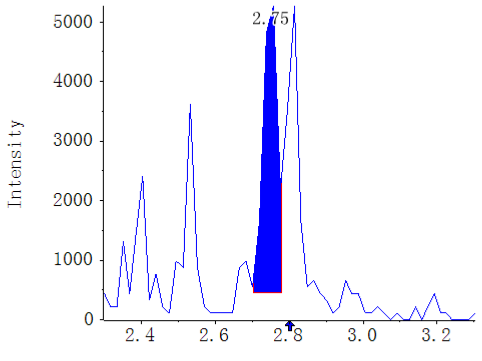

A21233264b_b
Miquelianin AREA:1.37e4
S/N:15.4

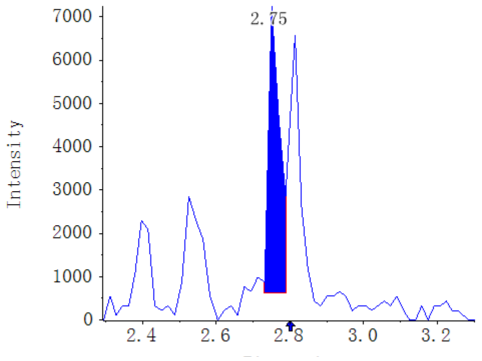

A21233265b_b
Miquelianin AREA:7.78e3
S/N:14.5

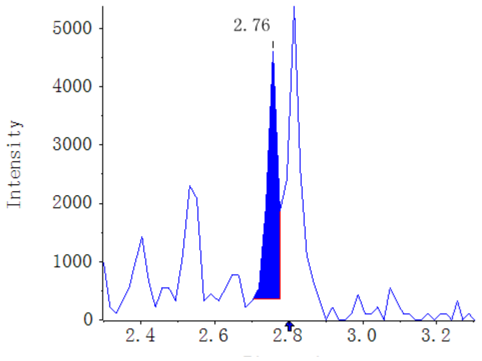

A21233266b_b
Miquelianin AREA:1.33e5
S/N:81.3

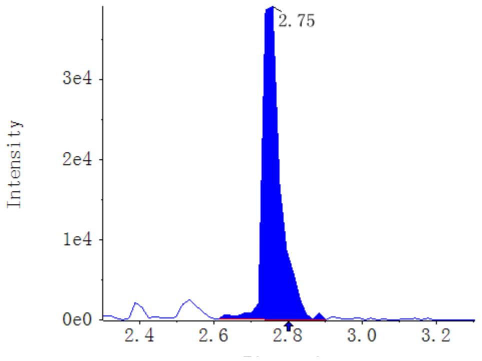

A21233267b_b
Miquelianin AREA:8.58e3
S/N:12.3

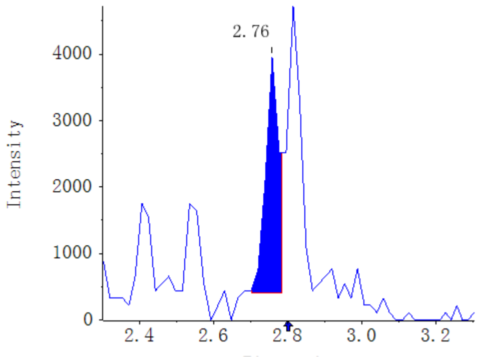

Result Table	MWXS-21-2101D-3_18_WH6500-5_A20-3_V1.0_TY_20211028	Algorithm Used	MQ4
Acquisition Method	Flavonoids_V1.0_WH6500-5_LT_20211025.dam	Instrument Name	QTRAP 6500+ Low Mass
Project	N/A	Analytes QTY	204:46

Compound name: Isorhamnetin (315.1 / 300.0)

Sample Name	Sample Type	Area (cps)	Is Area (cps)	RT (min)	S/N	Target Conc	Calculated Conc.()
STD_0.5nM	Standard	5.25e3	N/A	5.18	25.4	0.5000	4.166319e-1
STD_1nM	Standard	1.04e4	N/A	5.17	47.1	1.0000	8.931799e-1
STD_5nM	Standard	7.49e4	N/A	5.18	122.5	5.0000	6.866663e0
STD_10nM	Standard	1.07e5	N/A	5.18	159.4	10.0000	9.892007e0
STD_20nM	Standard	1.97e5	N/A	5.17	160.5	20.0000	1.820341e1
STD_50nM	Standard	5.43e5	N/A	5.18	210.0	50.0000	5.023156e1
STD_100nM	Standard	1.07e6	N/A	5.18	180.8	100.0000	9.924769e1
STD_200nM	Standard	2.17e6	N/A	5.17	199.8	200.0000	2.007489e2
STD_500nM	Standard	N/A	N/A	N/A	N/A	500.0000	N/A
STD_1000nM	Standard	N/A	N/A	N/A	N/A	1000.0000	N/A
STD_2000nM	Standard	N/A	N/A	N/A	N/A	2000.0000	N/A
V1.0_MW_RQC1_20211018	Quality Control	N/A	N/A	N/A	N/A	0.0000	N/A
Blank	Unknown	N/A	N/A	N/A	N/A	N/A	N/A
V1.0_MWMS_20211021_1	Unknown	1.85e6	N/A	5.19	207.0	N/A	1.713893e2
MWXS212101D3_R1	Quality Control	1.84e6	N/A	5.19	227.8	0.0000	1.700508e2
MWXS212101D3_R2	Quality Control	1.85e6	N/A	5.19	199.9	0.0000	1.713553e2
MWXS212101D3_R3	Quality Control	1.85e6	N/A	5.19	224.7	0.0000	1.713858e2
A21233250b_b	Unknown	N/A	N/A	N/A	N/A	N/A	N/A
A21233251b_b	Unknown	N/A	N/A	N/A	N/A	N/A	N/A
A21233252b_b	Unknown	N/A	N/A	N/A	N/A	N/A	N/A
A21233253b_b	Unknown	1.62e4	N/A	5.19	53.5	N/A	1.432749e0
A21233254b_b	Unknown	N/A	N/A	N/A	N/A	N/A	N/A
A21233255b_b	Unknown	N/A	N/A	N/A	N/A	N/A	N/A
A21233256b_b	Unknown	N/A	N/A	N/A	N/A	N/A	N/A
A21233257b_b	Unknown	N/A	N/A	N/A	N/A	N/A	N/A
A21233258b_b	Unknown	N/A	N/A	N/A	N/A	N/A	N/A
A21233259b_b	Unknown	N/A	N/A	N/A	N/A	N/A	N/A
A21233260b_b	Unknown	N/A	N/A	N/A	N/A	N/A	N/A
A21233261b_b	Unknown	N/A	N/A	N/A	N/A	N/A	N/A
A21233262b_b	Unknown	N/A	N/A	N/A	N/A	N/A	N/A
A21233263b_b	Unknown	N/A	N/A	N/A	N/A	N/A	N/A
A21233264b_b	Unknown	N/A	N/A	N/A	N/A	N/A	N/A
A21233265b_b	Unknown	N/A	N/A	N/A	N/A	N/A	N/A
A21233266b_b	Unknown	N/A	N/A	N/A	N/A	N/A	N/A
A21233267b_b	Unknown	N/A	N/A	N/A	N/A	N/A	N/A

Compound name: Isorhamnetin
Regression Equation: y = 10791.05651 x + 752.84498 (r = 0.99879) (weighting: 1 / x)

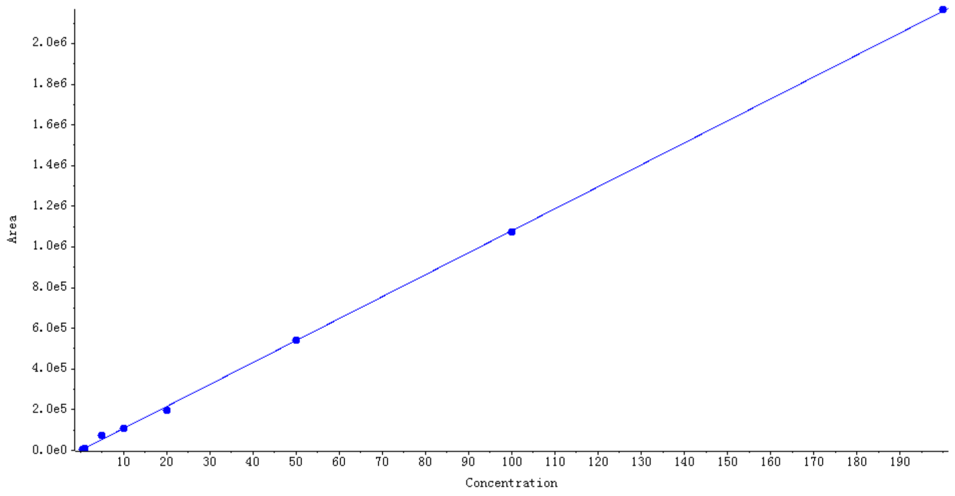

Peak Review

Blank
Isorhamnetin AREA:N/A S/N:N/A

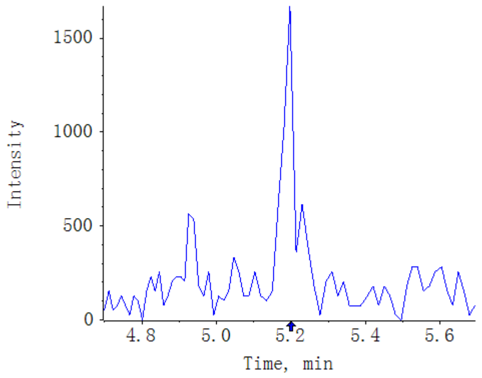

V1.0_MWMS_20211021_1
Isorhamnetin AREA:1.85e6 S/N:207.0

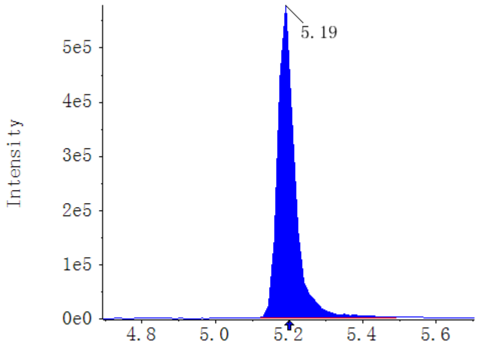

A21233250b_b
Isorhamnetin AREA:N/A S/N:N/A

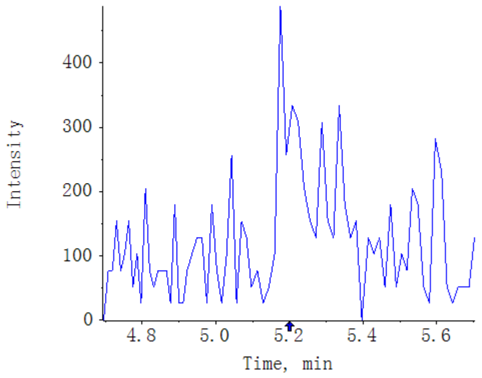

A21233251b_b
Isorhamnetin AREA:N/A S/N:N/A

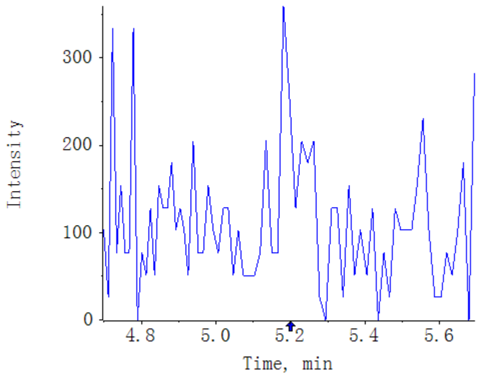

A21233252b_b
Isorhamnetin AREA:N/A S/N:N/A

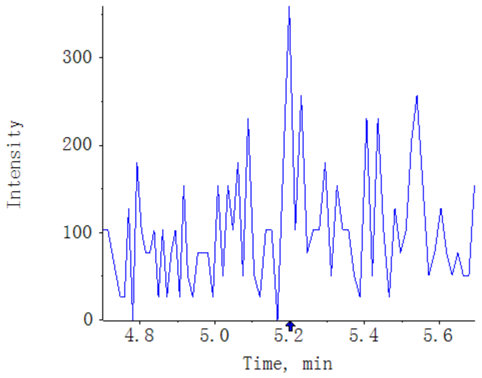

A21233253b_b
Isorhamnetin AREA:1.62e4 S/N:53.5

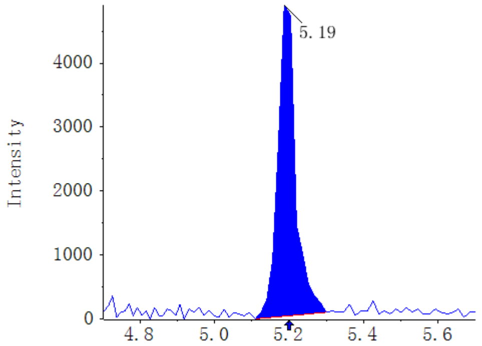

A21233254b_b
Isorhamnetin AREA:N/A S/N:N/A

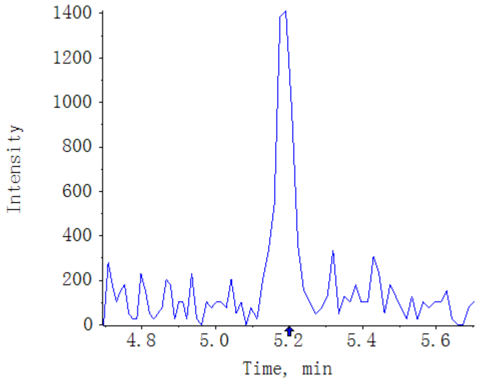

A21233255b_b
Isorhamnetin AREA:N/A S/N:N/A

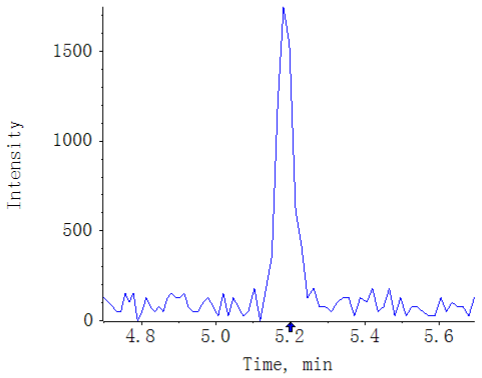

A21233256b_b
Isorhamnetin AREA:N/A S/N:N/A

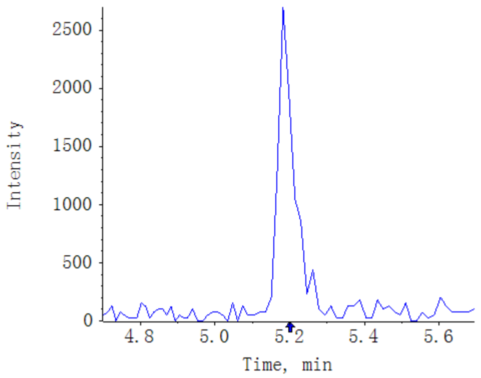

A21233257b_b
Isorhamnetin AREA:N/A S/N:N/A

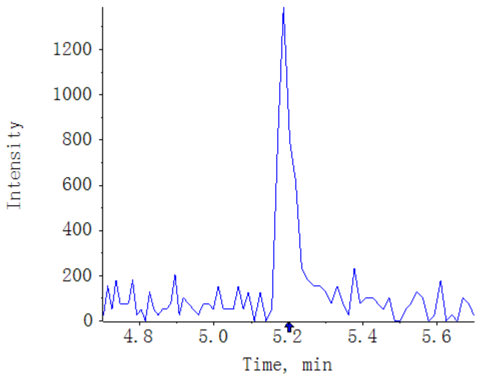

A21233258b_b
Isorhamnetin AREA:N/A S/N:N/A

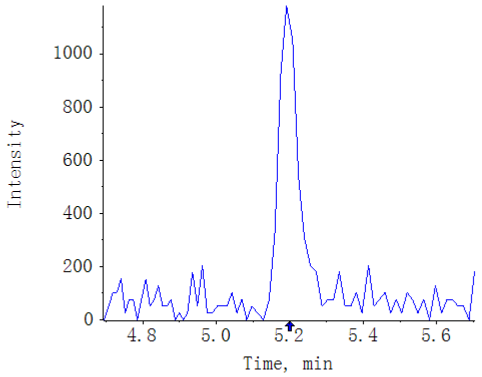

A21233259b_b
Isorhamnetin AREA:N/A S/N:N/A

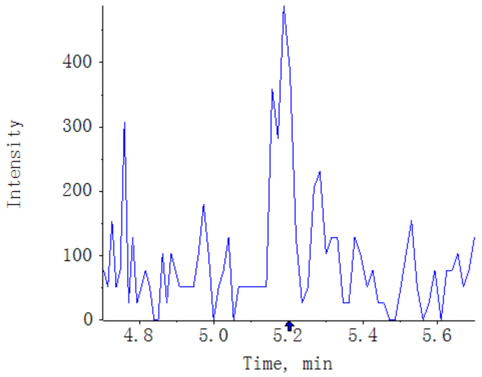

A21233260b_b
Isorhamnetin AREA:N/A S/N:N/A

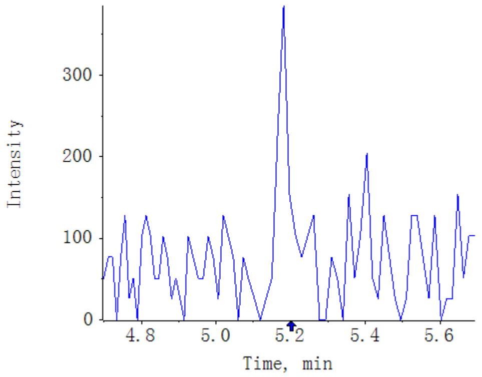

A21233261b_b
Isorhamnetin AREA:N/A S/N:N/A

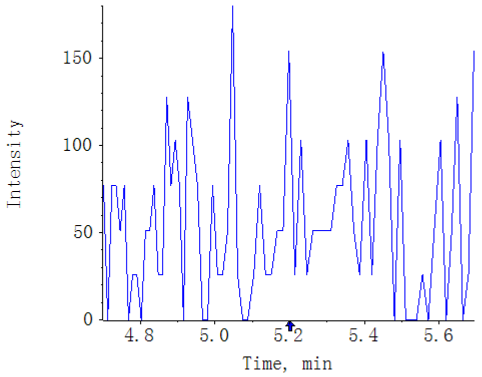

A21233262b_b
Isorhamnetin AREA:N/A S/N:N/A

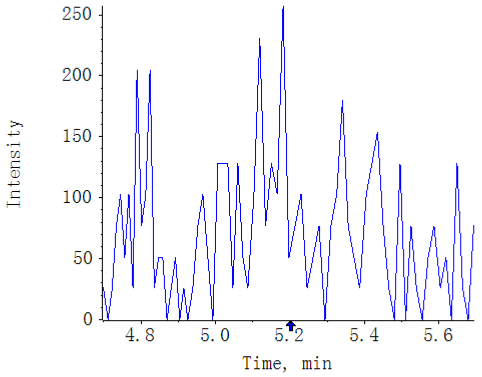

A21233263b_b
Isorhamnetin AREA:N/A S/N:N/A

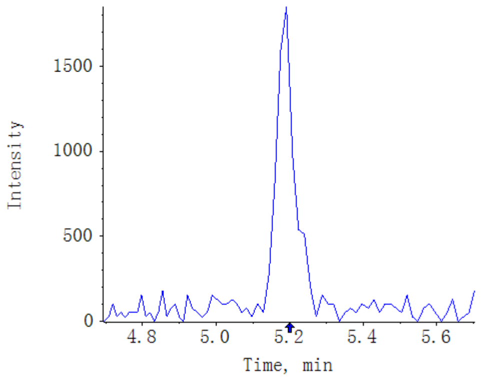

A21233264b_b
Isorhamnetin AREA:N/A S/N:N/A

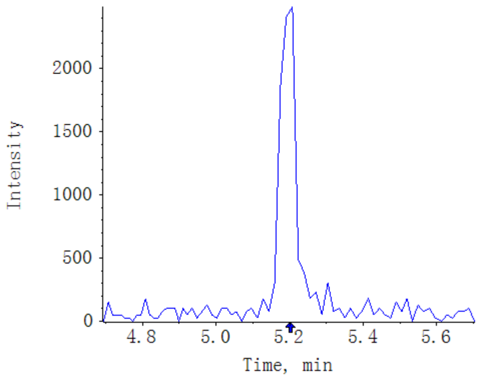

A21233265b_b
Isorhamnetin AREA:N/A S/N:N/A

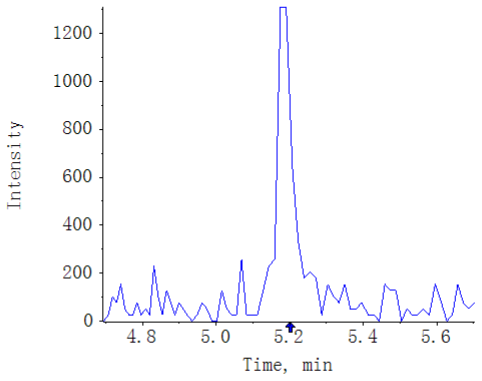

A21233266b_b
Isorhamnetin AREA:N/A S/N:N/A

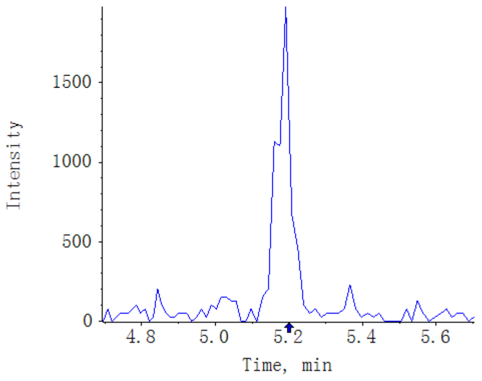

A21233267b_b
Isorhamnetin AREA:N/A S/N:N/A

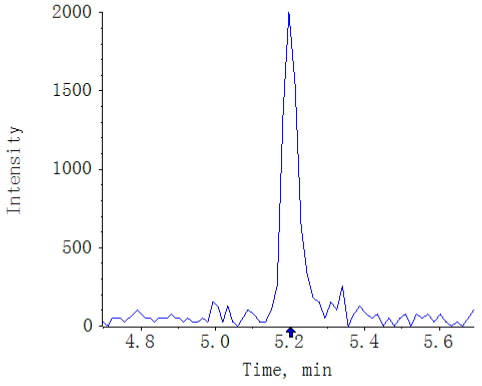

Result Table	MWXS-21-2101D-3_18_WH6500-5_A20-3_V1.0_TY_20211028	Algorithm Used	MQ4
Acquisition Method	Flavonoids_V1.0_WH6500-5_LT_20211025.dam	Instrument Name	QTRAP 6500+ Low Mass
Project	N/A	Analytes QTY	204:4

Compound name: Genistein (269.0 / 133.0)

Sample Name	Sample Type	Area (cps)	Is Area (cps)	RT (min)	S/N	Target Conc	Calculated Conc.()
STD_0.5nM	Standard	2.80e3	N/A	4.89	10.4	0.5000	5.937335e-1
STD_1nM	Standard	4.94e3	N/A	4.89	16.0	1.0000	9.820227e-1
STD_5nM	Standard	3.40e4	N/A	4.89	55.1	5.0000	6.247274e0
STD_10nM	Standard	5.16e4	N/A	4.89	63.2	10.0000	9.451783e0
STD_20nM	Standard	9.99e4	N/A	4.88	105.0	20.0000	1.821503e1
STD_50nM	Standard	2.47e5	N/A	4.89	122.6	50.0000	4.488963e1
STD_100nM	Standard	4.97e5	N/A	4.90	85.3	100.0000	9.022747e1
STD_200nM	Standard	9.06e5	N/A	4.89	100.5	200.0000	1.644241e2
STD_500nM	Standard	3.04e6	N/A	4.89	105.3	500.0000	5.514690e2
STD_1000nM	Standard	N/A	N/A	N/A	N/A	1000.0000	N/A
STD_2000nM	Standard	N/A	N/A	N/A	N/A	2000.0000	N/A
V1.0_MW_RQC1_20211018	Quality Control	N/A	N/A	N/A	N/A	0.0000	N/A
Blank	Unknown	N/A	N/A	N/A	N/A	N/A	N/A
V1.0_MWMS_20211021_1	Unknown	7.34e5	N/A	4.90	105.3	N/A	1.331472e2
MWXS212101D3_R1	Quality Control	7.34e5	N/A	4.90	111.1	0.0000	1.331452e2
MWXS212101D3_R2	Quality Control	7.23e5	N/A	4.90	106.6	0.0000	1.312784e2
MWXS212101D3_R3	Quality Control	7.06e5	N/A	4.90	113.2	0.0000	1.282239e2
A21233250b_b	Unknown	N/A	N/A	N/A	N/A	N/A	N/A
A21233251b_b	Unknown	N/A	N/A	N/A	N/A	N/A	N/A
A21233252b_b	Unknown	N/A	N/A	N/A	N/A	N/A	N/A
A21233253b_b	Unknown	6.94e3	N/A	4.91	19.7	N/A	1.344007e0
A21233254b_b	Unknown	1.02e4	N/A	4.90	30.4	N/A	1.939017e0
A21233255b_b	Unknown	1.27e4	N/A	4.90	38.0	N/A	2.390874e0
A21233256b_b	Unknown	1.42e4	N/A	4.91	37.5	N/A	2.664184e0
A21233257b_b	Unknown	1.12e4	N/A	4.91	39.1	N/A	2.110189e0
A21233258b_b	Unknown	1.80e4	N/A	4.91	64.7	N/A	3.351584e0
A21233259b_b	Unknown	N/A	N/A	N/A	N/A	N/A	N/A
A21233260b_b	Unknown	N/A	N/A	N/A	N/A	N/A	N/A
A21233261b_b	Unknown	N/A	N/A	N/A	N/A	N/A	N/A
A21233262b_b	Unknown	3.01e3	N/A	4.90	16.7	N/A	6.314724e-1
A21233263b_b	Unknown	1.33e4	N/A	4.90	31.0	N/A	2.498074e0
A21233264b_b	Unknown	1.03e4	N/A	4.91	28.5	N/A	1.947256e0
A21233265b_b	Unknown	3.27e4	N/A	4.91	63.8	N/A	6.014907e0
A21233266b_b	Unknown	1.58e4	N/A	4.90	43.2	N/A	2.948056e0
A21233267b_b	Unknown	2.12e4	N/A	4.91	55.5	N/A	3.932252e0

Compound name: Genistein
Regression Equation: y = 5513.24728 x + -474.44388 (r = 0.99220) (weighting: 1 / x)

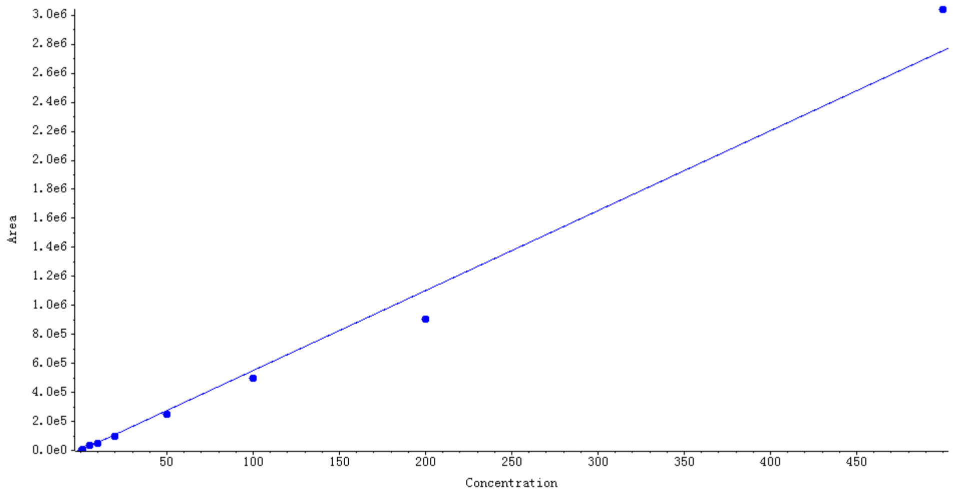

Peak Review

Blank
Genistein AREA:N/A S/N:N/A

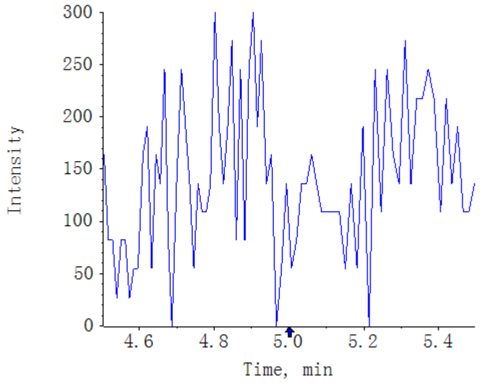

V1.0_MWMS_20211021_1
Genistein AREA:7.34e5 S/N:105.3

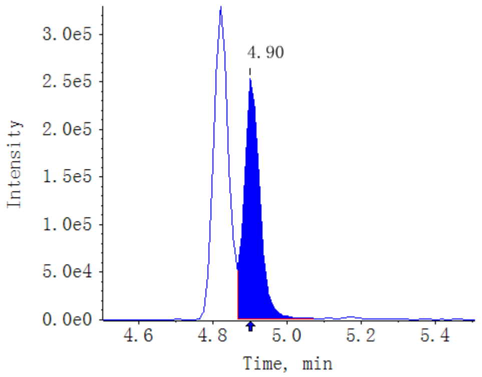

A21233250b_b
Genistein AREA:N/A S/N:N/A

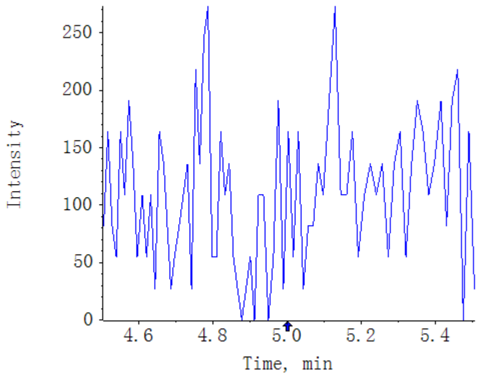

A21233251b_b
Genistein AREA:N/A S/N:N/A

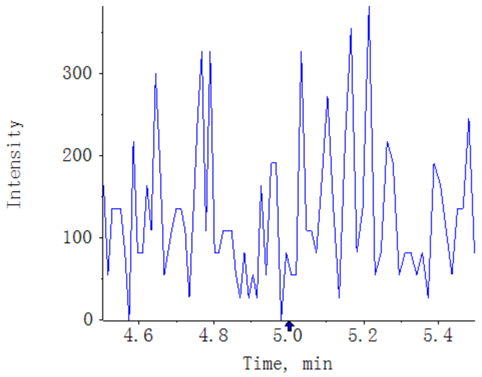

A21233252b_b
Genistein AREA:N/A S/N:N/A

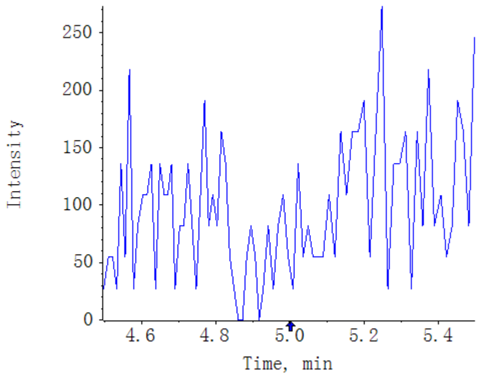

A21233253b_b
Genistein AREA:6.94e3 S/N:19.7

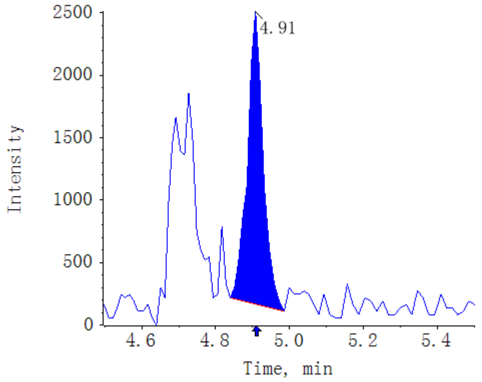

A21233254b_b
Genistein AREA:1.02e4 S/N:30.4

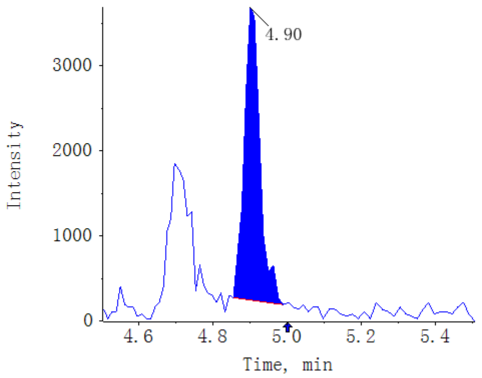

A21233255b_b
Genistein AREA:1.27e4 S/N:38.0

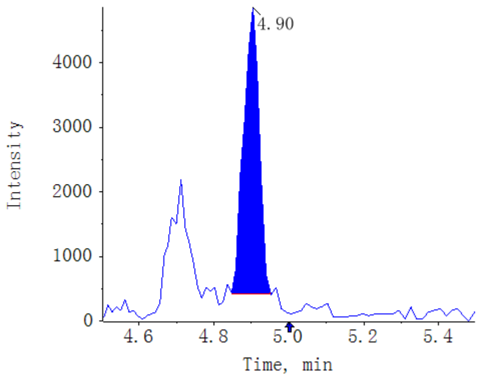

A21233256b_b
Genistein AREA:1.42e4 S/N:37.5

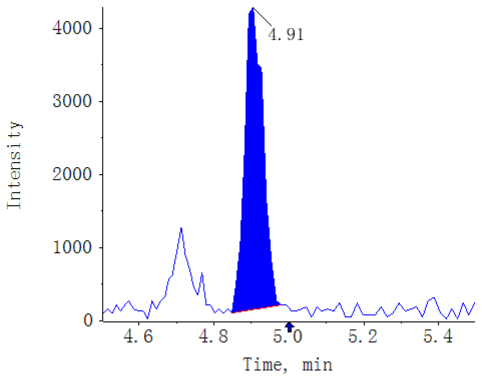

A21233257b_b
Genistein AREA:1.12e4 S/N:39.1

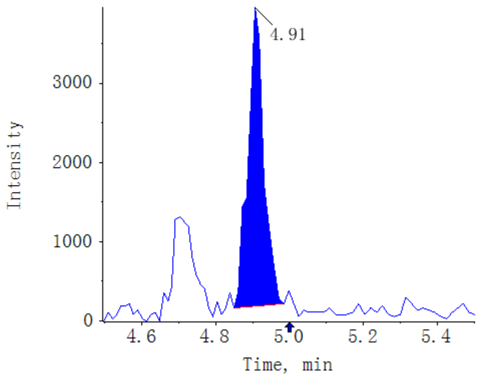

A21233258b_b
Genistein AREA:1.80e4 S/N:64.7

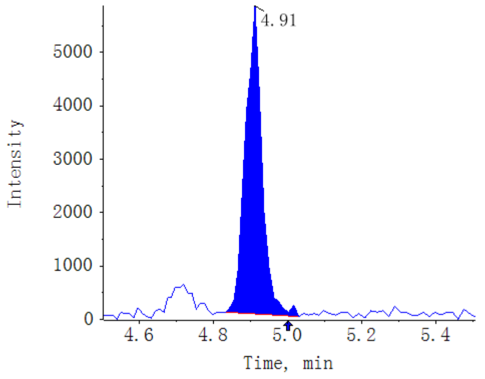

A21233259b_b
Genistein AREA:N/A S/N:N/A

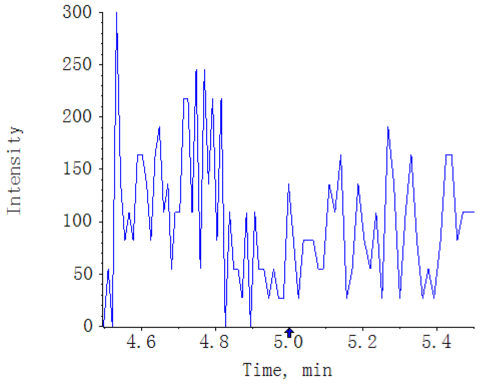

A21233260b_b
Genistein AREA:N/A S/N:N/A

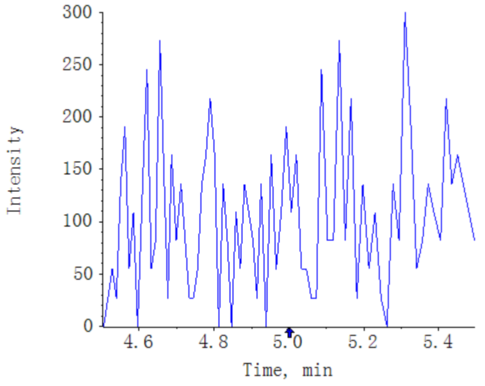

A21233261b_b
Genistein AREA:N/A S/N:N/A

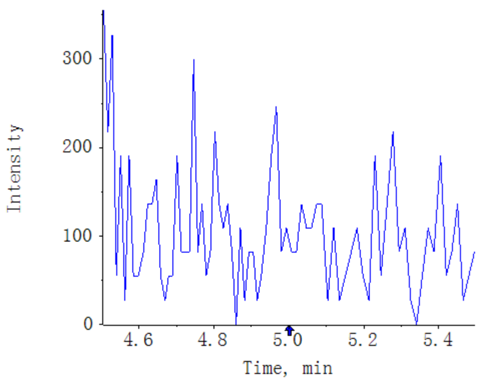

A21233262b_b
Genistein AREA:3.01e3 S/N:16.7

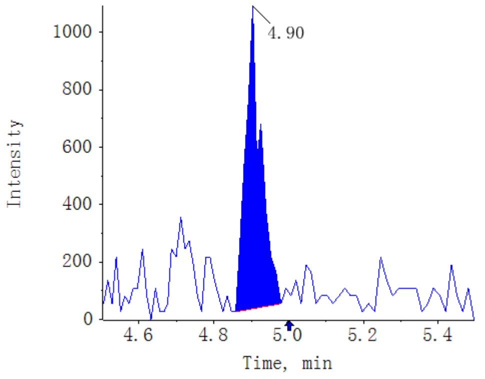

A21233263b_b
Genistein AREA:1.33e4 S/N:31.0

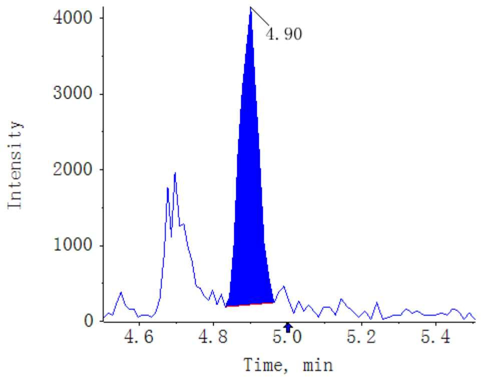

A21233264b_b
Genistein AREA:1.03e4 S/N:28.5

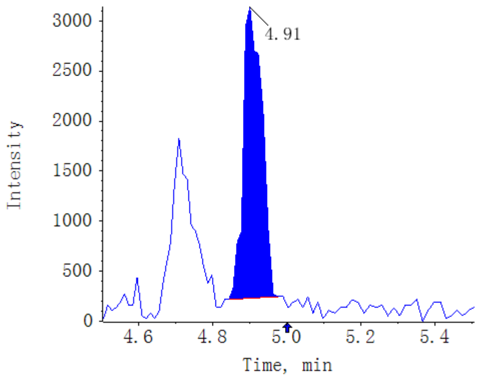

A21233265b_b
Genistein AREA:3.27e4 S/N:63.8

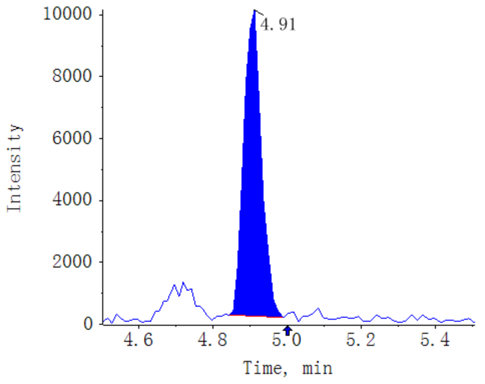

A21233266b_b
Genistein AREA:1.58e4 S/N:43.2

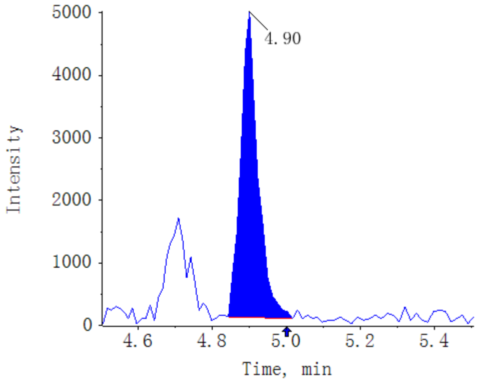

A21233267b_b
Genistein AREA:2.12e4 S/N:55.5

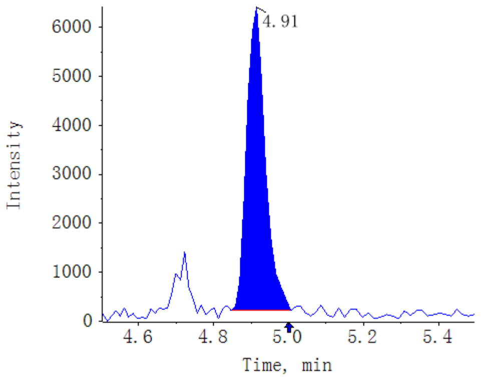

Result Table	MWXS-21-2101D-3_18_WH6500-5_A20-3_V1.0_TY_20211028	Algorithm Used	MQ4
Acquisition Method	Flavonoids_V1.0_WH6500-5_LT_20211025.dam	Instrument Name	QTRAP 6500+ Low Mass
Project	N/A	Analytes QTY	204:123

Compound name: Ononin (475.1 / 267.1)

Sample Name	Sample Type	Area (cps)	Is Area (cps)	RT (min)	S/N	Target Conc	Calculated Conc.()
STD_0.5nM	Standard	2.58e3	N/A	3.57	25.8	0.5000	5.022633e-1
STD_1nM	Standard	4.13e3	N/A	3.55	39.1	1.0000	7.521542e-1
STD_5nM	Standard	4.34e4	N/A	3.56	128.9	5.0000	7.088093e0
STD_10nM	Standard	5.81e4	N/A	3.56	205.0	10.0000	9.450087e0
STD_20nM	Standard	1.16e5	N/A	3.55	298.1	20.0000	1.882459e1
STD_50nM	Standard	2.93e5	N/A	3.56	440.3	50.0000	4.731097e1
STD_100nM	Standard	5.95e5	N/A	3.56	467.1	100.0000	9.607673e1
STD_200nM	Standard	1.28e6	N/A	3.55	630.4	200.0000	2.064951e2
STD_500nM	Standard	N/A	N/A	N/A	N/A	500.0000	N/A
STD_1000nM	Standard	N/A	N/A	N/A	N/A	1000.0000	N/A
STD_2000nM	Standard	N/A	N/A	N/A	N/A	2000.0000	N/A
V1.0_MW_RQC1_20211018	Quality Control	N/A	N/A	N/A	N/A	0.0000	N/A
Blank	Unknown	N/A	N/A	N/A	N/A	N/A	N/A
V1.0_MWMS_20211021_1	Unknown	9.31e5	N/A	3.57	544.6	N/A	1.503145e2
MWXS212101D3_R1	Quality Control	9.78e5	N/A	3.56	608.3	0.0000	1.578493e2
MWXS212101D3_R2	Quality Control	9.96e5	N/A	3.57	750.5	0.0000	1.607358e2
MWXS212101D3_R3	Quality Control	9.33e5	N/A	3.57	703.6	0.0000	1.505144e2
A21233250b_b	Unknown	5.29e4	N/A	3.51	16.1	N/A	8.613193e0
A21233251b_b	Unknown	1.12e4	N/A	3.51	8.6	N/A	1.900289e0
A21233252b_b	Unknown	N/A	N/A	N/A	N/A	N/A	N/A
A21233253b_b	Unknown	N/A	N/A	N/A	N/A	N/A	N/A
A21233254b_b	Unknown	N/A	N/A	N/A	N/A	N/A	N/A
A21233255b_b	Unknown	N/A	N/A	N/A	N/A	N/A	N/A
A21233256b_b	Unknown	N/A	N/A	N/A	N/A	N/A	N/A
A21233257b_b	Unknown	N/A	N/A	N/A	N/A	N/A	N/A
A21233258b_b	Unknown	N/A	N/A	N/A	N/A	N/A	N/A
A21233259b_b	Unknown	N/A	N/A	N/A	N/A	N/A	N/A
A21233260b_b	Unknown	N/A	N/A	N/A	N/A	N/A	N/A
A21233261b_b	Unknown	N/A	N/A	N/A	N/A	N/A	N/A
A21233262b_b	Unknown	N/A	N/A	N/A	N/A	N/A	N/A
A21233263b_b	Unknown	N/A	N/A	N/A	N/A	N/A	N/A
A21233264b_b	Unknown	N/A	N/A	N/A	N/A	N/A	N/A
A21233265b_b	Unknown	N/A	N/A	N/A	N/A	N/A	N/A
A21233266b_b	Unknown	N/A	N/A	N/A	N/A	N/A	N/A
A21233267b_b	Unknown	N/A	N/A	N/A	N/A	N/A	N/A

Compound name: Ononin
Regression Equation: y = 6199.63508 x + -533.36248 (r = 0.99791) (weighting: 1 / x)

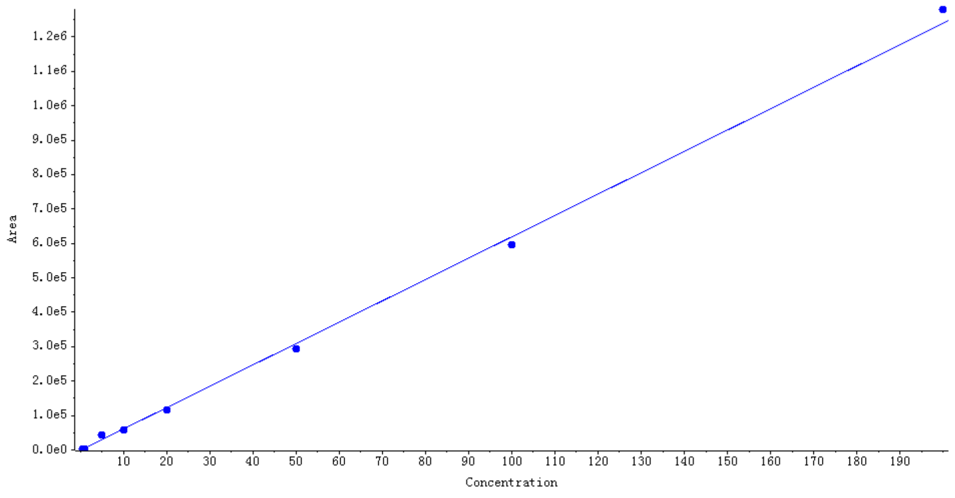

Peak Review

Blank
Ononin AREA:N/A S/N:N/A

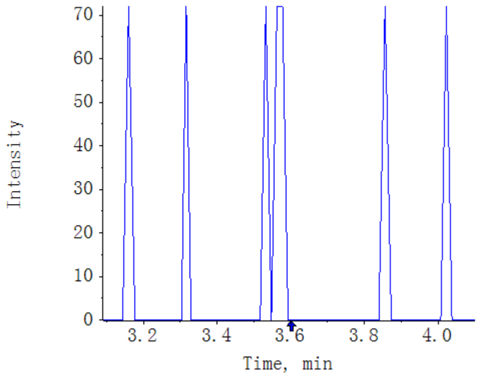

V1.0_MWMS_20211021_1
Ononin AREA:9.31e5 S/N:544.6

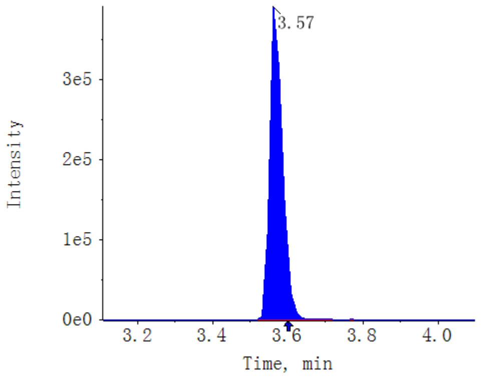

A21233250b_b
Ononin AREA:5.29e4 S/N:16.1

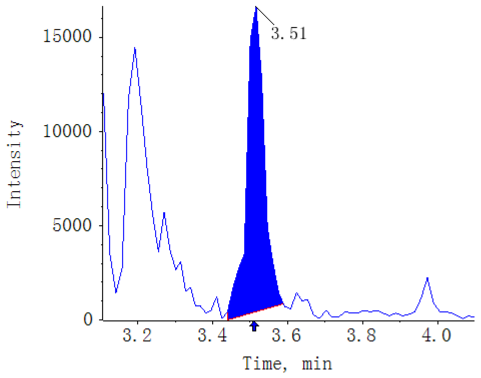

A21233251b_b
Ononin AREA:1.12e4 S/N:8.6

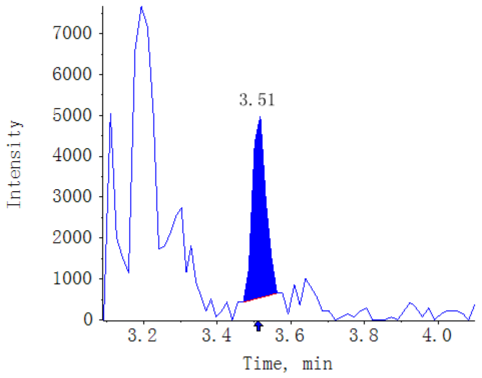

A21233252b_b
Ononin AREA:N/A S/N:N/A

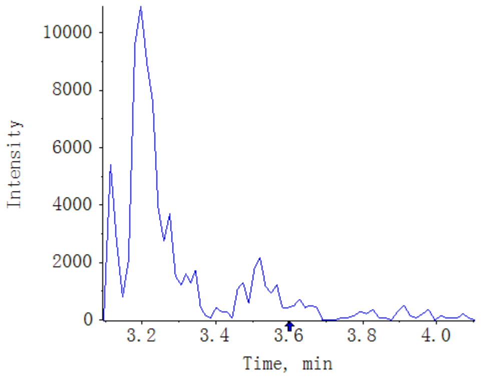

A21233253b_b
Ononin AREA:N/A S/N:N/A

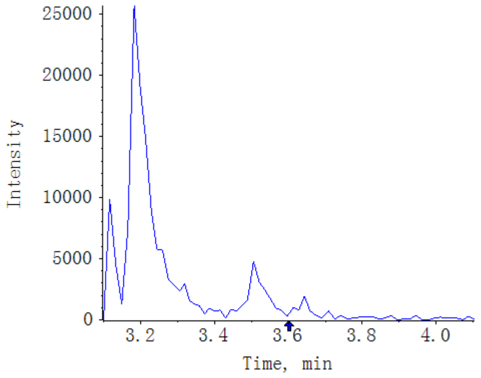

A21233254b_b
Ononin AREA:N/A S/N:N/A

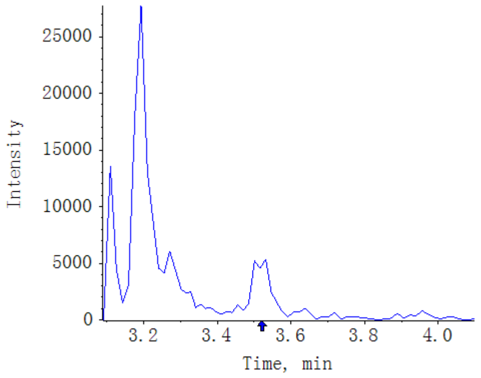

A21233255b_b
Ononin AREA:N/A S/N:N/A

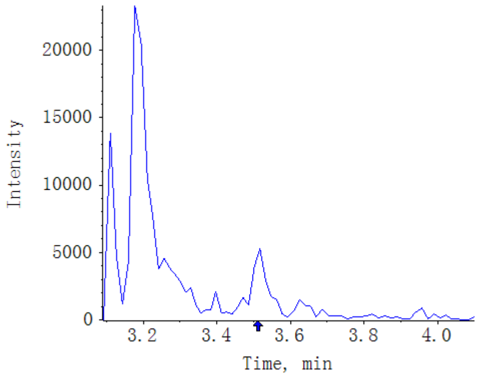

A21233256b_b
Ononin AREA:N/A S/N:N/A

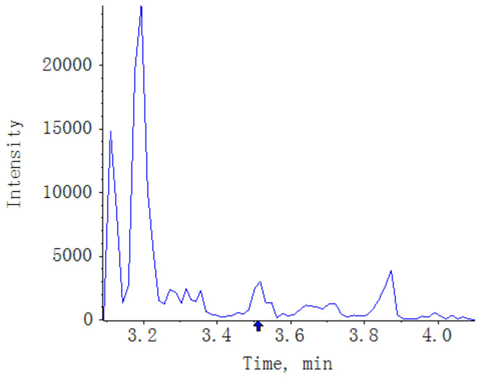

A21233257b_b
Ononin AREA:N/A S/N:N/A

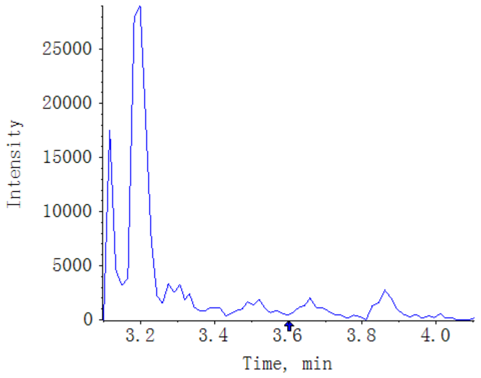

A21233258b_b
Ononin AREA:N/A S/N:N/A

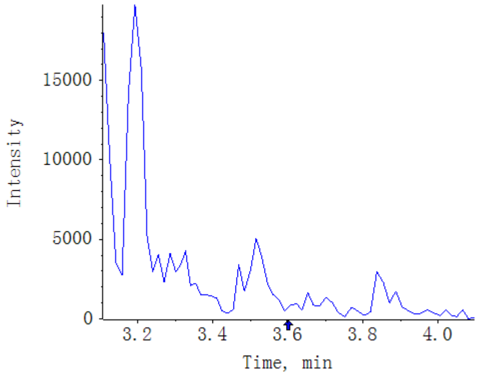

A21233259b_b
Ononin AREA:N/A S/N:N/A

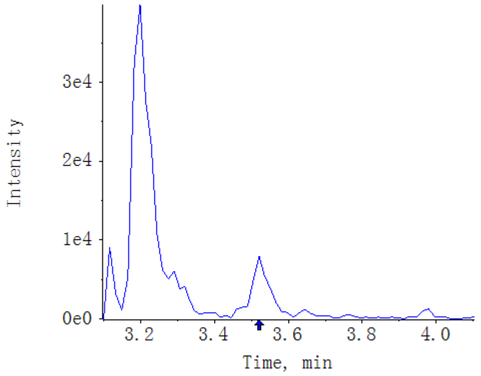

A21233260b_b
Ononin AREA:N/A S/N:N/A

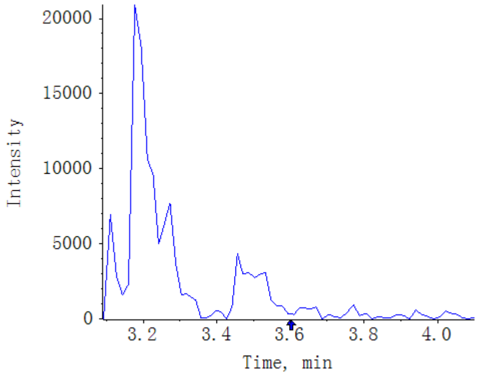

A21233261b_b
Ononin AREA:N/A S/N:N/A

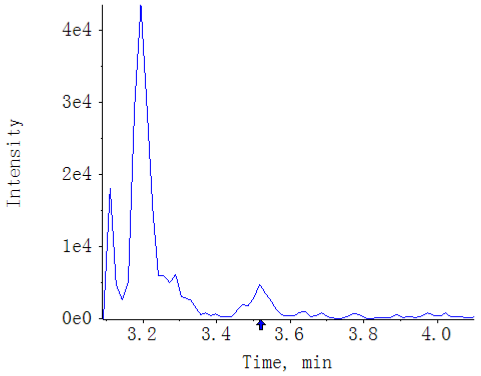

A21233262b_b
Ononin AREA:N/A S/N:N/A

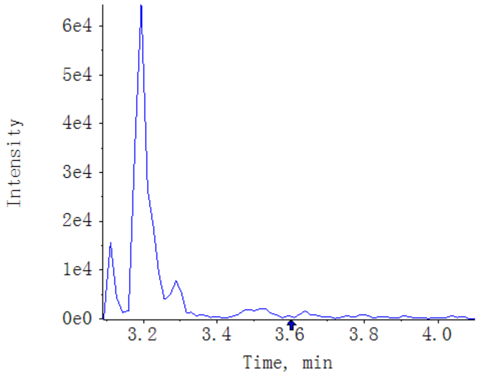

A21233263b_b
Ononin AREA:N/A S/N:N/A

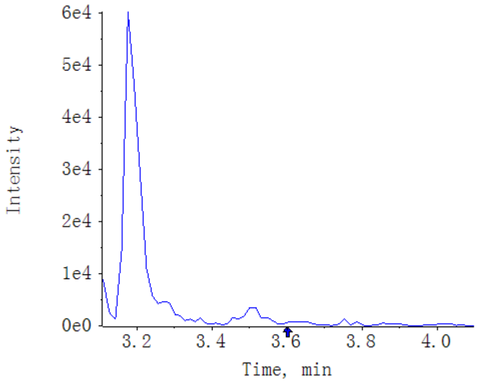

A21233264b_b
Ononin AREA:N/A S/N:N/A

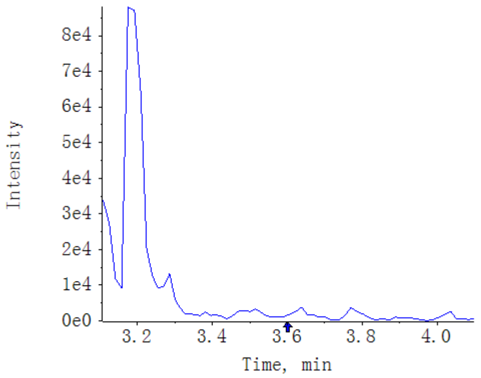

A21233265b_b
Ononin AREA:N/A S/N:N/A

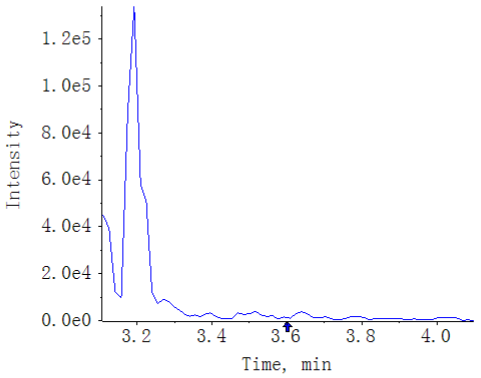

A21233266b_b
Ononin AREA:N/A S/N:N/A

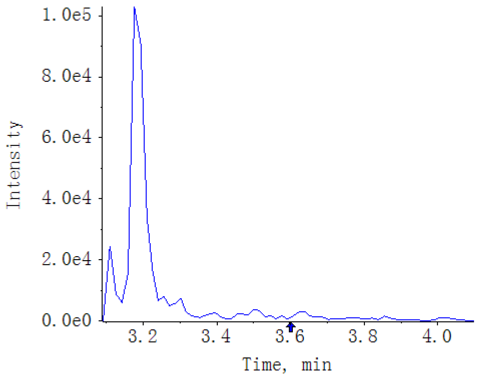

A21233267b_b
Ononin AREA:N/A S/N:N/A

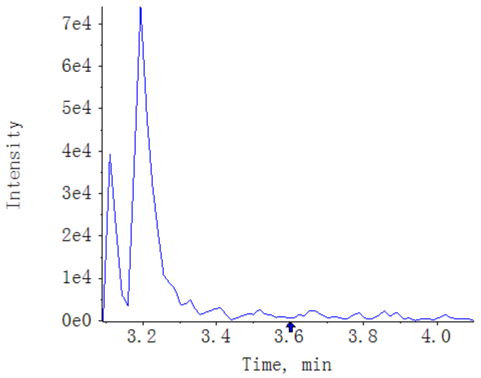

Supplement: Supplementary file 1 [file foods-12-04445-s001.zip › supplementary files/Supplementary File S3 - Calibration curves and quantitative details of the fifty Flavonoids.pdf]
